# Extensive TDDFT Benchmark Study of the Resonance Raman Spectra of Lumiflavin

**DOI:** 10.1002/jcc.70229

**Published:** 2025-10-04

**Authors:** Prokopis C. Andrikopoulos, Heba Halimeh

**Affiliations:** ^1^ First Faculty of Medicine Charles University, BIOCEV Vestec Czech Republic; ^2^ Institute of Biotechnology of the Czech Academy of Sciences, BIOCEV Vestec Czech Republic; ^3^ Rhine‐Waal University of Applied Sciences Kleve Germany

**Keywords:** benchmark, BLUF, flavins, LOV domains, resonance Raman, TDDFT

## Abstract

An extensive computational TDDFT resonance Raman study of lumiflavin is presented including 42 DFT functionals, benchmarked against the experimental Evolution Associated Spectra (EAS) of the equilibrated S_1_ and T_1_ states of FMN published earlier. Initially, off‐resonance spectra were computed, yielding adequate agreement, and fine‐tuning was achieved with the inclusion of specific frequency scaling factors. Since the experimental EAS were obtained under resonance for the singlet and near‐resonance for the triplet state, the subsequent inclusion of resonance effects in the calculations improved the correlation for most functionals. Their evaluation according to specific criteria narrowed down the choice to HCTH, OLYP, and TPSSh. Among the included criteria were the percent error of the 0–0 transitions, the quantification of the increase/decrease in correlation due to the addition of resonance enhancements, and the reproduction of the singlet‐triplet peak shifts. Owing to the extensive data set, valuable insights were gained to assist similar studies.

## Graphical abstract

An extensive computational TDDFT resonance Raman study of lumiflavin is presented including forty‐two different DFT functionals, benchmarked against the experimental spectra of FMN. The inclusion of resonance enhancements improved the agreement for most functionals. Following evaluation according to specific criteria, HCTH, OLYP and TPSSh emerged as the most accurate functionals. In the inset picture, the dependence of the rR intensity on the incident energy is illustrated for the OLYP functional.

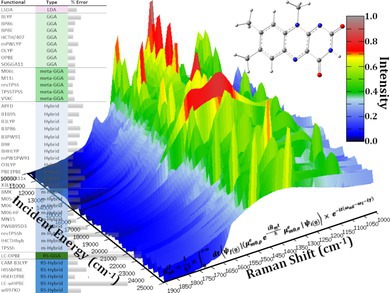

## 1 Introduction

When embarking on the computational vibrational study of systems under resonance conditions, the choice of the level of theory among the plethora of DFT functionals can be quite perplexing; the study presented here aims to address this issue.

**SCHEME 1:**
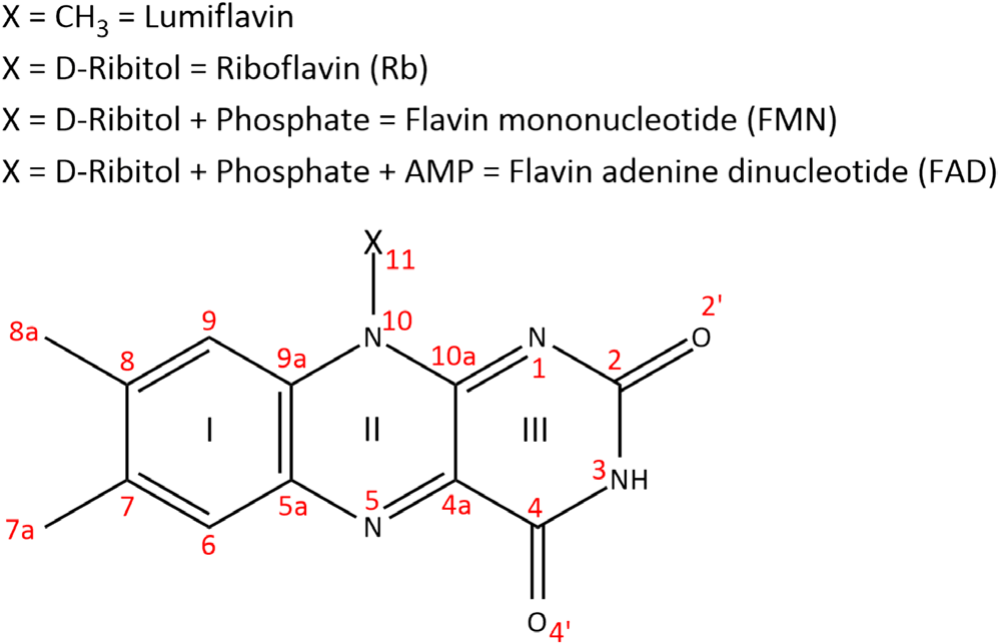
Numbering of atoms (in red), rings (in Latin numbers) and substitutions (X) in the family of flavin chromophores. Depending on the X substitution at N_10_, a different member of the family is defined starting from the simplest lumiflavin, up to Flavin adenine dinucleotide, FAD.

Flavins are a family of isoalloxazine‐containing chromophores that are present as co‐factors in many photosensitive proteins [1, 2, 3]. In addition to their essential roles in photoproteins, flavins are widespread in biology as components of redox‐active enzymes [4] and transcription factors [5]. As redox‐active enzymes and sensors, flavin‐dependent proteins contribute to pathogen metabolism, immune evasion, and oxidative stress responses, while also influencing host defense mechanisms [6]. Members of the family include lumiflavin, riboflavin (vitamin B_2_), flavin mononucleotide (FMN) and flavin adenine dinucleotide (FAD). The difference between the members depends on the substitution of the N_10_ atom of the isoalloxazine ring, from methyl in lumiflavinup to a combination of a sugar chain, phosphate group, and nucleotide base in FAD (Scheme 1). Lumiflavin in particular is broadly used as a computational analogue for the more substituted members of the family in many photochemical [7, 8, 9, 10], as well as in benchmark studies [11].

To study experimentally chromophore systems in solution, or embedded in photoproteins, one of the techniques of choice is time‐resolved vibrational spectroscopy. Transient Absorption (TA), ultrafast transient Infrared Spectroscopy (TRIR), resonance Raman (rR) and other time‐resolved spectroscopic techniques offer a wealth of information on the light response of photochemical and photobiological systems [12, 13, 14, 15]. Femtosecond Stimulated Raman Spectroscopy in particular (FSRS) [16], has unique advantages owed to its ultrafast resolution and tunability, which entails the targeting of specific electronic transitions of the target chromophore, under resonance conditions. When these conditions are met, the obtained spectra exhibit fewer features than their off‐resonance counterparts, are free from contamination from non‐resonant signals, and display enhanced signal strength [17, 18, 19]. Notwithstanding the above advantages, Raman spectra obtained under resonance pose an additional challenge in their interpretation. In broad terms, the normal modes pertaining to the electronic transitions in tune with the incident light are promoted in their intensities, while other signals are suppressed, and this ought to be taken into account in their analysis.

Theoretical calculations have proven to be indispensable, not only in the interpretation of time‐resolved spectroscopy [13, 20, 21, 22], but also in aiding the design of photobiological molecules with specific properties [9, 23]. For resonance Raman spectroscopy in particular, the time dependent theory of resonance Raman spectroscopy (TD‐RR) is currently implemented in computational chemistry codes, including Herzberg‐Teller contributions and solvent and anharmonic effects [24]. To compute the spectra, two different approaches exist, either requiring the optimization of the resonant state or utilizing only the gradients of the excitations in the geometry of the reference state, yielding similar results [25]. Several computational studies have appeared recently in the literature, tackling resonance Raman calculations of various systems ranging from thiophene derivatives to fluorescent protein chromophores, often including several DFT functionals in their studies [25, 26, 27, 28, 29, 30]. The popular density functional theory (DFT) functional B3LYP [31, 32] is used extensively for the computation of flavin‐containing systems, due to the accurate prediction of the excitation energies [12, 20, 21, 33, 34]. However, as Green *et al.* demonstrated [35], the calculated B3LYP off‐Resonance spectra of lumiflavin correlated far better with the experimental FSRS—which were obtained under resonance conditions—than the calculated resonance Raman spectra.

In this study, we present a comprehensive approach to evaluate the off‐resonance (offR) and resonance Raman spectra (rR) of a plethora of DFT functionals against the FSRS experimental spectrum of FMN. We included a total of 42 DFT functionals, with lumiflavin as the target compound, benchmarked against the experimental Evolution Associated Spectra (EAS) of FMN [19], obtained under resonance conditions, and assigned to the equilibrated first excited singlet and triplet states (S_1_ and T_1_). The choice of a modest polarized double‐ζ basis set for the majority of the calculations allows the findings presented here to be applicable for larger cluster or QM/MM studies of lumiflavin embedded in a protein environment, where a higher basis set might not be affordable. The functionals were scored against: (i) the predicted 0–0 transition energies, (ii) the percent error of the correlation between experimental and rR‐calculated spectra, (iii) the difference in percent errors between the offR and rR correlations—indicating whether the agreement improved or deteriorated with the addition of resonance, (iv) the predicted rR intensity of the strongest experimental peak in the fingerprint region at 1498 cm^−1^ (1514 cm^−1^ for the triplets) and finally, (v) the visual inspection of the spectra, to determine whether the experimental/theoretical spectral profiles are compatible, facilitating the assignment. An additional criterion is introduced in the triplet evaluation: whether the functionals can reproduce the singlet‐triplet peak shifts evidenced between the experimental EAS.

The aim of this benchmarking effort is not to delve deep into the particulars of each DFT functional, but instead to narrow down the chosen large set of DFT functionals to a smaller set that can be used to describe more faithfully flavin‐related systems with resonance Raman calculations. The insights gained from this benchmark study can hopefully be transferable to other chromophore/photoprotein systems.

## 2 Computational Details

All calculations were performed with the Gaussian program (G16 Rev. C.01) [36]. A total of 42 DFT functionals were utilized, as shown in Table S1, together with the percentage of Hartree Fock exchange, a short description, and references to original publications. The empirical dispersion correction parameters employed in this study are shown in Table S2 [37, 38]. Empirical corrections were included for all but three of the functionals, namely SOGGA11, VSXC, and LSDA (SWVN). Regarding the frequency scaling factors, no comprehensive study exists in the literature for excited states. Consequently, the available scaling factors for ground state calculations were utilized, as detailed in Table S3 [39, 40, 41]. For the functional/basis set combinations lacking published scaling factors, the FREQ program by Truhlar and co‐workers was employed to create them for this study, using the full scale factor optimization model [42, 43, 44]. Both literature and scaling factors derived from FREQ will be denoted as Sc_L_ further in the text. An additional scaling factor was devised called Specific Scaling Factor (Sc_S_, see Table S3), which aligns the two highest frequency peaks in the theoretical and experimental spectra: the S_1_ state symmetric C=O stretch of each DFT functional (v_75_) and the experimental FMN peak at 1626 cm^−1^ of the 3^rd^ EAS, respectively.

Due to the extended benchmarking, all calculations employed either the modest polarized double‐ζ basis set cc‐pVDZ or its expansion including diffuse functions, aug‐cc‐pVDZ [45, 46]. The latter always produced excited states within the experimental resonance window, set at the wide range of 750–800 ± 100 nm [19, 20, 35], which was not always the case for the modest basis set. Specifically, for B3LYP, a functional that has been associated with numerous computational studies of flavins [20, 21, 35], a basis set dependence/convergence study is included up to the polarized quadruple‐zeta cc‐pVQZ [45, 46, 47, 48], together with the equivalent augmented sets (up to triple‐ζ), ranging from 326 up to 1405 basis functions. The Polarizable Continuum Model (PCM) was used as the solvation method in all calculations with water as the solvent. Equilibrium solvation was used for both ground state [49, 50, 51] and excited state optimizations [52].

Vertical excitations, simulations of ground‐state UV–vis spectra, and excited state optimizations were carried out with the TDDFT formalism [52, 53, 54, 55] and were solved for a total of 40 states for each combination of functional/basis set. One photon absorption calculations (OPA) for the S_0_ → S_1_ excitation were performed with the time‐independent approach, employing the Franck‐Condon analysis and the Adiabatic Hessian representation [56, 57]. 2^18^ steps were used for integration with a time interval of 2^18^ × 10^−17^ s. For the plotting of the absorption curves, Gaussian broadening was employed with a half‐width at half maximum value of 400 cm^−1^ (HWHM). The key equations pertaining to the OPA calculations are included in Section 2.1 of the Supporting Information.

For the resonance Raman frequency calculations on excited singlet states, the Franck‐Condon analysis was used with the Adiabatic Hessian representation [56, 58, 59, 60]. The key equations of the time‐dependent Resonance Raman implementation [24] are included in Section 2.2 of the Supporting Information. The path‐integral approach was utilized with an explicit definition of the transition dipole moments. 2^12^ steps were used for integration with the correlation function computed at each step and a time interval of 2^12^ × 10^−17^ s. All resonance spectra were computed at the 0–0 transition between the reference (S_1_/T_1_) and the resonant (S_n_/T_n_) state. Test calculations employing the experimental incident light frequency produced no discernible differences in their relative intensities. A HWHM homogeneous broadening of 20 cm^−1^ was applied to the spectra. For the evaluation of the calculated resonance Raman intensities, the Huang‐Rhys factors (HRF) were computed and evaluated [61]. For the Raman spectra calculated at optimized structures on the S_1_ manifold (off‐resonance, offR) a HWHM value of 10 cm^−1^ was used to match the experimental line curves. The excited singlet state calculated spectra were compared to the experimental FSRS third Evolution Associated spectrum (EAS), with a lifetime of *τ* = 2.9 ns assigned to the equilibrated 1FMN* state [19].

For triplet state calculations, the lowest state T_1_ was obtained by setting the spin multiplicity to 3 and using the unrestricted formalism of the DFT functionals. This had the advantage of allowing resonance spectra to also be computed with the Franck‐Condon‐Herzberg‐Teller method (FCHT) [24, 60], since dipole derivatives were available for the T_1_ → T_n_ excitations. For FCHT, to evaluate computed peak intensities, the two‐state dipole–dipole interactions on the XY plane were inspected at each vibration (lumiflavin is almost planar) – since the computed HRF factors were identical to the FC‐computed spectra. The rest of the parameters were as described above in the excited singlet state resonance Raman calculations. As per the singlet case, the triplet off‐resonance spectra were calculated at optimized structures on the T_1_ manifold (off‐resonance, offR) and a HWHM value of 10 cm^−1^ was used to match the experimental line curves. The key equations pertaining to the FCHT calculations time‐dependant implementation [24] are included in Section 2.2 of the Supporting Information.

Additionally, since the closest T_1_−T_n_ transition lies further from the resonance window than in the case of the singlet state [19, 20], pre‐resonance spectra were calculated (preR) making use of the CPHF equations [62, 63]. The difference between the experimental Raman pump and the closest Triplet‐Triplet (T–T) transition was taken into account to compute the pre‐resonance Raman spectra. The incident light wavelength was calculated according to the equation: *λ*_IL(C)_ = (*λ*_RP(E)_ − *λ*_T−T(E)_) + λ_T−T(c)_, where λ_IL(C)_ is the incident light wavelength used for the calculation, λ_T−T(E)_ and λ_T−T(c)_ are the wavelengths of the experimental and calculated T–T transitions respectively, and λ_RP(E)_ is the wavelength of the Raman pump. For pre‐resonance spectra, a HWHM homogeneous broadening of 15 cm^−1^ was applied to the spectra to match the experimental spectral plots. All computed excited triplet state spectra were compared to the experimental FSRS fifth Evolution Associated spectrum (EAS), with a lifetime of *τ* = 458 μs assigned to the equilibrated 3FMN* state [19].

For selected functionals (B3LYP, HCTH, OLYP, TPSSh) a potential energy distribution analysis (PED) was performed using the program Veda [64]. PED provided an alternate set of peak assignments to normal modes as well as the coefficients for each mode. Average Maximum Potential Energy <EPm> values ranged between 46‐51 affording reasonable vibrational assignments.

Basic statistical analysis was performed, due to the vastness of the data set, including the simple absolute deviation *σ* = |*V*_T_−*V*_E_|, the percent error *δ* and their averages μ_σ_ and μ_δ_. The percent error is given by the equation $\delta =\left|\frac{V_{T}-V_{E}}{V_{E}}\right|*100$. Since the experiments are used as a reference, *V*_T_ and *V*_E_ in the equations are defined as the theoretical and experimental values, respectively. All spectral plots were made using the Spectragryph software [65], and unless stated otherwise, have been normalized.

## 3 Results and Discussion

### 3.1 Ground State and Vertical Excitations

**SCHEME 2:**
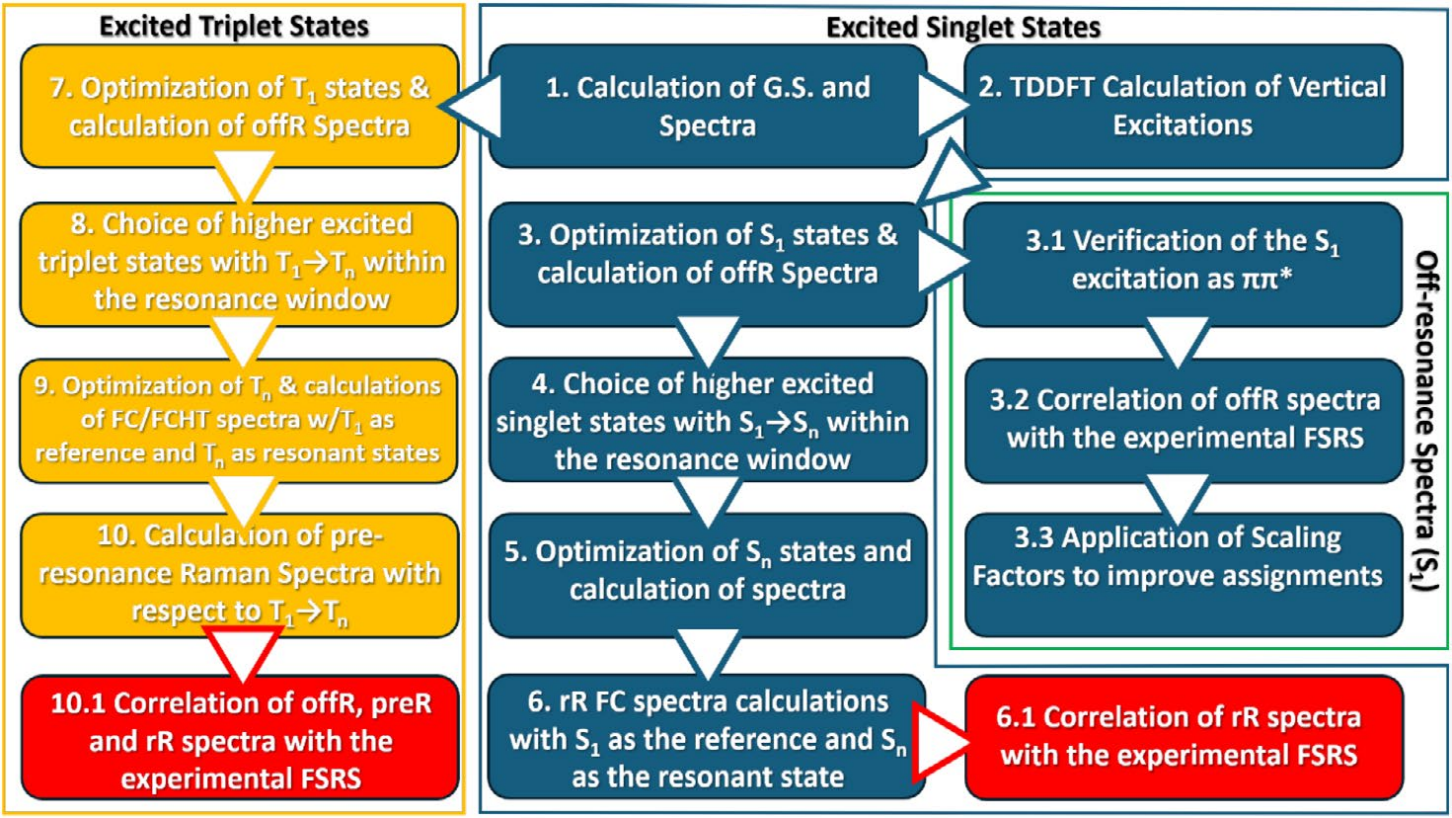
Schematic representation of the computational regime followed in this study.

The study proceeded with the optimization, excitation analysis for 40 states, and Raman frequency calculation of the ground state of all the selected DFT functionals (Scheme 2, steps 1 and 2). The two major excitations of lumiflavin (S_0_ → S_1_ and S_0_ → S_2_) were compared to the experimental *λ*_max_ values for FMN at 445 and 372 nm, respectively [20]. While there is no direct physical connection between the theoretical and experimental properties, the comparison is standard practice for analogous studies [11, 66]. A more robust comparison involves the determination of the 0–0 transitions, which will be discussed in the subsequent Section 3.2. The average percent error μ_δ_ of the two values for each DFT functional is included in Figure S1A, sorted from larger to smallest error. B3LYP predicts excitations close to the experimental values—which justifies its popularity among DFT functionals in the study of flavin systems [12, 33, 34] with 7.1% and 4.0% percent error for the cc‐pVDZ and augmented equivalent, respectively. Long‐range corrected functionals such as LC‐OPBE and CAM‐B3LYP fare much worse, with over 20% error, similarly to the BHandHLYP functional (50% HF exchange), irrespective of the basis set. Among the most accurate predictions are given by the Minnesota meta‐GGA functionals M06L and M11L with errors lower than ~3%. The separate absolute deviation values σ_S0→S1_ and σ_S0→S2_ and their average (*μ*_σ_) are included in Figure S1B for all DFT functionals. Likewise, all separate, averaged deviations and percent errors for the two excitations are collected in Table S4. Of particular interest are the predicted oscillator strengths ƒ(S_0→1_) and ƒ(S_0→2_), which are included in the last two columns. Approximately half of the DFT functionals correctly predict the ƒ(S_0→1_) band to be more intense, as per the experimental stationary UV–Vis spectrum of FMN [20]. Additionally, functionals that their intensities approach the experimental band intensity ratio of ~1.15 include M06 and M06‐HF, B1B95, PBE0, mPW1PW91, and PW6B95D3. B3LYP incorrectly predicts ƒ(S_0→2_) with higher oscillator strength, which was also observed in the full FMN structure [20]. The unnormalized simulated absorption spectra of all functionals are plotted in Figure S2.

### 3.2 Off‐Resonance Calculations

Subsequently, the first excited singlet state S_1_ was optimized (Scheme 2, Step 3). Most of the functionals predicted the first root (r_1_)—while some of the GGA functionals, particularly with the smaller basis set—predicted r_2_ as the lowest singlet excited state. The optimized S_1_ state was identified as the lowest energy ππ* transition, before and after optimization (Scheme 2, Step 3.1). For LSDA/aug‐cc‐pVDZ in particular, the r_1_ state was identified as nπ*, thus further analysis was carried out only the cc‐pVDZ basis set (see Table S10 for hole/electron information on the S_1_ ππ* states).

**FIGURE 1:**
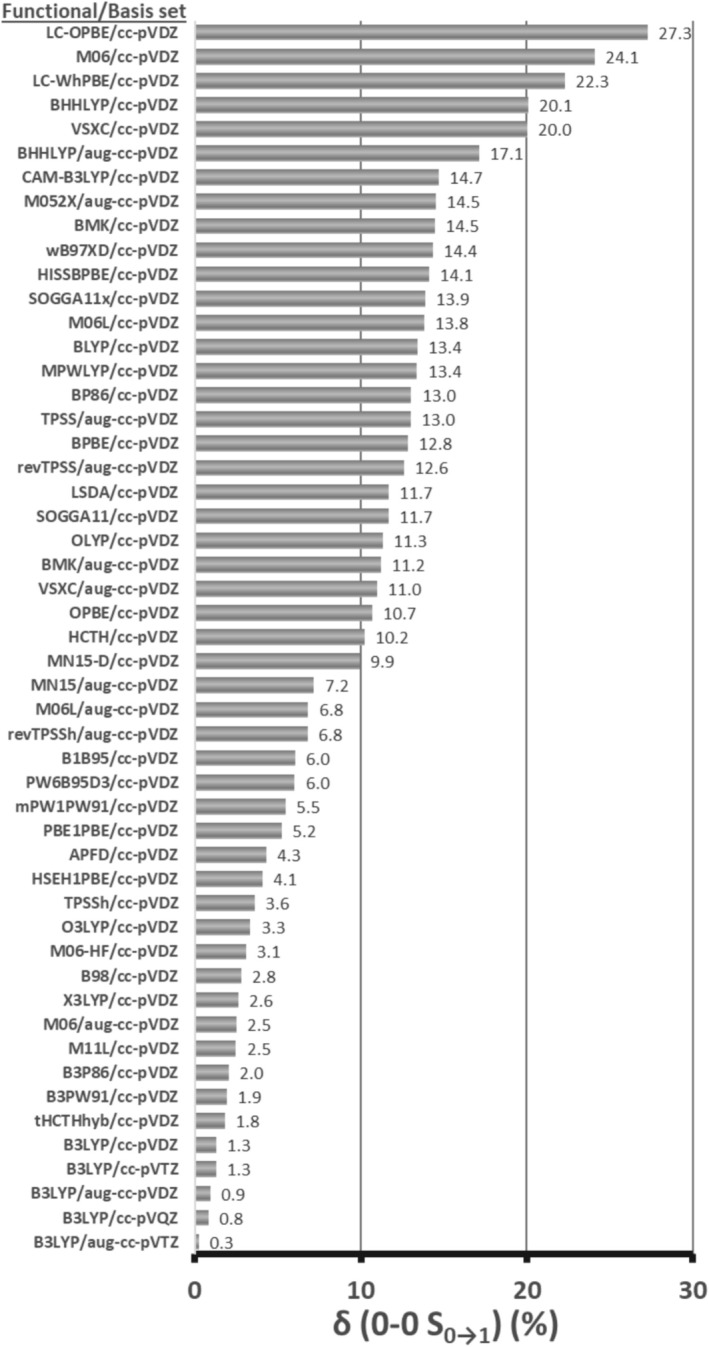
Percent error (δ, %) of the S_0→1_ 0–0 transition of lumiflavin with each of the tested DFT functionals with respect to the experimental absorption/fluorescence crossing point of FMN [20].

**FIGURE 2:**
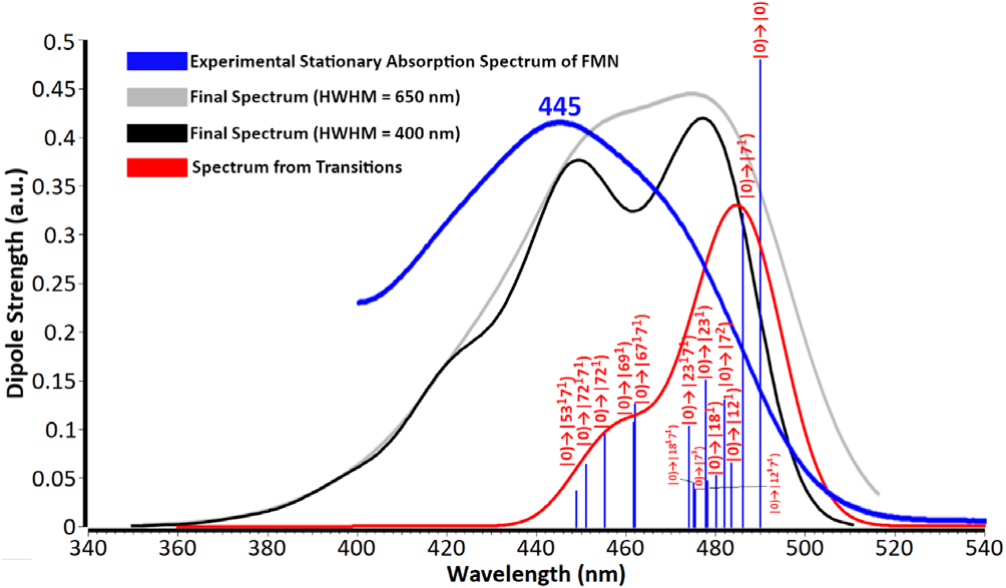
Composite vibronic absorption spectrum of lumiflavin derived from the averaged shifts and dipole strengths of all vibronic transitions reported in Tables S7 and S8. The experimental stationary S_0_→S_1_ band of FMN is included with a blue line [20], two final spectra with different HWHM values are plotted in gray and black, and the spectrum from transitions in red. All vibronic transitions are labeled and shown with blue sticks.

A more robust criterion than the comparison of vertical excitations with the experimental UV–vis bands is the 0–0 transition between the ground and the first excited state, which gives information about the shape of the excited PES and the relative energies of states. This was determined for all functionals and derived from the zero point energy corrected energies of the corresponding states, and compared to the experimental value of the absorption‐fluorescence crossing point of FMN, determined at 498 nm [20]. The percent error is shown in Figure 1 and displays a distinct ranking of functionals compared to the vertical excitation comparison of the previous section. M06L, which provided the most accurate vertical excitations, predicts the 0–0 transitions with 6.8% and 13.8% error with the larger and smaller functional, respectively. B3LYP shows the best overall performance regardless of basis set, with lower than 1.3% error, which justifies being the functional of choice for flavin‐based systems when accurate energy levels are a requirement. All terms used in the calculation of the 0–0 transitions are included in Table S6. However, accurate energies do not always yield accurate spectra [67], and corrections, such as the scaling schemes introduced in the next Section 3.3, or the incident light correction in Section 3.6, can compensate for those inaccuracies. Complementary to the 0–0 transitions, one photon absorption (OPA) calculations were performed to inspect the vibronic structure of lumiflavin regarding the S_0_ → S_1_ excitation. The spectra of all DFT functionals were scaled according to their adiabatic energies (E^adia^, listed in Table S6) and are shown in Figure S4. The obtained spectra exhibit three distinct features in their S_0_ → S_1_ band shape (using a HWHM value of 400 cm^−1^), in contrast to the experimental broad band of FMN in H_2_O, plotted in Figure 2 with a blue line [20]. However, both riboflavin and lumiflavin are known to exhibit similar vibronic band motifs in ethanol [68], as well as FMN embedded in photoproteins [5]. Common features present in all the OPA spectra include the following vibronic transitions: |0) → |7^1^/8^1^), |0) → |7^2^/8^2^), |0) → |23^1^), |0) → |23^1^7^1^) and |0) → |72^1^) (see Table S5 for their vectors). Since most DFT functionals exhibited similar vibronic composition, this allowed for a representative average spectrum to be constructed, shown in Figure 2, based on the average values of the shifts (with respect to 0–0) and dipole strengths of their vibronic transitions, included in Tables S7 and S8. The |0) → |0) transition is the most intense in all spectra, followed by the in‐plane |0) → |7^1^), and out‐of‐plane |0) → |8^1^) transitions. Of note is also |0) → |23^1^), present in all vibronic spectra, due to in‐plane compression‐stretch mode of the isoalloxazine rings II, III. Comparable findings were reported previously by Saalfrank and co‐workers in gas‐phase and DMSO vibronic calculations of riboflavin [69]. In comparison with the experiment, the functionals B1B95, MPW1PW91, MN15, revTPSSh, PWD6B95D3, and PBE0 produce spectra centered closer to the experimental absorption band wavelength (445 nm).

**FIGURE 3:**
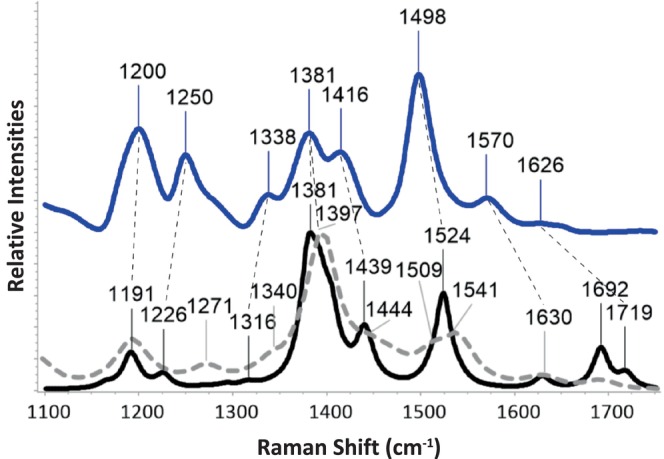
The Experimental FSRS 3^rd^ EAS of FMN assigned to the equilibrated 1FMN* state is shown on the top as a solid blue line [19], the calculated off‐Resonance S_1_ spectrum at the B3LYP/cc‐pVDZ level is shown on the bottom as a solid black line, and the calculated resonance Raman spectrum of the S_1_ → r_7_ transition at the same LOT is overlayed with a dashed gray line. All assigned peaks from Table 1 have been labeled and the experimental/theoretical associated peaks are connected with thin black dashed lines. The intensities of all included spectra have been normalized.

Then, the off‐Resonance Raman spectra of all verified S_1_ states were computed and compared to the experimental spectrum (Scheme 2, Step 3.2). The spectra were correlated with the 3^rd^ Evolution Associated Spectrum (EAS) of the FSRS of FMN, assigned to the equilibrated 1FMN* state (see Table S12) [19]. Eight prominent peaks are featured in the fingerprint region of the 3^rd^ EAS (Figure 3, blue line), and these were associated with the calculated peaks in the 1000–1900 cm^−1^ region. Due to the abundance of theoretical vibrations in the fingerprint region, multiple vibrations can be correlated to each experimental peak: for the FSRS EAS peak at 1200 cm^−1^ two computed peaks, mostly between v_48_‐v_52_ were correlated, for 1250 cm^−1^ there was a single peak match, most frequently either v_51_ or v_53_, the EAS 1338 cm^−1^ band was associated with vibrations v_54_‐v_56_, for 1381 cm^−1^ up to four computed peaks between v_57_‐v_61_ were correlated, for 1416 cm^−1^ either v_64_ or v_65_, for 1498 cm^−1^ mostly v_71_ was correlated and secondarily v_70_ or v_72_, for 1570 cm^−1^ either v_73_ or v_74_ and finally for 1626 cm^−1^, the last vibration in the fingerprint region was correlated (v_75_). Typical vibrations with displacement vectors are shown in Table S5 originating from the B3LYP/aug‐cc‐pVDZ S_1_ frequency calculation.

Following visual assignment of computed vibrations to normal modes as well as PED analysis, the computed spectra at each functional were correlated with the eight experimental peaks described above. An example of such correlation is shown in Figure 3 and the left portion of Table 1 for the unscaled off‐Resonance S_1_ spectrum (S_1_offR) computed at the B3LYP/cc‐pVDZ level (for the rest of the functionals, see Table S12). Computed peaks at 1381 cm^−1^ (together with peaks 1379, 1391 and 1405 cm^−1^) and 1524 cm^−1^ are straightforwardly assigned to the experimental 1381 and 1498 cm^−1^ peaks, respectively. Averaging the deviations *σ* for all experimental/theoretical matches gives the overall agreement, which in this example is μ_σ_(offR) = 37.3 cm^−1^. This somewhat large value is mostly due to the deviations of the higher frequency peak assignments at 1570 and 1626 cm^−1^. The above process was repeated for the off‐resonance spectra of each DFT functional and yielded μ_σ_(offR) values, which are included in the 3^rd^ column of Table 2. As mentioned above, the very weak peak of the 3^rd^ EAS at 1626 cm^−1^, was assigned to v_75_ in all cases, since this is the highest frequency peak in the fingerprint region of both the experimental and theoretical spectra. As seen in Table 1, v_75_ is attributed to the symmetric C=O stretching mode of lumiflavin (*s*CO_s_), which is expected to be more Raman active than the asymmetric equivalent v_74_ (*s*CO_as_). While other computed vibrations vary, the assignment of the v_74_ and v_75_ vibrations to the asymmetric and symmetric CO stretch modes, respectively, is valid for all the included DFT functionals.

**TABLE 1:** Correlation between the experimental FSRS 3^rd^ EAS of FMN (Exp.) and the off‐resonance (S_1_offR) and resonance Raman (r_7_rR) calculations of lumiflavin at the B3LYP/cc‐pVDZ level of theory. Peak intensities, vibration numbers, and normal mode assignments are included. The latter are simplified using the Latin numerals I–III to assort the modes to each of the isoalloxazine rings (see Scheme 1). Normal modes are reported from largest to smallest displacement vectors, and PED assignments are given in percentages. Absolute deviations from the experimental values (*σ*_offR_, *σ*_rR_) are included along with their averages (μ_σ_(offR), μ_σ_(r_7_)) in the last row. New assigned peaks due to the inclusion of resonance in the calculations are underlined.

Exp.	v_#_	S_1_offR	Assignment	*σ*_offR_	v_#_	r_7_rR	Assignment	*σ*_rR_
1200 s	v_49_	1191 w	*r*CH_I_ (10%), *r*NH_III_, *s*NC_II_ (10%), *s*CC_I_ (21%), *x*CH_3II_ (11%)	9	v_49_	1191 m	*r*CH_I_, *r*NH_III_, *s*NC_II_, *s*CC_III_	9
1250 m	v_51_	1226 w	*r*CH_I_, *r*NH_III_, *s*NC_III_ (36%), *s*CC_III_	24	v_52_	1271 w	*r*CH_I_ (42%), *s*CC_I_, *x*CH_3I_, *s*NC_II_	21
1338 w	v_54_	1316 vw	*r*CH_I_, *s*NC_II_ (19%), *s*NC_III_	22	v_55_	1340 m	*r*NH_III_ (11%), *s*CC_I,II_ (13%), *s*NC_II_ (26%), *r*CH_I_, *x*CH_3II_	2
1381 s	v_57_	1379 vs	*r*CH_I_, *r*NH_III_ (16%), *x*CH_3II_ (10%), *s*NC_II_, *s*CC_III_, sCC_I_ (13%)	8	v_60_	1397 vs	*x*CH_3I,II_ (28%), *s*NC_II,III_ (18%), *r*NH_III_, *s*CC_I_	16
	v_58_	1381 vs	*x*CH_3I_ (34%), *r*NH_III_, *s*CC_I_ (16%)					
	v_59_	1391 vs	*x*CH_3I_ (47%), *s*NC_II_, *s*NC_III_, *s*CC_II_					
	v_61_	1405 s	*r*NH_III_ (31%), *r*CH_I_, *s*CC_II,I_, *s*NC_III_					
1416 m	v_64_	1439 m	*x*CH_3I_, *s*NC_II,III_ (32%), *s*CC_II_	25	v_65_	1444 m	*s*NC_II_, *x*CH_3I_, *r*NH_III_, *s*NC_III_	28
	v_65_	1444 m	*s*NC_II,III_, *x*CH_3I,II_ (43%), *r*NH_III_					
1498 vs	v_71_	1524 s	*r*CH_I_, *s*NC_II_, *s*CC_I_ (44%), *s*CC_III_	26	v_70_	1509 m	*x*CH_3I,II_ (13%), *s*CC_I_ (14%), *s*NC_II_ (16%), *s*NC_I_	11
1570 w	v_73_	1630 w	*r*CH_I_, *s*CC_I_ (53%), *s*NC_III_	91	v_72_	1541 m	*s*NC_II_, *s*NC_III_ (10%), *r*CH_I_, *s*CC_I_	29
	v_74_	1692 m	*s*CO_as_ (70%), *r*NH_III_					
1626 vw	v_75_	1719 w	*s*CO_s_ (72%), *r*NH_III_	93	v_73_	1630 vw	*r*CH_I_, *s*CC_I_, *s*NC_III_	4
			**μ**_**σ**_ **(offR)**	**37.3**			**μ**_**σ**_ **(r**_**7**_ **)**	**15.0**

*Note:* Intensities: vs = very strong, s = strong, m = medium, w = weak, vw = very weak. Assignments: *w* = wagging, *r* = rocking, *t* = twisting, *s* = stretching, *x* = scissoring, s = symmetric, as = asymmetric.

**TABLE 2:** Statistical analysis of the correlation between the experimental spectrum of the 3^rd^ EAS assigned to 1FMN* and the lumiflavin calculated off‐resonance (offR) and resonance Raman (r_n_) spectra at each level of theory (LOT). The terms μ_σ_(offR) and μ_σ_(r_n_) are the average deviations of the offR and rR spectra, respectively and Δμ_σ_ their difference. The term μ_σ_(Sc_L_) gives the average deviation of offR spectra after applying the literature (or computed with FREQ for this study) scaling factor, and μ_σ_(Sc_S_) is the average deviation after applying the specific scaling factor included in Table S3. The term μ_σ_(Sc_S_)* involves the re‐assignment after scaling of DFT functionals with a Sc_S_ lower than 0.93. The terms μ_δ_(offR) and μ_δ_(r_n_) are the average percent errors of the offR and rR spectra, respectively and Δμ_δ_ their difference. All deviation values (σ) are given in cm^‐1^ and percent errors (δ) in %.

LOT	State	μ_σ_(offR)	μ_σ_(r_n_)	Δμ_σ_	μ_σ_(Sc_L_)	μ_σ_(Sc_S_)	μ_σ_(Sc_S_)*	μ_δ_(offR)	μ_δ_(r_n_)	Δμ_δ_
APFD/cc‐pVDZ	r_5_	47.1	51.3	−4.2	39.8	53.5	—	3.11	3.41	−0.30
B1B95/cc‐pVDZ	r_7_	68.5	36.9	31.7	31.6	46.6	44.3	4.75	2.53	2.22
B3LYP/cc‐pVDZ	r_7_	37.3	15.0	22.3	40.0	54.1	—	2.50	1.07	1.42
B3LYP/aug‐cc‐pVDZ	r_6_	23.6	17.4	6.2	34.8	27.1	—	1.61	1.23	0.38
B3LYP/cc‐pVTZ	r_7_	29.3	23.8	5.5	29.8	31.1	—	1.99	1.63	0.35
B3LYP/aug‐cc‐pVTZ	r_7_	23.5	22.3	1.3	28.7	20.9	—	1.61	1.54	0.07
B3LYP/cc‐pVQZ	r_6_	27.2	25.3	1.8	27.7	26.1	—	1.85	1.76	0.09
B3P86/cc‐pVDZ	r_7_	44.1	41.6	2.5	38.7	53.1	—	2.89	2.79	0.11
B3PW91/cc‐pVDZ	r_7_	43.1	40.1	3.0	34.1	52.1	—	2.83	2.69	0.14
B98/cc‐pVDZ	r_7_	44.3	32.1	12.1	31.7	50.9	—	3.02	2.15	0.87
BHHLYP/cc‐pVDZ	r_4_	96.1	50.3	45.9	59.0	79.7	28.5	6.48	3.45	3.03
BHHLYP/aug‐cc‐pVDZ	r_5_	83.0	48.2	34.9	48.6	51.0	16.9	5.65	3.48	2.17
BLYP/cc‐pVDZ	r_7_	27.2	39.8	−12.6	31.5	63.9	−	1.96	2.92	−0.97
BMK/cc‐pVDZ	—	61.4	—	—	49.5	91.6	24.4	4.06	—	—
BMK/aug‐cc‐pVDZ	r_5_	47.7	32.4	15.3	36.7	57.6	40.0	3.18	2.34	0.84
BP86/cc‐pVDZ	r_7_	27.8	22.2	5.6	27.5	48.3	—	2.02	1.63	0.40
BPBE/cc‐pVDZ	r_7_	22.3	17.8	4.6	30.9	44.8	—	1.59	1.30	0.30
CAM‐B3LYP/cc‐pVDZ	r_5_	60.2	30.9	29.3	47.9	68.3	42.3	3.95	2.21	1.73
HCTH/407/cc‐pVDZ	r_7_	30.6	16.7	13.9	29.8	49.0	—	2.05	1.19	0.86
HISSbPBE/cc‐pVDZ	r_7_	105.6	60.5	45.0	34.9	56.2	42.6	7.39	4.16	3.24
HSEH1PBE/cc‐pVDZ	r_7_	51.2	22.4	28.8	34.9	56.2	41.1	3.39	1.59	1.80
LC‐OPBE/cc‐pVDZ	r_4_	144.9	233.3	−88.5	64.9	80.0	38.5	9.81	16.46	−6.64
LC‐wHPBE/cc‐pVDZ	r_5_	154.0	188.8	−34.8	62.9	28.6	20.9	11.05	13.45	−2.40
LSDA/cc‐pVDZ	r_8_	36.4	27.1	9.3	31.5	63.9	—	2.48	2.02	0.46
LSDA/aug‐cc‐pVDZ	—	39.0	—	—	30.3	36.3	—	2.77	—	—
M05‐2X/aug‐cc‐pVDZ	r_4_	56.1	40.5	15.6	33.6	36.1	—	3.78	2.71	1.08
M06/cc‐pVDZ	r_7_	51.3	43.2	8.1	50.2	81.1	24.8	3.39	2.85	0.54
M06/aug‐cc‐pVDZ	r_5_	35.8	57.2	−21.4	37.4	49.7	—	2.36	4.04	−1.67
M06‐HF/cc‐pVDZ	r_6_	42.0	18.6	23.4	58.7	84.4	26.8	2.72	1.31	1.40
M06L/cc‐pVDZ	r_5_	60.4	45.7	14.6	19.9	52.5	17.1	4.21	3.14	1.06
M06L/aug‐cc‐pVDZ	r_7_	32.1	25.6	6.5	37.8	40.0	—	2.17	1.75	0.43
M11L/cc‐pVDZ	r_6_	56.3	22.6	33.7	50.4	85.7	22.9	3.71	1.60	2.12
MN15/cc‐pVDZ	r_5_	52.2	53.0	−0.7	49.3	73.7	16.0	3.41	3.58	−0.18
MN15/aug‐cc‐pVDZ	r_4_	45.2	25.1	20.1	37.4	44.7	—	3.04	1.85	1.19
mPW1PW91/cc‐pVDZ	r_7_	56.8	16.2	40.6	33.4	53.5	42.1	3.79	1.14	2.65
mPWLYP/cc‐pVDZ	r_7_	20.6	31.5	−10.9	22.9	27.2	—	1.56	2.32	−0.76
O3LYP/cc‐pVDZ	r_7_	40.6	14.8	25.8	45.2	63.1	—	2.75	1.05	1.70
OLYP/cc‐pVDZ	r_7_	36.1	14.4	21.7	36.2	58.8	—	2.56	1.04	1.52
OPBE/cc‐pVDZ	r_7_	39.9	22.3	17.6	45.0	67.1	—	2.72	1.63	1.10
PBE1PBE/cc‐pVDZ	r_5_	49.6	43.6	6.1	38.7	61.0	43.8	3.26	2.89	0.36
PW6B95D3/cc‐pVDZ	r_7_	54.4	65.9	−11.5	40.3	55.3	41.4	3.60	4.40	−0.80
revTPSS/aug‐cc‐pVDZ	r_7_	7.0	15.3	−8.3	25.2	6.4	—	0.50	1.13	−0.63
revTPSSh/aug‐cc‐pVDZ	r_7_	162.3	51.3	110.9	42.6	12.7	20.6	11.49	11.39	0.10
SOGGA11/cc‐pVDZ	r_9_	25.8	24.2	1.6	43.8	65.1	—	1.79	1.63	0.16
SOGGA11x/cc‐pVDZ	r_5_	73.6	54.6	18.9	43.8	65.1	40.1	4.95	3.82	1.13
tHCTHhyb/cc‐pVDZ	r_7_	34.9	22.1	12.8	42.0	56.8	—	2.35	1.48	0.87
TPSS/cc‐pVDZ	r_5_	27.3	21.3	5.9	18.5	34.2	—	1.90	1.46	0.44
TPSS/aug‐cc‐pVDZ	r_7_	7.0	15.5	−8.6	29.6	6.6	—	0.51	1.13	−0.62
TPSSh/cc‐pVDZ	r_7_	35.7	14.8	20.9	31.1	44.0	—	2.44	1.04	1.40
VSXC/cc‐pVDZ	r_5_	40.3	33.1	7.2	22.5	43.9	—	2.79	2.28	0.52
VSXC/aug‐cc‐pVDZ	r_7_	16.0	21.3	−5.4	27.1	13.1	—	1.14	1.51	−0.37
wB97XD/cc‐pVDZ	r_6_	71.4	43.3	28.0	35.4	58.5	36.8	4.84	2.96	1.89
X3LYP/cc‐pVDZ	r_7_	44.6	43.8	0.9	29.3	40.8	—	3.05	2.90	0.16

* Re‐assignment of peaks for functionals with Sc_s_ lower than 0.93.

### 3.3 Scaling the Frequencies

**FIGURE 4:**
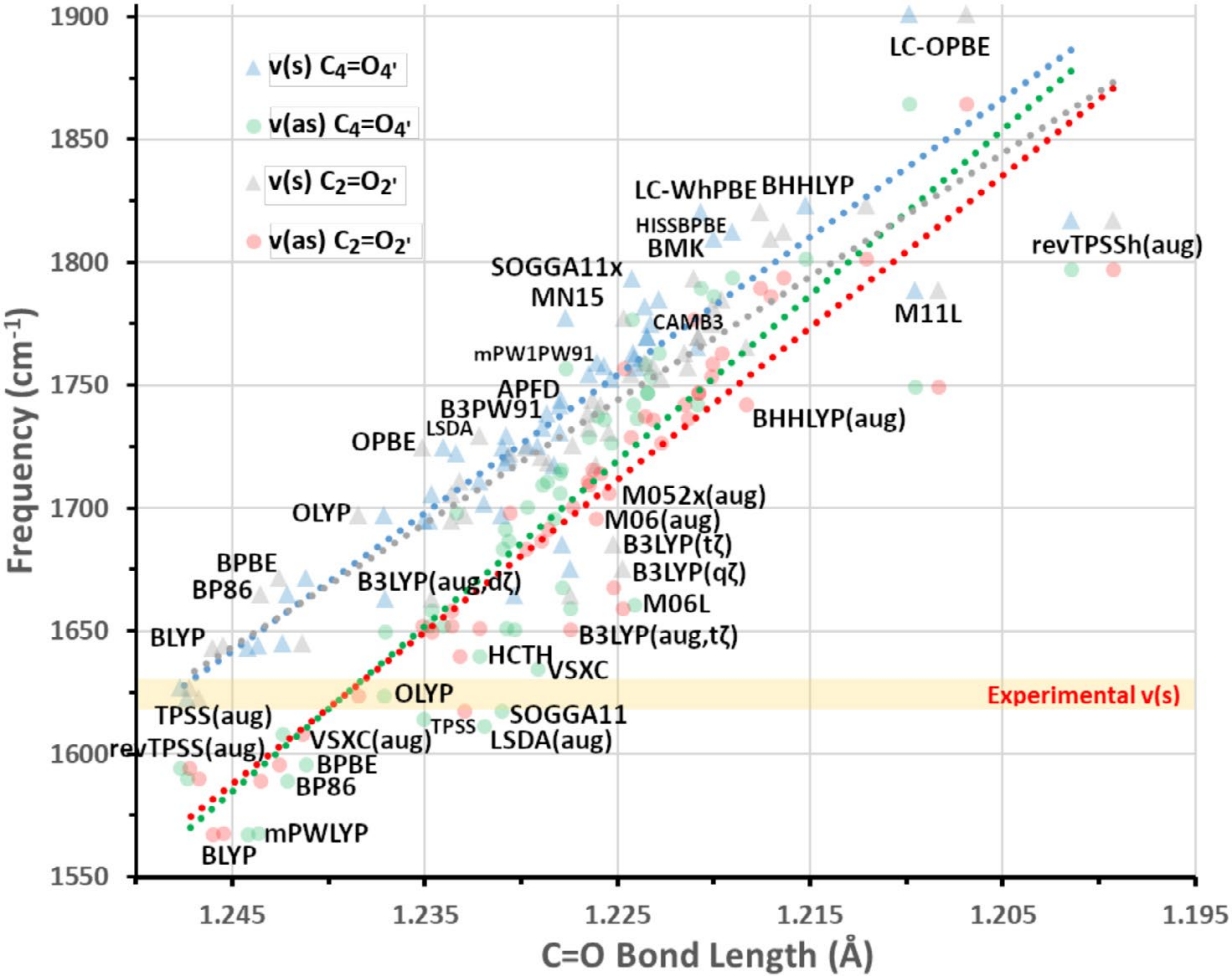
Relationship between the length of the C_2_=O_2′_ and C_4_=O_4′_ bonds (x‐axis) calculated with the tested DFT functionals, and their symmetric (s) and asymmetric (as) stretching frequencies (y‐axis). The v_s_/C_2_=O_2__'_ and C_4_=O_4__'_ bond length values are given in gray and blue triangles respectively, while the v_as_ C_2_=O_2′_ and C_4_=O_4′_ in red and green squares, respectively. Trendlines with corresponding color have been drawn for each of the four sets. The experimental C_2_=O_2′_/C_4_=O_4′_ symmetric stretching v(s) frequency (1626 cm^−1^) has been highlighted in yellow.

The above findings highlight a complication of all vibrational calculations and the predicted frequency values. In Figure 4, the relationship between the C_2_=O_2′_, and C_4_=O_4′_, bond lengths and their respective symmetric v(s) and asymmetric v(as) stretching frequencies is plotted. It can be seen that most DFT functionals predict v(s) to lie between 1800 and 1700 cm^−1^, and a few long‐range corrected functionals even near 1900 cm^−1^ (blue and gray triangles in Figure 4). The experimental value highlighted in yellow is centered at 1626 cm^−1^ highlighting this discrepancy. On the other hand, some functionals cluster in the lower range (> 1700 cm^−1^) including: TPSS and its revision, BLYP, mPWLYP, VSXC, B3LYP, BPBE, M06, OLYP, and SOGGA11 mentioned in order from closest to furthest from the experimental value. A structural solution that has been applied widely is microsolvation [20, 33], where explicit solvation is combined with the implicit solvent model. Water molecules are placed at key H‐bonding positions, including two proximal to the flavin C=O bonds. The resulting bond lengthening, concomitant with the shift of the stretching vibrations to the red, would skew the distribution to the bottom left part of the graph of Figure 4, closer to the experimental value.

For the context of this benchmark, the discrepancy was addressed by scaling the frequencies uniformly, which was undertaken either by applying literature scaling factors (derived from G.S. datasets) and alternatively the ones computed in this study by the FREQ program (Sc_L_), or by devising a specific scaling factor to align v_75_ to 1626 cm^−1^ (Sc_S_) (Step 3.3, Scheme 2). All scaling factors that were employed are included in Table S3. The Sc_L_ and Sc_S_ factors were applied uniformly to the unscaled peaks in each assignment table, yielding new μ_σ_(Sc_L_) and μ_σ_(Sc_S_) values, which are included in columns 6 and 7 of Table 2, respectively. The application of the scaling factors produced a mixed picture. It improved the worse correlations including the long range corrected LC‐wHPBE from 154.0 to 62.9 and 28.6 cm^−1^ for μ_σ_(Sc_L_) and μ_σ_(Sc_S_), respectively. Conversely, for functionals possessing reasonable agreement with their unscaled peaks, such as SOGGA11 (μ_σ_(offR) = 25.8 cm^−1^), the correlation worsened to 43.8 and 65.1 cm^−1^ for μ_σ_(Sc_L_) and μ_σ_(Sc_S_), respectively. For the levels of theory (LOTs) that their Sc_S_ factor was found below 0.93 (see Table S3, 19 DFT functionals—21 LOTs) a re‐assignment of peaks was performed, with new corresponding values μ_σ_(Sc_S_)* (8^th^ column, Table 2). The re‐assignment improved the correlations of most functionals compared to their μ_σ_(Sc_L_) and μ_σ_(Sc_S_) values (i.e., the LC‐wHPBE μ_σ_(Sc_S_)* value was reduced to 20.9 cm^−1^). Thus, scaling factors can be a useful tool, specifically in the absence of anharmonic corrections or microsolvation, but care should be taken in their usage. It should be mentioned here that only a few of the DFT functionals gave better agreement, either in the μ_σ_(S_1_), μ_σ_(Sc_L_) or μ_σ_(Sc_S_) values, than the full, micro‐solvated FMN structure, optimized at the B3LYP/def2‐TZVP level of theory (μ_σ_(Sc_L_) = 19 cm^−1^) [20]. Lastly, a relation between energetics and spectra is shown in Figure S3. The errors in the spectral correlation (y‐axis) are plotted together with the errors in 0–0 energies (x‐axis) for the ~20 DFT functionals that the Sc_L_ factor was applied. Two sets of data are included, before and after scaling with Sc_L_. When comparing the two sets, it is evidenced that errors in correlation drop below 4% after scaling (and re‐assignment), regardless of the accuracy of the 0–0 transitions. Notably, no linear relationship is established between energetic and spectral accuracy on both data sets.

### 3.4 Resonance Raman Calculations

The next step the in the computational regime is the choice of excitations within the experimental resonant window, defined as the wavelength of the Raman pump 800 ± 100 nm (Step 4, Scheme 2). The window was in practice extended to more than ±300 nm to accommodate as many states as possible in the study. Transition dipole moments, excitation energies, and oscillator strengths between S_1_ and the higher singlet states *r*_n_ were determined using the program Multiwfn 3.8 [70], and are collected in Table S9, along with the difference between pump and excitation energy (Δ*r*). From all the states chosen, the most suitable candidates are shown in bold, owing to the high oscillator strengths and proximity to the resonance wavelength. All states included in Table S9 were optimized (Step 5, Scheme 2), either leading to the expected state or regressing to other state potential energy surfaces (PES), including S_1_. This was verified from their respective energies and additionally from the hole–electron properties and distribution surfaces included in Tables S10 and S11, respectively. For example, optimization of the r_5_ state with BMK/cc‐pVDZ yielded the S_1_ state and no further analysis was completed for this level of theory (LOT). Thus, the basis set was increased to aug‐cc‐pVDZ, and steps 1–4 were repeated. Similarly, states r_5_–r_8_ yielded the same r_7_ state after optimization with M06/cc‐pVDZ, and accordingly the study proceeded only with r_7_ for that LOT. Apart from the above‐described augmentation of the basis set, other techniques to mitigate state crossing during optimizations were judged as nontrivial and prohibitive for the number of states involved and were not pursued, which is a limitation of the study presented here. However, as indicated in Table S9, for the majority of functionals, the states with the higher oscillator strength transitions were obtained.

Subsequently, the excited Raman spectra of all unique states for each LOT were computed. This led to the final step (Step 6, Scheme 2), the resonance Raman calculation using the r_n_ as the resonant and the corresponding S_1_ state as the reference state. Spectra were obtained at the 0–0 transition between reference and resonant states, and a broadening of 20 cm^−1^ was applied to the peaks. Correlation and re‐assignment of spectra were performed with the off‐resonance assignment as a basis (Step 6.1, Scheme 2). The correlations were established with the unscaled frequencies, depending on the normalized resonance‐enhanced intensities. Thus, the assignment of the 1626 cm^−1^ peak to v_75_ was not enforced as with the offR spectra and was distributed almost equally between vibrations v_73_, v_74_ and v_75_ among the DFT functionals, depending on which was predicted more intense. In the B3LYP case, the new assignment is shown in the right portion of Table 1. In that case, six out of eight assignments are new due to resonance enhancement, and this brings a superior correlation μ_σ_(r_7_) = 15 cm^−1^ than the off‐resonance value of 37.3 cm^−1^. An advantage of the rR calculation is that single resonance‐enhanced peaks are assigned to the experimental ones, in contrast to the averaging of peaks that was required for off‐resonance correlation. However, as was mentioned in the Introduction, the calculated resonance Raman spectrum of B3LYP produces worse relative intensities than the off‐resonance with respect to the experimental FSRS—which is obtained under resonant conditions itself. This is evident for the calculated peak at 1541 cm^−1^ of the S_1_↔r_7_ spectrum included in Figure 1 as a gray dashed line.

The same process of re‐assignment and correlation of the rR spectra was repeated for the other functionals, producing μ_σ_(r_n_) and μ_δ_(r_n_) averages for their correlation. The new assignments are included in Table S13, and offR and rR spectra of all functionals are overlayed in Figure S5.

**FIGURE 5:**
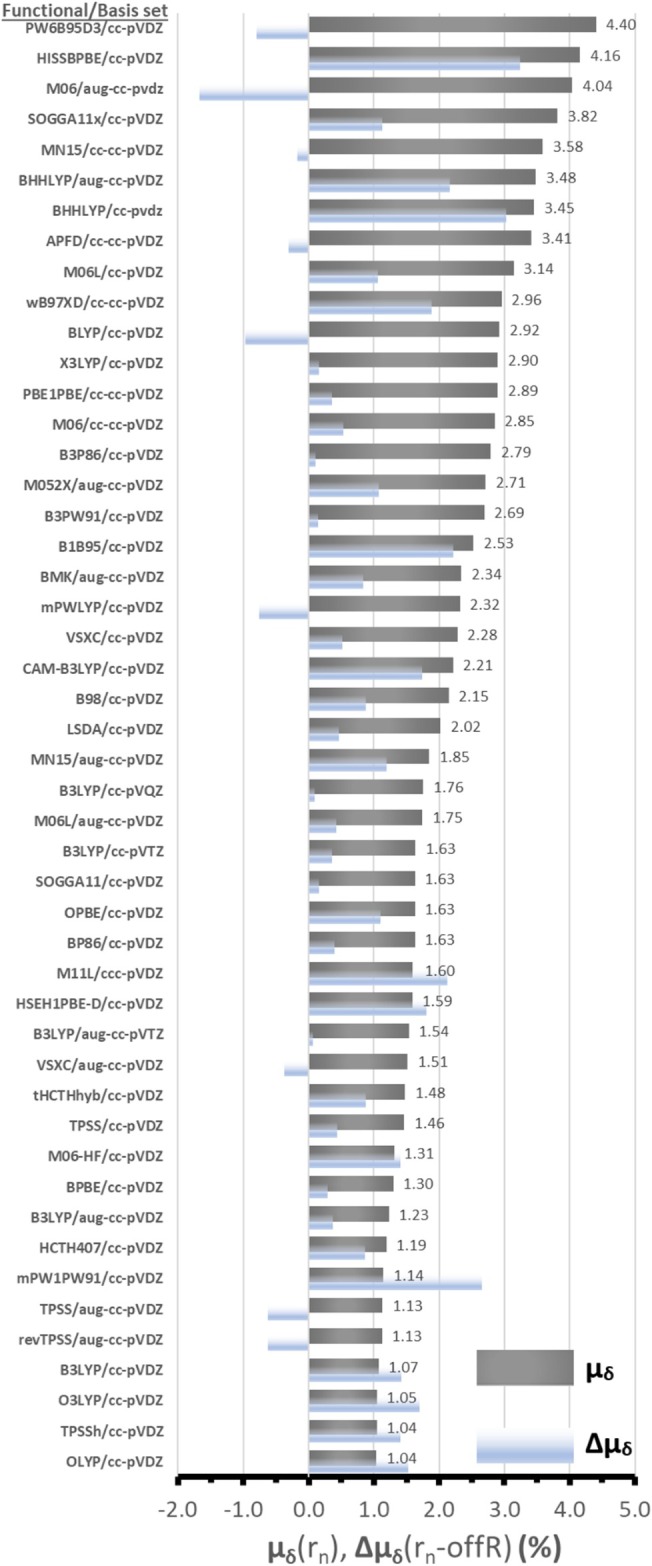
Average Percent error (μ_δ_, %) of the correlation of calculated major peaks of the singlet spectra of lumiflavin for each of the tested DFT functionals with respect to the experimental FSRS 3^rd^ EAS of FMN. The μ_δ_ values are based on the resonance Raman correlation rR(FC), while Δμ_δ_ gives the subtraction value between μ_δ_(r_n_) and μ_δ_(offR) for each functional. The functionals/states have been sorted by decreasing μ_δ_(r_n_) values which are labeled.

To specifically address the issue mentioned above for the B3LYP functional and gauge the measure of improvement or worsening of the correlation with the inclusion of resonance in the calculations, the term Δμ_δ_(r_n_‐offR) was devised. This is a simple subtraction of the μ_δ_(offR) and μ_δ_(r_n_) values returning a positive number if the correlation improves and negative if it worsens with the inclusion of resonance in the spectra of each combination of DFT functional/basis set. The μ_δ_(r_n_) and Δμ_δ_ terms are included together in Figure 5 which orders the functionals from higher to lower μ_δ_(r_n_) values (LC‐OPBE, LC‐wHPBE and revTPSSh have been omitted for scaling purposes). All mentioned terms are included in the final three columns of Table 2 (μ_δ_(offR), μ_δ_(r_n_), Δμ_δ_). For most functionals, the correlation was improved with the re‐computed intensities, with the exception of 10 functional/basis set combinations: APFD/cc‐pVDZ (−0.30%), M06/aug‐cc‐pVDZ (−1.67%), BLYP/cc‐pVDZ (−0.97%), mPWLYP/cc‐pVDZ (−0.76%), PW6B95D3/cc‐pVDZ (−0.80%), revTPSS/aug‐cc‐pVDZ (−0.63%), TPSS/aug‐cc‐pVDZ (−0.62%) and VSXC/aug‐cc‐pVDZ (−0.37%). The largest differences were found for the range‐corrected functionals LC‐OPBE/cc‐pVDZ (−6.64%) and LC‐wHPBE/cc‐pVDZ (−2.40%).

### 3.5 Evaluation of DFT Functionals

Due to the large amount of data considered, evaluation of all functionals according to specific criteria was required in order to extract useful information from the benchmarking. This was attempted according to the five criteria formulated below. Each functional can obtain up to five positive marks (■) according to its compliance with the criteria thresholds. For cases that lie outside, but close to the thresholds, a half‐mark was given (◧). A positive evaluation is given for the DFT functionals that:

- The percent error δ of the S_0→1_ 0–0 transition lies below 4.1% scoring a full mark, or below 11.7% scoring a half mark. The equivalent threshold values in absolute deviations (*σ*) are 0.3 and 0.1 eV, respectively.
- The mean percent error, μ_δ_(r_n_) of the rR computed peaks associated with the eight most prominent experimental FSRS peaks of the 3^rd^ EAS is equal to or lower than 1.5%. Half‐mark is given for DFT functionals between 1.5% and 2% of error. In that case, the equivalent mean deviation μ_σ_(r_n_) range would be 21–27 cm^−1^. For the functionals with more states found within the resonant window, the μ_δ_(r_n_) values pertain only to the state r_n_ with the higher oscillator strength for the S_1_ → r_n_ transition.
- The difference between the off‐resonance and resonance Raman mean percent errors (Δμ_δ_) is positive. As mentioned above, a positive sign signifies the improvement of the correlation after the inclusion of resonant effects to the computed intensities and vice versa. If Δμ_δ_ is negative, no mark is awarded, while if it is higher than 1%, the full mark is awarded; a half mark is given for functionals with positive values between 0.3% and 1%.
- For this criterion, the focus is on the most prominent experimental peak at 1498 cm^−1^. The HRFs of the eight vibrations correlated to the corresponding experimental peaks were normalized in the range of 0–1. Then, the normalized value of the vibration assigned to 1498 cm^−1^ (v_71_ for the majority of functionals) is evaluated according to the following criteria: For values between 0.8 and 1 a full mark is given, signifying that the DFT functional correctly (or almost correctly) predicts the strongest peak in the spectrum. A half mark is given for normalized values between 0.5–0.8, and below 0.5, no mark is awarded.
- Finally, a subjective criterion is introduced, that of visual inspection of the intensities of the computed resonance Raman spectra and their compatibility with the experimental FSRS intensities. The computed spectra fall within five categories as follows: The DFT functional is evaluated positively when the correlation of the theoretical–experimental spectra is facile: (i) with a full mark for very similar line shapes to the experimental curve or (ii) half mark for less similar but still providing for a facile correlation. A negative evaluation with no mark is given for computed spectra that either: (iii) bear no visible doublet peaks that can be easily correlated to the 1200–1250 cm^−1^ and 1381–1416 cm^−1^ experimental pairs, or (iv) require a scaling factor for the frequencies, usually when v_73_ is predicted with strong intensity and scaling would align it with the experimental peak at 1498 cm^−1^ or finally, (v) the C=O symmetric or asymmetric stretch is predicted as the strongest peak in the spectrum.

The evaluation of the functionals is given in Table 3 along with the total positive marks (out of 5) for each functional for the (a–e) criteria.

**TABLE 3:** Evaluation of the excited singlet state spectra of all DFT Functionals according to five criteria: (a) the percent error of the 0–0 transition between GS and S_1_, (b) the resonance Raman mean percent error, (c) the difference in the mean percent errors of the resonance and off‐resonance Raman spectra, (d) the normalized Huang‐Rhys Factor of the vibrations assigned to the 1498 cm^−1^ peak of the 3^rd^ EAS of FMN and (e) visual evaluation, where the computed resonance Raman spectra are classified after inspection according to the categories (i–v) described in the main text.

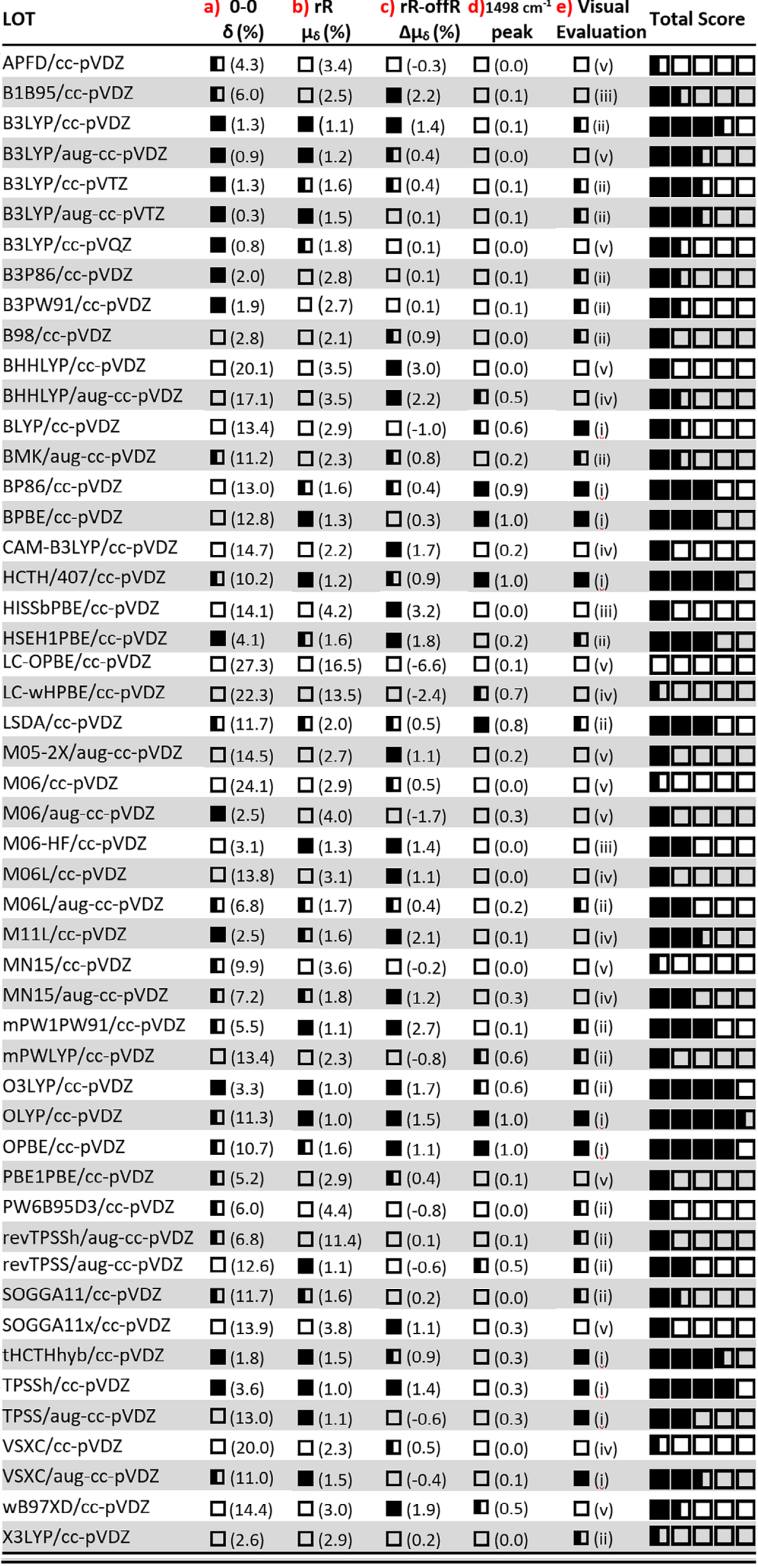

**FIGURE 6:**
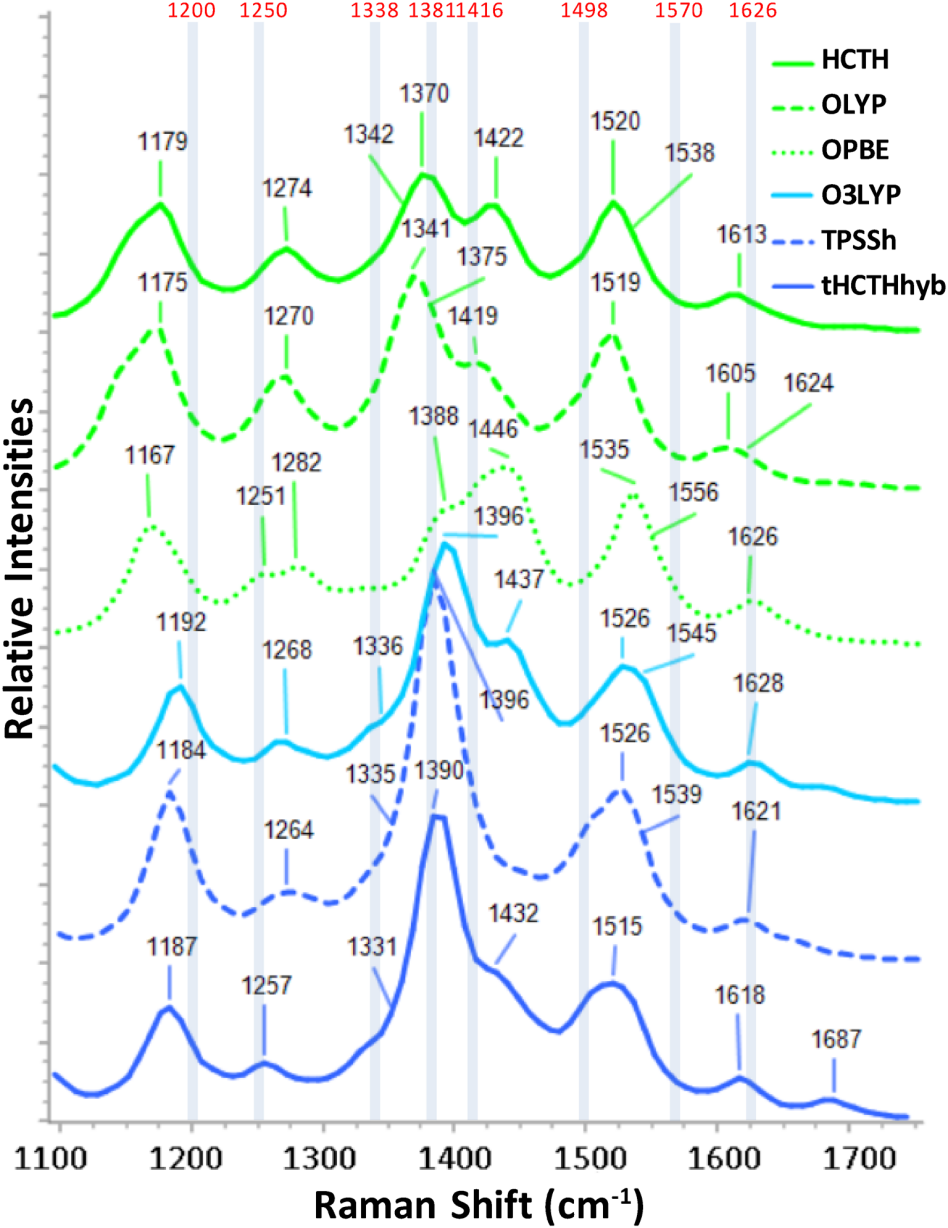
Calculated resonance Raman spectra of the best performing DFT functionals of the benchmark with the cc‐pVDZ basis set. The prominent peaks of the experimental 3^rd^ EAS of 1FMN* have been labeled with red numbers and indicated with blue‐gray bars [19]. The intensities of all included spectra have been normalized.

With 4 out of 5 points (4½ for OLYP), five DFT functionals stand out in their performance against the five criteria: the GGA functionals HCTH/407, OLYP and OPBE, the hybrid O3LYP functional with 11.6% HF‐exchange and the meta‐Hybrid functional TPSSh with 16% HF‐exchange (all employing the cc‐pVDZ basis set). Surprisingly, the revised TPSSh functional fared much worse in the evaluation. Close to the above with 3½ points, follow the hybrid B3LYP/cc‐pVDZ (with 20% HF exchange), and the meta‐Hybrid tHCTHhyb with 10% HF exchange. LSDA, the GGA functionals BP86 and BPBE, the hybrid mPW1PW91 (25% HF), and the range‐separated hybrid HSEH1PBE (25% HF) follow with 3 points. The resonance Raman spectra of the five best functionals (and tHCTHhyb) are included in Figure 6. As can be seen in Table 3, for four from the above mentioned functionals (HCTH, OLYP, OPBE and BPBE) v_71_ is the strongest peak in the fingerprint region, as per the experiment, and for O3LYP, LSDA and BP86 the second strongest (0.8–0.9). By inspection of spectral intensities in Figure 6 (and Figure S5), peaks in the region ~1350–1450 cm^−1^ appear more intense, however this is due to the concentration of quite a few medium‐to‐strong peaks in that region. Overall, the highest scoring DFT functionals mentioned above (≥ 3½) enabled a facile correlation with the experimental EAS, with clear spectral features close to the experimental peaks marked with gray bars in Figures 6 and S5. Regarding the functionals that scored three points, LSDA, BP86 and BPBE conform also to the above, while HSEH1PBE and mPW1PW91 fared worse in the spectral intensity criteria (d, e).

For the basis set dependence, it was reported before [71] that the addition of diffuse functions increases the accuracy of harmonic frequencies, while enlarging the basis set over the triple‐ζ limit brings only marginal gain. With regards to the B3LYP functional basis set study included here, there is clear improvement in the off‐resonance μ_σ_(offR)/μ_δ_(offR) values with the increase of the basis set which is more pronounced with the inclusion of diffuse functions (see Table 2 and μ_δ_ values in Figure 5). Concerning the rR μ_σ_(r_n_) values, marked improvement is achieved when compared to the corresponding μ_σ_(offR) values. Conversely, when comparing the μ_σ_(r_n_) values themselves with increasing basis set size, the opposite trend is evidenced, with slight increase in disagreement with every increase in basis set from 15 cm^−1^ with cc‐pVDZ to 25.3 cm^−1^ with cc‐pVQZ. Scaling with the literature scaling factors Sc_L_ does not improve any of the offR correlations. The specific ones (Sc_S_) improve only the larger sets in the series, aug‐cc‐pVTZ and cc‐pVQZ from 23.5 and 27.2 cm^−1^ to 20.9 and 26.1 cm^−1^, respectively. B3LYP/cc‐pVDZ performed better overall than the larger basis sets, while B3LYP/aug‐cc‐pVTZ was the most accurate DFT functional in the 0–0 transition prediction (Table 3).

### 3.6 Calculation and Evaluation of Triplet Spectra

For the study of the triplet spectra of lumiflavin, the eight best scoring functionals mentioned above were considered (mPW1PW91 and HSEH1PBE were excluded), with the addition of BLYP, BHHLYP(aug), mPWLYP, and revTPSS(aug). The latter four were included due to the half mark awarded for criterion (d) which was assumed would provide for facile assignment also in the triplet state. B3LYP was not included in the triplet study, since it has been analyzed extensively before [20, 35]. The T_6_ triplet state at the LSDA/cc‐pVDZ LOT, located within the resonance window, proved difficult to optimize, and the functional was excluded from further study.

As per the excited singlet state study, the triplet study commenced with the calculation of the T_1_ states and their spectra (offR) for the selected 12 DFT functionals (Step 7, Scheme 2). Correlation was performed for the off‐Resonance spectra with the 5^th^ EAS of FMN assigned to the 3FMN* state (for the assignments, see Table S17) [19]. The 5^th^ EAS possesses less spectral features than the 3^rd^ EAS with only five prominent bands in the fingerprint region at 1190, 1269, 1391, 1514 and 1626 cm^−1^. All experimental peaks were associated to single computed vibrations except 1391 cm^−1^, which was assigned to four vibrations (two for OPBE), between v_55_‐v_60_ for most functionals. Then, the choice of the higher triplet states (T_n_) ensued (Step 8, Scheme 2), assisted by the analysis included in Tables S14–S16. Except the states T_6_ of tHCTHhyb, T_3_ of BHHLYP and T_5_‐T_6_ of O3LYP, the rest of the higher triplet states listed in Table S14 were optimized successfully, indicating that triplet state crossing was less pronounced than in the excited singlets case. The triplet vibrational spectra also exhibited blue‐shifted C=O stretching modes, while the experimental peak in the 5^th^ EAS remained at 1626 cm^−1^ (Figure S7). However, in contrast to the singlet spectra, most functionals predict the v(s) mode below 1700 cm^−1^, and scaling of the frequencies was not required.

**FIGURE 7:**
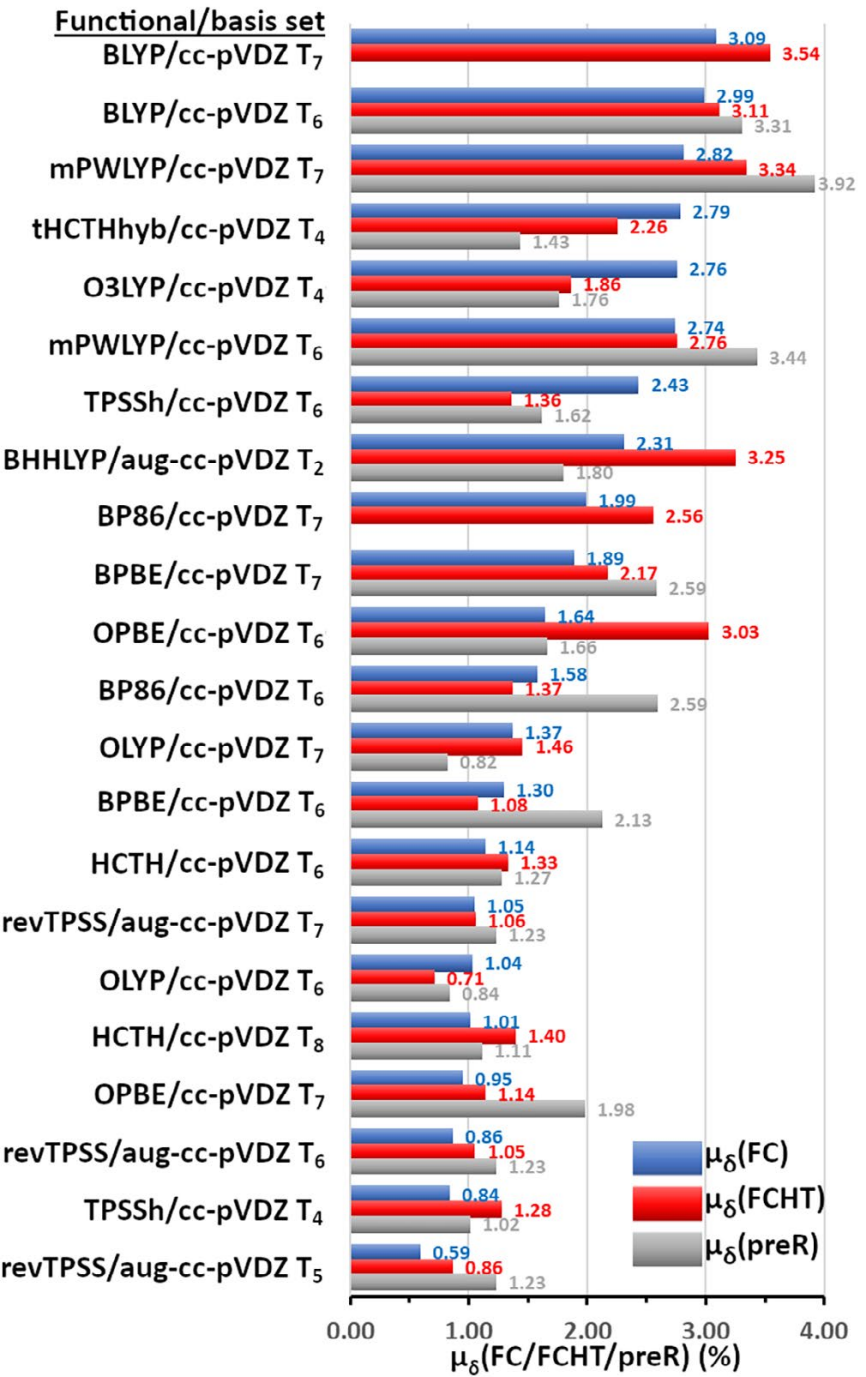
Average Percent error (μ_δ_, %) of the correlation of calculated major peaks of the triplet spectra of lumiflavin for each of the selected twelve DFT functionals at different T_n_ states, with respect to the experimental FSRS 5^th^ EAS of FMN. Depending on the color, the μ_δ_ values are based on the resonance Raman correlations (FC, blue or FCHT, red) or the pre‐resonance correlation (preR, gray). The functionals/states have been sorted by decreasing μ_δ_(FC) values. The μ_δ_ values have been labeled with the respective color.

The next step in the regime was the optimization and subsequent calculation of the resonance Raman spectra (Step 9, Scheme 2). After optimization, hole–electron surfaces are shown in Table S16, together with the acceptor SUMO orbitals. Since the relevant T–T transitions lie further than S–S with respect to the Raman pump, the pre‐Resonance spectra (preR) were also calculated for the same states (Step 10, Scheme 2). The incident light (λ_IL(C)_) used in the calculations for each T_n_ state is included in the last column of Table S14 and its determination was described in the Computational Details Section 2. As with offR, preR and both rR calculations, including FC or FCHT terms, were correlated with the experimental 5^th^ EAS (Step 10.1, Scheme 2). The new correlations are included in Table S18, and statistics are collected in Table S19. Similarly to the singlet study, triplet Δμ_δ_ values demonstrate that for most functionals (with the exception of BLYP), the experimental EAS matches better the preR, rR(FC) and rR(FCHT) spectra than the offR. The μ_δ_ values are included in Figure 7 for the preR, rR(FC) and rR(FCHT) correlations sorted by the μ_δ_(FC) values. In most cases, FC correlations are comparable to preR, and FCHT fare slightly worse. Among the 12 functionals, OLYP, HCTH, OPBE, revTPSS, and TPSSh stand out for their better correlation with the 5^th^ EAS. All computed spectra are included in Figure S6.

**FIGURE 8:**
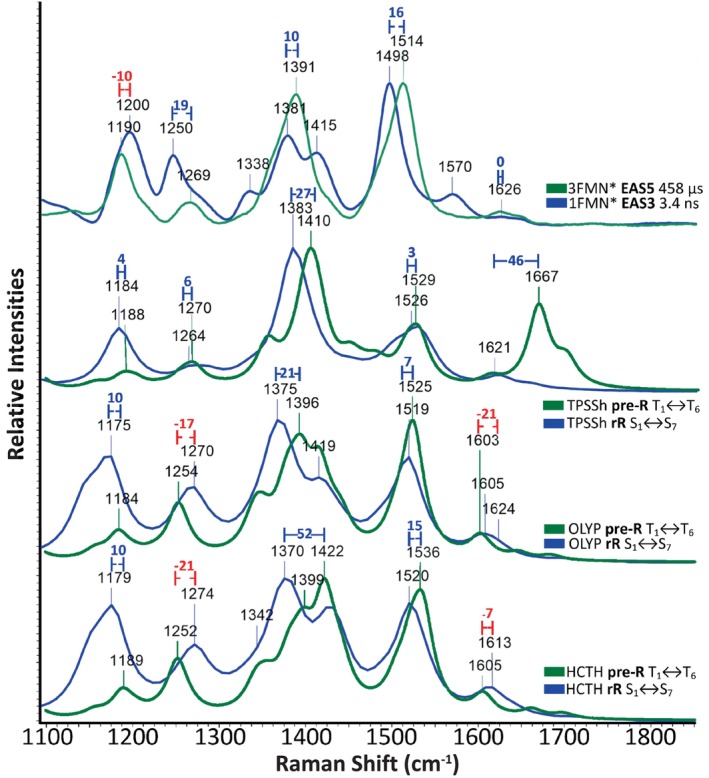
The experimental Singlet‐Triplet spectral shifts are highlighted on the experimental FSRS (top) 3^rd^ EAS assigned to 1FMN* (blue line) and 5^th^ EAS assigned to 3FMN* (green line), followed by the theoretical spectra of TPSSh (middle top) overlaying the excited singlet resonance Raman spectrum based on the S_1_ → S_7_ transition (blue line) with the pre‐resonance Triplet spectrum based on the T_1_ → T_6_ transition (green line), OLYP (middle bottom) overlaying the excited singlet resonance Raman spectrum based on the S_1_ → S_7_ transition (blue line) with the pre‐resonance Triplet spectrum based on the T_1_ → T_6_ transition (green line), and HCTH (bottom), overlaying the excited singlet resonance Raman spectrum based on the S_1_ → S_7_ transition (blue line) with the pre‐resonance Triplet spectrum based on the T_1_ → T_6_ transition (green line). Shifts are colored blue or red accordingly and the wavenumber difference between the shifted peaks is displayed. The intensities of all included spectra have been normalized.

As with the excited singlet study, evaluation of the DFT functionals for the triplet spectra was performed according to five criteria. These are described in Section 3.1 in the Supporting Information and include similar criteria to (b–e) described above for the Singlets. An additional criterion (d) was introduced, evaluating the Singlet‐Triplet shifts evidenced when comparing the experimental 3^rd^ and 5^th^ EAS (Figure 8, top). Among them stand out the characteristic blueshift at 1498 → 1514 cm^−1^ and the symmetrical C=O stretching vibration which remains unaltered at 1626 cm^−1^ both reported first by us previously [20]. The S‐T shifts are listed in Table S20 for the offR, preR, and rR spectra and are marked in Figure 8 on the spectra of functionals HCTH, OLYP, and TPSSh. Manifestly, this is a challenging criterion, since none of the functional/state/method combinations predicted correctly more than three shifts out of the five. The evaluation results are presented in Table S21, including, in the final column, the combined score of the functional. This incorporates the singlet rating together with the best score for the triplet state, which for the higher scoring functionals originates from the preR calculation (see Table S21 for the score and Figures 8 and S6 for the spectra). OLYP was the best overall performing functional with a score of 9/10, followed by HCTH and TPSSh with 8/10.

**FIGURE 9:**
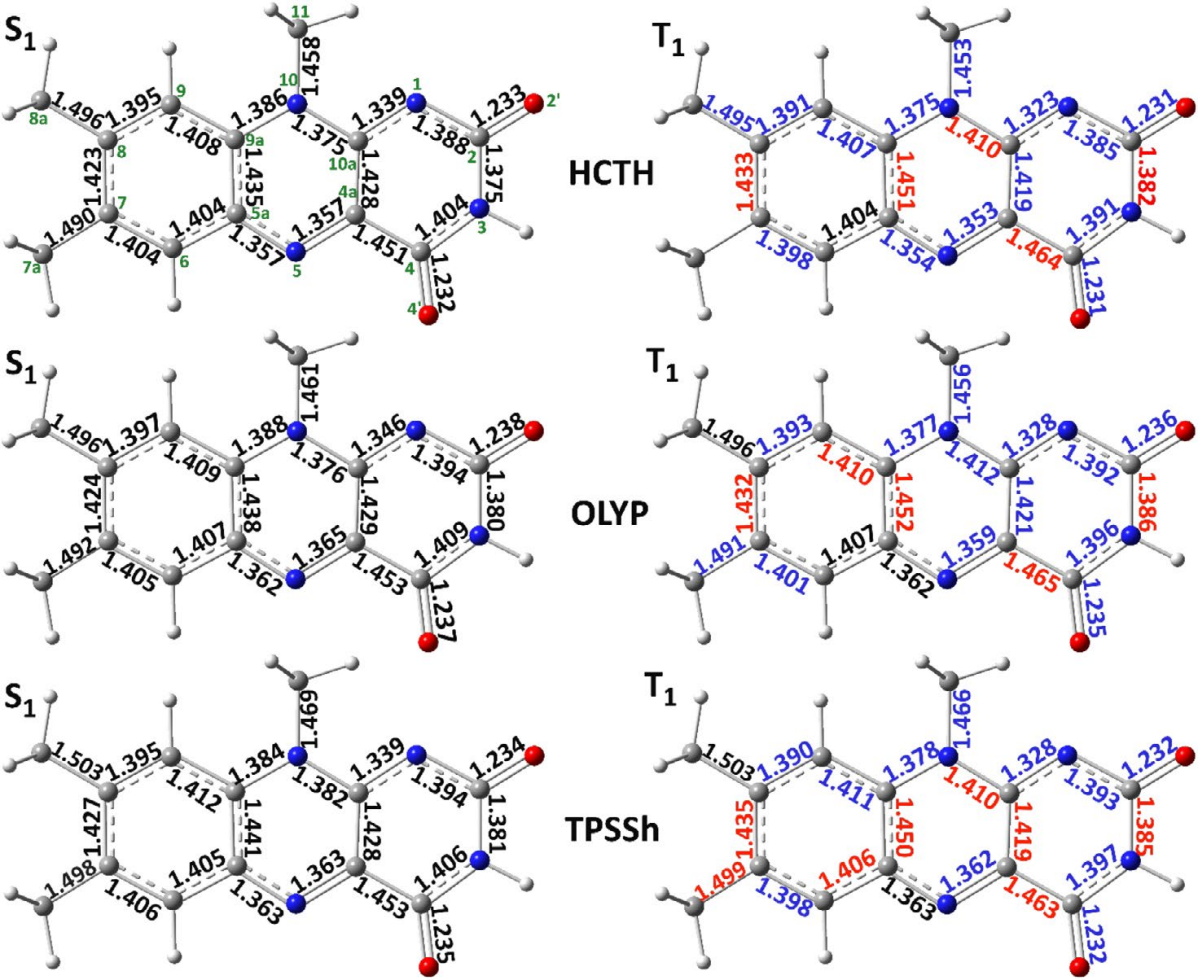
Bond lengths of optimized excited singlet (left) and triplet (right) state structures, for three DFT functionals: HCTH (top), OLYP (middle) and TPSSh (bottom). On the T_1_ structures the bond lengths are colored according to bond shortening (blue) or lengthening (red) with respect to the S_1_ structure. Atom numbering is included with green letters on the HCTH S_1_ structure (top left).

Finally, in an effort to rationalize the evidenced S‐T spectral changes that were part of the triplet evaluating criterion (d), the bond length differences between the S_1_ and T_1_ optimized structures were determined for OLYP, HCTH, and TPSSh (Figure 9). In a simplified approach, vibrations with normal modes involving bonds that are shortened from the S_1_ to the T_1_ equilibrated states will experience a blue shift in their frequencies; conversely, lengthening bonds will contribute to frequency shifts to the red. Following this notion, the normal modes involving bond stretching for the aforementioned three functionals were color‐coded accordingly in the respective assignment Tables S12 and S13. Then, a prediction of the S‐T shifts was made according to the constituent blue‐ and red‐shifting modes for each assigned vibration. To augment the assignment according to the displacement vectors employed throughout this study and provide an alternative prediction of S‐T shifts, PED assignments were determined for the S_1_ and T_1_ states of the three DFT functionals (Table S22). The PED and displacement shift predictions are included and take into account only normal modes involving bond stretching, as described above. These predictions can be judged both against the experimental shifts and the actual computed shifts for each functional. The two S‐T spectral features that proved the most difficult to reproduce are the 1200 → 1190 cm^−1^ red shift and the invariance of the 1626 cm^−1^ peak. The other three experimental blue shifts are predicted correctly by the PED and displacement assignments for both the HCTH and OLYP functionals, while displacement analysis predicts correctly the red shift of the 1200 cm^−1^ peak but not the blue shift at 1250–1269 cm^−1^ in TPSSh. From the above, it can be surmised that the proffered assignments are, by and large, accurate.

After a three‐tiered benchmark of the DFT functionals evaluating separately the produced singlet and triplet spectra and then the singlet‐triplet spectral changes between them, the three best scoring DFT functionals HCTH/407, OLYP and TPSSh can be recommended for resonance Raman studies on the flavin family of chromophores. Yet, the collective knowledge on excited state calculations warns against the usage of the first two, pure GGA functionals, since they tend to underestimate charge transfer (CT) over Local Excitation (LE) energies [72]. While this did not pose a significant problem with standalone lumiflavin, in larger systems, numerous CT states—real or artificial—would be present, for example, between residue side chains and the π‐system of the chromophore—and the inclusion of 10%–15% exact‐exchange would be warranted. Functionals that performed particularly well in the excited singlet evaluation, such as the pure GGA BP86 and BPBE functionals, the hybrid O3LYP (11.6% HF) and the meta‐hybrid tHCTHhyb (15% HF), should also be mentioned, and these could be employed in systems that do not cross over to the triplet state, such as BLUF. Lastly, the benchmarks affirmed B3LYP as one of the most accurate functionals in the prediction of flavin vertical excitations and 0–0 transitions, and its utilization is recommended for studies that the accuracy of resonance Raman spectra are not of primary concern.

It remains to be ascertained if the findings presented here are transferable to other well‐documented chromophore and photoprotein/chromophore systems. Additionally, a similar type of vibrational studies would benefit from comprehensive research on excited state scaling factors, particularly for some long‐range corrected functionals.

## 4 Conclusions

An extensive benchmarking resonance Raman study of lumiflavin was presented here involving 42 different DFT functionals in combination with the polarized double‐zeta basis set cc‐pVDZ, and the inclusion of diffuse functions in selected cases. Initially, the singlet vertical excitations were compared to the experimental values, followed by the calculation of off‐resonance singlet and triplet spectra. Scaling was applied to the vibrations of the S_1_ spectra aligning the computed symmetrical C=O stretch to the 1626 cm^−1^ experimental peak, which improved the correlation in most cases. A careful choice of resonant states lying within the experimental resonance window ensued, and resonance Raman spectra were computed, and in the case of the triplet, were complemented by pre‐resonance spectra. All DFT functionals underwent evaluation according to five criteria in their singlet state, and the best 12 functionals were considered for the further study of the triplet state of lumiflavin. Subsequent evaluation narrowed down the selection to the functionals HCTH/407, OLYP, and TPSSh as the most capable overall of reproducing the excited singlet and triplet spectra under resonance and pre‐resonance conditions, respectively, and the singlet‐triplet peak shifts of FMN.

The following strategies can be beneficial in the study of excited state resonance Raman calculations:

- For the rR approach utilized in this study, steps 1–6 are required for the computation of resonance Raman spectra. Steps 3.1–3.3 are optional but were useful within the context of the benchmark study.
- Functionals that provide more accurate energetics, manifest in their UV–Vis spectra or 0–0 transitions, do not guarantee the quality of the obtained vibrational spectra. Strategies such as the incident light compensation scheme applied to the pre‐resonant spectra, or the scaling of the frequencies, can correct for such errors.
- The usage of diffuse functions in the chosen basis set is recommended, as it is for all the excited state calculations, but can be avoided, specifically in the case of large protein cluster calculations, with a careful choice of the DFT functional. Furthermore, as was shown in the B3LYP basis set dependence study, augmentation of the double‐zeta basis set is preferable rather than an increase in size to triple‐ or quadruple‐ζ sets.
- In the absence of computed S‐S or T‐T transitions within the selected resonance window, augmenting the basis set can solve the issue. Moreover, state crossing during excited state optimizations can possibly be alleviated by the augmentation.
- In case of established marker bands in the studied system, these can help in the definition of a specific scaling factor, which in turn can assist in the assignments of off‐Resonance spectra. Long range corrected functionals, in particular, required scaling of their frequencies and re‐assignment. This process improved markedly the correlation compared to a mere application of the scaling factor to the correlation established by the unscaled frequencies. Scaling was not applied to the computed resonance Raman spectra, and assignments were based only on the resonance enhanced intensities yielding correlation between single computed and experimental peaks.
- Setting the incident energy to the reference/resonant 0–0 transition is an acceptable compromise, provided the energy differences are not large. Inclusion of the actual experimental value (< 3000 cm^−1^ difference for most functionals) had impact only in the absolute and not the relative intensities in this study.
- No particular class of DFT functionals has a marked advantage in rR calculations, as members of the GGA, hybrid, and meta‐hybrid categories performed well in the benchmark. However, in the case of hybrid functionals, a small percentage of HF exchange seems more favorable, judging from the performance of functionals such as BMK and BHandHLYP with over 40% HF exchange.
- If the resonance conditions are not fully met, as was the case for the triplet state of FMN in the experiment employed here, pre‐resonance spectra provide a better agreement than the equivalent resonance Raman [20]. Inspection of the experimental Transient Absorption spectrum can establish the actual conditions.
- Peak shifts between states can be predicted and rationalized according to the contributing normal stretching modes.

The results presented here should be, conceivably, an encouragement for further testing in more complex systems such as flavin embedded in LOV or BLUF domains, and also other chromophore/photoprotein systems.

## Conflicts of Interest

The authors declare no conflicts of interest.

## Supporting information


**Table S1:** DFT functionals included in the study, short description and citations.
**Table S2:** Dispersion correction terms included for each DFT functional.
**Table S3:** Literature and custom scaling factors for each DFT functional.
**Table S4:** Comparison of Excitation energies of DFT functionals with the Experimental.
**Figure S1:** Agreement chart between the experimental Absorption bands of FMN and the calculated excitation energies for all the DFT functionals.
**Figure S2:** Simulated UV–Vis spectra of all the DFT functionals.
**Table S5:** Typical vibrations of lumiflavin in the fingerprint region.
**Table S6:** 0–0 Transitions and adiabatic energies between the S_0_, S_1_ states of all DFT functionals.
**Figure S3:** Relationship between the offR correlation and 0–0 transition percent errors for the 19 DFT functionals scaled with the Sc_S_ factor.
**Tables S7‐S8:** 0–0 Shifts and dipole strengths of vibronic transitions of all DFT functionals.
**Figure S4:** Vibronic spectra based on the OPA calculation of the S_0_ → S_1_ excitation.
**Table S9:** Excitations from S_1_ to higher r_n_ singlet states within the Resonance window.
**Table S10:** Hole/Electron properties of the S_1_ and S_n_ states calculated for each DFT functional.
**Table S11:** Hole and Electron surfaces for resonant states calculated for each DFT functional.
**Table S12:** Assignment Tables between the peaks of the experimental FSRS 1FMN* 3^rd^ EAS Spectrum and the calculated S_1_ (off‐Resonance) spectra of each DFT functional.
**Table S13:** Assignment Tables between the peaks of the experimental FSRS 1FMN* 3^rd^ EAS Spectrum and the calculated Resonance spectra of each DFT functional.
**Figure S5:** Calculated excited singlet (off‐)resonance Raman spectra of all DFT functionals.
**Table S14:** Choice of excitations from T_1_ to higher T_n_ states within the Resonance window.
**Table S15:** Hole/Electron properties of the T_n_ resonant states of each DFT functional.
**Table S16:** Hole/Electron surfaces for the T_n_ resonant states calculated for each DFT functional.
**Table S17:** Assignment Tables between the peaks of the experimental FSRS 3FMN* 5^th^ EAS Spectrum and the calculated T_1_ (off‐resonance) Raman spectra of selected DFT functionals.
**Table S18:** Assignment Tables between the peaks of the experimental FSRS 3FMN* 5^th^ EAS Spectrum and the calculated (pre‐)resonance Raman spectra of selected DFT functionals.
**Table S19:** Statistical analysis of the correlation of the calculated triplet off‐, pre‐ and resonance Raman spectra with the experimental 5^th^ EAS assigned to the 3FMN* state.
**Table S20:** Singlet‐Triplet Shifts between the excited singlet resonance Raman and the off‐, pre‐and resonance Raman triplet state spectra of the selected DFT functionals.
**Figure S6:** Calculated excited triplet off‐, pre‐ and resonance spectra of DFT functionals.
**Figure S7:** Relationship between averaged C=O bond lengths and their v(s)/v(as) stretching frequencies for the selected DFT functionals calculated at the T_1_ state.
**Table S21:** Evaluation of DFT functionals by five criteria based on excited triplet calculations.
**Table S22:** Prediction of S‐T peak shifts according to displacement and PED assignments.

## Data Availability

The data that support the findings of this study are available from the corresponding author upon reasonable request.

## References

[1] S. Weber and E. E. Schleicher , Flavins and Flavoproteins Methods and Protocols (Springer, 2014).

[2] A. Losi and W. Gärtner , “Solving Blue Light Riddles: New Lessons From Flavin‐bindingLOVPhotoreceptors,” Photochemistry and Photobiology 93, no. 1 (2017): 141–158. 27861974 10.1111/php.12674

[3] T. Fujisawa and S. Masuda , “Light‐Induced Chromophore and Protein Responses and Mechanical Signal Transduction of BLUF Proteins,” Biophysical Reviews 10, no. 2 (2018): 327–337. 29235080 10.1007/s12551-017-0355-6 PMC5899715

[4] V. Massey , S. Strickland , S. G. Mayhew , et al., “The Production of Superoxide Anion Radicals in the Reaction of Reduced Flavins and Flavoproteins With Molecular Oxygen,” Biochemical and Biophysical Research Communications 36, no. 6 (1969): 891–897. 5388670 10.1016/0006-291x(69)90287-3

[5] A. S. Chaudhari , A. Chatterjee , C. A. O. Domingos , et al., “Genetically Encoded Non‐Canonical Amino Acids Reveal Asynchronous Dark Reversion of Chromophore, Backbone, and Side‐Chains inEL222,” Protein Science 32, no. 4 (2023): e4590. 36764820 10.1002/pro.4590 PMC10019195

[6] G. I. Morozov , N. Porat , T. Kushnir , et al., “Flavin Reductase Contributes to Pneumococcal Virulence by Protecting From Oxidative Stress and Mediating Adhesion and Elicits Protection Against Pneumococcal Challenge,” Scientific Reports 8, no. 1 (2018): 314. 29321514 10.1038/s41598-017-18645-8 PMC5762878

[7] K. Zenichowski , M. Gothe , and P. Saalfrank , “Exciting Flavins: Absorption Spectra and Spin–Orbit Coupling in Light–Oxygen–Voltage (LOV) Domains,” Journal of Photochemistry and Photobiology, A: Chemistry 190, no. 2–3 (2007): 290–300.

[8] S. Salzmann , V. Martinez‐Junza , B. Zorn , et al., “Photophysical Properties of Structurally and Electronically Modified Flavin Derivatives Determined by Spectroscopy and Theoretical Calculations,” Journal of Physical Chemistry. A 113, no. 33 (2009): 9365–9375. 19639947 10.1021/jp905724b

[9] Y. Orozco‐Gonzalez , M. P. Kabir , and S. Gozem , “Electrostatic Spectral Tuning Maps for Biological Chromophores,” Journal of Physical Chemistry B 123, no. 23 (2019): 4813–4824. 30869891 10.1021/acs.jpcb.9b00489

[10] R. K. Kar , V. A. Borin , Y. Ding , J. Matysik , and I. Schapiro , “Spectroscopic Properties of Lumiflavin: A Quantum Chemical Study,” Photochemistry and Photobiology 95, no. 2 (2019): 662–674. 30257038 10.1111/php.13023

[11] C. Neiss , P. Saalfrank , M. Parac , and S. Grimme , “Quantum Chemical Calculation of Excited States of Flavin‐Related Molecules,” Journal of Physical Chemistry. A 107, no. 1 (2002): 140–147.

[12] P. C. Andrikopoulos , A. S. Chaudhari , Y. Liu , et al., “QM Calculations Predict the Energetics and Infrared Spectra of Transient Glutamine Isomers in LOV Photoreceptors,” Physical Chemistry Chemical Physics 23, no. 25 (2021): 13934–13950. 34142688 10.1039/d1cp00447f PMC8246142

[13] Y. Hontani , J. Mehlhorn , T. Domratcheva , et al., “Spectroscopic and Computational Observation of Glutamine Tautomerization in the Blue Light Sensing Using Flavin Domain Photoreaction,” Journal of the American Chemical Society 145, no. 2 (2023): 1040–1052. 36607126 10.1021/jacs.2c10621 PMC9853863

[14] C. R. Hall , J. Tolentino Collado , J. N. Iuliano , et al., “Site‐Specific Protein Dynamics Probed by Ultrafast Infrared Spectroscopy of a Noncanonical Amino Acid,” Journal of Physical Chemistry. B 123, no. 45 (2019): 9592–9597. 31596584 10.1021/acs.jpcb.9b09425

[15] L. Goett‐Zink , L. Karsten , C. Mann , et al., “Photochemistry of Receptor‐Bound Flavin Resolved in Living Human Cells by Infrared Spectroscopy,” Journal of the American Chemical Society 147, no. 11 (2025): 9676–9685. 40054855 10.1021/jacs.4c17815 PMC11926855

[16] G. Batignani , C. Ferrante , G. Fumero , M. Martinati , and T. Scopigno , “Femtosecond Stimulated Raman Spectroscopy,” Nature Reviews Methods Primers 4 (2024): 1.

[17] Z. Wang , Y. Zhang , C. Chen , et al., “Mapping the Complete Photocycle That Powers a Large Stokes Shift Red Fluorescent Protein,” Angewandte Chemie (International Ed. in English) 62, no. 5 (2023): e202212209. 36440527 10.1002/anie.202212209

[18] P. Chrupkova , I. H. M. van Stokkum , T. Friedrich , et al., “Raman Vibrational Signatures of Excited States of Echinenone in the Orange Carotenoid Protein (OCP) and Implications for Its Photoactivation Mechanism,” Journal of Molecular Biology 436, no. 16 (2024): 168625. 38797429 10.1016/j.jmb.2024.168625

[19] Y. Liu , A. S. Chaudhari , A. Chatterjee , et al., “Sub‐Millisecond Photoinduced Dynamics of Free and EL222‐Bound FMN by Stimulated Raman and Visible Absorption Spectroscopies,” Biomolecules 13, no. 1 (2023): 161. 36671546 10.3390/biom13010161 PMC9855911

[20] P. C. Andrikopoulos , Y. Liu , A. Picchiotti , et al., “Femtosecond‐To‐Nanosecond Dynamics of Flavin Mononucleotide Monitored by Stimulated Raman Spectroscopy and Simulations,” Physical Chemistry Chemical Physics 22, no. 12 (2020): 6538–6552. 31994556 10.1039/c9cp04918e

[21] T. Domratcheva , E. Hartmann , I. Schlichting , and T. Kottke , “Evidence for Tautomerisation of Glutamine in BLUF Blue Light Receptors by Vibrational Spectroscopy and Computational Chemistry,” Scientific Reports 6 (2016): 22669. 26947391 10.1038/srep22669 PMC4780082

[22] X. P. Chang , Y. J. Gao , W. H. Fang , G. Cui , and W. Thiel , “Quantum Mechanics/Molecular Mechanics Study on the Photoreactions of Dark‐ and Light‐Adapted States of a Blue‐Light YtvA LOV Photoreceptor,” Angewandte Chemie (International Ed. in English) 56, no. 32 (2017): 9341–9345. 28632317 10.1002/anie.201703487

[23] S. Gozem , F. Melaccio , H. L. Luk , S. Rinaldi , and M. Olivucci , “Learning From Photobiology How to Design Molecular Devices Using a Computer,” Chemical Society Reviews 43, no. 12 (2014): 4019–4036. 24811294 10.1039/c4cs00037d

[24] A. Baiardi , J. Bloino , and V. Barone , “A General Time‐Dependent Route to Resonance‐Raman Spectroscopy Including Franck‐Condon, Herzberg‐Teller and Duschinsky Effects,” Journal of Chemical Physics 141, no. 11 (2014): 114108. 25240346 10.1063/1.4895534 PMC4608049

[25] F. Zutterman , V. Liegeois , and B. Champagne , “TDDFT Investigation of the Raman and Resonant Raman Spectra of Fluorescent Protein Chromophore Models,” Journal of Physical Chemistry. B 126, no. 18 (2022): 3414–3424. 35499480 10.1021/acs.jpcb.2c01202

[26] M. S. Barclay , C. G. Elles , and M. Caricato , “Benchmark Study of Ground‐State Raman Spectra in Conjugated Molecules,” Journal of Chemical Theory and Computation 16, no. 1 (2020): 612–620. 31790252 10.1021/acs.jctc.9b00960

[27] M. S. Barclay , T. J. Quincy , D. B. Williams‐Young , M. Caricato , and C. G. Elles , “Accurate Assignments of Excited‐State Resonance Raman Spectra: A Benchmark Study Combining Experiment and Theory,” Journal of Physical Chemistry. A 121, no. 41 (2017): 7937–7946. 28953391 10.1021/acs.jpca.7b09467

[28] J. S. Sandoval and D. W. McCamant , “The Best Models of Bodipy's Electronic Excited State: Comparing Predictions From Various DFT Functionals With Measurements From Femtosecond Stimulated Raman Spectroscopy,” Journal of Physical Chemistry. A 127, no. 39 (2023): 8238–8251. 37751471 10.1021/acs.jpca.3c05040 PMC10561280

[29] M. Staniszewska , S. Kupfer , M. Labuda , and J. Guthmuller , “Theoretical Assessment of Excited State Gradients and Resonance Raman Intensities for the Azobenzene Molecule,” Journal of Chemical Theory and Computation 13, no. 3 (2017): 1263–1274. 28118003 10.1021/acs.jctc.6b00966

[30] U. Ozuguzel , A. J. A. Aquino , R. Nieman , S. D. Minteer , and C. Korzeniewski , “Resonance Raman Spectra and Excited State Properties of Methyl Viologen and Its Radical Cation From Time‐Dependent Density Functional Theory,” Journal of Computational Chemistry 44, no. 31 (2023): 2414–2423. 37615205 10.1002/jcc.27207

[31] A. D. Becke , “Density‐Functional Exchange‐Energy Approximation With Correct Asymptotic Behavior,” Physical Review A 38, no. 6 (1988): 3098–3100. 10.1103/physreva.38.3098 9900728

[32] C. Lee , W. Yang , and R. G. Parr , “Development of the Colle‐Salvetti Correlation‐Energy Formula Into a Functional of the Electron Density,” Physical Review B 37, no. 2 (1988): 785–789. 10.1103/physrevb.37.785 9944570

[33] A. Weigel , A. Dobryakov , B. Klaumunzer , M. Sajadi , P. Saalfrank , and N. P. Ernsting , “Femtosecond Stimulated Raman Spectroscopy of Flavin After Optical Excitation,” Journal of Physical Chemistry. B 115, no. 13 (2011): 3656–3680. 21410155 10.1021/jp1117129

[34] J. N. Iuliano , C. R. Hall , D. Green , et al., “Excited State Vibrations of Isotopically Labeled FMN Free and Bound to a Light–Oxygen–Voltage (LOV) Protein,” Journal of Physical Chemistry. B 124, no. 33 (2020): 7152–7165. 32786715 10.1021/acs.jpcb.0c04943 PMC7533957

[35] D. Green , P. Roy , C. R. Hall , et al., “Excited State Resonance Raman of Flavin Mononucleotide: Comparison of Theory and Experiment,” Journal of Physical Chemistry. A 125, no. 28 (2021): 6171–6179. 34240863 10.1021/acs.jpca.1c04063 PMC8791451

[36] M. J. Frisch , G. W. Trucks , H. B. Schlegel , et al., Gaussian 16, Revision C.01 (Gaussian, Inc, 2016).

[37] S. Grimme , S. Ehrlich , and L. Goerigk , “Effect of the Damping Function in Dispersion Corrected Density Functional Theory,” Journal of Computational Chemistry 32, no. 7 (2011): 1456–1465. 21370243 10.1002/jcc.21759

[38] S. Grimme , “List of Functionals and Coefficients for BJ‐Damping,” https://www.chemie.uni‐bonn.de/grimme/de/software/dft‐d3/bj_damping.

[39] “Computational Chemistry Comparison and Benchmark DataBase,” https://cccbdb.nist.gov/vibscalejustx.asp.

[40] M. L. Laury , S. E. Boesch , I. Haken , P. Sinha , R. A. Wheeler , and A. K. Wilson , “Harmonic Vibrational Frequencies: Scale Factors for Pure, Hybrid, Hybrid Meta, and Double‐Hybrid Functionals in Conjunction With Correlation Consistent Basis Sets,” Journal of Computational Chemistry 32, no. 11 (2011): 2339–2347. 21598273 10.1002/jcc.21811

[41] M. K. Kesharwani , B. Brauer , and J. M. Martin , “Frequency and Zero‐Point Vibrational Energy Scale Factors for Double‐Hybrid Density Functionals (And Other Selected Methods): Can Anharmonic Force Fields Be Avoided?,” Journal of Physical Chemistry A 119, no. 9 (2015): 1701–1714. 25296165 10.1021/jp508422u

[42] H. S. Yu , L. J. Fiedler , I. M. Alecu , S. Kanchanakungwankul , and D. G. Truhlar , “FREQ v.2,” (2021).

[43] I. M. Alecu , J. Zheng , Y. Zhao , and D. G. Truhlar , “Computational Thermochemistry: Scale Factor Databases and Scale Factors for Vibrational Frequencies Obtained From Electronic Model Chemistries,” Journal of Chemical Theory and Computation 6, no. 9 (2010): 2872–2887. 26616087 10.1021/ct100326h

[44] H. S. Yu , L. J. Fiedler , I. M. Alecu , and D. G. Truhlar , “Computational Thermochemistry: Automated Generation of Scale Factors for Vibrational Frequencies Calculated by Electronic Structure Model Chemistries,” Computer Physics Communications 210 (2017): 132–138.

[45] T. H. Dunning , “Gaussian Basis Sets for Use in Correlated Molecular Calculations. I. The Atoms Boron Through Neon and Hydrogen,” Journal of Chemical Physics 90, no. 2 (1989): 1007–1023.

[46] E. R. Davidson , “Comment on Comment on Dunning's Correlation‐Consistent Basis Sets,” Chemical Physics Letters 260, no. 3–4 (1996): 514–518.

[47] R. A. Kendall , T. H. Dunning , and R. J. Harrison , “Electron Affinities of the First‐Row Atoms Revisited. Systematic Basis Sets and Wave Functions,” Journal of Chemical Physics 96, no. 9 (1992): 6796–6806.

[48] D. E. Woon and T. H. Dunning , “Gaussian Basis Sets for Use in Correlated Molecular Calculations. III. The Atoms Aluminum Through Argon,” Journal of Chemical Physics 98, no. 2 (1993): 1358–1371.

[49] J. Tomasi , B. Mennucci , and R. Cammi , “Quantum Mechanical Continuum Solvation Models,” Chemical Reviews 105, no. 8 (2005): 2999–3093. 16092826 10.1021/cr9904009

[50] G. Scalmani and M. J. Frisch , “Continuous Surface Charge Polarizable Continuum Models of Solvation. I. General Formalism,” Journal of Chemical Physics 132, no. 11 (2010): 114110. 20331284 10.1063/1.3359469

[51] M. Cossi , V. Barone , R. Cammi , and J. Tomasi , “Ab Initio Study of Solvated Molecules: A New Implementation of the Polarizable Continuum Model,” Chemical Physics Letters 255, no. 4–6 (1996): 327–335.

[52] G. Scalmani , M. J. Frisch , B. Mennucci , J. Tomasi , R. Cammi , and V. Barone , “Geometries and Properties of Excited States in the Gas Phase and in Solution: Theory and Application of a Time‐Dependent Density Functional Theory Polarizable Continuum Model,” Journal of Chemical Physics 124, no. 9 (2006): 94107. 16526845 10.1063/1.2173258

[53] F. Furche and R. Ahlrichs , “Adiabatic Time‐Dependent Density Functional Methods for Excited State Properties,” Journal of Chemical Physics 117, no. 16 (2002): 7433–7447.

[54] J. Liu and W. Liang , “Analytical Approach for the Excited‐State Hessian in Time‐Dependent Density Functional Theory: Formalism, Implementation, and Performance,” Journal of Chemical Physics 135, no. 18 (2011): 184111. 22088056 10.1063/1.3659312

[55] J. Liu and W. Liang , “Analytical Hessian of Electronic Excited States in Time‐Dependent Density Functional Theory With Tamm‐Dancoff Approximation,” Journal of Chemical Physics 135, no. 1 (2011): 014113. 21744894 10.1063/1.3605504

[56] V. Barone , J. Bloino , M. Biczysko , and F. Santoro , “Fully Integrated Approach to Compute Vibrationally Resolved Optical Spectra: From Small Molecules to Macrosystems,” Journal of Chemical Theory and Computation 5, no. 3 (2009): 540–554. 26610221 10.1021/ct8004744

[57] J. Bloino , M. Biczysko , F. Santoro , and V. Barone , “General Approach to Compute Vibrationally Resolved One‐Photon Electronic Spectra,” Journal of Chemical Theory and Computation 6, no. 4 (2010): 1256–1274. 10.1021/ct8004744 26610221

[58] F. Santoro , A. Lami , R. Improta , and V. Barone , “Effective Method to Compute Vibrationally Resolved Optical Spectra of Large Molecules at Finite Temperature in the Gas Phase and in Solution,” Journal of Chemical Physics 126, no. 18 (2007): 184102. 17508787 10.1063/1.2721539

[59] F. Santoro , R. Improta , A. Lami , J. Bloino , and V. Barone , “Effective Method to Compute Franck‐Condon Integrals for Optical Spectra of Large Molecules in Solution,” Journal of Chemical Physics 126, no. 8 (2007): 084509. 17343460 10.1063/1.2437197

[60] F. Santoro , A. Lami , R. Improta , J. Bloino , and V. Barone , “Effective Method for the Computation of Optical Spectra of Large Molecules at Finite Temperature Including the Duschinsky and Herzberg–Teller Effect: The Qx Band of Porphyrin as a Case Study,” Journal of Chemical Physics 128, no. 22 (2008): 224311. 18554017 10.1063/1.2929846

[61] K. Huang and A. Rhys , “Proceedings of the Royal Society of London,” Series A, Mathematical and Physical Sciences 204, no. 1078 (1950): 406–423.

[62] P. Pulay , “Second and Third Derivatives of Variational Energy Expressions: Application to Multiconfigurational Self‐Consistent Field Wave Functions,” Journal of Chemical Physics 78, no. 8 (1983): 5043–5051.

[63] C. E. Dykstra and P. G. Jasien , “Derivative Hartree—Fock Theory to All Orders,” Chemical Physics Letters 109, no. 4 (1984): 388–393.

[64] M. H. Jamroz , “Vibrational Energy Distribution Analysis (VEDA): Scopes and Limitations,” Spectrochimica Acta. Part A, Molecular and Biomolecular Spectroscopy 114 (2013): 220–230. 23778167 10.1016/j.saa.2013.05.096

[65] F. Menges , “Spectragryph—Optical Spectroscopy Software,” https://www.effemm2.de/spectragryph/.

[66] A. K. Narsaria , J. D. Ruijter , T. A. Hamlin , et al., “Performance of TDDFT Vertical Excitation Energies of Core‐Substituted Naphthalene Diimides,” Journal of Computational Chemistry 41, no. 15 (2020): 1448–1455. 32142173 10.1002/jcc.26188 PMC7317478

[67] F. Egidi , J. Bloino , C. Cappelli , and V. Barone , “A Robust and Effective Time‐Independent Route to the Calculation of Resonance Raman Spectra of Large Molecules in Condensed Phases with the Inclusion of Duschinsky, Herzberg–Teller, Anharmonic, and Environmental Effects,” Journal of Chemical Theory and Computation 10, no. 1 (2013): 346–363. 10.1021/ct400932e PMC4632188 26550003

[68] M. Sun , T. A. Moore , and P. S. Song , “Molecular Luminescence Studies of Flavins. I. The Excited States of Flavins,” Journal of the American Chemical Society 94, no. 5 (1972): 1730–1740. 5015676 10.1021/ja00760a052

[69] B. Klaumunzer , D. Kroner , and P. Saalfrank , “(TD‐)DFT Calculation of Vibrational and Vibronic Spectra of Riboflavin in Solution,” Journal of Physical Chemistry. B 114, no. 33 (2010): 10826–10834. 20681576 10.1021/jp100642c

[70] T. Lu and F. Chen , “Multiwfn: A Multifunctional Wavefunction Analyzer,” Journal of Computational Chemistry 33, no. 5 (2012): 580–592. 22162017 10.1002/jcc.22885

[71] R. Xu , Z. Jiang , Q. Yang , J. Bloino , and M. Biczysko , “Harmonic and Anharmonic Vibrational Computations for Biomolecular Building Blocks: Benchmarking DFT and Basis Sets by Theoretical and Experimental IR Spectrum of Glycine Conformers,” Journal of Computational Chemistry 45, no. 21 (2024): 1846–1869. 38682874 10.1002/jcc.27377

[72] C. Adamo and D. Jacquemin , “The Calculations of Excited‐State Properties With Time‐Dependent Density Functional Theory,” Chemical Society Reviews 42, no. 3 (2013): 845–856. 23117144 10.1039/c2cs35394f

