# Supplementary material for: Extensive TDDFT Benchmark Study of the Resonance Raman Spectra of Lumiflavin
Source: J Comput Chem. 2025 Oct 4;46(26):e70229. doi: 10.1002/jcc.70229 (PMC12495946; doi:10.1002/jcc.70229)
Supplement: Supplementary file 1 — Table S1: DFT functionals included in the study, short description and citations. Table S2: Dispersion correction terms included for each DFT functional. Table S3: Literature and custom scaling factors for each DFT functional. Table S4: Comparison of Excitation energies of DFT functionals with the Experimental. Figure S1: Agreement chart between the experimental Absorption bands of FMN and the calculated excitation energies for all the DFT functionals. Figure S2: Simulated UV–Vis spectra of all the DFT functionals. Table S5: Typical vibrations of lumiflavin in the fingerprint region. Table S6: 0–0 Transitions and adiabatic energies between the S0, S1 states of all DFT functionals. Figure S3: Relationship between the offR correlation and 0–0 transition percent errors for the 19 DFT functionals scaled with the ScS factor. Tables S7‐S8: 0–0 Shifts and dipole strengths of vibronic transitions of all DFT functionals. Figure S4: Vibronic spectra based on the OPA calculation of the S0 → S1 excitation. Table S9: Excitations from S1 to higher rn singlet states within the Resonance window. Table S10: Hole/Electron properties of the S1 and Sn states calculated for each DFT functional. Table S11: Hole and Electron surfaces for resonant states calculated for each DFT functional. Table S12: Assignment Tables between the peaks of the experimental FSRS 1FMN* 3rd EAS Spectrum and the calculated S1 (off‐Resonance) spectra of each DFT functional. Table S13: Assignment Tables between the peaks of the experimental FSRS 1FMN* 3rd EAS Spectrum and the calculated Resonance spectra of each DFT functional. Figure S5: Calculated excited singlet (off‐)resonance Raman spectra of all DFT functionals. Table S14: Choice of excitations from T1 to higher Tn states within the Resonance window. Table S15: Hole/Electron properties of the Tn resonant states of each DFT functional. Table S16: Hole/Electron surfaces for the Tn resonant states calculated for each DFT functional. Table S17: Assignment Table [file JCC-46-0-s001.pdf]

ELECTRONIC SUPPLEMENTARY INFORMATION

Extensive TDDFT Benchmark Study of the Resonance Raman Spectra of Lumiflavin

Prokopis C. Andrikopoulos,*[a,b] Heba Halimeh[c,d]

[a] Charles University, First Faculty of Medicine, BIOCEV, Průmyslová 595, CZ-252 50 Vestec, Czechia
[b] Institute of Biotechnology of the Czech Academy of Sciences, BIOCEV, Průmyslová 595, CZ-252 50 Vestec, Czechia
[c] Rhine-Waal University of Applied Sciences, Marie-Curie-Str.1 D-47533 Kleve, Germany
[d] Current Address:Goethe University, Faculty of Biological Sciences, Max-von-Laue-Str.9, Frankfurt am Main, 60439, Germany

* Correspondence to: Prokopis C. Andrikopoulos; e-mail: prokopios.andrikopoulos@lf1.cuni.cz

CONTENTS

1. Classification, Dispersion and Scaling factors of DFT Functionals

Page 2 **Table S1** DFT functionals included in the study, short description and citations.
Page 3 **Table S2** Dispersion correction terms included for each DFT functional.
Page 4 **Table S3** Literature and custom scaling factors for each DFT functional.

2. Time-Independent and Time-Dependent Formalism implementation in Gaussian

Page 5 **2.1** One-Photon Absorption Spectra.
Page 6 **2.2** Resonance Raman Spectra.

3. Excited Singlet State of lumiflavin

Page 7-8 **Table S4** Comparison of Excitation energies of DFT functionals with the Experimental Absorption bands of FMN.
Page 8 **Figure S1** Agreement chart between the experimental Absorption bands of FMN and the calculated excitation energies for all the DFT functionals.
Page 9-10 **Figure S2** Simulated UV-Vis spectra of all the DFT functionals.
Page 10 **Table S5** Typical vibrations of lumiflavin in the fingerprint region.
Page 11-12 **Table S6** 0-0 Transitions and adiabatic energies between the S_0, S_1 states of all DFT functionals.
Page 12 **Figure S3** Relationship between the offR correlation and 0-0 transition percent errors for the 19 DFT functionals scaled with the **Sc_S** factor.
Page 13-14 **Tables S7-S8** 0-0 Shifts and dipole strengths of vibronic transitions of all DFT functionals.
Page 15-17 **Figure S4** Vibronic spectra based on the OPA calculation of the $S_0 \rightarrow S_1$ excitation.
Page 18-19 **Table S9** Excitations from S_1 to higher r_n singlet states within the Resonance window.
Page 20-22 **Table S10** Hole/Electron properties of the S_1 and S_n states calculated for each DFT functional.
Page 23-24 **Table S11** Hole and Electron surfaces for resonant states calculated for each DFT functional.
Page 24-38 **Table S12** Assignment Tables between the peaks of the experimental FSRS 1FMN* 3rd EAS Spectrum and the calculated S_1 (off-Resonance) spectra of each DFT functional.
Page 39-49 **Table S13** Assignment Tables between the peaks of the experimental FSRS 1FMN* 3rd EAS Spectrum and the calculated Resonance spectra of each DFT functional.
Page 50-52 **Figure S5** Calculated excited singlet (off-)resonance Raman spectra of all DFT functionals.

4. Excited Triplet State of lumiflavin with selected DFT functionals

Page 53 **Table S14** Choice of excitations from T_1 to higher T_n states within the Resonance window.
Page 54 **Table S15** Hole/Electron properties of the T_n resonant states of each DFT functional.
Page 55 **Table S16** Hole/Electron surfaces for the T_n resonant states calculated for each DFT functional.
Page 56-57 **Table S17** Assignment Tables between the peaks of the experimental FSRS 3FMN* 5th EAS Spectrum and the calculated T_1 (off-resonance) Raman spectra of selected DFT functionals.
Page 57-60 **Table S18** Assignment Tables between the peaks of the experimental FSRS 3FMN* 5th EAS Spectrum and the calculated (pre-)resonance Raman spectra of selected DFT functionals.
Page 61 **Table S19** Statistical analysis of the correlation of the calculated triplet off-, pre- and resonance Raman spectra with the experimental 5th EAS assigned to the 3FMN* state.
Page 62 **Table S20** Singlet-Triplet Shifts between the excited singlet resonance Raman and the off-, pre- and resonance Raman triplet state spectra of the selected DFT functionals.
Page 63-65 **Figure S6** Calculated excited triplet off-, pre- and resonance spectra of DFT functionals.
Page 66 **Figure S7** Relationship between averaged C=O bond lengths and their v(s)/v(as) stretching frequencies for the selected DFT functionals calculated at theT_1 state.
Page 67-68 **Table S21** Evaluation of DFT functionals by five criteria based on excited triplet calculations.
Page 69 **Table S22** Prediction of S-T peak shifts according to displacement and PED assignments.

5. Additional References

6. Cartesian Coordinates of all optimized species

1. Classification, Dispersion and Scaling factors of DFT Functionals

Table S1 Classification of the forty-two studied DFT functionals including their type along the DFT Jacob's Ladder, percentage of HF-exchange (% Ex.) and short description including citations.

Functional	Type	% Ex.	Description + Citations
LSDA	LDA	-	Synonym of SVWN, combining the exchange Slater **S**[1] and correlation **VWN** [2] functionals
BLYP	GGA	-	GGA functional combining the exchange **B**[3] and correlation **LYP**[4,5] functionals
BP86	GGA	-	GGA Functional combining the exchange **B**[3] and correlation **P86**[6] functionals
BPBE	GGA	-	GGA Functional combining the exchange **B**[3] and **PBE**[7,8] correlation functionals
HCTH/407	GGA	-	Handy's GGA functional including gradient-corrected correlation[9-11]
mPWLYP	GGA	-	GGA functional combining the exchange **mPW**[12] and correlation **LYP**[4,5] functionals
OLYP	GGA	-	GGA functional combining Handy's **O**PTX modification[13,14] of the B exchange and the **LYP**[4,5] correlation functional
OPBE	GGA	-	GGA functional combining Handy's **O**PTX[13,14] modification and the **PBE**[7,8] correlation functionals
SOGGA11	GGA	-	GGA functional from the Truhlar group[15]
M06L	meta-GGA	-	meta-GGA functional from the Truhlar group[16]
M11L	meta-GGA	-	meta-GGA functional from the Truhlar group[17]
revTPSS	meta-GGA	-	The revised **revTPSS** exchange and correlation functionals of Perdew *et. al.*[18,19]
TPSSTPSS	meta-GGA	-	meta-GGA functional combining the **TPSS**[20] exchange and correlation functionals of Tao, Perdew, Staroverov, and Scuseria
VSXC	meta-GGA	-	van Voorhis and Scuseria τ-dependent gradient-corrected correlation functional[21]
APFD	Hybrid	23	The **A**ustin-**F**risch-**P**etersson hybrid functional[22]
B1B95	Hybrid	28	**B**ecke's one-parameter hybrid functional[23]
B3LYP	Hybrid	20	Part of Becke Three-Parameter Hybrid Functionals[24] that follow the formulation: $A*E_X^{Slater}+(1-A)*E_X^{HF}+B*\Delta E_X^{Becke}+E_C^{VWN}+C*\Delta E_C^{non\text{-}local}$ where the non-local correlation is provided by the **LYP**,[4,5] **P86**[6] and **PW91**[25-27] correlation functionals
B3P86	Hybrid	20	
B3PW91	Hybrid	20	
B98	Hybrid	22	Becke's 1998 revisions to B97[28,29]
BHHLYP	Hybrid	50	Half-and-Half Functional proposed by Becke[30]
mPW1PW91	Hybrid	25	Hybrid Functional combining the **mPW** exchange modified by Adamo and Barone[12] combined with **PW91**[25-27] correlation functional
O3LYP	Hybrid	11.6	Hybrid three-parameter functional similar to B3LYP[24] with the A, B and C terms defined by Cohen & Handy[31]
PBE1PBE	Hybrid	25	The pure **PBE** functional[7,8] made into hybrid by Adamo[32]
SOGGA11x	Hybrid	40.1	Global hybrid functional from the Truhlar group[33]
X3LYP	Hybrid	21.8	Functional of **X**u and Goddard[34]
BMK	m-Hybrid	42	**B**oese and **M**artin's τ-dependent 2004 hybrid functional[35]
M05-2X	m-Hybrid	56	meta-Hybrid functional from the Truhlar group[36]
M06	m-Hybrid	27	meta-Hybrid functional from the Truhlar group[37]
M06-HF	m-Hybrid	27	meta-Hybrid functional from the Truhlar group[38,39]
MN15	m-Hybrid	44	meta-Hybrid functional from the Truhlar group[40]
PW6B95D3	m-Hybrid	28	meta-Hybrid functional from the Truhlar group[41]
revTPSSh	m-Hybrid	15	meta-Hybrid functional employing the revised **revTPSS** exchange and correlation functionals[18,19]
tHCTHhyb	m-Hybrid	15	Hybrid functional using the **tHCTH** functional[42]
TPSSh	m-Hybrid	10	meta-Hybrid functional using the **TPSS** functionals[20,43]
LC-OPBE	RS-GGA	-	GGA functional combining **O**[13,14] exchange and **PBE**[7,8] correlation together with the **L**ong-range **C**orrection of Hirao and coworkers[44]
CAM-B3LYP	RS-Hybrid	10	Handy and coworkers' long-range-corrected version of B3LYP using the Coulomb-attenuating method[45]
HISSbPBE	RS-Hybrid	10	Middle-range corrected hybrid employing the **HISS** functional[46]
HSEH1PBE	RS-Hybrid	25	**HSE06** range-corrected hybrid functional[47-52] employing **PBE**[7,8] correlation.
LC-wHPBE	RS-Hybrid	10	Updated version of the long-range corrected hybrid **ωPBE** functional [50,53-55]
wB97XD	RS-Hybrid	10	Long-range corrected hybrid functional from Head-Gordon and coworkers[56]

Table S2 Empirical Dispersion correction parameters utilized for each DFT functional together with the source they were obtained from.

Functional	S6	S8	SR6	ABJ1	ABJ2	Type, Source
APFD	-	-	R_{6APFD}1.050	-	-	PFD,[22] Gaussian[57]
B1B95	1.0000	1.4507	-	0.2092	5.5545	GD3BJ, Grimme[58]
B3LYP	1.0000	1.9889	-	0.3981	4.4211	GD3BJ, Gaussian[57]
B3P86	1.0000	2.4830	-	0.5410	4.3060	GD3BJ, MolSSI[59]
B3PW91	1.0000	2.8524	-	0.4312	4.4693	GD3BJ, Gaussian[57]
B98	1.0000	0.7086	-	-1.0000	6.0672	GD3BJ, MolSSI[59]
BHHLYP	1.0000	1.0354	-	0.2793	4.9615	GD3BJ, Grimme[58]
BLYP	1.0000	2.6996	-	0.4298	4.2359	GD3BJ, Gaussian[57]
BMK	1.0000	2.0860	-	0.1940	5.9197	GD3BJ, Grimme[58]
BP86	1.0000	3.2822	-	0.3946	4.8516	GD3BJ, Gaussian[57]
BPBE	1.0000	4.0728	-	0.4567	4.3908	GD3BJ, Gaussian[57]
CAM-B3LYP	1.0000	2.0674	-	0.3708	5.4743	GD3BJ, Gaussian[57]
HCTH/407	1.0000	1.0821	-	0.3563	4.3360	GD3BJ, Grimme[58]
HISSbPBE	1.0000	1.6112	-	-1.0000	7.3539	GD3BJ, MolSSI[59]
HSEH1PBE	1.0000	2.3100	-	0.3830	5.6850	GD3BJ, MolSSI[59]
LC-OPBE	1.0000	3.3816	-	0.5512	2.9444	GD3BJ, Grimme[58]
LC-wHPBE	1.0000	1.8541		0.3919	5.0897	GD3BJ, Gaussian[57]
LSDA	-	-	-	-	-	-
M05-2X	1.0000	0.0000	1.4170	-	-	GD3, Gaussian[57]
M06	1.0000	0.0000	1.3250	-	-	GD3, Gaussian[57]
M06-HF	1.0000	0.0000	1.4460	-	-	GD3, Gaussian[57]
M06L	1.0000	0.0000	1.5810	-	-	GD3, Gaussian[57]
M11L	1.0000	1.1129	2.3933	-	-	GD3[60]
MN15	1.0000	2.0971	-	0.7862	7.5923	GD3BJ, MolSSI[59]
mPW1PW91	1.0000	1.9467	-	0.3168	4.7732	GD3BJ, MolSSI[59]
mPWLYP	1.0000	2.0077	-	0.4831	4.5323	GD3BJ, Grimme[58]
O3LYP	1.0000	1.8171	-	0.0963	5.9940	GD3BJ, MolSSI[59]
OLYP	1.0000	2.6205	-	0.5299	2.8065	GD3BJ, Grimme[58]
OPBE	1.0000	3.3816	-	0.5512	2.9444	GD3BJ, Grimme[58]
PBE1PBE	1.0000	1.2177	-	0.4145	4.8593	GD3BJ, Gaussian[57]
PW6B95D3	1.0000	0.7257	-	0.2076	6.3750	GD3BJ, Gaussian[57]
revTPSSh	1.0000	1.4076	-	0.2660	5.3761	GD3BJ, MolSSI[59]
revTPSS	1.0000	1.6151	-	0.2218	5.7985	GD3BJ, MolSSI[59]
SOGGA11	-	-	-	-	-	-
SOGGA11x	1.0000	1.1426		0.1330	5.7381	GD3BJ[60]
tHCTHhyb	1.0000	1.0821	-	0.3563	4.3360	GD3BJ, Grimme[58]
TPSS1KCIS	10000	1.0542	-	-1.0000	6.0201	GD3BJ, MolSSI[59]
TPSSh	1.0000	2.2382	-	0.4529	4.6550	GD3BJ, Grimme[58]
TPSSTPSS	1.0000	1.9435	-	0.4535	4.4752	GD3BJ, Gaussian[57]
VSXC	-	-	-	-	-	-
wB97XD	1.0000	-	1.1000	-	-	GD2, Gaussian[57]
X3LYP	1.0000	1.5744	-	0.2022	5.4184	GD3BJ, MolSSI[59]

Table S3 Scaling factors (**Sc_L**) utilized to correct the spectra of each DFT functional along with the source they were obtained from. The specific scaling factor **Sc_S** is applied to each DFT calculated spectrum in order to align the v_{75} C=O symmetric stretch vibration to the experimental FSRS 1FMN* 3rd EAS value of 1626 cm^{-1}.[61]

Functional	Scaling Factor (Sc_L) cc-pVDZ/aug-cc-pVDZ	Specific Sc.F. (Sc_S) cc-pVDZ/aug-cc-pVDZ	Source
APFD	0.9545	0.9322	Calculated in this work with FREQ[62-64]
B1B95	0.9612	0.9222	CCCBDB[65]
B3LYP	0.9700/0.9704/0.9571/ 0.9585/1.0000	0.9461/0.9776/0.9647/ 0.9769/0.9706	CCCBDB[65]
B3P86	0.9572	0.9334	Laury *et al*[66]
B3PW91	0.9650	0.9352	CCCBDB[65]
B98	0.9710	0.9383	Tantirungrotechai *et al*[67]
BHHLYP	0.9328/0.9326	0.8922/0.9210	Laury *et al*[66]
BLYP	1.0016	0.9896	CCCBDB[65]
BMK	0.9588/0.9588	0.8985/0.9277	Merrick *et al*[68]
BP86	1.0006	0.9764	Kesharwani *et al*[69]
BPBE	0.9869	0.9728	Calculated in this work with FREQ[62-64]
CAM-B3LYP	0.9530	0.9160	Calculated in this work with FREQ[62-64]
HCTH/407	0.9721	0.9502	Laury *et al*[66]
HISSbPBE	0.9283	0.8971	Calculated in this work with FREQ[62-64]
HSEH1PBE	0.9619	0.9272	CCCBDB[65]
LC-OPBE	0.9300	0.8554	Calculated in this work with FREQ[62-64]
LC-wHPBE	0.9417	0.8932	Calculated in this work with FREQ[62-64]
LSDA	0.9890/0.9887	0.9401/0.9554	CCCBDB[65]
M05-2X	0.9495/0.9501	-/0.9395	Laury *et al*[66]
M06	0.9670/0.9675	0.9187/0.9468	Laury *et al*[66]
M06-HF	0.9584	0.9187	Calculated in this work with FREQ[62-64]
M06L	0.9630/0.9630	0.9232/0.9593	Kesharwani *et al*[69]/Palafox[70]
M11L	0.9616	0.9092	Calculated in this work with FREQ[62-64]
MN15	0.9512/0.9563	0.9148/0.9441	Calculated in this work with FREQ[62-64]
mPW1PW91	0.9583	0.9249	CCCBDB[65]
mPWLYP	0.9953	0.9890	Calculated in this work with FREQ[62-64]
O3LYP	0.9696	0.9427	Tantirungrotechai *et al*[67]
OLYP	0.9875	0.9581	Tantirungrotechai *et al*[67]
OPBE	0.9702	0.9428	Calculated in this work with FREQ[62-64]
PBE1PBE	0.9615	0.9242	CCCBDB[65]
PW6B95D3	0.9502	0.9252	Calculated in this work with FREQ[62-64]
revTPSSh	-/0.9239	-/0.8947	Calculated in this work with FREQ[62-64]
revTPSS	-/0.9798	-/0.9993	Calculated in this work with FREQ[62-64]
SOGGA11	0.9788	0.9580	Calculated in this work with FREQ[62-64]
SOGGA11x	0.9403	0.9065	Calculated in this work with FREQ[62-64]
tHCTHhyb	0.9663	0.9449	Calculated in this work with FREQ[62-64]
TPSS1KCIS	0.9767	-	Calculated in this work with FREQ[62-64]
TPSSh	0.9720	0.9532	CCCBDB[65]
TPSS	0.9756/0.9801	0.9593/1.0018	Calculated in this work with FREQ[62-64]
VSXC	0.9770/0.9758	0.9424/0.9580	Tantirungrotechai *et al*[67]
wB97XD	0.9526	0.9124	CCCBDB[65]
X3LYP	0.9614	0.9423	Calculated in this work with FREQ[62-64]

2. Time-Independent and Time-Dependent Formalism implementation in Gaussian

- The one photon absorption spectra (OPA) in this work were calculated with the Time-Independent framework (TI), employing the Franck-Condon (FC) approximation of the electronic transition moments and the Adiabatic Hessian representation (AH). The equations extracted from the implementation articles by Barone and co-workers[71,72] for the Gaussian program (v.16, revC.01)[57] are outlined in **Section 2.1**.
- The Resonance Raman spectra in this work were calculated with the Time-Dependent framework (TD), employing the Franck-Condon (FC) and Franck-Condon Herzberg-Teller (FCHT) approximations using the Adiabatic Hessian representation (AH). The equations extracted from the implementation article by Baiardi *et al*,[73] for the Gaussian program (v.16, revC.01)[57] are outlined in **Section 2.2**.

2.1 One-Photon Absorption Spectra

The generalized equation governing the intensity of one-photon spectroscopies is given by:

$$I = a\omega^{\beta}\sum_{m}\sum_{n}\rho_{\gamma}[\boldsymbol{d}_{Amn}\cdot\boldsymbol{d}_{Bmn}^{*}]\delta(\omega_n-\omega_m-\omega)\ (Eq.1)$$

, where * signifies the conjugate form, δ is the Dirac function and for OPA, $I=\varepsilon(\omega), \alpha = \frac{10\pi\mathcal{N}_A}{3\varepsilon_0\ln(10)\hbar c}, \beta = 1, \gamma = m, \boldsymbol{d}_{Amn} = \boldsymbol{d}_{Bmn} = \boldsymbol{\mu}_{mn}$, ε(ω) is the molar absorption coefficient for a given angular frequency ω, $\mathcal{N}_A$ is the Avogadro constant, ε_0 is the vacuum permittivity, and $\boldsymbol{\mu}_{mn}$ is the electric transition dipole moment between the vibronic states m and n. The summation is performed over all vibrational levels of the ground and excited states m, n. To calculate $\boldsymbol{\mu}_{mn}$, a Taylor series near the equilibrium geometry of the chosen electronic state is employed:

$$\boldsymbol{\mu}_{mn}(\boldsymbol{Q}) = \boldsymbol{\mu}_{mn}(\boldsymbol{Q}_{eq}) + \sum_{k=1}^{N}(\frac{\partial\boldsymbol{\mu}_{mn}}{\partial\boldsymbol{Q}_k})_{eq}\boldsymbol{Q}_k + \ \dots,\ \ (Eq.2)$$

The FC approximation corresponds to the first term of *Eq. 2*, the HT to the second one and Franck-Condon Herzberg-Teller (FCHT) to both terms. As mentioned in the introductory paragraph, the FC approximation was employed for the calculation of the OPA, while both FC and FCHT approximations were used for the calculation of the Resonance Raman spectra in the following **Section 2.2**.

2.2 Resonance Raman Spectra

For the calculation of the resonance Raman spectra, the property of interest is the transition polarizability tensor, given below as a sum-over-states (TI):

$$\alpha_{\rho\sigma}^{if} = \frac{1}{\hbar}\sum_{n(\underline{m})}\frac{\langle\psi_{f(\underline{0})}|\,\mu_{m0,\rho}^{e}\,|\psi_{n(\underline{m})}\rangle\langle\psi_{n(\underline{m})}|\,\mu_{m0,\sigma}^{e}\,|\psi_{i(\underline{0})}\rangle}{\omega_{mi}-\omega_I-i\gamma}\ \ (Eq.3)$$

, where $\underline{0}$ represents the electronic ground state, $\underline{m}$ is the intermediate state and n($\underline{m}$) its vibrational levels, ω_{mi} is the energy separation between the initial and intermediate levels, γ a constant describing the lifetime of state m, and ω_I is the frequency of the incident light. Apart from determining $\boldsymbol{\mu}_{mn}$ as described in *Eq.2*, in order to calculate the polarizability tensor, a relation between the normal modes of the initial and intermediate electronic levels is required, given by the Duschinsky transformation:[74]

$$\overline{\boldsymbol{Q}} = \boldsymbol{J}\overline{\overline{\boldsymbol{Q}}} + \boldsymbol{K}\ (Eq.4)$$

where $\overline{\boldsymbol{Q}}$ and $\overline{\overline{\boldsymbol{Q}}}$, are normal modes of the initial and intermediate states, respectively, **J** is the Duschinsky matrix and **K** the shift vector. In the Adiabatic Hessian (AH) model, the full transformation is used, and the excited state Hessian matrix is required for computing **J**.

To transition from summation over states to time dependence, a property of the Fourier Transform is utilized:

$$\frac{1}{x} = \frac{i}{\hbar}\int_0^{+\infty} e^{-ixt/\hbar} dt \ \ (Eq.5)$$

Then the final time-dependent expression for the RR polarizability tensor expression becomes:

$$\alpha_{\rho\sigma}^{if} = \frac{i}{\hbar^2} \times \int_0^{+\infty} dt \left\langle \psi_{f(\underline{0})} \right| (\mu_{m0,\rho}^{e}\, e^{-\frac{i\hat{H}_{\underline{m}}t}{\hbar}}\, \mu_{m0,\sigma}^{e} \left| \psi_{i(\underline{0})} \right\rangle \times\, e^{-it(\omega_{ad}-\omega_I-i\gamma)}\ (Eq.6)$$

where ω_{ad} is the energy separation between the minima of the electronic ground and intermediate states, and $e^{-\frac{i\hat{H}_{\underline{m}}t}{\hbar}}$ is the exponential time-evolution operator. The TDMs can be taken out of the integral under the FC approximation. The integrand of *Eq. 6* is defined as the cross-correlation function $\chi_k(t)$, where the index k refers to its dependence on the excited oscillator of the final state and has the following expression:

$$\chi_k(t) = \sqrt{\frac{\bar{\Gamma}_k}{2}}\left[-\frac{1}{\sqrt{2}}\sum_{l=1}^{N} J_{kl}\left(\frac{\partial \chi_0(t)}{\partial v_l}\right) + K_k\chi_0(t)\right] (Eq.7)$$

The derivative $\partial\chi_0(t)/\partial v_k$ can be easily calculated by direct differentiation:

$$\frac{\partial \chi_0(t)}{\partial v_l} = \chi_0\left[\sum_{\lambda=1}^{N}\{D^{-1}\}_{l\lambda}\, v_\lambda + \sum_{\lambda=1}^{N}\{D^{-1}\}_{l\lambda}\, v_\lambda^{*}\right]\ (Eq.8)$$

, where $\chi_0(t)$ is the autocorrelation function for the one-photon vibronic spectroscopy under the Franck-Condon approximation (TD),[75] the auxiliary vector $\boldsymbol{v}$, is defined as $\boldsymbol{J}^T\bar{\boldsymbol{\Gamma}}\boldsymbol{K}$ and the matrix D, is defined as $\mathbf{D} = \bar{\bar{\mathbf{d}}} + \mathbf{J}^{\mathbf{T}}\bar{\mathbf{d}}\,\mathbf{J}$ (see the implementation article for more details on $\boldsymbol{v}$ and **D**).[73]

For the FCHT approximation, the first-order terms from the Taylor expansion in *Eq. 2* have to be inserted in *Eq. 6*. Then, the following additional terms have to be calculated:

$$\chi_{FCHT,1}^{(j)}(t) = \langle \bar{\mathbf{1}}_k |\, e^{-\frac{i\hat{H}_{\underline{m}}t}{\hbar}}\bar{\bar{Q}}_j\, |\bar{\mathbf{0}}\rangle\ (Eq.9)$$

$$\chi_{FCHT,2}^{(j)}(t) = \langle \bar{\mathbf{1}}_k |\, \bar{\bar{Q}}_j e^{-\frac{i\hat{H}_{\underline{m}}t}{\hbar}}\, |\bar{\mathbf{0}}\rangle\ (Eq.10)$$

$$\chi_{HT}^{(j,l)}(t) = \langle \bar{\mathbf{1}}_k |\, \bar{\bar{Q}}_j e^{-\frac{i\hat{H}_{\underline{m}}t}{\hbar}}\bar{\bar{Q}}_l\, |\bar{\mathbf{0}}\rangle\ (Eq.11)$$

The derivation of their analytic formulations follows a similar approach to the FC approximation above and will not be detailed here. The reader is encouraged to read the relevant section of the implementation article.[73]

3. Excited Singlet State of Lumiflavin

Table S4 Values and statistical analysis pertaining to the main excitation energies of lumiflavin for the different DFT functionals. The terms in the table include the **$S_0 \rightarrow S_1$** and **$S_0 \rightarrow S_2$** experimental absorption and computed excitation values (in nm), corresponding deviations (**σ_{S1}, σ_{S2}**), absolute mean deviation (**μ_σ**), and separate (**δ_{S1}, δ_{S2}**) and averaged (**μ_δ**) percent errors. For comparison, the full FMN values calculated at the B3LYP/def2-TZVP level are provided. Oscillator strengths (ƒ) for the two excitations are given in the last two columns, and for the LOTs that reproduce the experimental intensities, are underlined.

LOT	$S_0 \rightarrow S_1$	$S_0 \rightarrow S_2$	σ_{S1}	σ_{S2}	μ_σ	δ_{S1} (%)	δ_{S2} (%)	μ_δ (%)	$ƒ(S_{0\rightarrow1})$	$ƒ(S_{0\rightarrow2})$
**Experimental Abs.**	**445**	**372**								
**FMN B3LYP/def2-TZVP 2'[76]**	433	364	-12	-8	10	2.1	2.8	2.4	0.089	0.243
**FMN B3LYP/def2-TZVP 2''[76]**	431	366	-15	-6	10	1.6	3.4	2.5	0.167	0.353
APFD/cc-pVDZ	413	333	-32	-39	35	37.4	29.0	33.2	0.251	0.232
B1B95/cc-pVDZ	405	326	-40	-46	43	3.8	9.5	6.6	0.269	0.229
B3LYP/cc-pVDZ	423	341	-22	-31	27	9.1	5.2	7.1	0.235	0.224
B3LYP/aug-cc-pVDZ	433	354	-12	-18	15	5.1	2.8	4.0	0.234	0.261
B3LYP/cc-pVTZ	423	345	-22	-27	25	7.9	5.3	6.6	0.237	0.250
B3LYP/aug-cc-pVTZ	427	350	-18	-22	20	6.3	4.1	5.2	0.238	0.263
B3LYP/cc-pVQZ	425	347	-20	-25	22	7.1	4.8	5.9	0.237	0.258
B3P86/cc-pVDZ	421	339	-24	-33	28	24.2	19.1	21.6	0.234	0.229
B3PW91/cc-pVDZ	421	339	-24	-33	28	12.9	9.0	11.0	0.235	0.228
B98/cc-pVDZ	418	338	-27	-34	31	1.0	6.7	3.9	0.244	0.229
BHHLYP/cc-pVDZ	357	294	-88	-79	83	5.5	0.6	3.1	0.410	0.215
BHHLYP/aug-cc-pVDZ	366	305	-79	-67	73	4.0	8.6	6.3	0.423	0.229
BLYP/cc-pVDZ	494	389	49	17	33	1.9	6.8	4.3	0.147	0.165
BMK/cc-pVDZ	379	304	-66	-68	67	18.1	15.0	16.6	0.334	0.220
BMK/aug-cc-pVDZ	387	315	-58	-57	58	22.2	17.5	19.9	0.345	0.242
BP86/cc-pVDZ	492	387	47	15	31	11.3	7.7	9.5	0.148	0.170
BPBE/cc-pVDZ	490	386	45	14	29	2.0	8.1	5.1	0.148	0.170
CAM-B3LYP/cc-pVDZ	374	300	-71	-72	72	12.6	9.1	10.8	0.375	0.203
HCTH/407/cc-pVDZ	478	379	33	7	20	22.1	21.5	21.8	0.153	0.185
HISSbPBE/cc-pVDZ	377	310	-68	-62	65	3.5	9.2	6.3	0.336	0.238
HSEH1PBE/cc-pVDZ	413	334	-32	-38	35	24.3	18.8	21.5	0.249	0.233
LC-OPBE/cc-pVDZ	333	262	-112	-110	111	4.4	9.9	7.1	0.514	0.137
LC-wHPBE/cc-pVDZ	345	271	-100	-101	101	26.1	21.6	23.8	0.473	0.141
LSDA/cc-pVDZ	484	380	39	8	24	9.9	9.0	9.5	0.149	0.173
LSDA/aug-cc-pVDZ	495	391	50	19	35	21.7	18.8	20.2	0.145	0.214
M05-2X/cc-pVDZ	366	295	-79	-77	78	1.1	2.7	0.8	0.384	0.209
M05-2X/aug-cc-pVDZ	375	306	-70	-66	68	3.6	0.9	1.3	0.399	0.225
M06/cc-pVDZ	408	338	-37	-34	35	9.9	9.0	9.5	0.276	0.235
M06/aug-cc-pVDZ	418	350	-27	-22	24	4.5	1.6	3.1	0.277	0.264
M06-HF/cc-pVDZ	408	338	-37	-34	35	6.3	6.4	6.4	0.276	0.235
M06L/cc-pVDZ	449	359	4	-13	8	18.2	14.0	16.1	0.183	0.219
M06L/aug-cc-pVDZ	457	368	12	-4	8	4.6	0.6	2.6	0.187	0.248
M11L/cc-pVDZ	438	356	-7	-16	12	2.3	7.4	4.9	0.191	0.245
MN15/cc-pVDZ	390	315	-55	-57	56	2.2	6.8	4.5	0.335	0.213
MN15/aug-cc-pVDZ	400	327	-45	-45	45	1.0	4.5	1.7	0.347	0.236
mPW1PW91/cc-pVDZ	408	330	-37	-42	40	14.0	9.9	11.9	0.261	0.234
mPWLYP/cc-pVDZ	494	389	49	17	33	4.8	0.6	2.7	0.147	0.165
O3LYP/cc-pVDZ	442	355	-3	-17	10	12.8	9.1	10.9	0.197	0.216
OLYP/cc-pVDZ	483	381	38	9	24	18.2	14.0	16.1	0.152	0.178
OPBE/cc-pVDZ	480	378	35	6	20	11.4	7.7	9.6	0.153	0.183
PBE1PBE/cc-pVDZ	408	330	-37	-42	40	9.7	5.7	7.7	0.261	0.233
PW6B95D3/cc-pVDZ	405	327	-40	-45	43	10.2	6.4	8.3	0.269	0.227
revTPSSh/aug-cc-pVDZ	404	330	-41	-42	41	10.1	6.4	8.3	0.227	0.273
revTPSS/aug-cc-pVDZ	485	385	40	13	26	13.9	9.8	11.9	0.157	0.217
SOGGA11/cc-pVDZ	481	381	36	9	22	20.0	18.0	19.0	0.152	0.168

SOGGA11x/cc-pVDZ	378	308	-67	-64	66	1.8	7.5	4.7	0.347	0.233
tHCTHhyb/cc-pVDZ	436	351	-9	-21	15	4.5	1.7	3.1	0.210	0.220
TPSSh/cc-pVDZ	442	353	-3	-19	11	12.6	10.3	11.4	0.197	0.210
TPSS/cc-pVDZ	477	376	32	4	18	3.4	8.2	5.8	0.157	0.178
TPSS/aug-cc-pVDZ	487	387	42	15	29	4.9	10.2	7.5	0.155	0.214
VSXC/cc-pVDZ	466	368	21	-4	12	5.1	2.8	4.0	0.177	0.196
VSXC/aug-cc-pVDZ	477	380	32	8	20	9.1	5.2	7.1	0.174	0.232
wB97XD/cc-pVDZ	375	299	-70	-73	71	7.9	5.3	6.6	0.372	0.208
X3LYP/cc-pVDZ	418	338	-27	-34	30	6.3	4.1	5.2	0.245	0.226

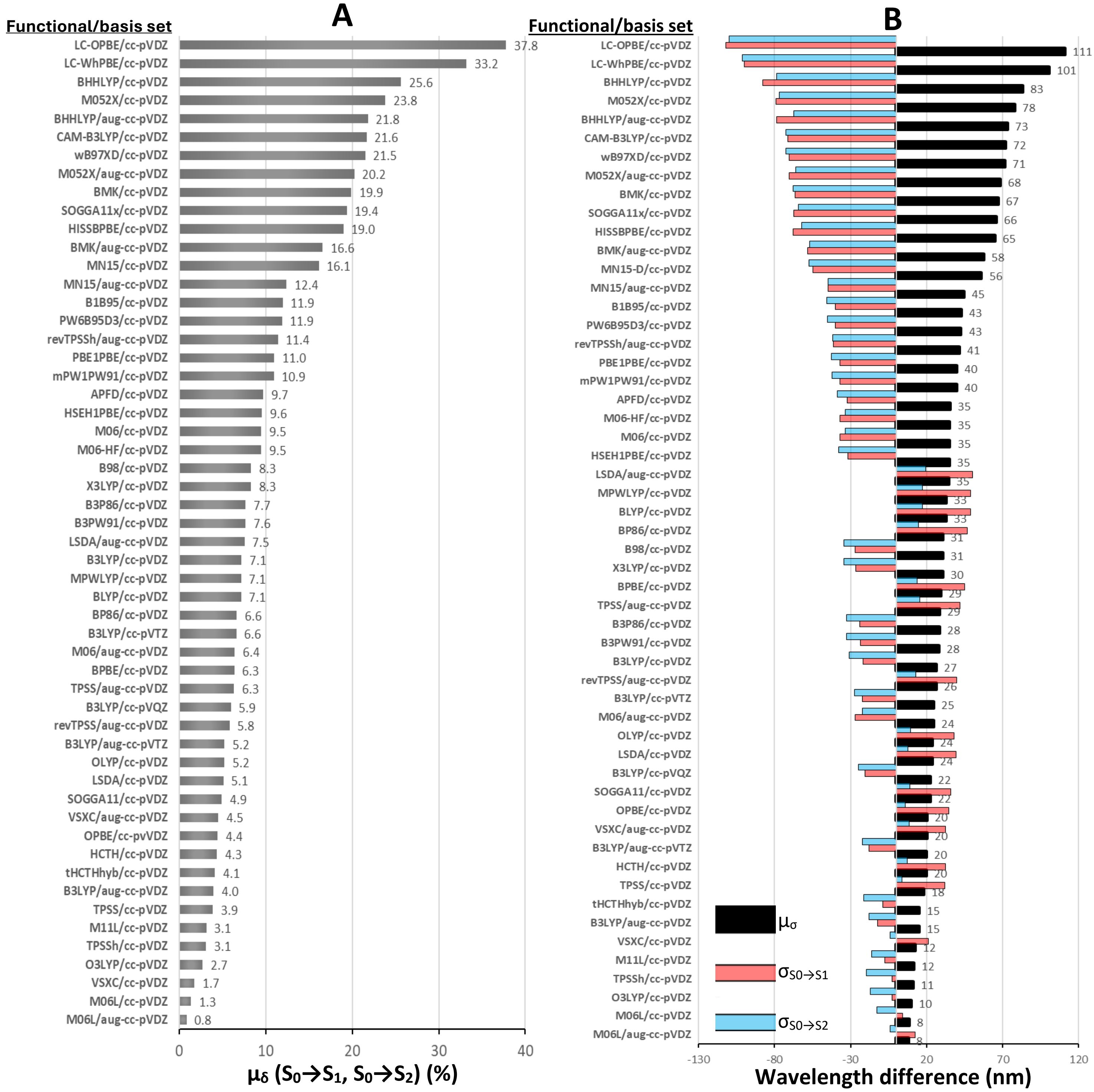

Figure S1 A: Average Percent error (μ_δ, %) of the two major excitations. **B**: Deviation values (**σ**) for the $S_0 \rightarrow S_1$ (light red), $S_0 \rightarrow S_2$ (light blue) and absolute mean deviations (μ_σ, black) of different DFT functionals with the experimental absorption values. All values are given in nm and are based on the data from **Table S4**. Functionals are sorted from top to bottom from higher μ_σ/μ_δ values (worse) to lower (better agreement with experiment).

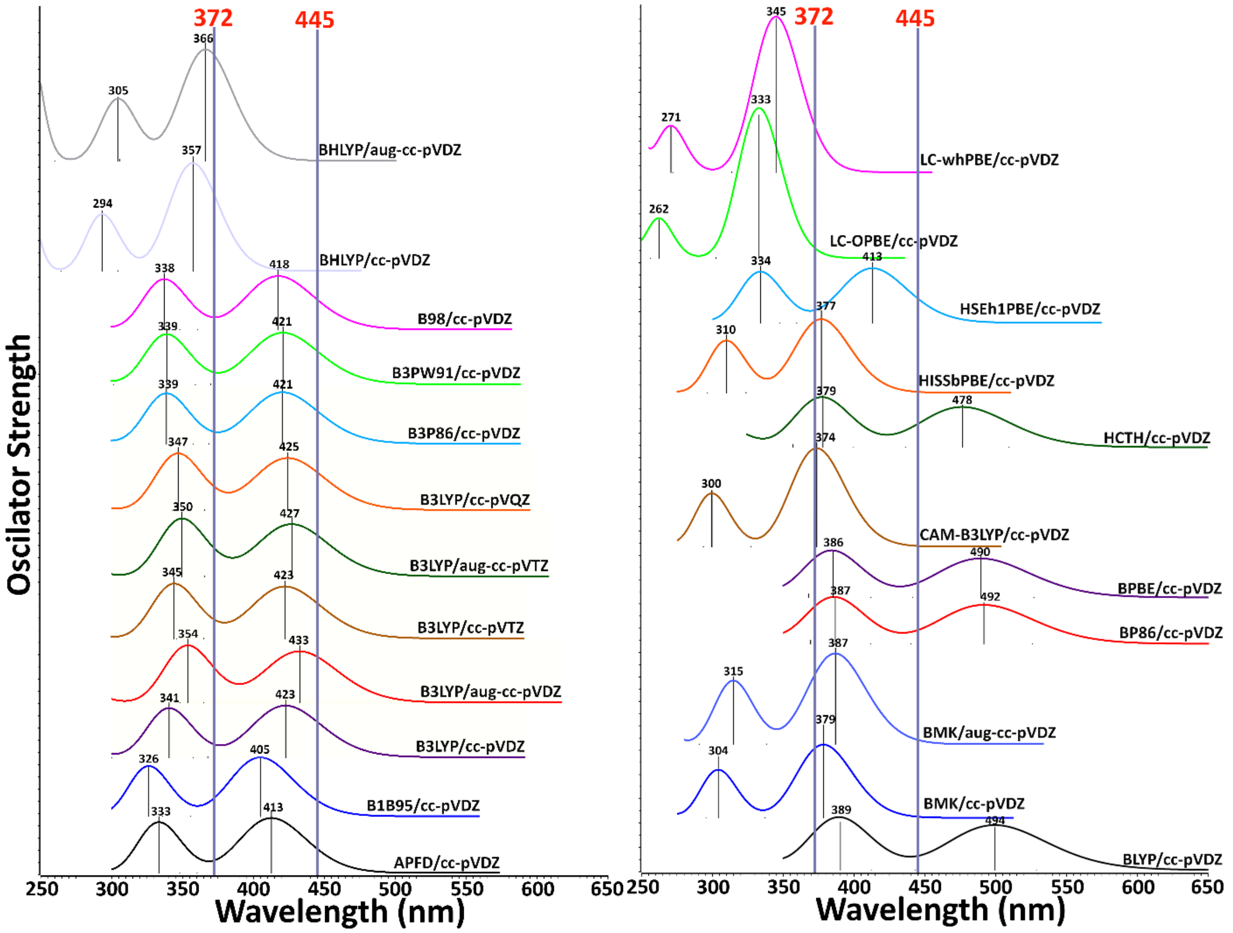

Figure S2 Simulated unnormalized UV-Vis spectra of all DFT functionals at their corresponding level of theory based on their vertical excitation profiles, plotted with a Half-Width at Half-Height value of 0.2 eV (HWHH). The major excitations $S_{0\rightarrow 1}$ and $S_{0\rightarrow 2}$ have been labelled, and the respective experimental λ_{max} values are shown with blue bars and labelled in red.

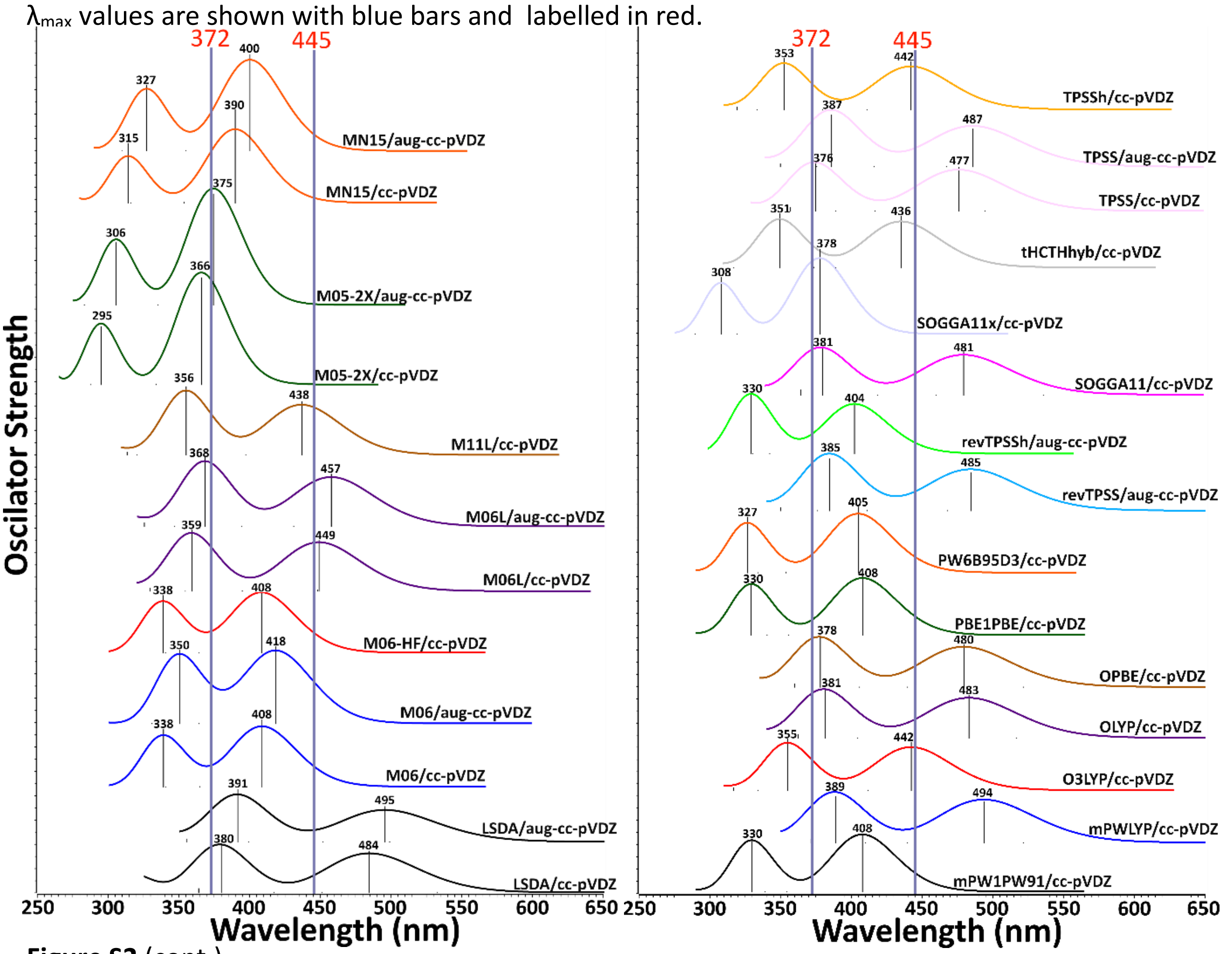

Figure S2 (cont.)

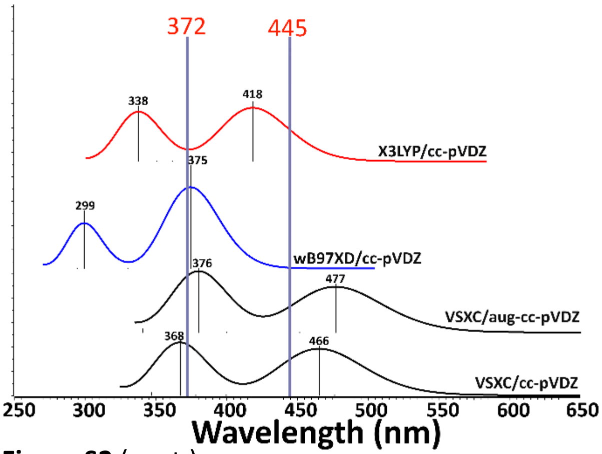

Figure S2 (cont.)

Table S5 Typical vibrations showing the displacement vector arrows in the fingerprint region of lumiflavin, based on the B3LYP/aug-cc-pVDZ S_1 calculation. Vibrations that are underlined signify important transitions in the vibronic spectrum of lumiflavin.

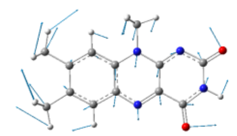	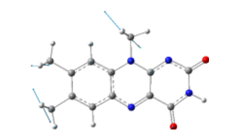	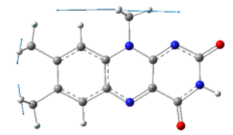	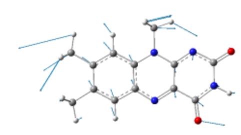	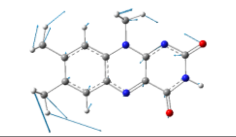
ν_7 (in-plane)	ν_8 (out-of-plane)	ν_{10}	ν_{12}	ν_{13}
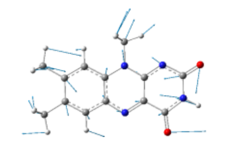	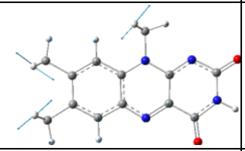	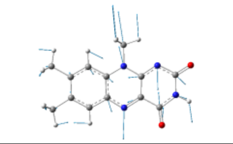	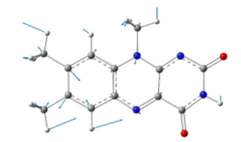	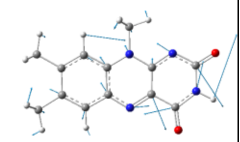
ν_{18}	ν_{19}	ν_{23}	ν_{48}	ν_{51}
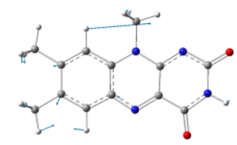	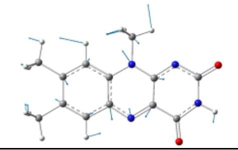	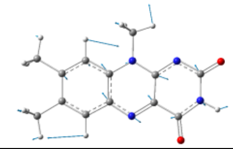	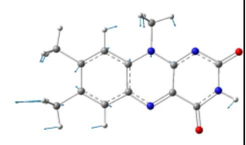	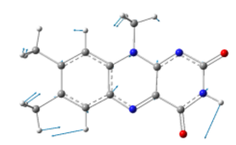
ν_{52}	ν_{53}	ν_{54}	ν_{56}	ν_{57}
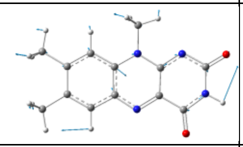	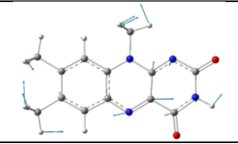	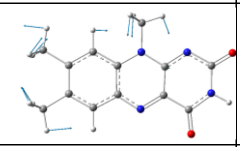	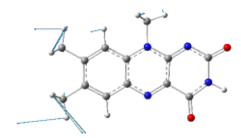	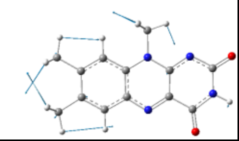
ν_{61}	ν_{64}	ν_{65}	ν_{66}	ν_{67}
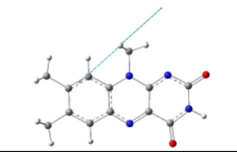	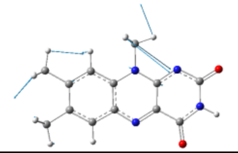	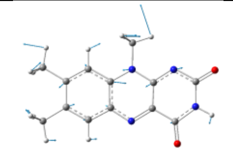	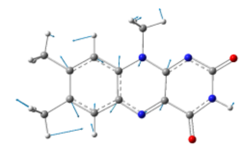	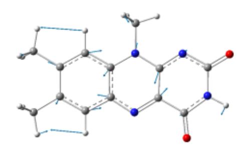
ν_{68}	ν_{69}	ν_{70}	ν_{71}	ν_{72}
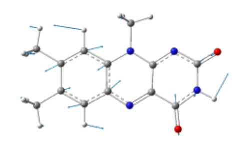	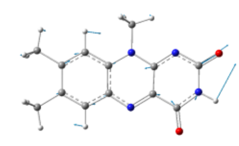	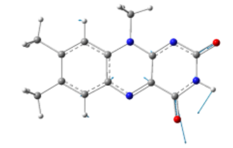		
ν_{73}	ν_{74}	ν_{75}		

Table S6 Data utilized for the determination of the 0-0 transitions and vibronic spectra for each DFT functional. The experimental crossing point and the 0-0 transition of full FMN calculated at the B3LYP/def2-TZVP level are included in the top three rows and are taken from our previous work.[76] Energies and zero-point corrections are given in Hartrees, and the 0-0 energy in nanometers.

LOT	Energy S_0*	ZPE S_0	Energy S_1*	ZPE S_1	0-0	E^{adia}
Experimental 0-0 Transition[76]	-	-	-	-	492#	-
FMN B3LYP/def2-TZVP 2'[76]					494	
FMN B3LYP/def2-TZVP 2''[76]					494	
APFD/cc-pVDZ	-871.271420	0.241	-871.174824	0.238	472	0.09960
B1B95/cc-pVDZ	-871.666059	0.242	-871.567857	0.239	464	0.10148
B3LYP/cc-pVDZ	-872.017078	0.239	-871.923261	0.236	486	0.09711
B3LYP/aug-cc-pVDZ	-872.079136	0.239	-871.987400	0.236	497	0.09492
B3LYP/cc-pVTZ	-872.272249	0.240	-872.178452	0.236	486	0.09707
B3LYP/aug-cc-pVTZ	-872.285024	0.239	-872.192174	0.236	491	0.09603
B3LYP/cc-pVQZ	-872.340673	0.239	-872.247309	0.236	488	0.09663
B3P86/cc-pVDZ	-874.416094	0.240	-874.321610	0.237	482	0.09770
B3PW91/cc-pVDZ	-871.697556	0.240	-871.603156	0.237	483	0.09762
B98/cc-pVDZ	-871.679309	0.239	-871.584126	0.236	479	0.09828
BHHLYP/cc-pVDZ	-871.492768	0.249	-871.381564	0.246	410	0.11398
BHHLYP/aug-cc-pVDZ	-871.545356	0.248	-871.436899	0.246	420	0.11116
BLYP/cc-pVDZ	-871.742555	0.231	-871.66237	0.229	568	0.08269
BMK/cc-pVDZ	-871.487033	0.241	-871.381026	0.239	430	0.10820
BMK/aug-cc-pVDZ	-871.541610	0.242	-871.438602	0.239	442	0.05016
BP86/cc-pVDZ	-872.040695	0.232	-871.960163	0.229	566	0.08324
BPBE/cc-pVDZ	-871.653635	0.233	-871.572903	0.230	564	0.08357
CAM-B3LYP/cc-pVDZ	-871.551393	0.242	-871.445178	0.239	429	0.10909
HCTH/407/cc-pVDZ	-871.884878	0.238	-871.801739	0.235	548	0.08595
HISSbPBE/cc-pVDZ	-871.095924	0.248	-870.990262	0.245	431	0.10876
HSEH1PBE/cc-pVDZ	-871.082794	0.241	-870.986415	0.238	473	0.09960
LC-OPBE/cc-pVDZ	-869.539422	0.250	-869.421532	0.248	387	0.12022
LC-wHPBE/cc-pVDZ	-871.386736	0.245	-871.273469	0.242	402	0.11579
LSDA/cc-pVDZ	-867.216455	0.234	-867.134654	0.231	557	0.08497
M05-2X/aug-cc-pVDZ	-871.940808	0.244	-871.834768	0.241	430	0.10880
M06/cc-pVDZ	-871.368786	0.238	-871.253872	0.234	397	0.10056
M06/aug-cc-pVDZ	-871.425640	0.238	-871.330703	0.235	480	0.09804
M06-HF/cc-pVDZ	-871.366933	0.238	-871.271456	0.235	477	0.09871
M06L/cc-pVDZ	-871.884768	0.240	-871.804952	0.237	571	0.08222
M06L/aug-cc-pVDZ	-871.923788	0.239	-871.837508	0.236	528	0.08988
M11L/cc-pVDZ	-871.759862	0.239	-871.669541	0.236	504	0.09398
MN15/cc-pVDZ	-870.944852	0.241	-870.843034	0.238	448	0.10472
MN15/aug-cc-pVDZ	-871.012785	0.241	-870.913541	0.238	459	0.10170
mPW1PW91/cc-pVDZ	-871.828538	0.242	-871.730877	0.239	467	0.10079
mPWLYP/cc-pVDZ	-871.698804	0.231	-871.618594	0.229	568	0.08270
O3LYP/cc-pVDZ	-871.732489	0.239	-871.642981	0.236	509	0.09295
OLYP/cc-pVDZ	-871.89935	0.237	-871.817218	0.234	555	0.08486
OPBE/cc-pVDZ	-871.807140	0.238	-871.724426	0.235	551	0.08567
PBE1PBE/cc-pVDZ	-871.019225	0.241	-870.921755	0.238	467	0.10074
PW6B95D3/cc-pVDZ	-873.080130	0.242	-872.981951	0.239	464	0.10140
revTPSSh/aug-cc-pVDZ	-889.233821	0.260	-889.134910	0.256	461	0.10223
revTPSS/aug-cc-pVDZ	-871.961827	0.235	-871.880904	0.231	563	0.08454
SOGGA11/cc-pVDZ	-872.045978	0.236	-871.964177	0.232	557	0.08534
SOGGA11x/cc-pVDZ	-871.764897	0.245	-871.659448	0.242	432	0.10835
tHCTHhyb/cc-pVDZ	-871.861567	0.237	-871.770646	0.234	501	0.09425

LOT	Energy S_0*	ZPE S_0	Energy S_1*	ZPE S_1	0-0	E^{adia}
TPSSh/cc-pVDZ	-872.067499	0.238	-871.978242	0.235	510	0.09272
TPSS/aug-cc-pVDZ	-872.228284	0.235	-872.147701	0.231	565	0.08417
VSXC/cc-pVDZ	-872.343105	0.237	-872.269028	0.235	615	0.07632
VSXC/aug-cc-pVDZ	-872.402597	0.237	-872.320136	0.233	553	0.08594
wB97XD/cc-pVDZ	-871.671274	0.242	-871.565358	0.239	430	0.10877
X3LYP/cc-pVDZ	-871.628185	0.240	-871.533134	0.237	479	0.09821

*: The reported energies are ZPE-corrected.
#: The value reported is the crossing point between the absorption and fluorescence bands of FMN in water.

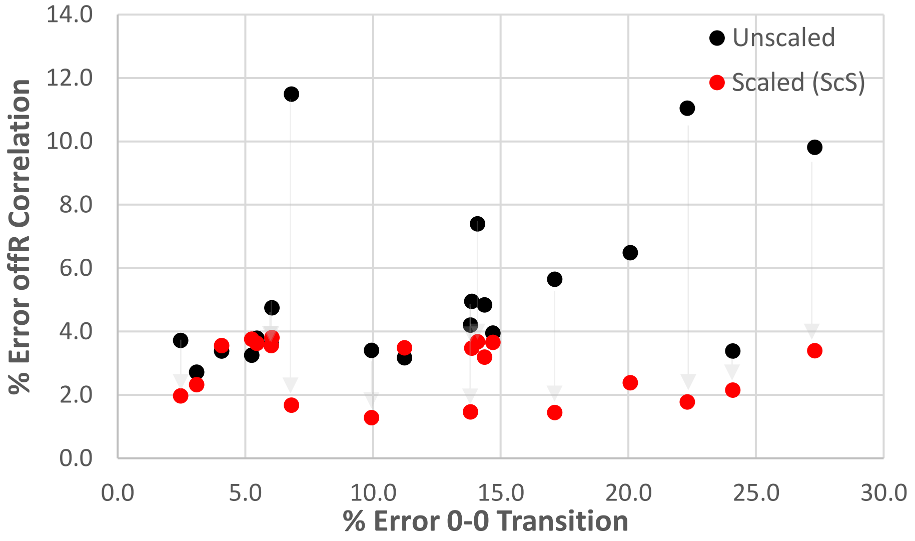

Figure S3 Relationship between the off-resonance correlation percent error and the 0-0 transition percent error for the 19 DFT functionals that the specific scaling factor was applied **(Sc_S)**. Two different sets are provided, one with the unscaled correlation (black points) and the other with the said **Sc_S** factor applied (red points). Faint grey arrows connect the corresponding set points demonstrating the improvement of the offR correlation after the application of the **Sc_S** scaling factor.

Table S7. Shifts of vibronic transitions of all the DFT functionals (in nm) with respect to the 0-0 transition energy of lumiflavin computed for the $S_0 \rightarrow S_1$ excitation. The vibronic transitions have been grouped according to their energy.

LOT	$\|0\rangle\rightarrow\|0\rangle$	$\|0\rangle\rightarrow\|6^1\rangle$<br>$\|0\rangle\rightarrow\|7^1\rangle$<br>$\|0\rangle\rightarrow\|8^1\rangle$	$\|0\rangle\rightarrow\|10^1\rangle$<br>$\|0\rangle\rightarrow\|12^1\rangle$<br>$\|0\rangle\rightarrow\|13^1\rangle$	$\|0\rangle\rightarrow\|7^2\rangle$<br>$\|0\rangle\rightarrow\|8^2\rangle$	$\|0\rangle\rightarrow\|18^1\rangle$<br>$\|0\rangle\rightarrow\|19^1\rangle$	$\|0\rangle\rightarrow\|10^17^1\rangle$<br>$\|0\rangle\rightarrow\|12^17^1\rangle$<br>$\|0\rangle\rightarrow\|12^18^1\rangle$<br>$\|0\rangle\rightarrow\|13^17^1\rangle$<br>$\|0\rangle\rightarrow\|13^18^1\rangle$	$\|0\rangle\rightarrow\|7^3\rangle$	$\|0\rangle\rightarrow\|23^1\rangle$	$\|0\rangle\rightarrow\|18^17^1\rangle$<br>$\|0\rangle\rightarrow\|19^18^1\rangle$	$\|0\rangle\rightarrow\|23^16^1\rangle$<br>$\|0\rangle\rightarrow\|23^17^1\rangle$<br>$\|0\rangle\rightarrow\|23^18^1\rangle$	$\|0\rangle\rightarrow\|51^1\rangle$<br>$\|0\rangle\rightarrow\|53^1\rangle$<br>$\|0\rangle\rightarrow\|66^1\rangle$<br>$\|0\rangle\rightarrow\|68^1\rangle$<br>$\|0\rangle\rightarrow\|69^1\rangle$	$\|0\rangle\rightarrow\|53^17^1\rangle$	$\|0\rangle\rightarrow\|70^1\rangle$<br>$\|0\rangle\rightarrow\|72^1\rangle$<br>$\|0\rangle\rightarrow\|73^1\rangle$	$\|0\rangle\rightarrow\|67^17^1\rangle$	$\|0\rangle\rightarrow\|72^17^1\rangle$<br>$\|0\rangle\rightarrow\|72^18^1\rangle$<br>$\|0\rangle\rightarrow\|73^17^1\rangle$
**APFD**	0	-4	-6	-7	-9			-11		-15			-32		-36
**B1B95**	0	-4		-7				-11		-14			-32		-35
**B3LYP(dz)**	0	-4	-6	-8	-9			-12		-16			-34		-37
**B3LYP(aug,dz)**	0	-4	-7	-8	-10	-11		-13	-14	-16			-35		
**B3LYP(tz)**	0	-4	-6	-8	-9			-12		-16					
**B3LYP(aug,tz)**	0	-4	-7	-8	-10			-12		-16					
**B3LYP(qz)**	0	-4	-7	-8	-10			-12		-16					
**B3P86**	0	-4	-6	-8				-12		-16			-34		-37
**B3PW91**	0	-4	-6	-8				-12		-16			-34		-37
**B98**	0	-4	-6	-7				-12		-15			-33		-36
**BHHLYP**	0	-3		-6				-9		-12	-24		-26		-27
**BHHLYP(aug)**	0	-3	-5	-6				-9		-12	-23			-28	
**BLYP**	0	-5		-10			-15	-16		-21			-44		-48
**BMK(aug)**	0	-16	-25	-31				-46		-60	-110		-125		
**BP86**	0	-5		-10			-15	-16		-21	-37	-42	-44		-49
**BPBE**	0	-5	-8	-10			-15	-16		-20	-37	-42	-44		-49
**CAM-B3LYP**	0	-3		-6				-9		-12	-26		-27	-28	-30
**HCTH**	0	-5	-8	-10			-14	-15		-20			-43		-47
**HISS**	0	-3	-5	-6				-10		-13			-28		-31
**HSEH**	0	-4	-6	-7				-11		-15			-33		-36
**LC-OPBE**	0	-3						-8		-10	-22		-24		-27
**LC-wHPBE**	0	-3						-8		-11	-23.5		-26		
**LSDA**	0	-5		-10	-12		-15	-15	-17	-20			-44		-49
**M06(aug)**	0	-4	-6	-7	-9			-12		-15	-27		-33		
**M06-HF**	0	-4	-6	-7				-11		-15			-33		-36
**M06L(aug)**	0	-5	-8	-9	-11	-13		-14		-18			-40		-44
**M11L-D**	0	-4	-7	-8	-10	-11		-13	-14	-17			-37		-41
**M052X(aug)**	0	-3	-5	-6				-9		-12	-24		-28		
**MN15(aug)**	0	-3	-6	-7				-10		-14	-29		-31		
**MN15**	0	-3	-5	-6				-10		-13	-27		-29		-32
**mPW1PW91**	0	-4	-6	-7				-11		-15			-32		-35
**mPWLYP**	0	-5		-10			-15	-16		-21			-44		-48
**O3LYP**	0	-4	-7	-8	-10	-11		-13		-17			-37		-41
**OLYP**	0	-5	-8	-10		-13	-15	-15		-20	-37	-41	-43		-48
**OPBE**	0	-5	-8	-10	-12	-13	-15	-15		-20	-36	-41	-43		-48
**PBE1PBE**	0	-4	-6	-7	-9			-11		-15			-32		-35
**PW6B95D3**	0	-4		-7	-9			-11		-14			-32		-35
**revTPSS(aug)**	0	-5		-10				-15		-20					
**revTPSSh(aug)**	0	-4	-6	-7	-9	-10		-11		-15			-33		-36
**SOGGA11**	0	-5		-10				-15		-20			-43		-48
**SOGGA11x**	0	-3	-5	-6				-10		-13	-26		-28		-31
**tHCTHhyb**	0	-4		-8	-10			-13		-16			-36		-39
**TPSS(aug)**	0	-5		-10				-16		-20			-37		-41
**TPSSh**	0	-4		-8			-12	-13		-17					
**VSXC(aug)**	0	-5	-8	-10	-12	-13		-15		-20			-43		
**wB97XD**	0	-3						-10		-12	-26		-27		
**X3LYP**	0	-4	-6	-8	-9			-12		-15			-33		

Table S8. Dipole Strengths of the vibronic transitions of lumiflavin (in a.u.) of all the DFT functionals computed for the $S_0 \rightarrow S_1$ excitation. The vibronic transitions have been grouped according to their energy.

LOT	$\|0\rangle\rightarrow\|0\rangle$	$\|0\rangle\rightarrow\|6^1\rangle$<br>$\|0\rangle\rightarrow\|7^1\rangle$<br>$\|0\rangle\rightarrow\|8^1\rangle$	$\|0\rangle\rightarrow\|10^1\rangle$<br>$\|0\rangle\rightarrow\|12^1\rangle$<br>$\|0\rangle\rightarrow\|13^1\rangle$	$\|0\rangle\rightarrow\|7^2\rangle$<br>$\|0\rangle\rightarrow\|8^2\rangle$	$\|0\rangle\rightarrow\|18^1\rangle$<br>$\|0\rangle\rightarrow\|19^1\rangle$	$\|0\rangle\rightarrow\|10^17^1\rangle$<br>$\|0\rangle\rightarrow\|12^17^1\rangle$<br>$\|0\rangle\rightarrow\|12^18^1\rangle$<br>$\|0\rangle\rightarrow\|13^17^1\rangle$<br>$\|0\rangle\rightarrow\|13^18^1\rangle$	$\|0\rangle\rightarrow\|7^3\rangle$	$\|0\rangle\rightarrow\|23^1\rangle$	$\|0\rangle\rightarrow\|18^17^1\rangle$<br>$\|0\rangle\rightarrow\|19^18^1\rangle$	$\|0\rangle\rightarrow\|23^16^1\rangle$<br>$\|0\rangle\rightarrow\|23^17^1\rangle$<br>$\|0\rangle\rightarrow\|23^18^1\rangle$	$\|0\rangle\rightarrow\|51^1\rangle$<br>$\|0\rangle\rightarrow\|53^1\rangle$<br>$\|0\rangle\rightarrow\|66^1\rangle$<br>$\|0\rangle\rightarrow\|68^1\rangle$<br>$\|0\rangle\rightarrow\|69^1\rangle$	$\|0\rangle\rightarrow\|53^17^1\rangle$	$\|0\rangle\rightarrow\|70^1\rangle$<br>$\|0\rangle\rightarrow\|72^1\rangle$<br>$\|0\rangle\rightarrow\|73^1\rangle$	$\|0\rangle\rightarrow\|67^17^1\rangle$	$\|0\rangle\rightarrow\|72^17^1\rangle$<br>$\|0\rangle\rightarrow\|72^18^1\rangle$<br>$\|0\rangle\rightarrow\|73^17^1\rangle$
**APFD**	0.4868	0.1911	0.0646	0.1097	0.0712			0.1377		0.0927			0.0909		0.0583
**B1B95**	0.5317	0.4002		0.1508				0.1574		0.1184			0.1041		0.0752
**B3LYP(dz)**	0.4594	0.3852	0.0524	0.1597	0.0532			0.1231		0.1030			0.0793		0.0638
**B3LYP(aug,dz)**	0.4671	0.3814	0.0648	0.1544	0.0621	0.0523		0.1430	0.0523	0.1167			0.0568		
**B3LYP(tz)**	0.4334	0.3563	0.0591	0.1450	0.0543			0.1294		0.1064					
**B3LYP(aug,tz)**	0.4475	0.3584	0.0634	0.1423	0.0587			0.1375		0.1101					
**B3LYP(qz)**	0.4419	0.3572	0.0608	0.1431	0.0572			0.1333		0.1078					
**B3P86**	0.4763	0.3689	0.0604	0.1437				0.1274		0.0987			0.0900		0.0668
**B3PW91**	0.4802	0.3738	0.0622	0.1464				0.1265		0.0986			0.0902		0.0673
**B98**	0.4842	0.3695	0.0624	0.1421				0.1341		0.1024			0.0875		0.0640
**BHHLYP**	0.7202	0.4196		0.1234				0.2654		0.1554	0.1963		0.1027	0.1130	
**BHHLYP(aug)**	0.7430	0.4355	0.0902	0.1289				0.2979		0.1755	0.1115			0.0835	
**BLYP**	0.2609	0.2608		0.1297			0.0428	0.0461		0.0458			0.0399		0.0380
**BMK(aug)**	0.6332	0.4156	0.0800	0.1437				0.2455		0.1622	0.0810		0.0956		
**BP86**	0.2971	0.2729		0.1256			0.0387	0.0528		0.0482	0.0428	0.0392	0.0476		0.0418
**BPBE**	0.3023	0.2769	0.0381	0.1272			0.0391	0.0529		0.0482	0.0436	0.0398	0.0486		0.0425
**CAM-B3LYP**	0.7219	0.4280		0.1245				0.2584		0.1530	0.1905		0.1427	0.1117	0.0809
**HCTH**	0.3123	0.2946	0.0380	0.1377			0.0425	0.0550		0.0514			0.0527		0.0477
**HISS**	0.6117	0.4383	0.0730	0.1586				0.1909		0.1372			0.0977		0.0671
**HSEH**	0.5003	0.3547	0.0610	0.1262				0.1351		0.0960			0.0943		0.0642
**LC-OPBE**	0.7695	0.3452						0.3069		0.1374	0.1424		0.1869		0.1064
**LC-wHPBE**	0.6599	0.2996						0.2592		0.1172	0.1197		0.1289		
**LSDA**	0.3217	0.3021		0.1406	0.0409		0.0433	0.0587	0.0403	0.0545			0.0510		0.0459
**M06(aug)**	0.5221	0.3647	0.0872	0.1264	0.0617			0.1832		0.1282	0.0619		0.0688		
**M06-HF**	0.5209	0.3877	0.0611	0.1423				0.1660		0.1235			0.0853		0.0608
**M06L(aug)**	0.3878	0.3211	0.0566	0.1339	0.0462	0.0455		0.0966		0.0797			0.0576		0.0458
**M11L-D**	0.3375	0.3007	0.0542	0.1348	0.0506	0.0493		0.0809	0.0454	0.0718			0.0664		0.0571
**M052X(aug)**	0.7169	0.3971	0.0941	0.1106				0.2899		0.1614	0.1012		0.0974		
**MN15(aug)**	0.6980	0.4413	0.0952	0.1390				0.2580		0.1636	0.0727		0.1099		
**MN15**	0.6725	0.4144	0.0745	0.1293				0.2243		0.1386	0.0853		0.1336		0.0789
**mPW1PW91**	0.5226	0.3771	0.0677	0.1367				0.1482		0.1072			0.0999		0.0691
**mPWLYP**	0.2597	0.2604		0.1299			0.0430	0.0460		0.0458			0.0406		0.0388
**O3LYP**	0.4014	0.3332	0.0534	0.1391	0.0464	0.0451		0.0910		0.0755			0.0729		0.0581
**OLYP**	0.3253	0.2922	0.0431	0.1318		0.0392	0.0397	0.0544		0.0486	0.0432	0.0386	0.0538		0.0462
**OPBE**	0.3222	0.2954	0.0468	0.1362	0.0382	0.0435	0.0421	0.0570		0.0519	0.0417	0.0381	0.0542		0.0477
**PBE1PBE**	0.4807	0.3975	0.0539	0.1625	0.0550			0.1405		0.1158			0.0947		0.0753
**PW6B95D3**	0.5348	0.1995		0.0852	0.0560			0.1593		0.0894			0.1031		0.0553
**revTPSS(aug)**	0.2180	0.2209		0.1116				0.0527		0.0533					
**revTPSSh(aug)**	0.3890	0.3248	0.0604	0.1359	0.0503	0.0486		0.1129		0.0944			0.0582		0.0466
**SOGGA11**	0.2932	0.2679		0.1192				0.0490		0.0439			0.0472		0.0413
**SOGGA11x**	0.6343	0.3824	0.0745	0.1168				0.2213		0.1338	0.0684		0.1165		0.0673
**tHCTHhyb**	0.3927	0.3488		0.1536	0.0482			0.0970		0.0859			0.0726		0.0620
**TPSS(aug)**	0.2489	0.2436		0.1190				0.0596		0.0582					
**TPSSh**	0.3294	0.3169		0.1510			0.0475	0.0788		0.0755			0.0618		0.0572
**VSXC(aug)**	0.3630	0.2580	0.0999	0.0908	0.0466	0.0717		0.0906		0.0640			0.0569		
**wB97XD**	0.7438	0.2117						0.2813		0.1222	0.0859		0.1679		
**X3LYP**	0.5146	0.1958	0.0645	0.0777	0.0550			0.1375		0.0767			0.0890		

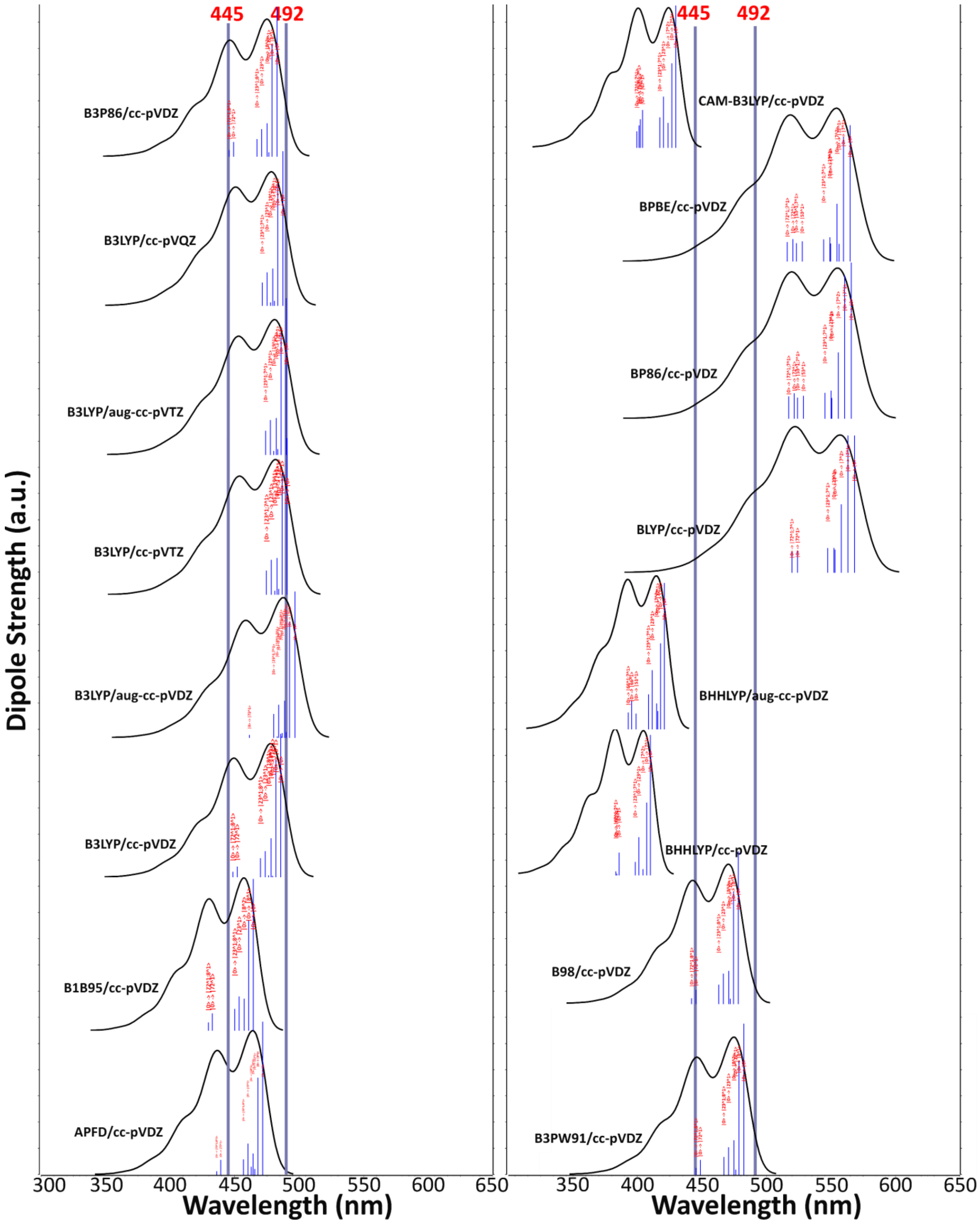

Figure S4 Unnormalized vibronic spectra of all DFT functionals at their corresponding level of theory based on the OPA calculation of the $S_0 \rightarrow S_1$ excitation, plotted with gaussian broadening and a Half-Width at Half-Maximum value of 400 cm^{-1} (HWHM). The major vibronic excitations have been labelled in red and are shown with blue sticks, and the experimental λ_{max} (445 nm) and 0-0 transition values (492 nm)[76] are indicated with blue bars.

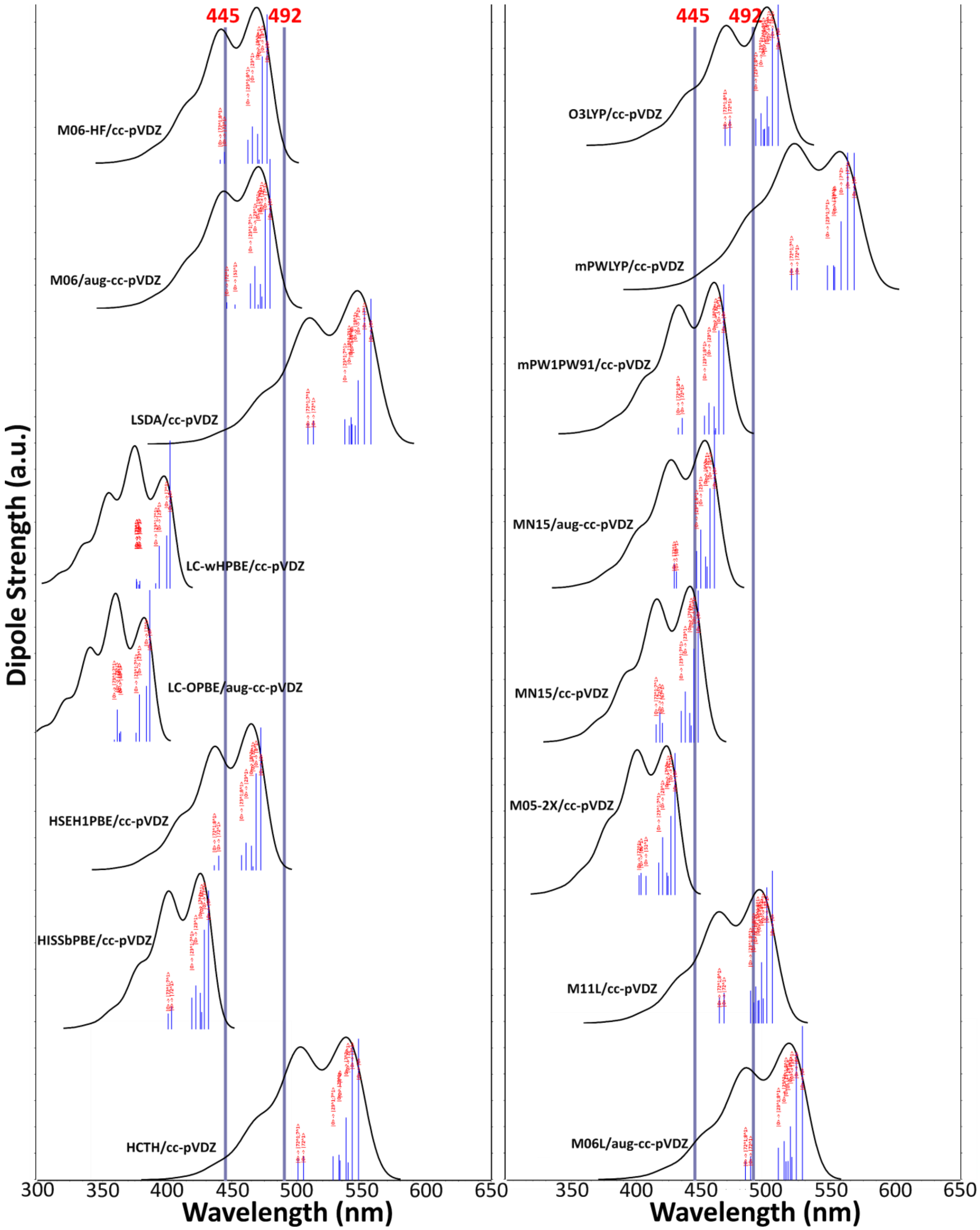

Figure S4 cont.

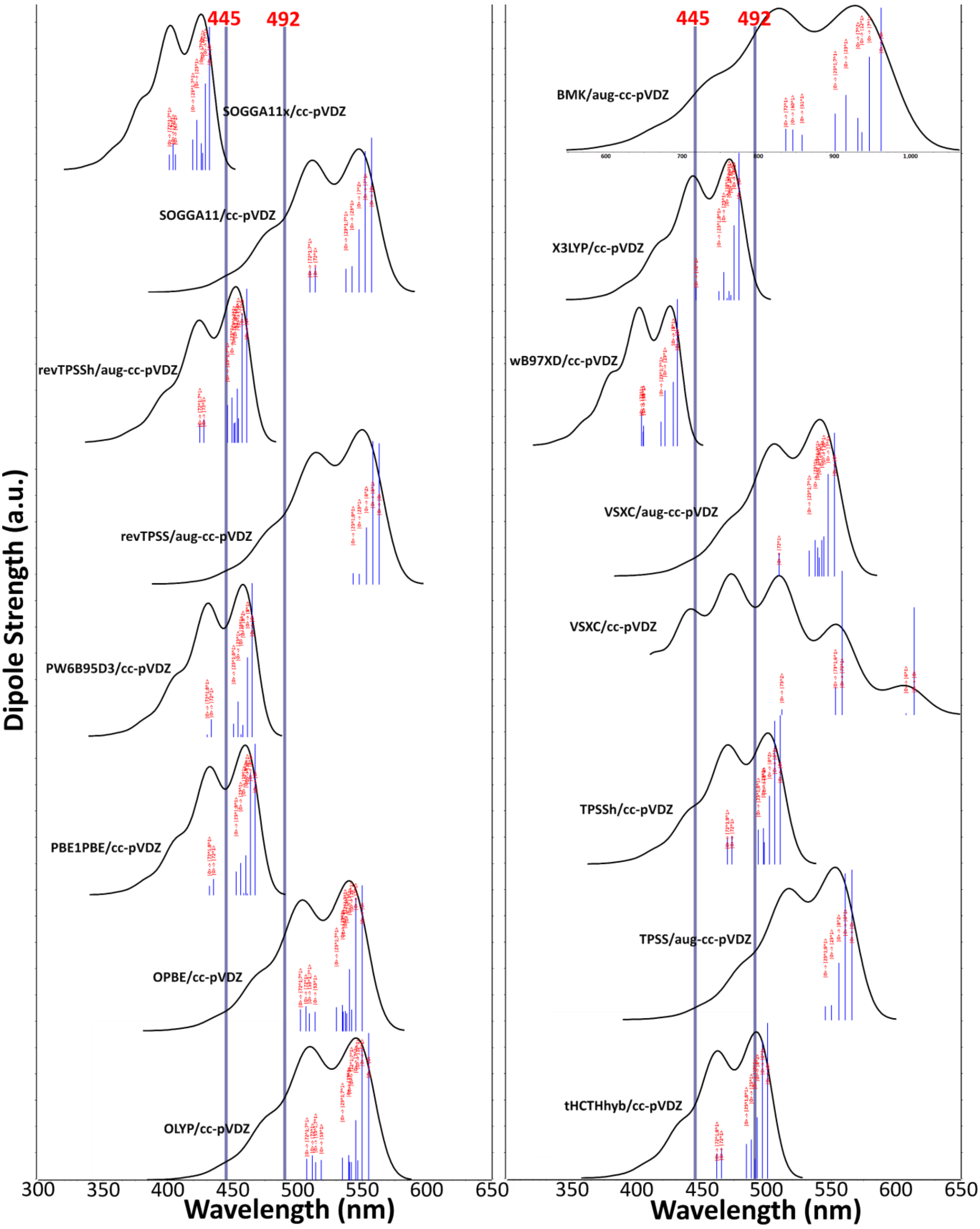

Figure S4 cont.

Table S9 Analysis of Excitations from the first excited state of lumiflavin (S_1) to higher singlet states r_n. Transition dipole moments (**TDM x, y, z**) are included, energies **EE** in eV and oscillator strengths **Osc.** as well as the difference **Δr** of the excitation from the wavelength of experimental Raman pump. Predominant excitations at each LOT, after optimization, are highlighted in bold. If after optimization a different state was obtained due to state crossing, the excitation is underlined (e.g. for B3LYP/aug-cc-pVDZ, optimization of r_7 yielded the r_6 state, see also subsequent **Table S10**). State numbers are signified as "roots" (i.e. root 5 = r_5 = S_5, root 7 = r_7 = S_7).

LOT	Excitation	TDM x	TDM y	TDM z	EE (eV)	Osc.	Δr (nm)
APFD/cc-pVDZ	**$S_1 \rightarrow r_5$**	-0.075	0.006	0.019	1.44	0.00021	4
	$\underline{S_1 \rightarrow r_6}$	-0.357	-0.544	-0.032	1.55	0.01615	69
B1B95/cc-pVDZ	$S_1 \rightarrow r_5$	0.032	-0.004	0.007	1.56	0.00004	72
	$\underline{S_1 \rightarrow r_6}$	-0.171	-0.620	-0.018	1.64	0.01665	119
	**$S_1 \rightarrow r_7$**	-3.574	1.299	-0.279	1.89	0.67387	259
B3LYP/cc-pVDZ	$\underline{S_1 \rightarrow r_6}$	0.009	-0.699	0.000	1.47	0.01763	26
	**$S_1 \rightarrow r_7$**	-3.518	-1.642	0.000	1.76	0.65090	187
B3LYP/aug-cc-pVDZ	$S_1 \rightarrow r_5$	0.001	0.000	0.008	1.56	0.00000	71
	**$S_1 \rightarrow r_6$**	-0.943	-0.292	0.000	1.61	0.03837	100
	$\underline{S_1 \rightarrow r_7}$	3.384	-1.798	0.000	1.76	0.63213	184
B3LYP/cc-pVTZ	$\underline{S_1 \rightarrow r_5}$	0.001	0.000	0.011	1.52	0.00000	49
	$\underline{S_1 \rightarrow r_6}$	-0.622	-0.427	0.000	1.60	0.02228	94
	**$S_1 \rightarrow r_7$**	3.443	-1.742	0.000	1.79	0.65149	200
B3LYP/aug-cc-pVTZ	$S_1 \rightarrow r_5$	-0.003	-0.001	-0.009	1.58	0.00000	82
	$S_1 \rightarrow r_6$	0.987	-0.258	0.000	1.63	0.04162	114
	**$S_1 \rightarrow r_7$**	-3.352	-1.789	0.000	1.79	0.63157	200
B3LYP/cc-pVQZ	$\underline{S_1 \rightarrow r_5}$	0.003	0.001	0.011	1.56	0.00001	76
	**$S_1 \rightarrow r_6$**	0.910	-0.287	0.000	1.63	0.03627	111
B3P86/cc-pVDZ	$\underline{S_1 \rightarrow r_6}$	-0.466	-0.498	-0.039	1.49	0.01702	33
	**$S_1 \rightarrow r_7$**	-3.636	1.380	-0.283	1.77	0.65807	189
B3PW91/cc-pVDZ	$\underline{S_1 \rightarrow r_6}$	-0.488	-0.491	-0.040	1.48	0.01744	28
	**$S_1 \rightarrow r_7$**	-3.624	1.382	-0.283	1.77	0.65427	189
B98/cc-pVDZ	$S_1 \rightarrow r_6$	-0.316	-0.555	-0.027	1.54	0.01547	63
	**$S_1 \rightarrow r_7$**	-3.632	1.353	-0.282	1.79	0.66419	205
BHHLYP/cc-pVDZ	**$S_1 \rightarrow r_4$**	0.020	0.003	0.005	1.60	0.00002	97
BHHLYP/aug-cc-pVDZ	$S_1 \rightarrow r_4$	0.004	-0.005	-0.005	1.76	0.00000	188
	**$S_1 \rightarrow r_5$**	-0.503	0.532	-0.041	2.03	0.02675	336
BLYP/cc-pVDZ	**$S_1 \rightarrow r_7$**	-3.338	-1.562	0.000	1.49	0.49596	34
	$S_1 \rightarrow r_8$	0.000	-0.001	-0.057	1.70	0.00013	154
BMK/cc-pVDZ	**$S_1 \rightarrow r_5$**	0.552	-0.738	0.042	1.94	0.04041	285
BMK/aug-cc-pVDZ	$S_1 \rightarrow r_4$	0.012	0.004	0.002	1.37	0.00001	-32
	**$S_1 \rightarrow r_5$**	-0.709	0.801	-0.055	1.93	0.05418	279
BP86/cc-pVDZ	**$S_1 \rightarrow r_7$**	-3.484	1.319	-0.270	1.49	0.50759	32
	$S_1 \rightarrow r_8$	0.022	-0.013	-0.052	1.71	0.00014	154
BPBE/cc-pVDZ	**$S_1 \rightarrow r_7$**	-3.484	1.319	-0.269	1.49	0.50852	33
	$S_1 \rightarrow r_8$	0.033	-0.015	-0.052	1.69	0.00017	147
CAM-B3LYP/cc-pVDZ	**$S_1 \rightarrow r_5$**	0.802	-0.102	0.000	1.98	0.03179	311
HCTH/407/cc-pVDZ	**$S_1 \rightarrow r_7$**	-3.412	-1.588	0.000	1.50	0.51938	38
	$S_1 \rightarrow r_8$	0.000	0.000	-0.055	1.69	0.00012	146
HISSbPBE/cc-pVDZ	$\underline{S_1 \rightarrow r_5}$	-0.080	0.009	0.014	1.86	0.00031	243
	$S_1 \rightarrow r_6$	0.163	-0.672	0.011	1.90	0.02226	265
	**$S_1 \rightarrow r_7$**	-3.539	1.384	-0.277	2.06	0.73311	354
HSEH1PBE/cc-pVDZ	$\underline{S_1 \rightarrow r_5}$	0.048	-0.001	0.007	1.42	0.00008	-6
	$\underline{S_1 \rightarrow r_6}$	-0.478	-0.474	-0.040	1.53	0.01710	58
	**$S_1 \rightarrow r_7$**	-3.650	1.436	-0.284	1.80	0.68145	207
LC-OPBE/cc-pVDZ	$\underline{S_1 \rightarrow r_3}$	0.128	-0.593	0.007	1.22	0.01103	-116
	**$S_1 \rightarrow r_4$**	0.011	0.002	0.008	1.67	0.00001	137
LC-wHPBE/cc-pVDZ	$\underline{S_1 \rightarrow r_4}$	0.000	0.000	0.009	1.49	0.00000	33
	**$S_1 \rightarrow r_5$**	0.140	-0.090	0.000	2.07	0.00140	358
LSDA/cc-pVDZ	$S_1 \rightarrow r_7$	-3.442	-1.615	0.000	1.48	0.52431	29
	**$S_1 \rightarrow r_8$**	0.001	0.000	-0.054	1.57	0.00011	79
M05-2X/aug-cc-pVDZ	**$S_1 \rightarrow r_4$**	-0.023	0.005	0.007	1.44	0.00002	8
	$\underline{S_1 \rightarrow r_5}$	-0.290	0.495	-0.021	1.96	0.01584	298
M06/cc-pVDZ	$\underline{S_1 \rightarrow r_5}$	0.000	0.000	-0.011	1.57	0.00000	77
	$\underline{S_1 \rightarrow r_6}$	1.214	-0.062	0.000	1.71	0.06203	159
	**$S_1 \rightarrow r_7$**	-3.301	-1.732	0.000	1.85	0.62971	235

LOT	Excitation	TDM x	TDM y	TDM z	EE (eV)	Osc.	Δr (nm)
	$S_1 \rightarrow r_8$	0.000	0.000	-0.064	1.99	0.00020	317
M06/aug-cc-pVDZ	**$S_1 \rightarrow r_5$**	0.007	0.002	-0.007	1.75	0.00000	180
	$S_1 \rightarrow r_6$	2.539	0.669	0.000	1.77	0.29882	191
	$S_1 \rightarrow r_7$	-2.379	-1.621	0.000	1.92	0.38895	272
M06-HF/cc-pVDZ	$S_1 \rightarrow r_5$	-0.002	-0.001	-0.011	1.57	0.00001	77
	**$S_1 \rightarrow r_6$**	1.213	-0.062	0.000	1.71	0.06193	159
M06L/cc-pVDZ	**$S_1 \rightarrow r_5$**	1.124	0.096	0.086	1.45	0.04561	14
	$S_1 \rightarrow r_6$	-0.035	0.000	-0.013	1.53	0.00005	55
M06L/aug-cc-pVDZ	$S_1 \rightarrow r_5$	-0.011	-0.002	0.006	1.03	0.00000	-222
	$S_1 \rightarrow r_6$	-0.707	-0.368	-0.057	1.27	0.01989	-88
	**$S_1 \rightarrow r_7$**	-3.700	1.444	-0.288	1.52	0.58864	48
M11L/cc-pVDZ	**$S_1 \rightarrow r_6$**	-0.497	-0.372	-0.041	1.28	0.01210	-85
	$S_1 \rightarrow r_7$	-3.773	1.459	-0.293	1.51	0.60863	45
MN15/cc-pVDZ	**$S_1 \rightarrow r_5$**	0.052	0.001	-0.010	1.81	0.00013	216
	$S_1 \rightarrow r_6$	0.418	-0.743	0.029	1.89	0.03359	256
MN15/aug-cc-pVDZ	**$S_1 \rightarrow r_4$**	-0.083	0.011	0.014	1.23	0.00022	-114
	$S_1 \rightarrow r_5$	-0.790	0.865	-0.060	1.89	0.06373	259
mPW1PW91/cc-pVDZ	$S_1 \rightarrow r_5$	-0.037	0.005	0.015	1.51	0.00006	48
	$S_1 \rightarrow r_6$	-0.280	-0.570	-0.024	1.61	0.01590	101
	**$S_1 \rightarrow r_7$**	-3.614	1.344	-0.280	1.85	0.67928	238
mPWLYP/cc-pVDZ	**$S_1 \rightarrow r_7$**	-3.343	-1.565	0.000	1.49	0.49702	34
	$S_1 \rightarrow r_8$	0.000	0.001	-0.057	1.70	0.00013	153
O3LYP/cc-pVDZ	**$S_1 \rightarrow r_7$**	-3.617	1.392	-0.280	1.64	0.60777	120
	$S_1 \rightarrow r_8$	0.043	-0.006	-0.060	1.87	0.00025	244
OLYP/cc-pVDZ	**$S_1 \rightarrow r_7$**	-3.473	1.324	-0.269	1.50	0.51151	42
	$S_1 \rightarrow r_8$	0.025	-0.014	-0.053	1.72	0.00015	161
OPBE/cc-pVDZ	**$S_1 \rightarrow r_7$**	0.029	-0.018	-0.051	1.71	0.00016	155
	$S_1 \rightarrow r_8$	2.265	-1.801	0.172	0.84	0.17341	-328
PBE1PBE/cc-pVDZ	**$S_1 \rightarrow r_5$**	-0.001	0.000	-0.013	1.50	0.00001	39
	$S_1 \rightarrow r_6$	0.199	-0.605	0.000	1.60	0.01587	95
PW6B95D3/cc-pVDZ	$S_1 \rightarrow r_5$	0.081	-0.008	0.001	1.57	0.00025	80
	$S_1 \rightarrow r_6$	-0.181	-0.617	-0.019	1.64	0.01665	119
	**$S_1 \rightarrow r_7$**	-3.570	1.299	-0.278	1.89	0.67277	259
revTPSSh/aug-cc-pVDZ	$S_1 \rightarrow r_5$	0.000	0.000	-0.015	1.53	0.00001	59
	$S_1 \rightarrow r_6$	0.271	-0.561	0.000	1.64	0.01562	120
	**$S_1 \rightarrow r_7$**	-3.398	-1.647	0.000	1.85	0.64449	233
revTPSS/aug-cc-pVDZ	**$S_1 \rightarrow r_7$**	-3.439	-1.626	0.000	1.52	0.53727	49
	$S_1 \rightarrow r_8$	0.001	0.000	-0.052	1.88	0.00012	254
SOGGA11/cc-pVDZ	$S_1 \rightarrow r_7$	-3.454	1.327	-0.265	1.50	0.50535	39
	$S_1 \rightarrow r_8$	0.096	-0.026	-0.053	1.55	0.00048	67
	**$S_1 \rightarrow r_9$**	-0.085	0.006	0.007	1.77	0.00032	192
SOGGA11x/cc-pVDZ	**$S_1 \rightarrow r_5$**	0.953	-0.906	0.073	1.97	0.08383	305
tHCTHhyb/cc-pVDZ	$S_1 \rightarrow r_6$	-0.064	-0.722	0.000	1.37	0.01766	-32
	**$S_1 \rightarrow r_7$**	-0.002	0.000	-0.064	1.93	0.00020	279
TPSSh/cc-pVDZ	**$S_1 \rightarrow r_7$**	-3.494	-1.595	0.000	1.66	0.60097	131
	$S_1 \rightarrow r_8$	0.000	0.000	-0.063	1.94	0.00019	286
TPSS/cc-pVDZ	**$S_1 \rightarrow r_5$**	0.697	0.703	0.000	1.43	0.03447	3
	$S_1 \rightarrow r_6$	0.000	-0.001	-0.015	1.55	0.00001	65
TPSS/aug-cc-pVDZ	**$S_1 \rightarrow r_7$**	-3.449	-1.638	0.000	1.50	0.53468	38
	$S_1 \rightarrow r_8$	0.002	-0.001	-0.051	1.84	0.00012	229
VSXC/cc-pVDZ	**$S_1 \rightarrow r_5$**	0.947	-0.053	0.070	1.53	0.03397	58
	$S_1 \rightarrow r_6$	0.024	-0.003	-0.017	1.58	0.00003	82
VSXC/aug-cc-pVDZ	**$S_1 \rightarrow r_7$**	-3.668	1.407	-0.282	1.50	0.57065	40
	$S_1 \rightarrow r_8$	0.082	-0.005	-0.055	1.93	0.00046	279
wB97XD/cc-pVDZ	$S_1 \rightarrow r_5$	0.674	-0.687	0.051	2.00	0.04547	319
	**$S_1 \rightarrow r_6$**	-0.025	0.005	-0.033	2.14	0.00009	396
X3LYP/cc-pVDZ	$S_1 \rightarrow r_6$	-0.424	-0.519	-0.037	1.52	0.01680	52
	**$S_1 \rightarrow r_7$**	-3.615	1.370	-0.282	1.79	0.65973	203

Table S10 Various hole/electron properties of all computed excited states of the various DFT functionals. **D** gives the distance of the centroids of the hole and electron in Å, **Sr** is the integral of hole and electron with 1 signifying perfect match, **H** is the overall measure of spatial distribution of the hole and electron in Å, **τ** is the measure of hole-electron separation in the CT direction, $\mathbf{E_{coul}}$ is the coulomb attractive energy between hole and electron, **HDI**/**EDI** are hole and electron delocalization indexes, respectively, **x,y,z** is the transition dipole moment from the G. S. in a.u., **EE** the excitation energy in eV (from the G. S.) and **Ex.** is the description of the excitation. Information about state and hole-electron properties is provided by the Multiwfn program.[77,78] State numbers are signified as "roots" (i.e. root 5 = r_5, root 7 = r_7). The first excited singlet state S_1 is usually the first root r_1.

LOT	Excitation	D (Å)	Sr (a.u.)	H (Å)	t (Å)	E_{coul} (eV)	HDI	EDI	x	y	z	EE (eV)	Ex.
APFD/cc-pVDZ	$S_0 \rightarrow S_1 (r_1)$	0.889	0.693	2.861	-1.064	5.20	8.94	9.39	0.993	-2.063	0.068	2.52	ππ*
	$S_0 \rightarrow r_5$	3.148	0.440	2.518	1.478	3.84	16.45	9.38	0.233	0.082	0.064	3.49	nπ*
B1B95/cc-pVDZ	$S_0 \rightarrow S_1 (r_1)$	0.857	0.697	2.852	-1.091	5.23	8.92	9.38	1.024	-2.106	0.070	2.57	ππ*
	$S_0 \rightarrow r_5/r_6$	3.142	0.437	2.478	1.501	4.42	16.39	9.41	0.305	0.052	0.063	3.61	CT
	$S_0 \rightarrow r_7$	1.042	0.709	3.037	-0.937	4.88	7.50	9.28	-0.424	0.873	-0.025	4.22	ππ*
B3LYP/cc-pVDZ	$S_0 \rightarrow S_1 (r_1)$	0.939	0.682	2.871	-0.411	5.37	9.21	9.51	2.132	-0.706	0.000	2.45	ππ*
	$S_0 \rightarrow r_7$	1.287	0.712	3.006	-0.880	5.17	7.18	9.37	-1.072	0.260	0.000	4.00	ππ*
B3LYP/aug-cc-pVDZ	$S_0 \rightarrow S_1 (r_1)$	0.858	0.693	2.928	-0.517	5.30	8.57	9.23	-2.159	-0.745	0.001	2.40	ππ*
	$S_0 \rightarrow r_5$	0.750	0.493	2.471	-1.102	6.12	14.88	9.16	-0.006	0.003	0.099	2.69	nπ*
	$S_0 \rightarrow r_6/r_7$	3.237	0.446	2.504	1.410	4.51	15.75	9.30	-0.398	0.368	-0.042	3.48	nπ*
B3LYP/cc-pVTZ	$S_0 \rightarrow S_1 (r_1)$	0.862	0.693	2.885	-0.455	5.37	8.74	9.45	-2.144	-0.725	0.000	2.45	ππ*
	$S_0 \rightarrow r_7$	1.236	0.473	2.553	-0.650	5.81	14.32	9.35	-0.001	0.000	-0.077	3.99	nπ*
B3LYP/aug-cc-pVTZ	$S_0 \rightarrow S_1 (r_1)$	0.854	0.694	2.913	-0.545	5.33	8.54	9.27	2.158	-0.742	0.000	2.42	ππ*
	$S_0 \rightarrow r_5$	2.841	0.405	2.736	0.532	4.73	10.14	9.06	0.000	0.000	0.094	4.73	nπ*
	$S_0 \rightarrow r_6$	3.239	0.442	2.491	1.422	4.52	15.92	9.35	0.409	0.365	0.043	3.51	CT
	$S_0 \rightarrow r_7$	0.946	0.707	3.161	-0.644	5.04	7.91	9.20	-0.937	0.413	-0.012	3.96	ππ*
B3LYP/cc-pVQZ	$S_0 \rightarrow S_1 (r_1)$	0.859	0.695	2.891	-0.498	5.36	8.61	9.40	2.150	-0.734	0.000	2.44	ππ*
	$S_0 \rightarrow r_6$	3.239	0.440	2.481	1.590	4.37	16.01	9.39	0.498	-0.034	-0.010	3.51	CT
B3P86/cc-pVDZ	$S_0 \rightarrow S_1 (r_1)$	0.922	0.687	2.861	-1.033	5.20	9.06	9.46	0.965	-2.013	0.067	2.47	ππ*
	$S_0 \rightarrow r_7$	1.290	0.708	3.012	-0.656	4.84	7.15	9.33	-0.560	0.936	-0.033	4.03	ππ*
B3PW91/cc-pVDZ	$S_0 \rightarrow S_1 (r_1)$	0.925	0.684	2.872	-1.041	5.75	9.14	9.47	0.970	-2.016	0.067	2.47	ππ*
	$S_0 \rightarrow \mathbf{r_6}$	0.861	0.688	2.893	-1.130	5.07	8.92	9.34	-1.390	2.846	-0.097	2.47	ππ*
	$S_0 \rightarrow r_7$	1.262	0.711	3.012	-0.686	4.24	7.16	9.33	-0.562	0.937	-0.033	4.03	ππ*
B98/cc-pVDZ	$S_0 \rightarrow S_1 (r_1)$	0.906	0.690	2.861	-1.046	5.20	8.98	9.43	0.987	-2.048	0.068	2.48	ππ*
	$S_0 \rightarrow \mathbf{r_6}$	2.963	0.343	2.380	1.437	4.67	21.55	9.63	-0.002	0.006	-0.008	2.74	CT
	$S_0 \rightarrow r_7$	1.157	0.713	3.042	-0.814	4.84	7.27	9.23	-0.507	0.930	-0.029	4.05	ππ*
BHHLYP/cc-pVDZ	$S_0 \rightarrow S_1 (r_1)$	0.603	0.736	2.786	-1.279	5.37	8.01	9.07	1.239	-2.407	0.086	2.89	ππ*
	$S_0 \rightarrow r_4$	2.003	0.406	2.043	0.574	6.06	25.48	10.3	0.005	0.001	-0.048	3.97	nπ*
BHHLYP/aug-cc-pVDZ	$S_0 \rightarrow S_1 (r_1)$	0.593	0.737	2.839	-1.302	5.26	7.75	8.76	1.267	-2.481	0.088	2.82	ππ*
	$S_0 \rightarrow r_4$	2.035	0.395	2.092	0.595	5.90	25.13	9.82	-0.002	-0.003	0.042	4.12	nπ*
	$S_0 \rightarrow r_5$	0.418	0.810	3.007	-1.542	5.05	7.78	7.50	2.770	-1.175	0.228	4.61	ππ*
BLYP/cc-pVDZ	$S_0 \rightarrow S_1 (r_2)$	1.386	0.613	2.925	-0.208	5.14	10.62	9.83	1.828	-0.573	0.000	2.05	ππ*
	$S_0 \rightarrow r_7$	1.988	0.706	2.967	-0.422	5.01	6.93	9.62	-1.327	0.162	-0.006	3.43	ππ*
	$S_0 \rightarrow r_8$	1.017	0.465	2.590	-0.962	5.85	14.81	9.01	0.000	0.000	-0.079	3.29	nπ*
BMK/cc-pVDZ	$S_0 \rightarrow S_1(r_1)/r_5$	0.747	0.717	2.830	-1.182	5.28	8.51	9.20	1.116	-2.268	0.076	2.74	ππ*
BMK/aug-cc-pVDZ	$S_0 \rightarrow S_1 (r_1)$	0.701	0.724	2.880	-1.239	5.19	8.19	8.90	1.153	-2.345	0.079	2.68	ππ*
	$S_0 \rightarrow r_4$	2.225	0.399	2.212	0.725	5.54	23.88	9.54	0.008	-0.001	-0.051	3.64	nπ*
	$S_0 \rightarrow r_5$	0.479	0.776	2.884	-1.291	5.18	8.50	7.95	2.500	-1.214	0.373	4.05	ππ*
BP86/cc-pVDZ	$S_0 \rightarrow S_1 (r_2)$	1.325	0.618	2.937	-0.708	5.00	10.40	9.73	0.855	-1.718	0.060	2.07	ππ*
	$S_0 \rightarrow r_7$	1.990	0.703	2.976	0.025	4.63	6.84	9.54	-0.808	1.051	-0.054	3.45	ππ*
	$S_0 \rightarrow r_8$	1.011	0.466	2.602	-0.784	5.72	14.48	8.94	0.007	0.002	-0.078	3.31	nπ*
BPBE/cc-pVDZ	$S_0 \rightarrow S_1 (r_1)$	1.321	0.618	2.934	-0.711	5.00	10.41	9.74	0.855	-1.717	0.061	2.08	ππ*
	$S_0 \rightarrow r_7$	1.993	0.703	2.973	0.029	4.63	6.85	9.55	-0.805	1.046	-0.053	3.46	ππ*
	$S_0 \rightarrow r_8$	1.006	0.467	2.599	-0.788	5.73	14.49	8.95	0.007	0.001	-0.078	3.30	nπ*
CAM-B3LYP/cc-pVDZ	$S_0 \rightarrow S_1 (r_1)$	0.659	0.732	2.787	-0.651	5.59	8.41	9.33	2.562	-0.761	0.000	2.76	ππ*
	$S_0 \rightarrow r_5$	1.609	0.720	2.474	-0.306	5.66	8.45	9.37	2.057	0.550	0.035	3.39	ππ*
HCTH/407/cc-pVDZ	$S_0 \rightarrow S_1 (r_2)$	1.239	0.626	2.923	-0.227	5.20	10.20	9.75	1.834	-0.594	0.000	2.14	ππ*
	$S_0 \rightarrow r_7$	1.934	0.703	2.964	-0.470	5.04	6.94	9.52	-1.266	0.149	-0.012	3.53	ππ*

LOT	Excitation	D (Å)	Sr (a.u.)	H (Å)	t (Å)	E_{coul} (eV)	HDI	EDI	x	y	z	EE (eV)	Ex.
	$S_0 \rightarrow r_8$	1.007	0.467	2.582	-0.967	5.87	14.58	8.95	-0.001	-0.001	-0.075	3.36	nπ*
HISSbPBE/cc-pVDZ	$S_0 \rightarrow S_1 (r_1)$	0.702	0.719	2.858	-1.213	5.24	8.27	9.14	1.121	-2.250	0.077	2.76	ππ*
	$S_0 \rightarrow r_6$	3.270	0.429	2.445	1.646	4.37	16.45	9.02	0.393	0.058	0.000	4.05	CT
	$S_0 \rightarrow r_7$	0.892	0.705	3.093	-1.179	4.84	8.08	9.15	-0.136	0.744	-0.001	4.48	ππ*
HSEH1PBE/cc-pVDZ	$S_0 \rightarrow S_1 (r_1)$	0.866	0.693	2.880	-1.099	5.18	8.88	9.29	0.992	-2.055	0.068	2.52	ππ*
	$S_0 \rightarrow r_7$	1.208	0.709	3.028	-0.745	4.85	7.20	9.19	-0.517	0.908	-0.030	4.10	ππ*
LC-OPBE/cc-pVDZ	$S_0 \rightarrow S_1 (r_1)$	0.442	0.755	2.685	-1.378	5.58	8.06	9.22	1.441	-2.572	0.102	3.02	ππ*
	$S_0 \rightarrow r_4$	1.608	0.427	1.835	0.294	6.90	26.68	11.2	-0.002	0.004	-0.031	4.05	nπ*
LC-wHPBE/cc-pVDZ	$S_0 \rightarrow S_1 (r_1)$	0.514	0.750	2.691	-0.827	5.78	8.29	9.41	2.816	-0.747	0.000	2.90	ππ*
	$S_0 \rightarrow r_5$	0.282	0.812	2.894	-2.016	5.63	8.19	7.63	-2.686	-1.111	0.067	4.79	ππ*
LSDA/cc-pVDZ	$S_0 \rightarrow S_1 (r_2)$	1.230	0.625	2.949	-0.277	5.17	10.20	9.71	1.817	-0.606	0.000	2.13	ππ*
	$S_0 \rightarrow r_7$	4.546	0.253	2.151	2.979	3.64	26.32	9.52	0.000	0.000	-0.023	1.37	CT
	$S_0 \rightarrow r_8$	1.974	0.701	2.992	-0.458	4.98	6.68	9.47	-1.282	0.114	0.001	3.50	ππ*
LSDA/aug-cc-pVDZ	$S_0 \rightarrow S_1(r_2)/r_1$	4.498	0.258	2.221	2.975	3.46	25.33	9.38	-0.001	0.000	0.032	1.66	CT
	$S_0 \rightarrow r_5$	0.672	1.458	2.831	-0.522	5.06	7.79	9.60	-1.777	1.677	-0.132	2.778	ππ*
M05-2X/cc-pVDZ	$S_0 \rightarrow S_1(r_1)/r_5$	0.679	0.704	2.850	-1.249	5.13	8.23	8.96	1.328	-2.893	0.090	2.83	ππ*
M05-2X/aug-cc-pVDZ	$S_0 \rightarrow S_1 (r_1)$	0.605	0.734	2.845	-1.315	5.26	8.01	8.79	1.247	-2.446	0.087	2.76	ππ*
	$S_0 \rightarrow r_4$	1.867	0.409	2.053	0.436	6.13	25.43	10.1	0.015	-0.007	-0.033	3.72	nπ*
M06/cc-pVDZ	$S_0 \rightarrow S_1 (r_1)$	0.759	0.711	2.861	-0.602	5.44	8.34	9.38	2.280	-0.747	0.000	2.55	ππ*
	$S_0 \rightarrow r_5/r_6/r_7/r_8$	3.141	0.385	2.521	1.367	4.58	18.71	9.26	0.072	0.073	-0.033	3.60	CT
M06/aug-cc-pVDZ	$S_0 \rightarrow S_1 (r_1)$	0.776	0.716	2.903	-0.860	5.37	7.96	9.07	2.324	-0.776	0.000	2.48	ππ*
	$S_0 \rightarrow r_5/r_6$	3.193	0.433	2.551	1.320	4.52	16.57	9.13	0.374	0.283	-0.029	3.72	nπ*
M06-HF/cc-pVDZ	$S_0 \rightarrow S_1 (r_1)$	0.759	0.711	2.861	-0.602	5.44	8.34	9.38	2.280	-0.747	0.000	2.55	ππ*
	$S_0 \rightarrow r_5$	3.130	0.378	2.519	1.363	4.59	18.84	9.30	0.000	0.000	-0.032	3.60	nπ*
M06L/cc-pVDZ	$S_0 \rightarrow S_1 (r_1)/r_6$	4.609	0.241	2.134	3.141	3.39	26.19	9.44	0.002	-0.002	-0.024	1.65	CT
	$S_0 \rightarrow r_5$	3.197	0.363	2.557	1.506	4.39	18.38	9.41	0.002	-0.001	-0.017	2.59	CT
M06L/aug-cc-pVDZ	$S_0 \rightarrow S_1 (r_1)$	0.977	0.666	2.961	-1.017	5.03	9.00	9.32	0.912	-1.893	0.063	2.26	ππ*
	$S_0 \rightarrow r_6$	4.567	0.242	2.196	3.056	3.43	25.29	9.33	0.002	0.004	-0.031	1.90	CT
	$S_0 \rightarrow r_7$	1.677	0.693	3.023	-0.301	4.69	6.95	9.25	-0.691	1.006	-0.042	3.62	ππ*
M11L/cc-pVDZ	$S_0 \rightarrow S_1 (r_1)$	0.924	0.669	2.914	-1.027	5.10	8.73	9.20	0.872	-1.871	0.061	2.36	ππ*
	$S_0 \rightarrow r_6$	1.423	0.701	2.708	-0.474	5.25	7.68	9.29	1.835	-1.611	0.137	3.01	ππ*
	$S_0 \rightarrow r_7$	1.501	0.700	3.003	-0.446	4.77	6.86	9.03	-0.596	0.914	-0.036	3.72	ππ*
MN15/cc-pVDZ	$S_0 \rightarrow S_1 (r_1)$	0.735	0.720	2.826	-1.195	5.29	8.61	9.37	1.176	-2.292	0.081	2.65	ππ*
	$S_0 \rightarrow r_5/r_6$	2.637	0.424	2.531	0.864	4.93	20.36	8.89	-0.003	0.000	0.030	3.97	nπ*
MN15/aug-cc-pVDZ	$S_0 \rightarrow S_1 (r_1)/r_5$	0.735	0.720	2.826	-1.195	5.29	8.62	9.37	1.176	-2.292	0.081	2.65	ππ*
	$S_0 \rightarrow r_4$	2.637	0.425	2.531	0.864	4.93	20.36	8.89	-0.003	0.000	0.030	3.97	CT
mPW1PW91/cc-pVDZ	$S_0 \rightarrow S_1 (r_1)/r_5$	0.869	0.696	2.855	-1.077	5.22	8.88	9.39	1.011	-2.087	0.070	2.55	ππ*
	$S_0 \rightarrow r_6$	3.177	0.433	2.480	1.535	4.39	16.40	9.41	0.290	0.062	0.064	4.53	CT
	$S_0 \rightarrow r_7$	1.099	0.709	3.046	-0.872	4.85	7.35	9.21	-0.454	0.890	-0.025	4.17	ππ*
mPWLYP/cc-pVDZ	$S_0 \rightarrow S_1 (r_2)$	1.377	0.613	2.926	-0.209	5.14	10.60	9.84	1.828	-0.574	0.000	2.05	ππ*
	$S_0 \rightarrow r_7$	1.989	0.706	2.968	-0.422	5.01	6.93	9.62	-1.325	0.160	-0.007	3.43	ππ*
	$S_0 \rightarrow r_8$	1.020	0.465	2.591	-0.959	5.85	14.81	9.00	0.000	0.000	-0.079	3.28	nπ*
O3LYP/cc-pVDZ	$S_0 \rightarrow S_1 (r_1)$	1.051	0.660	2.899	-0.938	5.12	9.50	9.57	0.913	-1.897	0.063	2.34	ππ*
	$S_0 \rightarrow r_7$	1.562	0.709	2.986	-0.385	4.79	7.02	9.38	-0.673	0.980	-0.040	3.82	ππ*
OLYP/cc-pVDZ	$S_0 \rightarrow S_1 (r_2)$	1.301	0.622	2.923	-0.722	5.02	10.35	9.73	0.867	-1.726	0.061	2.12	ππ*
	$S_0 \rightarrow r_7$	1.961	0.704	2.961	0.005	4.66	6.91	9.52	-0.790	1.037	-0.053	3.51	ππ*
	$S_0 \rightarrow r_8$	1.007	0.468	2.589	-0.780	5.75	14.54	8.95	0.008	0.003	-0.075	3.37	nπ*
OPBE/cc-pVDZ	$S_0 \rightarrow S_1 (r_2)$	1.246	0.626	2.931	-0.783	5.03	10.17	9.65	0.858	-1.729	0.062	2.14	ππ*
	$S_0 \rightarrow r_7$	1.962	0.701	2.969	0.003	4.65	6.83	9.45	-0.785	1.017	-0.052	3.54	ππ*
	$S_0 \rightarrow r_8$	0.991	0.470	2.603	-0.808	5.73	14.18	8.89	0.007	0.004	-0.074	3.38	nπ*
PBE1PBE/cc-pVDZ	$S_0 \rightarrow S_1 (r_1)$	0.870	0.696	2.860	-0.455	5.41	8.88	9.36	2.205	-0.729	0.000	2.55	ππ*
	$S_0 \rightarrow r_5/r_6$	3.167	0.436	2.495	1.346	4.58	16.41	9.39	0.147	0.247	-0.043	3.56	nπ*
PW6B95D3/cc-pVDZ	$S_0 \rightarrow S_1 (r_1)$	0.857	0.698	2.854	-1.093	5.22	8.92	9.41	1.028	-2.107	0.070	2.57	ππ*
	$S_0 \rightarrow r_6$	3.142	0.439	2.471	1.495	4.46	16.28	9.45	0.325	0.050	-0.012	3.61	CT
	$S_0 \rightarrow r_7$	1.028	0.711	3.043	-0.956	4.88	7.52	9.23	-0.418	0.874	-0.023	4.21	ππ*
revTPSSh/aug-cc-pVDZ	$S_0 \rightarrow S_1 (r_1)/r_5$	0.860	0.683	2.832	-0.454	5.48	8.96	9.60	2.015	-0.697	0.000	2.58	ππ*
	$S_0 \rightarrow r_6$	1.483	0.690	2.604	-0.614	5.62	8.27	9.75	-2.454	-0.168	0.000	3.23	ππ*
	$S_0 \rightarrow r_7$	1.033	0.633	3.012	-0.856	4.83	7.81	9.26	0.811	0.779	-0.003	4.21	ππ*
revTPSS/aug-cc-pVDZ	$S_0 \rightarrow S_1 (r_1)$	1.119	0.641	2.957	-0.231	2.11	9.56	9.55	1.888	-0.624	0.000	2.11	ππ*

LOT	Excitation	D (Å)	Sr (a.u.)	H (Å)	t (Å)	E_{coul} (eV)	HDI	EDI	x	y	z	EE (eV)	Ex.
	$S_0 \rightarrow r_7$	1.858	0.694	3.002	-0.535	5.00	6.92	9.38	-1.339	0.223	-0.010	3.49	ππ*
	$S_0 \rightarrow r_8$	0.947	0.475	2.617	-1.035	5.81	13.90	9.04	-0.011	0.005	-0.088	3.53	nπ*
SOGGA11/cc-pVDZ	$S_0 \rightarrow S_1$ (r_2)	1.323	0.620	2.912	-0.693	5.04	10.62	9.83	0.861	-1.715	0.060	2.13	ππ*
	$S_0 \rightarrow r_7/r_8$	1.015	0.469	2.574	-0.756	5.78	14.93	9.07	0.015	-0.003	-0.073	3.22	nπ*
	$S_0 \rightarrow r_9$	0.362	3.703	2.253	2.257	4.01	17.28	9.76	-0.757	0.181	-0.060	2.59	CT
SOGGA11x/cc-pVDZ	$S_0 \rightarrow S_1$ (r_1)	0.711	0.723	2.833	-1.206	5.27	8.21	9.07	1.151	-2.297	0.080	2.75	ππ*
	$S_0 \rightarrow r_5$	3.063	0.453	2.446	1.433	4.49	15.14	9.21	0.490	-0.041	0.053	4.17	CT
tHCTHhyb/cc-pVDZ	$S_0 \rightarrow S_1$ (r_1)$_{/r_6}$	1.007	0.670	2.897	-0.336	5.31	9.30	9.53	2.046	-0.687	0.000	2.37	ππ*
	$S_0 \rightarrow r_7$	1.428	0.709	3.000	-0.810	5.13	7.06	9.39	-1.138	0.228	-0.005	3.88	ππ*
TPSSh/cc-pVDZ	$S_0 \rightarrow S_1$ (r_1)	1.110	0.655	2.894	-0.311	5.28	9.70	9.62	1.994	-0.655	0.000	2.32	ππ*
	$S_0 \rightarrow r_7$	1.586	0.710	2.967	-0.726	5.14	6.97	9.45	-1.207	0.212	-0.005	3.83	ππ*
	$S_0 \rightarrow r_8$	1.108	0.471	2.580	-0.827	5.83	14.14	9.15	0.000	0.000	-0.081	3.82	nπ*
TPSS/cc-pVDZ	$S_0 \rightarrow S_1$ (r_2)	4.586	0.249	2.143	3.035	3.60	26.45	9.61	0.000	0.000	-0.023	1.47	CT
	$S_0 \rightarrow r_5/r_6$	3.247	0.356	2.543	1.479	4.48	19.28	9.59	0.000	0.000	0.012	2.34	nπ*
TPSS/aug-cc-pVDZ	$S_0 \rightarrow S_1$ (r_1)	1.112	0.642	2.962	-0.227	5.17	9.52	9.56	1.874	-0.632	0.000	2.10	ππ*
	$S_0 \rightarrow r_7$	1.865	0.693	3.007	-0.532	4.99	6.94	9.39	-1.334	0.220	-0.007	3.46	ππ*
	$S_0 \rightarrow r_8$	0.989	0.473	2.615	-0.993	5.80	14.07	9.00	0.006	-0.004	-0.088	3.48	nπ*
VSXC/cc-pVDZ	$S_0 \rightarrow S_1$ (r_2)$_{/r_6}$	4.642	0.241	2.109	3.194	-	26.84	9.57	0.002	0.000	-0.022	1.51	CT
	$S_0 \rightarrow r_5$	3.285	0.349	2.535	1.640	4.31	19.46	9.56	-0.004	0.000	0.011	2.40	CT
VSXC/aug-cc-pVDZ	$S_0 \rightarrow S_1$ (r_1)	1.057	0.651	2.969	-0.967	5.00	9.38	9.52	0.912	-1.857	0.062	2.15	ππ*
	$S_0 \rightarrow r_7$	1.760	0.696	3.027	-0.232	4.66	6.93	9.36	-0.741	1.085	-0.050	3.51	ππ*
	$S_0 \rightarrow r_8$	1.003	0.476	2.622	-0.813	5.68	14.25	8.86	-0.048	0.116	-0.092	3.60	nπ*
wB97XD/cc-pVDZ	$S_0 \rightarrow S_1$ (r_1)$_{/r_5}$	0.682	0.730	2.761	-1.200	5.41	8.49	9.46	1.229	-2.367	0.085	2.76	ππ*
	$S_0 \rightarrow r_6$	2.656	0.500	2.547	0.925	4.79	15.08	9.29	-0.374	0.011	-0.019	4.28	nπ*
X3LYP/cc-pVDZ	$S_0 \rightarrow S_1$ (r_1)	0.916	0.688	2.860	-1.038	5.20	9.08	9.48	0.993	-2.049	0.069	2.48	ππ*
	$S_0 \rightarrow r_6$	3.135	0.448	2.513	1.457	4.45	16.08	9.47	-0.249	-0.090	0.025	3.44	CT
	$S_0 \rightarrow r_7$	1.195	0.713	3.026	-0.764	4.85	7.25	9.28	-0.523	0.932	-0.031	4.05	ππ*

Table S11 Hole-Electron distribution surfaces of the $S_0 \rightarrow S_n(r_n)$ excitations of each functional. The hole surfaces are depicted in blue colour while green is the position of the electron typically overlapping with the π* MO of lumiflavin. All surfaces were produced with the Multiwfn program.[77] Three main types of states can be clearly distinguished: (i) ππ* (*i.e.* the B3LYP and MPWLYP r_7 states) (ii) intramolecular "Charge transfer" type (*i.e.* APFD, LC-wHPBE) and (iii) nπ* (*i.e.* BPBE and OPBE r_8 states).

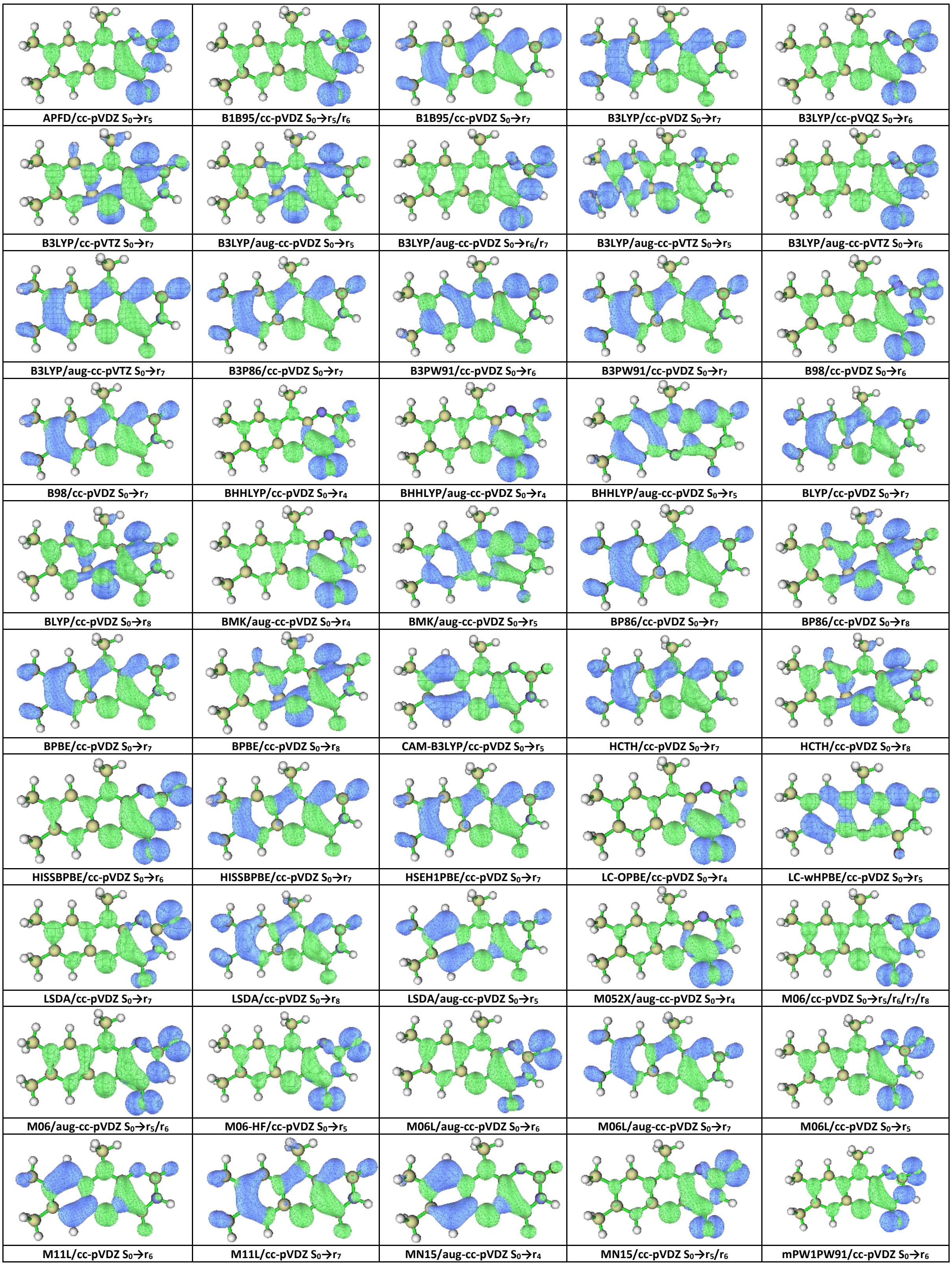

APFD/cc-pVDZ $S_0 \rightarrow r_5$	B1B95/cc-pVDZ $S_0 \rightarrow r_5/r_6$	B1B95/cc-pVDZ $S_0 \rightarrow r_7$	B3LYP/cc-pVDZ $S_0 \rightarrow r_7$	B3LYP/cc-pVQZ $S_0 \rightarrow r_6$
B3LYP/cc-pVTZ $S_0 \rightarrow r_7$	B3LYP/aug-cc-pVDZ $S_0 \rightarrow r_5$	B3LYP/aug-cc-pVDZ $S_0 \rightarrow r_6/r_7$	B3LYP/aug-cc-pVTZ $S_0 \rightarrow r_5$	B3LYP/aug-cc-pVTZ $S_0 \rightarrow r_6$
B3LYP/aug-cc-pVTZ $S_0 \rightarrow r_7$	B3P86/cc-pVDZ $S_0 \rightarrow r_7$	B3PW91/cc-pVDZ $S_0 \rightarrow r_6$	B3PW91/cc-pVDZ $S_0 \rightarrow r_7$	B98/cc-pVDZ $S_0 \rightarrow r_6$
B98/cc-pVDZ $S_0 \rightarrow r_7$	BHHLYP/cc-pVDZ $S_0 \rightarrow r_4$	BHHLYP/aug-cc-pVDZ $S_0 \rightarrow r_4$	BHHLYP/aug-cc-pVDZ $S_0 \rightarrow r_5$	BLYP/cc-pVDZ $S_0 \rightarrow r_7$
BLYP/cc-pVDZ $S_0 \rightarrow r_8$	BMK/aug-cc-pVDZ $S_0 \rightarrow r_4$	BMK/aug-cc-pVDZ $S_0 \rightarrow r_5$	BP86/cc-pVDZ $S_0 \rightarrow r_7$	BP86/cc-pVDZ $S_0 \rightarrow r_8$
BPBE/cc-pVDZ $S_0 \rightarrow r_7$	BPBE/cc-pVDZ $S_0 \rightarrow r_8$	CAM-B3LYP/cc-pVDZ $S_0 \rightarrow r_5$	HCTH/cc-pVDZ $S_0 \rightarrow r_7$	HCTH/cc-pVDZ $S_0 \rightarrow r_8$
HISSBPBE/cc-pVDZ $S_0 \rightarrow r_6$	HISSBPBE/cc-pVDZ $S_0 \rightarrow r_7$	HSEH1PBE/cc-pVDZ $S_0 \rightarrow r_7$	LC-OPBE/cc-pVDZ $S_0 \rightarrow r_4$	LC-wHPBE/cc-pVDZ $S_0 \rightarrow r_5$
LSDA/cc-pVDZ $S_0 \rightarrow r_7$	LSDA/cc-pVDZ $S_0 \rightarrow r_8$	LSDA/aug-cc-pVDZ $S_0 \rightarrow r_5$	M052X/aug-cc-pVDZ $S_0 \rightarrow r_4$	M06/cc-pVDZ $S_0 \rightarrow r_5/r_6/r_7/r_8$
M06/aug-cc-pVDZ $S_0 \rightarrow r_5/r_6$	M06-HF/cc-pVDZ $S_0 \rightarrow r_5$	M06L/aug-cc-pVDZ $S_0 \rightarrow r_6$	M06L/aug-cc-pVDZ $S_0 \rightarrow r_7$	M06L/cc-pVDZ $S_0 \rightarrow r_5$
M11L/cc-pVDZ $S_0 \rightarrow r_6$	M11L/cc-pVDZ $S_0 \rightarrow r_7$	MN15/aug-cc-pVDZ $S_0 \rightarrow r_4$	MN15/cc-pVDZ $S_0 \rightarrow r_5/r_6$	mPW1PW91/cc-pVDZ $S_0 \rightarrow r_6$

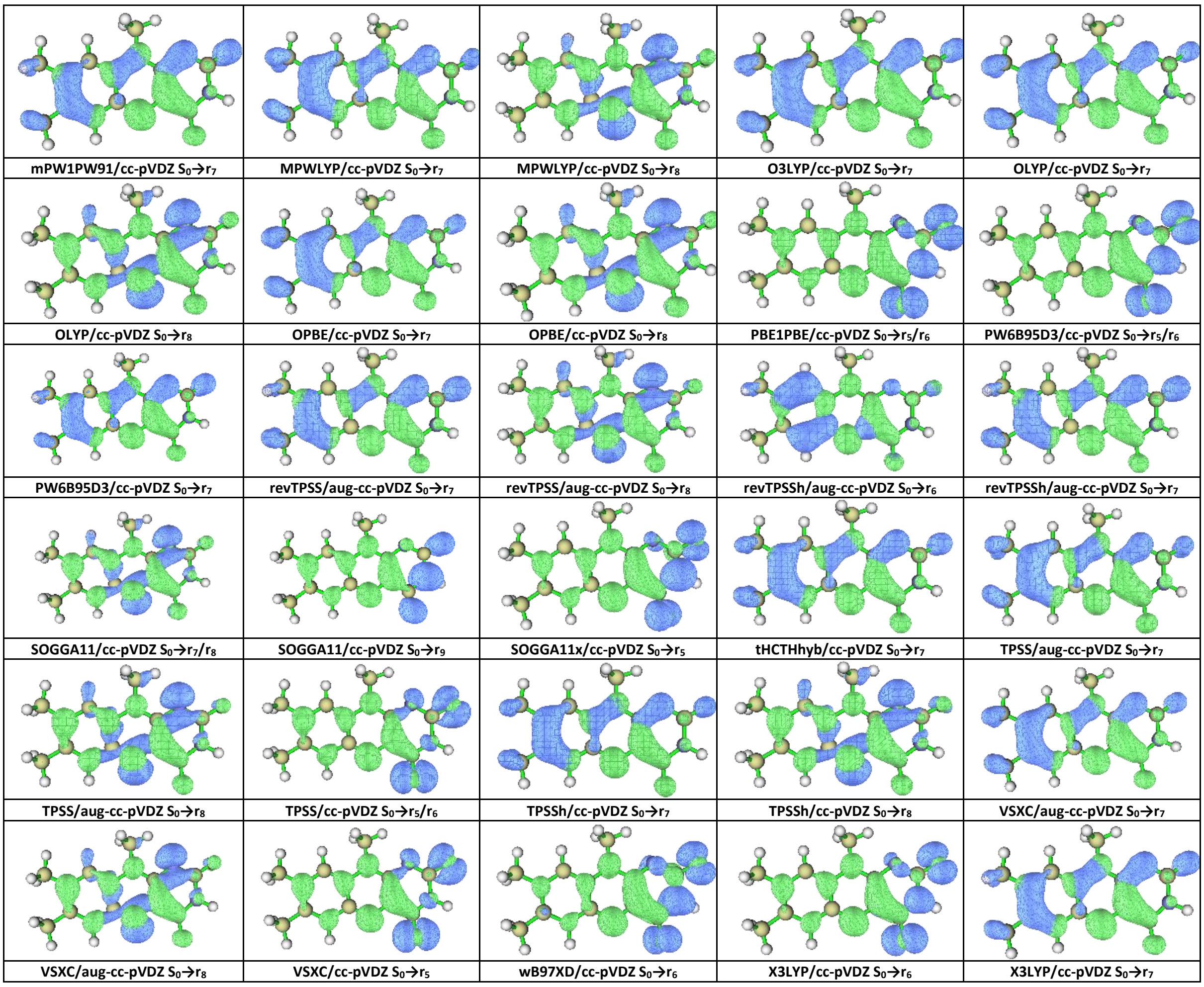

Table S12 Assignment Tables between the experimental FSRS 3rd EAS of 1FMN* (**Exp. FSRS**)[61,76] and the calculated first excited singlet state (S_1) off-Resonance spectra of each DFT functional (**S_1offR**), including vibration number **$v_\#$** and **Assignment** of each vibration to normal modes. The numbering of atoms is taken from **Scheme 1** of the main text. Symmetric and asymmetric C=O stretches are signified as (as) and (**s**). For the functionals HCTH, OLYP and TPSSh, the assigned stretching modes have been colour-coded according to their blue- or red- shift contribution (see the singlet-triplet shift discussion in the main text). Normal modes are ordered from the ones containing the largest to the smallest displacement vectors.

Exp. FSRS	APFD/cc-pvdz			B1B95/cc-pVDZ		
	$v_\#$	S_1offR	Assignment	$v_\#$	S_1offR	Assignment
1200	-	-	-	v_{51}	1263	C_4-N_3, N_3-H, C_2-N_3, C_{10a}-C_{4a}, N_1-C_2, C_9-H, C_{9a}-C_{5a}
	v_{50}	1209	C_6-H, C_9-C_{9a}, C_7-C_6, N_5-C_{4a}, C_{10a}-N_1, C_{4a}-C_4	-	-	-
1250	v_{51}	1243	C_2-N_3, N_3-H, C_{4a}-C_4, N_1-C_2, N_5-C_{4a}, C_7-C_6, C_9-H	v_{53}	1323	N_{10}-C_{10a}, C_{11}-H_3, N_5-C_{4a}, C_8-C_7, C_6-C_{5a}, N_5-C_{4a}
1338	-	-	-	-	-	-
	v_{56}	1368	C_{7a}-H_3, N_3-H, C_{5a}-N_5, C_{10a}-N_1	v_{56}	1378	N_3-H, C_{5a}-N_5, C_{7a}-H_3, C_{10a}-N_1, C_4-N_3
1381	v_{58}	1388	C_{11}-H_3, N_3-H, C_6-H, C_4-N_3, C_7-C_6, C_6-C_{5a}, C_{9a}-N_{10}	-	-	-
	v_{59}	1400	$C_{8a,11}$-H_3, N_3-H, C_9-C_7, C_6-C_{5a}, C_{9a}-C_{5a}, C_4-N_3	v_{58}	1395	$C_{11,7a}$-H_3, N_3-H, C_7-C_6, C_4-N_3, N_{10}-C_{4a}
	v_{60}	1416	C_{11}-H_3, C_{9a}-N_{10}, C_{10a}-N_1, N_1-C_2, C_4-N_3	v_{59}	1406	$C_{11,8a}$-H_3, C_8-C_7, C_{9a}-C_{5a}, N_3-H, C_8-C_9, C_4-N_3
	v_{62}	1422	N_3-H, $C_{7a,11}$-H_3, C_6-H, C_4-N_3, C_6-C_{5a}, C_{9a}-C_{5a}, C_{10a}-C_{4a}	-	-	-

1416	ν_{65}	1448	C_{8a}-H_3, C_9-H, C_{10a}-C_{4a}, N_1-C_2, C_{11}-H_3	ν_{63}	1435	N_3-H, C_6-H, C_{9a}-C_{5a}, C_7-C_6, $C1_{0a}$-C_{4a}, C_4-N_3, C_{10a}-N_1
	-	-	-	ν_{65}	1453	C_8a-H_3, C_9-H, N_1-C_2, C_{10a}-C_{4a}, C_{9a}-C_{5a}
1498	ν_{71}	1539	C_8-C_7, C_{9a}-C_{5a}, $C_{9,6}$-H, C_6-C_{5a}, C_9-C_{9a}, C_{7a}-H_3, N_5-C_{4a}, C_{10a}-C_{4a}	ν_{71}	1553	C_8-C_7, C_9-C_{9a}, C_{9a}-C_{5a}, C_6-C_{5a}, $C_{6,9}$-H, C_{7a}-H_3, N_5-C_{4a}, C_{10a}-C_{4a}
1570	ν_{73}	1650	C_8-C_9, C_6-C_{5a}, $C_{9,6}$-H, C_7-C_6, C_9-C_{9a}, N_5-C_{4a}, C_{10a}-N_1	ν_{73}	1663	C_8-C_9, C_6-C_{5a}, $C_{6,9}$-H, C_7-C_6, N_{10}-C_{9a}, N_5-$C_{4a,}$ C_{10a}-N_1
	ν_{74}	1716	C_2-$O_{2'}$, N_3-H, C_4-$O_{4'}$, C_{10a}-N_1 (as)	ν_{74}	1742	C_2-$O_{2'}$, C_4-$O_{4'}$ (as), N_3-H, C_{4a}-C_{10a}
1626	ν_{75}	1744	C_4-$O_{4'}$, N_3-H, C_2-$O_{2'}$, C_{4a}-C_4 (s)	ν_{75}	1763	C_4-$O_{4'}$, C_2-$O_{2'}$ (s), N_3-H, C_{4a}-C_{10a}
**Exp. RR FMN S_1**	**B3LYP/cc-pVDZ**			**B3LYP/aug-cc-pVDZ**		
	**ν#**	**S_1offR**	**Assignment**	**ν#**	**S_1offR**	**Assignment**
1200	ν_{49}	1191	$C_{6,9}$-H, N_3-H, N_{5}-C_{5a}, C_6-C_7	ν_{49}	1193	$C_{6,9}$-H, C_{11}-H_3, N_3-H, N_3-C_4, C_{5a}-N_5
	-	-	-	-	-	-
1250	ν_{51}	1226	$C_{6,9}$-H, N_3-H, N_3-C_2, C_{10a}-C_{4a}, C_{4a}-C_4	ν_{51}	1223	N_3-H, N_3-C_2, C_{10a}-C_{4a}, C_{4a}-C_4, C_6-H
1338	ν_{54}	1316	$C_{6,9}$-H, N_5-C_{5a}, C_{10a}-N_1, C_{9a}-N_{10}	ν_{54}	1317	$C_{6,9}$-H, N_3-H, N_3-C_2, C_{10a}-C_{4a}, C_{4a}-C_4
	-	-	-	ν_{55}	1339	$C_{6,9}$-H, C_{5a}-N_5 N_{10}-C10a, C_2-N_3
1381	ν_{57}	1379	C_6-H, N_3-H, $C_{11}H_3$, C_{9a}-N_{10}, C_{10a}-C_{4a}	ν_{57}	1376	C_6-H, N_3-H, $C_{11}H_3$, C_6-C_{5a}
	ν_{58}	1381	$C_{7a,8a}$-H_3, N_3-H, C_8-C_7, C_{9a}-C_{5a}	ν_{58}	1382	$C_{7a,8a}$-H_3, C_{9a}-C_{5a}
	ν_{59}	1391	$C_{7a,8a}$-H_3, C_{9a}-N_{10}, N_1-C_2, C_{9a}-C_{5a}	ν_{59}	1394	C_{8a}-H_3, N_3-H, N_5-C_{4a}, N_{10}-C_{10a}
	ν_{61}	1405	N_3-H, C_6-H, C_{9a}-C_{5a}, N_3-C_4, C_{10a}-C_{4a}	-	-	-
1416	ν_{64}	1439	$C_{7a,8a}$-H_3, C_{9a}-N_{10}, N_1-C_2, C_{9a}-C_{5a}	ν_{64}	1436	N_5-C_{4a}, C_{7a}-H_3, C_{11}-H_3, N_3-H
	ν_{65}	1444	N_5-C_{4a}, C_{7a}-H_3, N_3-H, N_4-C_3, C_{10a}-N_1	-	-	-
1498	ν_{71}	1524	C_9-H, C_{9a}-N_{10}, C_8-C_7, C_{5a}-C_{9a}, C_{10a}-C_{4a}	ν_{71}	1514	C_{7a}-H_3, C_{9a}-C_{5a}, C_8-C_7, C_{10a}-C_{4a}
1570	ν_{73}	1630	$C_{6,9}$-H, C_8-C_9, C_{5a}-C_6, C_{10a}-N_1	ν_{73}	1625	$C_{6,9}$-H, C_8-C_9, C_{5a}-C_6, N_3-H, C_2-$O_{2'}$, C_4-$O_{4'}$ (as)
	ν_{74}	1692	C_2-$O_{2'}$, N_3-H, C_4-$O_{4'}$ (as)	ν_{74}	1650	C_2-$O_{2'}$, N_3-H, $C_{6,9}$-H, C_8-C_9, C_{5a}-C_6 (as)
1626	ν_{75}	1719	C_4-$O_{4'}$, N_3-H, C_2-$O_{2'}$ (s)	ν_{75}	1663	C_4-$O_{4'}$, N_3-H, C_2-$O_{2''}$, C_{5a}-C_6 (s)
**Exp. RR FMN S_1**	**B3LYP/cc-pVTZ**			**B3LYP/aug-cc-pVTZ**		
	**ν#**	**S_1offR**	**Assignment**	**ν#**	**S_1offR**	**Assignment**
1200	ν_{49}	1194	$C_{6,9}$-H, C_{11}-H_3, N_3-H, N_3-C_4, C_2-N_1	ν_{49}	1193	$C_{6,9}$-H, N_3-H, N_3-C_4, C_{11}-$H_{3,}$ C_{5a}-N_5
	ν_{51}	1213	C_9-H, N_3-C_2, C_{10a}-C_{4a}, C_{4a}-C_4, C_{11}-H_3	ν_{51}	1211	C_9-H, N_3-C_2, C_{10a}-C_{4a}, C_{4a}-C_4, C_{11}-H_3
1250	ν_{52}	1284	C_9-H, C_8-C_7, C_{7a}-H_3	ν_{52}	1285	C_9-H, C_{5a}-N_5, C_8-C_7, C_{7a}-H_3
1338	ν_{54}	1317	$C_{6,9}$-H, N_5-C_{5a}, C_{10a}-N_{10}	ν_{54}	1316	$C_{6,9}$-H, N_5-C_{5a}, C_9-C_{9a}, C_{10a}-N_{10}, N_3-H
	-	-	-	ν_{55}	1337	$C_{6,9}$-H, N_3-H, N_5-C_{5a}, N_{10}-C_{9a}, C_{10a}-N_1
1381	ν_{57}	1381	C_6-H, N_3-H, $C_{11}H_3$, C_6-C_{5a}, C_{10a}-C_{4a}	ν_{57}	1378	C_6-H, N_3-H, $C_{11}H_3$, C_6-C_{5a}, C_{10a}-C_{4a}
	ν_{58}	1391	N_3-H, N_5-C_{4a}, C_{10a}-N_1, C_{9a}-N_{10}	ν_{58}	1392	N_3-H, N_5-C_{4a}, C_{10a}-N_1, C_{9a}-N_{10}
	-	-	-	-	-	-
	-	-	-	-	-	-
1416	ν_{62}	1429	C_{11}-H_3, N_5-C_{4a}, N_3-H, C_{8a}-H_3	ν_{62}	1428	N_5-C_{4a}, C_{11}-H_3, N_3-H, N_3-C_4
	ν_{63}	1441	C_{11}-H_3, C_{7a}-H_3, N_5-C_{4a}	ν_{63}	1440	C_{11}-H_3, C_{7a}-H_3, N_5-C_{4a}
1498	ν_{71}	1513	C_{7a}-H_3, C_8-C_7, C_{9a}-C_{5a}, C_{10a}-C_{4a}, C_{11}-H_3	ν_{71}	1511	C_{7a}-H_3, C_8-C_7, C_{9a}-C_{5a}, C_{10a}-C_{4a}, C_{11}-H_3
1570	ν_{73}	1625	C_8-C_9, C_6-C_{5a}, C_9-H, N_5-C_{4a}	ν_{73}	1622	C_8-C_9, C_6-C_{5a}, C_9-H, N_3-H, C_2-$O_{2'}$, C_4-$O_{4'}$ (as)
	ν_{74}	1668	C_2-$O_{2'}$, N_3-H, C_4-$O_{4'}$ (as)	ν_{74}	1651	C_2-$O_{2'}$, N_3-H, C_4-$O_{4'}$, C_8-C_9, C_6-C_{5a}, C_9-H (as)
1626	ν_{75}	1686	C_4-$O_{4'}$, C_2-$O_{2'}$, N_3-H (s)	ν_{75}	1664	C_4-$O_{4'}$, C_2-$O_{2'}$, N_3-H (s)
**Exp. RR**	**B3LYP/cc-pVQZ**			**B3P86/cc-pVDZ**		

FMN S_1	$\nu_{\#}$	S_1offR	Assignment	$\nu_{\#}$	S_1offR	Assignment
1200	ν_{49}	1194	$C_{6,9}$-H, N_3-H, N_3-C_4, C_{11}-H_3, C_{5a}-N_5	ν_{49}	1197	C_6-H, C_8-C_7, N_3-H, C_{5a}-N_5, C_7-C_6, C_{10a}-N_1, C_4-N_3
	ν_{51}	1211	C_9-H, N_3-C_2, C_{9a}-C_9, C_{10a}-C_{4a}, C_{4a}-C_4, C_{11}-H_3	ν_{50}	1210	$C_{6,9}$-H, C_{11}-H_3, C_9-C_{9a}, C_{4a}-C_4, C_4-N_3, N_3-H, C_7-C_6, $C1_{0a}$-N_1
1250	ν_{52}	1285	C_9-H, C_{5a}-N_5, C_8-C_7, C_{7a}-H_3	ν_{51}	1248	C_2-N_3, C_{4a}-C_4, N_3-H, C_9-H, N_1-C_2, N_5-C_{4a}, C_{9a}-C_{5a}, C_7-C_6
1338	ν_{54}	1317	$C_{6,9}$-H, N_5-C_{5a}, C_9-C_{9a}, C_{10a}-N_{10}, N_3-H	-	-	-
	-	-	-	ν_{56}	1366	C_{7a}-H_3, N_3-H, $C_{6,9}$-H, C_{10a}-N_1, C_{5a}-N_5
1381	ν_{57}	1379	C_6-H, N_3-H, $C_{11}H_3$, C_6-C_{5a}, C_{10a}-C_{4a}	ν_{58}	1384	$C_{11,7a}$-H_3, N_3-H, C_6-H, C_7-C_6, C_6-C_{5a}, C_{5a}-C_{9a}, C_{9a}-N_{10}, C_{10a}-C_{4a}
	ν_{58}	1393	N_3-H, C_{10a}-N_1, N_5-C_{4a}, C_{9a}-N_{10}	ν_{59}	1398	$C_{8a,11}$-H_3, N_3-H, C_9-H, C_6-C_{5a}, C_4-N_3, C_{9a}-C_{5a}, C_8-C_7, C_2-N_3
	-	-	-	ν_{60}	1414	C_{11}-H_3, C_{9a}-N_{10}, C_{10a}-N_1, $C_{7a,8a}$-H_3, C_{10a}-C_{4a}, N_5-C_{4a}, N_1-C_2
	-	-	-	ν_{61}	1416	N_3-H, C_{11}-H_3, C_6-H, C_{9a}-C_{5a}, C_{10a}-C_{4a}, C_8-C_7, C_4-N_3
1416	ν_{62}	1429	N_5-C_{4a}, C_{11}-H_3, N_3-H, N_3-C_4, N_1-C_2	-	-	-
	ν_{63}	1441	C_{11}-H_3, C_{7a}-H_3, N_5-C_{4a}, N_3-H	ν_{65}	1445	$C_{8a,11,7a}$-H_3, C_9-H, C_{10a}-N_1, C_{10a}-C_{4a}, C_{9a}-C_{5a}
1498	ν_{71}	1512	C_{7a}-H_3, C_8-C_7, C_{9a}-C_{5a}, C_{10a}-C_{4a}, C_{11}-H_3	ν_{71}	1540	C_8-C_7, C_9-C_{9a}, C_{9a}-C_{5a}, C_6-C_{5a}, $C_{6,9}$-H, C_{7a}-H_3, N_5-C_{4a}, C_{10a}-C_{4a}
1570	ν_{73}	1623	$C_{6,9}$-H, C_8-C_9, C_6-C_{5a}, N_3-H, C_2-$O_{2'}$, C_4-$O_{4'}$ (as)	ν_{73}	1648	C_8-C_9, C_6-C_{5a}, $C_{6,9}$-H, C_7-C_6, N_{10}-C_{9a}, N_5-C_{4a}, C_{10a}-N_1
	ν_{74}	1659	C_2-$O_{2'}$, N_3-H, C_4-$O_{4'}$, C_8-C_9, C_6-C_{5a}, C_{10a}-N_1 (as)	ν_{74}	1714	C_2-$O_{2'}$, N_3-H, C_4-$O_{4'}$ (as), C_{4a}-C_{10a}, C_4-N_3
1626	ν_{75}	1675	C_4-$O_{4'}$, C_2-$O_{2'}$, N_3-H (s)	ν_{75}	1742	C_4-$O_{4'}$, N_3-H, C_2-$O_{2'}$, C_4-C_{4a} (s)

Exp. RR FMN S_1	B3PW91/cc-pVDZ			B98/cc-pVDZ		
	$\nu_{\#}$	S_1offR	Assignment	$\nu_{\#}$	S_1offR	Assignment
1200	ν_{49}	1197	C_6-H, C_8-C_7, N_3-H, C_{5a}-N_5, C_7-C_6, C_{10a}-N_1, C_4-N_3	ν_{51}	1228	C_2-N_3, C_{4a}-C_4, N_3-H, C_9-H, N_{10}-C_{10a}, C_{5a}-N_5, C_{5a}-C_{9a}
	ν_{50}	1209	$C_{6,9}$-H, C_{11}-H_3, C_9-C_{9a}, C_{4a}-C_4, C_4-N_3, N_3-H, C_7-C_6, $C1_{0a}$-N_1	-	-	-
1250	ν_{51}	1245	C_2-N_3, C_{4a}-C_4, N_3-H, C_9-H, N_1-C_2, N_5-C_{4a}, C_{9a}-C_{5a}, C_7-C_6	ν_{53}	1294	N_{10}-C_{10}a, C_{9a}-C_{5a}, C_7-C_6, C_{11}-H_3, N_5-C_{4a}, N_3-C_2, $C_{6,9}$-H
1338	-	-	-	ν_{54}	1314	C_{5a}-N_5, $C_{6,9}$-H, C_9-C_{9a}, N_{10}-C_{10a}, C_{10a}-C_{4a}, C_2-N_3
	ν_{56}	1366	C_{7a}-H_3, N_3-H, $C_{6,9}$-H, C_{10a}-N_1, C_{5a}-N_5	-	-	-
1381	ν_{58}	1384	$C_{11,7a}$-H_3, N_3-H, C_6-H, C_7-C_6, C_6-C_{5a}, C_{5a}-C_{9a}, C_{9a}-N_{10}, C_{10a}-C_{4a}	ν_{57}	1378	$C_{11,7a}$-H_3, N_3-H, C_6-H, C_{10a}-C_{4a}, C_7-C_6, C_{9a}-C_{5a}, N_{10}-C_{11}
	ν_{59}	1397	$C_{8a,11}$-H_3, N_3-H, C_9-H, C_6-C_{5a}, C_4-N_3, C_{9a}-C_{5a}, C_8-C_7, C_2-N_3	ν_{58}	1380	$C_{7a,8a}$-H_3, C_8-C_7, C_{9a}-C_{5a}
	ν_{60}	1412	C_{11}-H_3, C_{9a}-N_{10}, C_{10a}-N_1, $C_{7a,8a}$-H_3, C_{10a}-C_{4a}, N_5-C_{4a}, N_1-C_2	ν_{59}	1390	C_{8a}-H_3, C_{9a}-C_{5a}, C_{9a}-N_{10}, C_2-N_3
	ν_{61}	1414	N_3-H, C_{11}-H_3, C_6-H, C_{9a}-C_{5a}, C_{10a}-C_{4a}, C_8-C_7, C_4-N_3	ν_{61}	1406	N_3-H, C_{9a}-C_{5a}, C_6-H, C_4-N_3, N_5-C_{4a}, C_{11}-H_3
1416	-	-	-	ν_{64}	1440	N_5-C_{4a}, C_{7a}-H_3, C_{4a}-C_4, C_{10a}-N_1, N_3-H, C_2-N_3, C_8-C_7
	ν_{65}	1445	$C_{8a,11,7a}$-H_3, C_9-H, C_{10a}-N_1, C_{10a}-C_{4a}, C_{9a}-C_{5a}	ν_{65}	1443	$C_{7a,8a,11}$-H_3, C_{10a}-N_1, C_{10a}-C_{4a}, C_8-C_9
1498	ν_{71}	1537	C_8-C_7, C_9-C_{9a}, C_{9a}-C_{5a}, C_6-C_{5a}, $C_{6,9}$-H, C_{7a}-H_3, N_5-C_{4a}, C_{10a}-C_{4a}	ν_{71}	1519	C_8-C_7, C_9-C_{9a}, C_{9a}-C_{5a}, C_6-C_{5a}, $C_{6,9}$-H, C_{7a}-H_3, N_5-C_{4a}, C_{10a}-C_{4a}

1570	ν_{73}	1645	C_8-C_9, C_6-C_{5a}, $C_{6,9}$-H, C_7-C_6, N_{10}-C_{9a}, N_5-C_{4a}, C_{10a}-N_1	ν_{73}	1626	C_8-C_9, C_6-C_{5a}, $C_{6,9}$-H, C_7-C_6, N_{10}-C_{9a}, N_5-C_{4a}, C_{10a}-N_1
	ν_{74}	1711	C_2-$O_{2'}$, N_3-H, C_4-$O_{4'}$ (as), C_{4a}-C_{10a}, C_4-N_3	ν_{74}	1709	C_2-$O_{2'}$, N_3-H, C_4-$O_{4'}$ (as), C_{4a}-C_{10a}, C_4-N_3
1626	ν_{75}	1739	C_4-$O_{4'}$, N_3-H, C_2-$O_{2'}$, C_4-C_{4a} (s)	ν_{75}	1733	C_4-$O_{4'}$, N_3-H, C_2-$O_{2'}$ (s), C_{4a}-C_{10a}
**Exp. RR FMN S_1**	**BHHLYP/cc-pVDZ**			**BHHLYP/aug-cc-pVDZ**		
	**$\nu_\#$**	**S_1offR**	**Assignment**	**$\nu_\#$**	**S_1offR**	**Assignment**
1200	ν_{46}	1144	C_{11}-H_3, N_3-H, C_4-C_{4a}, C_{9a}-C_{5a}, C_4-N_3, C_{9a}-N_{10}, C_8-C_7	ν_{46}	1146	C_{11}-H_3, N_3-H, C_{4a}-C_{10a}, N_{10}-C_{10a}, C_{9a}-N_{10}, C_4-N_3, C_{8a}-H_3, C_8-C_7
	-	-	-	-	-	-
1250	ν_{50}	1264	C_6-H, $C_{11,7a}$-H_3, C_9-C_{9a}, C_7-C_6, C_{4a}-C_4	ν_{50}	1265	C_6-H, C_7-C_6, C_9-C_{9a}, N_5-C_{4a}, C_2-N_3, C_{10a}-C_{4a}
1338	ν_{54}	1381	C_{5a}-N_5, C_{9a}-C_{5a}, C_{10a}-C_{4a}, C_9-C_{9a}, N_3-H, C_9-H, N_{10}-C_{9a}	ν_{54}	1374	C_{5a}-N_5, C_9-C_{9a}, C_{10a}-C_{4a}, N_3-H, C_9-H, C_{9a}-N_{10}
	-	-	-	-	-	-
1381	-	-	-	ν_{56}	1438	$C_{7a,11}$-H_3, C_6-H, C_7-C_6, C_6-C_{5a}, C_{9a}-N_{10}, N_{10}-C_{10a}, C_{10a}-N_1, C_{5a}-N_5, C_4-N_3
	ν_{57}	1442	C_{11}-H_3, C_6-H, C_7-C_6, C_{9a}-N_{10}, C_{10a}-N_1, C_4-N_3	ν_{57}	1442	$C_{7a,8a}$-H_3, C_8-C_7, C_9-C_{9a}, C_6-C_{5a}
	ν_{58}	1448	C_8-H_3, C_6-C_{5a}, C_{9a}-C_{5a}	ν_{58}	1449	N_3-H, $C_{8a,7a}$-H_3, C_4-N_3, C_6-C_{5a}, C_{10a}-N_1
	-	-	-	-	-	-
1416	ν_{60}	1477	C_{11}-H_3, $C_{7a,8a}$-H_3, C_7-C_6, N_{10}-C_{10a}	ν_{60}	1482	$C_{11,7a,8a}$-H_3, C_{9a}-N_{10}, N_{10}-C_{10a}, N_3-H, N_1-C_2
	ν_{64}	1504	C_{8a}-H_3, C_{10a}-C_{4a}, C_{9a}-C_{5a}, N_3-H	ν_{63}	1507	C_6-H, C_{7a}-H_3, C_{9a}-C_{5a}, C_8-C_7, C_{10a}-C_{4a}, $C_{8a,11}$-H_3, C_{10a}-N_1
1498	ν_{72}	1632	(ν_{71}, ν_{70}) C_{10a}-N_1, N_5-C_{4a}, C_4-N_3, C_8-C_7, C_9-H, N_3-H	ν_{72}	1630	(ν_{70}, ν_{71}) C_{10a}-N_1, C_{4a}-N_3, N_5-C_{4a}, C_8-C_9, N_3-H, C_9-H
1570	ν_{73}	1696	C_8-C_9, C_6-C_{5a}, C_{9a}-N_{10}, C_7-C_6, C_9-H, C_{10a}-N_1, N_5-C_{4a}	ν_{73}	1693	C_8-C_9, C_6-C_{5a}, C_{9a}-N_{10}, C_7-C_6, C_9-H, C_{10a}-N_1, N_5-C_{4a}
	ν_{74}	1801	C_4-$O_{4'}$, N_3-H, C_2-$O_{2'}$ (as)	ν_{74}	1742	N_3-H, C_2-$O_{2'}$, C_4-$O_{4'}$(as)
1626	ν_{75}	1822	C_2-$O_{2'}$, C_4-$O_{4'}$, N_3-H, N_{10}-C_{10a} (s)	ν_{75}	1765	C_2-$O_{2'}$, C_4-$O_{4'}$, N_3-H, C_{10a}-N_1 (s)
**Exp. RR FMN S_1**	**BLYP/cc-pVDZ**			**BMK/cc-pVDZ**		
	**$\nu_\#$**	**S_1offR**	**Assignment**	**$\nu_\#$**	**S_1offR**	**Assignment**
1200	ν_{51}	1168	C_2-N_3, C_{10a}-C_{4a}, $C_{6,9}$-H, N_{10}-C_{4a}, C_9-C_{9a}, C_{9a}-C_{5a}	ν_{50}	1229	C_6-H, C_{11}-H_3, C_4-N_3, N_{10}-C_{10a}, C_9-C_{9a}, C_{4a}-C_4, C_7-C_6, C_{9a}-C_{5a}
	ν_{52}	1219	$C_{6,9}$-H, C_8-C_7, C_6-C_{5a}, C_4-N_3, N_3-H	-	-	-
1250	ν_{53}	1238	$C_{6,9}$-H, N_3-H, C_{11}-H_3, N_{10}-C_{10}a, N_5-C_{4a}, C_8-C_9	ν_{51}	1267	N_3-H, C_2-N_3, C_{4a}-C_4, C_4-N_3, N_5-C_{4a}, C_6-H, C_7-C_6
1338	ν_{54}	1258	$C_{6,9}$-H, C_{9a}-C_{5a}, C_{10a}-C_{4a}, N_5-C_{4a}	ν_{53}	1321	C_9-H, C_8-C_7, C_6-C_{5a}, C_8-C_9, C_{5a}-N_5, $C_{7a,8a,11}$-H_3
	ν_{55}	1284	C_{11}-H_3, N_3-H, N_{10}-C_{10a}, C_{9a}-N_{10}, C_7-C_6, C_{9a}-C_{5a}	-	-	-
1381	ν_{56}	1311	C_{5a}-C_{9a}, C_{4a}-C_4, C_8-C_9, N_1-C_2, C_{9a}-N_{10}, N_5-C_{4a}	ν_{56}	1391	$C_{11,8a,7a}$-H_3, N_3-H, C_8-C_7, C_{5a}-N_5, C_7-C_6, C_{9a}-C_{5a}, C_{10a}-N_1
	ν_{59}	1340	C_{7a}-H_3, N_3-H, N_5-C_{4a}, C_4-N_3	ν_{57}	1395	$C_{8a,7a,11}$-H_3, N_3-H, C_9-H, C_{5a}-N_5, C_{9a}-N_{10}, C_{10a}-C_{4a}, C_{10a}-N_1
	ν_{60}	1343	N_3-H, C_{9a}-C_{5a}, C_6-H, C_{8a}-H_3, C_8-C_7, C_4-N_3, N_5-C_{4a}	ν_{58}	1401	$C_{8a,7a}$-H_3, N_3-H, C_6-C_{5a}, C_8-C_7, C_8-C_9
	-	-	-	ν_{59}	1406	N_3-H, C_{11}-H_3, C_6-H, C_{10a}-C_{4a}, C_2-N_3, C_{9a}-C_{5a}, C_4-N_3
1416	ν_{62}	1366	$C_{11,8a}$-H_3, C_{4a}-C_4, N_1-C_2	ν_{61}	1437	N_3-H, $C_{7a,8a,11}$-H_3, C_{9a}-N_{10}, N_5-C_{4a}, N_1-C_2, C_8-C_7

	ν_{65}	1397	$C_{11,7a,8a}$-H_3, N_{10}-C_{10a}, C_9-C_{9a}	ν_{65}	1465	$C_{7a,8a}$-H_3, N_3-H, C_{5a}-N_5, C_{4a}-C_4, N_1-C_2, C_2-N_3
1498	ν_{71}	1475	$C_{6,9}$-H, C_8-C_7, C_9-C_{9a}, C_6-C_{5a}	ν_{71}	1544	(ν_{72}) C_8-C_7, C_{9a}-C_{5a}, $C_{7a,11}$-H_3, C_9-H, C_{9a}-N_{10}, C_{10a}-C_{4a}, C_{10a}-N_1
1570	ν_{73}	1554	C_8-C_9, C_6-C_{5a}, C_2-$O_{2'}$, $C_{6,9}$-H, N_3-H	ν_{73}	1648	C_9-H, C_8-C_9, C_6-C_{5a}, C_{9a}-N_{10}, C_7-C_6, C_{10a}-N_1, N_5-C_{4a}
	ν_{74}	1568	C_2-$O_{2'}$, N_3-H, C_{10a}-C_{4a}, C_6-C_{5a}, C_8-C_9, C_9-H ~(as)	ν_{74}	1786	C_2-$O_{2'}$, C_4-$O_{4'}$, N_3-H (as)
1626	ν_{75}	1643	C_4-$O_{4'}$, N_3-H, C_{4a}-C_4 ~(s)	ν_{75}	1810	C_2-$O_{2'}$, C_4-$O_{4'}$ (s)
**Exp. RR FMN S_1**	**BMK/aug-cc-pVDZ**			**BP86/cc-pVDZ**		
	**$\nu_\#$**	**S_1offR**	**Assignment**	**$\nu_\#$**	**S_1offR**	**Assignment**
1200	ν_{50}	1227	C_6-H, C_7-C_6, C_9-C_{9a}, C_{10a}-C_{4a}, $C_{11,7a,8a}$-H_3	ν_{48}	1118	C_6-H, C_7-C_6, C_{5a}-N_5, N_5-C_{4a}, C_{10a}-N_1, C_4-N_3
	-	-	-	ν_{51}	1184	C_2-N_3, C_{4a}-C_4, $C_{9,6}$-H, N_3-H
1250	ν_{51}	1260	C_2-N_3, C_4-N_3, N_3-H, $C_{6,9}$-H, C_{4a}-C_4, N_1-C_2, C_7-C_6	ν_{53}	1241	$C_{6,9}$-H, N_3-H, C_{11}-H_3, C_{10a}-N_1, N_5-C_{4a}
1338	ν_{54}	1333	C_8-C_7, C_{9a}-C_{5a}, C_{5a}-N_5, N_3-H, N_{10}-C_{10a}, C_{10a}-C_{4a}, C_4-N_3, N_1-C_2	-	-	-
	ν_{55}	1378	C_{7a}-H_3, C_9-H, C_8-C_7, C_6-C_{5a}, N_3-H, C_4-N_3	ν_{55}	1297	N_3-H, N_{10}-C_{10a}, C_7-C_6, C_6-C_{5a}, C_4-N_3, N_1-C_2
1381	ν_{56}	1390	N_3-H, $C_{7a,11,8a}$-H_3, C_{5a}-N_5, C_{10a}-N_1, C_4-N_3, C_7-C_6, C_{9a}-N_{10}	ν_{57}	1326	N_3-H, $C_{11,7a}$-H_3, C_6-C_{5a}, C_{9a}-N_{10}, C_2-N_1
	ν_{57}	1396	$C_{7a,8a}$-H_3, N_3-H, C_6-C_{5a}, C_8-C_7	-	-	-
	ν_{58}	1401	N_3-H, C_{8a}-H_3, C_{10a}-C_{4a}, C_{9a}-C_{5a}, C_2-N_3	ν_{60}	1346	N_3-H, C_{11}-H_3, C_6-H, N_5-C_{4a}, N_{10}-C_{10a}
	ν_{59}	1406	C_{8a}-H_3, N_3-H, C_6-H, C_4-N_3, C_{10a}-C_{4a}	ν_{61}	1356	C_8-C_7, C_{9a}-C_{5a}, C_9-H, $C_{8a,11}$-H_3, N_3-H
1416	ν_{61}	1441	C_{11}-H_3, C_6-H, N_3-H, C_{9a}-C_{5a}, C_{10a}-C_{4a}, N_{10}-C_{10a}, N_5-C_{4a}	ν_{64}	1391	$C_{7a,8a,11}$-H_3, N_5-C_{4a}, C_{4a}-C_4
	ν_{64}	1463	N_5-C_{4a}, $C_{8a,7a}$-H_3, N_3-H, C_{10a}-N_1, C_2-N_3	ν_{65}	1391	$C_{8a,7a,11}$-H_3, C_{10a}-N_1, C_{5a}-N_5
1498	ν_{71}	1534	(ν_{72}) C_{9a}-N_{10}, C_7-C_6, $C_{11,7a,8a}$-H_3, C_8-C_9, C_{10a}-N_1, C_4-N_3, N_5-C_{4a}	ν_{71}	1487	C_8-C_7, C_6-C_{5a}, C_9-C_{9a}, $C_{6,9}$-H, C_{7a}-H_3
1570	ν_{73}	1638	C_8-C_9, C_6-C_{5a}, C_9-H, C_{9a}-N_{10}, C_7-C_6, C_{10a}-N_1, N_5-C_{4a}	ν_{73}	1572	C_8-C_9, C_6-C_{5a}, $C_{6,9}$-H, C_2-$O_{2'}$, C_{9a}-N_{10}, N_3-H, C_{10a}-N_1
	ν_{74}	1726	C_2-$O_{2'}$, C_4-$O_{4'}$, N_3-H (as)	ν_{74}	1589	C_2-$O_{2'}$, N_3-H, C_8-C_9, N_{10}-C_{10a}, C_{4a}-C_4, C_{9a}-C_{5a} ~(as)
1626	ν_{75}	1753	C_2-$O_{2'}$, C_4-$O_{4'}$ (s)	ν_{75}	1665	C_4-$O_{4'}$, N_3-H, C_{4a}-C_4 ~(s)
**Exp. RR FMN S_1**	**BPBE/cc-pVDZ**			**CAM-B3LYP/cc-pVDZ**		
	**$\nu_\#$**	**S_1offR**	**Assignment**	**$\nu_\#$**	**S_1offR**	**Assignment**
1200	ν_{48}	1126	C_6-H, C_8-C_7, C_{7a}-H_3, C_{5a}-N_5, C_{9a}-N_{10}, C_{11}-H_3	ν_{48}	1181	C_6-H, C_{7a}-H_3, C_8-C_7, C_{5a}-N_5, N_{10}-C_{10a}
	ν_{51}	1191	C_2-N_3, C_{10a}-C_{4a}, $C_{6,9}$-H, N_3-H, C_{5a}-N_5	ν_{49}	1216	$C_{6,9}$-H, N_3-H, C_4-N_3, N_5-C_{4a}, C_8-C_7
1250	ν_{53}	1248	$C_{6,9}$-H, N_3-H, C_{11}-H_3, C_{10a}-N_1, C_8-C_7, N_4-C_{4a}	ν_{50}	1234	C_6-H, C_{11}-H_3, C_{4a}-C_4, N_{10}-C_{10a}, C_8-C_7
1338	ν_{55}	1303	N_{10}-C_{10a}, N_1-C_2, N_3-H, C_{11}-H_3, C_7-C_6, C_5a-N_5	ν_{53}	1319	N_{10}-C_{10a}, C_{11}-H_3, C_{9a}-C_{5a}, N_5-C_{4a}, C_9-H, C_7-C_6
	ν_{57}	1333	N_3-H, C_{11}-H_3, C_6-H, C_{7a}-H_3, C_{9a}-N_{10}, C_6-C_{5a}	ν_{54}	1348	C_{9a}-C_{5a}, C_{10a}-C_{4a}, C_{5a}-N_5, N_3-H, N_{10}-C_{10a}, C_9-H, N_1-C_2
1381	-	-	-	ν_{57}	1402	N_3-H, C_{11}-H_3, C_4-N_3, C_{10a}-N_1, C_{5a}-N_5, C_{9a}-C_{5a}
	ν_{58}	1340	N_3-H. C_{8a}-H_3, N_1-C_2, N_3-C_4, C_8-C_9, C_6-C_{5a}	ν_{58}	1407	$C_{8a,7a}$-H_3, C_{9a}-C_{5a}, C_8-C_7, C_6-C_{5a}
	ν_{60}	1353	N_3-H, C_{11}-H_3, C_6-H, N_5-C_{4a}, C_{10a}-C_{4a}	ν_{59}	1409	N_3-H, $C_{11,7a}$-H_3, C_6-H, C_4-N_3, C_{9a}-N_{10}

	ν_{61}	1363	$C_{6,9}$-H, C_7-C_8, C_{9a}-C_{5a}, N_3-H, C_{11}-H_3, C_{8a}-H_3	ν_{60}	1430	$C_{11,7a,8a}$-H_3, N_{10}-C_{10a}, C_{10a}-N_1, C_{5a}-N_5
1416	ν_{64}	1397	C_{11}-H_3, C_{8a}-H_3, C_{10a}-C_{4a}, C_8-C_9	ν_{64}	1459	C_{8a}-H_3, C_9-H, C_{10a}-C_{4a}, C_2-N_3, C_{9a}-C_{5a}
	-	-	-	ν_{68}	1483	$C_{7a,8a,11}$-H_3, C_{10a}-C_{4a}, C_7-C_6, N_3-H
1498	ν_{71}	1494	C_9-C_{9a}, C_6-C_{5a}, C_8-C_7, $C_{6,9}$-H, C_{7a}-H_3	ν_{72}	1585	(ν_{71}) C_{10a}-N_1, N_{5a}-C_4, C_{9a}-N_{10}, C_4-N_3, C_7-C_6, C_8-C_9, C_{9a}-N_{10}
1570	ν_{73}	1579	C_2-$O_{2'}$, $C_{6,9}$-H, N_3-H, C_8-C_9, C_6-C_{5a}, N_1-C_{10a}, N_5-C_{4a}	ν_{73}	1661	C_8-C_9, C_6-C_{5a}, C_9-H, C_{9a}-N_{10}, N_5-C_{4a}, C_{10a}-N_1
	ν_{74}	1596	C_2-$O_{2'}$, N_3-H, C_8-C_9, C_6-C_{5a}, N_{10}-C_{10a}, C_{4a}-C_4, C_9-H ~(as)	ν_{74}	1754	C_4-$O_{4'}$, N_3-H, C_2-$O_{2'}$ (as)
1626	ν_{75}	1671	C_4-$O_{4'}$, N_3-H ~(s)	ν_{75}	1775	C_4-$O_{4'}$, C_2-$O_{2'}$(s)
**Exp. RR FMN S_1**	**HCTH/407/cc-pVDZ**			**HISSbPBE/cc-pVDZ**		
	**$\nu_\#$**	**S_1offR**	**Assignment**	**$\nu_\#$**	**S_1offR**	**Assignment**
1200	ν_{48}	1150	C_6-H, C_{8a}-H_3, C_{10a}-N_1, N_5-C_{4a}, C_6-C_{5a}, C_7-C_6, C_{5a}-N_5	ν_{51}	1310	C_9-H, N_3-H, C_4-N_3, C_3-C_2, C_{9a}-C_{5a}, C_8-C_7
	ν_{51}	1216	C_2-N_3, C_{10a}-C_{4a}, $C_{9,6}$-H, C_{9a}-C_{5a}, N_{10}-C_{10a}	ν_{52}	1310	C_2-N_3, N_3-H, $C_{6,9}$-H, C_{10a}-C_{4a}, N_{10}-C_{10a}, C_8-C_7
1250	ν_{53}	1274	$C_{6,9}$-H, C_{11}-H_3, N_3-H, C_{10a}-N_1	ν_{53}	1362	N_{10}-C_{10a}, N_5-C_{4a}, C_{9a}-C_{5a}, C_7-C_6, C_{11}-H_3, C_8-C_7, C_2-N_3
1338	ν_{55}	1325	N_3-H, N_{10}-C_{10a}, C_{5a}-N_5, C_{11}-H_3, C_7-C_6, C_{9a}-C_{5a}, C_{10a}-N_1	ν_{54}	1380	C_{5a}-N_5, C_9-C_{9a}, C_{10a}-C_{4a}, C_2-N_3, C_{7a}-H_3, $C_{6,9}$-H, N_3-H
	ν_{56}	1342	C_{7a}-H_3, C_9-H, C_8-C_7	ν_{55}	1399	C_{7a}-H_3, C_8-C_7, N_{10}-C_{10a}, N3-H
1381	ν_{57}	1360	$C_{8a,11}$-H_3, N_3-H, C_{9a}-N_{10}	ν_{57}	1427	N_3-H, C_{5a}-N_5, C_{10a}-N_1, C_4-N_3, C_8-C_7, $C_{7a,8a,11}$-H_3
	ν_{58}	1360	C_{8a}-H_3, N_3-H, C_6-H, C_{9a}-C_9, C_7-C_6	ν_{58}	1430	$C_{11,7a}$-H_3, N_3-H, C_7-C_6, C_6-C_{5a}, C_{10a}-C_{4a}, N_3-C_2
	ν_{59}	1370	N_3-H, C_{8a}-H_3, N_1-C_2, C_{9a}-N_{10}, C_4-N_3, N_5-C_{4a}	ν_{59}	1440	$C_{11,8a,7a}$-H_3, C_8-C_7, C_6-C_{5a}, C_9-C_{9a}, N_3-H
	ν_{61}	1388	N_3-H, C_6-H, C_{9a}-C_{5a}, C_8-C_7, $C_{7a,8a,11}$-H_3, C_{10a}-C_{4a}, C_4-N_3	ν_{60}	1457	$C_{7a,11}$-H_3, N_{10}-C1$0_a$, C_8-C_7, C_4-N_3, N_3-H
1416	ν_{65}	1422	$C_{8a,11}$-H_3, C_9-H, N_{10}-C_{10a}, N_1-C_2, C_8-C_9	ν_{63}	1474	C_{5a}-C_{9a}, C_6-H, N_3-H, C_4-N_3, C_{10a}-N_1
	-	-	-	ν_{65}	1492	$C_{8a,11}$-H_3, N_1-C_2, C_{4a}-C_4, C_{9a}-C_{5a}
1498	ν_{72}	1538	C_8-C_7, C_6-C_{5a}, C_9-C_{9a}, $C_{6,9}$-H	ν_{70}	1580	C_8-C_7, C_9-C_{9a}, C_{9a}-C_{5a}, C_6-C_{5a}, $C_{6,9}$-H, C_{7a}-H_3, N_1-C_{10a}
1570	ν_{73}	1613	C_8-C_9, C_6-C_{5a}, $C_{6,9}$-H, C_9-C_{9a}, C_7-C_6, N_3-H, C_2-$O_{2'}$, C_{10a}-N_1, C_4-N_3	ν_{73}	1702	C_8-C_9, C_6-C_{5a}, $C_{6,9}$-H, C_7-C_6, N_{10}-C_{9a}, N_5-C_{4a}, C_{10a}-N_1
	ν_{74}	1640	C_2-$O_{2'}$, N_3-H, C_{10a}-C_{4a}, C_4-N_3, C_8-C_9	ν_{74}	1794	C_2-$O_{2'}$, N_3-H, C_4-$O_{4'}$ (as), C_{4a}-C_{10a}, C_4-N_3
1626	ν_{75}	1711	C_2-$O_{2'}$, N_3-H, C_{4a}-C_4 ~(s)	ν_{75}	1813	C_4-$O_{4'}$, C_2-$O_{2'}$ (s), N_3-H, C_{4a}-C_{10a}
**Exp. RR FMN S_1**	**HSEH1PBE/cc-pVDZ**			**LC-OPBE/cc-pVDZ**		
	**$\nu_\#$**	**S_1offR**	**Assignment**	**$\nu_\#$**	**S_1offR**	**Assignment**
1200	ν_{49}	1201	$C_{6,9}$-H, N_3-H, C_{8a}-H_3, C_{5a}-N_5, C_7-C_6	-	-	-
	ν_{50}	1215	$C_{6,9}$-H, N_3-H, C_{11}-H_3, C_4-N_3, C_{10a}-N_1, N_{10}-C_{10a}, C_{5a}-N_5	ν_{50}	1266	$C_{6,9}$-H, C_{11}-H_3, C_{4a}-C_4, C_{9a}-C_{5a}
1250	ν_{51}	1257	C_9-H, N_3-H, C_{4a}-C_4, N_1-C_2, N_5-C_{4a}, C_{9a}-C_{5a}	ν_{51}	1308	C_9-H, N_3-H, C_{11}-H_3, N_{10}-C_{10a}, C_4-C_{4a}
1338	-	-	-	ν_{53}	1382	$C_{7a,8a}$-H_3, $C_{6,9}$-H, C_{9a}-C_{5a}, C_8-C_7, N_3-H
	ν_{56}	1369	C_{7a}-H_3, N_3-H, C_6-H, C_{5a}-N_5, C_{10a}-N_1	-	-	-
1381	ν_{58}	1389	C_{11}-H_3, C_6-H, N_3-H, C_{7a}-H_3, C_7-C_6, C_{9a}-N_{10}, C_{10a}-C_{4a}	ν_{59}	1439	$C_{7a,8a}$-H_3, N_3-H, C_{11}-H_3, C_6-H

	ν_{59}	1402	C_{11}-H_3, C_{8a}-H_3, C_9-H, N_3-H, C_8-C_9, C_6-C_{5a}, C_4-N_3	ν_{61}	1459	$C_{7a,8a}$-H_3, C_{11}-H_3, C_9-H, C_{9a}-C_{5a}, C_{11}-N_{10}
	ν_{61}	1422	C_{11}-H_3, C_{8a}-H_3, N_3-H, C_{9a}-C_{5a}, C_{9a}-N_{10}, N_1-C_2, N_5-C_{4a}	ν_{64}	1474	N_3-H, C_{11}-H_3, C_4-N_3, C_9-C_{9a}, C_8-C_7, $C_{6,9}$-H
	ν_{62}	1423	N_3-H, C_6-H, C_{7a}-H_3, C_9-C_{9a}, C_{9a}-C_{5a}, C_4-N_3	ν_{66}	1538	C_{7a}-H_3, C_7-C_6, C_8-C_9, C_{9a}-N_{10}, C_{5a}-N_5, C_{4a}-C_4, C_{10a}-N_1, C_2-N_3
1416	-	-	-	ν_{67}	1557	C_{4a}-C_{10a}, C_2-N_3, C_9-C_{9a}, C_7-C_6, N_3-H, N_3-C_4
	ν_{65}	1449	C_{8a}-H_3, C_{11}-H_3, N_1-C_2, C_{10a}-C_{4a}	ν_{68}	1594	N_5-C_{4a}, C_{9a}-N_{10}, C_6-C_{5a}, C_{10a}-N_1, C_8-C_9, $C_{6,9}$-H, C_{11}-H_3
1498	ν_{71}	1543	C_8-C_7, C_{9a}-C_{5a}, C_9-H, C_{7a}-H_3, N_5-C_{4a}, N_{10a}-C_{4a}	ν_{71}	1701	C_7-C_6, C_{9a}-N_{10}, C_{9a}-C_{5a}, $C_{6,9}$-H, C_{10a}-C_{4a}
1570	ν_{73}	1653	C_8-C_9, C_6-C_{5a}, C_{9a}-N_{10}, $C_{6,9}$-H, C_{10a}-N_1, N_5-C_{4a}, C_2-$O_{2'}$	ν_{73}	1788	C_{10a}-N_1, N_5-C_{4a}, C_4-N_3, C_{5a}-N_5, C_{9a}-N_{10}, C_8-C_9, N_3-H
	ν_{74}	1728	C_2-$O_{2'}$, N_3-H, C_4-$O_{4'}$ (as)	ν_{74}	1865	C_2-$O_{2'}$, C_4-$O_{4'}$ (as), N_3-H, C_{4a}-C_{10a}
1626	ν_{75}	1754	C_4-$O_{4'}$, N_3-H, C_2-$O_{2'}$ (s)	ν_{75}	1901	C_4-$O_{4'}$, C_2-$O_{2'}$ (s), N_3-H, C_{4a}-N_5, C_{10a}-N_1

Exp. RR FMN S_1	LC-wHPBE/cc-pVDZ			LSDA/cc-pVDZ		
	$\nu_\#$	S_1offR	Assignment	$\nu_\#$	S_1offR	Assignment
1200	ν_{54}	1369	N_3-H, C_{9a}-C_{5a}, C_{5a}-N_5, C_{10a}-C_{4a}, C_{7a}-H_3, $C_{6,9}$-H	ν_{49}	1160	$C_{8a,7a}$-H_3, C_8-C_7, C_7-C_6, $C_{6,9}$-H, C_{10a}-N_1, C_{9a}-N_{10}
	ν_{55}	1383	$C_{7a,8a}$-H_3, C_9-H, C_{9a}-C_{5a},	-	-	-
1250	ν_{60}	1437	$C_{7a,8a}$-H_3, C_{11}-H_3, C_4-N_3, C_{5a}-N_5	ν_{53}	1266	C_{10a}-N_1, N_3-H, C_6-H, $C_{11,8a}$-H_3, C_6-C_{5a}
1338	-	-	-	ν_{57}	1322	$C_{7a,11}$-H_3, N_3-H, N_{10}-C_{10a}, C_2-N_3, C_{6a}-N_5
	ν_{64}	1469	$C_{11,8a}$-H_3, C_9-H, N_1-C_2, C_{9a}-C_{5a}, C_4-N_3, C_8-C_7	ν_{58}	1324	C_{11}-H_3, N_3-H, C_{9a}-N_{10}
1381	-	-	-	-	-	-
	ν_{65}	1469	C_{11}-H_3, C_{8a}-H_3, C_9-H, N_1-C_2	ν_{65}	1390	$C_{8a,11}$-H_3, N_1-C_2, C_4-N_3, C_9-C_{9a}, C_8-C_7, N_{10}-C_{10a}
	ν_{66}	1477	C_{7a}-H_3, C_{11}-H_3, C_{5a}-N_5, N_5-C_{4a}, C_4-N_3, C_{10a}-N_1	ν_{66}	1402	C_8-C_7, $C_{7a,11}$-H_3, C_{9a}-C_{5a}, C_{10a}-C_{4a}, C_6-C_{5a}, C_9-C_{9a}, N_1-C_2
	ν_{67}	1499	C_{10a}-C_{4a}, C_9-C_{9a}, C_{4a}-C_4, N_1-C_2, C_{11}-H_3, C_{5a}-N_5	-	-	-
1416	-	-	-	ν_{67}	1413	C_{9a}-C_{5a}, C_{9a}-N_{10}, $C_{11,7a,8a}$-H_3, C_8-C_7, C_{4a}-C_4, N_1-C_2
	ν_{70}	1579	C_6-C_{5a}, C_{5a}-N_5, C_8-C_7, C_{10a}-N_1, $C_{6,9}$-H	-	-	-
1498	ν_{71}	1629	C_7-C_6, C_{9a}-C_{5a}, C_{9a}-N_{10}, $C_{6,9}$-H, N_5-C_{4a}, C_{10a}-C_{4a}	ν_{71}	1529	C_9-C_{9a}, C_8-C_7, C_6-C_{5a}, $C_{9,6}$-H
1570	ν_{72}	1675	N_5-C_{4a}, C_{10a}-N_1, C_6-C_{5a}, C_8-C_9, C_4-N_3, $C_{6,9}$-H	ν_{73}	1623	C_8-C_9, C_6-C_{5a}, $C_{9,6}$-H, C_9-C_{9a}, C_7-C_6, C_{10}-N_1, N_4-C_{4a}, C_2-$O_{2'}$
	ν_{73}	1695	C_8-C_9, C_{10a}-N_1, N_5-C_{4a}, C_6-C_{5a}, C_9-H	ν_{74}	1652	C_2-$O_{2'}$, N_3-H, N_{10}-C_{10a}, C_{4a}-C_4
	ν_{74}	1790	C_4-$O_{4'}$, C_2-$O_{2'}$, N_3-H (as)	-	-	-
1626	ν_{75}	1821	C_2-$O_{2'}$, C_4-$O_{4'}$ (s)	ν_{75}	1730	C_4-$O_{4'}$, N_3-H, C_{4a}-C_4 ~(s)

Exp. RR FMN S_1	LSDA/aug-cc-pVDZ			M05-2X/aug-cc-pVDZ		
	$\nu_\#$	S_1offR	Assignment	$\nu_\#$	S_1offR	Assignment
1200	-	-	-	ν_{50}	1239	C_6-H, C_{11}-H_3, C_9-C_{9a}, C_7-C_6, C_2-N_3, C_{10a}-C_{4a}
	ν_{52}	1259	N_3-H, C_2-$O_{2'}$, C_4-N_3, C_{10a}-N_1, C_6-C_{5a}	-	-	-
1250	ν_{53}	1282	N_3-H, C_2-$O_{2'}$, C_{4a}-C_4, C_4-N_3, C_{11}-H_3, N_{10}-C_{10a}, C_6-C_{5a}, C_8-C_7	ν_{51}	1266	N_3-H, C_4-N_3, N_3-H, N_1-C_2, $C_{6,9}$-H
1338	ν_{54}	1305	N_5-C_{4a}, N_{10}-C_{11}, C_{9a}-C_{5a}, $C_{7a,8a}$-H_3, C_6-H, C_{4a}-C_4, C_8-C_9	ν_{53}	1327	C_{9a}-C_{5a}, N_{10}-C_{10a}, N_4-C_{4a}, C_{11}-H_3, C_8-C_7

	-	-	-	ν_{54}	1346	$\mathrm{N_3{-}H, C_{5a}{-}N_5, C_9{-}C_{9a}, N_{10}{-}C_{10a}, C_9{-}H, C_{4a}{-}C_{10a}}$
1381	-	-	-	ν_{57}	1410	$\mathrm{N_3{-}H, C_{11}{-}H_3, C_4{-}N_3, C_{10a}{-}N_1}$
	ν_{58}	1355	$\mathrm{C_{11,7a}{-}H_3, N_3{-}H, C_{4a}{-}C_4, C_{10a}{-}C_{4a}, C_{9a}{-}C_{5a}, C_2{-}N_3}$	ν_{58}	1412	$\mathrm{N_3{-}H, C_6{-}H, C_{11}{-}H_3, C_7{-}C_6, C_{9a}{-}N_{10}}$
	ν_{62}	1377	$\mathrm{C_{7a,8a,11}{-}H_3, N_3{-}H, C_{11}{-}N_{10}, C_8{-}C_7, N_{10}{-}C_{10a}}$	-	-	-
	ν_{63}	1383	$\mathrm{C_8{-}C_7, C_9{-}C_{9a}, C_{5a}{-}N_5, C_{7a,8a}{-}H_3, N_3{-}H, C_2{-}N_3}$	-	-	-
1416	ν_{67}	1426	$\mathrm{N_3{-}H, C_2{-}N_3, C_{11,8a}{-}H_3, C_8{-}C_9, C_6{-}C_{5a}, C_{9a}{-}C_{5a}, N_{10}{-}C_{10a}}$	ν_{60}	1451	$\mathrm{C_{11,7a,8a}{-}H_3, C_{9a}{-}N_{10}, C_{10a}{-}C_{4a}}$
	ν_{68}	1467	$\mathrm{N_3{-}H, C_2{-}N_3, C_{4a}{-}C_{10a}, C_8{-}C_9, C_7{-}C_6, C_{6,9}{-}H, C_{8a}{-}H_3}$	ν_{63}	1476	$\mathrm{C_{7a,8a,11}{-}H_3, C_9{-}H, C_9{-}C_{9a}, C_{10a}{-}C_{4a}, N_1{-}C_2}$
1498	ν_{71}	1547	$\mathrm{C_9{-}C_{9a}, C_{10a}{-}N_1, N_{10a}{-}C_{10a}, C_8{-}C_7, C_{11}{-}H_3, C_9{-}H, C_6{-}C_{5a}}$	ν_{72}	1594	$\mathrm{C_{10a}{-}N_1, N_5{-}C_{4a}, C_4{-}N_3, C_8{-}C_9, C_{9a}{-}N_{10}, C_9{-}H}$
1570	ν_{72}	1577	$\mathrm{C_8{-}C_7, C_9{-}C_{9a}, C_{9a}{-}C_{5a}, C_6{-}C_{5a}, C_{10a}{-}C_{4a}, C_{6,9}{-}H, C_{7a}{-}H_3, N_1{-}C_{10a}}$	ν_{73}	1662	$\mathrm{C_8{-}C_9, C_6{-}C_{5a}, C_9{-}H, C_{9a}{-}N_{10}, C_{10a}{-}N_1, N_5{-}C_{4a}}$
	ν_{73}	1611	$\mathrm{N_1{-}C_2, C_4{-}O_{4'}, N_3{-}H, C_{10a}{-}C_{4a}}$	ν_{74}	1706	$\mathrm{C_4{-}O_{4'}, C_2{-}O_{2'}, N_3{-}H}$ (as)
1626	ν_{75}	1702	$\mathrm{C_4{-}O_{4'}, N_1{-}C_2, C_2{-}O_{2'}}$ (s), $\mathrm{N_3{-}H, C_{4a}{-}C_4}$	ν_{75}	1731	$\mathrm{C_2{-}O_{2'}, C_4{-}O_{4'}}$ (s)
**Exp. RR FMN S_1**	**M06/cc-pVDZ**			**M06/aug-cc-pVDZ**		
	**$\nu_{\#}$**	**S_1offR**	**Assignment**	**$\nu_{\#}$**	**S_1offR**	**Assignment**
1200	ν_{49}	1183	$\mathrm{C_{6,9}{-}H, N_3{-}H, C_4{-}N_3, C_{5a}{-}N_5, C_8{-}C_7, C_2{-}N_3}$	ν_{49}	1188	$\mathrm{C_{6,9}{-}H, N_3{-}H, C_4{-}N_3, C_{11}{-}H_3, C_{5a}{-}N_5}$
	ν_{50}	1198	$\mathrm{C_{6,9}{-}H, C_9{-}C_{9a}, C_{4a}{-}C_4, N_{10}{-}C_{10a}, C_{10a}{-}N_1, C_2{-}N_3, C_7{-}C_6}$	ν_{50}	1203	$\mathrm{C_{6,9}H, C_2{-}C_3, C_{10a}{-}C_{4a}, C_9{-}C_{9a}, C_{11}{-}H_3}$
1250	ν_{51}	1234	$\mathrm{N_3{-}H, C_4{-}N_3, C_{4a}{-}C_4, N_1{-}C_2, C_9{-}H, N_5{-}C_{4a}, C_{9a}{-}C_{5a}}$	ν_{51}	1235	$\mathrm{N_3{-}H, C_2{-}N_3, N_3{-}C_4, C_{4a}{-}C_4, C_{6,9}{-}H, C_{5a}{-}N_5, C_7{-}C_6}$
1338	ν_{52}	1250	$\mathrm{C_9{-}H, C_8{-}C_7, N_{10}{-}C_{10a}, C_{10a}{-}C_{4a}, N_1{-}C_2, C_9{-}C_{9a}}$	-	-	-
	ν_{56}	1357	$\mathrm{C_{8a,11}{-}H_3, N_3{-}H, C_{5a}{-}N_5, C_{10a}{-}N_1, C_4{-}N_3, C_{9a}{-}N_{10}, C_8{-}C_7}$	ν_{56}	1360	$\mathrm{N_3{-}H, C_{11}{-}H_3, C_{10a}{-}N_1, C_{5a}{-}N_5, N_3{-}C_4, C_{9a}{-}N_{10}}$
1381	ν_{58}	1367	$\mathrm{N_3{-}H, C_6{-}H, C_{10a}{-}C_{4a}, C_{11}{-}H_3, C_{9a}{-}C_{5a}, C_7{-}C_6, N_3{-}C_2}$	ν_{57}	1367	$\mathrm{N_3{-}H, C_6{-}H, C_{7a,11}{-}H_3, C_7{-}C_6, C_{10a}{-}C_{4a}, C_{9a}{-}C_{5a}}$
	ν_{59}	1380	$\mathrm{C_{8a,7a,11}{-}H_3, C_6{-}C_{5a}, C_8{-}C_7, C_{6,9}{-}H}$	ν_{58}	1375	$\mathrm{C_{8a}{-}H_3, N_3{-}H, C_4{-}N_3, C_{9a}{-}C_{5a}, C_7{-}C_8}$
	ν_{63}	1410	$\mathrm{N_3{-}H, C_{9a}{-}C_{5a}, C_6{-}H, C_{10a}{-}C_{4a}, C_4{-}N_3}$	ν_{59}	1385	$\mathrm{C_{7a,8a,11}{-}H_3, N_3{-}H, C_6{-}C_{5a}, C_8{-}C_9, N_{10}{-}C_{10a}}$
	ν_{66}	1425	$\mathrm{C_{8a,11}{-}H_3, C_{10a}{-}C_{4a}, C_4{-}N_3, C_9{-}H, N_1{-}C_2, C_8{-}C_7}$	-	-	-
1416	ν_{68}	1450	$\mathrm{N_{10}{-}C_{10a}, N_5{-}C_4, C_{11}{-}H_3, C_{10a}{-}N_1, C_9{-}C_{9a}, C_8{-}C_7}$	ν_{66}	1433	$\mathrm{C_{8a}{-}H_3, C_9{-}H, C_{11}{-}H_3, N_1{-}C_2, C_4{-}N_3, C_6{-}C_{5a}}$
	ν_{69}	1460	$\mathrm{N_5{-}C_{4a}, C_8{-}C_7, C_{10a}{-}N_1, C_{7a,8a}{-}H_3, C_{9a}{-}C_{5a}, C_2{-}N_3, N_3{-}H}$	ν_{69}	1457	$\mathrm{C_{8a,11}{-}H_3, N_5{-}C_{4a}, N_{10}{-}C_{10a}, N_1{-}C_2, C_4{-}N_3}$
1498	ν_{71}	1526	$\mathrm{C_8{-}C_7, C_{9a}{-}C_{5a}, C_{10a}{-}C_{4a}, C_{6,9}{-}H, C_{7a}{-}H_3}$	ν_{71}	1518	$\mathrm{C_8{-}C_7, C_{9a}{-}C_{5a}, C_{10a}{-}C_{4a}, C_{6,9}{-}H, C_{7a}{-}H_3}$
1570	ν_{73}	1646	$\mathrm{C_8{-}C_9, C_6{-}C_{5a}, C_{6,9}{-}H, C_{10a}{-}N_1, C_{4a}{-}C_4}$	ν_{73}	1644	$\mathrm{C_8{-}C_9, C_6{-}C_{5a}, C_{6,9}{-}H, C_{9a}{-}N_{10}, N_4{-}C_{4a}, C_2{-}O_{2'}, C_{10a}{-}N_1}$
	ν_{74}	1747	$\mathrm{C_2{-}O_{2'}, C_4{-}O_{4'}}$ (as), $\mathrm{N_3{-}H, C_{4a}{-}C_4, C_4{-}N_3}$	ν_{74}	1696	$\mathrm{C_2{-}O_{2'}, N_3{-}H, C_4{-}O_{4'}}$ (as)
1626	ν_{75}	1770	$\mathrm{C_2{-}O_{2'}, C_4{-}O_{4'}}$ (s), $\mathrm{N_3{-}H, C_{4a}{-}C_4}$	ν_{75}	1717	$\mathrm{C_4{-}O_{4'}, C_2{-}O_{2'}}$ (s)
**Exp. RR FMN S_1**	**M06-HF/cc-pVDZ**			**M06L/cc-pVDZ**		
	**$\nu_{\#}$**	**S_1offR**	**Assignment**	**$\nu_{\#}$**	**S_1offR**	**Assignment**
1200	ν_{49}	1183	$\mathrm{C_{6,9}{-}H, N_3{-}H, C_{11,8a}{-}H_3, C_4{-}N_3, C_8{-}C_7, N_{10}{-}C_{10a}}$	ν_{51}	1255	$\mathrm{C_9{-}H, C_2{-}N_3, C_{10a}{-}C_{4a}, C_4{-}N_3, N_3{-}H, C_6{-}C_{5a}, C_8{-}C_9}$
	ν_{50}	1198	$\mathrm{C_{6,9}{-}H, C_4{-}N_3, C_{4a}{-}C_4, C_{10a}{-}N_1, C_{9a}{-}N_{10}}$	ν_{52}	1266	$\mathrm{C_{6,9}{-}H, C_2{-}O_{2'}, N_3{-}H, C_8{-}C_7, C_{9a}{-}N_{10}}$

1250	ν_{51}	1234	N$_3$-H, C$_9$-H, C$_2$-N$_3$, C$_{4a}$-C$_4$, N$_5$-C$_{4a}$, C$_7$-C$_6$	ν_{53}	1301	C$_{6,9}$-H, C$_2$-O$_{2'}$, C$_{4a}$-C$_4$, N$_3$-H, N$_{10}$-C$_{10a}$, C$_9$-C$_{9a}$, C$_8$-C$_7$
1338	ν_{54}	1317	C$_{9a}$-C$_{5a}$, C$_{5a}$-N$_5$, C$_{10a}$-C$_{4a}$, C$_6$-H, C$_{7a}$-H$_3$, N$_{10}$-C$_{10a}$	ν_{55}	1347	C$_4$-C$_{4a}$, C$_{10a}$-N$_1$, C$_{9a}$-N$_{10}$, C$_{5a}$-N$_5$, N$_3$-H, C$_8$-C$_7$, C$_{11,8a,7a}$-H$_3$
	ν_{56}	1357	C$_{11,8a}$-H$_3$, N$_3$-H, C$_{5a}$-N$_5$, C$_{10a}$-N$_1$	ν_{56}	1365	C$_{5a}$-N$_5$, C$_8$-C$_7$, C$_{9a}$-C$_{5a}$, C$_{4a}$-C$_4$, N$_3$-H, C$_9$-H, C$_{7a,8a}$-H$_3$
1381	ν_{57}	1363	C$_{8a,11}$-H$_3$, C$_{10a}$-N$_1$, C$_{5a}$-N$_5$	ν_{61}	1424	C$_{11}$-H$_3$, N$_3$-H, N$_{10}$-C$_{10a}$, C$_2$-N$_3$, C$_7$-C$_6$, C$_8$-C$_9$
	ν_{58}	1367	N$_3$-H, C$_6$-H, C$_{11}$-H$_3$, C$_{10a}$-C$_{4a}$, C$_4$-N$_3$, C$_7$-C$_6$	ν_{63}	1432	C$_{7a,8a}$-H$_3$, C$_{5a}$-N$_5$, C$_7$-C$_6$
	ν_{59}	1380	C$_{7a,8a,11}$-H$_3$, C$_6$-C$_{5a}$, C$_{10a}$-N$_1$	-	-	-
	-	-	-	ν_{65}	1446	C$_{11,7a,8a}$-H$_3$, C$_{9a}$-C$_{5a}$, C$_{6,9}$-H
1416	ν_{63}	1410	N$_3$-H, C$_{5a}$-C$_{9a}$, C$_6$-H, C$_{10a}$-C$_{4a}$, C$_4$-N$_3$	ν_{67}	1458	C$_{8a,11}$-H$_3$, N$_3$-H, C$_{9a}$-N$_{10}$, C$_6$-C$_{5a}$, C$_8$-C$_9$, C$_{10a}$-N$_1$
	ν_{66}	1425	C$_{8a,11}$-H$_3$, C$_9$-H, C$_{10a}$-C$_{4a}$, N$_1$-C$_2$, C$_{4a}$-C$_4$	ν_{68}	1490	N$_3$-H, C$_{10a}$-C$_{4a}$, C$_2$-C$_3$, C$_8$-C$_9$, C$_7$-C$_6$, C$_{10a}$-C$_{4a}$, C$_{6,9}$-H, C$_{11,7a,8a}$-H$_3$
1498	ν_{71}	1526	C$_8$-C$_7$, C$_{9a}$-C$_{5a}$, C$_{10a}$-C$_{4a}$, C$_{9,6}$-H, C$_{7a}$-H$_3$	ν_{71}	1531	C$_8$-C$_7$, C$_9$-C$_{9a}$, C$_6$-C$_{5a}$, C$_{6,9}$-H, C$_{10a}$-N$_1$, N$_5$-C$_{4a}$, C$_{8a}$-H$_3$
1570	ν_{73}	1646	C$_8$-C$_9$, C$_6$-C$_{5a}$, C$_{9,6}$-H, C$_{10a}$-N$_1$, N$_5$-C$_{4a}$	ν_{73}	1626	N$_1$-C$_2$, N$_3$-H, C$_4$-O$_{4'}$, C$_4$-N$_3$, C$_{9a}$-C$_{5a}$, C$_8$-C$_7$
	ν_{74}	1747	C$_2$-O$_{2'}$, N$_3$-H, C$_4$-O$_{4'}$ (as)	ν_{74}	1661	C$_8$-C$_9$, C$_6$-C$_{5a}$, C$_7$-C$_6$, C$_{6,9}$-H, C$_9$-C$_{9a}$, C$_{10a}$-C$_{4a}$
1626	ν_{75}	1770	C$_4$-O$_{4'}$, C$_2$-O$_{2'}$, N$_3$-H (s)	ν_{75}	1761	C$_4$-O$_{4'}$, C$_2$-O$_{2'}$ (s), N$_3$-H, C$_{4a}$-C$_4$, C$_{10a}$-N$_1$
**Exp. RR FMN S$_1$**	**M06L/aug-cc-pVDZ**			**M11L/cc-pVDZ**		
	**$\nu_{\#}$**	**S$_1$offR**	**Assignment**	**$\nu_{\#}$**	**S$_1$offR**	**Assignment**
1200	ν_{48}	1174	C$_6$-H, C$_{7a,8a}$-H$_3$, C$_8$-C$_7$, C$_8$-C$_9$, N$_{10}$-C$_{10a}$, N$_5$-C$_{4a}$	-	-	-
	ν_{49}	1180	C$_6$-H, C$_{5a}$-N$_5$, C$_7$-C$_6$, C$_{7a,11}$-H$_3$, N$_3$-H	ν_{49}	1178	C$_{8a,7a}$-H$_3$, C$_6$-H, C$_8$-C$_7$, C$_7$-C$_6$, N$_3$-H
1250	ν_{51}	1226	N$_3$-H, C$_2$-N$_3$, C$_{9,6}$-H, C$_{4a}$-C$_4$, N$_5$-C$_{4a}$, C$_{5a}$-C$_{9a}$, N$_{10}$-C$_{10a}$	ν_{52}	1269	N$_3$-H, C$_2$-N$_3$, C$_{4a}$-C$_4$, N$_5$-C$_{4a}$, C$_9$-H, C$_{5a}$-N$_5$
1338	ν_{54}	1308	C$_{6,9}$-H, C$_{9a}$-C$_{5a}$, C$_{10a}$-C$_{4a}$, C$_{5a}$-N$_5$, C$_{10a}$-N$_1$, C$_{7a}$-H$_3$	ν_{53}	1306	C$_6$-H, C$_{11,8a}$-H$_3$, N$_3$-H, C$_{10a}$-N$_1$, N$_5$-C$_{4a}$, C$_8$-C$_7$, C$_2$-N$_3$, C$_6$-C$_{5a}$
	ν_{56}	1363	C$_{7a}$-H$_3$, C$_9$-H, C$_{11}$-H$_3$, C$_{9a}$-C$_{5a}$	ν_{55}	1339	C$_{7a}$-H$_3$, C$_6$-H, C$_{9a}$-C$_{5a}$, C$_8$-C$_7$, C$_{10a}$-N$_1$
1381	ν_{57}	1370	N$_3$-H, C$_6$-H, C$_{11,7a}$-H$_3$, C$_{9a}$-N$_{10}$, C$_6$-C$_{5a}$, C$_7$-C$_6$, N$_5$-C$_{4a}$	ν_{56}	1357	C$_{7a,11}$-H$_3$, N$_3$-H, C$_6$-C$_{5a}$, C$_{10a}$-C$_{4a}$, C$_{10a}$-N$_1$, C$_{9a}$-N$_{10}$
	ν_{58}	1382	C$_{8a}$-H$_3$, C$_{11}$-H$_3$, N$_1$-C$_2$, C$_{9a}$-N$_{10}$	ν_{58}	1375	C$_{11}$-H$_3$, C$_6$-H, N$_3$-H, C$_7$-C$_6$, N$_{10}$-C$_{10a}$, C$_{9a}$-C$_{5a}$
	ν_{59}	1389	C$_8$a-H$_3$, C$_8$-C$_9$, C$_6$-C$_{5a}$, C$_{9a}$-N$_{10}$, N$_5$-C$_{4a}$, C$_{10a}$-N$_1$, C$_4$-N$_3$	ν_{60}	1387	C$_{11,8a,7a}$-H$_3$, N$_{10}$-C$_{10}$a, C$_6$-C$_{5a}$, C$_{9a}$-C$_{5a}$
	ν_{61}	1403	N$_3$-H, C$_6$-H, C$_4$-N$_3$, C$_{9a}$-C$_{5a}$, C$_{11}$-H$_3$	ν_{63}	1414	C$_{11,8a,7a}$-H$_3$, C$_8$-C$_7$, C$_{9a}$-C$_{5a}$, C$_4$-N$_3$
1416	ν_{65}	1438	C$_{7a,8a,11}$-H$_3$, C$_{10a}$-N$_1$, N$_5$-C$_{4a}$	ν_{66}	1439	C$_{11,8a,7a}$-H$_3$, C$_9$-H, N$_1$-C$_2$, N$_5$-C$_{4a}$, C$_8$-C$_7$, C$_{9a}$-C$_{5a}$
	ν_{66}	1440	C$_{7a,8a}$-H$_3$, N$_5$-C$_{4a}$, C$_4$-N$_3$, C$_{10a}$-C$_4$	ν_{69}	1486	C$_{11}$-H$_3$, C$_{4a}$-C$_4$, N$_5$-C$_{4a}$, N$_1$-C$_2$, N$_{10}$-C$_{4a}$, C$_8$-C$_7$
1498	ν_{71}	1535	C$_8$-C$_7$, C$_{9a}$-C$_{5a}$, C$_{6,9}$-H, C$_{7a}$-H$_3$, C$_{10a}$-C$_{4a}$	ν_{71}	1543	C$_8$-C$_7$, C$_{9a}$-C$_{5a}$, C$_{6,9}$-H, C$_9$-C$_{9a}$, C$_6$-C$_{5a}$, C$_{10a}$-C$_{4a}$, C$_{7a}$-H$_3$
1570	ν_{73}	1635	C$_2$-O$_{2'}$, N$_3$-H, C$_{6,9}$-H, C$_8$-C$_9$, C$_6$-C$_{5a}$, C$_4$-O$_{4'}$, N$_5$-C$_{4a}$ (as)	ν_{73}	1664	C$_8$-C$_9$, C$_6$-C$_{5a}$, C$_{6,9}$-H, C$_{9a}$-N$_{10}$, C$_7$-C$_6$, N$_5$-C$_{4a}$, C$_{10a}$-N$_1$
	ν_{74}	1652	C$_2$-O$_{2'}$, C$_8$-C$_9$, C$_6$-C$_{5a}$, N$_3$-H, C$_{10a}$-N$_1$, N$_5$-C$_{4a}$, C$_{9a}$-N$_{10}$	ν_{74}	1750	C$_2$-O$_{2'}$, N$_3$-H, C$_{10a}$-N$_1$, C$_4$-O$_{4'}$ (as)
1626	ν_{75}	1695	C$_4$-O$_{4'}$, N$_3$-H, C$_{4a}$-C$_4$, C$_6$-C$_{5a}$, C$_2$-O$_{2'}$ (s)	ν_{75}	1789	C$_4$-O$_{4'}$, N$_3$-H, C$_{4a}$-C$_4$, C$_2$-O$_{2'}$ (s)
**Exp. RR**	**MN15/cc-pVDZ**			**MN15/aug-cc-pVDZ**		

FMN S_1	$\nu_\#$	S_1offR	Assignment	$\nu_\#$	S_1offR	Assignment
1200	ν_{49}	1196	$C_{6,9}$-H, N_3-H, C_{8a}-H_3, C_4-N_3, C_7-C_6, C_{5a}-N_5	-	-	-
	ν_{50}	1211	$C_{6,9}$-H, C_{11}-H_3, C_9-C_{9a}, C_{4a}-C_4, C_7-C_6	ν_{50}	1214	C_6-H, C_{11}-H_3, C_9-H, C_9-C_{9a}, C_{4a}-C_4, C_7-C_6
1250	ν_{52}	1264	N_3-H, $C_{6,9}$-H, C_2-N_3, C_{4a}-C_4, N_{10}-C_{10a}	ν_{51}	1258	C_9-H, N_3-H, C_4-N_3, N_1-C_2
1338	ν_{53}	1313	N_{10}-C_{10a}, C_{11}-H_3, N_5-C_{4a}, C_6-C_{5a}, C_2-N_3, C_{8a}-H_3, C_8-C_7	ν_{53}	1308	N_{10}-C_{10a}, C_{5a}-N_5, N_5-C_{4a}, C_{9a}-C_{5a}, C_7-C_6, C_{11}-H_3
	ν_{55}	1351	C_{7a}-H_3, C_9-H, N_3-H, C_8-C_7, C_{10a}-C_{4a}, C_2-N_3	-	-	-
1381	ν_{58}	1384	$C_{11,7a}$-H_3, C_6-H, C_6-C_{5a}, C_7-C_6, C_{10a}-N_1	ν_{58}	1386	$C_{11,7a}$-H_3, C_6-H, C_7-C_6, C_6-C_{5a}, C_{5a}-N_5, C_{10a}-N_1
	ν_{59}	1391	$C_{11,8a,7a}$-H_3, C_8-C_7, C_{9a}-C_{5a}, C_6-C_{5a}	ν_{59}	1393	$C_{7a,8a,11}$-H_3, C_8-C_7, C_6-C_{5a}, C_{9a}-C_{5a}
	ν_{61}	1405	$C_{7a,11,8a}$-H_3, N_{10}-C_{10a}, C_{10a}-N_1, N_3-H, C_4-N_3	ν_{64}	1438	$C_{7a,8a}$-H_3, C_6-H, C_{9a}-C_{5a}, C_{5a}-N_5, C_{10a}-C_{4a}
	-	-	-	ν_{65}	1446	$C_{8a,11}$-H_3, C_9-H, N_1-C_2, C_{10a}-C_{4a}, C_6-C_{5a}, C_9-C_{9a}
1416	ν_{67}	1457	C_6-H, C_7-C_6, $C_{7a,11}$-H_3, C_6-C_{5a}, C_{9a}-C_{5a}, C_{10a}-C_{4a}, C_7-C_6, N_5-C_{4a}	ν_{67}	1456	C_{7a}-H_3, C_6-H, C_7-C_6, C_6-C_{5a}, N_5-C_{4a}, C_{10a}-C_{4a}
	ν_{68}	1470	C_{11}-H_3, C_{9a}-N_{10}, C_{10a}-N_1, N_{10}-C_{10a}, N_5-C_{4a}	ν_{68}	1470	N_5-C_{4a}, N_{10a}-N_1, C_{5a}-N_5, C_{11}-H_3, N_3-H, C_4-N_3, C_{9a}-C_{5a}
1498	ν_{71}	1547	(ν_{70}, ν_{72}) C_7-C_6, C_{9a}-N_{10}, C_8-C_9, C_{10a}-N_1, $C_{7a,11}$-H_3, N_5-C_{4a}, C_4-N_3	ν_{70}	1530	(ν_{72}) C_8-C_7, C_{9a}-C_{5a}, $C_{6,9}$-H, C_6-C_{5a}, C_9-C_{9a}, C_{7a}-H_3, N_5-C_{4a}
1570	ν_{73}	1650	C_8-C_9, C_6-C_{5a}, $C_{6,9}$-H, C_7-C_6, C_{9a}-N_{10}, C_{10a}-N_1, N_5-C_{4a}	ν_{73}	1641	C_8-C_9, C_6-C_{5a}, $C_{6,9}$-H, C_7-C_6, C_{9a}-N_{10}, C_{10a}-N_1, N_5-C_{4a}
	ν_{74}	1757	C_2-$O_{2'}$, C_4-$O_{4'}$, N_3-H (as)	ν_{74}	1698	C_2-$O_{2'}$, C_4-$O_{4'}$, N_3-H (as)
1626	ν_{75}	1777	C_4-$O_{4'}$, C_2-$O_{2'}$, N_{10}-C_{10a} (s)	ν_{75}	1722	C_4-$O_{4'}$, C_2-$O_{2'}$, N_{10}-C_{10a} (s)
Exp. RR FMN S_1	mPW1PW91/cc-pVDZ			mPWLYP/cc-pVDZ		
	$\nu_\#$	S_1offR	Assignment	$\nu_\#$	S_1offR	Assignment
1200	ν_{49}	1209	$C_{6,9}$-H, N_3-H, C_{5a}-N_5, C_7-C_6, C_4-N_3	ν_{49}	1125	C_6-H, $C_{7a,8a}$-H_3, C_8-C_7, C_{5a}-N_5, N_{10}-C_{10a}
	ν_{50}	1222	$C_{6,9}$-H, C_{11}-H_3, C_4-N_3, N_{10}-C_{10a}, C_{10a}-N_1, N_5-C_{4a}, C_9-C_{9a}	ν_{50}	1137	$C_{6,9}$-H, C_{11}-H_3, C_4-N_3, N_3-H, C_{5a}-N_5
1250	ν_{51}	1262	C_2-N_3, N_3-H, C_{4a}-C_4, C_9-H, N_1-C_2, C_7-C_6, C_{5a}-N_5	ν_{55}	1282	N_{10}-C_{10a}, C_{11}-H_3, N_3-H, C_{9a}-C_{5a}, C_7-C_6, C_{10a}-N_1, C_{4a}-C_4, C_{10a}-C_{4a}
1338	-	-	-	-	-	-
	ν_{56}	1377	C_{7a}-H_3, N_3-H, C_{5a}-N_5, C_{10a}-N_1	ν_{60}	1341	N_3-H, $C_{8a,7a}$-H_3, C_6-H, C_{9a}-C_{5a}, C_8-C_7, C_2-N_3
1381	ν_{58}	1396	N_3-H, $C_{11,7a}$-H_3, C_6-H, C_7-C_6, C_{9a}-N_{10}, C_{10a}-C_{4a}	-	-	-
	ν_{59}	1408	$C_{8a,11}$-H_3, C_8-C_7, N_3-H, C_{9a}-C_{5a}, C_4-N_3, C_6-C_{5a}	ν_{62}	1365	C_{11}-H_3, C_{4a}-C_4, N_1-C_2, C_4-N_3, C_{8a}-H_3
	ν_{61}	1428	C_{11}-H_3, N_3-H, N_1-C_2, C_{9a}-N_{10}, C_{10a}-C_{4a}, C_{5a}-N_5	ν_{63}	1383	$C_{7a,11}$-H_3, N_5-C_{4a}, C_{10a}-C_{4a}, C_7-C_6
	ν_{62}	1432	N_3-H, C_6-H, C_4-N_3, C_{9a}-C_{5a}, C_{11}-H_3, C_{10a}-C_{4a}	-	-	-
1416	ν_{65}	1456	C_{8a}-H_3, C_9-H, N_1-C_2, C_{10a}-C_{4a}	ν_{67}	1415	$C_{7a,11,8a}$-H_3, C_{9a}-C_{5a}, C_{10a}-C_{4a}
	-	-	-	-	-	-
1498	ν_{71}	1549	C_{9a}-N_{10}, C_{10a}-N_1, C_{11}-H_3, C_{5a}-N_5, C_8-C_9, $C_{7a,8a}$-H_3	ν_{71}	1474	C_8-C_7, $C_{6,9}$-H, C_9-C_{9a}, C_6-C_{5a}, C_{9a}-C_{5a}, $C_{7a,8a}$-H_3
1570	ν_{73}	1661	C_8-C_9, C_6-C_{5a}, $C_{9,6}$-H, C_9-C_{9a}, C_7-C_6, C_{10a}-N_1, N_5-C_{4a}	ν_{73}	1553	C_2-$O_{2'}$, C_8-C_9, C_6-C_{5a}, $C_{9,6}$-H, N_3-H, C_7-C_6, C_9-C_{9a}

	ν_{74}	1736	C_2-$O_{2'}$, N_3-H, C_4-$O_{4'}$, C_4-N_3 (as)	ν_{74}	1568	C_2-$O_{2'}$, N_3-H, C_8-C_9, C_6-C_{5a}, N_{10}-C_{10a}, C_{4a}-C_4
1626	ν_{75}	1758	C_4-$O_{4'}$, C_2-$O_{2'}$, N_3-H (s)	ν_{75}	1644	C_4-$O_{4'}$, N_3-H, C_{4a}-C_4
**Exp. RR FMN S_1**	**O3LYP/cc-pVDZ**			**OLYP/cc-pVDZ**		
	**ν#**	**S_1offR**	**Assignment**	**ν#**	**S_1offR**	**Assignment**
1200	ν_{49}	1183	C_6-H, N_5-C_{5a}, C_6-C_7, N_1-C_{10a}	ν_{48}	1146	C_6-H, N_1-C_{10a}, C_7-C_6, N_5-C_{4a}, N_3-C_4, $C_{7a,8a}$-H_3
	-	-	-	ν_{50}	1175	N_3-H, $C_{6,9}$-H, C_{11}-H_3, N_3-C_4, N_1-C_2
1250	ν_{51}	1228	C_2-N_3, C_{4a}-C_4, N_3-H, $C_{6,9}$-H, C_9-C_{9a}	ν_{51}	1215	N_3-H, N_3-C_2, C_{10a}-C_{4a}, C_{4a}-C_4, $C_{6,9}$-H
1338	ν_{53}	1290	C_{11}-H_3, $C_{6,9}$-H, N_{10}-C_{10a}, N_5-C_{4a}, C_8-C_7	ν_{53}	1270	$C_{6,9}$-H, N_3-H, C_{11}-H_3, N_1-C_{10a}, N_5-C_{4a}, N_3-C_2
	-	-	-	-	-	-
1381	ν_{57}	1373	C_{11}-H_3, N_3-H, C_6-H, C_{7a}-H_3, C_7-C_6, C_{10a}-C_{4a}, C_{9a}-N_{10}	ν_{57}	-	-
	ν_{58}	1376	C_{8a}-H_3, C_8-C_7, C_{9a}-C_{5a}, N_3-H	ν_{58}	-	-
	ν_{59}	1385	C_{8a}-H_3, N_3-H, N_6-C_{5a}, N_3-C_4, N_1-C_2, C_8-C_9	ν_{59}	-	-
	ν_{61}	1402	N_3-H, C_6-H, C_{9a}-C_{5a}, N_3-C_4, C_{10a}-C_{4a}	ν_{61}	1387	N_3-H, $C_{6,9}$-H, C_{9a}-C_{5a}, C_8-C_7, $C_{7a,8a}$-H_3
1416	ν_{64}	1437	C_{8a}-H_3, C_9-H, C_{11}-H_3, C_4-C_{4a}, N_1-C_2	ν_{64}	1419	C_{7a}-H_3, C_{11}-H_3, C_{8a}-H_3, C_9-H
	-	-	-	ν_{66}	1420	C_{7a}-H_3, N_5-C_{4a}, C_{8a}-H_3
1498	ν_{71}	1526	$C_{6,9}$-H, C_8-C_7, C_{5a}-C_{9a}, C_{10a}-C_{4a}, C_{7a}-H_3	ν_{71}	1519	$C_{6,9}$-H, C_9-C_{9a}, C_6-C_{5a}, C_8-C_7
1570	ν_{73}	1628	$C_{6,9}$-H, C_8-C_9, C_{5a}-C_6, C_{10a}-N_1	ν_{73}	1605	C_8-C_9, C_6-C_{5a}, $C_{6,9}$-H, C_2-$O_{2'}$, N_3-H, N_1-C_{10a}
	ν_{74}	1684	C_2-$O_{2'}$, N_3-H, C_4-C_{4a} ~(as)	ν_{74}	1624	C_2-$O_{2'}$, N_3-H, N_{10}-C_{10a}, C_8-C_9, C_6-C_{5a}, C_{4a}-C_4 ~(as)
1626	ν_{75}	1725	C_2-$O_{2'}$, N_3-H, C_4-$O_{4'}$ ~(s)	ν_{75}	1697	C_4-$O_{4'}$, N_3-H ~(s)
**Exp. RR FMN S_1**	**OPBE/cc-pVDZ**			**PBE1PBE/cc-pVDZ**		
	**ν#**	**S_1offR**	**Assignment**	**ν#**	**S_1offR**	**Assignment**
1200	ν_{49}	1167	C_6-H, $C_{7a,8a}$-H_3, N_1-C_{10a}, C_7-C_6, N_5-C_{4a}, N_5-C_{5a}	ν_{49}	1205	$C_{6,9}$-H, N_3-H, C_{5a}-N_5, C_4-N_3, C_8-C_7
	-	-	-	ν_{50}	1219	$C_{6,9}$-H, C_{11}-H_3, C_9-C_{9a}, C_{4a}-C_4, C_4-N_3, C_{10a}-N_1
1250	ν_{51}	1237	$C_{6,9}$-H, N_3-H, N_3-C_2, N_5-C_{4a}, C_4-C_{4a}, N_5-C_{5a}	ν_{51}	1262	N_3-H, C_4-N_3. C_9-H, C_{4a}-C_4, N_1-C_2, C_{9a}-C_{5a}
1338	ν_{53}	1282	C_6-H, N_3-H, C_{11}-H_3, $C_{7a,8a}$-H_3, N_1-C_{10a}, N_5-C_{4a}, C_6-C_{5a}	ν_{53}	1322	N_{10}-C_{10a}, C_6-C_{5a}, C_{11}-H_3, N_5-C_{4a}, C_8-C_7, C_2-N_3, C_{8a}-H_3
	-	-	-	ν_{56}	1373	N_3-H, C_{5a}-N_5, C_{10a}-N_1, C_{7a}-H_3, C_{11}-H_3
1381	-	-	-	-	-	-
	ν_{58}	1359	C_{11}-H_3, N_3-H, C_{8a}-H_3, C_6-H, N_{10}-C_{9a}	ν_{58}	1392	N_3-H, C_{11}-H_3, C_6-H, C_{7a}-H_3, C_7-C_6
	ν_{62}	1394	$C_{7a,8a}$-H_3, C_{11}-H_3, C_6-H, N_3-C_4, N_3-H, C_{9a}-C_{5a}, C_{10a}-C_{4a}	ν_{59}	1404	C_{11}-H_3, C_{8a}-H_3, C_8-C_7, N_3-H, C_6-C_{5a}
	ν_{63}	1408	$C_{7a,8a}$-H_3, C_{11}-H_3, C_8-C_7, C_{9a}-C_{5a}, C_{10a}-C_{4a}, C_6-H	-	-	-
1416	-	-	-	-	-	-
	ν_{66}	1422	C_{7a}-H_3, C_{11}-H_3, C_{9a}-C_{5a}, C_{10a}-C_{4a}, N_1-C_2	ν_{65}	1451	C_{8a}-H_3, C_9-H, N_1-C_2, C_{11}-H_3
1498	ν_{71}	1535	$C_{6,9}$-H, C_{9a}-C_{9a}, C_6-C_{5a}, C_8-C_7	ν_{71}	1548	C_8-C_7, C_{9a}-C_{5a}, $C_{6,9}$-H, C_{7a}-H_3
1570	ν_{73}	1626	C_8-C_9, C_6-C_{5a}, $C_{6,9}$-H, C_2-$O_{2'}$, N_3-H, C_{10a}-N_1	ν_{73}	1659	C_8-C_9, C_6-C_{5a}, $C_{6,9}$-H, N_5-C_{4a}
	ν_{74}	1652	C_2-$O_{2'}$, N_3-H, N_{10}-C_{10a}, C_8-C_9, C_6-C_{5a}, C_{4a}-C_4 ~(as)	ν_{74}	1738	C_2-$O_{2'}$, N_3-H, C_4-$O_{4'}$ (as)
1626	ν_{75}	1725	C_4-$O_{4'}$, N_3-H ~(s)	ν_{75}	1759	C_4-$O_{4'}$, C_2-$O_{2'}$, N_3-H (s)

Exp. RR FMN S_1	PW6B95D3/cc-pVDZ			revTPSSh/aug-cc-pVDZ		
	$\nu_\#$	S_1offR	Assignment	$\nu_\#$	S_1offR	Assignment
1200	ν_{49}	1208	C_6-H, N_3-H, C_8-C_7, C_{5a}-N_5, C_7-C_6, C_{5a}-N_5, C_4-N_3	ν_{51}	1354	C_2-N_3, C_{10a}-C_{4a}, N_3-H, $C_{6,9}$-H, C_4-N_3, C_{9a}-C_{5a}
	ν_{50}	1222	$C_{6,9}$-H, C_{4a}-C_4, N_5-C_{4a}, N_{10}-C_{10a}, C_{11}-H_3, C_9-C_{9a}, C_{11}-H_3, C_{10a}-N_1	-	-	-
1250	ν_{51}	1257	C_2-N_3, N_3-H, C_{4a}-C_4, C_{5a}-N_5, C_9-H, N_1-C_2, C_{9a}-C_{5a}, C_7-C_6	ν_{52}	1382	C_9-H, C_8-C_7, C_{5a}-N_5, C_6-H, C_{7a}-H_3, C_{10a}-C_{4a}
1338	ν_{54}	1339	C_{5a}-N_5, C_9-C_{9a}, N_{10}-C_{10a}, $C_{6,9}$-H, C_8-C_7, C_2-N_3	-	-	-
	ν_{56}	1378	C_{7a}-H_3, N_3-H, C_{10a}-N_1, C_{5a}-N_5, C_8-C_7	ν_{56}	1491	N_3-H, C_6-H, C_6-C_{5a}, C_{9a}-N_{10}, C_7-C_6, C_8-C_9, C_{10a}-C_{4a}, C_4-N_3
1381	ν_{58}	1396	N_3-H, $C_{11,7a}$-H_3, C_6-H, C_7-C_6, C_{9a}-N_{10}, C_{5a}-N_5, C_{10a}-C_{4a}	ν_{57}		
	ν_{59}	1405	$C_{11,7a,8a}$-H_3, C_8-C_7, C_{9a}-C_{5a}, N_3-H, C_6-C_{5a}	ν_{58}	1516	$C_{7a,8a}$-H_3, C_8-C_7, C_{5a}-C_6, C_2-N_3, N_3-C_4
	ν_{60}	1425	$C_{11,7a,8a}$-H_3, C_{9a}-N_{10}, N_5-C_{4a}, C_{10a}-N_1, C_8-C_7, N_3-H	ν_{59}	1522	N_3-H, C_6-H, C_{9a}-C_{5a}, C_{4a}-C_{10a}, C_9-C_{9a}, C_{10a}-C_{4a}, C_4-N_3
	ν_{62}	1432	N_3-H, C_{9a}-C_{5a}, $C_{7a,11}$-H_3, C_6-H, C_{10a}-N_1, C_7-C_6, C_4-N_3	ν_{60}	1526	C_{7a}-H_3, C_{9a}-C_{5a}, C_{10a}-C_{4a}, C_2-N_3, N_3-H
1416	ν_{65}	1455	$C_{8a,11}$-H_3, C_2-N_1, C_{10a}-C_4	ν_{63}	1574	N_5-C_{4a}, C_{10a}-C_{4a}, C_4-N_3, C_{10a}-N_1, $C_{11,7a}$-H_3, C_8-C_7
	ν_{68}	1475	N_5-C_{4a}, C_{11}-H_3, C_{10a}-C_{4a}, C_7-C_6, C_2-N_3, C_{7a}-H_3	-	-	-
1498	ν_{71}	1549	C_8-C_7, C_9-C_{9a}, C_{9a}-C_{5a}, C_6-C_{5a}, $C_{6,9}$-H, C_{7a}-H_3, C_{10a}-C_{4a}	ν_{71}	1653	C_8-C_7, C_{9a}-C_{5a}, $C_{6,9}$-H, C_{7a}-H_3, C_6-C_{5a}, C_9-C_{9a}, C_{10a}-C_{4a}
1570	ν_{73}	1659	C_8-C_9, C_6-C_{5a}, $C_{6,9}$-H, C_7-C_6, N_{10}-C_{9a}, N_5-C_{4a}, C_{10a}-N_1	ν_{73}	1770	C_8-C_9, C_6-C_{5a}, $C_{6,9}$-H, C_7-C_6, C_9-C_{9a}, C_2-$O_{2'}$, C_4-$O_{4'}$, C_{10a}-N_1 (as)
	ν_{74}	1737	C_2-$O_{2'}$, N_3-H, C_4-$O_{4'}$ (as), C_{4a}-C_{10a}	ν_{74}	1797	C_2-$O_{2'}$, N_3-H, C_2-N_3, C_6-C_{5a}, C_8-C_9
1626	ν_{75}	1757	C_4-$O_{4'}$, C_2-$O_{2'}$ (s), N_3-H, C_{4a}-C_{10a}	ν_{75}	1817	C_4-$O_{4'}$, N_3-H, C_{4a}-C_4, C_6-C_{5a}, C_2-$O_{2'}$ (s), C_8-C_9

Exp. RR FMN S_1	revTPSS/aug-cc-pVDZ			SOGGA11/cc-pVDZ		
	$\nu_\#$	S_1offR	Assignment	$\nu_\#$	S_1offR	Assignment
1200	ν_{51}	1199	C_2-N_3, C_{10}-C_{4a}, N_3-H, $C_{6,9}$-H, C_9-C_{9a}, C_{9a}-C_{5a}, N_5-C_4, N_1-C_2	ν_{49}	1229	$C_{6,9}$-H, N_3-H, C_{5a}-N_5, C_4-N_3, C_7-C_6, C_8-C_9
	-	-	-	ν_{50}	1240	$C_{6,9}$-H, C_{11}-H_3, C_{4a}-C_4, C_9-C_{9a}, N_{10}-C_{10a}
1250	ν_{53}	1265	C_6-H, C_{11}-H_3, C_{10a}-C_{4a}, C_{9a}-N_{10}, N_3-H, C_{9a}-C_{5a}, C_8-C_7	ν_{51}	1286	N_3-H, C_6-H, C_2-N_3, C_{4a}-C_4, N_1-C_2, N_5-C_{4a}, C_{5a}-C_{9a}
1338	-	-	-	ν_{53}	1340	N_{10}-C_{10a}, N_5-C_{4a}, C_{9a}-C_{5a}, C_8-C_7, N_5-C_{4a}, C_{11}-H_3, C_2-N_3
	ν_{56}	1340	N_3-H, C_6-H, C_{11}-H_3, C_{9a}-N_{10}, C_7-C_6, C_8-C_9, C_6-C_{5a}, N_1-C_2	ν_{54}	1361	C_{5a}-N_5, C_{10a}-C_{4a}, N_3-H, C_9-C_{9a}, N_{10}-C_{10a}, C_2-N_3, $C_{6,9}$-H, C_{7a}-H_3
1381	ν_{57}	1356	N_3-H, C_{10a}-N_1, C_6-H, N_{11}-H_3, N_1-C_2, C_{9a}-C_{5a}, N_5-C_{4a}, C_{9a}-N_{10}	ν_{57}	1414	$C_{11,7a}$-H_3, C_{9a}-N_{10}, C_6-C_{5a}, C_{10a}-N_1, C_7-C_6
	ν_{58}	1366	N_3-H, C_{10a}-N_{10}, N_5-C_{4a}, C_{10a}-N_1, C_7-C_6, C_6-H, C_8-C_7, $C_{7a}H_3$	ν_{58}	1422	$C_{11,7a,8a}$-H_3, C_8-C_7, C_6-C_{5a}, C_{9a}-C_{5a}, N_3-H
	ν_{59}	1371	N_3-H, C_6-H, C_9-C_{9a}, C_{9a}-C_{5a}, C_4-N_3, C_8-C_7, C_{10a}-C_{4a}	ν_{59}	1427	N_3-H, C_4-N_3, C_{10a}-C_{4a}, C_6-H, C_{7a}-H_3, C_{9a}-C_{5a}
	ν_{60}	1377	$C_{7a,8a}$-H_3, C_8-C_7, C_{9a}-C_{5a}, N_5-C_{4a}	ν_{60}	1445	$C_{11,7a,8a}$-H_3, N_{10}-C_{10a}, C_{10a}-C_{4a}, N_5-C_{5a}
1416	ν_{63}	1420	$C_{11,7a}$-H_3, C_7-C_6, C_{5a}-N_5, C_9-C_{9a}, N_{10}-C_{10a}, N_5-C_{4a}	ν_{65}	1477	$C_{7a,11}$-H_3, N_1-C_2, C_{10a}-C_{4a}, C_6-C_{5a}
	-	-	-	ν_{68}	1498	N_5-C_{4a}, C_{10a}-N_1, C_{9a}-N_{10}, C_{11}-H_3, N_3-H, C_2-N_3, $C_{7a,8a}$-H_3

1498	ν_{71}	1506	C_8-C_7, C_{9a}-C_{5a}, $C_{6,9}$-H, C_{7a}-H_3, C_6-C_{5a}, C_9-C_{9a}, C_{10a}-C_{4a}	ν_{70}	1561	C_8-C_7, C_9-C_{9a}, C_{9a}-C_{5a}, C_6-C_{5a}, C_{10a}-N_1, $C_{6,9}$-H, C_{7a}-H_3
1570	ν_{73}	1569	C_2-$O_{2'}$, N_3-H, C_2-N_3, C_6-C_{5a}, C_8-C_9	ν_{73}	1679	C_8-C_9, C_6-C_{5a}, $C_{6,9}$-H, C_7-C_6, N_{10}-C_{9a}, N_5-$C_{4a,}$ C_{10a}-N_1
	ν_{74}	1594	(s) C_8-C_9, C_6-C_{5a}, $C_{6,9}$-H, C_7-C_6, C_9-C_{9a}, C_2-$O_{2'}$, C_4-$O_{4',}$ C_{10a}-N_1	ν_{74}	1777	C_2-$O_{2'}$, N_3-H, C_4-$O_{4'}$ (as), C_{4a}-C_{10a}
1626	ν_{75}	1627	C_4-$O_{4'}$, N_3-H, C_{4a}-C_4, C_6-C_{5a}, C_8-C_9	ν_{75}	1794	C_4-$O_{4'}$, C_2-$O_{2'}$ (s), C_{4a}-C_{10a}
**Exp. RR FMN S_1**	**SOGGA11x/cc-pVDZ**			**tHCTHhyb/cc-pVDZ**		
	**ν#**	**S_1offR**	**Assignment**	**ν#**	**S_1offR**	**Assignment**
1200	ν_{49}	1229	$C_{6,9}$-H, N_3-H, C_{5a}-N_5, C_4-N_3, C_7-C_6, C_8-C_9	ν_{49}	1177	C_6-H, C_{5a}-N_5, C_{11}-H_3, N_3-H, C_8-C_7
	ν_{50}	1240	$C_{6,9}$-H, C_{11}-H_3, C_{4a}-C_4, C_9-C_{9a}, N_{10}-C_{10a}	ν_{50}	1187	C_9-H, C_4-N_3, N_3-H, C_{11}-H_3, C_{4a}-C_4, C_{10a}-N_1, C_{9a}-N_{10}
1250	ν_{51}	1286	N_3-H, C_6-H, C_2-N_3, C_{4a}-C_4, N_1-C_2, N_5-C_{4a}, C_{5a}-C_{9a}	ν_{51}	1220	C_2-N_3, N_3-H, C_9-H, C_{4a}-C_4, N_5-C_{4a}, C_{9a}-C_{5a}
1338	ν_{53}	1340	N_{10}-C_{10a}, N_5-C_{4a}, C_{9a}-C_{5a}, C_8-C_7, N_5-C_{4a}, C_{11}-H_3, C_2-N_3	-	-	-
	ν_{54}	1361	C_{5a}-N_5, C_{10a}-C_{4a}, N_3-H, C_9-C_{9a}, N_{10}-C_{10a}, C_2-N_3, $C_{6,9}$-H, C_{7a}-H_3	ν_{56}	1355	C_{7a}-H_3, C_8-C_7, C_9-H, C_{11}-H_3
1381	ν_{57}	1414	$C_{11,7a}$-H_3, C_{9a}-N_{10}, C_6-C_{5a}, C_{10a}-N_1, C_7-C_6	ν_{57}	1367	N_3-H, C_6-H, $C_{11,7a}$-H_3, C_6-C_{5a}, C_{9a}-C_{5a}, C_{9a}-N_{10}, C_{10a}-C_{4a}
	ν_{58}	1422	$C_{11,7a,8a}$-H_3, C_8-C_7, C_6-C_{5a}, C_{9a}-C_{5a}, N_3-H	ν_{58}	1371	C_{8a}-H_3, C_8-C_9, C_{9a}-C_{5a}
	ν_{59}	1427	N_3-H, C_4-N_3, C_{10a}-C_{4a}, C_6-H, C_{7a}-H_3, C_{9a}-C_{5a}	ν_{59}	1379	C_{8a}-H_3, N_3-H, C_{9a}-C_{5a}, C_6-C_{5a}, C_8-C_7, N_1-C_2
	ν_{60}	1445	$C_{11,7a,8a}$-H_3, N_{10}-C_{10a}, C_{10a}-C_{4a}, N_5-C_{5a}	ν_{61}	1394	N_3-H, N_3-C_4, C_6-H, C_{9a}-C_{5a}, C_{10a}-C_{4a}
1416	ν_{65}	1477	$C_{7a,11}$-H_3, N_1-C_2, C_{10a}-C_{4a}, C_6-C_{5a}	ν_{64}	1432	N_5-C_{4a}, C_{7a}-H_3, C_{10a}-C_{4a}, C_{10a}-N_1
	ν_{68}	1498	N_5-C_{4a}, C_{10a}-N_1, C_{9a}-N_{10}, C_{11}-H_3, N_3-H, C_2-N_3, $C_{7a,8a}$-H_3	ν_{65}	1433	$C_{8a,11,7a}$-H_3, C_9-H, N_{10}-C_{10a}, N_1-C_2, C_4-N_3, N_3-H
1498	ν_{70}	1561	C_8-C_7, C_9-C_{9a}, C_{9a}-C_{5a}, C_6-C_{5a}, C_{10a}-N_1, $C_{6,9}$-H, C_{7a}-H_3	ν_{71}	1515	C_8-C_7, C_{9a}-C_{5a}, C_9-H, C_{7a}-H_3, C_9-C_{9a}, C_6-C_{5a}, C_{10a}-C_{4a}
1570	ν_{73}	1679	C_8-C_9, C_6-C_{5a}, $C_{6,9}$-H, C_7-C_6, N_{10}-C_{9a}, N_5-$C_{4a,}$ C_{10a}-N_1	ν_{73}	1618	C_8-C_9, C_6-C_{5a}, $C_{6,9}$-H, C_9-C_{9a}, C_7-C_6, C_{10a}-N_1, C_{4a}-N_5
	ν_{74}	1777	C_2-$O_{2'}$, N_3-H, C_4-$O_{4'}$ (as), C_{4a}-C_{10a}	ν_{74}	1687	C_2-$O_{2'}$, N_3-H, C_{10a}-N_1, N_3-C_4
1626	ν_{75}	1794	C_4-$O_{4'}$, C_2-$O_{2'}$ (s), C_{4a}-C_{10a}	ν_{75}	1721	C_4-$O_{4'}$, N_3-H, C_{4a}-C_4, C_2-$O_{2'}$ (s)
**Exp. RR FMN S_1**	**TPSSh/cc-pVDZ**			**TPSSTPSS/cc-pVDZ**		
	**ν#**	**S_1offR**	**Assignment**	**ν#**	**S_1offR**	**Assignment**
1200	ν_{49}	1173	C_6-H, C_{10a}-N_1, C_4-N_3, C_{5a}-N_5, C_7-C_6, N_5-C_{4a}	ν_{51}	1221	C_9-H, C_2-N_3, C_{10a}-C_{4a}, C_4-N_3, C_6-C_{5a}, C_9-C_{9a}
	ν_{50}	1184	$C_{6,9}$-H, C_{11}-H_3, C_4-N_3, N_3-H, C_8-C_7, C_{10a}-C_{4a}	ν_{52}	1246	C_2-$O_{2'}$, N_3-H, $C_{6,9}$-H, C_9-N_{10}, C_8-C_9, N_1-C_2
1250	ν_{51}	1220	C_2-N_3, C_{10a}-C_{4a}, N_3-H, $C_{6,9}$-H, C_4-N_3, C_{9a}-C_{5a}	ν_{54}	1276	N_3-H, C_2-$O_{2'}$, $C_{6,9}$-H, C_{4a}-C_4, N_{10}-C_{10a}, C_{5a}-C_{9a}, C_{11}-H_3
1338	-	-	-	-	-	-
	ν_{56}	1369	N_3-H, C_6-H, C_{11}-H_3, C_{9a}-N_{10}, C_7-C_6, C_8-C_9, C_{10a}-C_{4a}, C_4-N_3	ν_{56}	1330	C_{5a}-N_5, C_9-C_{9a}, C_8-C_7, N_3-H, C_9-H, N_{10}-C_{10a}
1381	ν_{57}	1375	N_3-H, C_{7a}-H_3, C_8-C_7, C_{9a}-C_{5a}, C_4-N_3, $C_{6,9}$-H	ν_{57}	1353	N_3-H, C_{10a}-N_1, C_6-H, N_{11}-H_3, N_1-C_2, C_{9a}-C_{5a}, N_5-C_{4a}, C_{9a}-N_{10}
	ν_{58}	1383	$C_{7a,11}$-H_3, C_{10a}-N_1, C_{9a}-N_{10}, C_2-N_3, N_5-C_{4a}	ν_{58}	1367	C_8-C_9, C_6-C_{5a}, C_{9a}-C_{5a}, C_{11}-H_3, N_3-H, C_{10a}-C_{4a}

	ν_{60}	1396	N_3-H, C_{8a}-H_3, C_6-H, C_{9a}-C_{5a}, N_4-C_{4a}, C_4-N_3	-	-	-
	ν_{61}	1401	C_{8a}-H_3, C_{9a}-C_{5a}, N_3-H, C_6-H	ν_{61}	1412	N_3-H, C_2-N_3, C_{11}-H_3, N_5-C_{4a}
1416	ν_{63}	1435	$C_{7a,11}$-H_3, N_5-C_{4a}, C_8-C_7, C_{10a}-C_{4a}, C_4-N_3	-	-	-
	-	-	-	ν_{64}	1446	N_3-H, $C_{7a,8a,11}$-H_3, C_7-C_6, C_8-C_9, C_{10a}-C_{4a}, C_2-N_3
1498	ν_{71}	1526	C_8-C_7, C_{9a}-C_{5a}, $C_{9,6}$-H, C_9-C_{9a}, C_6-C_{5a}, $C_{7a,8a}$-H_3	ν_{71}	1518	C_8-C_7, C_9-C_{9a}, C_6-C_{5a}, $C_{6,9}$-H, N_{10}-C_{10a}, N_5-C_{4a}, C_{10a}-N_1, C_{11}-H_3
1570	ν_{73}	1621	$C_{6,9}$-H, C_8-C_9, C_6-C_{5a}, C_7-C_6, C_9-C_{9a}, C_{10a}-N_1, C_2-$O_{2'}$	ν_{73}	1582	C_2-$O_{2'}$, N_1-C_2, N_3-H, C_2-N_3, C_4-$O_{4'}$, C_6-C_{5a}, C_8-C_9
	ν_{74}	1658	C_2-$O_{2'}$, N_3-H, C_{10a}-N_1	ν_{74}	1614	C_8-C_9, C_6-C_{5a}, $C_{6,9}$-H, C_7-C_6, C_9-C_{9a}, C_{10a}-N_1 (as)
1626	ν_{75}	1706	C_4-$O_{4'}$, N_3-H, C_{4a}-C_4, C_2-$O_{2'}$ (s)	ν_{75}	1695	C_4-$O_{4'}$, C_{4a}-C_4, C_2-$O_{2'}$ (s), N_1-C_2, N_3-H
**Exp. RR FMN S_1**	**TPSSTPSS/aug-cc-pVDZ**			**VSXC/cc-pVDZ**		
	**ν#**	**S_1offR**	**Assignment**	**ν#**	**S_1offR**	**Assignment**
1200	ν_{51}	1191	C_2-N_3, C_{10}-C_{4a}, N_3-H, $C_{6,9}$-H, C_9-C_{9a}, C_{9a}-C_{5a}, N_5-C_4, N_1-C_2	ν_{51}	1244	C_9-H, C_{4a}-C_4, N_3-H, C_6-C_{5a}, C_2-N_3, C_{9a}-C_{5a}, C_{11}-H_3
	-	-	-	ν_{52}	1259	N_3-H, C_2-$O_{2'}$, $C_{6,9}$-H, C_{9a}-N_{10}, C_{10a}-N_1
1250	ν_{53}	1261	C_6-H, C_{11}-H_3, C_{10a}-C_{4a}, C_{9a}-N_{10}, N_3-H, C_{9a}-C_{5a}, C_8-C_7	ν_{53}	1282	N_5-C_{4a}, $C_{6,9}$-H, C_{9a}-C_{5a}, C_{10a}-N_1, C_8-C_7
1338	-	-	-	ν_{55}	1328	C_{10a}-N_1, C_{9a}-N_{10}, C_{4a}-C_4, C_{5a}-N_5, C_8-C_7, C_{11}-H_3, N_3-H
	ν_{56}	1336	C_6-H, N_3-H, C_{11}-H_3, C_{9a}-N_{10}, C_7-C_6, C_8-C_9, C_6-C_{5a}, N_1-C_2	ν_{56}	1342	C_{5a}-N_5, N_3-H, C_9-H, C_{7a}-H_3, C_9-C_{9a}, C_{4a}-C_4, C_8-C_7
1381	ν_{57}	1351	N_3-H, C_{10a}-N_1, C_6-H, N_{11}-H_3, N_1-C_2, C_{9a}-C_{5a}, N_5-C_{4a}, C_{9a}-N_{10}	-	-	-
	ν_{59}	1367	N_3-H, C_6-H, C_9-C_{9a}, C_{9a}-C_{5a}, C_4-N_3, C_8-C_7, C_{10a}-C_{4a}	ν_{58}	1361	C_{9a}-C_{5a}, N_{10}-C_{10a}, $C_{11,7a,8a}$-H_3, N_5-C_{4a}, N_3-H
	ν_{60}	1371	$C_{7a,8a}$-H_3, C_8-C_7, C_{9a}-C_{5a}, N_5-C_{4a}	ν_{59}	1366	$C_{11,7a,8a}$-H_3, N_3-H, C_{10a}-C_{4a}, C_{9a}-C_{5a}
	-	-	-	ν_{63}	1417	$C_{11,7a}$-H_3, C_{9a}-C_{5a}, N_3-H, C_{10a}-N_1
1416	ν_{63}	1414	$C_{11,7a}$-H_3, C_7-C_6, C_{5a}-N_5, C_9-C_{9a}, N_{10}-C_{10a}, N_5-C_{4a}	ν_{66}	1442	$C_{11,7a,8a}$-H_3, N_3-H, C_2-N_3, N_{10}-C_{10a}
	-	-	-	ν_{68}	1479	N_3-H, C_2-N_3, C_{10a}-C_{4a}, $C_{6,9}$-H, C_8-C_9, C_{11}-H_3
1498	ν_{71}	1501	C_8-C_7, C_{9a}-C_{5a}, $C_{6,9}$-H, C_{7a}-H_3, C_6-C_{5a}, C_9-C_{9a}, C_{10a}-C_{4a}	ν_{71}	1536	C_8-C_7, C_9-C_{9a}, C_{9a}-C_{5a}, C_6-C_{5a}, C_{10a}-N_1, $C_{6,9}$-H, C_{7a}-H_3
1570	ν_{73}	1566	C_4-$O_{4'}$, N_3-H, C_2-N_3, C_6-C_{5a}, C_8-C_9	ν_{73}	1612	C_8-C_9, C_6-C_{5a}, $C_{6,9}$-H, C_7-C_6, N_{10}-C_{9a}, N_5-C_{4a}, C_{10a}-N_1
	ν_{74}	1590	(s) C_8-C_9, C_6-C_{5a}, $C_{6,9}$-H, C_7-C_6, C_9-C_{9a}, C_2-$O_{2'}$, C_4-$O_{4'}$, C_{10a}-N_1	ν_{74}	1635	C_2-$O_{2'}$, N_3-H, C_4-$O_{4'}$ (as), C_{4a}-C_{10a}
1626	ν_{75}	1623	C_2-$O_{2'}$, N_3-H, C_{4a}-C_4, C_6-C_{5a}, C_8-C_9	ν_{75}	1725	C_4-$O_{4'}$, C_2-$O_{2'}$ (s), C_{4a}-C_{10a}, C_{10a}-N_1
**Exp. RR FMN S_1**	**VSXC/aug-cc-pVDZ**			**wB97XD/cc-pVDZ**		
	**ν#**	**S_1offR**	**Assignment**	**ν#**	**S_1offR**	**Assignment**
1200	ν_{49}	1150	C_6-H, C_{11}-H_3, C_{5a}-N_5, C_{10a}-N_1, C_7-C_6, C_{4a}-C_4	ν_{50}	1232	C_6-H, C_{11}-H_3, C_9-C_{9a}, C_{10a}-C_{4a}, C_{4a}-C_4
	ν_{51}	1204	C_2-N_3, N_3-H, C_{10a}-C_{4a}, $C_{6,9}$-H, C_{9a}-C_{5a}, N_5-C_{4a}	-	-	-

1250	ν_{53}	1271	C_9-H, C_{11}-H_3, N_3-H, C_{10a}-N_1, C_{9a}-C_{5a}, C_{10a}-C_{4a}, N_5-C_{4a}	ν_{53}	1319	C_{11}-H_3, N_{10}-C_{10a}, C_{9a}-C_{5a}, N_5-C_{4a}, C_8-C_7, C_4-N_3, C_9-C_{9a}
1338	-	-	-	ν_{54}	1344	C_{5a}-N_1, C_9-C_{9a}, C_{10a}-C_{4a}, N_{10}-C_{10a}, N_3-H, C_{7a}-H_3, N_3-C_2
	ν_{56}	1344	N_3-H, $C_{7a,11}$-H_3, C_6-C_{5a}, C_6-H, C_{9a}-N_{10}, C_2-N_3	-	-	-
1381	ν_{58}	1363	$C_{8a,11}$-H_3, C_{9a}-N_{10}, C_{10a}-C_{4a}, N_1-C_2, C_4-N_3, C_{5a}-N_5	-	-	-
	ν_{59}	1365	C_{8a}-H_3, C_9-H, N_5-C_{4a}, C_{9a}-C_{5a}	ν_{57}	1398	C_{11}-H_3, N_3-H, C_{10a}-N_1, C_{5a}-N_5, C_7-C_6, C_{9a}-N_{10}
	ν_{60}	1368	N_3-H, C_6-H, C_{9a}-C_{5a}, C_{10a}-C_{4a}, C_4-N_3	ν_{58}	1404	$C_{8a,7a}$-H_3, C_6-C_{5a}, C_8-C_7, C_{9a}-C_{5a}
	ν_{61}	1380	$C_{11,8a,7a}$-H_3, C_{9a}-C_{5a}, C_8-C_7, C_{10a}-N_1	ν_{60}	1426	$C_{11,7a}$-H_3, C_{5a}-N_5, N_{10}-C_{10a}, C_2-N_3
1416	ν_{64}	1415	C_{7a}-H_3, N_5-C_{4a}, C_4-N_3, C_{10a}-N_1	ν_{64}	1459	C_{8a}-H_3, C_{10a}-C_{4a}, N_3-H, C_2-N_3, C_{9a}-C_{5a}, N_1-C_2
	ν_{65}	1428	$C_{11,8a}$-H_3, C_9-H, C_{10a}-C_{4a}, N_1-C_2	ν_{68}	1484	C_{11}-H_3, N_{10}-C_{10a}, N_5-C_{4a}, $C_{7a,8a}$-H_3, N_1-C_2
1498	ν_{71}	1511	C_8-C_7, C_{9a}-C_{5a}, $C_{6,9}$-H, $C_{7a,8a}$-H_3, C_6-C_{5a}, C_9-C_{9a}, C_{10a}-C_{4a}	ν_{72}	1585	C_{10a}-N_1, N_5-C_{4a}, C_8-C_9, C_7-C_6, C_{9a}-C_{5a}, $C_{6,9}$-H, N_3-H, C_4-N_3
1570	ν_{73}	1587	C_2-$O_{2'}$, N_3-H, C_6-C_{5a}, $C_{9,6}$-H	ν_{73}	1656	C_8-C_9, C_6-C_{5a}, C_{9a}-N_{10}, C_7-C_6, $C_{9,6}$-H, C_{10a}-N_1, C_{4a}-C_4
	ν_{74}	1608	(s) C_2-$O_{2'}$, C_4-$O_{4'}$, C_8-C_9, C_6-C_{5a}, $C_{6,9}$-H, C_{9a}-N_{10}, C_{10a}-N_1	ν_{74}	1759	C_4-$O_{4'}$, N_3-H, C_2-$O_{2'}$ (as)
1626	ν_{75}	1645	C_4-$O_{4'}$, N_3-H, C_{4a}-C_4, C_6-C_{5a}, C_8-C_9	ν_{75}	1782	C_2-$O_{2'}$, C_4-$O_{4'}$, N_3-H (s)

**Exp. RR FMN S_1**	**X3LYP/cc-pVDZ**		
	**ν#**	**S_1offR**	**Assignment**
1200	ν_{51}	1232	C_2-N_3, N_3-H, C_{10a}-C_{4a}, C_9-H, C_{9a}-C_{5a}, N_{10}-C_{11}
	-	-	-
1250	ν_{53}	1298	N_{10}-C_{10a}, C_{11}-H_3, $C_{6,9}$-H, C_8-C_7, N_5-C_{4a}, N_3-C_2, C_{10a}-C_{4a}
1338	ν_{54}	1321	C_{5a}-N_5, $C_{6,9}$-H, C_9-C_{9a}, N_{10}-C_{10a}, C_{4a}-C_4, N_3-C_2, C_8-C_7
	ν_{55}	1345	N_3-H, N_{10}-C_{10a}, C_{4a}-C_{10a}, C_{5a}-C_{9a}, C_7-C_6, C_{11}-H_3, C_6-H
1381	ν_{57}	1383	N_3-H, C_6-H, C_{11}-H_3, C_7-C_6, C_{9a}-N_{10}, C_6-C_{5a}, C_{10a}-C_{4a}, C_4-N_3
	ν_{59}	1394	C_{8a}-H_3, C_6-C_{5a}, C_8-C_9, N_1-C_2, C_4-N_3
	ν_{60}	1403	C_{10a}-N_1, N_5-C_{4a}, C_{9a}-N_{10}, N_1-C_2, N_3-H, N_2-C_3, C_9-C_{9a}, $C_{11,7a,8a}$-H_3
	ν_{61}	1410	N_3-H, C_4-N_3, C_6-H, C_{9a}-C_{5a}, N_5-C_{4a}, C_{10a}-N_1
1416	ν_{64}	1444	N_5-C_{4a}, C_{10a}-N_1, C_4-N_3, C_2-N_3, $C_{7a,11}$-H_3, C_8-C_7
	ν_{65}	1447	$C_{8a,11}$-H_3, C_{10a}-C_{4a}, N_1-C_2, C_8-C_9, C_7-C_6, C_{4a}-C_4
1498	ν_{71}	1527	C_8-C_7, C_9-C_{9a}, C_{9a}-C_{5a}, C_6-C_{5a}, C_{10a}-C_{4a}, $C_{6,9}$-H, C_{7a}-H_3
1570	ν_{73}	1635	C_8-C_9, C_6-C_{5a}, $C_{6,9}$-H, C_7-C_6, N_{10}-C_{9a}, N_5-C_{4a}, C_{10a}-N_1
	ν_{74}	1701	C_2-$O_{2'}$, N_3-H, C_4-$O_{4'}$ (as), C_{4a}-C_{10a}, C_4-N_3
1626	ν_{75}	1726	C_4-$O_{4'}$, C_2-$O_{2'}$ (s), N_3-H, C_{4a}-C_4

Table S13 Assignment Tables between the experimental FSRS 3rd EAS of 1FMN* (**Exp. FSRS**)[61,76] and the calculated excited singlet state Resonance Raman spectra of each DFT functional (**R_nRes**). The off-Resonance assignment is shown with black numbers (**S_1offR**) and any new assignment due to the new rR intensities is highlighted in red and the vibration number is given in the **$\nu_{\#}$** column. For those vibrations that assignment to normal modes is missing in **Table S12**, assignments have been inserted according to the notation introduced in **Table 1** of the main text. For the functionals HCTH, OLYP and TPSSh, the assigned stretching modes have been colour-coded according to their blue- or red- shift contribution (see the singlet-triplet shift discussion in the main text). Normal modes are ordered from the ones containing the largest to the smallest displacement vectors.

**Exp. RR FMN S_1**	**APFD/cc-pvdz**					**B1B95/cc-pVDZ**				
	**$\nu_{\#}$**	**S_1offR**	**$\nu_{\#}$**	**R_5Res.**	**Assignment**	**$\nu_{\#}$**	**S_1offR**	**$\nu_{\#}$**	**R_7Res.**	**Assignment**
1200	-	-		1209		ν_{51}	1263		1208	
	ν_{50}	1209				-	-			
1250	ν_{51}	1243		1243		ν_{53}	1323		1280	
1338	-	-				-	-			
	ν_{56}	1368	ν_{52}	1279	rCH_{I}, sCC_{I}, xCH_{3I}	ν_{56}	1378	ν_{55}	1367	xCH_{3I}, sCC_{I}, rNH_{III}, sNC_{II}
1381	ν_{58}	1388				-	-			
	ν_{59}	1400	ν_{54}	1332	sNC_{II}, $sCC_{II,I}$, rCH_{I}, sNC_{III}	ν_{58}	1395			
	ν_{60}	1416				ν_{59}	1406	ν_{61}	1428	$xCH_{3I,II}$, $sNC_{II,III}$, rNH_{III}, sCC_{II}
	ν_{62}	1422				-	-			
1416	ν_{65}	1448		1416		ν_{63}	1435		1453	
	-	-				ν_{65}	1453			
1498	ν_{71}	1539	ν_{69}	1476	$xCH_{3II,I}$, sCC_{I}, sNC_{II}, sCC_{II}	ν_{71}	1553	ν_{68}	1477	xCH_{3II}, $sNC_{II,III}$, rNH_{III}
1570	ν_{73}	1650		1716		ν_{73}	1663		1577	
	ν_{74}	1716				ν_{74}	1742			
1626	ν_{75}	1744		1744		ν_{75}	1763		1742	
**Exp. RR FMN S_1**	**B3LYP/cc-pVDZ**					**B3LYP/aug-cc-pVDZ**				
	**$\nu_{\#}$**	**S_1offR**	**$\nu_{\#}$**	**R_7Res.**	**Assignment**	**$\nu_{\#}$**	**S_1offR**	**$\nu_{\#}$**	**R_6Res.**	**Assignment**
1200	ν_{49}	1191		1191		ν_{49}	1193		1193	
	-	-				-	-			
1250	ν_{51}	1226	ν_{52}	1271	rCH_{I}, $sNC_{II,III}$, rNH_{III}	ν_{51}	1223		1223	
1338	ν_{54}	1316	ν_{55}	1340	rNH_{III}, sCC_{I}, $sNC_{II,III}$	ν_{54}	1317			
	-	-				ν_{55}	1339		1317	
1381	ν_{57}	1379				ν_{57}	1376			
	ν_{58}	1381				ν_{58}	1382			
	ν_{59}	1391	ν_{60}	1397	$xCH_{3I,II}$, $sNC_{II,III}$, sCC_{I}	ν_{59}	1394		1394	
	ν_{61}	1405				-	-			
1416	ν_{64}	1439		1444		ν_{64}	1436		1407	
	ν_{65}	1444				-	-			
1498	ν_{71}	1524	ν_{70}	1509	$xCH_{3II,I}$, $sNC_{II,III}$, sCC_{I}	ν_{71}	1514	ν_{70}	1502	sCC_{I}, $xCH_{3II,I}$, $sNC_{II,III}$, sCC_{III}
1570	ν_{73}	1630	ν_{72}	1541	sCC_{II}, sCC_{I}, rCH_{I}, sNC_{III}	ν_{73}	1625	ν_{72}	1537	sCC_{I}, rCH_{I}, sCC_{II}, $sNC_{II,III}$
	ν_{74}	1692				ν_{74}	1650			
1626	ν_{75}	1719		1630		ν_{75}	1663		1650	
**Exp. RR FMN S_1**	**B3LYP/cc-pVTZ**					**B3LYP/aug-cc-pVTZ**				
	**$\nu_{\#}$**	**S_1offR**	**$\nu_{\#}$**	**R_7Res.**	**Assignment**	**$\nu_{\#}$**	**S_1offR**	**$\nu_{\#}$**	**R_7Res.**	**Assignment**
1200	ν_{49}	1194	ν_{50}	1200	rCH_{I}, $sNC_{II,III}$, sCC_{I}	ν_{49}	1193		1193	

	ν_{51}	1213				ν_{51}	1211			
1250	ν_{52}	1284		1284		ν_{52}	1285		1285	
1338	ν_{54}	1317				ν_{54}	1316			
	-	-		1317		ν_{55}	1337		1337	
1381	ν_{57}	1381				ν_{57}	1378			
	ν_{58}	1391				ν_{58}	1392		1392	
	-	-		1391		-	-			
	-	-				-	-			
1416	ν_{62}	1429		1429		ν_{62}	1428	ν_{60}	1407	*r*NH$_{III}$, *r*CH$_{I}$, *s*CC$_{I}$, *s*NC$_{III}$
	ν_{63}	1441				ν_{63}	1440			
1498	ν_{71}	1513		1513		ν_{71}	1511	ν_{72}	1536	*r*CH$_{I}$, *s*CC$_{I,II}$, *s*NC$_{II}$, *x*CH$_{3II}$
1570	ν_{73}	1625		1625		ν_{73}	1622		1622	
	ν_{74}	1668				ν_{74}	1651			
1626	ν_{75}	1686		1668		ν_{75}	1664		1651	
**Exp. RR FMN S$_1$**	**B3LYP/cc-pVQZ**					**B3P86/cc-pVDZ**				
	**$\nu_{\#}$**	**S$_1$offR**	**$\nu_{\#}$**	**R$_7$Res.**	**Assignment**	**$\nu_{\#}$**	**S$_1$offR**	**$\nu_{\#}$**	**R$_5$Res.**	**Assignment**
1200	ν_{49}	1194		1211		ν_{51}	1263		1197	
	ν_{51}	1211				-	-			
1250	ν_{52}	1285		1285		ν_{53}	1323	ν_{52}	1272	*r*CH$_{I}$, *x*CH$_{3II,I}$, *s*CC$_{I}$, *s*NC$_{II}$
1338	ν_{54}	1317				-	-			
	-	-		1317		ν_{56}	1378	ν_{55}	1355	*r*NH$_{III}$, *s*CC$_{I}$, *s*NC$_{II,III}$, , *x*CH$_{3II,I}$
1381	ν_{57}	1379				-	-			
	ν_{58}	1393		1393		ν_{58}	1395	ν_{60}	1414	
	-	-				ν_{59}	1406			
	-	-				-	-			
1416	ν_{62}	1429		1429		ν_{63}	1435		1445	
	ν_{63}	1441				ν_{65}	1453			
1498	ν_{71}	1512	ν_{70}	1506	*x*CH$_{3II,I}$, *r*CH$_{I}$, *s*CC$_{I}$, *s*NC$_{III}$	ν_{71}	1553	ν_{72}	1561	*s*CC$_{II,III}$, *s*NC$_{II}$, *r*CH$_{I}$, *s*NC$_{III}$
1570	ν_{73}	1623		1623		ν_{73}	1663		1648	
	ν_{74}	1659				ν_{74}	1742			
1626	ν_{75}	1675		1675		ν_{75}	1763		1714	
**Exp. RR FMN S$_1$**	**B3PW91/cc-pVDZ**					**B98/cc-pVDZ**				
	**$\nu_{\#}$**	**S$_1$offR**	**$\nu_{\#}$**	**R$_7$Res.**	**Assignment**	**$\nu_{\#}$**	**S$_1$offR**	**$\nu_{\#}$**	**R$_7$Res.**	**Assignment**
1200	ν_{49}	1197		1197		ν_{51}	1228	ν_{49}	1191	*r*CH$_{I}$, *x*CH$_{3II}$, *r*NH$_{III}$, *s*NC$_{II}$
	ν_{50}	1209				-	-			
1250	ν_{51}	1245	ν_{52}	1272	*r*CH$_{I}$, *s*CC$_{I}$, *x*CH$_{3II,I}$	ν_{53}	1294	ν_{52}	1267	*r*CH$_{I}$, *s*CC$_{I}$, *x*CH$_{3II}$, *s*CC$_{II}$
1338						ν_{54}	1314			
	ν_{56}	1366	ν_{55}	1353	*r*NH$_{III}$, *s*CC$_{I}$, *s*NC$_{II,III}$, , *x*CH$_{3II,I}$	-	-	ν_{55}	1343	*r*NH$_{III}$, *s*CC$_{I,II}$, *s*NC$_{II}$, *x*CH$_{3II}$
1381	ν_{58}	1384				ν_{57}	1378			
	ν_{59}	1397		1412		ν_{58}	1380	ν_{60}	1400	*s*NC$_{II,III}$, *x*CH$_{3II}$ *s*CC$_{I}$, *r*NH$_{III}$
	ν_{60}	1412				ν_{59}	1390			
	ν_{61}	1414				ν_{61}	1406			
1416						ν_{64}	1440		1443	
	ν_{65}	1445		1445		ν_{65}	1443			
1498	ν_{71}	1537	ν_{72}	1558	*s*CC$_{II,I}$, *r*CH$_{I}$, *s*NC$_{III}$, *r*CH$_{I}$	ν_{71}	1519	ν_{72}	1539	*s*CC$_{II,I}$, *s*NC$_{II,III}$, *r*CH$_{I}$

1570	ν_{73}	1645		1645		ν_{73}	1626		1626	
	ν_{74}	1711				ν_{74}	1709			
1626	ν_{75}	1739		1711		ν_{75}	1733		1709	
**Exp. RR FMN S_1**	**BHHLYP/cc-pVDZ**					**BHHLYP/aug-cc-pVDZ**				
	**$\nu_{\#}$**	**S_1offR**	**$\nu_{\#}$**	**R_4Res.**	**Assignment**	**$\nu_{\#}$**	**S_1offR**	**$\nu_{\#}$**	**R_5Res.**	**Assignment**
1200	ν_{46}	1144		1146		ν_{46}	1146		1146	
	-	-				-	-			
1250	ν_{50}	1264	ν_{48}	1212	sCC_{I}, rCH_{I}, $xCH_{3I,II}$, $sNC_{II,III}$	ν_{50}	1265	ν_{48}	1212	sCC_{I}, rCH_{I}, $xCH_{3I,II}$, $sNC_{II,III}$
1338	ν_{54}	1381		1304		ν_{54}	1374			
	-	-				-	-		1265	
1381	-	-				ν_{56}	1438			
	ν_{57}	1442				ν_{57}	1442			
	ν_{58}	1448		1383		ν_{58}	1449	ν_{51}	1299	$sNC_{II,III}$, rNH_{III}, rCH_{I}, $sCC_{III,I}$
	-	-				-	-			
1416	ν_{60}	1477	ν_{59}	1458	rNH_{III}, sNC_{III}, sCC_{II}, xCH_{3I}	ν_{60}	1482		1442	
	ν_{64}	1504				ν_{63}	1507			
1498	ν_{72}	1632	ν_{61}	1486	$xCH_{3II,I}$, rNH_{III}, $sNC_{II,III}$	ν_{72}	1630	ν_{66}	1518	$sNC_{II,III}$, rNH_{III}, $sCC_{III,I}$, xCH_{3I}, rCH_{I}
1570	ν_{73}	1696	ν_{68}	1526	$xCH_{3II,I}$, $sCC_{I,II}$, rNH_{III}, sNC_{III}	ν_{73}	1693	ν_{71}	1595	$sNC_{II,III}$, sCC_{I}, $xCH_{3I,II}$, rCH_{I}
	ν_{74}	1801				ν_{74}	1742			
1626	ν_{75}	1822		1801		ν_{75}	1765		1693	
**Exp. RR FMN S_1**	**BLYP/cc-pVDZ**					**BMK/aug-cc-pVDZ**				
	**$\nu_{\#}$**	**S_1offR**	**$\nu_{\#}$**	**R_7Res.**	**Assignment**	**$\nu_{\#}$**	**S_1offR**	**$\nu_{\#}$**	**R_5Res.**	**Assignment**
1200	ν_{51}	1168	ν_{50}	1138	rCH_{I}, sNC_{III}, xCH_{3II}, rNH_{III}	ν_{50}	1227	ν_{48}	1174	rCH_{I}, xCH_{3I}, sCC_{I}, sNC_{II}
	ν_{52}	1219				-	-			
1250	ν_{53}	1238		1238		ν_{51}	1260		1260	
1338	ν_{54}	1258				ν_{54}	1333			
	ν_{55}	1284		1284		ν_{55}	1378		1401	
1381	ν_{56}	1311				ν_{56}	1390			
	ν_{59}	1340	ν_{57}	1322	$sNC_{II,III}$, rCH_{I}, xCH_{3II}, rNH_{III}	ν_{57}	1396	ν_{60}	1430	rNH_{III}, $sNC_{II,III}$, xCH_{3II}, sCC_{II}
	ν_{60}	1343				ν_{58}	1401			
	-	-				ν_{59}	1406			
1416	ν_{62}	1366		1340		ν_{61}	1441	ν_{66}	1466	$xCH_{3I,II}$, rCH_{I}, $sNC_{II,III}$, sCC_{I}
	ν_{65}	1397				ν_{64}	1463			
1498	ν_{71}	1475		1475		ν_{71}	1534		1534	
1570	ν_{73}	1554		1554		ν_{73}	1638	ν_{72}	1557	$sNC_{II,III}$, sCC_{I}, rCH_{I}, rNH_{III}
	ν_{74}	1568				ν_{74}	1726			
1626	ν_{75}	1643		1643		ν_{75}	1753		1638	
**Exp. RR FMN S_1**	**BP86/cc-pVDZ**					**BPBE/cc-pVDZ**				
	**$\nu_{\#}$**	**S_1offR**	**$\nu_{\#}$**	**R_7Res.**	**Assignment**	**$\nu_{\#}$**	**S_1offR**	**$\nu_{\#}$**	**R_7Res.**	**Assignment**
1200	ν_{48}	1118		1145		ν_{48}	1126	ν_{50}	1151	rNH_{III}, rCH_{I}, xCH_{3II}, $sNC_{III,II}$, sCC_{I}
	ν_{51}	1184				ν_{51}	1191			
1250	ν_{53}	1241		1241		ν_{53}	1248		1248	
1338	-	-				ν_{55}	1303			
	ν_{55}	1297	ν_{55}	1333	rNH_{III}, $sNC_{II,III}$, sCC_{III} $xCH_{3I,II}$	ν_{57}	1333		1340	

1381	ν_{57}	1326				-	-			
	-	-		1346		ν_{58}	1340		1353	
	ν_{60}	1346				ν_{60}	1353			
	ν_{61}	1356				ν_{61}	1363			
1416	ν_{64}	1391		1391		ν_{64}	1397	ν_{65}	1399	$xCH_{3II,I}$, $sNC_{II,III}$, sCC_{I}
	ν_{65}	1391				-	-			
1498	ν_{71}	1487		1487		ν_{71}	1494		1494	
1570	ν_{73}	1572		1572		ν_{73}	1579		1579	
	ν_{74}	1589				ν_{74}	1596			
1626	ν_{75}	1665		1589		ν_{75}	1671		1596	
**Exp. RR FMN S_1**	**CAM-B3LYP/cc-pVDZ**					**HCTH/407/cc-pVDZ**				
	**$\nu_{\#}$**	**S_1offR**	**$\nu_{\#}$**	**R_5Res.**	**Assignment**	**$\nu_{\#}$**	**S_1offR**	**$\nu_{\#}$**	**R_7Res.**	**Assignment**
1200	ν_{48}	1181		1216		ν_{48}	1150	ν_{50}	1179	$C_{6,9}$-H, N_3-H, C_{11}-H_3, C_4-N_3, N_1-C_2, N_5-C_{4a}, N_{10}-C_{10a}
	ν_{49}	1216				ν_{51}	1216			
1250	ν_{50}	1234	ν_{52}	1286	rCH_{I}, sCC_{I}, $xCH_{3I,II}$, $sNC_{II,III}$	ν_{53}	1274		1274	
1338	ν_{53}	1319				ν_{55}	1325			
	ν_{54}	1348		1402		ν_{56}	1342		1342	
1381	ν_{57}	1402				ν_{57}	1360			
	ν_{58}	1407		1409		ν_{58}	1360		1370	
	ν_{59}	1409				ν_{59}	1370			
	ν_{60}	1430				ν_{61}	1388			
1416	ν_{64}	1459		1430		ν_{65}	1422		1422	
	ν_{68}	1483				-	-			
1498	ν_{72}	1585		1459		ν_{72}	1538	ν_{71}	1520	C_9-C_{9a}, C_6-C_{5a}, C_8-C_7, $C_{6,9}$-H, C_{10a}-N_1
1570	ν_{73}	1661		1585		ν_{73}	1613		1538	
	ν_{74}	1754				ν_{74}	1640			
1626	ν_{75}	1775		1661		ν_{75}	1711		1613	
**Exp. RR FMN S_1**	**HISSbPBE/cc-pVDZ**					**HSEH1PBE/cc-pVDZ**				
	**$\nu_{\#}$**	**S_1offR**	**$\nu_{\#}$**	**R_7Res.**	**Assignment**	**$\nu_{\#}$**	**S_1offR**	**$\nu_{\#}$**	**R_7Res.**	**Assignment**
1200	ν_{51}	1310	ν_{46}	1140	rNH_{III}, xCH_{3II}, $sNC_{II,III}$, sCC_{I}	ν_{49}	1201		1202	
	ν_{52}	1310				ν_{50}	1215			
1250	ν_{53}	1362	ν_{50}	1252	rCH_{I}, xCH_{3II}, $sNC_{II,III}$, $sCC_{I,II}$	ν_{51}	1257	ν_{52}	1275	rCH_{I}, xCH_{3I}, $sNC_{II,III}$, sCC_{I}
1338	ν_{54}	1380				-	-			
	ν_{55}	1399		1310		ν_{56}	1369	ν_{55}	1361	rNH_{III}, $xCH_{3I,II}$, $sNC_{II,III}$, $sCC_{I,III}$
1381	ν_{57}	1427				ν_{58}	1389			
	ν_{58}	1430				ν_{59}	1402	ν_{60}	1419	$xCH_{3I,II}$, sCC_{I}, $sNC_{II,III}$
	ν_{59}	1440		1457		ν_{61}	1422			
	ν_{60}	1457				ν_{62}	1423			
1416	ν_{63}	1474		1492		-	-		1450	
	ν_{65}	1492				ν_{65}	1449			
1498	ν_{70}	1580	ν_{68}	1521	xCH_{3II}, $sNC_{II,III}$, sCC_{I}	ν_{71}	1543	ν_{68}	1471	$xCH_{3I,II}$, $sNC_{II,III}$
1570	ν_{73}	1702	ν_{72}	1622	$sNC_{III,II}$, sCC_{I}, rCH_{I}	ν_{73}	1653	ν_{72}	1570	$sCC_{I,II}$, $sNC_{II,III}$, rCH_{I}
	ν_{74}	1794				ν_{74}	1728			
1626	ν_{75}	1813		1794		ν_{75}	1754		1656	
**Exp. RR**	**LC-OPBE/cc-pVDZ**					**LC-OPBE/cc-pVDZ (scaled)**				

FMN S_1	$v_\#$	S_1offR	$v_\#$	R_4Res.	Assignment	$v_\#$	S_1offR	$v_\#$	R_4Res.	Assignment
1200	-	-	v_{55}	1409	*x*CH$_{3I,II}$, *s*CC$_{I,II}$, *s*NC$_{II,III}$	-	-	v_{55}	1205	-
	v_{50}	1266				v_{52}	1259			
1250	v_{51}	1308	v_{57}	1432	*x*CH$_{3II,I}$, *s*CC$_{I}$, *s*NC$_{II,III}$	v_{53}	1282	v_{57}	1224	-
1338	v_{53}	1382				v_{54}	1305			
	-	-		1538		-	-		1316	
1381	v_{59}	1439				-	-			
	v_{61}	1459				v_{58}	1355			
	v_{64}	1474		1557		v_{62}	1377		1332	
	v_{66}	1538				v_{63}	1383			
1416	v_{67}	1557	v_{70}	1656	*s*NC$_{III,II}$, *s*CC$_{I}$, *r*CH$_{I}$, *r*NH$_{III}$	v_{67}	1426	v_{70}	1417	-
	v_{68}	1594				v_{68}	1467			
1498	v_{71}	1701		1788		v_{71}	1547		1529	
1570	v_{73}	1788		1865		v_{72}	1577		1595	
	v_{74}	1865				v_{73}	1611			
1626	v_{75}	1901		1901		v_{75}	1702		1626	
**Exp. RR**	**LC-wHPBE/cc-pVDZ**					**LSDA/cc-pVDZ**				
**FMN S_1**	$v_\#$	S_1offR	$v_\#$	R_5Res.	**Assignment**	$v_\#$	S_1offR	$v_\#$	R_8Res.	**Assignment**
1200	v_{54}	1369		1383		v_{49}	1160		1141	
	v_{55}	1383				-	-			
1250	v_{60}	1437	v_{60}	1424	*r*NH$_{III}$, *s*CC$_{I}$, *x*CH$_{3II,I}$, *s*NC$_{II,III}$	v_{53}	1266		1266	
1338	-	-				v_{57}	1322			
	v_{64}	1469		1499		v_{58}	1324	v_{63}	1365	*x*CH$_{3II,I}$, *s*NC$_{III}$, *r*NH$_{III}$, *s*CC$_{I}$
1381	-	-				-	-			
	v_{65}	1469				v_{65}	1390		1413	
	v_{66}	1477		1569		v_{66}	1402			
	v_{67}	1499				-	-			
1416	-	-		1629		v_{67}	1413	v_{68}	1448	*s*NC$_{II,III}$, *s*CC$_{I}$, *x*CH$_{3II,I}$
	v_{70}	1579				-	-			
1498	v_{71}	1629		1675		v_{71}	1529		1529	
1570	v_{72}	1675		1790		v_{73}	1623	v_{72}	1553	*s*CC$_{II,I}$, *s*NC$_{II,III}$, *s*CO, *r*CH$_{I}$
	v_{73}	1695				v_{74}	1652			
	v_{74}	1790		1821		-	-		1623	
1626	v_{75}	1821				v_{75}	1730			
**Exp. RR**	**M052X/aug-cc-pVDZ**					**M06/cc-pVDZ**				
**FMN S_1**	$v_\#$	S_1offR	$v_\#$	R_4Res.	**Assignment**	$v_\#$	S_1offR	$v_\#$	R_7Res.	**Assignment**
1200	v_{50}	1239	v_{48}	1184	*x*CH$_{3I}$, *r*CH$_{I}$, *s*CC$_{I}$, *s*NC$_{II}$	v_{49}	1183		1198	
	-	-				v_{50}	1198			
1250	v_{51}	1266		1266		v_{51}	1234		1234	
1338	v_{53}	1327				v_{52}	1250		1367	
	v_{54}	1346		1327		v_{56}	1357			
1381	v_{57}	1410				v_{58}	1367			
	v_{58}	1412		1410		v_{59}	1380	v_{62}	1404	*x*CH$_{3I,II}$, *s*NC$_{II,III}$, *s*CC$_{I}$, *r*CH$_{I}$
	-	-				v_{63}	1410			

	-	-				ν_{66}	1425			
1416	ν_{60}	1451		1451		ν_{68}	1450		1450	
	ν_{63}	1476				ν_{69}	1460			
1498	ν_{72}	1594	ν_{64}	1478	$xCH_{3I,II}$, rCH_I, $sNC_{II,III}$, sCC_I	ν_{71}	1526	ν_{70}	1521	$sNC_{II,III}$, sCC_I, $xCH_{3I,II}$, rCH_I
1570	ν_{73}	1662		1662		ν_{73}	1646		1646	
	ν_{74}	1706				ν_{74}	1747			
1626	ν_{75}	1731		1731		ν_{75}	1770		1770	
**Exp. RR FMN S_1**	**M06/aug-cc-pVDZ**					**M06-HF/cc-pVDZ**				
	**$\nu_\#$**	**S_1offR**	**$\nu_\#$**	**R_5Res.**	**Assignment**	**$\nu_\#$**	**S_1offR**	**$\nu_\#$**	**R_6Res.**	**Assignment**
1200	ν_{49}	1188	ν_{46}	1094	xCH_{3II}, rNH_{III}, $sNC_{II,III}$, sCC_I	ν_{49}	1183		1183	
	ν_{50}	1203				ν_{50}	1198			
1250	ν_{51}	1235		1235		ν_{51}	1234	ν_{52}	1250	rCH_I, xCH_{3II}, $sNC_{II,III}$
1338	-	-				ν_{54}	1317		1357	
	ν_{56}	1360		1385		ν_{56}	1357			
1381	ν_{57}	1367				ν_{57}	1363			
	ν_{58}	1375		1404		ν_{58}	1367	ν_{62}	1404	xCH_{3II}, $sNC_{II,III}$, sCC_I, rNH_{III}
	ν_{59}	1385				ν_{59}	1380			
	-	-				-	-			
1416	ν_{66}	1433	ν_{68}	1450	$sNC_{II,III}$, xCH_{3II}	ν_{63}	1410	ν_{68}	1450	$sNC_{II,III}$, $xCH_{3II,I}$
	ν_{69}	1457				ν_{66}	1425			
1498	ν_{71}	1518	ν_{70}	1514	$sNC_{II,III}$, sCC_I, xCH_{3II}, rCH_I	ν_{71}	1526	ν_{70}	1521	$sNC_{II,III}$, sCC_I, $xCH_{3II,I}$, rCH_I
1570	ν_{73}	1644		1696		ν_{73}	1646	ν_{72}	1556	$sNC_{II,III}$, $sCC_{I,II}$, rCH_I
	ν_{74}	1696				ν_{74}	1747			
1626	ν_{75}	1717		1717		ν_{75}	1770		1646	
**Exp. RR FMN S_1**	**M06L/cc-pVDZ**					**M06L/aug-cc-pVDZ**				
	**$\nu_\#$**	**S_1offR**	**$\nu_\#$**	**R_5Res.**	**Assignment**	**$\nu_\#$**	**S_1offR**	**$\nu_\#$**	**R_7Res.**	**Assignment**
1200	ν_{51}	1255		1255		ν_{48}	1174	ν_{50}	1190	rNH_{III}, rCH_I, $sNC_{II,III}$, xCH_{3II}
	ν_{52}	1266				ν_{49}	1180			
1250	ν_{53}	1301		1266		ν_{51}	1226	ν_{52}	1273	rCH_I, xCH_{3I}, sCC_I
1338	ν_{55}	1347				ν_{54}	1308			
	ν_{56}	1365		1347		ν_{56}	1363	ν_{55}	1337	rNH_{III}, $sNC_{II,III}$, $sCC_{I,II}$, $xCH_{3I,II}$
1381	ν_{61}	1424				ν_{57}	1370			
	ν_{63}	1432		1424		ν_{58}	1382		1389	
	-	-				ν_{59}	1389			
	ν_{65}	1446				ν_{61}	1403			
1416	ν_{67}	1458		1458		ν_{65}	1438		1438	
	ν_{68}	1490				ν_{66}	1440			
1498	ν_{71}	1531		1490		ν_{71}	1535	ν_{72}	1549	rCH_I, $sCC_{I,II}$, $sNC_{II,III}$
1570	ν_{73}	1626		1626		ν_{73}	1635		1635	
	ν_{74}	1661				ν_{74}	1652			
1626	ν_{75}	1761		1761		ν_{75}	1695		1652	
**Exp. RR FMN S_1**	**M11L/cc-pVDZ**					**MN15/cc-pVDZ**				
	**$\nu_\#$**	**S_1offR**	**$\nu_\#$**	**R_6Res.**	**Assignment**	**$\nu_\#$**	**S_1offR**	**$\nu_\#$**	**R_5Res.**	**Assignment**

1200	-	-		1178		ν_{49}	1196	ν_{48}	1164	sCC_{I}, $xCH_{3I,II}$, rCH_{I}
	ν_{49}	1178				ν_{50}	1211			
1250	ν_{52}	1269		1269		ν_{52}	1264		1264	
1338	ν_{53}	1306				ν_{53}	1313			
	ν_{55}	1339		1306		ν_{55}	1351		1313	
1381	ν_{56}	1357				ν_{58}	1384			
	ν_{58}	1375		1387		ν_{59}	1391	ν_{54}	1333	$sCC_{I,II}$, $sNC_{II,III}$, rNH_{III}
	ν_{60}	1387				ν_{61}	1405			
	ν_{63}	1414				-	-			
1416	ν_{66}	1439	ν_{61}	1409	$xCH_{3II,I}$, sCC_{I}, $sNC_{II,III}$, rCH_{I}	ν_{67}	1457		1457	
	ν_{69}	1486				ν_{68}	1470			
1498	ν_{71}	1543		1543		ν_{71}	1547		1470	
1570	ν_{73}	1664	ν_{72}	1581	sCC_{I}, $sNC_{II,III}$, rCH_{I}, rNH_{III}	ν_{73}	1650		1650	
	ν_{74}	1750				ν_{74}	1757			
1626	ν_{75}	1789		1664		ν_{75}	1777		1777	
**Exp. RR FMN S_1**	**MN15/aug-cc-pVDZ**					**mPW1PW91/cc-pVDZ**				
	**$\nu_{\#}$**	**S_1offR**	**$\nu_{\#}$**	**R_4Res.**	**Assignment**	**$\nu_{\#}$**	**S_1offR**	**$\nu_{\#}$**	**R_7Res.**	**Assignment**
1200	-	-	ν_{48}	1154	sCC_{I}, rCH_{I}, $xCH_{3II,I}$	ν_{49}	1209		1209	
	ν_{50}	1214				ν_{50}	1222			
1250	ν_{51}	1258	ν_{52}	1266	rCH_{I}, $xCH_{3II,I}$, sCC_{I}, $sNC_{II,III}$	ν_{51}	1262	ν_{52}	1281	rCH_{I}, $xCH_{3II,I}$, sCC_{I}, $sNC_{II,III}$
1338	ν_{53}	1308				-	-			
	-	-		1308		ν_{56}	1377	ν_{54}	1342	$sNC_{II,III}$, sCC_{I}, rCH_{I}, $xCH_{3II,I}$
1381	ν_{58}	1386				ν_{58}	1396			
	ν_{59}	1393		1386		ν_{59}	1408	ν_{55}	1369	rNH_{III}, $xCH_{3II,I}$, $sCC_{I,II}$, sNC_{III}
	ν_{64}	1438				ν_{61}	1428			
	ν_{65}	1446				ν_{62}	1432			
1416	ν_{67}	1456		1470		ν_{65}	1456		1428	
	ν_{68}	1470				-	-			
1498	ν_{70}	1530		1530		ν_{71}	1549	ν_{68}	1476	xCH_{3II}, $sNC_{II,III}$, sCC_{I}
1570	ν_{73}	1641	ν_{72}	1566	$sNC_{II,III}$, sCC_{I}, rNH_{III}, rCH_{I}	ν_{73}	1661	ν_{72}	1574	$sCC_{II,I}$, $sNC_{II,III}$, rCH_{I}
	ν_{74}	1698				ν_{74}	1736			
1626	ν_{75}	1722		1641		ν_{75}	1758		1661	
**Exp. RR FMN S_1**	**mPWLYP/cc-pVDZ**					**O3LYP/cc-pVDZ**				
	**$\nu_{\#}$**	**S_1offR**	**$\nu_{\#}$**	**R_7Res.**	**Assignment**	**$\nu_{\#}$**	**S_1offR**	**$\nu_{\#}$**	**R_7Res.**	**Assignment**
1200	ν_{49}	1125		1137		ν_{49}	1183	ν_{50}	1192	rNH_{III}, $sNC_{II,III}$, $xCH_{3II,I}$, sCC_{I}
	ν_{50}	1137				-	-			
1250	ν_{55}	1282	ν_{53}	1237	rCH_{I}, $sNC_{II,III}$, sCC_{I}	ν_{51}	1228	ν_{52}	1268	rCH_{I}, sCC_{I}, $xCH_{3II,I}$
1338	-	-				ν_{53}	1290	ν_{55}	1336	rNH_{III}, $sCC_{I,II}$, $sNC_{II,III}$, $xCH_{3II,I}$
	ν_{60}	1341		1310		-	-			
1381	-	-				ν_{57}	1373			
	ν_{62}	1365	ν_{57}	1320	$sNC_{II,III}$, rNH_{III}, sCC_{I}, $xCH_{3II,I}$	ν_{58}	1376	ν_{60}	1396	sCC_{I}, $xCH_{3II,I}$, $sNC_{II,III}$
	ν_{63}	1383				ν_{59}	1385			
	-	-				ν_{61}	1402			
1416	ν_{67}	1415	ν_{70}	1443	sCC_{I}, $xCH_{3II,I}$, sNC_{II}, rCH_{I}	ν_{64}	1437		1437	

	-	-				-	-			
1498	ν_{71}	1474		1474		ν_{71}	1526		1526	
1570	ν_{73}	1553		1553		ν_{73}	1628	ν_{72}	1545	$sCC_{I,II}$, $sNC_{II,III}$, rCH_{I}
	ν_{74}	1568				ν_{74}	1684			
1626	ν_{75}	1644		1644		ν_{75}	1725		1628	
**Exp. RR FMN S_1**	**OLYP/cc-pVDZ**					**OPBE/cc-pVDZ**				
	**$\nu_{\#}$**	**S_1offR**	**$\nu_{\#}$**	**R_7Res.**	**Assignment**	**$\nu_{\#}$**	**S_1offR**	**$\nu_{\#}$**	**R_7Res.**	**Assignment**
1200	ν_{48}	1146		1175		ν_{49}	1167		1167	
	ν_{50}	1175				-	-			
1250	ν_{51}	1215		1270		ν_{51}	1237	ν_{52}	1251	rCH_{I}, $sNC_{II,III}$, $xCH3_{II,I}$, sCC_{I}
1338	ν_{53}	1270				ν_{53}	1282			
	-	-	ν_{56}	1341	C_{7a}-H_3, C_9-H, C_8-C_9, C_{9a}-C_{5a}, C_{10a}-N_1	-	-		1282	
1381	ν_{57}	-				-	-			
	ν_{58}	-	ν_{60}	1375	N_3-H, $C_{11,7a}$-H_3, N_5-C_{4a}, N_1-C_2, C_2-N_3, C_6-C_{5a}, C_8-C_7	ν_{58}	1359	ν_{60}	1388	$xCH3_{II,I}$, sCC_{I}, $sNC_{II,III}$, rNH_{III}
	ν_{59}	-				ν_{62}	1394			
	ν_{61}	1387				ν_{63}	1408			
1416	ν_{64}	1419		1419		-	-		1446	
	ν_{66}	1420				ν_{66}	1422			
1498	ν_{71}	1519		1519		ν_{71}	1535		1535	
1570	ν_{73}	1605		1605		ν_{73}	1626	ν_{72}	1556	$sCC_{I,II}$, $sNC_{II,III}$, rCH_{I}, sCO_{III}
	ν_{74}	1624				ν_{74}	1652			
1626	ν_{75}	1697		1624		ν_{75}	1725		1626	
**Exp. RR FMN S_1**	**PBE1PBE/cc-pVDZ**					**PW6B95D3/cc-pVDZ**				
	**$\nu_{\#}$**	**S_1offR**	**$\nu_{\#}$**	**R_5Res.**	**Assignment**	**$\nu_{\#}$**	**S_1offR**	**$\nu_{\#}$**	**R_7Res.**	**Assignment**
1200	ν_{49}	1205		1219		ν_{49}	1208		1208	
	ν_{50}	1219				ν_{50}	1222			
1250	ν_{51}	1262		1262		ν_{51}	1257	ν_{52}	1285	rCH_{I}, $xCH3_{II,I}$, sCC_{I}
1338	ν_{53}	1322				ν_{54}	1339			
	ν_{56}	1373	ν_{54}	1339	$sCC_{I,II}$, $sNC_{II,III}$, rCH_{I}, $xCH3_{II,I}$	ν_{56}	1378	ν_{55}	1368	rNH_{III}, $xCH3_{II,I}$, $sCC_{I,II}$, $sNC_{II,III}$
1381	-	-				ν_{58}	1396			
	ν_{58}	1392	ν_{62}	1425	$xCH3_{II,I}$, rNH_{III}, $sNC_{II,III}$, sCC_{I}	ν_{59}	1405		1425	
	ν_{59}	1404				ν_{60}	1425			
	-	-				ν_{62}	1432			
1416	-	-		1451		ν_{65}	1455	ν_{65}	1455	rCH_{I}, $xCH3_{II,I}$, $sCC_{I,II}$, $sNC_{II,III}$
	ν_{65}	1451				ν_{68}	1475			
1498	ν_{71}	1548	ν_{69}	1483	$xCH3_{II,I}$, $sNC_{II,III}$, sCC_{I}	ν_{71}	1549	ν_{72}	1571	$sCC_{I,II}$, $sNC_{II,III}$, rCH_{I}
1570	ν_{73}	1659		1659		ν_{73}	1659		1737	
	ν_{74}	1738				ν_{74}	1737			
1626	ν_{75}	1759		1759		ν_{75}	1757		1757	
**Exp. RR FMN S_1**	**revTPSSh/aug-cc-pVDZ**					**revTPSS/aug-cc-pVDZ**				
	**$\nu_{\#}$**	**S_1offR**	**$\nu_{\#}$**	**R_6Res.**	**Assignment**	**$\nu_{\#}$**	**S_1offR**	**$\nu_{\#}$**	**R_7Res.**	**Assignment**
1200	ν_{51}	1354		1354		ν_{51}	1199	ν_{50}	1167	rCH_{I}, $xCH3_{II}$, rNH_{III}, $sNC_{II,III}$
	-	-				-	-			
1250	ν_{52}	1382		1382		ν_{53}	1265		1265	

1338	-	-				-	-			
	ν_{56}	1491		1491		ν_{56}	1340		1340	
1381	ν_{57}					ν_{57}	1356			
	ν_{58}	1516				ν_{58}	1366		1366	
	ν_{59}	1522		1522		ν_{59}	1371			
	ν_{60}	1526				ν_{60}	1377			
1416	ν_{63}	1574		1574		ν_{63}	1420	ν_{64}	1440	$xCH_{3II,I}$, $sNC_{II,III}$, sCC_{I}
	-	-				-	-			
1498	ν_{71}	1653		1653		ν_{71}	1506		1506	
1570	ν_{73}	1770		1770		ν_{73}	1569		1594	
	ν_{74}	1797				ν_{74}	1594			
1626	ν_{75}	1817		1817		ν_{75}	1627		1627	
**Exp. RR FMN S_1**	**SOGGA11/cc-pVDZ**					**SOGGA11x/cc-pVDZ**				
	**$\nu_{\#}$**	**S_1offR**	**$\nu_{\#}$**	**R_9Res.**	**Assignment**	**$\nu_{\#}$**	**S_1offR**	**$\nu_{\#}$**	**R_5Res.**	**Assignment**
1200	ν_{49}	1229		1200		ν_{49}	1229	ν_{46}	1128	xCH_{3II}, rNH_{III}, rCH_{I}, $sNC_{II,III}$, sCC_{I}
	ν_{50}	1240				ν_{50}	1240			
1250	ν_{51}	1286		1257		ν_{51}	1286		1286	
1338	ν_{53}	1340				ν_{53}	1340			
	ν_{54}	1361		1308		ν_{54}	1361		1361	
1381	ν_{57}	1414				ν_{57}	1414			
	ν_{58}	1422		1344		ν_{58}	1422			
	ν_{59}	1427				ν_{59}	1427		1422	
	ν_{60}	1445				ν_{60}	1445			
1416	ν_{65}	1477		1403		ν_{65}	1477		1477	
	ν_{68}	1498				ν_{68}	1498			
1498	ν_{70}	1561		1500		ν_{70}	1561		1506	
1570	ν_{73}	1679		1587		ν_{73}	1679	ν_{72}	1598	$sNC_{II,III}$, $sCC_{I,II}$, rCH_{I}, rNH_{III}
	ν_{74}	1777		1618		ν_{74}	1777			
1626	ν_{75}	1794		1697		ν_{75}	1794		1794	
**Exp. RR FMN S_1**	**tHCTHhyb/cc-pVDZ**					**TPSSh/cc-pVDZ**				
	**$\nu_{\#}$**	**S_1offR**	**$\nu_{\#}$**	**R_7Res.**	**Assignment**	**$\nu_{\#}$**	**S_1offR**	**$\nu_{\#}$**	**R_7Res.**	**Assignment**
1200	ν_{49}	1177		1187		ν_{49}	1173		1184	
	ν_{50}	1187				ν_{50}	1184			
1250	ν_{51}	1220	ν_{52}	1257	rCH_{I}, $xCH_{3I,II}$, $sCC_{I,II}$	ν_{51}	1220	ν_{52}	1264	C_9-H, C_8-C_7, C_{10a}-C_{4a}, $C_{7a,11}$-H_3, C_{9a}-C_{5a}, N_3-H
1338	-	-				-	-			
	ν_{56}	1355	ν_{55}	1331	rNH_{III}, $sNC_{II,III}$, sCC_{I}, xCH_{3II}	ν_{56}	1369	ν_{55}	1335	N_3-H, N_{10}-C_{10a}, C_7-C_6, C_{9a}-C_{5a}, C_{10a}-C_{4a}, C_{10a}-N_1, C_{11}-H_3
1381	ν_{57}	1367				ν_{57}	1375			
	ν_{58}	1371	ν_{60}	1390	$xCH_{3II,I}$, $sNC_{II,III}$, sCC_{I}	ν_{58}	1383		1383	
	ν_{59}	1379				ν_{60}	1396			
	ν_{61}	1394				ν_{61}	1401			
1416	ν_{64}	1432		1432		ν_{63}	1435		1396	
	ν_{65}	1433				-	-			
1498	ν_{71}	1515		1515		ν_{71}	1526		1526	
1570	ν_{73}	1618		1618		ν_{73}	1621	ν_{72}	1539	C_{10a}-C_{4a}, C_{9a}-C_{5a}, C_8-C_9, C_7-C_6, $C_{6,9}$-H, C_{5a}-N_5, C_4-N_3

	ν_{74}	1687				ν_{74}	1658			
1626	ν_{75}	1721		1687		ν_{75}	1706		1621	
**Exp. RR FMN S$_1$**	**TPSS/cc-pVDZ**					**TPSS/aug-cc-pVDZ**				
	**$\nu_{\#}$**	**S$_1$offR**	**$\nu_{\#}$**	**R$_5$Res.**	**Assignment**	**$\nu_{\#}$**	**S$_1$offR**	**$\nu_{\#}$**	**R$_7$Res.**	**Assignment**
1200	ν_{51}	1221		1221		ν_{51}	1191	ν_{50}	1163	*r*CH$_{I}$, *x*CH$_{3II}$, *r*NH$_{III}$, *s*NC$_{III,II}$
	ν_{52}	1246				-	-			
1250	ν_{54}	1276		1246		ν_{53}	1261	ν_{52}	1247	*r*CH$_{I}$, *s*CC$_{I,II}$, *s*NC$_{II,III}$
1338	-	-				-	-			
	ν_{56}	1330	ν_{55}	1313	*s*NC$_{II,III}$, *s*CC$_{I,II}$, *r*CH$_{I}$, *r*NH$_{III}$	ν_{56}	1336		1336	
1381	ν_{57}	1353				ν_{57}	1351			
	ν_{58}	1367		1367		ν_{59}	1367	ν_{58}	1361	*r*NH$_{III}$, *x*CH$_{3I}$, *s*CC$_{I}$, *s*NC$_{III,II}$
	-	-				ν_{60}	1371			
	ν_{61}	1412				-	-			
1416	-	-		1412		ν_{63}	1414	ν_{64}	1434	*x*CH$_{3II,I}$, *s*CC$_{I,II}$, *s*NC$_{II,III}$
	ν_{64}	1446				-	-			
1498	ν_{71}	1518		1518		ν_{71}	1501		1476	
1570	ν_{73}	1582		1582		ν_{73}	1566		1590	
	ν_{74}	1614				ν_{74}	1590			
1626	ν_{75}	1695		1695		ν_{75}	1623		1623	
**Exp. RR FMN S$_1$**	**VSXC/cc-pVDZ**					**VSXC/aug-cc-pVDZ**				
	**$\nu_{\#}$**	**S$_1$offR**	**$\nu_{\#}$**	**R$_5$Res.**	**Assignment**	**$\nu_{\#}$**	**S$_1$offR**	**$\nu_{\#}$**	**R$_5$Res.**	**Assignment**
1200	ν_{51}	1244	ν_{49}	1152	*s*CC$_{I}$, *r*CH$_{I}$, *x*CH$_{3I}$, *s*NC$_{III,II}$	ν_{49}	1150	ν_{50}	1172	*x*CH$_{3I,II}$, *r*NH$_{III}$, *r*CH$_{I}$, *s*NC$_{II,III}$, *s*CC$_{I}$
	ν_{52}	1259				ν_{51}	1204			
1250	ν_{53}	1282		1244		ν_{53}	1271		1271	
1338	ν_{55}	1328				-	-			
	ν_{56}	1342		1328		ν_{56}	1344	ν_{57}	1347	*r*CH$_{I}$, *r*NH$_{III}$, *s*CC$_{I}$, *s*NC$_{II,III}$
1381	-	-				ν_{58}	1363			
	ν_{58}	1361		1342		ν_{59}	1365		1363	
	ν_{59}	1366				ν_{60}	1368			
	ν_{63}	1417				ν_{61}	1380			
1416	ν_{66}	1442		1417		ν_{64}	1415		1428	
	ν_{68}	1479				ν_{65}	1428			
1498	ν_{71}	1536		1479		ν_{71}	1511	ν_{70}	1481	*x*CH$_{3I,II}$, *s*NC$_{II,III}$, *s*CC$_{I}$
1570	ν_{73}	1612		1612		ν_{73}	1587	ν_{72}	1522	*s*CC$_{I}$, *s*NC$_{II,III}$, *r*CH$_{I}$
	ν_{74}	1635				ν_{74}	1608			
1626	ν_{75}	1725		1725		ν_{75}	1645		1608	
**Exp. RR FMN S$_1$**	**wB97XD/cc-pVDZ**					**X3LYP/cc-pVDZ**				
	**$\nu_{\#}$**	**S$_1$offR**	**$\nu_{\#}$**	**R$_6$Res.**	**Assignment**	**$\nu_{\#}$**	**S$_1$offR**	**$\nu_{\#}$**	**R$_7$Res.**	**Assignment**
1200	ν_{50}	1232		1232		ν_{51}	1232	ν_{49}	1195	*r*CH$_{I}$, *r*NH$_{III}$, *x*CH$_{3I,II}$, *s*NC$_{II,III}$, *s*CC$_{I}$
	-	-				-	-			

1250	v_{53}	1319	v_{52}	1272	rCH_{I}, $sNC_{II,III}$, $xCH_{3I,II}$, rNH_{III}	v_{53}	1298	v_{52}	1276	rCH_{I}, $xCH_{3I,II}$, sCC_{I}
1338	v_{54}	1344		1344		v_{54}	1321			
	-	-				v_{55}	1345		1345	
1381	-	-				v_{57}	1383			
	v_{57}	1398	v_{62}	1440	xCH_{3I}, sNC_{II}	v_{59}	1394		1403	
	v_{58}	1404				v_{60}	1403			
	v_{60}	1426				v_{61}	1410			
1416	v_{64}	1459		1459		v_{64}	1444		1447	
	v_{68}	1484				v_{65}	1447			
1498	v_{72}	1585		1484		v_{71}	1527		1527	
1570	v_{73}	1656		1585		v_{73}	1635		1701	
	v_{74}	1759				v_{74}	1701			
1626	v_{75}	1782		1782		v_{75}	1726		1726	

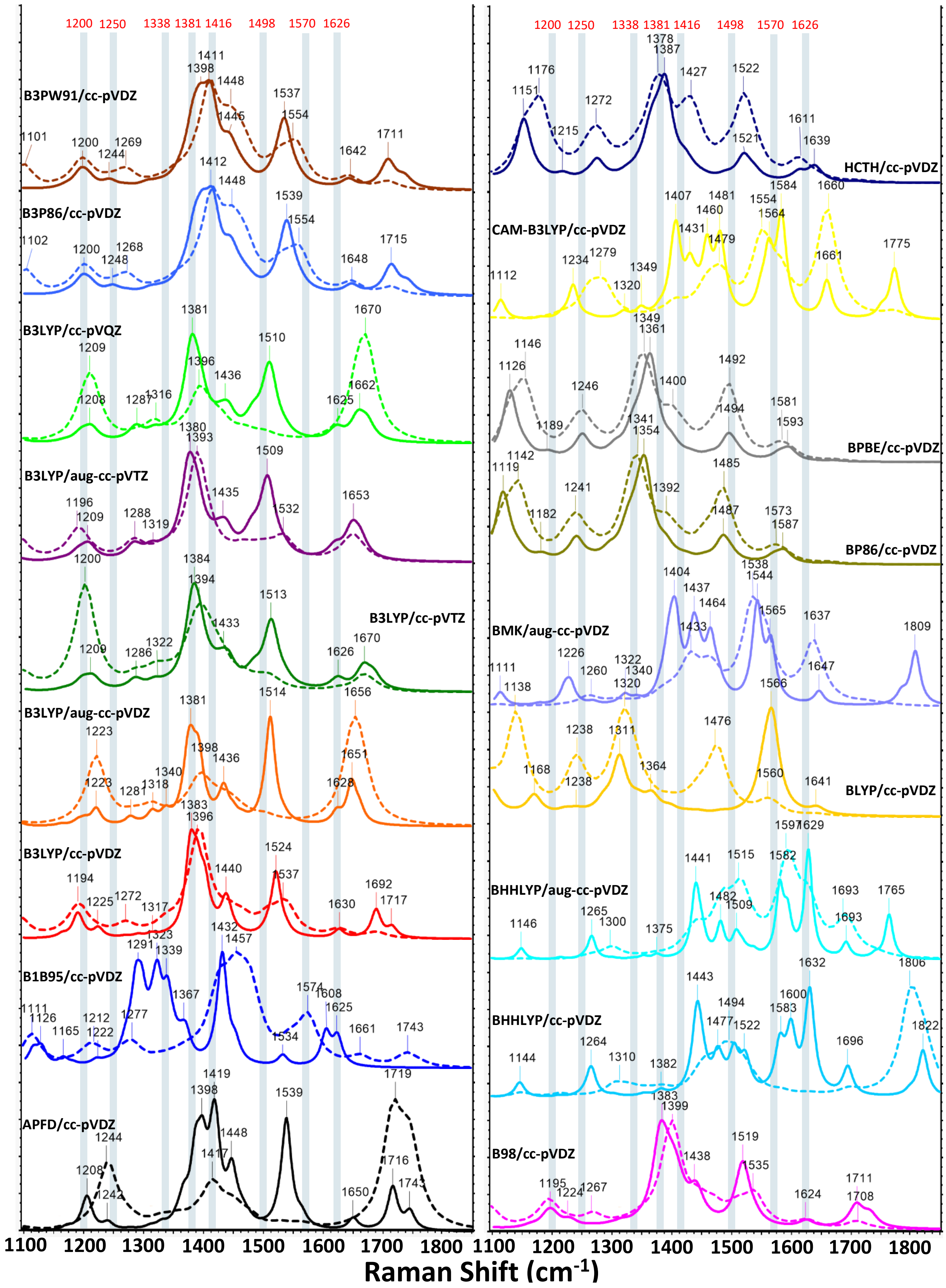

Figure S5 Calculated off-Resonance (solid lines) and Resonance spectra (dashed lines) of the various DFT functionals. Experimental peaks of the 1FMN* 3rd EAS have been labelled and shown as light blue bars. Peak wavenumbers are approximate according to the Spectragryph program[79] that produced the plots, and will differ slightly from the precise values included in **Tables S12-S13**. The intensities of all spectra have been normalized.

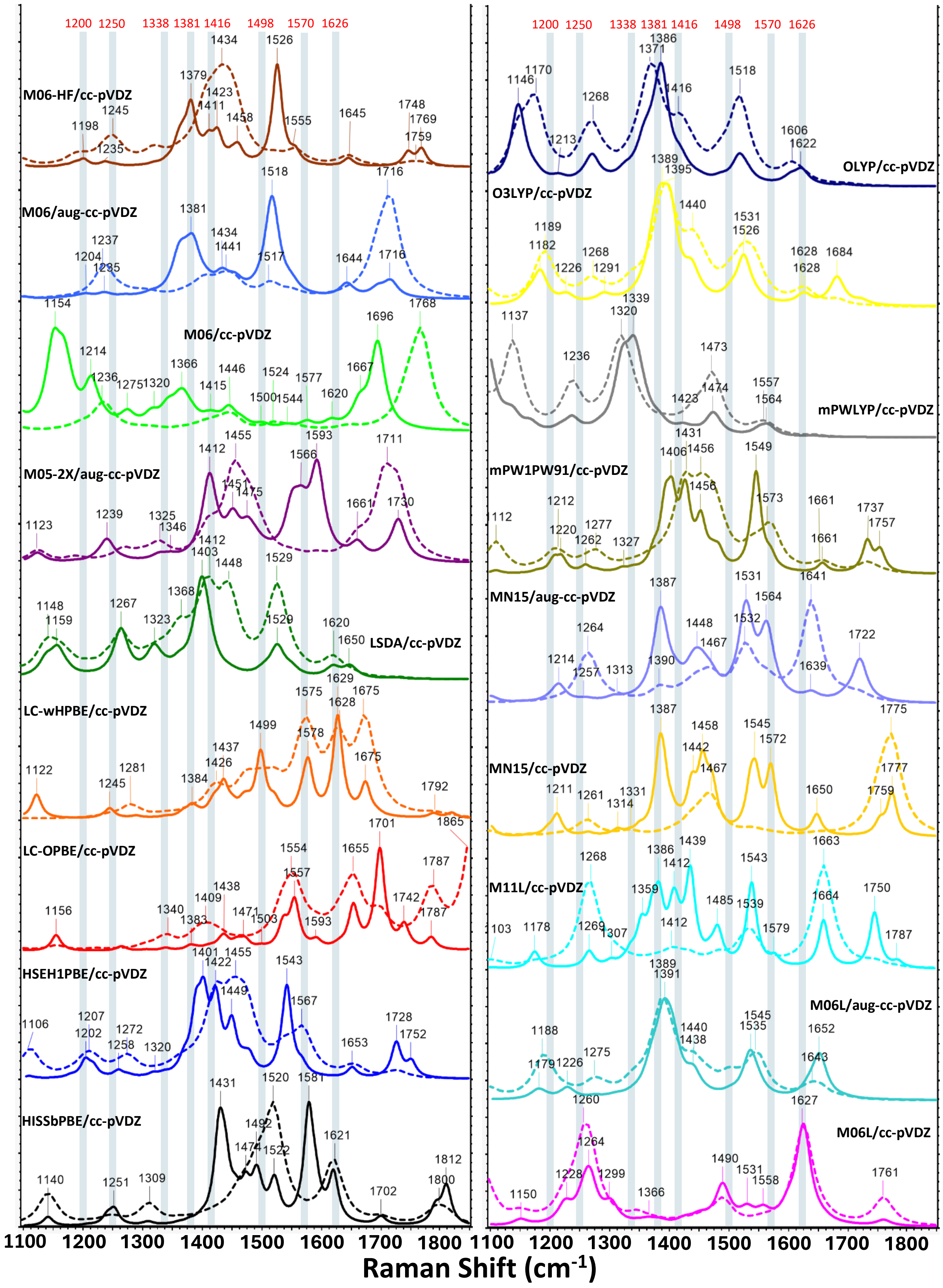

Figure S5 (Cont.)

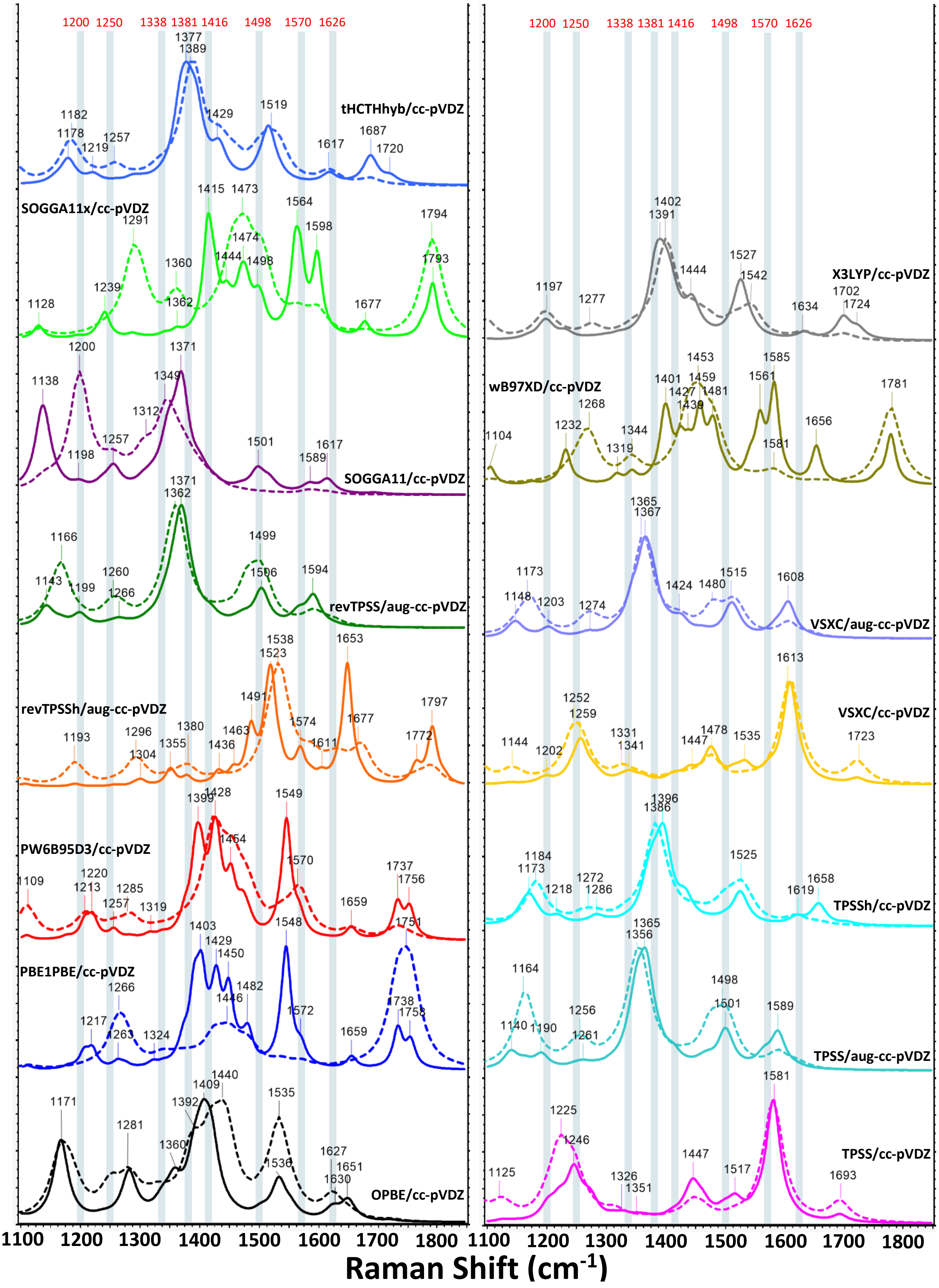

Figure S5 (Cont.)

4. Excited Triplet State of Lumiflavin with selected DFT Functionals

Table S14 Analysis of excitations from the T_1 state of lumiflavin to higher triplet states T_n for the selected functionals. Transition dipole moments (**TDM x, y, z**) are included, as well as energies **EE** in eV and oscillator strengths **Osc.** Additionally, the **$T_1 \rightarrow T_n$** excitation wavelengths and the incident light (**IL**) wavelength used for the pre-resonance calculations are provided.

LOT	Excitation	TDM x	TDM y	TDM z	EE (eV)	Osc.	$T_1 \rightarrow T_n$	IL
BHHLYP/aug-cc-pVDZ	$T_1 \rightarrow T_2$	-0.015	0.010	-0.102	1.91	0.00050	649	737
	$T_1 \rightarrow T_3$	2.608	-1.314	0.201	2.01	0.42205	617	705
BLYP/cc-pVDZ	$T_1 \rightarrow T_6$	0.863	-0.670	0.067	1.68	0.04942	736	824
	$T_1 \rightarrow T_7$	2.514	-1.239	0.192	1.91	0.36969	649	737
BP86/cc-pVDZ	$T_1 \rightarrow T_6$	0.910	-0.666	0.071	1.69	0.05287	733	821
	$T_1 \rightarrow T_7$	2.521	-1.234	0.192	1.91	0.37027	649	737
BPBE/cc-pVDZ	$T_1 \rightarrow T_6$	0.963	-0.688	0.076	1.70	0.05846	731	819
	$T_1 \rightarrow T_7$	2.506	-1.227	0.191	1.91	0.36621	649	737
HCTH/407/cc-pVDZ	$T_1 \rightarrow T_6$	1.110	0.260	0.000	1.72	0.05488	719	807
	$T_1 \rightarrow T_8$	2.600	0.896	0.000	1.94	0.35906	640	728
mPWLYP/cc-pVDZ	$T_1 \rightarrow T_6$	1.061	0.152	0.000	1.68	0.04736	737	825
	$T_1 \rightarrow T_7$	2.651	0.943	0.000	1.91	0.37101	648	736
O3LYP/cc-pVDZ	$T_1 \rightarrow T_4$	0.914	0.214	0.074	1.62	0.03522	764	852
OLYP/cc-pVDZ	$T_1 \rightarrow T_6$	1.011	-0.599	0.080	1.74	0.05912	713	801
	$T_1 \rightarrow T_7$	2.324	-1.139	0.210	1.94	0.32103	638	726
OPBE/cc-pVDZ	$T_1 \rightarrow T_6$	1.099	-0.608	0.087	1.75	0.06789	709	797
	$T_1 \rightarrow T_7$	2.444	-1.190	0.197	1.94	0.35342	639	727
revTPSS/aug-cc-pVDZ	$T_1 \rightarrow T_5$	0.303	0.778	0.001	1.41	0.02411	879	967
	$T_1 \rightarrow T_6$	1.672	0.336	-0.001	1.79	0.12724	694	782
	$T_1 \rightarrow T_7$	2.481	1.114	0.000	1.95	0.35236	637	725
tHCTHhyb/cc-pVDZ	$T_1 \rightarrow T_4$	0.479	0.767	0.000	1.73	0.03459	718	806
	$T_1 \rightarrow T_6$	1.512	0.324	0.000	1.86	0.10872	668	756
TPSSh/cc-pVDZ	$T_1 \rightarrow T_4$	0.568	0.839	0.000	1.62	0.04088	763	851
	$T_1 \rightarrow T_6$	1.882	0.436	0.000	1.86	0.16985	667	755

Table S15 Various hole/electron properties of all computed triplet excited states for the selected DFT functionals. **D** gives the distance of the centroids of the hole and electron in Å, **Sr** is the integral of hole and electron with 1 signifying perfect match, **H** is the overall measure of spatial distribution of the hole and electron in Å, **τ** is the measure of hole-electron separation in the CT direction, $\mathbf{E_{coul}}$ is the coulomb attractive energy between hole and electron, **HDI**/**EDI** are hole and electron delocalization indexes, respectively, **EE** the excitation energy in eV (after optimization) and **Ex.** is the description of the excitation. Information about state and hole-electron properties is provided by the Multiwfn program.[77]

LOT	Excitation	D (Å)	Sr (a.u.)	H (Å)	t (Å)	E_{coul} (eV)	HDI	EDI	EE (eV)	Ex.
BHHLYP/aug-cc-pVDZ	$T_1 \rightarrow T_2$	0.575	0.451	2.504	-0.867	6.01	15.21	8.32	0.94	nπ*
BLYP/cc-pVDZ	$T_1 \rightarrow T_6$	1.170	0.734	2.898	-0.798	5.08	8.78	7.75	1.25	ππ*
	$T_1 \rightarrow T_7$	0.840	0.895	3.244	-1.438	4.72	6.91	7.90	1.71	ππ*
BP86/cc-pVDZ	$T_1 \rightarrow T_6$	1.166	0.733	2.896	-0.800	5.08	8.72	7.70	1.26	ππ*
	$T_1 \rightarrow T_7$	0.763	0.895	3.247	-1.514	4.72	6.84	7.80	1.71	ππ*
BPBE/cc-pVDZ	$T_1 \rightarrow T_6$	1.168	0.734	2.893	-0.795	5.09	8.73	7.71	1.27	ππ*
	$T_1 \rightarrow T_7$	0.758	0.894	3.244	-1.513	4.73	6.85	7.80	1.71	ππ*
HCTH/407/cc-pVDZ	$T_1 \rightarrow T_6$	1.279	0.735	2.889	-1.004	5.37	8.68	7.69	1.30	ππ*
	$T_1 \rightarrow T_8$	0.604	0.892	3.214	-2.248	5.19	6.91	7.77	1.73	ππ*
mPWLYP/cc-pVDZ	$T_1 \rightarrow T_6$	1.183	0.734	2.898	-1.082	5.38	8.77	7.76	1.24	ππ*
	$T_1 \rightarrow T_7$	0.845	0.895	3.246	-2.030	5.12	6.91	7.91	1.71	ππ*
O3LYP/cc-pVDZ	$T_1 \rightarrow T_4$	3.089	0.432	2.644	1.541	4.19	16.73	9.11	0.91	CT
OLYP/cc-pVDZ	$T_1 \rightarrow T_6$	1.126	0.732	2.873	-0.824	5.13	8.73	7.75	1.30	ππ*
	$T_1 \rightarrow T_7$	0.728	0.894	3.216	-1.528	4.77	6.87	7.75	1.75	ππ*
OPBE/cc-pVDZ	$T_1 \rightarrow T_6$	1.131	0.731	2.869	-0.814	5.13	8.68	7.71	1.32	ππ*
	$T_1 \rightarrow T_7$	0.648	0.892	3.217	-1.602	4.77	6.82	7.67	1.74	ππ*
revTPSS/aug-cc-pVDZ	$T_1 \rightarrow T_5$	3.364	0.439	2.750	1.298	4.36	16.05	8.69	0.80	CT
	$T_1 \rightarrow T_6$	1.008	0.748	2.951	-1.135	5.24	8.53	7.38	1.39	ππ*
	$T_1 \rightarrow T_7$	1.858	0.694	3.002	-0.569	5.00	6.92	9.38	3.49	ππ*
tHCTHhyb/cc-pVDZ	$T_1 \rightarrow T_4$	3.007	0.448	2.619	1.086	4.65	16.39	8.99	1.02	CT
	$T_1 \rightarrow T_6$	3.225	0.383	2.605	1.321	-	16.12	8.68	1.02	CT
TPSSh/cc-pVDZ	$T_1 \rightarrow T_4$	2.206	0.606	2.774	0.322	4.76	8.41	9.15	0.36	ππ*
	$T_1 \rightarrow T_6$	3.014	0.451	2.637	1.467	4.25	16.11	9.13	0.94	CT

Table S16 Hole-Electron distribution surfaces of the $T_1 \rightarrow T_n$ excitations for the selected DFT functionals. The hole surfaces are depicted in blue colour while green is the position of the electron typically overlapping with a combination of π* SUMOs of lumiflavin. Typical electron-accepting canonical SUMOs are displayed in the last row of the table for BP86/cc-pVDZ. All hole/electron figures were produced with the Multiwfn program.[77] Three main types of states can be clearly distinguished: (i) ππ* (*i.e.* HCTH and BPBE T_6 states) (ii) intramolecular "Charge transfer" type (*i.e.* O3LYP T_4, TPSSh T_6) and (iii) nπ* (BHHLYP T_2). It can be seen that for tHCTHhyb/cc-pVDZ, $T_1 \rightarrow T_4$ and $T_1 \rightarrow T_6$ are practically identical.

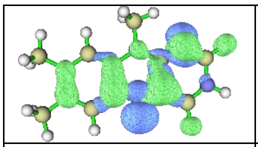	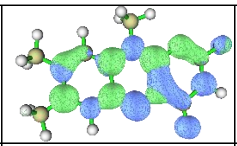	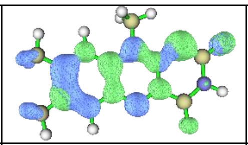	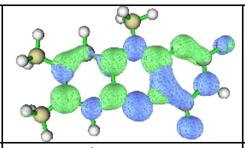	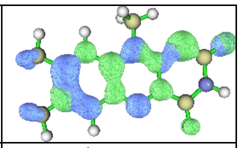
**BHHLYP/aug-cc-pVDZ $T_1 \rightarrow T_2$**	**BLYP/cc-pVDZ $T_1 \rightarrow T_6$**	**BLYP/cc-pVDZ $T_1 \rightarrow T_7$**	**BP86/cc-pVDZ $T_1 \rightarrow T_6$**	**BP86/cc-pVDZ $T_1 \rightarrow T_7$**
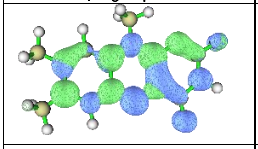	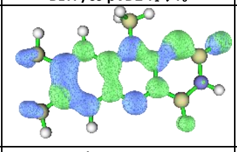	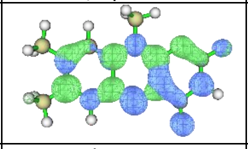	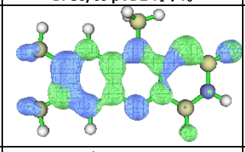	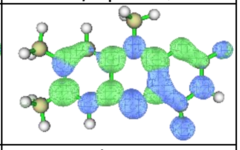
**BPBE/cc-pVDZ $T_1 \rightarrow T_6$**	**BPBE/cc-pVDZ $T_1 \rightarrow T_7$**	**HCTH/cc-pVDZ $T_1 \rightarrow T_6$**	**HCTH/cc-pVDZ $T_1 \rightarrow T_8$**	**MPWLYP/cc-pVDZ $T_1 \rightarrow T_6$**
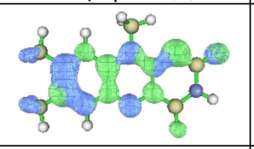	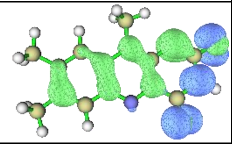	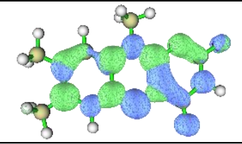	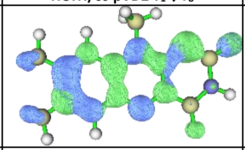	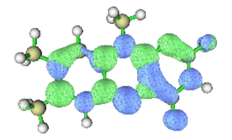
**MPWLYP/cc-pVDZ $T_1 \rightarrow T_7$**	**O3LYP/cc-pVDZ $T_1 \rightarrow T_4$**	**OLYP/cc-pVDZ $T_1 \rightarrow T_6$**	**OLYP/cc-pVDZ $T_1 \rightarrow T_7$**	**OPBE/cc-pVDZ $T_1 \rightarrow T_6$**
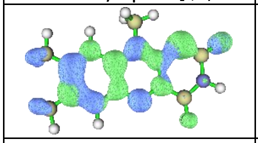	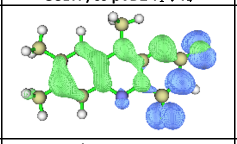	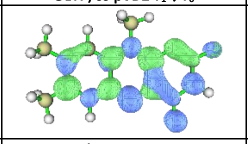	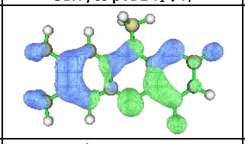	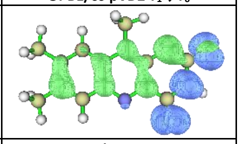
**OPBE/cc-pVDZ $T_1 \rightarrow T_7$**	**revTPSS/aug-cc-pVDZ $T_1 \rightarrow T_5$**	**revTPSS/aug-cc-pVDZ $T_1 \rightarrow T_6$**	**revTPSS/aug-cc-pVDZ $T_1 \rightarrow T_7$**	**tHCTHhyb/cc-pVDZ $T_1 \rightarrow T_4$**
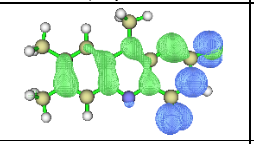	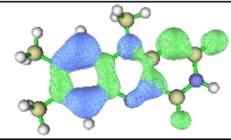	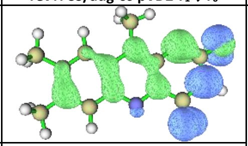		
**tHCTHhyb/cc-pVDZ $T_1 \rightarrow T_6$**	**TPSSh/cc-pVDZ $T_1 \rightarrow T_4$**	**TPSSh/cc-pVDZ $T_1 \rightarrow T_6$**		
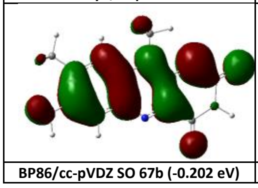	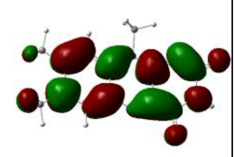	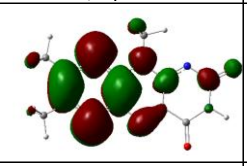	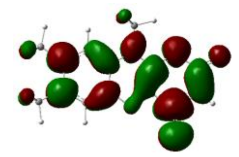	
**BP86/cc-pVDZ SO 67b (-0.202 eV)**	**BP86/cc-pVDZ SO 69a (-0.080)**	**BP86/cc-pVDZ SO 70a (-0.052)**	**BP86/cc-pVDZ SO 71a (-0.040)**	

Table S17 Assignment Tables between the experimental FSRS 5th EAS assigned to 3FMN* (**Exp. FSRS**)[61,76] and the calculated first excited triplet state (T_1) off-Resonance spectra of the subset of twelve DFT functionals (**T_1offR**), including vibration numbers **$\nu_\#$** and **Assignment** of each vibration to normal modes. The numbering of atoms is taken from **Scheme 1** of the main text. The symmetric C=O stretch is signified as (**s**). Normal modes are ordered from the ones containing the largest to the smallest displacement vectors.

**Exp. RR FMN T_1**	**BHHLYP/aug-cc-pVDZ**			**BLYP/cc-pVDZ**		
	**$\nu_\#$**	**T_1offR**	**Assignment**	**$\nu_\#$**	**T_1offR**	**Assignment**
1190	ν_{47}	1173	C_9-C_{9a}, C_2-N_3	ν_{50}	1164	C_2-N_3, $C_{6,9}-H$, $C_{10a}-C_{4a}$, $C_{11}-H_3$, C_6-C_{5a}, C_9-C_{9a}
1269	ν_{50}	1254	$C_{6,9}-H$, N_3-H, C_8-C_7, $C_{11}-H_3$, C_4-N_3, $N_{10}-C_{10a}$	ν_{52}	1220	$C_{6,9}-H$, C_8-C_7, C_2-N_3, $C_{5a}-N_5$
1391	ν_{55}	1407	C_8-C_7, $C_{9a}-C_{5a}$, $C_{6,9}-H$, $C_{7a,8a,11}-H_3$, $C_{10a}-C_{4a}$	ν_{55}	1301	$C_{10a}-N_1$, C_4-N_3, N_3-H, N_5-C_{4a}, C_7-C_6, C_8-C_9, $C_{9a}-C_{5a}$, $C_{11}-H_3$
	ν_{62}	1491	$C_{11,8a,7a}-H_3$, $C_{9a}-N_{10}$, $C_{10a}-N_1$, N_5-C_{4a}	ν_{56}	1326	N_3-H,$C_{9a}-N_{10}$,$C_{5a}-N_5$,$C_{11}-H_3$,C_7-C_6,$C_{10a}-N_1$
	ν_{67}	1526	$C_{7a,11}-H_3$, $C_{6,9}-H$, $C_{10a}-C_{4a}$, C_6-C_{5a}, C_7-C_6	ν_{58}	1339	$C_{7a}-H_3$, $C_{9a}-C_{5a}$, $C_{10a}-C_{4a}$, N_3-H
	ν_{69}	1539	$C_{11}-H_3$, $N_{10}-C_{10a}$, N_5-C_{4a}	ν_{60}	1350	$C_{7a}-H_3$, N_3-H, N_5-C_{4a}, N_3-C_2
1514	ν_{71}	1593	C_7-C_6, C_8-C_9, $C_{9a}-N_{10}$, N_5-C_{4a}, $C_{6,9}-H$, $C_{10a}-C_{4a}$	ν_{72}	1473	C_7-C_6, C_8-C_9, $C_{9a}-N_{10}$, N_5-C_{4a}, $C_{6,9}-H$, $C_{10a}-C_{4a}$
1626	ν_{75}	1771	(s) $C_2-O_{2'}$, $C_4-O_{4'}$, N_3-H, $C_{10a}-C_{4a}$	ν_{75}	1628	(s) $C_2-O_{2'}$, $C_4-O_{4'}$, N_3-H, $C_{10a}-C_{4a}$
**Exp. RR FMN T_1**	**BP86/cc-pVDZ**			**BPBE/cc-pVDZ**		
	**$\nu_\#$**	**T_1offR**	**Assignment**	**$\nu_\#$**	**T_1offR**	**Assignment**
1190	ν_{50}	1175	$C_{6,9}-H$, N_3-C_2, $C_{4a}-C_4$, C_9-C_{9a}, C_6-C_{5a}, $C_{10a}-N_1$	ν_{50}	1180	C_6-H, $C_{10a}-C_{4a}$, N_3-C_2, $C_{5a}-C_{9a}$, C_8-C_7
1269	ν_{53}	1236	$C_{6,9}-H$, $N_{10}-C_{10a}$, C_8-C_7, $C_{11}-H_3$	ν_{53}	1242	C_9-H, $C_{11}-H_3$, $N_{10}-C_{10a}$, N_3-H, N_5-C_{4a}, C_3-N_3, $C_{9a}-C_{5a}$, C_8-C_7
1391	ν_{55}	1317	$C_{7a,11}-H_3$, N_3-H, $C_{10a}-N_1$,C_8-C_9, C_4-N_3, N_5-C_{4a}	ν_{55}	1323	$C_{7a}-H_3$, N_3-H, C_4-N_3, $C_{10a}-N_1$, C_8-C_9
	ν_{57}	1332	$C_{11}-H_3$, N_3-H, C_6-H, $C_{9a}-N_{10}$, C_7-C_6, C_6-C_{5a}, C_4-N_3	ν_{57}	1338	N_3-H, $C_{11}-H_3$, C_6-H, $C_{9a}-N_{10}$, C_6-C_{5a}, C_4-N_3, C_7-C_6, $C_{5a}-N_5$
	ν_{59}	1347	$C_{8a}-H_3$, N_3-H, C_6-C_{5a}, C_8-C_7, $C_{9a}-C_{5a}$, C_2-N_3	ν_{59}	1354	$C_{8a}-H_3$, N_3-H, $C_{9,6}-H$, C_6-C_{5a}, C_8-C_7, $C_{9a}-C_{5a}$, C_2-N_3, $C_{10a}-C_{4a}$
	ν_{62}	1376	$C_{7a}-H_3$, N_5-C_{4a}, N_3-H, $C_{9a}-C_{5a}$, C_8-C_7, $C_{4a}-C_4$, $C_{10a}-N_1$	ν_{60}	1383	$C_{11,7a}-H_3$, N_3-H, $C_{10a}-N_1$, N_5-C_{4a}, C_9-C_{9a}
1514	ν_{72}	1492	N_5-C_{4a}, $C_{10a}-N_1$, $C_{9a}-N_{10}$, C_9-H, C_4-N_3, N_3-H, C_8-C_9, C_6-C_{5a}, $C_{6,9}-H$	ν_{72}	1500	N_5-C_{4a}, $C_{10a}-N_1$, $C_{9a}-N_{10}$, C_9-H, C_4-N_3, N_3-H, C_8-C_9, C_6-C_{5a}, $C_{6,9}-H$
1626	ν_{75}	1652	(s) $C_2-O_{2'}$, $C_4-O_{4'}$, N_3-H, $C_{10a}-C_{4a}$	ν_{75}	1659	(s) $C_2-O_{2'}$, $C_4-O_{4'}$, N_3-H, $C_{10a}-C_{4a}$
**Exp. RR FMN T_1**	**HCTH/407/cc-pVDZ**			**mPWLYP/cc-pVDZ**		
	**$\nu_\#$**	**T_1offR**	**Assignment**	**$\nu_\#$**	**T_1offR**	**Assignment**
1190	ν_{50}	1204	C_6-H, C_7-C_6, C_2-N_3, $C_{10a}-C_{4a}$, C_6-C_{5a}	ν_{50}	1163	C_2-N_3, $C_{4a}-C_{10a}$, $C_{9,6}-H$, $C_{9a}-C_{5a}$
1269	ν_{53}	1268	$C_{6,9}-H$, $C_{11}-H_3$, N_5-C_{4a}, $N_{10}-C_{10a}$, C_2-N_3, N_3-H	ν_{52}	1218	$N_{10}-C_{10a}$, $C_{11}-H_3$, N_3-H, N_5-C_{4a}, C_8-C_7, C_6-H
1391	ν_{55}	1343	$C_{7a}-H_3$, C_9-H, C_8-C_7, $C_{9a}-C_{5a}$	ν_{55}	1299	$C_{10a}-N_1$, N_3-H, C_4-N_3, N_5-C_4, C_8-C_9, C_7-C_6, $C_{9a}-C_{5a}$, $C_{11}-H_3$
	ν_{56}	1355	N_3-H, $C_{8a,11,7a}-H_3$, $C_{10a}-N_1$, C_7-C_6,C_4-N_3,C_8-C_9	ν_{56}	1325	N_3-H, C_7-C_6, $C_{5a}-N_5$,$C_{11,7a}-H_3$,$C_{9a}-N_{10}$,$C_{10a}-N_1$
	ν_{58}	1367	N_3-H, $C_{11,8a,7a}-H_3$, C_6-C_{5a},C_6-H, $C_{9a}-N_{10}$,C_2-N_3	ν_{58}	1336	$C_{7a}-H_3$, $C_{9a}-N_{10}$, $C_{10a}-C_{4a}$
	ν_{59}	1381	N_3-H, $C_{5a}-C_{9a}$, C_8-C_7, C_6-C_{5a}, $C_{6,9}-H$, $C_{8a,7a}-H_3$	ν_{60}	1349	$C_{8a}-H_3$, N_3-H, N_5-C_{4a}, C_2-N_3
1514	ν_{72}	1536	$C_{10a}-N_1$, N_5-C_{4a}, C_4-N_3, N_3-H, C_8-C_9	ν_{72}	1471	N_5-C_{4a}, $C_{9a}-N_{10}$, $C_{10a}-C_{4a}$, $C_{6,9}-H$, C_7-C_6, C_8-C_9
1626	ν_{75}	1698	(s) $C_2-O_{2'}$, $C_4-O_{4'}$, N_3-H, $C_{10a}-C_4$	ν_{75}	1630	(s) $C_2-O_{2'}$, $C_4-O_{4'}$, N_3-H, $C_{10a}-C_{4a}$
**Exp. RR FMN T_1**	**O3LYP/cc-pVDZ**			**OLYP/cc-pVDZ**		
	**$\nu_\#$**	**T_1offR**	**Assignment**	**$\nu_\#$**	**T_1offR**	**Assignment**
1190	ν_{50}	1208	C_6-H, C_2-N_3, N_5-C_{4a}, C_8-C_7, C_6-C_{5a}	ν_{50}	1202	C_6-H, $C_{10a}-C_{4a}$, C_2-N_3, C_9-C_{9a}, $C_{7a,8a,11}-H_3$
1269	ν_{53}	1276	$C_{6,9}-H$, $C_{10a}-C_{4a}$, C_8-C_7, N_3-H, C_2-N_3	ν_{53}	1267	$C_{6,9}-H$, $N_{10}-C_{10a}$, $C_{11}-H_3$, N_3-H, $C_{4a}-C_4$, N_5-C_{4a}
1391	ν_{55}	1360	$C_{7a,8a}-H_3$,C_9-H, C_8-C_7, $C_{10a}-N_1$, $C_{9a}-C_{5a}$, $C_{4a}-C_4$	ν_{55}	1341	$C_{7a}-H_3$,C_9-H, C_8-C_7, $C_{9a}-C_{5a}$
	ν_{58}	1380	N_3-H, $C_{11a,7a,8a}-H_3$, C_6-C_{5a}, $C_{9a}-N_{10}$, C_7-C_6	ν_{58}	1362	N_3-H, $C_{8a,7a}-H_3$, C_7-C_6, C_6-C_{5a}, $C_{9a}-N_{10}$, C_2-N_3
	ν_{59}	1389	N_3-H, $C_{8a}-H_3$, C_8-C_7, $C_{9a}-C_{5a}$, C_6-C_{5a}, C_2-N_3	ν_{59}	1378	N_3-H, $C_{8a,7a}-H_3$, C_8-C_7, $C_{6,9}-H$, C_6-C_{5a}, $C_{5a}-C_{9a}$

	ν_{60}	1405	$C_{11a,7a,8a}$-H_3, C_9-C_{9a}, N_3-H, C_{10a}-N_1	ν_{60}	1388	$C_{11,7a}$-H_3, N_3-H, C_{10a}-N_1, N_5-C_{4a}, C_9-C_{9a}
1514	ν_{72}	1545	N_5-C_{4a}, C_{10a}-N_1, C_{9a}-N_{10}, C_9-H, N_3-H, C_8-C_9	ν_{72}	1525	N_1-C_{10a}, C_{10a}-C_{4a}, C_{4a}-N_5,$C_{6,9}$-H,C_8-C_9,C_4-N_3
1626	ν_{75}	1720	(s) C_2-$O_{2'}$, C_4-$O_{4'}$, N_3-H, C_{10a}-C_{4a}	ν_{75}	1684	(s) C_2-$O_{2'}$, C_4-$O_{4'}$, N_3-H
**Exp. RR FMN T_1**	**OPBE/cc-pVDZ**			**tHCTHhyb/cc-pVDZ**		
	**$\nu_\#$**	**T_1offR**	**Assignment**	**$\nu_\#$**	**T_1offR**	**Assignment**
1190	ν_{50}	1206	C_6-H, C_8-C_7, N_1-C_2, C_9-C_{9a}, C_{10a}-C_{4a}, C_2-N_3	ν_{50}	1196	C_6-H, C_{5a}-N_5, C_{4a}-C_4, C_2-N_3, C_{10a}-C_{4a}, N_3-H
1269	ν_{53}	1283	N_{10}-C_{10a}, N_5-C_{4a}, C_6-H, N_3-H, C_{11}-H_3, C_8-C_7	ν_{53}	1263	$C_{6,9}$-H, C_{10a}-C_{4a}, C_8-C_7, N_3-H, N_1-C_2
1391	-	-	-	ν_{55}	1353	C_{7a}-H_3, C_9-H, C_8-C_7, C_{9a}-C_{5a},C_{10a}-N_1, C_4-N_3, C_2-N_3, N_3-H
	ν_{57}	1358	C_{11}-H_3, C_{10a}-N_1, C_8-C_9, C_4-N_3, N_3-H	ν_{57}	1370	$C_{8a,11}$-H_3, C_{5a}-C_6, C_8-C_7, C_{9a}-N_{10}, C_{10a}-N_1
	ν_{64}	1422	$C_{8a,C7a}$-H_3, C_{10a}-C_{4a}, C_2-N_3, C_{4a}-C_4	ν_{58}	1374	C_{8a}-H_3, N_3-H, C_7-C_6, C_{9a}-C_{5a}, C_4-N_3, C_{10a}-N_1
	-	-	-	ν_{60}	1399	$C_{11a,7a}$-H_3, C_9-C_{9a}, C_{10a}-N_1, N_{10}-C_{10a}, C_{5a}-N_5
1514	ν_{72}	1556	C_{10a}-N_1, N_5-C_{4a}, C_8-C_9, C_4-N_3, C_8-C_9, N_3-H	ν_{72}	1531	N_5-C_{4a}, C_{10a}-N_1, C_{9a}-N_{10}, C_9-H, C_4-N_3, N_3-H, C_8-C_9
1626	ν_{75}	1714	(s) C_2-$O_{2'}$, C_4-$O_{4'}$, N_3-H, C_{10a}-C_{4a}	ν_{75}	1717	(s) C_2-$O_{2'}$, C_4-$O_{4'}$, N_3-H, C_{10a}-C_{4a}
**Exp. RR FMN T_1**	**revTPSS/aug-cc-pVDZ**			**TPSSh/cc-pVDZ**		
	**$\nu_\#$**	**T_1offR**	**Assignment**	**$\nu_\#$**	**T_1offR**	**Assignment**
1190	ν_{50}	1191	C_6-H, C_2-N_3, C_{10a}-C_{4a}, C_{4a}-C_4, C_6-C_{5a}	ν_{50}	1202	C_6-H, C_2-N_3, C_{4a}-C_4, C_{5a}-N_5, C_{10a}-C_{4a}
1269	ν_{52}	1251	$C_{9,6}$-H, C_2-N_3, C_8-C_7, C_{5a}-N_5, C_{7a}-H_3	ν_{52}	1256	C_{11}-H_3, N_3-H, N_{10}-C_{4a}, C_8-C_7, C_9-C_{9a}, N_5-C_{4a}, C_2-N_3
1391	ν_{55}	1332	N_3-H, C_{10a}-N_1, C_4-N3, C_7-C_6, C_8-C_9, C_{9a}-C_{5a}, C_{11}-H_3	ν_{55}	1354	N_3-H, C_{10a}-N_1, C_8-C_9, N_5-C_{4a}, C_4-N_3, C_7-C_6, C_4-C_{4a}
	ν_{56}	1348	N_3-H, C_{9a}-N_{10}, C_7-C_6, C_{9a}-C_{5a}, C_4-N_3, C_{11}-H_3	ν_{56}	1374	C_6-H, $C_{7a,11}$-H_3, C_6-C_{5a}, C_8-C_7, C_{9a}-N_{10}, N_3-H
	ν_{58}	1376	C_{7a}-H_3, N_3-H, N_5-C_{4a}, C_{10a}-N_1, C_9-C_{9a}	ν_{59}	1397	C_{7a}-H_3, N_3-H, C_6-C_{5a}, C_2-N_3
	ν_{60}	1386	C_{8a}-H_3, C_9-C_{9a}, N_5-C_{4a}, C_2-N_3	ν_{61}	1410	$C_{8a,7a}$-H_3, N_3-H, C_9-C_{9a}, N_1-C_2, C_{4a}-C_4, C_{9a}-C_{5a}, C_{10a}-C_{4a}
1514	ν_{70}	1487	C_7-C_6, C_8-C_9, C_{9a}-N_{10}, N_5-C_{4a}, $C_{6,9}$-H, C_{10a}-C_{4a}	ν_{72}	1529	N_5-C_{4a}, C_{10a}-N_1, C_{9a}-N_{10}, C_9-H, C_4-N_3, N_3-H, C_8-C_9, C_6-C_{5a}
1626	ν_{75}	1617	(s) C_2-$O_{2'}$, C_4-$O_{4'}$, N_3-H, C_{10a}-C_{4a}	ν_{75}	1699	(s) C_2-$O_{2'}$, C_4-$O_{4'}$, N_3-H, C_{10a}-C_{4a}

Table S18 Assignment Tables between the experimental FSRS 5th EAS assigned to 3FMN* (**Exp. FSRS**)[61,76] and the calculated excited triplet state pre-Resonance and Resonance Raman spectra of each of the selected DFT functionals (**Pre-T_n** and **FC/FCHT-T_n**). The off-Resonance assignment is shown with black numbers (**T_1offR**) and any new assignment due to the new rR intensities is highlighted in red and the vibration number is given in the **$\nu_\#$** column. For those vibrations that assignment to normal modes is missing in **Table S17**, assignments have been inserted according to the notation introduced in **Table 1** of the main text. Normal modes are ordered from the ones containing the largest to the smallest displacement vectors.

**Exp. RR FMN T_1**	**BHHLYP/aug-cc-pVDZ**																							
	**$\nu_\#$**	**T_1offR**	**$\nu_\#$**	**Pre-T_2**	**Assignment**	**$\nu_\#$**	**Pre-T_3**	**Assignment**	**$\nu_\#$**	**FC-T_2**	**Assignment**	**$\nu_\#$**	**FC-T_3**	**Assignment**	**$\nu_\#$**	**FCHT-T_2**	**Assignment**	**$\nu_\#$**	**FCHT-T_3**	**Assignment**				
1190	ν_{47}	1173	ν_{47}	1173		ν_{47}	1173		ν_{47}	1173		-	-	-	ν_{47}	1173		-	-	-				
1269	ν_{50}	1254	ν_{50}	1254		ν_{50}	1254		ν_{50}	1254		-	-	-	ν_{50}	1254		-	-	-				
1391	ν_{55}	1407	ν_{56}	1428	rNH$_{III}$, sNC$_{III}$, sCC$_I$	ν_{56}	1428		ν_{54}	1352	sCC$_I$, sNC$_{II}$, xCH$_{3I,II}$, rNH$_{III}$	-	-	-	ν_{53}	1336	rCH$_I$, sCC$_I$, sNC$_{II,III}$	-	-	-				
	ν_{62}	1491																						
	ν_{67}	1526																						
	ν_{69}	1539																						
1514	ν_{71}	1593	ν_{69}	1539	xCH$_{3II,I}$, sNC$_{II,III}$		1539		ν_{67}	1526		-	-	-	ν_{67}	1526		-	-	-				
1626	ν_{75}	1771	ν_{71}	1593		ν_{71}	1593		ν_{69}	1539	xCH$_{3II,I}$, sNC$_{II,III}$	-	-	-	ν_{69}	1539	xCH$_{3II,I}$, sNC$_{II,III}$	-	-	-				
**Exp. RR FMN T_1**	**BLYP/cc-pVDZ**																							
	**$\nu_\#$**	**T_1offR**	**$\nu_\#$**	**Pre-T_6**	**Assignment**	**$\nu_\#$**	**Pre-T_7**	**Assignment**	**$\nu_\#$**	**FC-T_6**	**Assignment**	**$\nu_\#$**	**FC-T_7**	**Assignment**	**$\nu_\#$**	**FCHT-T_6**	**Assignment**	**$\nu_\#$**	**FCHT-T_7**	**Assignment**				

1190	ν_{50}	1164	ν_{49}	1144	sNC_{III}, rNH_{III}, xCH_{3II}, rCH_{I}	-	-	-	ν_{50}	1164		ν_{50}	1164		ν_{50}	1164		ν_{50}	1164	
1269	ν_{52}	1220	ν_{53}	1238	rCH_{I}, sCC_{I}, sNC_{III}	-	-	-	ν_{55}	1301		ν_{55}	1301		ν_{52}	1220		ν_{52}	1220	
1391	ν_{55}<br>ν_{56}<br>ν_{58}<br>ν_{60}	1301<br>1326<br>1339<br>1350	ν_{59}	1346	rNH_{III}, $sNC_{III,II}$, sCC_{I}, $xCH_{3I,II}$	-	-	-	ν_{59}	1346	rNH_{III}, $sNC_{III,II}$, sCC_{I}, $xCH_{3I,II}$	ν_{58}	1339		ν_{58}	1339		ν_{58}	1339	
1514	ν_{72}	1473	ν_{72}	1473		-	-	-	ν_{72}	1473		ν_{72}	1473		ν_{70}	1459	sCC_{I}, $sNC_{III,II}$, rCH_{I}, xCH_{3I}	ν_{70}	1459	sCC_{I}, $sNC_{III,II}$, rCH_{I}, xCH_{3I}
1626	ν_{75}	1628	ν_{73}	1556	sCC_{I}, rCH_{I}, sCO_{III}	-	-	-	ν_{73}	1556	sCC_{I}, rCH_{I}, sCO_{III}	ν_{73}	1556	sCC_{I}, rCH_{I}, sCO_{III}	ν_{74}	1591	(as) sCO_{III}, rNH_{III}, sNC_{III}	ν_{73}	1556	sCC_{I}, rCH_{I}, sCO_{III}
**Exp. RR FMN T_1**	**BP86/cc-pVDZ**																			
	$\nu_{\#}$	T_1offR	$\nu_{\#}$	Pre-T_6	Assignment	$\nu_{\#}$	Pre-T_7	Assignment	$\nu_{\#}$	FC-T_6	Assignment	$\nu_{\#}$	FC-T_7	Assignment	$\nu_{\#}$	FCHT-T_6	Assignment	$\nu_{\#}$	FCHT-T_7	Assignment
1190	ν_{50}	1175	ν_{49}	1155	rCH_{I}, rNH_{III}, $sNC_{III,II}$, xCH_{3II}	-	-	-	ν_{50}	1175		ν_{50}	1175		ν_{51}	1195	rCH_{I}, rNH_{III}, sNC_{III}, sCC_{I}	ν_{50}	1175	
1269	ν_{53}	1236	ν_{52}	1225	rCH_{I}, xCH_{3II}, $sNC_{III,II}$	-	-	-	ν_{54}	1270	sNC_{II} $sCC_{I,II}$, rNH_{III}, rCH_{I}	ν_{55}	1317		ν_{53}	1236		ν_{52}	1225	rCH_{I}, xCH_{3II}, $sNC_{III,II}$
1391	ν_{55}<br>ν_{57}<br>ν_{59}<br>ν_{62}	1317<br>1332<br>1347<br>1376	ν_{61}	1368	xCH_{3II}, $sNC_{III,II}$, sCC_{I}	-	-	-	ν_{61}	1368	xCH_{3II}, $sNC_{III,II}$, sCC_{I}	ν_{64}	1391		ν_{61}	1368	xCH_{3II}, $sNC_{III,II}$, sCC_{I}	ν_{59}	1347	
1514	ν_{72}	1492	ν_{72}	1492		-	-	-	ν_{72}	1492		ν_{72}	1492		ν_{72}	1492		ν_{72}	1492	
1626	ν_{75}	1652	ν_{73}	1570	sCC_{I}, rCH_{I}, sNC_{III}, sCO_{III}	-	-	-	ν_{73}	1570	sCC_{I}, rCH_{I}, sNC_{III}, sCO_{III}		1570	sCC_{I}, rCH_{I}, sNC_{III}, sCO_{III}	ν_{74}	1614	(as) sCO_{III}, rNH_{III}, sNC_{III}	ν_{73}	1570	sCC_{I}, rCH_{I}, sNC_{III}, sCO_{III}
**Exp. RR FMN T_1**	**BPBE/cc-pVDZ**																			
	$\nu_{\#}$	T_1offR	$\nu_{\#}$	Pre-T_6	Assignment	$\nu_{\#}$	Pre-T_7	Assignment	$\nu_{\#}$	FC-T_6	Assignment	$\nu_{\#}$	FC-T_7	Assignment	$\nu_{\#}$	FCHT-T_6	Assignment	$\nu_{\#}$	FCHT-T_7	Assignment
1190	ν_{50}	1180	ν_{49}	1161	rCH_{I}, rNH_{III}, $sNC_{III,II}$, sCC_{I}	ν_{50}	1180		ν_{50}	1180		ν_{50}	1180		ν_{50}	1180		ν_{50}	1180	
1269	ν_{53}	1242	ν_{52}	1230	rCH_{I}, $sNC_{II,III}$, xCH_{3II}	ν_{53}	1242		ν_{54}	1276	$sNC_{II,III}$, rNH_{III}, rCH_{I}, xCH_{3I}	ν_{55}	1323		ν_{54}	1276	$sNC_{II,III}$, rNH_{III}, rCH_{I}, xCH_{3I}	ν_{55}	1323	
1391	ν_{55}<br>ν_{57}<br>ν_{59}<br>ν_{60}	1323<br>1338<br>1354<br>1383	ν_{61}	1374	xCH_{3II}, $sNC_{II,III}$	ν_{58}	1340	xCH_{3I}, rNH_{III}, sCC_{I}	ν_{61}	1374	xCH_{3II}, $sNC_{II,III}$	ν_{64}	1397	$xCH_{3I,II}$, rCH_{I}, $sNC_{II,III}$	ν_{61}	1374	xCH_{3II}, $sNC_{II,III}$	ν_{64}	1397	$xCH_{3I,II}$, rCH_{I}, $sNC_{II,III}$
1514	ν_{72}	1500	ν_{72}	1500		ν_{68}	1423	$xCH_{3II,I}$, rCH_{I}, sNC_{II}	ν_{72}	1500		ν_{72}	1500		ν_{72}	1500		ν_{70}	1479	sCC_{I}, rCH_{I}, xCH_{3I}, sNC_{II}
1626	ν_{75}	1659	ν_{73}	1577	sCC_{I}, rCH_{I}, sNC_{II}, xCH_{3I}	ν_{74}	1621	(as) sCO_{III}, rNH_{III}, sNC_{III}	ν_{73}	1577	sCC_{I}, rCH_{I}, sNC_{II}, xCH_{3I}	ν_{73}	1577	sCC_{I}, rCH_{I}, sNC_{II}, xCH_{3I}	ν_{74}	1621	(as) sCO_{III}, rNH_{III}, sNC_{III}	ν_{73}	1577	sCC_{I}, rCH_{I}, sNC_{II}, xCH_{3I}
**Exp. RR FMN T_1**	**HCTH/407/cc-pVDZ**																			
	$\nu_{\#}$	T_1offR	$\nu_{\#}$	Pre-T_6	Assignment	$\nu_{\#}$	Pre-T_8	Assignment	$\nu_{\#}$	FC-T_6	Assignment	$\nu_{\#}$	FC-T_8	Assignment	$\nu_{\#}$	FCHT-T_6	Assignment	$\nu_{\#}$	FCHT-T_8	Assignment
1190	ν_{50}	1204	ν_{49}	1189	rCH_{I}, rNH_{III}, $sNC_{II,III}$, sCC_{I}	ν_{49}	1189	rCH_{I}, rNH_{III}, $sNC_{II,III}$, sCC_{I}	ν_{50}	1204		ν_{50}	1204		ν_{50}	1204		ν_{50}	1204	
1269	ν_{53}	1268	ν_{52}	1252	rCH_{I}, sNC_{II} xCH_{3II}, sCC_{I}	ν_{53}	1268		ν_{52}	1252	rCH_{I}, sNC_{II} xCH_{3II}, sCC_{I}	ν_{52}	1252	rCH_{I}, sNC_{II} xCH_{3II}, sCC_{I}	ν_{52}	1252	rCH_{I}, sNC_{II} xCH_{3II}, sCC_{I}	ν_{52}	1252	rCH_{I}, sNC_{II} xCH_{3II}, sCC_{I}
1391	ν_{55}<br>ν_{56}<br>ν_{58}<br>ν_{59}	1343<br>1355<br>1367<br>1381	ν_{65}	1422	xCH_{3I}, rCH_{I}, sNC_{III}, sCC_{I}	ν_{56}	1355		ν_{61}	1398	xCH_{3II}, $sNC_{II,III}$, sCC_{I}	ν_{59}	1381		ν_{61}	1398	xCH_{3II}, $sNC_{II,III}$, sCC_{I}	ν_{56}	1355	
1514	ν_{72}	1536	ν_{72}	1536		ν_{70}	1505	sCC_{I}, rCH_{I}, sNC_{II}, xCH_{3I}	ν_{72}	1536		ν_{71}	1522	sCC_{I}, rCH_{I}, $sNC_{II,III}$, $xCH_{3I,II}$	ν_{72}	1536		ν_{70}	1505	sCC_{I}, rCH_{I}, sNC_{II}, xCH_{3I}
1626	ν_{75}	1698	ν_{73}	1605	sCC_{I}, rCH_{I}, xCH_{3I}, sNC_{III}		1662		ν_{73}	1605	sCC_{I}, rCH_{I}, xCH_{3I}, sNC_{III}	ν_{73}	1605	sCC_{I}, rCH_{I}, xCH_{3I}, sNC_{III}	ν_{74}	1662	(as) sCO_{III}, rNH_{III}, sNC_{III}	ν_{73}	1605	sCC_{I}, rCH_{I}, xCH_{3I}, sNC_{III}
**Exp. RR FMN T_1**	**mPWLYP/cc-pVDZ**																			
	$\nu_{\#}$	T_1offR	$\nu_{\#}$	Pre-T_6	Assignment	$\nu_{\#}$	Pre-T_7	Assignment	$\nu_{\#}$	FC-T_6	Assignment	$\nu_{\#}$	FC-T_7	Assignment	$\nu_{\#}$	FCHT-T_6	Assignment	$\nu_{\#}$	FCHT-T_7	Assignment
1190	ν_{50}	1163	ν_{49}	1143	rNH_{III}, sNC_{III}, rCH_{I}, xCH_{3II}	ν_{49}	1143	rNH_{III}, sNC_{III}, rCH_{I}, xCH_{3II}	ν_{50}	1163		ν_{51}	1184	rCH_{I}, sCC_{I}, sNC_{III}, $xCH_{3I,II}$	ν_{52}	1218		ν_{50}	1163	
1269	ν_{52}	1218	ν_{53}	1235	rCH_{I}, sCC_{I}, sNC_{III}, $xCH_{3I,II}$	ν_{53}	1235	rCH_{I}, sCC_{I}, sNC_{III}, $xCH_{3I,II}$	ν_{54}	1258	sNC_{II}, rCH_{I}, sCC_{I}, rNH_{III}	ν_{55}	1299		ν_{55}	1299		ν_{55}	1299	
1391	ν_{55}<br>ν_{56}	1299<br>1325	ν_{59}	1345	rNH_{III}, $sNC_{II,III}$, xCH_{3II}, sCC_{I}	ν_{61}	1358	xCH_{3II}, rNH_{III}, sCC_{I}, $sNC_{II,III}$	ν_{59}	1345	rNH_{III}, $sNC_{II,III}$, xCH_{3II}, sCC_{I}	ν_{58}	1336		ν_{59}	1345	rNH_{III}, $sNC_{II,III}$, xCH_{3II}, sCC_{I}	ν_{58}	1336	

	ν_{58}	1336																		
	ν_{60}	1349																		
1514	ν_{72}	1471	ν_{72}	1471		ν_{68}	1420	$xCH_{3I,II}$, sNC_{II}	ν_{72}	1471		ν_{72}	1471		ν_{70}	1458	sCC_{I}, rCH_{I}, $sNC_{II,III}$, xCH_{3I}	ν_{70}	1458	sCC_{I}, rCH_{I}, $sNC_{II,III}$, xCH_{3I}
1626	ν_{75}	1630	ν_{73}	1554	sCC_{I}, rCH_{I}, xCH_{3I}, sCO_{III}	ν_{73}	1554	sCC_{I}, rCH_{I}, xCH_{3I}, sCO_{III}	ν_{73}	1554	sCC_{I}, rCH_{I}, xCH_{3I}, sCO_{III}	ν_{73}	1554	sCC_{I}, rCH_{I}, xCH_{3I}, sCO_{III}	ν_{74}	1592	(as) sCO_{III}, rNH_{III}, sNC_{III}	ν_{73}	1554	sCC_{I}, rCH_{I}, xCH_{3I}, sCO_{III}
**Exp. RR FMN T_1**	**O3LYP/cc-pVDZ**																			
	**$\nu_{\#}$**	**T_1offR**	**$\nu_{\#}$**	**Pre-T_4**	**Assignment**	**$\nu_{\#}$**	**Pre-T_x**	**Assignment**	**$\nu_{\#}$**	**FC-T_4**	**Assignment**	**$\nu_{\#}$**	**FC-T_x**	**Assignment**	**$\nu_{\#}$**	**FCHT-T_4**	**Assignment**	**$\nu_{\#}$**	**FCHT-T_x**	**Assignment**
1190	ν_{50}	1208	ν_{49}	1197	rCH_{I}, rNH_{III}, $sNC_{II,III}$, xCH_{3II}	-	-	-	ν_{51}	1236	rCH_{I}, rNH_{III}, sNC_{III}, sCC_{I}	-	-	-	ν_{50}	1208		-	-	-
1269	ν_{53}	1276	ν_{52}	1260	xCH_{3II}, rCH_{I}, $sNC_{II,III}$, sCC_{I}	-	-	-	ν_{52}	1260	xCH_{3II}, rCH_{I}, $sNC_{II,III}$, sCC_{I}	-	-	-	ν_{53}	1276		-	-	-
1391	ν_{55}	1360	ν_{61}	1413	xCH_{3II}, $sNC_{II,III}$, sCC_{I}	-	-	-	ν_{61}	1413	xCH_{3II}, $sNC_{II,III}$, sCC_{I}	-	-	-	ν_{61}	1413	xCH_{3II}, $sNC_{II,III}$, sCC_{I}	-	-	-
	ν_{58}	1380																		
	ν_{59}	1389																		
	ν_{60}	1405																		
1514	ν_{72}	1545	ν_{72}	1545		-	-	-	ν_{69}	1486	xCH_{3II}, sCC_{I}, sNC_{II}, rCH_{I}	-	-	-	ν_{69}	1486	xCH_{3II}, sCC_{I}, sNC_{II}, rCH_{I}	-	-	-
1626	ν_{75}	1720	ν_{74}	1689	(as) sCO_{III}, rNH_{III}, sNC_{III}	-	-	-	ν_{75}	1720		-	-	-	ν_{74}	1689	(as) sCO_{III}, rNH_{III}, sNC_{III}	-	-	-
**Exp. RR FMN T_1**	**OLYP/cc-pVDZ**																			
	**$\nu_{\#}$**	**T_1offR**	**$\nu_{\#}$**	**Pre-T_6**	**Assignment**	**$\nu_{\#}$**	**Pre-T_7**	**Assignment**	**$\nu_{\#}$**	**FC-T_6**	**Assignment**	**$\nu_{\#}$**	**FC-T_7**	**Assignment**	**$\nu_{\#}$**	**FCHT-T_6**	**Assignment**	**$\nu_{\#}$**	**FCHT-T_7**	**Assignment**
1190	ν_{50}	1202	ν_{49}	1184	rNH_{III}, rCH_{I}, $sNC_{II,III}$, xCH_{3II}	ν_{49}	1184	rNH_{III}, rCH_{I}, $sNC_{II,III}$, xCH_{3II}	ν_{50}	1202		ν_{50}	1202		ν_{50}	1202		ν_{50}	1202	
1269	ν_{53}	1267	ν_{52}	1254	rCH_{I}, xCH_{3II}, sCC_{I}, sNC_{II}	ν_{52}	1254	rCH_{I}, xCH_{3II}, sCC_{I}, sNC_{II}	ν_{53}	1267		ν_{52}	1254	rCH_{I}, xCH_{3II}, sCC_{I}, sNC_{II}	ν_{53}	1267		ν_{52}	1254	rCH_{I}, xCH_{3II}, sCC_{I}, sNC_{II}
1391	ν_{55}	1341	ν_{61}	1396	xCH_{3II}, $sNC_{II,III}$, sCC_{I}, rCH_{I}	ν_{61}	1396	xCH_{3II}, $sNC_{II,III}$, sCC_{I}, rCH_{I}	ν_{64}	1417	$xCH_{3I,II}$, $sNC_{II,III}$	ν_{59}	1378		ν_{61}	1396	xCH_{3II}, $sNC_{II,III}$, sCC_{I}, rCH_{I}	ν_{56}	1349	rNH_{III}, sNC_{III}, $xCH_{3I,II}$, sCC_{I}
	ν_{58}	1362																		
	ν_{59}	1378																		
	ν_{60}	1388																		
1514	ν_{72}	1525	ν_{72}	1525		ν_{70}	1504	sCC_{I}, rCH_{I}, xCH_{3I}, sNC_{II}	ν_{72}	1525		ν_{69}	1479	sCC_{I}, $sNC_{II,III}$, xCH_{3I}, rCH_{I}	ν_{72}	1525		ν_{70}	1504	sCC_{I}, rCH_{I}, xCH_{3I}, sNC_{II}
1626	ν_{75}	1684	ν_{73}	1603	sCC_{I}, rCH_{I}, sNC_{II}, sCO_{III}	ν_{73}	1603	sCC_{I}, rCH_{I}, sNC_{II}, sCO_{III}	ν_{73}	1603	sCC_{I}, rCH_{I}, sNC_{II}, sCO_{III}	ν_{73}	1603	sCC_{I}, rCH_{I}, sNC_{II}, sCO_{III}	ν_{74}	1647	(as) sCO_{III}, rNH_{III}, sNC_{III}	ν_{73}	1603	sCC_{I}, rCH_{I}, sNC_{II}, sCO_{III}
**Exp. RR FMN T_1**	**OPBE/cc-pVDZ**																			
	**$\nu_{\#}$**	**T_1offR**	**$\nu_{\#}$**	**Pre-T_6**	**Assignment**	**$\nu_{\#}$**	**Pre-T_7**	**Assignment**	**$\nu_{\#}$**	**FC-T_6**	**Assignment**	**$\nu_{\#}$**	**FC-T_7**	**Assignment**	**$\nu_{\#}$**	**FCHT-T_6**	**Assignment**	**$\nu_{\#}$**	**FCHT-T_7**	**Assignment**
1190	ν_{50}	1206	ν_{49}	1193	rCH_{I}, rNH_{III}, $sNC_{III,II}$, sCC_{I}	ν_{49}	1193	rCH_{I}, rNH_{III}, $sNC_{III,II}$, sCC_{I}	ν_{50}	1206		ν_{50}	1206		ν_{51}	1243	rCH_{I}, rNH_{III}, $sNC_{III,II}$, sCC_{I}	ν_{50}	1206	
1269	ν_{53}	1283	ν_{52}	1252	rCH_{I}, rNH_{III}, $sNC_{III,II}$, sCC_{I}	ν_{52}	1252	rCH_{I}, rNH_{III}, $sNC_{III,II}$, sCC_{I}	ν_{52}	1252	rCH_{I}, rNH_{III}, $sNC_{III,II}$, sCC_{I}	ν_{52}	1252	rCH_{I}, rNH_{III}, $sNC_{III,II}$, sCC_{I}	ν_{53}	1283		ν_{52}	1252	rCH_{I}, rNH_{III}, $sNC_{III,II}$, sCC_{I}
1391	-	-	ν_{67}	1443	$xCH_{3II,I}$, $sNC_{II,III}$, sCC_{I}	ν_{67}	1443	$sNC_{II,III}$, sCC_{I}	ν_{66}	1425	$xCH_{3II,I}$, $sNC_{II,III}$	ν_{59}	1384	$xCH_{3I,II}$, sCC_{I}, $sNC_{II,III}$, rNH_{III}	ν_{67}	1443	$xCH_{3II,I}$, $sNC_{II,III}$, sCC_{I}	ν_{57}	1358	
	ν_{57}	1358																		
	ν_{64}	1422																		
	-	-																		
1514	ν_{72}	1556	ν_{72}	1556		ν_{70}	1519	sCC_{I}, rCH_{I}, sNC_{II}, xCH_{3I}	ν_{72}	1556		ν_{69}	1495	sCC_{I}, $sNC_{II,III}$, $xCH_{3I,II}$, rCH_{I}	ν_{72}	1556		ν_{70}	1519	sCC_{I}, rCH_{I}, sNC_{II}, xCH_{3I}
1626	ν_{75}	1714	ν_{73}	1621	sCC_{I}, rCH_{I}, $sNC_{II,III}$, sCO_{III}		1556		ν_{73}	1621	sCC_{I}, rCH_{I}, $sNC_{II,III}$, sCO_{III}	ν_{73}	1621	sCC_{I}, rCH_{I}, $sNC_{II,III}$, sCO_{III}	ν_{74}	1676	(as) sCO_{III}, rNH_{III}, sNC_{III}	ν_{73}	1621	sCC_{I}, rCH_{I}, $sNC_{II,III}$, sCO_{III}
**Exp. RR FMN T_1**	**tHCTHhyb/cc-pVDZ**																			
	**$\nu_{\#}$**	**T_1offR**	**$\nu_{\#}$**	**Pre-T_4**	**Assignment**	**$\nu_{\#}$**	**Pre-T_6**	**Assignment**	**$\nu_{\#}$**	**FC-T_4**	**Assignment**	**$\nu_{\#}$**	**FC-T_6**	**Assignment**	**$\nu_{\#}$**	**FCHT-T_4**	**Assignment**	**$\nu_{\#}$**	**FCHT-T_6**	**Assignment**
1190	ν_{50}	1196	ν_{49}	1188	rCH_{I}, rNH_{III}, $sNC_{II,III}$, xCH_{3II}	ν_{49}	1188	rCH_{I}, rNH_{III}, $sNC_{II,III}$, xCH_{3II}	ν_{51}	1228	rCH_{I}, rNH_{III}, sNC_{II}, $xCH_{3I,II}$	-	-	-	ν_{51}	1228	rCH_{I}, rNH_{III}, sNC_{II}, $xCH_{3I,II}$	-	-	-
1269	ν_{53}	1263		1252			1252			1252		-	-	-	ν_{53}	1263		-	-	-
1391	ν_{55}	1353	ν_{60}	1399		ν_{60}	1399		ν_{62}	1410	xCH_{3II}, $sNC_{II,III}$, sCC_{I}	-	-	-	ν_{62}	1410	xCH_{3II}, $sNC_{II,III}$, sCC_{I}	-	-	-
	ν_{57}	1370																		
	ν_{58}	1374																		
	ν_{60}	1399																		
1514	ν_{72}	1531	ν_{72}	1531		ν_{72}	1531		ν_{69}	1477	$xCH_{3II,I}$, rCH_{I}, sNC_{II}, sCC_{I}	-	-	-	ν_{69}	1477	$xCH_{3II,I}$, rCH_{I}, sNC_{II}, sCC_{I}	-	-	-
1626	ν_{75}	1717	ν_{74}	1688	(as) sCO_{III}, rNH_{III}, sNC_{III}	ν_{74}	1688	(as) sCO_{III}, rNH_{III}, sNC_{III}	ν_{75}	1717		-	-	-	ν_{74}	1688	(as) sCO_{III}, rNH_{III}, sNC_{III}	-	-	-

Exp. RR FMN T_1	revTPSS/aug-cc-pVDZ																			
	$\nu_\#$	T_1offR	$\nu_\#$	Pre-T_5	Assignment	$\nu_\#$	Pre-T_6	Assignment	$\nu_\#$	FC-T_5	Assignment	$\nu_\#$	FC-T_6	Assignment	$\nu_\#$	FCHT-T_5	Assignment	$\nu_\#$	FCHT-T_6	Assignment
1190	ν_{50}	1191	ν_{49}	1170	xCH_{3II}, rCH_{I}, rNH_{III}, $sNC_{III,II}$	ν_{49}	1170	xCH_{3II}, rCH_{I}, rNH_{III}, $sNC_{III,II}$	ν_{50}	1191		ν_{50}	1191		ν_{50}	1191		ν_{50}	1191	
1269	ν_{52}	1251	ν_{53}	1259	rCH_{I}, sCC_{I}, $sNC_{II,III}$, xCH_{3I}	ν_{53}	1259	rCH_{I}, sCC_{I}, $sNC_{II,III}$, xCH_{3I}	ν_{54}	1274	sNC_{II}, rCH_{I}, rNH_{III}	ν_{54}	1274	sNC_{II}, rCH_{I}, rNH_{III}	ν_{54}	1274	sNC_{II}, rCH_{I}, rNH_{III}	ν_{52}	1251	
1391	ν_{55}	1332	ν_{59}	1377	$sNC_{II,III}$, xCH_{3I}, rNH_{III}, sCC_{I}	ν_{59}	1377	$sNC_{II,III}$, xCH_{3I}, rNH_{III}, sCC_{I}	ν_{59}	1377	$sNC_{II,III}$, xCH_{3I}, rNH_{III}, sCC_{I}	ν_{59}	1377	$sNC_{II,III}$, xCH_{3I}, rNH_{III}, sCC_{I}	ν_{59}	1377	$sNC_{II,III}$, xCH_{3I}, rNH_{III}, sCC_{I}	ν_{59}	1377	$sNC_{II,III}$, xCH_{3I}, rNH_{III}, sCC_{I}
	ν_{56}	1348																		
	ν_{58}	1376																		
	ν_{60}	1386																		
1514	ν_{70}	1487	ν_{72}	1501	$sCC_{I,II}$, rCH_{I}, $sNC_{II,III}$, xCH_{3I}	ν_{72}	1501	$sCC_{I,II}$, rCH_{I}, $sNC_{II,III}$, xCH_{3I}	ν_{71}	1500	$xCH_{3I,II}$, sCC_{I}, sNC_{II}, rCH_{I}	ν_{71}	1500	$xCH_{3I,II}$, sCC_{I}, sNC_{II}, rCH_{I}	ν_{71}	1500	$xCH_{3I,II}$, sCC_{I}, sNC_{II}, rCH_{I}	ν_{72}	1501	$sCC_{I,II}$, rCH_{I}, $sNC_{II,III}$, xCH_{3I}
1626	ν_{75}	1617	ν_{74}	1595	sCO_{III}, rNH_{III}, sCC_{I}, rCH_{I}	ν_{74}	1595	sCO_{III}, rNH_{III}, sCC_{I}, rCH_{I}	ν_{75}	1617		ν_{74}	1595	sCO_{III}, rNH_{III}, sCC_{I}, rCH_{I}	ν_{74}	1595	sCO_{III}, rNH_{III}, sCC_{I}, rCH_{I}	ν_{74}	1595	sCO_{III}, rNH_{III}, sCC_{I}, rCH_{I}

Exp. RR FMN T_1	revTPSS/aug-cc-pVDZ (continued)											
	$\nu_\#$	T_1offR	$\nu_\#$	Pre-T_7	Assignment	$\nu_\#$	FC-T_7	Assignment	$\nu_\#$	FCHT-T_7	Assignment	
1190	ν_{50}	1191	ν_{49}	1170	xCH_{3II}, rCH_{I}, rNH_{III}, $sNC_{III,II}$	ν_{50}	1191		ν_{50}	1191		
1269	ν_{52}	1251	ν_{53}	1259	rCH_{I}, sCC_{I}, $sNC_{II,III}$, xCH_{3I}	ν_{54}	1274	sNC_{II}, rCH_{I}, rNH_{III}	ν_{54}	1274	sNC_{II}, rCH_{I}, rNH_{III}	
1391	ν_{55}	1332	ν_{59}	1377	$sNC_{II,III}$, xCH_{3I}, rNH_{III}, sCC_{I}	ν_{59}	1377	$sNC_{II,III}$, xCH_{3I}, rNH_{III}, sCC_{I}	ν_{59}	1377	$sNC_{II,III}$, xCH_{3I}, rNH_{III}, sCC_{I}	
	ν_{56}	1348										
	ν_{58}	1376										
	ν_{60}	1386										
1514	ν_{70}	1487	ν_{72}	1501	$sCC_{I,II}$, rCH_{I}, $sNC_{II,III}$, xCH_{3I}	ν_{72}	1501	$sCC_{I,II}$, rCH_{I}, $sNC_{II,III}$, xCH_{3I}	ν_{71}	1500	$xCH_{3I,II}$, sCC_{I}, sNC_{II}, rCH_{I}	
1626	ν_{75}	1617	ν_{74}	1595	sCO_{III}, rNH_{III}, sCC_{I}, rCH_{I}	ν_{73}	1579	sCO_{III}, rNH_{III}, sCC_{I}, rCH_{I}	ν_{75}	1617		

Exp. RR FMN T_1	TPSSh/cc-pVDZ																			
	$\nu_\#$	T_1offR	$\nu_\#$	Pre-T_4	Assignment	$\nu_\#$	Pre-T_6	Assignment	$\nu_\#$	FC-T_4	Assignment	$\nu_\#$	FC-T_6	Assignment	$\nu_\#$	FCHT-T_4	Assignment	$\nu_\#$	FCHT-T_6	Assignment
1190	ν_{50}	1202	ν_{49}	1188	rCH_{I}, rNH_{III}, $sNC_{III,II}$, xCH_{3II}	ν_{50}	1202		ν_{49}	1188	rCH_{I}, rNH_{III}, $sNC_{III,II}$, xCH_{3II}	ν_{51}	1231	rCH_{I}, $sNC_{III,II}$, rNH_{III}, sCC_{I}	ν_{50}	1202		ν_{50}	1202	
1269	ν_{52}	1256	ν_{53}	1270	rCH_{I}, sCC_{I}, $sNC_{III,II}$	ν_{53}	1270	rCH_{I}, sCC_{I}, $sNC_{III,II}$	ν_{52}	1256		ν_{52}	1256		ν_{51}	1231	rCH_{I}, $sNC_{III,II}$, rNH_{III}, sCC_{I}	ν_{53}	1270	rCH_{I}, sCC_{I}, $sNC_{III,II}$
1391	ν_{55}	1354	ν_{61}	1410		ν_{58}	1388	xCH_{3I}, rNH_{III}, sCC_{I}	ν_{61}	1410		ν_{61}	1410		ν_{60}	1401	rNH_{III}, $sNC_{III,II}$, sCC_{I}	ν_{61}	1410	
	ν_{56}	1374																		
	ν_{59}	1397																		
	ν_{61}	1410																		
1514	ν_{72}	1529	ν_{72}	1529		ν_{63}	1450	xCH_{3I}, $sNC_{III,II}$	ν_{71}	1526	sCC_{I}, xCH_{3I}, rCH_{I}, $sNC_{II,III}$	ν_{69}	1486	$xCH_{3II,I}$, sCC_{I}, rCH_{I}, $sNC_{II,III}$	ν_{71}	1526	sCC_{I}, xCH_{3I}, rCH_{I}, $sNC_{II,III}$	ν_{69}	1486	$xCH_{3II,I}$, sCC_{I}, rCH_{I}, $sNC_{II,III}$
1626	ν_{75}	1699	ν_{73}	1667	sCC_{I}, rCH_{I}, $sNC_{II,III}$, sCO_{III}	ν_{74}	1667	(as) sCO_{III}, rNH_{III}, sNC_{III}	ν_{73}	1612	sCC_{I}, rCH_{I}, $sNC_{II,III}$, sCO_{III}	ν_{75}	1699		ν_{73}	1612	sCC_{I}, rCH_{I}, $sNC_{II,III}$, sCO_{III}	ν_{74}	1667	(as) sCO_{III}, rNH_{III}, sNC_{III}

Table S19 Statistical analysis pertaining to the agreement between the experimental fifth EAS assigned to 3FMN* and the lumiflavin calculated spectra of the T_1 state including off-Resonance (offR), pre-Resonance (preR), and Resonance Raman (FC/FCHT) methodologies. The terms **μ_σ(offR)**, **μ_σ(preR), μ_σ(FC)** and **μ_σ(FCHT)** are the average deviations of the off-resonance, pre-resonance and FC- and FCHT-resonance Raman spectra, respectively and **$\Delta\mu_\sigma$** the difference of each of the latter three terms with **μ_σ(offR)**. Equivalent **μ_δ**, **$\Delta\mu_\delta$** terms are provided for their percent errors. All deviation values (σ) are given in cm^{-1} and percent errors (δ) in %. All calculations utilize the cc-pVDZ basis set, with the exception of the functionals revTPSS and BHHLYP which use the aug-cc-pVDZ basis set.

Functional	State	μ_σ(offR)	μ_σ(FC)	$\Delta\mu_\sigma$(FC)	μ_σ(FCHT)	$\Delta\mu_\sigma$(FCHT)	μ_σ(preR)	$\Delta\mu_\sigma$(preR)	μ_δ(offR)	μ_δ(FC)	$\Delta\mu_\delta$(FC)	μ_δ(FCHT)	$\Delta\mu_\delta$(FCHT)	μ_δ(preR)	$\Delta\mu_\delta$(preR)
BHHLYP	T_2	71.2	34.0	37.2	46.2	25.0	25.5	45.7	4.8	2.3	2.5	3.3	1.5	1.8	3.0
	T_3		-	-	-	-	25.5	45.7		-	-	-	-	1.8	3.0
BLYP	T_6	36.1	42.8	-6.7	43.4	-7.3	46.5	-10.5	2.7	3.0	-0.3	3.1	-0.4	3.3	-0.6
	T_7		44.2	-8.1	50.4	-14.3	-	-		3.1	-0.4	3.5	-0.9	-	-
BP86	T_6	28.8	23.4	5.4	19.0	9.8	36.0	-7.2	2.1	1.6	0.5	1.4	0.7	2.6	-0.5
	T_7		28.2	0.6	36.2	-7.4	-	-		2.0	0.1	2.6	-0.5	-	-
BPBE	T_6	24.8	19.2	5.6	14.5	10.3	29.5	-4.7	1.8	1.3	0.5	1.1	0.7	2.1	-0.4
	T_7		26.6	-1.8	30.8	-6.0	36.8	-12.0		1.9	-0.1	2.2	-0.4	2.6	-0.8
HCTH	T_6	27.6	16.1	11.5	19.1	8.5	18.3	9.3	1.8	1.1	0.7	1.3	0.5	1.3	0.6
	T_8		14.0	13.6	19.4	8.2	16.6	11.0		1.0	0.8	1.4	0.4	1.1	0.7
mPWLYP	T_6	37.7	39.8	-2.1	38.8	-1.1	48.4	-10.7	2.8	2.7	0.0	2.8	0.0	3.4	-0.7
	T_7		41.2	-3.5	48.0	-10.3	55.9	-18.2		2.8	0.0	3.3	-0.6	3.9	-1.1
O3LYP	T_4	31.5	39.8	-8.3	27.4	4.1	26.4	5.1	2.1	2.8	-0.7	1.9	0.9	1.8	0.3
OLYP	T_6	21.5	14.8	6.7	10.2	11.3	12.0	9.5	1.4	1.0	0.4	0.7	0.7	0.8	0.6
	T_7		19.6	1.9	20.4	1.1	11.8	9.8		1.4	0.1	1.5	0.0	0.8	0.6
OPBE	T_6	32.2	22.8	9.4	42.2	-10.0	23.5	8.7	2.1	1.6	0.5	3.0	-0.9	1.7	0.5
	T_7		12.8	19.4	15.2	17.0	29.2	3.0		0.9	1.2	1.1	1.0	2.0	0.2
revTPSS	T_5	17.1	8.6	8.5	13.0	4.1	17.4	-0.3	1.2	0.6	0.6	0.9	0.3	1.2	0.0
	T_6		13.0	4.1	15.4	1.7	17.4	-0.3		0.9	0.3	1.1	0.2	1.2	0.0
	T_7		16.0	1.1	16.2	0.9	17.4	-0.3		1.0	0.2	1.1	0.1	1.2	0.0
tHCTHhyb	T_4	27.5	40.4	-12.9	32.4	-4.9	21.6	6.0	1.8	2.8	-1.0	2.3	-1.0	1.4	0.4
	T_6		-	-	-	-	21.6	6.0		-	-	-	-	1.4	0.4
TPSSh	T_4	23.9	12.0	11.9	17.2	6.7	15.5	8.5	1.6	0.8	0.8	1.3	0.3	1.0	0.6
	T_6		34.8	-10.9	20.2	3.7	24.3	-0.4		2.4	-0.8	1.4	0.2	1.6	0.0

Table S20 Comparison of the experimental and computed Singlet-Triplet peak shifts. The experimental shifts are based on the 3rd and 5th EAS assigned to 1FMN* and 3FMN*, respectively. The computed shifts of the twelve selected DFT functionals, are based on comparison of the excited singlet resonance Raman spectrum $S_1 \leftrightarrow r_7$ ($S_1 \leftrightarrow r_5$ for BHHLYP) of each corresponding functional, with the computed triplet spectra including off-Resonance, pre-Resonance and resonance FC and FCHT. Shifts predicted with the same sign as the experimental spectra are shaded grey, and the total number of correctly predicted shifts is given under **#Shift**. The mean absolute deviation of the shifts is also provided in cm^{-1} (**μ_σ**).

Exp. Shifts		BHHLYP/aug-cc-pVDZ						
1FMN*→3FMN* S_1→T_1		offR T_1	Pre T_2	PreT_3	FC T_2	FC T_3	FCHT T_2	FCHT T_3
1200→1190	-10	27	27	27	27	-	27	-
1250→1269	19	-11	42	42	42	-	-3	-
1381→1391	10	48	129	129	53	-	37	-
1498→1514	16	-36	21	21	8	-	8	-
1626→1626	0	6	-100	-100	-154	-	-154	-
#Shift		1	3	3	3	-	2	-
μ_σ		32.4	56.9	56.9	53.1	-	49.6	-

Exp. Shifts		BLYP/cc-pVDZ						
1FMN*→3FMN* S_1→T_1		offR T_1	Pre T_6	PreT_7	FC T_6	FC T_7	FCHT T_6	FCHT T_7
1200→1190	-10	-29	6	-	26	26	26	26
1250→1269	19	-19	-1	-	63	63	-18	-18
1381→1391	10	-2	25	-	24	17	17	17
1498→1514	16	-3	-3	-	-2	-2	-16	-16
1626→1626	0	-15	-87	-	-87	-87	-52	-87
#Shift		1	1	-	2	2	1	1
μ_σ		20.5	31.2	-	39.9	38.5	33.0	40.0

Exp. Shifts		BP86/cc-pVDZ						
1FMN*→3FMN* S_1→T_1		offR T_1	Pre T_6	PreT_7	FC T_6	FC T_7	FCHT T_6	FCHT T_7
1200→1190	-10	23	10	-	30	30	50	30
1250→1269	19	-5	-16	-	29	76	-5	-16
1381→1391	10	0	22	-	22	45	22	1
1498→1514	16	6	6	-	6	6	6	6
1626→1626	0	-13	-19	-	-19	-19	25	-19
#Shift		1	2	-	3	3	2	2
μ_σ		18.2	19.2	-	18.4	32.4	26.4	22.8

Exp. Shifts		BPBE/cc-pVDZ						
1FMN*→3FMN* S_1→T_1		offR T_1	Pre T_6	PreT_7	FC T_6	FC T_7	FCHT T_6	FCHT T_7
1200→1190	-10	22	10	29	29	29	29	29
1250→1269	19	-5	-18	-6	28	75	-6	75
1381→1391	10	-3	21	-13	21	44	21	44
1498→1514	16	7	6	-71	6	6	6	-15
1626→1626	0	-13	-19	25	-19	-19	25	-19
#Shift		1	2	0	3	3	2	2
μ_σ		18.2	19.2	39.8	17.5	31.6	21.8	35.8

Exp. Shifts		HCTH/cc-pVDZ						
1FMN*→3FMN* S_1→T_1		offR T_1	Pre T_6	PreT_8	FC T_6	FC T_8	FCHT T_6	FCHT T_8
1200→1190	-10	21	10	10	25	25	25	25
1250→1269	19	-5	-21	-5	-21	-22	-21	-22
1381→1391	10	-8	52	-15	28	11	28	-15
1498→1514	16	15	15	-16	15	2	15	-15
1626→1626	0	-13	-7	49	-7	-8	49	-8
#Shift		1	2	0	2	2	2	0
μ_σ		17.4	22.1	30.0	20.4	19.9	28.8	28.0

Exp. Shifts		mPWLYP/cc-pVDZ						
1FMN*→3FMN* S_1→T_1		offR T_1	Pre T_6	PreT_7	FC T_6	FC T_7	FCHT T_6	FCHT T_7
1200→1190	-10	32	6	6	26	47	81	26
1250→1269	19	-65	-2	-2	21	62	62	62
1381→1391	10	-47	25	38	25	16	25	16
1498→1514	16	-2	-3	-54	-3	-3	-16	-16
1626→1626	0	-14	-90	-90	-90	-90	-52	-90
#Shift		0	1	1	2	2	2	2
μ_σ		43.0	32.2	45.0	32.4	43.0	46.6	41.4

Exp. Shifts		O3LYP/cc-pVDZ						
1FMN*→3FMN* S_1→T_1		offR T_1	Pre T_4	-	FC T_4	-	FCHT T_4	-
1200→1190	-10	26	5	-	44	-	16	-
1250→1269	19	48	-8	-	-8	-	8	-
1381→1391	10	-1	17	-	17	-	17	-
1498→1514	16	19	19	-	-40	-	-40	-
1626→1626	0	-5	61	-	92	-	60	-
#Shift		2	2	-	1	-	2	-
μ_σ		16.6	22.7	-	47.2	-	32.0	-

Exp. Shifts		OLYP/cc-pVDZ						
1FMN*→3FMN* S_1→T_1		offR T_1	Pre T_6	PreT_7	FC T_6	FC T_7	FCHT T_6	FCHT T_7
1200→1190	-10	42	10	10	27	27	27	0
1250→1269	19	53	-17	-17	-3	-16	-3	-13
1381→1391	10	-20	21	21	42	3	21	-68
1498→1514	16	7	7	-15	6	-40	6	-21
1626→1626	0	-13	-21	-21	-21	-21	23	0
#Shift		2	2	1	2	1	2	1
μ_σ		27.7	19.4	23.7	24.5	31.2	20.8	31.4

Exp. Shifts		OPBE/cc-pVDZ						
1FMN*→3FMN* S_1→T_1		offR T_1	Pre T_6	PreT_7	FC T_6	FC T_7	FCHT T_6	FCHT T_7
1200→1190	-10	39	26	26	39	39	76	39
1250→1269	19	46	2	2	1	1	32	1
1381→1391	10	3	55	55	37	-4	55	-30
1498→1514	16	21	21	-16	21	-40	21	-16
1626→1626	0	-11	-5	70	-5	-5	50	-5
#Shift		3	3	2	3	1	3	1
μ_σ		19.8	21.4	40.0	20.7	28.5	39.7	28.9

Exp. Shifts		TPSSh/cc-pVDZ						
1FMN*→3FMN* S_1→T_1		offR T_1	Pre T_4	Pre T_6	FC T_4	FC T_6	FCHT T_4	FCHT T_6
1200→1190	-10	24	4	18	4	47	18	18
1250→1269	19	36	6	6	-8	-8	-33	6
1381→1391	10	-5	27	5	27	27	18	27
1498→1514	16	2	3	-76	0	-40	0	-40
1626→1626	0	-7	46	46	-9	78	-9	46
#Shift		2	3	2	1	1	1	2
μ_σ		17.2	20.8	36.8	16.6	47.0	22.6	32.0

Exp. Shifts		revTPSS/aug-cc-pVDZ										
1FMN*→3FMN* S_1→T_1		offR T_1	offR T_1*	Pre T_5	Pre T_6	Pre T_7	FC T_5	FC T_6	FC T_7	FCHT T_5	FCHT T_6	FCHT T_7
1200→1190	-10	-163	-21	-23	-23	-23	-2	-2	-2	-2	-2	-2
1250→1269	19	-132	14	-37	-37	-37	-22	-22	-22	-22	-45	-22
1381→1391	10	-161	-1	-5	-5	-5	-5	-5	-5	-5	-5	-5
1498→1514	16	-166	8	-38	-38	-38	-40	-40	-39	-40	-39	-40
1626→1626	0	-200	-9	-202	-202	-202	-180	-202	-218	-202	-202	-218
#Shift		1	3	1	1	1	1	1	1	1	1	1
μ_σ		171.4	8.7	67.9	67.9	67.9	60.0	64.4	67.4	64.4	68.8	67.6

Exp. Shifts		tHCTHhyb/cc-pVDZ						
1FMN*→3FMN* S_1→T_1		offR T_1	Pre T_4	PreT_6	FC T_4	FC T_6	FCHT T_4	FCHT T_6
1200→1190	-10	14	1	1	41	-	41	-
1250→1269	19	42	-5	-5	-5	-	6	-
1381→1391	10	-3	9	9	20	-	20	-
1498→1514	16	16	16	16	-38	-	-38	-
1626→1626	0	-4	1	1	30	-	1	-
#Shift		2	3	3	1	-	3	-
μ_σ		13.1	7.5	7.5	33.8	-	25.8	-

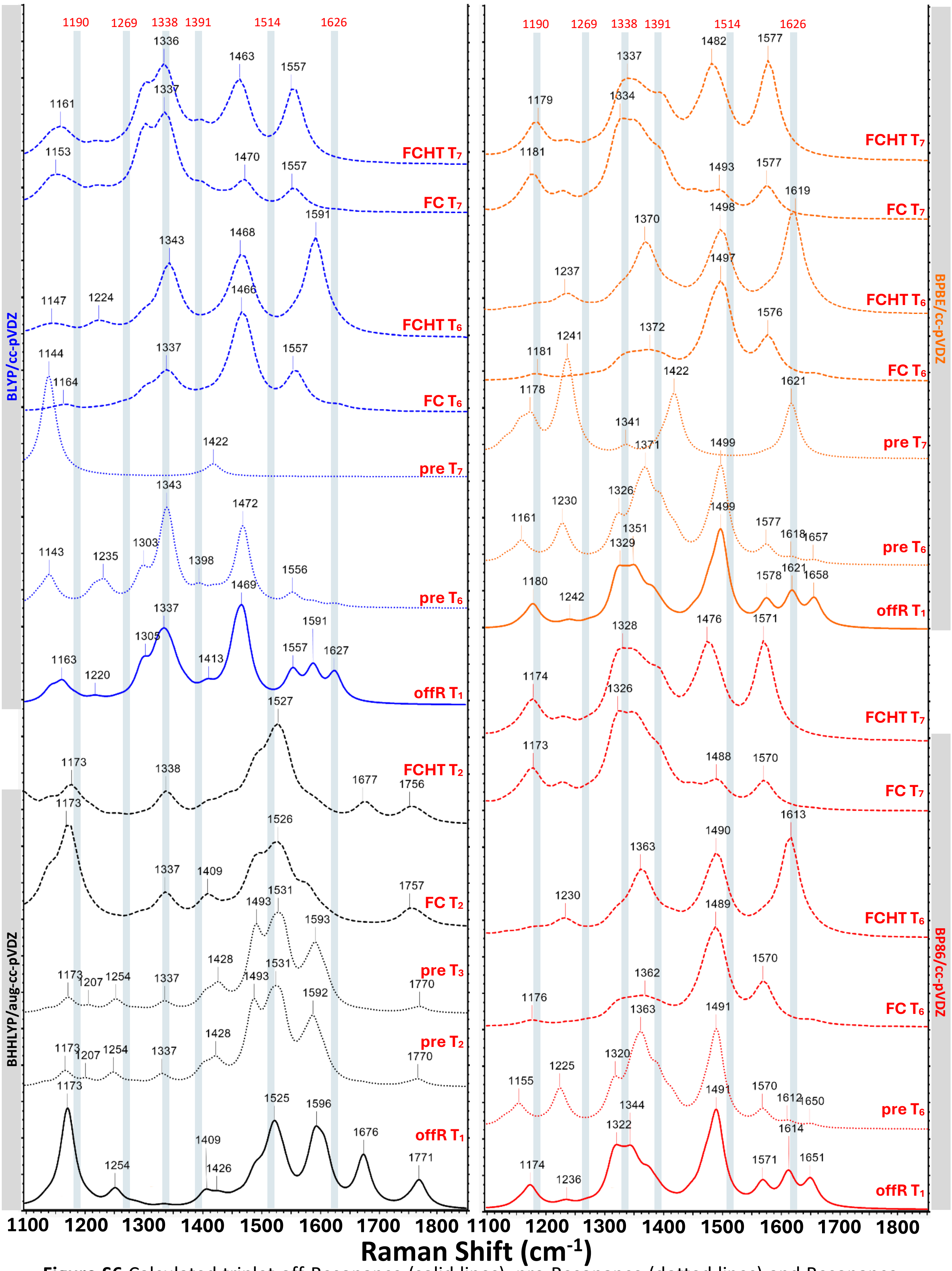

Figure S6 Calculated triplet off-Resonance (solid lines), pre-Resonance (dotted lines) and Resonance spectra (dashed lines) of the various DFT functionals. Experimental peaks of the 3FMN* EAS have been marked and shown as light blue bars. Peak wavenumbers are approximate according to the Spectragryph program that produced the plots and will differ slightly from the precise values included in **Tables S17-S18**. The intensities of all spectra have been normalized.

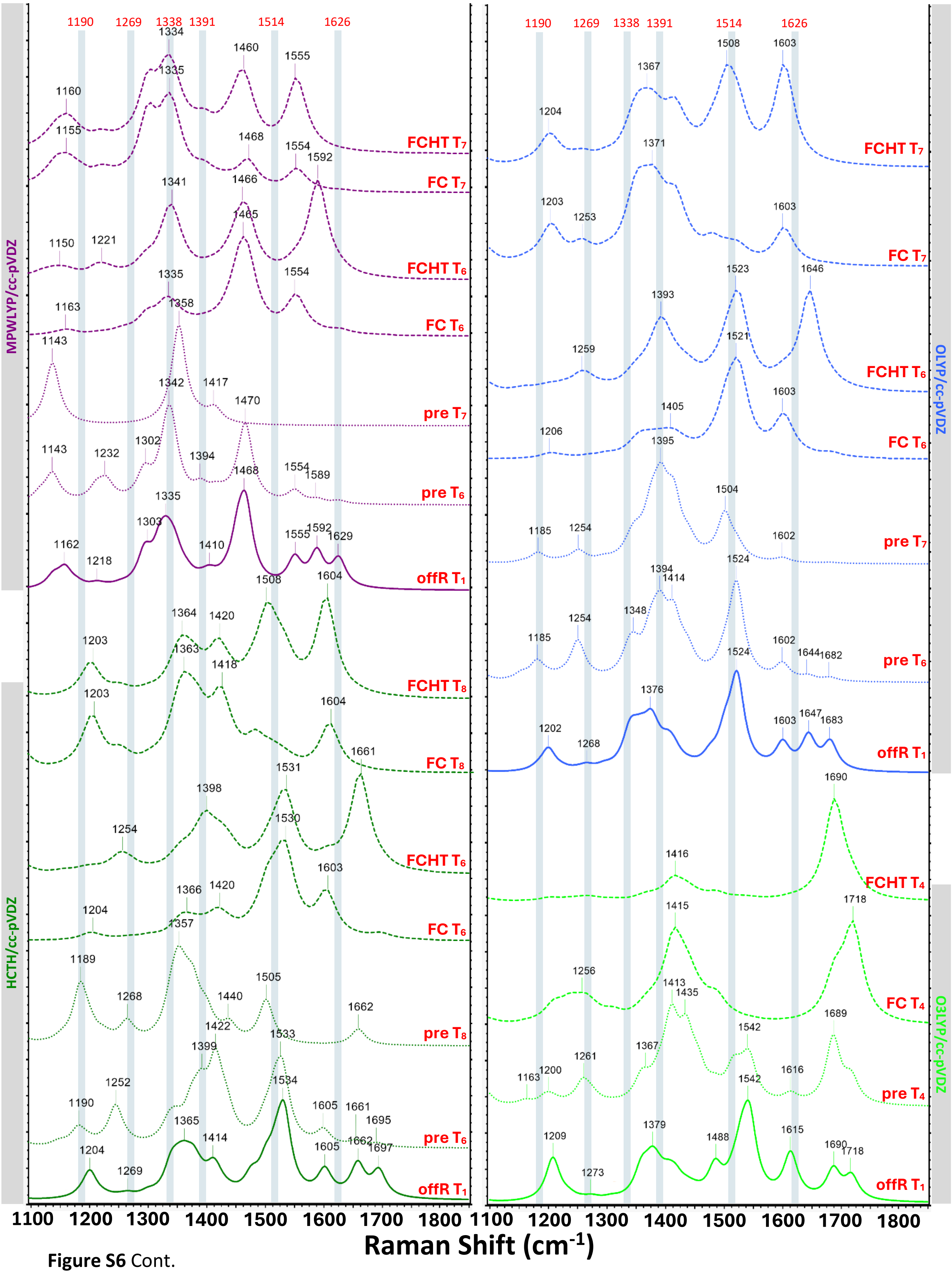

Figure S6 Cont.

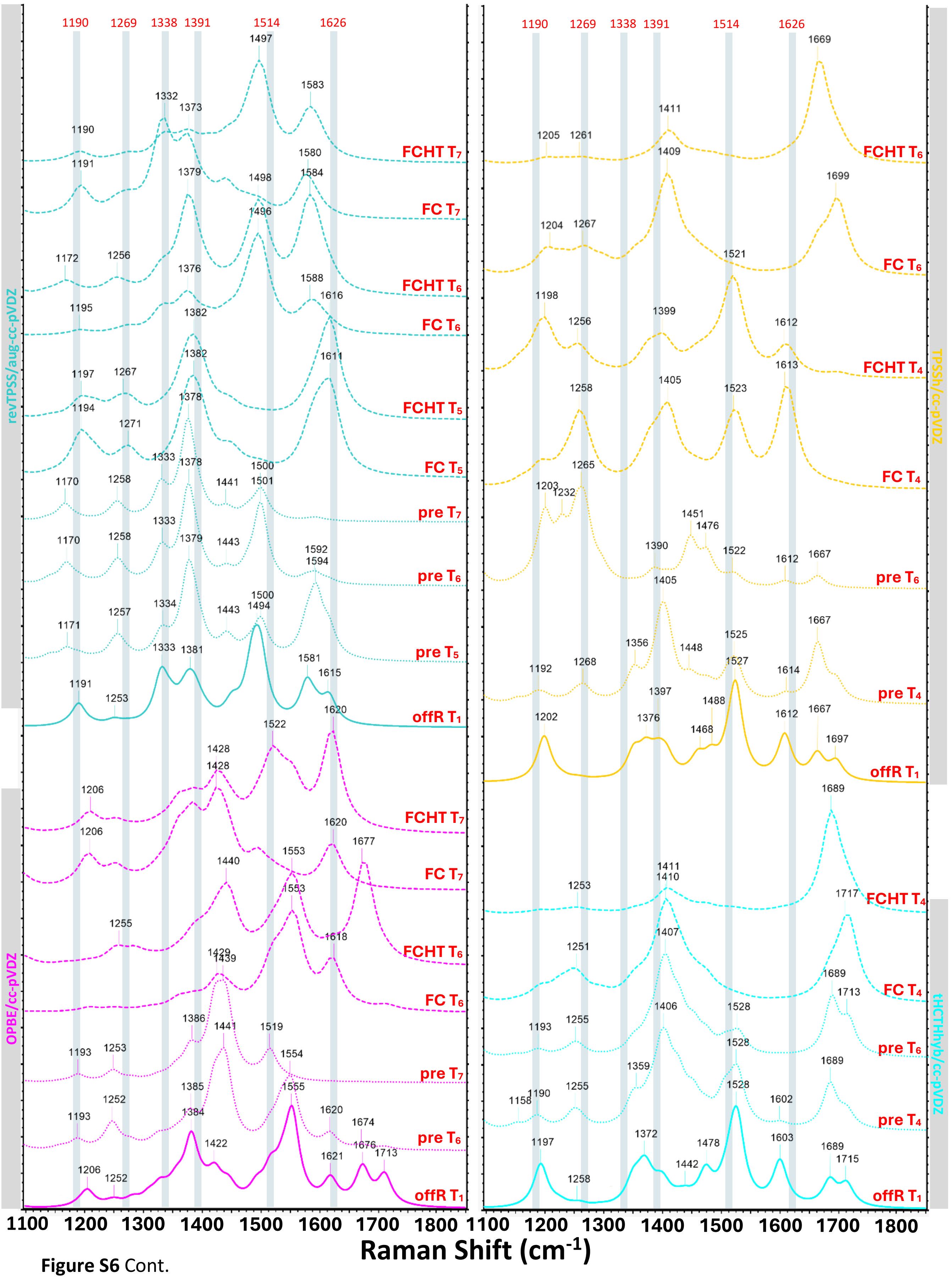

Figure S6 Cont.

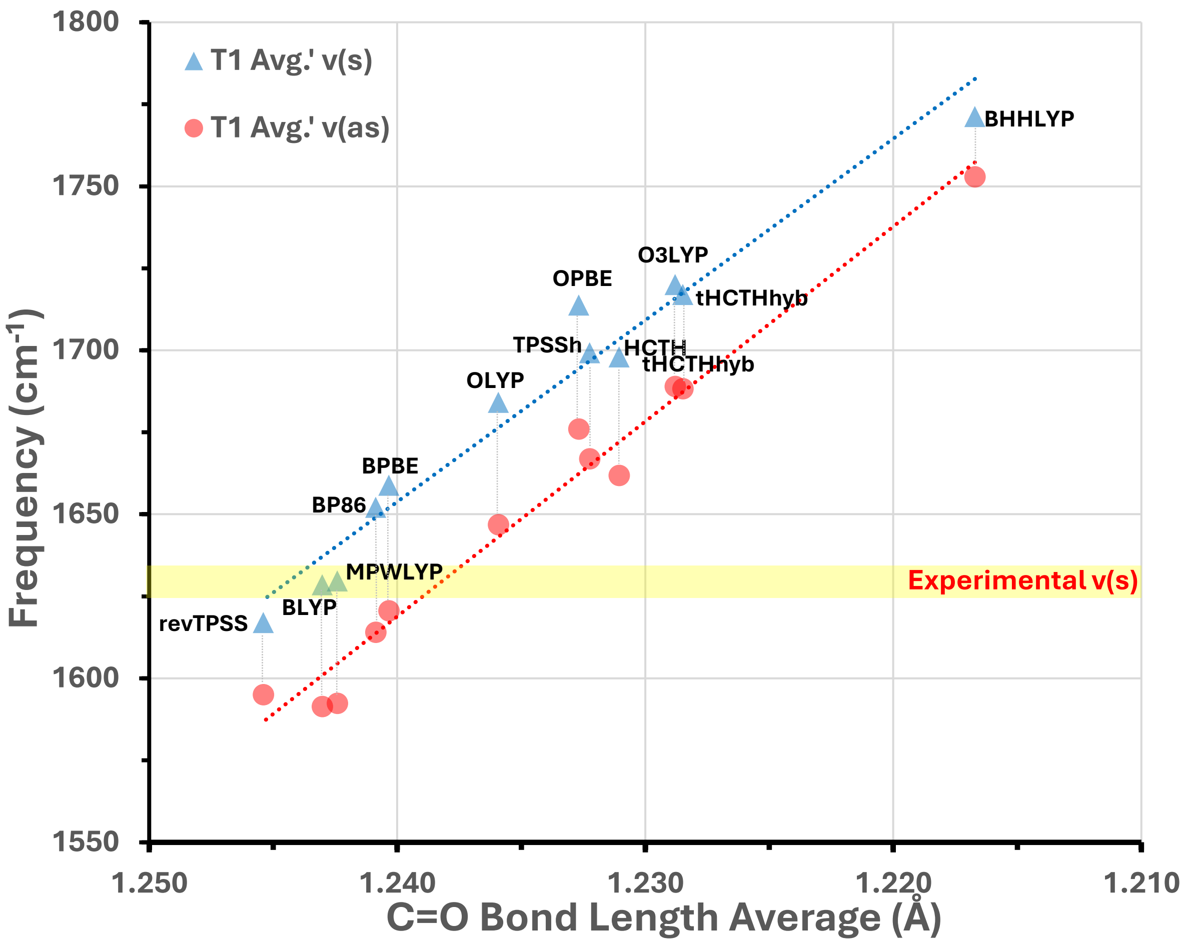

Figure S7 Relationship between the length of the $C_2{=}O_{2'}$ and $C_4{=}O_{4'}$ bonds (x-axis, averaged) calculated for the T_1 state with the selected twelve DFT functionals, and their corresponding symmetric v(s) and asymmetric v(as) stretching frequencies (y-axis). The vs/C=O points are shown with blue triangles and the v(as)/C=O points in red circles. Trendlines with corresponding color have been drawn for each of the two sets. The experimental $C_2{=}O_{2'}/C_4{=}O_{4'}$ symmetric stretching v(s) frequency (1626 cm^{-1}) is highlighted in yellow.

4.1 Evaluation of the DFT Functionals on the Triplet Spectra

The criteria for the evaluation of the computed triplet pre-Resonance and Resonance spectra, similarly to the excited singlet variants, are as follows:

a) For this criterion, a full mark is given if the mean percent error, μ_δ of the offR, preR or rR (FC or FCHT) computed peaks associated with the five most prominent experimental FSRS peaks of the 5th EAS, is equal or lower than 1.2%. A half-mark is given for DFT functionals with an error between 1.4-1.2%.

b) The difference between the off-Resonance and (pre-)resonance Raman mean percent errors ($\Delta\mu_\delta$) is positive. If $\Delta\mu_\delta$ is negative, no mark is awarded, if it is higher than 0.5% the full mark is awarded and a half mark is given for functionals with positive $\Delta\mu_\delta$.

c) The normalized value of the vibration assigned to 1514 cm^{-1} (in the range v_{67}-v_{72}, mostly v_{72}). For values between 0.8-1.0 a full mark is given, signifying that the DFT functional correctly (or almost correctly) predicts the strongest peak in the spectrum. A half mark is given for normalized values between 0.5-0.8, and below 0.5, no mark is awarded.

d) The fourth criterion involves the singlet-triplet peak shifts, as documented in **Table S20**. It combines the correct prediction of the shift sign, i.e. blueshift or redshift,

combined with the value of the mean absolute deviation (μ_σ) of the shift between calculation and experiment. A half mark is added if the functional/method correctly predicts the sign of three out of the five experimental shifts. Another half mark is awarded for μ_σ values below 25 cm^{-1}.

e) The final fifth criterion, entails visual inspection of the spectra and comparison with he experimental line shape. The DFT functional is evaluated positively when the correlation of the theoretical-experimental spectra is facile: (i) with a full mark for very similar line shapes to the experimental curve or (ii) half mark for less similar but still providing for a facile correlation. A negative evaluation with no mark is given for computed spectra that combine one or more of the following attributes: (iii) exhibit more (or less) than 4-5 strong peaks in the vicinity of the experimental peaks at ~1190 (m), 1270 (w), 1390 (vs), 1514 (vs) and 1626 cm^{-1} (vw), (iv) doublet peaks at 1190-1269 cm^{-1} and the single peak at 1391 cm^{-1} cannot be clearly assigned or the predicted peaks are of strong intensity, and (v) the C=O symmetric or asymmetric stretch (v_{75} and v_{74} respectively) is predicted as the strongest peak in the spectrum.

Table S21 Evaluation of DFT Functionals on the prediction of the triplet experimental spectra of FMN, according to the five criteria described above: **a)** the mean percent error of their correlation, **b)** the difference in the mean percent errors of the resonance (FC, FCHT) or pre-Resonance and the off-Resonance Raman spectra (T_1), **c)** the intensity of the normalized theoretical peak assigned to the most prominent experimental peak at 1514 cm^{-1}, **d)** the of the Singlet-Triplet shifts by combining the shift sign and the absolute deviation, and **e)** visual evaluation, where the computed resonance Raman spectra are classified after visual inspection according to the categories (i)-(v) described above. After the triplet score, in the last column the combined score for the DFT functional is given, using the highest possible evaluation from each **Table 3** in the main text and **S19** for a total of 10 points (i.e. for BP86, the T_6 FC scoring is used).

LOT	a) μ_δ (%)	b) rR-offR $\Delta\mu_\delta$ (%)	c) 1514 cm^{-1} peak	d) S-T Shifts	e) Visual Evaluation	Triplet Score	Combined Score
BHHLYP T_2 pre	□ (1.8)	■ (3.0)	■ (0.9)	◧ (3/56.9)	□ (iii)	■■◧□□	
BHHLYP T_2 FC	□ (2.3)	■ (2.5)	■ (1.0)	◧ (3/53.1)	□ (iv)	■■◧□□	4/10
BHHLYP T_2 FCHT	□ (3.3)	■ (1.5)	■ (0.8)	□ (2/49.6)	◧ (ii)	■■◧□□	
BLYP T_6 pre	□ (3.3)	□ (-0.6)	■ (0.8)	□ (1/31.2)	□ (ii)	■□□□□	
BLYP T_6 FC	□ (3.0)	□ (-0.3)	■ (0.8)	□ (2/39.9)	□ (iv)	■□□□□	
BLYP T_6 FCHT	□ (3.1)	□ (-0.4)	□ (0.3)	□ (1/33.0)	□ (v)	□□□□□	2.5/10
BLYP T_7 FC	□ (3.1)	□ (-0.4)	□ (0.3)	□ (2/38.5)	□ (iv)	□□□□□	
BLYP T_7 FCHT	□ (3.5)	□ (-0.9)	◧ (0.7)	□ (1/40.0)	□ (v)	◧□□□□	
BP86 T_6 pre	□ (2.6)	□ (-0.5)	■ (1.0)	◧ (2/19.2)	◧ (ii)	■■□□□	
BP86 T_6 FC	□ (1.6)	◧ (0.5)	■ (1.0)	■ (3/18.4)	□ (iv)	■■◧□□	
BP86 T_6 FCHT	◧ (1.4)	■ (0.7)	□ (0.3)	□ (2/26.4)	□ (v)	■◧□□□	5.5/10
BP86 T_7 FC	□ (2.0)	◧ (0.1)	□ (0.3)	◧ (3/32.4)	□ (iv)	■□□□□	
BP86 T_7 FCHT	□ (2.6)	□ (-0.5)	□ (0.2)	◧ (2/22.8)	□ (v)	◧□□□□	
BPBE T_6 pre	□ (2.1)	□ (-0.4)	■ (1.0)	◧ (2/19.2)	◧ (ii)	■■□□□	
BPBE T_6 FC	◧ (1.3)	◧ (0.5)	■ (1.0)	■ (3/17.5)	□ (iv)	■■■□□	
BPBE T_6 FCHT	■ (1.1)	■ (0.7)	□ (0.3)	◧ (2/21.8)	□ (v)	■■◧□□	6/10
BPBE T_7 pre	□ (2.6)	□ (-0.8)	◧ (0.7)	□ (0/39.8)	□ (iii)	◧□□□□	
BPBE T_7 FC	□ (1.9)	□ (-0.1)	□ (0.3)	◧ (3/31.6)	□ (iv)	◧□□□□	
BPBE T_7 FCHT	□ (2.2)	□ (-0.4)	■ (0.8)	□ (2/35.8)	□ (v)	■□□□□	
HCTH T_6 pre	◧ (1.3)	■ (0.6)	■ (0.9)	◧ (2/22.1)	■ (i)	■■■■□	
HCTH T_6 FC	■ (1.1)	■ (0.7)	■ (1.0)	◧ (2/20.4)	□ (iii)	■■■◧□	
HCTH T_6 FCHT	◧ (1.3)	◧ (0.5)	□ (0.2)	□ (2/28.8)	□ (v)	■□□□□	8/10
HCTH T_8 pre	■ (1.1)	■ (0.7)	◧ (0.5)	□ (0/30.0)	◧ (ii)	■■■□□	
HCTH T_8 FC	■ (1.0)	■ (0.8)	□ (0.3)	◧ (2/19.9)	□ (iii)	■■◧□□	
HCTH T_8 FCHT	◧ (1.4)	◧ (0.4)	■ (0.8)	□ (0/28.0)	□ (v)	■■□□□	

mPWLYP T_6 pre	□ (3.4)	□ (-0.7)	■ (0.8)	□ (1/32.2)	◧ (ii)	■◧□□□	2.5/10
mPWLYP T_6 FC	□ (2.7)	□ (0.0)	■ (0.9)	□ (2/23.4)	□ (iii)	■□□□□	
mPWLYP T_6 FCHT	□ (2.8)	□ (0.0)	□ (0.3)	□ (2/46.6)	□ (v)	□□□□□	
mPWLYP T_7 pre	□ (3.9)	□ (-1.1)	□ (0.2)	□ (1/45.0)	□ (iii)	□□□□□	
mPWLYP T_7 FC	□ (2.8)	□ (0.0)	□ (0.4)	□ (2/43.0)	□ (iii)	□□□□□	
mPWLYP T_7 FCHT	□ (3.3)	□ (-0.6)	◧ (0.7)	□ (2/41.4)	◧ (ii)	■□□□□	
O3LYP T_4 pre	□ (1.8)	◧ (0.3)	◧ (0.6)	◧ (2/22.7)	□ (v)	■◧□□□	5.5/10
O3LYP T_4 FC	□ (2.8)	□ (-0.7)	□ (0.3)	□ (1/47.2)	□ (v,iii)	□□□□□	
O3LYP T_4 FCHT	□ (1.9)	■ (0.9)	□ (0.3)	□ (2/32.0)	□ (v,iii)	■□□□□	
OLYP T_6 pre	■ (0.8)	■ (0.6)	■ (1.0)	◧ (2/19.4)	■ (i)	■■■■◧	9/10
OLYP T_6 FC	■ (1.0)	◧ (0.4)	■ (1.0)	◧ (2/24.5)	□ (iv)	■■■□□	
OLYP T_6 FCHT	■ (0.7)	■ (0.7)	□ (0.3)	◧ (2/20.8)	□ (v)	■■◧□□	
OLYP T_7 pre	■ (0.8)	■ (0.6)	◧ (0.5)	◧ (1/23.7)	□ (iii)	■■■□□	
OLYP T_7 FC	◧ (1.4)	◧ (0.1)	□ (0.4)	□ (1/31.2)	□ (iii)	■□□□□	
OLYP T_7 FCHT	□ (1.5)	□ (0.0)	■ (0.8)	□ (1/31.4)	□ (v)	■□□□□	
OPBE T_6 pre	□ (1.7)	◧ (0.5)	◧ (0.7)	■ (3/21.4)	◧ (ii)	■■◧□□	6.5/10
OPBE T_6 FC	□ (1.6)	◧ (0.5)	■ (1.0)	■ (3/20.7)	□ (iv)	■■◧□□	
OPBE T_6 FCHT	□ (3.0)	□ (-0.9)	□ (0.3)	◧ (3/39.7)	□ (v)	◧□□□□	
OPBE T_7 pre	□ (2.0)	◧ (0.2)	□ (0.3)	□ (2/40.0)	□ (iii)	◧□□□□	
OPBE T_7 FC	■ (0.9)	■ (1.2)	◧ (0.5)	□ (1/28.5)	□ (iii)	■■◧□□	
OPBE T_7 FCHT	■ (1.1)	■ (1.0)	◧ (0.7)	□ (1/28.9)	□ (v)	■■◧□□	
revTPSS T_5 pre	■ (1.2)	□ (0.0)	□ (0.4)	□ (1/67.9)	□ (v)	■□□□□	5/10
revTPSS T_5 FC	■ (0.6)	■ (0.6)	□ (0.0)	□ (1/60.0)	□ (v)	■■□□□	
revTPSS T_5 FCHT	■ (0.9)	◧ (0.3)	□ (0.4)	□ (1/64.4)	□ (v)	■◧□□□	
revTPSS T_6 pre	■ (1.2)	□ (0.0)	■ (0.8)	□ (1/67.9)	■ (i)	■■■□□	
revTPSS T_6 FC	■ (0.9)	◧ (0.3)	■ (1.0)	□ (1/64.4)	□ (iii)	■■◧□□	
revTPSS T_6 FCHT	■ (1.1)	◧ (0.2)	□ (0.1)	□ (1/68.8)	◧ (ii)	■■□□□	
revTPSS T_7 pre	■ (1.2)	□ (0.0)	□ (0.3)	□ (1/67.9)	◧ (ii)	■◧□□□	
revTPSS T_7 FC	■ (1.0)	◧ (0.2)	□ (0.1)	□ (1/67.4)	□ (iii)	■◧□□□	
revTPSS T_7 FCHT	■ (1.1)	◧ (0.1)	■ (1.0)	□ (1/67.6)	□ (iii)	■■◧□□	
tHCTHhyb T_4 pre	◧ (1.4)	◧ (0.4)	◧ (0.6)	■ (3/7.5)	◧ (ii)	■■■□□	6.5/10
tHCTHhyb T_4 FC	□ (2.8)	□ (-1.0)	□ (0.2)	□ (1/33.8)	□ (v)	□□□□□	
tHCTHhyb T_4 FCHT	□ (2.3)	□ (-1.0)	□ (0.3)	◧ (3/25.8)	□ (v)	◧□□□□	
tHCTHhyb T_6 pre	◧ (1.4)	◧ (0.4)	□ (0.3)	■ (3/7.5)	□ (iii)	■■□□□	
TPSSh T_4 pre	■ (1.0)	■ (0.6)	◧ (0.5)	■ (3/20.8)	◧ (ii)	■■■■□	8/10
TPSSh T_4 FC	■ (0.8)	■ (0.8)	◧ (0.5)	◧ (1/16.6)	□ (v)	■■■□□	
TPSSh T_4 FCHT	◧ (1.3)	◧ (0.3)	■ (1.0)	◧ (1/22.6)	◧ (ii)	■■■□□	
TPSSh T_6 pre	□ (1.6)	□ (0.0)	◧ (0.5)	□ (2/36.8)	□ (iii)	◧□□□□	
TPSSh T_6 FC	□ (2.4)	□ (-0.8)	□ (0.1)	□ (1/47.0)	□ (v)	□□□□□	
TPSSh T_6 FCHT	◧ (1.4)	◧ (0.2)	□ (0.2)	□ (2/32.0)	□ (v)	■□□□□	

Table S22 Prediction of singlet-triplet peak shifts according to assignments made utilizing either the displacement or potential energy distribution analysis (PED), for the best three performing DFT functionals. Red and blue experimental/calculated shifts are coloured accordingly. In the PED S_1 assignment column, normal modes are colour coded based on the singlet-triplet bond adjustments shown in **Figure 8** of the main text. In both S_1 and T_1 PED assignment columns, normal modes common with the displacement assignment (**Tables S12-S13** and **S17-S18**, respectively) are underlined. PED assignments were computed with the Veda program.[80] Prediction of the peak shifts is based on the number of normal modes contributing to blue or red shifts and their relative displacements. For PED, the magnitude of the coefficient of each normal mode is also factored in the prediction.

Experimental Shifts		Calculated Shifts	PED Assignment (S_1)	Displacement Prediction	PED Prediction	PED Assignment (T_1)
1200→1190 -10	HCTH	1179→1189 10	(27%) C_4-N_3, C_{5a}-N_5, C_2-N_3<br>(11%) $C_{6,9}$-H	Blue	Blue	(24%) C_{4a}-C_{10a}, N_5-C_{4a}, C_4-N_3<br>(11%) C_9-H
1250→1269 19	HCTH	1274→1252 -21	(28%) C_6-H	Blue	-	(21%) N_{10}-C_{10a}<br>(12%) C_9-H<br>(10%) $C_{7a,11a}$-H_3
1381→1391 10	HCTH	1370→1422 52	(33%) C_2-N_3, C_4-N_3, C_{9a}-N_{10}, N_5-C_{4a}<br>(12%) N_3-H	Blue	Blue	(30%) $C_{7a,8a}$-H_3<br>(12%) C_{10a}-N_1, C_{5a}-N_5, N_5-C_{4a}
1498→1514 16	HCTH	1520→1536 15	(55%) C_8-C_7, C_9-C_{9a}, C_6-C_{5a}	Blue	-	(64%) C_{10a}-N_1, C_{4a}-N_5, C_{4a}-C_{10a}<br>(11%) C_2-N_1-C_{10a}, C_{4a}-C_{10a}-N_{10}
1626→1626 0	HCTH	1613→1605 -7	(53%) C_8-C_9, C_6-C_{5a}, C_7-C_6	Blue	Blue	(46%) C_8-C_9, C_6-C_{5a}
1200→1190 -10	OLYP	1175→1184 10	(21%) N_5-C_{5a}, C_4-N_3<br>(11%) $C_{6,9}$-H	Blue	Blue	(13%) N_5-C_{4a}, N_5-C_{5a}<br>(11%) C_8-C_{8a}, N_3-C_2<br>(12%) C_9-H
1250→1269 19	OLYP	1270→1254 -17	(30%) C_6-H	Blue	-	(27%) C_9-H<br>(10%) C_{11}-H_3
1381→1391 10	OLYP	1375→1396 21	(18%) N_3-H<br>(15%) C_{11}-H_3	Blue	-	(20%) C_{11}-H_3<br>(12%) C_4-N_3, C_{9a}-N_{10}, N_{10}-C_{10a}, N_3-C_2<br>(11%) C_{4a}-N_5, C_{10a}-N_1<br>(10%) N_1-C_2, C_{10a}-N_{10}
1498→1514 16	OLYP	1519→1526 7	(63%) C_8-C_7, C_9-C_{9a}, C_6-C_{5a}<br>(13%) $C_{6,9}$-H	-	Red	(52%) N_5-C_{4a}, C_{10a}-C_{4a}, N_1-C_{10a}<br>(10%) C_2-N_1-C_{10a}, C_{4a}-C_{10a}-N_{10}
1626→1626 0	OLYP	1624→1603 -21	(56%) C_2=$O_{2'}$	Blue	Blue	(52%) C_8-C_9, C_6-C_{5a}, C_7-C_6
1200→1190 -10	TPSSh	1184→1188 4	(18%) C_2-N_3, C_4-N_3<br>(11%) C_6-H<br>(10%) N_5-C_{5a}, N_5-C_{4a}	Red	Blue	(12%) $C_{6,9}$-H<br>(11%) N_{10}-C_{10a}, C_4-N_3, C_{9a}-N_{10}, C_2-N_3<br>(10%) C_{10a}-C_{4a}, N_5-C_{4a}, N_5-C_{5a}
1250→1269 19	TPSSh	1264→1270 6	(42%) C_9-H<br>(22%) C_{5a}-C_6, C_8-C_{8a}, C_7-C_{7a}	Red	Red	(50%) $C_{6,9}$-H
1381→1391 10	TPSSh	1383→1410 27	(26%) N_5-C_{4a}, N_1-C_{10a}<br>(13%) $C_{7a,8a}$-H_3	Blue	Blue	(10%) N_1-C_2, N_{10}-C_{10a}, N_{10}-C_{9a}
1498→1514 16	TPSSh	1526→1529 3	(55%) C_8-C_7, C_9-C_{9a}, C_6-C_{5a}	~Blue	Red	(51%) C_{10a}-C_{4a}, N_5-C_{4a}
1626→1626 0	TPSSh	1621→1667 46	(53%) C_8-C_9, C_6-C_{5a}	Blue	-	(70%) C_2-$O_{2'}$

5. Additional References

1. Slater, J. C.; Phillips, J. C. Physics Today 1974, 27(12), 49-50.
2. Vosko, S. H.; Wilk, L.; Nusair, M. Canadian Journal of Physics 1980, 58(8), 1200-1211.
3. Becke, A. D. Phys Rev A Gen Phys 1988, 38(6), 3098-3100.
4. Lee, C.; Yang, W.; Parr, R. G. Phys Rev B Condens Matter 1988, 37(2), 785-789.
5. Miehlich, B.; Savin, A.; Stoll, H.; Preuss, H. Chem Phys Lett 1989, 157(3), 200-206.
6. Perdew, J. P. Phys Rev B Condens Matter 1986, 33(12), 8822-8824.
7. Perdew, J. P.; Burke, K.; Ernzerhof, M. Phys Rev Lett 1996, 77(18), 3865-3868.
8. Perdew, J. P.; Burke, K.; Ernzerhof, M. Physical Review Letters 1997, 78(7), 1396-1396.
9. Hamprecht, F. A.; Cohen, A. J.; Tozer, D. J.; Handy, N. C. J Chem Phys 1998, 109(15), 6264-6271.
10. Boese, A. D.; Doltsinis, N. L.; Handy, N. C.; Sprik, M. J Chem Phys 2000, 112(4), 1670-1678.
11. Boese, A. D.; Handy, N. C. J Chem Phys 2001, 114(13), 5497-5503.
12. Adamo, C.; Barone, V. J Chem Phys 1998, 108(2), 664-675.
13. Handy, N. C.; Cohen, A. J. Molecular Physics 2009, 99(5), 403-412.
14. Hoe, W.-M.; Cohen, A. J.; Handy, N. C. Chem Phys Lett 2001, 341(3-4), 319-328.
15. Peverati, R.; Zhao, Y.; Truhlar, D. G. The Journal of Physical Chemistry Letters 2011, 2(16), 1991-1997.
16. Zhao, Y.; Truhlar, D. G. J Chem Phys 2006, 125(19), 194101.
17. Peverati, R.; Truhlar, D. G. The Journal of Physical Chemistry Letters 2011, 3(1), 117-124.
18. Perdew, J. P.; Ruzsinszky, A.; Csonka, G. I.; Constantin, L. A.; Sun, J. Phys Rev Lett 2009, 103(2), 026403.
19. Perdew, J. P.; Ruzsinszky, A.; Csonka, G. I.; Constantin, L. A.; Sun, J. Physical Review Letters 2011, 106(17).
20. Tao, J.; Perdew, J. P.; Staroverov, V. N.; Scuseria, G. E. Phys Rev Lett 2003, 91(14), 146401.
21. Van Voorhis, T.; Scuseria, G. E. J Chem Phys 1998, 109(2), 400-410.
22. Austin, A.; Petersson, G. A.; Frisch, M. J.; Dobek, F. J.; Scalmani, G.; Throssell, K. J Chem Theory Comput 2012, 8(12), 4989-5007.
23. Becke, A. D. J Chem Phys 1996, 104(3), 1040-1046.
24. Becke, A. D. J Chem Phys 1993, 98(7), 5648-5652.
25. Perdew, J. P.; Chevary, J. A.; Vosko, S. H.; Jackson, K. A.; Pederson, M. R.; Singh, D. J.; Fiolhais, C. Phys Rev B Condens Matter 1992, 46(11), 6671-6687.
26. Perdew, J. P.; Chevary, J. A.; Vosko, S. H.; Jackson, K. A.; Pederson, M. R.; Singh, D. J.; Fiolhais, C. Phys Rev B Condens Matter 1993, 48(7), 4978.
27. Perdew, J. P.; Burke, K.; Wang, Y. Phys Rev B Condens Matter 1996, 54(23), 16533-16539.
28. Becke, A. D. J Chem Phys 1997, 107(20), 8554-8560.
29. Schmider, H. L.; Becke, A. D. J Chem Phys 1998, 108(23), 9624-9631.
30. Becke, A. D. J Chem Phys 1993, 98(2), 1372-1377.
31. Cohen, A. J.; Handy, N. C. Molecular Physics 2001, 99(7), 607-615.
32. Adamo, C.; Barone, V. J Chem Phys 1999, 110(13), 6158-6170.
33. Peverati, R.; Truhlar, D. G. J Chem Phys 2011, 135(19), 191102.
34. Xu, X.; Goddard, W. A., 3rd. Proc Natl Acad Sci U S A 2004, 101(9), 2673-2677.
35. Boese, A. D.; Martin, J. M. J Chem Phys 2004, 121(8), 3405-3416.
36. Zhao, Y.; Schultz, N. E.; Truhlar, D. G. J Chem Theory Comput 2006, 2(2), 364-382.
37. Zhao, Y.; Truhlar, D. G. Theoretical Chemistry Accounts 2007, 120(1-3), 215-241.
38. Zhao, Y.; Truhlar, D. G. J Phys Chem A 2006, 110(15), 5121-5129.
39. Zhao, Y.; Truhlar, D. G. J Phys Chem A 2006, 110(49), 13126-13130.
40. Yu, H. S.; He, X.; Li, S. L.; Truhlar, D. G. Chem Sci 2016, 7(8), 5032-5051.
41. Zhao, Y.; Truhlar, D. G. J Phys Chem A 2005, 109(25), 5656-5667.
42. Boese, A. D.; Handy, N. C. J Chem Phys 2002, 116(22), 9559-9569.

43. Staroverov, V. N.; Scuseria, G. E.; Tao, J.; Perdew, J. P. J Chem Phys 2003, 119(23), 12129-12137.
44. Iikura, H.; Tsuneda, T.; Yanai, T.; Hirao, K. J Chem Phys 2001, 115(8), 3540-3544.
45. Yanai, T.; Tew, D. P.; Handy, N. C. Chem Phys Lett 2004, 393(1-3), 51-57.
46. Henderson, T. M.; Izmaylov, A. F.; Scuseria, G. E.; Savin, A. J Chem Theory Comput 2008, 4(8), 1254-1262.
47. Heyd, J.; Scuseria, G. E. J Chem Phys 2004, 121(3), 1187-1192.
48. Heyd, J.; Scuseria, G. E. J Chem Phys 2004, 120(16), 7274-7280.
49. Heyd, J.; Peralta, J. E.; Scuseria, G. E.; Martin, R. L. J Chem Phys 2005, 123(17), 174101.
50. Henderson, T. M.; Izmaylov, A. F.; Scalmani, G.; Scuseria, G. E. J Chem Phys 2009, 131(4), 044108.
51. Izmaylov, A. F.; Scuseria, G. E.; Frisch, M. J. J Chem Phys 2006, 125(10), 104103.
52. Krukau, A. V.; Vydrov, O. A.; Izmaylov, A. F.; Scuseria, G. E. J Chem Phys 2006, 125(22), 224106.
53. Vydrov, O. A.; Scuseria, G. E. J Chem Phys 2006, 125(23), 234109.
54. Vreven, T.; Frisch, M. J.; Kudin, K. N.; Schlegel, H. B.; Morokuma, K. Molecular Physics 2007, 104(5-7), 701-714.
55. Vydrov, O. A.; Scuseria, G. E.; Perdew, J. P. J Chem Phys 2007, 126(15), 154109.
56. Chai, J. D.; Head-Gordon, M. Phys Chem Chem Phys 2008, 10(44), 6615-6620.
57. M. J. Frisch; G. W. Trucks; H. B. Schlegel; G. E. Scuseria; M. A. Robb; J. R. Cheeseman; G. Scalmani; V. Barone; G. A. Petersson; H. Nakatsuji; X. Li; M. Caricato; A. V. Marenich; J. Bloino; B. G. Janesko; R. Gomperts; B. Mennucci; H. P. Hratchian; J. V. Ortiz; A. F. Izmaylov; J. L. Sonnenberg; D. Williams-Young; F. Ding; F. Lipparini; F. Egidi; J. Goings; B. Peng; A. Petrone; T. Henderson; D. Ranasinghe; V. G. Zakrzewski; J. Gao; N. Rega; G. Zheng; W. Liang; M. Hada; M. Ehara; K. Toyota; R. Fukuda; J. Hasegawa; M. Ishida; T. Nakajima; Y. Honda; O. Kitao; H. Nakai; T. Vreven; K. Throssell; J. A. Montgomery, J.; J. E. Peralta; F. Ogliaro; M. J. Bearpark; J. J. Heyd; E. N. Brothers; K. N. Kudin; V. N. Staroverov; T. A. Keith; R. Kobayashi; J. Normand; K. Raghavachari; A. P. Rendell; J. C. Burant; S. S. Iyengar; J. Tomasi; M. Cossi; J. M. Millam; M. Klene; C. Adamo; R. Cammi; J. W. Ochterski; R. L. Martin; K. Morokuma; O. Farkas; J. B. Foresman; Fox, D. J. Gaussian 16, Revision C.01, C.01; Gaussian, Inc., Wallingford CT: 2016.
58. Grimme, S. List of functionals and coefficients for BJ-damping: https://www.chemie.uni-bonn.de/grimme/de/software/dft-d3/bj_damping.
59. MolSSI Empirical dispersion resources, and references therein. : https://github.com/MolSSI/QCEngine/blob/master/qcengine/programs/empirical_dispersion_resources.py.
60. Goerigk, L. J Phys Chem Lett 2015, 6(19), 3891-3896.
61. Liu, Y.; Chaudhari, A. S.; Chatterjee, A.; Andrikopoulos, P. C.; Picchiotti, A.; Rebarz, M.; Kloz, M.; Lorenz-Fonfria, V. A.; Schneider, B.; Fuertes, G. Biomolecules 2023, 13(1), 161.
62. Yu, H. S.; Fiedler, L. J.; Alecu, I. M.; Kanchanakungwankul, S.; Truhlar, D. G. FREQ v.2; 2021.
63. Alecu, I. M.; Zheng, J.; Zhao, Y.; Truhlar, D. G. J Chem Theory Comput 2010, 6(9), 2872-2887.
64. Yu, H. S.; Fiedler, L. J.; Alecu, I. M.; Truhlar, D. G. Comput Phys Commun 2017, 210, 132-138.
65. Computational Chemistry Comparison and Benchmark DataBase: https://cccbdb.nist.gov/vibscalejustx.asp.
66. Laury, M. L.; Boesch, S. E.; Haken, I.; Sinha, P.; Wheeler, R. A.; Wilson, A. K. J Comput Chem 2011, 32(11), 2339-2347.
67. Tantirungrotechai, Y.; Phanasant, K.; Roddecha, S.; Surawatanawong, P.; Sutthikhum, V.; Limtrakul, J. Journal of Molecular Structure: THEOCHEM 2006, 760(1-3), 189-192.
68. Merrick, J. P.; Moran, D.; Radom, L. J Phys Chem A 2007, 111(45), 11683-11700.
69. Kesharwani, M. K.; Brauer, B.; Martin, J. M. J Phys Chem A 2015, 119(9), 1701-1714.
70. Palafox, M. A. Physical Sciences Reviews 2018, 3(6).

71. Barone, V.; Bloino, J.; Biczysko, M.; Santoro, F. J Chem Theory Comput 2009, 5(3), 540-554.
72. Bloino, J.; Biczysko, M.; Santoro, F.; Barone, V. J Chem Theory Comput 2010, 6(4), 1256-1274.
73. Baiardi, A.; Bloino, J.; Barone, V. J Chem Phys 2014, 141(11), 114108.
74. Duschinsky, F. Acta Physicochim URSS 1937, 7, 551.
75. Baiardi, A.; Bloino, J.; Barone, V. J Chem Theory Comput 2013, 9(9), 4097-4115.
76. Andrikopoulos, P. C.; Liu, Y.; Picchiotti, A.; Lenngren, N.; Kloz, M.; Chaudhari, A. S.; Precek, M.; Rebarz, M.; Andreasson, J.; Hajdu, J.; Schneider, B.; Fuertes, G. Physical Chemistry Chemical Physics 2020, 22(12), 6538-6552.
77. Lu, T.; Chen, F. J Comput Chem 2012, 33(5), 580-592.
78. Liu, Z.; Lu, T.; Chen, Q. Carbon 2020, 165, 461-467.
79. Menges, F. Spectragryph - optical spectroscopy software - https://www.effemm2.de/spectragryph/, 1.2.11.
80. Jamroz, M. H. Spectrochim Acta A Mol Biomol Spectrosc 2013, 114, 220-230.

6. Cartesian Coordinates of all optimized species

APFD/cc-pVDZ_S0

N	0.10908169	0.71006368	-0.48337163
C	-0.23464048	2.03784139	-0.50530242
O	-1.39072912	2.42553452	-0.60266522
N	0.78857088	3.00137502	-0.41059636
C	2.13711455	2.77137642	-0.30013137
O	2.96270255	3.66580493	-0.22392600
C	2.47620153	1.32177930	-0.28396735
N	3.73127941	0.99244870	-0.18496436
C	4.04097879	-0.33069547	-0.16900334
C	5.39550039	-0.71061668	-0.06481249
C	5.78058087	-2.03621954	-0.04313285
C	7.22668984	-2.41743596	0.06912646
C	4.76917589	-3.03877099	-0.12938704
C	5.14992862	-4.48719545	-0.10979308
C	3.42956295	-2.68210928	-0.23213257
C	3.04048532	-1.33356977	-0.25409332
N	1.72097417	-0.94531219	-0.35601451
C	1.37596558	0.38123830	-0.37958017
C	0.67327819	-1.95743545	-0.44012778
H	0.48491644	3.97211247	-0.42922373
H	6.13212237	0.09318259	-0.00181855
H	7.54987696	-3.02389976	-0.79220444
H	7.86913743	-1.52860842	0.12041734
H	7.41201867	-3.02578996	0.96908743
H	5.69599453	-4.73843807	0.81383247
H	4.27082548	-5.14049134	-0.17927049
H	5.82674735	-4.72759177	-0.94567436
H	2.68403827	-3.47335389	-0.29639463
H	0.83065757	-2.58012670	-1.33054362
H	-0.28355945	-1.43593216	-0.51192094
H	0.69351940	-2.58611080	0.45977471

APFD/cc-pVDZ_S1(r1)

N	0.14045909	0.70320723	-0.47490823
C	-0.24695781	2.03886510	-0.50275229
O	-1.43068895	2.34502419	-0.59713445
N	0.74189704	2.99734406	-0.42039111
C	2.11535257	2.77066505	-0.31038304
O	2.90311004	3.71030457	-0.24442869
C	2.48363280	1.36421683	-0.28379997
N	3.79074569	1.04163682	-0.17829059
C	4.06699266	-0.28500374	-0.16471246
C	5.39845912	-0.70264165	-0.05962937
C	5.77448782	-2.05341506	-0.04142210
C	7.21351946	-2.42579488	0.07409460
C	4.77188250	-3.06272099	-0.13331905
C	5.14228972	-4.51321767	-0.11895496
C	3.44133716	-2.67439732	-0.23631880
C	3.06170587	-1.31621171	-0.25324290
N	1.74515749	-0.93620459	-0.35221707
C	1.42815143	0.41216410	-0.37239478
C	0.69455306	-1.94164327	-0.43253863
H	0.44120032	3.96822504	-0.44270672
H	6.15881020	0.07793954	0.00913171
H	7.53154959	-3.04184172	-0.78406140
H	7.86186283	-1.54257049	0.12907289
H	7.38853134	-3.04553104	0.97008842
H	5.68099014	-4.78142855	0.80492968
H	4.25425435	-5.15452156	-0.19253335
H	5.81449087	-4.76462168	-0.95611662
H	2.67930966	-3.44998100	-0.30571648
H	0.84914233	-2.56867965	-1.32171763
H	-0.26366929	-1.42402754	-0.50292317
H	0.71644499	-2.57208694	0.46748186

APFD/cc-pVDZ_r5

N	0.11005136	0.71906593	-0.59548684
C	-0.21945724	2.00800246	-0.60774462
O	-1.33034486	2.46649842	-0.97000663
N	0.72332829	2.96569721	-0.21432585
C	2.07932363	2.72011304	-0.23750608
O	2.86861717	3.68724026	-0.20801851
C	2.46535201	1.33164838	-0.26632147
N	3.77965831	1.02285887	-0.17489121
C	4.06390942	-0.29850764	-0.16815809
C	5.41339907	-0.71284949	-0.08268284
C	5.78788881	-2.04872093	-0.05900857
C	7.23548466	-2.43337110	0.03517198
C	4.77988704	-3.04310055	-0.12500319
C	5.14030020	-4.49867807	-0.10330399
C	3.43888773	-2.65795446	-0.21271184
C	3.06119706	-1.31168318	-0.23483125
N	1.72244826	-0.92444950	-0.32370385
C	1.40179938	0.40741773	-0.38459579
C	0.68401105	-1.93830056	-0.39650358
H	0.43007399	3.94276999	-0.24778459
H	6.16650841	0.07719053	-0.03413457
H	7.54642080	-3.04471024	-0.82785022
H	7.88357370	-1.54764273	0.07582901
H	7.43169178	-3.04132626	0.93370411
H	5.68960541	-4.76353259	0.81547299
H	4.24841557	-5.13705779	-0.16052463
H	5.80149935	-4.76208905	-0.94562455
H	2.68166692	-3.44016088	-0.26102806
H	0.83277974	-2.57084315	-1.28371048
H	-0.28113094	-1.43237783	-0.46666876
H	0.71215790	-2.56809535	0.50413724

B1B95/cc-pVDZ_S0

N	0.11686034	0.70743263	-0.48366319
C	-0.22533707	2.03002715	-0.50491253
O	-1.37768311	2.41594683	-0.60246930
N	0.79244729	2.98947062	-0.40939933
C	2.13582344	2.76346551	-0.29806384
O	2.95478194	3.65850475	-0.22063295
C	2.47799072	1.31992118	-0.28258774
N	3.73045139	0.99239214	-0.18388703
C	4.04114806	-0.32947712	-0.16868725
C	5.39128104	-0.71057130	-0.06538458
C	5.77199317	-2.03305688	-0.04416163
C	7.21055919	-2.42466227	0.06707032
C	4.76206823	-3.03089971	-0.12964281
C	5.14729501	-4.47329290	-0.10887014
C	3.42749335	-2.67355109	-0.23224291
C	3.04428759	-1.32774315	-0.25382236
N	1.72735998	-0.94140093	-0.35599737
C	1.38102552	0.38054969	-0.37918548
C	0.68780237	-1.95721631	-0.44104109
H	0.48761632	3.95693828	-0.42734060
H	6.12744756	0.08927761	-0.00268087
H	7.52146855	-3.04127987	-0.78762649
H	7.85755209	-1.54257799	0.10835126
H	7.38950789	-3.02532139	0.96983648
H	5.69914631	-4.71610028	0.80993409
H	4.27122830	-5.12601421	-0.17180484
H	5.81900152	-4.70921163	-0.94619636
H	2.68068785	-3.45935274	-0.29700387
H	0.84961534	-2.57463175	-1.33113864
H	-0.27091542	-1.44542799	-0.50761502
H	0.71899187	-2.58908326	0.45305188

B1B95/cc-pVDZ_S1(r1)

N	0.14864752	0.69982909	-0.47478317
C	-0.23610748	2.03124671	-0.50183026
O	-1.41471358	2.33770049	-0.59723260
N	0.74821028	2.98599896	-0.41760879
C	2.11516452	2.76228573	-0.30707121
O	2.89643940	3.70233770	-0.24004943
C	2.48512958	1.35973069	-0.28183155
N	3.79004089	1.04035577	-0.17664320
C	4.06670477	-0.28311848	-0.16401878
C	5.39412410	-0.70237345	-0.06059747
C	5.76530305	-2.04858664	-0.04289849
C	7.19734474	-2.43106036	0.07065627
C	4.76370703	-3.05449955	-0.13292016
C	5.13912345	-4.49865617	-0.11668156
C	3.43826998	-2.66608359	-0.23535492
C	3.06347309	-1.31121372	-0.25260556
N	1.75079458	-0.93482925	-0.35246364
C	1.43274764	0.41122032	-0.37182472
C	0.70832384	-1.94337418	-0.43568107
H	0.44757498	3.95405946	-0.43933161
H	6.15367814	0.07451279	0.00716939
H	7.50285887	-3.05703785	-0.78081699
H	7.84948484	-1.55399435	0.11521182
H	7.36645338	-3.04221556	0.96998073
H	5.68695873	-4.75727297	0.80091799
H	4.25409034	-5.13981489	-0.17965725
H	5.80323929	-4.74709182	-0.95727120
H	2.67546364	-3.43658535	-0.30418677
H	0.86976833	-2.56615677	-1.32352211
H	-0.25170211	-1.43580319	-0.50393174
H	0.73840080	-2.57645591	0.45906385

B1B95/cc-pVDZ_r5/r6

N	0.11653801	0.71634520	-0.59905753
C	-0.20841061	1.99920621	-0.61006537
O	-1.30667067	2.46536816	-0.98901900
N	0.72785644	2.95377243	-0.19956477
C	2.08474223	2.71241133	-0.23497814
O	2.85803373	3.68405730	-0.21261639
C	2.46696888	1.32776117	-0.26888931
N	3.77871846	1.02396839	-0.18088947
C	4.06235469	-0.29824480	-0.17251068
C	5.40641070	-0.71289437	-0.08835183
C	5.77822397	-2.04566984	-0.06150047
C	7.21887130	-2.43828170	0.03135992
C	4.77284953	-3.03606337	-0.12232642
C	5.13836497	-4.48551101	-0.09610159
C	3.43679071	-2.65075124	-0.20938352
C	3.06429973	-1.30765908	-0.23441228
N	1.72725947	-0.92320202	-0.32447435
C	1.40708796	0.40447309	-0.38564541
C	0.69728280	-1.94008849	-0.40047881
H	0.43146432	3.92837274	-0.21426115
H	6.15879311	0.07361022	-0.04265315
H	7.51718386	-3.06461482	-0.82139126
H	7.87017789	-1.55825326	0.05551061
H	7.41128914	-3.03266239	0.93620678
H	5.70148729	-4.73820541	0.81394291
H	4.24922457	-5.12362479	-0.13628506
H	5.78684352	-4.74846840	-0.94456802
H	2.67855581	-3.42787960	-0.25544129
H	0.85524744	-2.57074507	-1.28428314
H	-0.26893048	-1.44335280	-0.47235890
H	0.73008785	-2.57012010	0.49667290

B1B95/cc-pVDZ_r7

N	0.10016601	0.71178391	-0.47807592
C	-0.24179781	2.02180936	-0.50347573
O	-1.42371594	2.37986308	-0.59997467
N	0.75248018	2.97752344	-0.41882105
C	2.11519030	2.75256437	-0.30756002
O	2.90102770	3.69099445	-0.23902360
C	2.48209507	1.34798758	-0.28213817
N	3.78321022	1.03204175	-0.17632757
C	4.07255550	-0.29340740	-0.16221927
C	5.40655315	-0.69828532	-0.05995226
C	5.78716733	-2.04973455	-0.04174703
C	7.21176551	-2.42819157	0.07389524
C	4.77064242	-3.05860471	-0.13386050
C	5.12855991	-4.49647823	-0.12090820
C	3.43305107	-2.65604641	-0.23629278
C	3.08619480	-1.31662011	-0.24952153
N	1.71765712	-0.91392260	-0.35384064
C	1.41519070	0.40080404	-0.37344287
C	0.70283926	-1.93580566	-0.43356518
H	0.45276181	3.94532255	-0.44055245
H	6.16227145	0.08206503	0.00638706
H	7.52164715	-3.06799615	-0.76874683
H	7.86525686	-1.55261434	0.11391635
H	7.38500401	-3.04117736	0.97441244
H	5.68630625	-4.76195405	0.79175565
H	4.24174938	-5.13381910	-0.18409639
H	5.79923135	-4.74886193	-0.95855835
H	2.67196157	-3.42735490	-0.30599057
H	0.88050762	-2.56514172	-1.31680342
H	-0.26886260	-1.45045518	-0.50308696
H	0.75032926	-2.57323463	0.46040101

B3LYP/cc-pVDZ_S0

N	-2.56188465	-1.30864440	-0.00007841
C	-3.75704896	-0.63129437	0.00000149
O	-4.84436568	-1.19636861	0.00027637
N	-3.73292603	0.77968058	0.00000397
C	-2.62333410	1.59311068	-0.00002096
O	-2.69589766	2.81303293	0.00012604
C	-1.34266462	0.82803769	-0.00012888
N	-0.22354472	1.49992803	-0.00010890
C	0.94644127	0.79818578	-0.00006598
C	2.16960076	1.50648403	-0.00001683
C	3.38988703	0.85605877	0.00001867
C	4.68057237	1.62974507	-0.00000085
C	3.40077219	-0.57313945	0.00004712
C	4.70992839	-1.31104014	0.00019776
C	2.20567300	-1.28761431	0.00000921
C	0.96604093	-0.62429606	-0.00007253
N	-0.24008570	-1.30460517	-0.00012663
C	-1.43700517	-0.62444031	-0.00017787
C	-0.24365520	-2.77228048	-0.00009369
H	-4.64155744	1.23793586	0.00022087
H	2.11077759	2.59602098	-0.00000196
H	5.29244851	1.38765757	-0.88449367
H	4.49211763	2.71176150	0.00082342
H	5.29330308	1.38641502	0.88353874
H	5.31247904	-1.04321344	0.88375466
H	4.55993119	-2.39861362	-0.00067873
H	5.31355718	-1.04187441	-0.88218969
H	2.24850057	-2.37441162	0.00002464
H	0.27141426	-3.14168601	-0.89725274
H	-1.28439594	-3.10126707	-0.00008413
H	0.27137139	-3.14165404	0.89710914

B3LYP/cc-pVDZ_S1(r1)

N	-2.53499488	-1.29407141	-0.00007135
C	-3.76560135	-0.63966573	-0.00003673
O	-4.81396133	-1.28031025	-0.00001488
N	-3.76335305	0.74140018	0.00003377
C	-2.63753737	1.57545335	0.00004592
O	-2.77260990	2.79885991	0.00018595
C	-1.36792613	0.86092201	0.00001571
N	-0.21595100	1.57805635	0.00003583
C	0.93298105	0.84967153	0.00002797
C	2.16725262	1.51518140	0.00003945
C	3.39911643	0.84014631	0.00005318
C	4.67944398	1.61454146	0.00000156
C	3.41843949	-0.58704259	0.00006810
C	4.72060338	-1.33628552	0.00016052
C	2.20652009	-1.27252886	0.00003308
C	0.96746570	-0.59473972	0.00000404
N	-0.23051138	-1.27934224	-0.00005395
C	-1.42163348	-0.56614869	-0.00003876
C	-0.23918907	-2.74461721	-0.00012959
H	-4.66986260	1.20228371	0.00010340
H	2.14229010	2.60579283	0.00004436
H	5.29320757	1.36005246	-0.88132196
H	4.49893560	2.69699814	0.00043615
H	5.29375860	1.35938511	0.88073173
H	5.32962941	-1.08277477	0.88426099
H	4.55454187	-2.42196689	-0.00027944
H	5.33017515	-1.08211544	-0.88336188
H	2.22604999	-2.36036581	0.00002787
H	0.27834359	-3.11515533	-0.89660276
H	-1.27744424	-3.07874782	-0.00018706
H	0.27827116	-3.11525093	0.89634580

B3LYP/cc-pVDZ_r7

N	-2.57593979	-1.31764723	-0.00348552
C	-3.76632336	-0.65081900	0.00613232
O	-4.85137420	-1.25532581	0.00878831
N	-3.75176291	0.74091195	0.01281459
C	-2.62958772	1.57074529	0.00922146
O	-2.75666115	2.79629627	0.01449765
C	-1.35925814	0.85182948	-0.00005579
N	-0.21643409	1.56805633	-0.00656811
C	0.94575364	0.84508606	-0.00699654
C	2.17118177	1.52723897	-0.00775168
C	3.41478499	0.85701100	-0.00358253
C	4.68631728	1.62826496	-0.01705628
C	3.42672430	-0.58635566	0.00801131
C	4.71197129	-1.34098586	0.02558411
C	2.19608474	-1.27131732	0.00620956
C	0.98826491	-0.58458351	-0.00340217
N	-0.26672441	-1.29275983	-0.00779063
C	-1.42757341	-0.58825163	-0.00286997
C	-0.24830468	-2.74682258	-0.01825287
H	-4.65685499	1.20381429	0.01936553
H	2.13886391	2.61725189	-0.01182532
H	5.28999401	1.38260087	-0.91154335
H	4.50814224	2.71039898	-0.00119985
H	5.32778718	1.35897661	0.84253391
H	5.30864568	-1.09543066	0.92413131
H	4.54441631	-2.42539870	0.00829175
H	5.35168440	-1.07080150	-0.83441309
H	2.21739610	-2.35848223	0.01374393
H	0.29086165	-3.10449942	-0.91009293
H	-1.27980753	-3.10238402	-0.02962877
H	0.27418198	-3.11900247	0.87774666

B3LYP/cc-pVTZ_S0

N	-2.55230018	-1.29759083	-0.00006819
C	-3.74000667	-0.62988822	-0.00012026
O	-4.82595697	-1.19275826	-0.00016980
N	-3.72010108	0.77597337	-0.00021650
C	-2.61486022	1.58455061	0.00006667
O	-2.69049287	2.79988998	0.00030830
C	-1.33735867	0.82377412	0.00007697
N	-0.22138876	1.48664007	0.00009285
C	0.94559469	0.79528485	0.00004452
C	2.16266755	1.50066422	-0.00001981
C	3.37440868	0.85353650	-0.00005709
C	4.66011681	1.62810639	-0.00032593
C	3.38558605	-0.56903965	-0.00000658
C	4.68905091	-1.30847858	0.00018495
C	2.19766929	-1.27920179	0.00001433
C	0.96480796	-0.62020525	0.00005627
N	-0.23774308	-1.29716460	0.00007351
C	-1.42742595	-0.62131560	0.00005685
C	-0.23691170	-2.76417106	0.00002973
H	-4.62263468	1.23082287	0.00006941
H	2.10765438	2.58018777	-0.00002139
H	5.26450785	1.39143110	-0.87793507
H	4.46766693	2.69852945	0.00157671
H	5.26674150	1.38863005	0.87493829
H	5.28479906	-1.04681408	0.87679964
H	4.53395819	-2.38449165	-0.00171615
H	5.28695878	-1.04390490	-0.87403555
H	2.23884343	-2.35586138	-0.00004127
H	0.27367361	-3.12667477	-0.88913612
H	-1.26410234	-3.09892558	0.00035573

H	0.27423146	-3.12668812	0.88886238

B3LYP/cc-pVTZ_S1(r1)

N	2.51986189	-1.28811878	-0.00002912
C	3.74696038	-0.64605758	-0.00054014
O	4.79206117	-1.28555101	0.00046188
N	3.75043154	0.72930168	-0.00013167
C	2.62985581	1.55934958	-0.00001237
O	2.76922385	2.77932689	-0.00002517
C	1.36290584	0.85341557	-0.00014463
N	0.21760612	1.56229709	-0.00011960
C	-0.93383238	0.84341638	-0.00006737
C	-2.15630550	1.50732406	-0.00001461
C	-3.38640428	0.83671383	0.00002574
C	-4.65670788	1.61651219	0.00024027
C	-3.40901414	-0.58162861	-0.00000687
C	-4.70676656	-1.33072721	-0.00015488
C	-2.20514841	-1.26364648	0.00000604
C	-0.96877732	-0.59210353	-0.00001222
N	0.22141268	-1.27625335	0.00004589
C	1.41273274	-0.56859585	-0.00001778
C	0.22536275	-2.74043129	0.00019200
H	4.65114573	1.18616633	0.00001843
H	-2.13498725	2.58778062	-0.00001902
H	-5.26483876	1.36871490	0.87430198
H	-4.46938826	2.68682609	-0.00100193
H	-5.26648335	1.36683737	-0.87209738
H	-5.30819482	-1.08214978	-0.87725804
H	-4.53703431	-2.40496717	0.00104431
H	-5.30952993	-1.08034718	0.87548913
H	-2.22364837	-2.34127865	0.00002555
H	-0.28630448	-3.10574161	0.88891214
H	1.25101100	-3.07796374	0.00018376
H	-0.28637925	-3.10591698	-0.88841022

B3LYP/cc-pVTZ_r7

N	-2.57396880	-1.22496776	-0.00003244
C	-3.78876322	-0.64042112	-0.00010487
O	-4.84495183	-1.26636444	0.00004288
N	-3.75511531	0.74984232	-0.00040262
C	-2.62292403	1.56794229	-0.00014548
O	-2.72558940	2.79016070	-0.00010852
C	-1.37121540	0.84204802	0.00003922
N	-0.18713705	1.49342666	0.00032932
C	0.98076232	0.85502906	0.00026801
C	2.22627013	1.51078455	0.00020736
C	3.42187313	0.82064456	0.00001395
C	4.72424698	1.57119482	0.00007908
C	3.41135103	-0.59083382	-0.00022142
C	4.69078132	-1.37343296	-0.00042192
C	2.18631343	-1.25299260	-0.00017924
C	0.97770427	-0.58229428	0.00014979
N	-0.25224199	-1.25791081	0.00025982
C	-1.41230276	-0.55679196	-0.00000321
C	-0.28733871	-2.72020082	0.00032745
H	-4.65165642	1.21677455	-0.00047372
H	2.21194852	2.59143573	0.00027328
H	5.32335882	1.31801175	-0.87654193
H	4.55653936	2.64570454	0.00027033
H	5.32343840	1.31772231	0.87656316
H	5.29905810	-1.13902901	0.87580958
H	4.49652634	-2.44368232	-0.00105293
H	5.29941598	-1.13804495	-0.87612793
H	2.17939842	-2.33506189	-0.00043915
H	0.20409175	-3.10544973	-0.89144633
H	-1.32538078	-3.04021715	0.00163470
H	0.20628226	-3.10532128	0.89092514

B3LYP/cc-pVQZ_S0

N	-2.55223236	-1.29676069	-0.00018371
C	-3.73802172	-0.62993726	-0.00026630
O	-4.82426575	-1.19231777	-0.00026191
N	-3.71982518	0.77526897	-0.00028182
C	-2.61534841	1.58314154	0.00013960
O	-2.69237779	2.79780066	0.00055454
C	-1.33701816	0.82344867	0.00005531
N	-0.22115294	1.48526552	0.00007748
C	0.94541291	0.79597992	0.00000976
C	2.16262610	1.50151684	-0.00009347
C	3.37349150	0.85390583	-0.00011401
C	4.65966535	1.62720612	-0.00035604
C	3.38443936	-0.56875928	-0.00003425
C	4.68783311	-1.30763871	0.00016505
C	2.19691622	-1.27891652	0.00004551
C	0.96449843	-0.61973642	0.00006944
N	-0.23782139	-1.29640420	0.00011490
C	-1.42642067	-0.62121041	-0.00000347
C	-0.23533831	-2.76302814	0.00024869
H	-4.62202857	1.22947797	0.00003272
H	2.10898002	2.58061330	-0.00011034
H	5.26330138	1.38962060	-0.87733017
H	4.46829866	2.69712941	0.00123575
H	5.26519973	1.38727960	0.87463588
H	5.28299017	-1.04542698	0.87613640
H	4.53313221	-2.38301740	-0.00144798
H	5.28486464	-1.04296070	-0.87374247
H	2.23816639	-2.35499931	0.00006535
H	0.27605079	-3.12431005	-0.88811253
H	-1.26083986	-3.10061418	0.00018891
H	0.27584169	-3.12407568	0.88882449

B3LYP/cc-pVQZ_S1(r1)

N	-2.51926608	-1.28721357	-0.00008762
C	-3.74563140	-0.64613321	-0.00012263
O	-4.79033061	-1.28532674	0.00004482
N	-3.75057568	0.72820714	0.00002633
C	-2.63057138	1.55768738	0.00009553
O	-2.77132920	2.77706587	0.00020728
C	-1.36285617	0.85337698	-0.00001117
N	-0.21914928	1.56029184	-0.00003582
C	0.93381918	0.84334440	-0.00003754
C	2.15488867	1.50799466	-0.00008136
C	3.38557271	0.83718137	-0.00003062
C	4.65542656	1.61669633	-0.00021167
C	3.40851428	-0.58072669	0.00008690
C	4.70655480	-1.32903115	0.00028727
C	2.20529365	-1.26301802	0.00008365
C	0.96858751	-0.59124089	0.00000206
N	-0.22065878	-1.27572354	-0.00005651
C	-1.41272010	-0.56964411	-0.00004750
C	-0.22187952	-2.73959316	-0.00013277
H	-4.65085689	1.18467626	0.00012525
H	2.13423616	2.58796445	-0.00014498
H	5.26309219	1.36812718	-0.87351657
H	4.46864083	2.68642791	0.00064687
H	5.26428707	1.36683120	0.87185829
H	5.30722537	-1.07968358	0.87677470
H	4.53725810	-2.40261484	-0.00051908
H	5.30825864	-1.07848921	-0.87512938
H	2.22409777	-2.34007651	0.00013825
H	0.29028154	-3.10356467	-0.88829800
H	-1.24580044	-3.08013937	-0.00024403
H	0.29011423	-3.10366840	0.88808632

B3LYP/cc-pVQZ_r6

N	0.10046030	0.72596883	-0.33455649
C	-0.24090886	2.00147733	-0.37585198
O	-1.38857759	2.46548088	-0.18367466
N	0.75376475	2.96259478	-0.61784991
C	2.09458093	2.71640102	-0.38467303
O	2.85020491	3.70101457	-0.28131018
C	2.47050106	1.33585247	-0.29831150
N	3.78025087	1.01483168	-0.18420466
C	4.07146136	-0.30233700	-0.16469910
C	5.41269240	-0.71788339	-0.04140693
C	5.78034951	-2.04844535	-0.02503097
C	7.22479163	-2.43697568	0.10762415
C	4.77719760	-3.03807135	-0.13408169
C	5.13456971	-4.49514775	-0.12289200
C	3.44563957	-2.65212604	-0.25204334
C	3.07301335	-1.31175952	-0.27033706
N	1.73461150	-0.92036561	-0.38254277
C	1.40955716	0.40672459	-0.35362384
C	0.69946502	-1.94405512	-0.49216242
H	0.46306526	3.93348667	-0.65320711
H	6.16135904	0.05741815	0.04038151
H	7.55589487	-3.02861779	-0.74749935
H	7.86032001	-1.55772318	0.17877221
H	7.39010036	-3.04986543	0.99529719
H	5.64803292	-4.77046265	0.80021960
H	4.24805521	-5.11767792	-0.21576741
H	5.81173703	-4.74364143	-0.94220584
H	2.69577019	-3.42178227	-0.33269745
H	0.88942958	-2.56291942	-1.36658287
H	-0.25873113	-1.45604429	-0.58983479
H	0.70033808	-2.57229612	0.39693851

B3LYP/aug-cc-pVDZ_S0

N	-2.56257617	-1.30685871	-0.00010108
C	-3.74890211	-0.63243048	0.00005562
O	-4.84529488	-1.19760165	-0.00003080
N	-3.73274218	0.77668510	-0.00017066
C	-2.62368266	1.58746132	0.00019290
O	-2.70474333	2.81111737	0.00043633
C	-1.34319177	0.82744080	0.00005882
N	-0.22150016	1.49589260	0.00001725
C	0.94708578	0.80078832	-0.00003937
C	2.17098363	1.50966665	-0.00012721
C	3.38833709	0.85708141	-0.00011000
C	4.68044424	1.62899114	-0.00049786
C	3.39787264	-0.57330033	0.00011579
C	4.70667705	-1.31149926	0.00060621
C	2.20373961	-1.28740031	0.00007968
C	0.96614840	-0.62232607	-0.00002199
N	-0.23984642	-1.30059608	-0.00013092
C	-1.43161915	-0.62179829	-0.00007308
C	-0.23759521	-2.77067435	-0.00025445
H	-4.63942706	1.23283141	-0.00007011
H	2.11767250	2.59735322	-0.00018697
H	5.28685423	1.38741537	-0.88509851
H	4.49001890	2.70766169	0.00172330
H	5.28957187	1.38410558	0.88127125
H	5.30432791	-1.04516061	0.88420971
H	4.55261015	-2.39546886	-0.00172673
H	5.30731379	-1.04158832	-0.87982068
H	2.24542591	-2.37189818	0.00016295
H	0.27766370	-3.13071497	-0.89704274
H	-1.27084769	-3.10959881	-0.00014301
H	0.27799072	-3.13092697	0.89626482

B3LYP/aug-cc-pVDZ_S1(r1)

N	2.52924920	-1.29679805	0.00020383
C	3.75633205	-0.64892512	0.00006313
O	4.81013308	-1.29223047	0.00012015
N	3.76415974	0.73014180	-0.00013692
C	2.64045531	1.56220713	-0.00017376
O	2.78542780	2.79075775	-0.00045786
C	1.36845103	0.85814671	-0.00002396
N	0.22085278	1.57109891	-0.00001694
C	-0.93577947	0.84720947	0.00001918
C	-2.16323945	1.51564235	0.00004970
C	-3.40062633	0.84072618	0.00001634
C	-4.67651359	1.61933244	0.00029457
C	-3.42249378	-0.58471464	-0.00015737
C	-4.72593385	-1.33268748	-0.00050263
C	-2.21244223	-1.27069919	-0.00007715
C	-0.97053584	-0.59306100	0.00004214
N	0.22289648	-1.28041637	0.00019005
C	1.41750360	-0.57144921	0.00012882
C	0.22373823	-2.74786084	0.00040099
H	4.66958075	1.18726548	-0.00028778
H	-2.14261281	2.60429615	0.00009871
H	-5.28701748	1.36624729	0.88095568
H	-4.49049273	2.69777237	-0.00084864
H	-5.28868555	1.36450320	-0.87866011
H	-5.32962454	-1.07926526	-0.88434658
H	-4.55696308	-2.41485280	0.00062228
H	-5.33116085	-1.07759167	0.88177813
H	-2.23150827	-2.35623042	-0.00012935
H	-0.29259045	-3.11063520	0.89677628
H	1.25527852	-3.09019961	0.00061785
H	-0.29229192	-3.11091250	-0.89603487

B3LYP/aug-cc-pVDZ_r5

N	-2.57720373	-1.23813387	-0.00005169
C	-3.79881040	-0.63235595	-0.00024506
O	-4.86231970	-1.25854183	0.00051961
N	-3.78054137	0.75721342	-0.00074743
C	-2.65627980	1.57786038	-0.00033211
O	-2.75475664	2.81135385	0.00009080
C	-1.40329949	0.85144131	0.00012565
N	-0.20775932	1.42414085	0.00033765
C	0.98714509	0.82588849	0.00070245
C	2.21334978	1.52289288	0.00007934
C	3.42725739	0.84482929	-0.00004147
C	4.72191910	1.61434252	-0.00023377
C	3.43430524	-0.57298528	-0.00002772
C	4.73359341	-1.33172653	-0.00018029
C	2.21889420	-1.27155000	0.00014891
C	0.99297555	-0.60855945	0.00045809
N	-0.24112500	-1.27578258	0.00010458
C	-1.42693475	-0.59799573	-0.00009942
C	-0.24949790	-2.74227415	-0.00026657
H	-4.68241877	1.22150130	-0.00038602
H	2.18087026	2.61042181	-0.00019633
H	5.32830228	1.36726204	-0.88350029
H	4.53854145	2.69435718	-0.00006230
H	5.32881553	1.36703924	0.88261309
H	5.34057031	-1.08147554	0.88237319
H	4.55951416	-2.41336704	-0.00033197
H	5.34050405	-1.08119261	-0.88268979
H	2.24545880	-2.35746496	0.00016957
H	0.26108105	-3.11209586	-0.89682560
H	-1.28700448	-3.07620108	-0.00033651
H	0.26107742	-3.11219212	0.89625979

B3LYP/aug-cc-pVDZ_r6/r7

N	2.57009644	-1.30662642	0.08033047
C	3.72938581	-0.65815580	0.05789614
O	4.86140443	-1.15207095	0.31292313
N	3.73626881	0.71887334	-0.23734279
C	2.61143073	1.52147431	-0.08741170
O	2.79440629	2.76031225	-0.02674669
C	1.35225832	0.83092838	-0.03090053
N	0.20101886	1.55314085	0.00557497
C	-0.94999472	0.83664374	0.00832865
C	-2.19275152	1.51465292	0.05225751
C	-3.40953778	0.84541822	0.04830752
C	-4.70518530	1.61147410	0.09659229
C	-3.41400519	-0.57485691	-0.00125915
C	-4.71256115	-1.33504526	-0.01126236
C	-2.19871677	-1.26648310	-0.04048795
C	-0.97289705	-0.59255995	-0.03646700
N	0.24783734	-1.28340193	-0.06675266
C	1.42756655	-0.58602667	-0.02013128
C	0.23972818	-2.74637394	-0.10993466
H	4.64053226	1.18863883	-0.25130545
H	-2.16241603	2.60313216	0.08922412
H	-5.29695377	1.33683857	0.98212327
H	-4.52210482	2.69132822	0.12773142
H	-5.32991162	1.39298788	-0.78182514
H	-5.32931942	-1.05826589	-0.87922449
H	-4.53764507	-2.41614169	-0.04635145
H	-5.31139433	-1.11247398	0.88428832
H	-2.22477050	-2.35154904	-0.07606240
H	-0.25818521	-3.14243486	0.78362109
H	1.26867050	-3.09709808	-0.14265646
H	-0.29785858	-3.08133366	-1.00480914

B3LYP/aug-cc-pVTZ_S0

N	-2.55306296	-1.29777165	-0.00029762
C	-3.73766182	-0.62999095	-0.00030907
O	-4.82708184	-1.19271429	-0.00040148
N	-3.72003812	0.77524688	-0.00007063
C	-2.61494316	1.58264919	0.00017704
O	-2.69324444	2.79963686	0.00047197
C	-1.33721864	0.82353313	0.00004149
N	-0.22062694	1.48624476	0.00010962
C	0.94570289	0.79653759	0.00004113
C	2.16351172	1.50193218	-0.00000417
C	3.37442652	0.85408959	-0.00008048
C	4.66062614	1.62728244	-0.00041080
C	3.38499226	-0.56920695	-0.00005287
C	4.68790768	-1.30869759	0.00014394
C	2.19703171	-1.27906349	-0.00001719
C	0.96463198	-0.61963878	0.00001987
N	-0.23792607	-1.29635430	0.00010015
C	-1.42633321	-0.62082189	-0.00006354
C	-0.23522170	-2.76387175	0.00042551
H	-4.62254462	1.22991552	0.00033286
H	2.11021368	2.58153068	0.00004532
H	5.26385212	1.39008419	-0.87850621
H	4.46940829	2.69780185	0.00221722
H	5.26688914	1.38620419	0.87447363
H	5.28292276	-1.04716683	0.87717171
H	4.53277139	-2.38457785	-0.00242275
H	5.28578266	-1.04322736	-0.87368601
H	2.23819509	-2.35562729	-0.00012253
H	0.27642821	-3.12445535	-0.88869504
H	-1.26114319	-3.10180814	0.00070162
H	0.27669909	-3.12401017	0.88957050

B3LYP/aug-cc-pVTZ_S1(r1)

N	-2.51970556	-1.28800152	-0.00012789
C	-3.74542866	-0.64629442	-0.00011508
O	-4.79281127	-1.28629163	-0.00000406
N	-3.75113332	0.72813698	0.00007221
C	-2.63056548	1.55682158	0.00007516
O	-2.77251930	2.77897879	0.00035507
C	-1.36305722	0.85380568	-0.00002255
N	-0.21976589	1.56142989	-0.00004926
C	0.93419844	0.84331144	-0.00004698
C	2.15489495	1.50802040	-0.00011245
C	3.38660943	0.83724907	-0.00005594
C	4.65569723	1.61750969	-0.00036117
C	3.40966887	-0.58089574	0.00014262
C	4.70738763	-1.32962239	0.00046308
C	2.20609974	-1.26271768	0.00012697
C	0.96879294	-0.59074908	0.00000337
N	-0.22027757	-1.27599224	-0.00008687
C	-1.41291350	-0.56990521	-0.00008232
C	-0.22113588	-2.74075991	-0.00020015
H	-4.65175030	1.18501047	0.00022282
H	2.13469136	2.58849075	-0.00020434
H	5.26348016	1.36911783	-0.87444355
H	4.46854391	2.68771864	0.00097444
H	5.26538563	1.36710567	0.87176640
H	5.30798373	-1.08037162	0.87775622
H	4.53781675	-2.40372340	-0.00074802
H	5.30956936	-1.07858065	-0.87519831
H	2.22462210	-2.34025769	0.00020622
H	0.29135758	-3.10400269	-0.88909439
H	-1.24555766	-3.08150803	-0.00032120
H	0.29118748	-3.10414923	0.88873096

B3LYP/aug-cc-pVTZ_r5

N	-2.55683411	1.28748457	0.00002212
C	-3.75285604	0.64865646	-0.00002503
O	-4.83939737	1.23475898	-0.00016682
N	-3.73764859	-0.74483391	-0.00003678
C	-2.61626887	-1.55482530	-0.00002179
O	-2.72372244	-2.78476031	-0.00009687

C	-1.36382017	-0.82746690	-0.00000697
N	-0.21755778	-1.53594636	0.00004569
C	0.95897306	-0.83418597	0.00005186
C	2.17687025	-1.53761601	0.00006936
C	3.31705121	-0.78436238	-0.00000129
C	4.72662342	-1.66088423	0.00018115
C	3.43133257	0.58315535	-0.00009679
C	4.71678142	1.35310873	-0.00034966
C	2.19353534	1.26911937	-0.00005709
C	0.98000393	0.58458713	0.00004407
N	-0.23406099	1.27510304	0.00013039
C	-1.42640869	0.58541118	0.00003597
C	-0.23270340	2.73289527	0.00029306
H	-4.63492775	-1.20726611	-0.00009700
H	2.15557039	-2.61658320	0.00012146
H	5.20617052	-1.33289786	0.91850141
H	4.47429343	-2.71084724	-0.00065133
H	5.20711944	-1.33169154	-0.91720886
H	5.30815661	1.11548959	-0.88736801
H	4.53266156	2.42430395	0.00045371
H	5.30921809	1.11432977	0.88563858
H	2.22904053	2.34559031	-0.00012419
H	0.27407167	3.10744380	0.88891495
H	-1.26213801	3.06625912	0.00051709
H	0.27374808	3.10767245	-0.88841942

B3LYP/aug-cc-pVTZ_r6

N	-2.56215812	-1.29814706	-0.07988862
C	-3.71573279	-0.65423023	-0.06211278
O	-4.84107559	-1.14427790	-0.32527695
N	-3.72320872	0.71743917	0.23723546
C	-2.60141259	1.51516546	0.09057700
O	-2.77873386	2.74875589	0.03422144
C	-1.34731650	0.82539018	0.03039745
N	-0.20024005	1.54330647	-0.00545371
C	0.94774224	0.83249029	-0.00816763
C	2.18463108	1.50686241	-0.05335624
C	3.39489879	0.84205413	-0.05013088
C	4.68456167	1.60962305	-0.10014579
C	3.40047025	-0.57093909	0.00001520
C	4.69314020	-1.33229119	0.00920722
C	2.19136498	-1.25843117	0.04093541
C	0.97088166	-0.59023650	0.03833804
N	-0.24665428	-1.27945866	0.07044730
C	-1.42231428	-0.58472739	0.02077703
C	-0.23867997	-2.73947495	0.11796540
H	-4.62314676	1.18561577	0.25473689
H	2.15431831	2.58715106	-0.09093494
H	5.27283562	1.33844432	-0.97916433
H	4.50026481	2.68121462	-0.13204861
H	5.30671704	1.39618115	0.77136438
H	5.30758558	-1.05969068	0.87009212
H	4.51718354	-2.40520500	0.04477288
H	5.28822002	-1.11440486	-0.88026826
H	2.21695461	-2.33555216	0.07700136
H	0.25030226	-3.13838538	-0.76977022
H	-1.26029074	-3.08848479	0.15704388
H	0.29995101	-3.07221745	1.00343933

B3LYP/aug-cc-pVTZ_r7

N	-2.56788153	-1.30084600	-0.00037230
C	-3.73669704	-0.63444172	-0.00020560
O	-4.83357248	-1.23862950	-0.00011753
N	-3.73664855	0.74526627	0.00027251
C	-2.61125601	1.56115737	0.00033552
O	-2.74201711	2.78697763	0.00078212
C	-1.34939771	0.84771858	0.00005142
N	-0.20456443	1.55116578	-0.00000649
C	0.95264174	0.83563522	-0.00009202
C	2.17536157	1.51276871	-0.00023096
C	3.40538723	0.84259383	-0.00020579
C	4.67610363	1.60943004	-0.00113394
C	3.41251544	-0.59210161	0.00032676
C	4.69028363	-1.35246253	0.00140461
C	2.18848439	-1.27017642	0.00020832
C	0.98831285	-0.58505209	-0.00005357
N	-0.26359033	-1.28513182	-0.00023962
C	-1.41383885	-0.58278259	-0.00017490
C	-0.24541094	-2.73944457	-0.00059663
H	-4.63424056	1.20681398	0.00053003
H	2.15137932	2.59286317	-0.00045318
H	5.28897767	1.35666953	-0.87310759
H	4.49787381	2.68084615	0.00059567
H	5.29221726	1.35407772	0.86770597
H	5.30080290	-1.10275632	0.87532243
H	4.51483205	-2.42484297	-0.00041572
H	5.30421837	-1.10008982	-0.86926459
H	2.20617430	-2.34726347	0.00044965
H	0.28006526	-3.09679218	-0.88693587
H	-1.26349320	-3.09928836	-0.00085322
H	0.27976386	-3.09725549	0.88573164

B3P86/cc-pVDZ_S0

N	0.11112983	0.70905769	-0.48271203
C	-0.23415688	2.03341100	-0.50470047
O	-1.39073845	2.41868287	-0.60145483
N	0.78542828	2.99619613	-0.41081942
C	2.13320585	2.77118962	-0.30085700
O	2.95131869	3.67223947	-0.22558546
C	2.47792457	1.32557331	-0.28413669
N	3.73459089	0.99552633	-0.18528760
C	4.04478229	-0.32777484	-0.16914854
C	5.39778569	-0.71075986	-0.06516376
C	5.77973122	-2.03662081	-0.04346614
C	7.22240345	-2.42360856	0.06842166
C	4.76765826	-3.03776457	-0.12940381
C	5.14882780	-4.48391190	-0.10923506
C	3.42944496	-2.67901867	-0.23200460
C	3.04453735	-1.33101091	-0.25396378
N	1.72555262	-0.94321705	-0.35577184
C	1.37968699	0.38353292	-0.37922046
C	0.67961219	-1.95670907	-0.43968025
H	0.48002933	3.96610506	-0.42961125
H	6.13565198	0.09050179	-0.00232623
H	7.54187411	-3.03027624	-0.79341233
H	7.86611667	-1.53651856	0.12021988
H	7.40396088	-3.03296660	0.96774964
H	5.69355249	-4.73375359	0.81489098
H	4.26847211	-5.13437380	-0.17948501
H	5.82600560	-4.72322387	-0.94437171
H	2.68100656	-3.46627008	-0.29628453
H	0.83960201	-2.58058089	-1.32844456
H	-0.27706220	-1.43561200	-0.51268660
H	0.70106149	-2.58499068	0.46013762

B3P86/cc-pVDZ_S1(r1)

N	0.14068853	0.70049059	-0.47410551
C	-0.24517294	2.03379157	-0.50219113
O	-1.42887820	2.33839767	-0.59607304
N	0.73920405	2.99266387	-0.42128377
C	2.11213683	2.77058559	-0.31169774
O	2.89100117	3.71787030	-0.24776235
C	2.48497891	1.36687547	-0.28367237
N	3.79381764	1.04491386	-0.17776269
C	4.07042360	-0.28282594	-0.16408203
C	5.40016376	-0.70326937	-0.05925416
C	5.77355906	-2.05464866	-0.04141969
C	7.20953566	-2.43158647	0.07354355
C	4.77045144	-3.06129290	-0.13337925
C	5.14005771	-4.51012043	-0.11920875
C	3.44146730	-2.66961620	-0.23606841
C	3.06670288	-1.31174490	-0.25244521
N	1.74908403	-0.93303993	-0.35140122
C	1.43136326	0.41263101	-0.37168799
C	0.70147849	-1.94140986	-0.43150137
H	0.43686529	3.96265919	-0.44430872
H	6.16190245	0.07457790	0.00957306
H	7.52460081	-3.04705410	-0.78543127
H	7.85815462	-1.54940993	0.12943596
H	7.38158219	-3.05258790	0.96854937
H	5.67686536	-4.77804536	0.80514031
H	4.25035696	-5.14789248	-0.19410003
H	5.81236209	-4.76057564	-0.95575523
H	2.67596883	-3.44049603	-0.30585394
H	0.85946843	-2.56888436	-1.31933707
H	-0.25758525	-1.42613155	-0.50234484
H	0.72639175	-2.57177139	0.46807135

B3P86/cc-pVDZ_r7

N	0.09610323	0.71300076	-0.47745567
C	-0.25324146	2.02720295	-0.51006227
O	-1.43632295	2.38209890	-0.60932831
N	0.74671143	2.98424763	-0.42843439
C	2.11407439	2.76167205	-0.31257977
O	2.89908065	3.70552444	-0.24641616
C	2.48371003	1.35456477	-0.28217614
N	3.78620333	1.03760434	-0.17167874
C	4.07533400	-0.29497217	-0.15769971
C	5.41163161	-0.69832778	-0.05535328
C	5.79615853	-2.05433921	-0.03972135
C	7.22372666	-2.42667487	0.07968297
C	4.77670912	-3.06603627	-0.13790450
C	5.12990719	-4.50689376	-0.12997584
C	3.43496978	-2.66108971	-0.24054078
C	3.08705934	-1.31905987	-0.24764294
N	1.71516742	-0.91637956	-0.34930765
C	1.41149697	0.40273384	-0.37262535
C	0.69389160	-1.93645474	-0.42135288
H	0.44720646	3.95473050	-0.45380143
H	6.16815658	0.08415170	0.01266738
H	7.54536996	-3.05654451	-0.76970197
H	7.87357140	-1.54596123	0.13263145
H	7.39897349	-3.05078605	0.97542301
H	5.68171500	-4.78173907	0.78675161
H	4.23938400	-5.14225656	-0.20267660
H	5.80758394	-4.75942799	-0.96540683
H	2.67185493	-3.43302534	-0.31504483
H	0.85722404	-2.56739707	-1.30908098
H	-0.27691997	-1.44153536	-0.48308927
H	0.74250589	-2.57557711	0.47408704

B3PW91/cc-pVDZ_S0

N	0.10936577	0.71084426	-0.48310965
C	-0.23648148	2.03639796	-0.50515676
O	-1.39381686	2.42126804	-0.60220485
N	0.78399436	2.99982841	-0.41088847
C	2.13276763	2.77432299	-0.30073375
O	2.95153662	3.67558684	-0.22528798
C	2.47745040	1.32699423	-0.28408825
N	3.73465201	0.99647609	-0.18513411
C	4.04493064	-0.32811797	-0.16906658
C	5.39894459	-0.71107248	-0.06499575
C	5.78193302	-2.03771065	-0.04327656
C	7.22674096	-2.42301334	0.06880560
C	4.76928838	-3.03975463	-0.12934192
C	5.14946738	-4.48793184	-0.10941071
C	3.43010772	-2.68067011	-0.23200862
C	3.04407984	-1.33192936	-0.25398264
N	1.72401776	-0.94352736	-0.35581448
C	1.37818995	0.38444079	-0.37935487
C	0.67668077	-1.95770806	-0.43968433
H	0.47898038	3.96986396	-0.42968006
H	6.13671367	0.09067911	-0.00210378
H	7.54704597	-3.02926888	-0.79345933
H	7.86933571	-1.53460965	0.12040922
H	7.40905300	-3.03174736	0.96884187
H	5.69384613	-4.73844669	0.81513384
H	4.26806154	-5.13764394	-0.17959911
H	5.82600952	-4.72778477	-0.94535465
H	2.68216796	-3.46858676	-0.29628829
H	0.83615482	-2.58123609	-1.32923338
H	-0.28042267	-1.43684513	-0.51227984
H	0.69820113	-2.58604397	0.46053398

B3PW91/cc-pVDZ_S1(r1)

N	0.13864893	0.70217314	-0.47426775
C	-0.24759219	2.03674556	-0.50247980
O	-1.43202994	2.34114715	-0.59642609
N	0.73774379	2.99605336	-0.42157154
C	2.11182156	2.77341491	-0.31182793
O	2.89141665	3.72094422	-0.24790175
C	2.48425500	1.36817671	-0.28371387
N	3.79398729	1.04544182	-0.17762716
C	4.07062505	-0.28319429	-0.16395907
C	5.40158762	-0.70362032	-0.05893532
C	5.77585918	-2.05554045	-0.04115097
C	7.21392767	-2.43127187	0.07411497
C	4.77201079	-3.06301828	-0.13341743
C	5.14064466	-4.51379955	-0.11962441
C	3.44194901	-2.67124859	-0.23620136
C	3.06607214	-1.31283379	-0.25250102
N	1.74756239	-0.93327713	-0.35140964
C	1.42988471	0.41355311	-0.37175163
C	0.69845566	-1.94225576	-0.43139068
H	0.43572994	3.96615691	-0.44467376
H	6.16316062	0.07478069	0.01004033
H	7.52984887	-3.04614697	-0.78535790
H	7.86156257	-1.54787688	0.12994718
H	7.38656074	-3.05158900	0.96986464
H	5.67725049	-4.78229856	0.80509422
H	4.25000173	-5.15092850	-0.19451529
H	5.81247491	-4.76452891	-0.95694216
H	2.67713454	-3.44297151	-0.30606293
H	0.85604915	-2.56949378	-1.31989933
H	-0.26099955	-1.42715482	-0.50197102
H	0.72340006	-2.57248721	0.46870373

B3PW91/cc-pVDZ_r6

N	0.13892903	0.70242189	-0.47389665
C	-0.24723605	2.03695997	-0.50313241
O	-1.43183158	2.34118246	-0.59595146
N	0.73818826	2.99623112	-0.42466078
C	2.11233608	2.77351126	-0.31472564
O	2.89197558	3.72113919	-0.25256279
C	2.48471787	1.36846141	-0.28510213
N	3.79437672	1.04549536	-0.17893510
C	4.07081880	-0.28312248	-0.16451055
C	5.40174071	-0.70373564	-0.05961570
C	5.77574731	-2.05568015	-0.04136301
C	7.21375468	-2.43176785	0.07369924
C	4.77172722	-3.06321457	-0.13295131
C	5.14032800	-4.51397612	-0.11879143
C	3.44168795	-2.67130002	-0.23540966
C	3.06610612	-1.31282946	-0.25204468
N	1.74751115	-0.93295668	-0.35022958
C	1.43032952	0.41383150	-0.37171210
C	0.69818494	-1.94194668	-0.42835405
H	0.43598570	3.96626311	-0.44843565
H	6.16321497	0.07481158	0.00879167
H	7.52902441	-3.04774108	-0.78519611
H	7.86177869	-1.54856433	0.12826946
H	7.38642400	-3.05100830	0.97018196
H	5.67767902	-4.78219262	0.80556454
H	4.24949435	-5.15098332	-0.19264616
H	5.81137391	-4.76522991	-0.95658144
H	2.67679066	-3.44297037	-0.30478508
H	0.85450365	-2.56934039	-1.31698041
H	-0.26134006	-1.42683281	-0.49763917
H	0.72468239	-2.57186464	0.47189144

B3PW91/cc-pVDZ_r7

N	0.09412347	0.71471882	-0.47765401
C	-0.25568445	2.03005519	-0.51209551
O	-1.43922309	2.38487403	-0.61263034
N	0.74554058	2.98758997	-0.43065138
C	2.11382446	2.76458204	-0.31320691
O	2.89957379	3.70860005	-0.24661376
C	2.48305903	1.35586170	-0.28173554
N	3.78620686	1.03843310	-0.16975577
C	4.07545422	-0.29529294	-0.15605603
C	5.41277400	-0.69849738	-0.05402019
C	5.79842245	-2.05528629	-0.03933243
C	7.22784514	-2.42639294	0.08105793
C	4.77835548	-3.06789911	-0.13903196
C	5.13091606	-4.51054929	-0.13253039
C	3.43563065	-2.66268615	-0.24164009
C	3.08650223	-1.32007190	-0.24695041
N	1.71353342	-0.91668436	-0.34809450
C	1.40998388	0.40350925	-0.37240370
C	0.69065091	-1.93741617	-0.41863232
H	0.44647226	3.95821775	-0.45676623
H	6.16921674	0.08442708	0.01432034
H	7.55052885	-3.05599737	-0.76850511
H	7.87683584	-1.54459536	0.13422327
H	7.40299709	-3.04982748	0.97768567
H	5.68280728	-4.78641766	0.78421353
H	4.23962606	-5.14545002	-0.20550633
H	5.80796468	-4.76246787	-0.96910380
H	2.67324993	-3.43539453	-0.31743845
H	0.85155090	-2.56760579	-1.30775944
H	-0.28059722	-1.44253974	-0.47814272
H	0.74086243	-2.57674517	0.47694188

B98/cc-pVDZ_S0

N	0.09883393	0.71833452	-0.48335145
C	-0.24729666	2.04932992	-0.50558646
O	-1.40480872	2.43458575	-0.60226540
N	0.77735255	3.01617573	-0.41183441
C	2.13045090	2.78971898	-0.30048741
O	2.94965113	3.69089509	-0.22434716
C	2.47568272	1.33515515	-0.28366804
N	3.73463644	1.00248027	-0.18453963
C	4.04583763	-0.32675974	-0.16860062
C	5.40493450	-0.70984651	-0.06422478
C	5.78916878	-2.04033407	-0.04267822
C	7.24247853	-2.42673095	0.06980056
C	4.77371636	-3.04597014	-0.12908490
C	5.15686306	-4.50230099	-0.10910913
C	3.42983412	-2.68731643	-0.23205502
C	3.04151106	-1.33357315	-0.25402759
N	1.71640175	-0.94311883	-0.35639624
C	1.37055544	0.38905639	-0.37952652
C	0.66399518	-1.96378071	-0.44133983
H	0.47201435	3.98697593	-0.43051824
H	6.14300320	0.09281457	-0.00123696
H	7.56303770	-3.03335358	-0.79423012
H	7.88372520	-1.53534114	0.12111935
H	7.42519804	-3.03577034	0.97158077
H	5.70097425	-4.75259082	0.81761925
H	4.27342328	-5.15186958	-0.18012462
H	5.83451102	-4.74148459	-0.94651080
H	2.68272282	-3.47700044	-0.29621024
H	0.82783045	-2.58796114	-1.33139006
H	-0.29501877	-1.44507341	-0.51505504
H	0.68777640	-2.59229208	0.46046477

B98/cc-pVDZ_S1(r1)

N	0.12886894	0.70949312	-0.47553179
C	-0.25896983	2.04981594	-0.50338030
O	-1.44313975	2.35511063	-0.59723038
N	0.73060951	3.01185839	-0.42177034
C	2.10919614	2.78818019	-0.31146044
O	2.88965215	3.73526139	-0.24716918
C	2.48174713	1.37615172	-0.28348011
N	3.79490261	1.05179432	-0.17684100
C	4.07260401	-0.28097227	-0.16340971
C	5.40810369	-0.70178449	-0.05796624
C	5.78322385	-2.05796017	-0.04050003
C	7.22958788	-2.43553884	0.07525533
C	4.77604351	-3.06937985	-0.13330646
C	5.14816694	-4.52802105	-0.11945931

C 3.44142185 -2.67858813 -0.23647873
C 3.06289022 -1.31487136 -0.25282624
N 1.74075978 -0.93385813 -0.35217046
C 1.42231947 0.41924979 -0.37247259
C 0.68574195 -1.94838104 -0.43266781
H 0.42824201 3.98276912 -0.44469137
H 6.17007903 0.07738495 0.01129362
H 7.54565222 -3.04995691 -0.78653559
H 7.87603403 -1.54930496 0.13118660
H 7.40219417 -3.05596181 0.97290293
H 5.68606114 -4.79549650 0.80677570
H 4.25567489 -5.16538051 -0.19387853
H 5.82041072 -4.77789447 -0.95888208
H 2.67708187 -3.45176279 -0.30637203
H 0.84680944 -2.57688385 -1.32140991
H -0.27488164 -1.43393359 -0.50485985
H 0.71190872 -2.57808518 0.46952207

B98/cc-pVDZ_r6

N 0.08277030 0.72735311 -0.47957704
C -0.26918601 2.02013348 -0.50090656
O -1.41041345 2.47983663 -0.59075853
N 0.75987611 3.01611064 -0.41159577
C 2.08345096 2.70438320 -0.30739108
O 2.88833907 3.68053832 -0.23700742
C 2.45468532 1.33267670 -0.28626761
N 3.78331665 1.03397706 -0.18291705
C 4.06759889 -0.29366721 -0.16830271
C 5.42108489 -0.70967199 -0.06373900
C 5.79550055 -2.04842345 -0.04217081
C 7.24955749 -2.43776782 0.07122916
C 4.78515937 -3.04700285 -0.12820971
C 5.14969812 -4.50999846 -0.10865834
C 3.44074306 -2.66214656 -0.23187210
C 3.06195088 -1.31313731 -0.25356944
N 1.71389129 -0.92926123 -0.35761110
C 1.38441175 0.40183527 -0.37697496
C 0.67387858 -1.95292132 -0.44416495
H 0.47207359 3.99270866 -0.42893773
H 6.17306809 0.08053998 0.00054128
H 7.57107580 -3.04399888 -0.79326889
H 7.89452576 -1.54870990 0.12531488
H 7.43155335 -3.05000071 0.97146213
H 5.68612898 -4.77750537 0.81861564
H 4.25541746 -5.14578778 -0.18186734
H 5.82292341 -4.76696376 -0.94529250
H 2.68433922 -3.44387991 -0.29666734
H 0.83839049 -2.58006762 -1.33368816
H -0.29339038 -1.44883749 -0.51873613
H 0.69657703 -2.58728977 0.45517498

B98/cc-pVDZ_r7

N 0.08146044 0.72255588 -0.47896821
C -0.26542246 2.04223714 -0.51062752
O -1.45074452 2.39908810 -0.60973916
N 0.73757865 3.00354338 -0.42853468
C 2.11057129 2.77858945 -0.31264563
O 2.89707800 3.72268377 -0.24667105
C 2.48008583 1.36367145 -0.28208675
N 3.78753677 1.04400735 -0.17127956
C 4.07776992 -0.29283377 -0.15737683
C 5.42011401 -0.69697524 -0.05369956
C 5.80607933 -2.05749315 -0.03842148
C 7.24397192 -2.43204929 0.08129791
C 4.78259002 -3.07393872 -0.13750713
C 5.13909860 -4.52465352 -0.13006532
C 3.43519138 -2.66914254 -0.24074244
C 3.08459243 -1.32194494 -0.24811900
N 1.70575330 -0.91558977 -0.35023588
C 1.40327344 0.40808741 -0.37323960
C 0.67781943 -1.94286314 -0.42295333
H 0.43777039 3.97475915 -0.45421811
H 6.17708370 0.08659420 0.01528444
H 7.56513515 -3.06024993 -0.77164672
H 7.89223247 -1.54765972 0.13387526
H 7.41866069 -3.05458158 0.98032630
H 5.69074234 -4.79863564 0.78921557
H 4.24593074 -5.15976709 -0.20273090
H 5.81615185 -4.77575688 -0.96888047
H 2.67311221 -3.44330078 -0.31496589
H 0.84524579 -2.57415627 -1.31162888
H -0.29530489 -1.45070133 -0.48633130
H 0.72783841 -2.58047036 0.47550173

BHHLYP/cc-pVDZ_S0

N -2.54467413 -1.29613534 -0.00015460
C -3.72847971 -0.62054294 0.00020223
O -4.80361461 -1.17809345 -0.00062157
N -3.70804912 0.77608756 0.00191065
C -2.60800277 1.57971948 0.00034839
O -2.67566538 2.78453665 -0.00107654
C -1.33252599 0.82032515 0.00006280
N -0.22729071 1.48055380 -0.00003679
C 0.93981589 0.78780059 -0.00010962
C 2.15297674 1.49619810 -0.00003400
C 3.36510760 0.85323893 0.00001738
C 4.64888564 1.62712920 -0.00010544
C 3.37589556 -0.56672583 0.00009420
C 4.67965317 -1.30257785 0.00036136
C 2.19067466 -1.27721966 0.00007142
C 0.95693306 -0.61876708 -0.00017442
N -0.24263692 -1.29727702 -0.00033091
C -1.42480181 -0.62722782 0.00004670
C -0.24035430 -2.75668455 -0.00032206
H -4.60892154 1.22898971 0.00093935
H 2.09252064 2.57772720 0.00006313
H 5.25432962 1.39300861 -0.88087920
H 4.45558945 2.70037237 0.00324097
H 5.25772133 1.38800410 0.87691058
H 5.27502024 -1.04052483 0.88012966
H 4.52718499 -2.38184919 -0.00260848
H 5.27807789 -1.03598238 -0.87589739
H 2.23391823 -2.35607684 0.00034431
H 0.27277591 -3.12166210 -0.89038943
H -1.26998198 -3.09235066 -0.00211423
H 0.26989504 -3.12179898 0.89137349

BHHLYP/cc-pVDZ_S1(r1)

N -2.51610802 -1.27477740 -0.00033193
C -3.73656011 -0.62702965 -0.00028511
O -4.77711139 -1.24870771 -0.00028632
N -3.73278714 0.74558811 0.00054809
C -2.62031744 1.56332119 0.00071287
O -2.73707964 2.77294531 0.00114757
C -1.35458249 0.84806877 0.00021432
N -0.21639121 1.55542868 0.00021139
C 0.92314680 0.84402011 0.00007884
C 2.14977493 1.51026807 0.00002239
C 3.36676356 0.84330801 0.00006111
C 4.64294840 1.61337676 -0.00031026
C 3.38939980 -0.58413327 0.00025850
C 4.68940858 -1.32266252 0.00074783
C 2.19363666 -1.27051861 0.00015179
C 0.95641744 -0.60467240 -0.00001621
N -0.22226732 -1.28267712 -0.00032799
C -1.41827683 -0.56901625 -0.00014283
C -0.23301779 -2.73799873 -0.00076704
H -4.63183639 1.20134276 0.00075849
H 2.12045122 2.59242236 -0.00001818
H 5.24789517 1.36362341 -0.87827218
H 4.45963646 2.68738129 0.00125968
H 5.25011592 1.36128447 0.87539229
H 5.29043029 -1.06936492 0.88024168
H 4.52708823 -2.40078416 -0.00078791
H 5.29254848 -1.06709283 -0.87659281
H 2.21665420 -2.35021034 0.00021998
H 0.27803364 -3.10971134 -0.89090442
H -1.26332107 -3.07054379 -0.00128875
H 0.27728580 -3.11028379 0.88956704

BHHLYP/cc-pVDZ_r4

N -2.55503770 -1.28903673 -0.00058870
C -3.74206340 -0.64763160 -0.00069154
O -4.82257957 -1.20217862 -0.00182036
N -3.73857883 0.75044706 0.00042741
C -2.59493948 1.48549587 0.00053017
O -2.69956409 2.75467747 0.00127779
C -1.33852202 0.83703486 0.00046765
N -0.21800447 1.53172920 0.00072197
C 0.94154698 0.81898323 0.00048036
C 2.16466991 1.50391791 0.00054446
C 3.37874332 0.84807349 0.00032759
C 4.66429186 1.62129578 0.00046557
C 3.38696296 -0.56157543 -0.00004671
C 4.68307144 -1.31516746 -0.00059273
C 2.18420099 -1.25443335 -0.00010925
C 0.96168823 -0.58810071 0.00011868
N -0.25509989 -1.27724424 0.00007759
C -1.43146980 -0.61159955 0.00011588
C -0.24418799 -2.73317982 0.00002670
H -4.63089313 1.21833719 -0.00017662
H 2.12162275 2.58670102 0.00074750
H 5.27269100 1.38400690 -0.87788911
H 4.47481551 2.69557936 0.00070267
H 5.27258361 1.38369447 0.87882152
H 5.28838159 -1.06557822 0.87667741
H 4.51406856 -2.39277580 0.00022270
H 5.28677812 -1.06675637 -0.87933578
H 2.21482912 -2.33437266 -0.00027403
H 0.27122435 -3.09858500 -0.88948910
H -1.26979369 -3.08097776 -0.00043798
H 0.27054246 -3.09858802 0.88996022

BHHLYP/aug-cc-pVDZ_S0

N 0.12780667 0.71180516 -0.48322996
C -0.20798261 2.02421654 -0.50530892
O -1.35854778 2.41338850 -0.60371582
N 0.79774336 2.98682955 -0.41099304
C 2.13385201 2.75702555 -0.29921989
O 2.94435812 3.65305679 -0.22243654
C 2.48133273 1.31635472 -0.28204380
N 3.72101530 0.98010648 -0.18331903
C 4.03977803 -0.33473970 -0.16707551
C 5.38983110 -0.71236850 -0.06342271
C 5.76697715 -2.02963177 -0.04359331
C 7.21001446 -2.41798361 0.06745297
C 4.75768017 -3.02567634 -0.13049831
C 5.13938069 -4.47220393 -0.11213609
C 3.42762758 -2.67083345 -0.23225255
C 3.04544472 -1.32672811 -0.25264816
N 1.72912521 -0.93765599 -0.35464385
C 1.38919732 0.37239090 -0.37827370
C 0.69011428 -1.96121853 -0.43813458
H 0.49491763 3.94557981 -0.42980380
H 6.12387143 0.07880037 -0.00012030
H 7.52457985 -3.01482201 -0.79042565
H 7.84336120 -1.53515923 0.11959587
H 7.38800413 -3.02030641 0.96009340
H 5.67588686 -4.71819256 0.80592147
H 4.26292797 -5.11169075 -0.18266922
H 5.80732692 -4.70562004 -0.94320042
H 2.68666221 -3.45021318 -0.29725152
H 0.85752008 -2.57215800 -1.32178321
H -0.26699667 -1.46703205 -0.50625419
H 0.72618653 -2.58226653 0.45357617

BHHLYP/aug-cc-pVDZ_S1(r1)

N 0.16560340 0.70680818 -0.47537642
C -0.21770356 2.02844610 -0.50402802
O -1.39035249 2.34450716 -0.60043294
N 0.75935897 2.98528229 -0.42038105
C 2.11422316 2.75539242 -0.30869060
O 2.89570321 3.69097623 -0.24193470
C 2.48807567 1.35398864 -0.28123848
N 3.77940758 1.02749274 -0.17528830
C 4.06824809 -0.28721108 -0.16158677
C 5.39325265 -0.69940137 -0.05717637
C 5.76010737 -2.04022514 -0.04167360
C 7.19459212 -2.42181817 0.07192382
C 4.75616944 -3.04936099 -0.13500127
C 5.13429641 -4.49525807 -0.12183748
C 3.43653174 -2.67010977 -0.23683303
C 3.05584889 -1.31704380 -0.25235577
N 1.75407265 -0.94384232 -0.35004822
C 1.43197709 0.40889467 -0.37178225
C 0.70788034 -1.95463363 -0.42966643
H 0.46075526 3.94488539 -0.44336832
H 6.14967719 0.06901726 0.01206476
H 7.50146311 -3.02658383 -0.78467104
H 7.83403625 -1.54492486 0.12819965
H 7.36003266 -3.03529121 0.96089074
H 5.66624361 -4.75452499 0.79606804
H 4.25234717 -5.12715012 -0.19604160
H 5.79969592 -4.73557333 -0.95393800
H 2.68091537 -3.43525684 -0.30675111
H 0.86489346 -2.57130761 -1.31286837
H -0.24630998 -1.45504415 -0.49628670
H 0.73795460 -2.57807620 0.46229330

BHHLYP/aug-cc-pVDZ_r4

N 0.12550631 0.72551073 -0.47907489
C -0.23504895 2.02074817 -0.50168470
O -1.39018152 2.41012908 -0.59522612
N 0.75654892 2.99432357 -0.41337359
C 2.08059184 2.68298313 -0.30719896
O 2.91309241 3.64268545 -0.23439274
C 2.48868958 1.33188648 -0.28252910
N 3.76193329 1.01245270 -0.18187337
C 4.06201704 -0.31641287 -0.16647091
C 5.40117904 -0.71538030 -0.06294991
C 5.77215695 -2.04342126 -0.04292019
C 7.21635485 -2.43185293 0.06964038
C 4.76985058 -3.03023148 -0.12973662
C 5.13381858 -4.48409024 -0.11222702
C 3.44036384 -2.65068704 -0.23211810
C 3.07088223 -1.30891620 -0.25162254
N 1.73302641 -0.91640235 -0.35495312
C 1.39312403 0.38529619 -0.37690661
C 0.70341258 -1.94416994 -0.43948127
H 0.47222643 3.95770791 -0.43220910
H 6.14790952 0.06432673 0.00166313
H 7.53289153 -3.02892927 -0.78784000
H 7.85193922 -1.55025931 0.12345405
H 7.39433822 -3.03573561 0.96168682
H 5.66277940 -4.74629472 0.80629849
H 4.24710028 -5.11036510 -0.18513247
H 5.79743691 -4.73352829 -0.94272374
H 2.69021775 -3.42210455 -0.29782269
H 0.87400768 -2.55701692 -1.32209689
H -0.25951084 -1.46124104 -0.50914872
H 0.74034330 -2.56795693 0.45115399

BHHLYP/aug-cc-pVDZ_r5

N 0.13702182 0.72025598 -0.48572666
C -0.20620457 2.02760597 -0.50743580
O -1.37123572 2.40694229 -0.60637719
N 0.79219798 3.00628481 -0.40992852
C 2.13578559 2.77705543 -0.29623246
O 2.93334862 3.69027313 -0.22062212
C 2.50192759 1.35257260 -0.27897240
N 3.74131805 1.01627315 -0.18145485
C 4.04997077 -0.30177618 -0.16694635
C 5.38137564 -0.69679592 -0.06495320
C 5.76446118 -2.02628662 -0.04450899
C 7.19845823 -2.40546317 0.06633886
C 4.74096935 -3.05720053 -0.13207461
C 5.14563128 -4.49490361 -0.11214049
C 3.43005974 -2.70717556 -0.23133650
C 3.02384921 -1.33927883 -0.25428944
N 1.74810967 -0.96585965 -0.35367111
C 1.40752098 0.37060308 -0.37709708
C 0.68823196 -1.97292721 -0.43814829
H 0.48505457 3.96224295 -0.42876930
H 6.12839315 0.08265588 -0.00076510
H 7.51495089 -3.01272795 -0.78715634
H 7.83292662 -1.52384149 0.11637442
H 7.37913787 -3.01390171 0.95793309
H 5.68634282 -4.73511700 0.80545840
H 4.27631059 -5.14459914 -0.18040438
H 5.81463512 -4.72318718 -0.94433856
H 2.68446276 -3.48295498 -0.29585423
H 0.84948994 -2.58533901 -1.32107850
H -0.25863299 -1.46013147 -0.50940901
H 0.71312863 -2.59024428 0.45577015

BHHLYP/aug-cc-pVDZ_T1

N 0.16257100 0.70938300 -0.47376100
C -0.21829600 2.03621700 -0.50139200
O -1.39032200 2.34561200 -0.59599000
N 0.75775700 2.99174400 -0.41917800
C 2.11207800 2.75396900 -0.30963500
O 2.89580400 3.68345900 -0.24427200
C 2.48292700 1.35008700 -0.28252200
N 3.78968400 1.02887200 -0.17643000
C 4.06677200 -0.28981200 -0.16353900
C 5.39941900 -0.70449700 -0.05951300
C 5.76164900 -2.03357500 -0.04287300
C 7.19675400 -2.42318300 0.07013700
C 4.75267100 -3.04723600 -0.13434600
C 5.13413600 -4.49279000 -0.11975100
C 3.43733200 -2.66985400 -0.23513400
C 3.05894900 -1.31759800 -0.25203800
N 1.75037500 -0.94577000 -0.35025300
C 1.43047800 0.40733700 -0.37150100
C 0.70608700 -1.95859900 -0.43047000
H 0.46201500 3.95224400 -0.44132500
H 6.15300100 0.06677300 0.00810100
H 7.50027600 -3.02662400 -0.78764000
H 7.83838400 -1.54778400 0.12625300
H 7.35947800 -3.03531500 0.95957700
H 5.66636800 -4.74880700 0.79834700
H 4.25329600 -5.12617400 -0.19337200
H 5.79970000 -4.73115000 -0.95166400
H 2.68226900 -3.43559500 -0.30344400
H 0.86216500 -2.57376400 -1.31424100
H -0.25000100 -1.46243900 -0.49675700
H 0.73521400 -2.58207600 0.46081400

BHHLYP/aug-cc-pVDZ_T2

N 0.14860700 0.76693500 -0.52901500
C -0.24939100 2.06468900 -0.54246100
O -1.41379800 2.40361800 -0.64053300
N 0.74737300 3.01483000 -0.44921400
C 2.09265500 2.76778800 -0.31982200
O 2.90950400 3.67240800 -0.23787000
C 2.42662400 1.37404200 -0.28508900
N 3.67388100 0.91272900 -0.13099900
C 4.07157000 -0.34893900 -0.13281700
C 5.42724200 -0.73272900 -0.03392200
C 5.79294200 -2.05490700 -0.03467000
C 7.23942900 -2.44641400 0.07389600
C 4.79076100 -3.03272300 -0.13934400
C 5.15216900 -4.48645400 -0.14705100
C 3.44932600 -2.65995600 -0.23910300
C 3.06153100 -1.33207300 -0.23351400
N 1.72458300 -0.92303400 -0.32680100
C 1.38678300 0.38205800 -0.38207800
C 0.68782500 -1.94357100 -0.38915900
H 0.45770200 3.97702500 -0.47328700
H 6.16448700 0.05361100 0.04188900
H 7.55799600 -3.02643700 -0.79370600

H	7.87360200	-1.56477900	0.14260900
H	7.41710600	-3.06159600	0.95743700
H	5.68277300	-4.76065000	0.76673400
H	4.26652800	-5.11243500	-0.22982200
H	5.81623600	-4.71823100	-0.98202400
H	2.70597400	-3.43635600	-0.32684400
H	0.81536100	-2.54929000	-1.28471600
H	-0.27378200	-1.44861800	-0.41990500
H	0.74539100	-2.57748400	0.49338600

BLYP/cc-pVDZ_S0

N	-2.58088044	-1.32171952	-0.00008994
C	-3.78779354	-0.64060084	-0.00009354
O	-4.88638115	-1.21281338	-0.00050317
N	-3.76033481	0.78538687	0.00032543
C	-2.63970984	1.60883319	0.00014266
O	-2.71647236	2.84249051	-0.00012623
C	-1.35297585	0.83615171	0.00004047
N	-0.22138385	1.51807649	0.00008370
C	0.95376013	0.80600925	0.00007187
C	2.18627266	1.51601709	0.00007450
C	3.41590380	0.86028892	-0.00000965
C	4.71393447	1.63826026	-0.00008434
C	3.42764010	-0.57868813	-0.00008788
C	4.74422200	-1.32063696	-0.00018086
C	2.22283341	-1.29795157	-0.00003385
C	0.97574720	-0.63051886	0.00004464
N	-0.23900022	-1.31468192	0.00011016
C	-1.44996422	-0.62402479	0.00000334
C	-0.24680691	-2.79390463	0.00033082
H	-4.67557486	1.24882010	0.00026907
H	2.12761397	2.61243639	0.00013770
H	5.33097876	1.39323383	-0.88931148
H	4.52655033	2.72749184	0.00083049
H	5.33199456	1.39186294	0.88803369
H	5.35250907	-1.05016790	0.88783465
H	4.59540392	-2.41540044	-0.00112037
H	5.35335819	-1.04868530	-0.88713120
H	2.26613493	-2.39146247	-0.00008611
H	0.27159350	-3.16630627	-0.90231374
H	-1.29654618	-3.11809444	0.00048585
H	0.27162671	-3.16598655	0.90309209

BLYP/cc-pVDZ_S1(r2)

N	-2.55955654	-1.31666499	0.00014062
C	-3.79586053	-0.65050052	-0.00012075
O	-4.85443756	-1.30770305	-0.00057350
N	-3.79488294	0.73980766	-0.00016604
C	-2.65235859	1.58949879	0.00023273
O	-2.80097461	2.82482787	0.00011460
C	-1.38007960	0.87122986	0.00041517
N	-0.21067124	1.59623363	0.00039962
C	0.94293311	0.85623875	0.00033538
C	2.19076989	1.52555028	0.00022057
C	3.42850784	0.84519606	0.00004875
C	4.72004270	1.62103884	-0.00012447
C	3.44392508	-0.58910397	-0.00007325
C	4.75122313	-1.34743959	-0.00026286
C	2.21922664	-1.27852788	0.00002167
C	0.97884484	-0.59219412	0.00026187
N	-0.24072119	-1.28138727	0.00034698
C	-1.42701112	-0.56746525	0.00051999
C	-0.24577705	-2.76099125	0.00020090
H	-4.70769957	1.20531846	-0.00038809
H	2.16437524	2.62323522	0.00026179
H	5.33875036	1.36385214	-0.88620505
H	4.54267678	2.71125413	0.00014054
H	5.33926186	1.36347289	0.88547941
H	5.36679961	-1.09328350	0.88823244
H	4.58271139	-2.43987121	-0.00053787
H	5.36684046	-1.09282029	-0.88858727
H	2.23947322	-2.37302518	-0.00008344
H	0.27876410	-3.12692910	-0.90199047
H	-1.28999911	-3.09805914	0.00038138
H	0.27915747	-3.12707073	0.90209767

BLYP/cc-pVDZ_r7

N	-2.58972983	-1.32624362	-0.00527015
C	-3.80068681	-0.65825813	0.00631399
O	-4.89214698	-1.26739950	0.01005577
N	-3.77969637	0.75069290	0.01374785
C	-2.64673215	1.59072951	0.00833923
O	-2.77313565	2.82958196	0.01368558
C	-1.36686917	0.86228177	-0.00298922
N	-0.22186873	1.58705186	-0.01349735
C	0.95710827	0.84182135	-0.01148484
C	2.18498326	1.53261666	-0.00924930
C	3.44483748	0.86212619	-0.00118608
C	4.71830556	1.64254895	-0.01924090
C	3.45649133	-0.59097622	0.01516471
C	4.74507783	-1.35172478	0.04120495
C	2.21371253	-1.28368836	0.01075505
C	0.99580936	-0.59215790	-0.00582158
N	-0.26262209	-1.30746697	-0.01333884
C	-1.44363181	-0.59602393	-0.00563911
C	-0.25087377	-2.77267557	-0.03023553
H	-4.69120885	1.21966785	0.02099941
H	2.14807119	2.62940383	-0.01457287
H	5.32796742	1.40000159	-0.92104618
H	4.53757554	2.73112892	-0.00018318
H	5.37643820	1.37077543	0.83745886
H	5.34200485	-1.10541904	0.94964947
H	4.57938589	-2.44312112	0.01944732
H	5.40249426	-1.07423628	-0.81335431
H	2.23778012	-2.37737538	0.02320719
H	0.29294199	-3.13408572	-0.92639196
H	-1.29340442	-3.11856596	-0.04838201
H	0.26587559	-3.15729273	0.87259304

BLYP/cc-pVDZ_r8

N	-2.55955654	-1.31666499	0.00014062
C	-3.79586053	-0.65050052	-0.00012075
O	-4.85443756	-1.30770305	-0.00057350
N	-3.79488294	0.73980766	-0.00016604
C	-2.65235859	1.58949879	0.00023273
O	-2.80097461	2.82482787	0.00011460
C	-1.38007960	0.87122986	0.00041517
N	-0.21067124	1.59623363	0.00039962
C	0.94293311	0.85623875	0.00033538
C	2.19076989	1.52555028	0.00022057
C	3.42850784	0.84519606	0.00004875
C	4.72004270	1.62103884	-0.00012447
C	3.44392508	-0.58910397	-0.00007325
C	4.75122313	-1.34743959	-0.00026286
C	2.21922664	-1.27852788	0.00002167
C	0.97884484	-0.59219412	0.00026187
N	-0.24072119	-1.28138727	0.00034698
C	-1.42701112	-0.56746525	0.00051999
C	-0.24577705	-2.76099125	0.00020090
H	-4.70769957	1.20531846	-0.00038809
H	2.16437524	2.62323522	0.00026179
H	5.33875036	1.36385214	-0.88620505
H	4.54267678	2.71125413	0.00014054
H	5.33926186	1.36347289	0.88547941
H	5.36679961	-1.09328350	0.88823244
H	4.58271139	-2.43987121	-0.00053787
H	5.36684046	-1.09282029	-0.88858727
H	2.23947322	-2.37302518	-0.00008344
H	0.27876410	-3.12692910	-0.90199047
H	-1.28999911	-3.09805914	0.00038138
H	0.27915747	-3.12707073	0.90209767

BLYP/cc-pVDZ_T1

N	0.10192900	0.72828700	-0.47720800
C	-0.28653700	2.07593800	-0.50340500
O	-1.48432800	2.39619000	-0.59714100
N	0.71994000	3.04306100	-0.42018900
C	2.10630800	2.81197000	-0.31139400
O	2.90595300	3.76092800	-0.24625400
C	2.47311500	1.38441600	-0.28542500
N	3.79908500	1.06197200	-0.18008600
C	4.07643800	-0.28008600	-0.16622500
C	5.42265100	-0.70253400	-0.06016700
C	5.79915700	-2.05946600	-0.04085300
C	7.25087000	-2.44257400	0.07545600
C	4.78533900	-3.08127600	-0.13220100
C	5.16599500	-4.54296600	-0.11545700
C	3.44293300	-2.69430400	-0.23601400
C	3.05997200	-1.32826500	-0.25464400
N	1.72766800	-0.94859400	-0.35395700
C	1.40320800	0.43862600	-0.37414700
C	0.66260300	-1.96451600	-0.43482600
H	0.42026800	4.02353100	-0.44174900
H	6.18533600	0.08396900	0.00783700
H	7.56805700	-3.06405300	-0.78848900
H	7.90497500	-1.55446400	0.13046600
H	7.42444100	-3.06706700	0.97744000
H	5.71077000	-4.80815000	0.81484300
H	4.27405300	-5.19126300	-0.18957100
H	5.84655500	-4.79288000	-0.95637800
H	2.67802400	-3.47438500	-0.30422500
H	0.81884200	-2.59818900	-1.32838100
H	-0.29967900	-1.44014800	-0.50588500
H	0.68505000	-2.60065400	0.47041700

BLYP/cc-pVDZ_T6

N	0.07629100	0.72902100	-0.48184700
C	-0.27818000	2.06449500	-0.50358400
O	-1.45855500	2.44686800	-0.59799400
N	0.74879500	3.05219700	-0.41337500
C	2.12568600	2.82129400	-0.30326200
O	2.92872600	3.76715600	-0.23161800
C	2.48948200	1.38332400	-0.28400100
N	3.76745800	1.05572800	-0.18488200
C	4.07767400	-0.29030800	-0.16835800
C	5.41490600	-0.68822600	-0.06574900
C	5.81175500	-2.04590100	-0.04221800
C	7.25901900	-2.42834200	0.07195600
C	4.76254900	-3.09321600	-0.13097100
C	5.16799500	-4.54554400	-0.10886800
C	3.43325400	-2.72913900	-0.23239400
C	3.02918000	-1.34887600	-0.25621700
N	1.71776100	-0.95229900	-0.35682100
C	1.37456600	0.39765200	-0.37879100
C	0.64284600	-1.97119500	-0.44257700
H	0.44095100	4.03051600	-0.43202500
H	6.16980300	0.10755700	-0.00202300
H	7.58984100	-3.05060300	-0.78921800
H	7.90956100	-1.53613500	0.12258800
H	7.45035400	-3.04844600	0.97618900
H	5.72208600	-4.79610200	0.82077300
H	4.28845000	-5.21144300	-0.17840300
H	5.85409400	-4.78599400	-0.94892400
H	2.67571000	-3.51778600	-0.29638000
H	0.80785600	-2.60137200	-1.33480100
H	-0.31537700	-1.44050900	-0.51873500
H	0.66445300	-2.60131800	0.46471700

BLYP/cc-pVDZ_T7

N	0.06599400	0.73119700	-0.47526200
C	-0.29568500	2.06516400	-0.50206900
O	-1.49466000	2.42076600	-0.59373400
N	0.72070000	3.03434200	-0.42256000
C	2.10795600	2.80869400	-0.31336500
O	2.90564300	3.76115600	-0.24953500
C	2.46786700	1.38023200	-0.28721600
N	3.78445800	1.05100400	-0.18358100
C	4.08193600	-0.28593800	-0.16675700
C	5.43082100	-0.68916100	-0.06089800
C	5.82231000	-2.06736900	-0.03931700
C	7.26599900	-2.44577100	0.08197100
C	4.78242000	-3.09034200	-0.13451500
C	5.15219300	-4.54429800	-0.12095400
C	3.43332000	-2.70203300	-0.23866200
C	3.06236500	-1.32672000	-0.25452400
N	1.72032500	-0.93017600	-0.35369400
C	1.39488200	0.42950300	-0.37392800
C	0.66046200	-1.95294200	-0.43298400
H	0.41918400	4.01406100	-0.44503900
H	6.19026000	0.09985500	0.00553400
H	7.59825100	-3.07713800	-0.77294000
H	7.91773800	-1.55559700	0.13265700
H	7.45119900	-3.06785600	0.98724600
H	5.71067500	-4.80900900	0.80399100
H	4.26322100	-5.19531600	-0.19383600
H	5.84162100	-4.79154100	-0.95847700
H	2.67196200	-3.48425900	-0.30901800
H	0.82424600	-2.58461500	-1.32650800
H	-0.30421100	-1.43300600	-0.50099100
H	0.69554100	-2.58983100	0.47115500

BMK/cc-pVDZ_S0

N	0.10164975	0.71891374	-0.48449097
C	-0.24191292	2.05002639	-0.50593605
O	-1.39129445	2.43396607	-0.60412534
N	0.77883226	3.01447719	-0.40853743
C	2.12748072	2.79245903	-0.29838404
O	2.94170478	3.68659348	-0.22253488
C	2.47668767	1.33479889	-0.28280534
N	3.72680805	1.00222268	-0.18449291
C	4.04264089	-0.32433436	-0.16879690
C	5.40393388	-0.70509144	-0.06462666
C	5.78282755	-2.03646905	-0.04346374
C	7.23414653	-2.43803995	0.06873877
C	4.76588975	-3.04024648	-0.12958419
C	5.16108879	-4.49558309	-0.10811531
C	3.42190860	-2.68548338	-0.23283695
C	3.03713248	-1.32761089	-0.25445111
N	1.71626314	-0.93804292	-0.35678863
C	1.36818769	0.38467741	-0.38008054
C	0.67615355	-1.96368836	-0.44180286
H	0.47064242	3.98423211	-0.42745908
H	6.14275354	0.09903167	-0.00188966
H	7.54195761	-3.05127697	-0.79663347
H	7.88434000	-1.55128194	0.11720174
H	7.40563472	-3.04717658	0.97400467
H	5.71034344	-4.73366233	0.82016211
H	4.28119662	-5.15250379	-0.17617890
H	5.83982613	-4.72475010	-0.94896912
H	2.67393774	-3.47566942	-0.29749889
H	0.84802470	-2.58430251	-1.33450692
H	-0.29142364	-1.45931043	-0.51301545
H	0.71163466	-2.59382104	0.46008385

BMK/cc-pVDZ_S1(r1/r5)

N	0.13724884	0.71146362	-0.47579817
C	-0.25261771	2.05098485	-0.50314798
O	-1.42639508	2.35864036	-0.59744990
N	0.73626501	3.01064026	-0.41882982
C	2.10637149	2.79045867	-0.30793929
O	2.88654309	3.72615907	-0.24308117
C	2.48153187	1.37281075	-0.28199594
N	3.78738532	1.04935956	-0.17665540
C	4.07052133	-0.27580185	-0.16348066
C	5.40955470	-0.69484959	-0.05851856
C	5.77641000	-2.04853538	-0.04156469
C	7.22049534	-2.44446205	0.07320522
C	4.76578688	-3.06311079	-0.13347297
C	5.15430961	-4.51894033	-0.11679726
C	3.43054203	-2.68102091	-0.23709132
C	3.05185566	-1.31520720	-0.25398863
N	1.74066668	-0.93740279	-0.35308777
C	1.41831249	0.41798217	-0.37280654
C	0.69422977	-1.95127955	-0.43537129
H	0.43168176	3.98099059	-0.44143276
H	6.17131307	0.08646526	0.00982996
H	7.52175600	-3.06600708	-0.79011446
H	7.87730866	-1.56385383	0.12532457
H	7.38073730	-3.06386617	0.97519013
H	5.70062732	-4.77132676	0.81016511
H	4.26685916	-5.16616564	-0.18551317
H	5.82711338	-4.75819526	-0.96046532
H	2.66751250	-3.45687097	-0.30646075
H	0.85943208	-2.57650080	-1.32771233
H	-0.27182164	-1.44467869	-0.50458137
H	0.72746703	-2.58482813	0.46582780

BMK/aug-cc-pVDZ_S0

N	0.10151279	0.72323626	-0.48408171
C	-0.23706930	2.04782961	-0.50523013
O	-1.39180658	2.43811172	-0.60262548
N	0.77920953	3.01558681	-0.41046061
C	2.12588709	2.79047601	-0.29879959
O	2.93866477	3.69082988	-0.22173879
C	2.47636122	1.33408025	-0.28273303
N	3.72547831	0.99688239	-0.18444340
C	4.04323288	-0.32518977	-0.16858231
C	5.40562011	-0.70593165	-0.06396481
C	5.78396893	-2.03627780	-0.04292431
C	7.23733080	-2.43481481	0.07016979
C	4.76506383	-3.04130076	-0.13005931
C	5.15678720	-4.49844584	-0.10926831
C	3.42192745	-2.68599120	-0.23352638
C	3.03713707	-1.32834801	-0.25472760
N	1.71668662	-0.93711988	-0.35706765
C	1.37245871	0.38296754	-0.37963874
C	0.67640831	-1.96660476	-0.44216345
H	0.47183440	3.98341114	-0.42849113
H	6.14541244	0.09456896	-0.00075899
H	7.54647874	-3.04361068	-0.79450108
H	7.88007552	-1.54598983	0.11930454
H	7.40896087	-3.04108140	0.97410067
H	5.70222205	-4.73758285	0.81786523
H	4.27463558	-5.14763311	-0.17809444
H	5.83233785	-4.72804367	-0.94910165
H	2.67480221	-3.47383532	-0.29861298
H	0.85290873	-2.58227422	-1.33418164
H	-0.29249448	-1.47245499	-0.51278914
H	0.71696300	-2.59239636	0.45931225

BMK/aug-cc-pVDZ_S1(r1)

N	0.13987108	0.71665506	-0.47544211
C	-0.24796802	2.05197656	-0.50195905
O	-1.42677356	2.36301552	-0.59598651
N	0.73623364	3.01377720	-0.41901806
C	2.10416646	2.78920738	-0.30905434
O	2.88445617	3.73172549	-0.24391472
C	2.48315286	1.37467079	-0.28227281
N	3.78390165	1.04773699	-0.17714868
C	4.07141172	-0.27911216	-0.16340406
C	5.40790753	-0.69442540	-0.05813758
C	5.77737803	-2.05044042	-0.04096408
C	7.22296724	-2.43989883	0.07476923
C	4.76644904	-3.06581530	-0.13380050
C	5.15075704	-4.52408219	-0.11821304
C	3.43263838	-2.68268817	-0.23738909
C	3.05254709	-1.31562814	-0.25406393
N	1.74188872	-0.93849126	-0.35329233
C	1.41929758	0.41770525	-0.37299103
C	0.69541141	-1.95628220	-0.43556470
H	0.43361506	3.98255704	-0.44112196
H	6.17145653	0.08244640	0.01077125
H	7.52619961	-3.05807707	-0.78728221
H	7.87153089	-1.55660937	0.12696804
H	7.38417802	-3.05648205	0.97563794
H	5.69173741	-4.77822267	0.80814911
H	4.26079933	-5.16291967	-0.18902199
H	5.82091296	-4.76341986	-0.96036648
H	2.66961279	-3.45535667	-0.30711772
H	0.86380637	-2.57729461	-1.32698230
H	-0.27118560	-1.45863206	-0.50566688

H 0.73063994 -2.58454378 0.46606707
BMK/aug-cc-pVDZ_r4
N 0.09832962 0.73646450 -0.48000517
C -0.26313509 2.03792298 -0.50110650
O -1.42110539 2.43686913 -0.59302502
N 0.73829134 3.02020886 -0.41235354
C 2.06971247 2.71623229 -0.30708159
O 2.88907507 3.68498985 -0.23463036
C 2.48106253 1.34919845 -0.28408662
N 3.76979963 1.02902897 -0.18366533
C 4.06778521 -0.30459368 -0.16845332
C 5.41987778 -0.70933372 -0.06371501
C 5.79086898 -2.05047521 -0.04224433
C 7.24522718 -2.45057514 0.07281699
C 4.77819266 -3.04565821 -0.12933762
C 5.14984451 -4.51096203 -0.10927342
C 3.43518207 -2.66400550 -0.23363458
C 3.06337589 -1.30933571 -0.25418120
N 1.72211378 -0.91455173 -0.35793561
C 1.37965500 0.39891590 -0.37855312
C 0.69049280 -1.94797895 -0.44363790
H 0.44942093 3.99304091 -0.42987189
H 6.17341465 0.07855568 0.00080914
H 7.55612935 -3.06020273 -0.79104760
H 7.89101081 -1.56357900 0.12325051
H 7.41633326 -3.05788622 0.97653882
H 5.68734047 -4.76730641 0.81827800
H 4.25590050 -5.14492558 -0.17996108
H 5.81993938 -4.75818873 -0.94887506
H 2.67798685 -3.44284601 -0.29941741
H 0.86920273 -2.56585243 -1.33455433
H -0.28353386 -1.46393700 -0.51589099
H 0.73120632 -2.57618173 0.45703164
BMK/aug-cc-pVDZ_r5
N 0.21669739 0.75594327 -0.88216886
C -0.16961098 2.06212445 -0.78870253
O -1.28074822 2.45085304 -1.14996435
N 0.73134083 3.00097885 -0.24398808
C 2.09289272 2.79049928 -0.09373844
O 2.86870002 3.71323872 0.09882099
C 2.47550541 1.36333075 -0.15628153
N 3.73353724 1.01137992 -0.16508339
C 4.04215854 -0.30305334 -0.19258404
C 5.40377592 -0.70051369 -0.12405431
C 5.78554222 -2.03338507 -0.09267423
C 7.23689650 -2.42504232 -0.01774493
C 4.75788784 -3.06052507 -0.12297751
C 5.16302313 -4.51058396 -0.08635910
C 3.42095006 -2.71069423 -0.18977863
C 3.01587750 -1.34000454 -0.24396008
N 1.72489161 -0.96676821 -0.31609949
C 1.37024844 0.37584046 -0.29407705
C 0.65658193 -1.97051694 -0.34478457
H 0.41541423 3.96502071 -0.23673465
H 6.15164869 0.09443812 -0.08275663
H 7.51909451 -3.05699295 -0.87668779
H 7.88116197 -1.53641911 -0.00822419
H 7.43738599 -3.01600920 0.89221315
H 5.74514430 -4.72683830 0.82438012
H 4.28417419 -5.16735787 -0.10930895
H 5.80994677 -4.75285732 -0.94568663
H 2.67106494 -3.49899331 -0.22308780
H 0.82071033 -2.65026960 -1.19050012
H -0.29572603 -1.45063296 -0.46474068
H 0.66282935 -2.53313781 0.59952080
BP86/cc-pVDZ_S0
N -2.57339590 -1.32077986 -0.00002269
C -3.77576735 -0.63953856 0.00006105
O -4.87323929 -1.20978602 0.00011316
N -3.74802315 0.78133841 -0.00005030
C -2.63216527 1.60418420 -0.00019463
O -2.70822633 2.83583315 -0.00016046
C -1.34891940 0.83414778 -0.00003678
N -0.22033677 1.51818040 0.00007529
C 0.95024770 0.80608224 0.00008620
C 2.18137562 1.51385140 0.00017440
C 3.40922388 0.85763526 0.00009317
C 4.70315122 1.63083145 0.00013878
C 3.41904680 -0.57975490 -0.00010367
C 4.72920635 -1.32056019 -0.00020528
C 2.21475109 -1.29675424 -0.00013018
C 0.97137245 -0.62802006 -0.00001481
N -0.23872392 -1.30973064 0.00000308
C -1.44570853 -0.62169797 -0.00002790
C -0.24965662 -2.78021730 0.00012433
H -4.66466157 1.24403124 0.00011703
H 2.12326307 2.61236392 0.00031195
H 5.32041918 1.38553477 -0.89010783
H 4.51716321 2.72135538 0.00120551
H 5.32141425 1.38390525 0.88922171
H 5.33841153 -1.05096988 0.88876682
H 4.57916784 -2.41620651 -0.00140726
H 5.33948406 -1.04908068 -0.88783661
H 2.25577804 -2.39303993 -0.00027650
H 0.26588816 -3.15628676 -0.90424697
H -1.30409086 -3.09807980 0.00024217
H 0.26589847 -3.15609704 0.90456265
BP86/cc-pVDZ_S1(r2)
N 0.10180790 0.70800980 -0.47207301
C -0.27977691 2.05480679 -0.50308843
O -1.48273888 2.35501561 -0.59893067
N 0.71128998 3.02202692 -0.42480372
C 2.10693065 2.79937199 -0.31476095
O 2.88677459 3.76429341 -0.25405188
C 2.47931349 1.38951928 -0.28438437
N 3.80660829 1.06232516 -0.17649100
C 4.07801448 -0.27834747 -0.16268346
C 5.42079807 -0.70565922 -0.05654231
C 5.79627547 -2.06642926 -0.03907411
C 7.24231994 -2.44821060 0.07722468
C 4.78506563 -3.07676889 -0.13296452
C 5.15192853 -4.53656323 -0.12037588
C 3.44306267 -2.67855697 -0.23663053
C 3.07186587 -1.31250182 -0.25155762
N 1.73564786 -0.92299204 -0.35110578
C 1.42077790 0.42209982 -0.37084294
C 0.67997646 -1.94316544 -0.43180891
H 0.40723938 4.00112484 -0.45020685
H 6.18794850 0.07978949 0.01318697
H 7.56205391 -3.06882844 -0.78825909
H 7.89825980 -1.56016229 0.13513107
H 7.41689154 -3.07628563 0.97826882
H 5.69308580 -4.81062292 0.81081405
H 4.25407093 -5.17831902 -0.19751722
H 5.83119927 -4.79099812 -0.96245715
H 2.67198283 -3.45635348 -0.30764246
H 0.84437917 -2.57293229 -1.32755330
H -0.28677396 -1.42322923 -0.50125002
H 0.71272618 -2.57840501 0.47461607
BP86/cc-pVDZ_r7
N 0.07397723 0.72243484 -0.48145894
C -0.28849498 2.05313557 -0.51605966
O -1.47983374 2.40960812 -0.61724404
N 0.72878866 3.01801169 -0.43249758
C 2.11154207 2.79559333 -0.31385043
O 2.90675695 3.74868959 -0.24556298
C 2.48146890 1.37404628 -0.28277103
N 3.79307375 1.06250960 -0.16793835
C 4.07768991 -0.29748519 -0.15572227
C 5.42345640 -0.69755599 -0.05499352
C 5.81970559 -2.06649147 -0.03945333
C 7.25616803 -2.43188140 0.08734819
C 4.79264958 -3.08587498 -0.14089491
C 5.14270188 -4.53497551 -0.13655680
C 3.43579837 -2.67729851 -0.24377861
C 3.08307621 -1.32350776 -0.24696341
N 1.70279529 -0.92389651 -0.34793994
C 1.39013594 0.41485706 -0.37514795
C 0.66806153 -1.94704621 -0.41605745
H 0.42842738 3.99872882 -0.45755207
H 6.18302218 0.09463895 0.01296226
H 7.58993636 -3.08123991 -0.75611507
H 7.90920868 -1.54213439 0.13330186
H 7.43928426 -3.05488785 0.99542260
H 5.71174248 -4.81673994 0.77979124
H 4.24535933 -5.17641833 -0.20420072
H 5.82425149 -4.79245349 -0.98106490
H 2.66859853 -3.45724773 -0.32245916
H 0.82210015 -2.58559105 -1.31141635
H -0.30494367 -1.43505167 -0.47595031
H 0.71250065 -2.59142416 0.48700910
BP86/cc-pVDZ_r8
N 0.09193004 0.74923114 -0.47877115
C -0.33799471 2.05745734 -0.50669825
O -1.52692807 2.38832559 -0.60047384
N 0.69085892 3.01660483 -0.41893599
C 2.08142791 2.79731552 -0.30861781
O 2.89144880 3.73864947 -0.23929006
C 2.43268078 1.38726310 -0.28817383
N 3.73336587 0.98554772 -0.18609942
C 4.11980498 -0.28924891 -0.16341853
C 5.47683440 -0.71732301 -0.05897778
C 5.82695627 -2.06863863 -0.03980788
C 7.27690961 -2.47357820 0.07390185
C 4.80939523 -3.06840102 -0.12708603
C 5.15892898 -4.53265335 -0.10856383
C 3.46255644 -2.65660315 -0.23112973
C 3.08340169 -1.31436958 -0.25230252
N 1.73601157 -0.91285904 -0.35582125
C 1.39630281 0.42170073 -0.37645915
C 0.67334566 -1.92153099 -0.44184244
H 0.38397225 3.99566785 -0.43908207
H 6.24429920 0.06696982 0.00623514
H 7.58935660 -3.09097676 -0.79494470
H 7.94041966 -1.59016190 0.12819882
H 7.44884374 -3.09423325 0.97895862
H 5.69779559 -4.81071571 0.82307390
H 4.25412449 -5.16534954 -0.18207771
H 5.83461807 -4.80052565 -0.94966209
H 2.67639568 -3.42529529 -0.29835323
H 0.81930470 -2.55220921 -1.34106915
H -0.29393119 -1.39100651 -0.51716044
H 0.67661938 -2.55600162 0.46663601
BP86/cc-pVDZ_T1
N 0.11037300 0.71882800 -0.47631100
C -0.27696400 2.06218000 -0.50203400
O -1.47296300 2.38110300 -0.59440900
N 0.72541700 3.02789200 -0.42014200
C 2.10738400 2.80174500 -0.31171100
O 2.90486300 3.74976900 -0.24723100
C 2.47692800 1.37826100 -0.28556700
N 3.80034000 1.06101700 -0.18070600
C 4.07533300 -0.27826200 -0.16661000
C 5.41863500 -0.70120000 -0.06045700
C 5.79419800 -2.05768700 -0.04065000
C 7.23985300 -2.43885800 0.07573900
C 4.78179700 -3.07813700 -0.13198000
C 5.15908700 -4.53449400 -0.11532900
C 3.44160000 -2.68921400 -0.23589700
C 3.06179400 -1.32487900 -0.25457300
N 1.73363700 -0.94833100 -0.35400100
C 1.40956000 0.43302000 -0.37382100
C 0.67386800 -1.95717400 -0.43563400
H 0.42314100 4.00849700 -0.44164000
H 6.18383400 0.08574700 0.00776100
H 7.55810400 -3.05983200 -0.78969200
H 7.89502600 -1.55039700 0.13286900
H 7.41307800 -3.06621800 0.97716600
H 5.70202600 -4.80163200 0.81671000
H 4.26554400 -5.18216700 -0.19108600
H 5.84073100 -4.78578700 -0.95627400
H 2.67246200 -3.46893000 -0.30459000
H 0.82700900 -2.59121800 -1.33112500
H -0.28785300 -1.42652500 -0.50742500
H 0.69115400 -2.59406200 0.47083800
BP86/cc-pVDZ_T6
N 0.08396900 0.72056600 -0.48076800
C -0.26826200 2.05300700 -0.50262600
O -1.44656100 2.43457500 -0.59651700
N 0.75445800 3.03772800 -0.41361800
C 2.12690800 2.81026300 -0.30414800
O 2.92925600 3.75363100 -0.23307800
C 2.49091000 1.37555400 -0.28455300
N 3.76792400 1.05276000 -0.18561200
C 4.07553000 -0.29068400 -0.16890300
C 5.41062600 -0.68749500 -0.06584000
C 5.80727500 -2.04450200 -0.04189200
C 7.24899100 -2.42459100 0.07406800
C 4.75951700 -3.08983600 -0.13175100
C 5.16182900 -4.53684400 -0.11090400
C 3.43170200 -2.72488100 -0.23338200
C 3.03066500 -1.34686100 -0.25643600
N 1.72291100 -0.95271500 -0.35647900
C 1.38016800 0.39249100 -0.37839200
C 0.65290100 -1.96349800 -0.44177300
H 0.44468300 4.01639000 -0.43252300
H 6.16648400 0.11015400 -0.00171700
H 7.58216100 -3.04866900 -0.78622200
H 7.89977000 -1.53126000 0.12414300
H 7.44041200 -3.04353800 0.98039300
H 5.71589900 -4.78947900 0.81941300
H 4.28067900 -5.20228000 -0.18102800
H 5.84797600 -4.77804400 -0.95205900
H 2.67128900 -3.51431700 -0.29856100
H 0.81302700 -2.59384600 -1.33645000
H -0.30456300 -1.42593500 -0.51703400
H 0.67046200 -2.59479200 0.46643200
BP86/cc-pVDZ_T7
N 0.07484000 0.72171600 -0.47446100
C -0.28646800 2.05254800 -0.50097900
O -1.48306900 2.40643700 -0.59163200
N 0.72603600 3.01958400 -0.42247400
C 2.10910400 2.79897700 -0.31449300
O 2.90423000 3.75040000 -0.25072000
C 2.47109700 1.37403600 -0.28768500
N 3.78464300 1.04925200 -0.18429200
C 4.08030800 -0.28467200 -0.16727200
C 5.42666700 -0.68796100 -0.06116300
C 5.81719000 -2.06672700 -0.03907200
C 7.25556800 -2.44219700 0.08318300
C 4.77875000 -3.08702700 -0.13477300
C 5.14422400 -4.53604500 -0.12188400
C 3.43198800 -2.69754100 -0.23916400
C 3.06403500 -1.32320900 -0.25471100
N 1.72638500 -0.93001400 -0.35363600
C 1.39992800 0.42523400 -0.37387700
C 0.67220000 -1.94556600 -0.43277500
H 0.42215700 3.99953700 -0.44490200
H 6.18816500 0.10193400 0.00558600
H 7.59066700 -3.07323100 -0.77232700
H 7.90725000 -1.55076800 0.13543600
H 7.44154400 -3.06574400 0.98858700
H 5.70231900 -4.80343000 0.80388400
H 4.25338500 -5.18615800 -0.19595200
H 5.83489900 -4.78507200 -0.95931800
H 2.66695200 -3.47990100 -0.31048100
H 0.83261400 -2.57808000 -1.32810700
H -0.29220700 -1.41960900 -0.50076300
H 0.70359900 -2.58365000 0.47242200
BPBE/cc-pVDZ_S0
N -2.57173258 -1.31805096 -0.00002146
C -3.77278980 -0.63753033 0.00009992
O -4.86938620 -1.20752580 -0.00001908
N -3.74462280 0.78149302 0.00004837
C -2.62954816 1.60281521 -0.00007088
O -2.70494289 2.83348723 -0.00021050
C -1.34731397 0.83275453 -0.00002912
N -0.21963408 1.51546579 0.00005081
C 0.94953699 0.80381433 0.00007427
C 2.17960401 1.51112911 0.00015757
C 3.40744925 0.85717953 0.00008104
C 4.69811816 1.63372557 0.00011746
C 3.41760073 -0.57919313 -0.00010002
C 4.72536063 -1.32223018 -0.00019440
C 2.21381360 -1.29512769 -0.00011685
C 0.97053651 -0.62870887 -0.00001356
N -0.23875384 -1.30967319 0.00000557
C -1.44437903 -0.62139457 -0.00003987
C -0.25153665 -2.77841574 0.00014545
H -4.65931550 1.24397468 0.00013007
H 2.12095350 2.60785799 0.00028348
H 5.31496056 1.39160511 -0.88885898
H 4.50941589 2.72190944 0.00120218
H 5.31599304 1.38995475 0.88790257
H 5.33398972 -1.05524521 0.88750799
H 4.57374941 -2.41579546 -0.00139283
H 5.33507501 -1.05336731 -0.88655525
H 2.25579238 -2.38957069 -0.00025235
H 0.26143539 -3.15563155 -0.90300079
H -1.30444192 -3.09550433 0.00029867
H 0.26151386 -3.15542792 0.90332277
BPBE/cc-pVDZ_S1(r1)
N -2.54883085 -1.31125803 -0.00005435
C -3.77985966 -0.64728319 -0.00006216
O -4.83607151 -1.30176747 0.00015938
N -3.77887127 0.73819188 0.00003635
C -2.64387144 1.58460923 0.00014335
O -2.79045336 2.81707825 0.00019083
C -1.37352251 0.86908663 0.00014618
N -0.21107339 1.59369679 0.00017866
C 0.93771640 0.85280293 0.00013636
C 2.18260915 1.51901988 0.00011521
C 3.41987444 0.84096920 0.00005418
C 4.70212941 1.61766984 0.00000306
C 3.43507279 -0.59013704 0.00000710
C 4.73350044 -1.34980644 0.00000708
C 2.21090788 -1.27509366 -0.00002228
C 0.97335148 -0.59031600 0.00004095
N -0.23921972 -1.27788146 0.00001117
C -1.42227761 -0.56561461 0.00006573
C -0.24942925 -2.74600472 -0.00014476
H -4.69091697 1.20325520 0.00009499
H 2.15776133 2.61710613 0.00015797
H 5.32197089 1.36357031 -0.88520812
H 4.52229167 2.70661307 0.00050723
H 5.32257923 1.36279428 0.88454913
H 5.34904820 -1.09959884 0.88842973
H 4.56178646 -2.44088565 -0.00048926
H 5.34952547 -1.09883790 -0.88785818
H 2.22846876 -2.37061329 -0.00008309
H 0.26964102 -3.11812491 -0.90257178
H -1.29697746 -3.07612201 -0.00017803
H 0.26964408 -3.11834551 0.90218964
BPBE/cc-pVDZ_r7
N 0.07873563 0.72038417 -0.48144663
C -0.28393212 2.04914404 -0.51798615
O -1.47426780 2.40479292 -0.62061740
N 0.73194155 3.01282456 -0.43453607
C 2.11266660 2.79073378 -0.31415462
O 2.90723383 3.74295835 -0.24531330

C 2.48222369 1.37067868 -0.28219976
N 3.79198985 1.05919038 -0.16588923
C 4.07640670 -0.29892087 -0.15410211
C 5.42090973 -0.69847202 -0.05455965
C 5.81741775 -2.06561818 -0.03984626
C 7.25241865 -2.42886321 0.08824413
C 4.79165027 -3.08387839 -0.14229404
C 5.13973238 -4.53160386 -0.13942905
C 3.43646521 -2.67545220 -0.24470746
C 3.08310048 -1.32351724 -0.24589906
N 1.70508939 -0.92442219 -0.34623021
C 1.39276438 0.41277316 -0.37471091
C 0.67114175 -1.94526254 -0.41243566
H 0.43229000 3.99169555 -0.46019086
H 6.17909944 0.09237357 0.01302820
H 7.58648184 -3.07727489 -0.75302983
H 7.90352844 -1.54017359 0.13413320
H 7.43462004 -3.04971194 0.99539916
H 5.70805379 -4.81383372 0.77462254
H 4.24341906 -5.17106400 -0.20682352
H 5.81885832 -4.78794854 -0.98345708
H 2.67120942 -3.45431308 -0.32493935
H 0.82164063 -2.58194810 -1.30726840
H -0.30022245 -1.43376051 -0.47010988
H 0.71633089 -2.58845832 0.48893571

BPBE/cc-pVDZ_r8

N -2.58648940 -1.28941781 -0.00011036
C -3.82289962 -0.68578868 -0.00034689
O -4.89122470 -1.30879680 0.00010556
N -3.78875437 0.72127436 -0.00005578
C -2.65901546 1.56572262 -0.00003151
O -2.76545911 2.80404945 0.00038217
C -1.40468352 0.83387137 0.00004737
N -0.20758835 1.48665487 0.00009958
C 0.97405638 0.87449570 0.00011329
C 2.22933318 1.55020249 0.00020305
C 3.44213066 0.86108807 0.00009717
C 4.74356722 1.62431242 0.00002723
C 3.44618121 -0.56713379 0.00000067
C 4.73656614 -1.34077313 -0.00012300
C 2.20904366 -1.24624850 0.00005453
C 0.98265416 -0.58411271 0.00006250
N -0.24718699 -1.27125736 0.00003666
C -1.43926265 -0.58356767 0.00001638
C -0.26979319 -2.73717581 -0.00000701
H -4.70256881 1.18423679 0.00017589
H 2.20364205 2.64738264 0.00028108
H 5.35589120 1.37032263 -0.88879531
H 4.57203933 2.71526313 0.00054316
H 5.35648605 1.36953924 0.88820389
H 5.35618442 -1.09834760 0.88794060
H 4.55457186 -2.43038655 -0.00078805
H 5.35666126 -1.09730699 -0.88755258
H 2.21064545 -2.34585648 0.00002496
H 0.23329795 -3.12571337 -0.90543049
H -1.32479668 -3.06199747 -0.00002948
H 0.23327477 -3.12576217 0.90540699

BPBE/cc-pVDZ_T1

N 0.11491500 0.71668600 -0.47615600
C -0.27269600 2.05808400 -0.50201200
O -1.46791500 2.37591400 -0.59396200
N 0.72821500 3.02258300 -0.42089800
C 2.10850300 2.79689700 -0.31307800
O 2.90514400 3.74423700 -0.24914300
C 2.47796100 1.37499100 -0.28586400
N 3.79960600 1.05785500 -0.18101600
C 4.07435300 -0.27987700 -0.16692000
C 5.41628400 -0.70212200 -0.06069100
C 5.79213600 -2.05687200 -0.04117400
C 7.23653500 -2.43583800 0.07537000
C 4.78084600 -3.07623200 -0.13272900
C 5.15583200 -4.53132200 -0.11677100
C 3.44223200 -2.68734200 -0.23636100
C 3.06231900 -1.32490700 -0.25464900
N 1.73572800 -0.94860000 -0.35300400
C 1.41226200 0.43080500 -0.37343300
C 0.67697400 -1.95521300 -0.43246800
H 0.42652400 4.00130900 -0.44270000
H 6.17985500 0.08368000 0.00759600
H 7.55416600 -3.05735600 -0.78711900
H 7.88979200 -1.54828200 0.12996600
H 7.40984700 -3.05891300 0.97718600
H 5.69885100 -4.79827600 0.81274400
H 4.26299200 -5.17674600 -0.19079400
H 5.83395700 -4.78256300 -0.95782700
H 2.67486800 -3.46594300 -0.30508400
H 0.82713600 -2.58790700 -1.32697400
H -0.28319100 -1.42526300 -0.50258500
H 0.69495800 -2.59041300 0.47273700

BPBE/cc-pVDZ_T6

N 0.08842300 0.71868900 -0.47999900
C -0.26395500 2.04921000 -0.50204800
O -1.44145400 2.42998500 -0.59578400
N 0.75731500 3.03251900 -0.41352900
C 2.12786200 2.80523200 -0.30462000
O 2.92986300 3.74753500 -0.23402000
C 2.49151500 1.37198200 -0.28482600
N 3.76700000 1.04938000 -0.18641800
C 4.07436100 -0.29228500 -0.16947900
C 5.40825900 -0.68840900 -0.06700500
C 5.80528500 -2.04363200 -0.04274500
C 7.24584100 -2.42132600 0.07244700
C 4.75871000 -3.08791900 -0.13152700
C 5.15860000 -4.53371000 -0.11045200
C 3.43226700 -2.72281500 -0.23258100
C 3.03112200 -1.34701600 -0.25606100
N 1.72480500 -0.95313000 -0.35575600
C 1.38265800 0.39042000 -0.37792200
C 0.65611400 -1.96163000 -0.44035300
H 0.44840000 4.00938600 -0.43261300
H 6.16260900 0.10802400 -0.00359600
H 7.57819600 -3.04382100 -0.78655600
H 7.89449400 -1.52888800 0.12176700
H 7.43758900 -3.03824000 0.97747600
H 5.71164200 -4.78596500 0.81808000
H 4.27818100 -5.19689000 -0.17956200
H 5.84217300 -4.77533400 -0.95083200
H 2.67355400 -3.51112900 -0.29685800
H 0.81455000 -2.59081200 -1.33376000
H -0.29981300 -1.42496500 -0.51526000
H 0.67282200 -2.59139000 0.46657800

BPBE/cc-pVDZ_T7

N 0.07944500 0.71927400 -0.47302500
C -0.28205800 2.04817400 -0.50085800
O -1.47778000 2.40140600 -0.59028100
N 0.72904100 3.01422900 -0.42302200
C 2.11022000 2.79427300 -0.31473300
O 2.90477000 3.74484500 -0.25266000
C 2.47214400 1.37073800 -0.28795100
N 3.78367600 1.04618600 -0.18522300
C 4.07946900 -0.28614900 -0.16787500
C 5.42399200 -0.68869800 -0.06231000
C 5.81513100 -2.06580500 -0.03991400
C 7.25221400 -2.43923900 0.08259400
C 4.77749900 -3.08520700 -0.13513400
C 5.14104100 -4.53280400 -0.12261800
C 3.43258700 -2.69589700 -0.23893600
C 3.06401700 -1.32327700 -0.25447500
N 1.72891800 -0.93064600 -0.35252100
C 1.40247800 0.42295000 -0.37307600
C 0.67529500 -1.94364900 -0.43064000
H 0.42564700 3.99225500 -0.44589600
H 6.18412300 0.09990200 0.00383400
H 7.58664600 -3.07043300 -0.77020100
H 7.90197200 -1.54873600 0.13260600
H 7.43810100 -3.05875700 0.98816400
H 5.69894200 -4.79961300 0.80086300
H 4.25124000 -5.18107800 -0.19538000
H 5.82886000 -4.78142800 -0.95972100
H 2.66931900 -3.47720800 -0.30993900
H 0.83319200 -2.57456700 -1.32504800
H -0.28720300 -1.41782000 -0.49763600
H 0.70604800 -2.58016700 0.47319900

CAM-B3LYP/cc-pVDZ_S0

N -2.55226089 -1.30418078 -0.00005670
C -3.74367191 -0.62669707 0.00008268
O -4.82516084 -1.18845234 -0.00000589
N -3.72218846 0.77839657 0.00028599
C -2.61715698 1.58691517 -0.00002048
O -2.68540530 2.80018893 -0.00025750
C -1.33669823 0.82423330 -0.00000285
N -0.22769730 1.49099910 0.00004930
C 0.94427840 0.79163304 0.00005248
C 2.16159665 1.50062205 0.00008208
C 3.37711558 0.85418242 0.00004818
C 4.66507693 1.62700419 -0.00005089
C 3.38807448 -0.56970795 -0.00001495
C 4.69557473 -1.30533689 0.00008216
C 2.19922281 -1.28125205 -0.00005661
C 0.96240440 -0.61885603 -0.00000014
N -0.24134357 -1.30101954 -0.00003999
C -1.43026096 -0.62790737 -0.00003575
C -0.24116813 -2.76333997 -0.00010670
H -4.63092799 1.23430518 0.00027088
H 2.10084968 2.58960369 0.00014087
H 5.27534585 1.38733853 -0.88449400
H 4.47490632 2.70771862 0.00136319
H 5.27687225 1.38522750 0.88273221
H 5.29602898 -1.03911203 0.88363919
H 4.54497077 -2.39184193 -0.00132885
H 5.29755481 -1.03694000 -0.88174487
H 2.24143059 -2.36770123 -0.00015956
H 0.27271296 -3.13278816 -0.89667159
H -1.28016200 -3.09521308 0.00008474
H 0.27305192 -3.13281445 0.89624936

CAM-B3LYP/cc-pVDZ_S1(r1)

N -2.52433980 -1.28333300 -0.00005000
C -3.75289709 -0.63211668 0.00015063
O -4.79907015 -1.26001028 -0.00034801
N -3.74710572 0.74686132 0.00017903
C -2.62789278 1.56938164 0.00025092
O -2.74623499 2.78694734 0.00025940
C -1.35897512 0.85048323 0.00017289
N -0.21571136 1.56493040 0.00018901
C 0.92852883 0.84560091 0.00010946
C 2.16014179 1.51374272 0.00009560
C 3.37877237 0.84495607 0.00003845
C 4.66043276 1.61325533 -0.00007706
C 3.40013787 -0.58591715 0.00001648
C 4.70345094 -1.32492633 0.00009002
C 2.20069924 -1.27475632 -0.00001573
C 0.96186154 -0.60483548 0.00001598
N -0.22295111 -1.28575310 -0.00006218
C -1.42107117 -0.56923510 0.00001268
C -0.23447539 -2.74406422 -0.00023125
H -4.65292116 1.20787532 0.00010836
H 2.12842539 2.60338444 0.00013988
H 5.27019396 1.35964907 -0.88201881
H 4.48023243 2.69486524 0.00057591
H 5.27105246 1.35866525 0.88096723
H 5.30941235 -1.06778401 0.88355117
H 4.54317520 -2.41031743 -0.00057304
H 5.31015002 -1.06677457 -0.88255333
H 2.22385908 -2.36198076 -0.00006202
H 0.27971114 -3.11919407 -0.89593451
H -1.27331173 -3.07498892 -0.00028462
H 0.27968327 -3.11940417 0.89539945

CAM-B3LYP/cc-pVDZ_r5

N -2.56926480 -1.30531201 -0.08402833
C -3.76294970 -0.64944909 -0.05475760
O -4.84975662 -1.21965307 -0.08784793
N -3.73942844 0.74650549 0.01828035
C -2.62313643 1.55658944 0.06225985
O -2.72018786 2.77805713 0.12880892
C -1.36694035 0.81764671 0.02135608
N -0.20608785 1.53769462 0.05558686
C 0.91367245 0.81039677 0.02909319
C 2.18260386 1.49220115 0.06438035
C 3.42658345 0.84574840 0.04512974
C 4.69814222 1.63571103 0.09046198
C 3.43596661 -0.54473426 -0.01556023
C 4.70742113 -1.32058206 -0.04404694
C 2.18220411 -1.24479903 -0.05735158
C 0.94101091 -0.59789525 -0.03048809
N -0.24974053 -1.29815609 -0.06699541
C -1.45433883 -0.59560254 -0.04686298
C -0.25029998 -2.75165500 -0.12663662
H -4.64278438 1.21035352 0.04144057
H 2.13266218 2.58167546 0.11348520
H 5.32692891 1.41800614 -0.78638762
H 4.49835276 2.71350074 0.11550509
H 5.29025065 1.37139932 0.98050357
H 5.33589481 -1.07573762 0.82786410
H 4.52757537 -2.40183337 -0.05432785
H 5.30396510 -1.05684114 -0.93395784
H 2.22346104 -2.33057604 -0.11698726
H 0.26127578 -3.10147028 -1.03498307
H -1.29254034 -3.07499592 -0.14429575
H 0.25263434 -3.17079778 0.75718376

HCTH/cc-pVDZ_S0

N -2.56013326 -1.30380241 -0.00029766
C -3.75213392 -0.62886858 -0.00022429
O -4.84077445 -1.19528421 -0.00052098
N -3.72347110 0.77868896 0.00025491
C -2.61578621 1.59224240 0.00006107
O -2.69019462 2.81389952 0.00009011
C -1.33918157 0.82554348 0.00002131
N -0.21797499 1.49977510 0.00007263
C 0.94316723 0.79448393 0.00003865
C 2.16602280 1.49861187 0.00002757
C 3.39020702 0.85479299 -0.00002555
C 4.66698264 1.63742418 -0.00002567
C 3.40264578 -0.57376781 -0.00012536
C 4.70042444 -1.31750770 -0.00031449
C 2.20522191 -1.28544105 -0.00001137
C 0.96461271 -0.62981036 0.00005479
N -0.23855241 -1.30706645 0.00019870
C -1.43650288 -0.62071386 -0.00003735
C -0.25100152 -2.76544665 0.00061689
H -4.62862953 1.23928890 0.00040983
H 2.10426891 2.58808884 0.00008060
H 5.28361804 1.40285118 -0.88197917
H 4.47234468 2.71715460 0.00072307
H 5.28435106 1.40172122 0.88109750
H 5.30799564 -1.05529347 0.88026310
H 4.54796087 -2.40357721 -0.00104760
H 5.30849798 -1.05412643 -0.88017918
H 2.25313243 -2.37165977 -0.00003232
H 0.25976908 -3.14262747 -0.89518598
H -1.29250708 -3.09111913 0.00072654
H 0.25980217 -3.14204722 0.89663335

HCTH/cc-pVDZ_S1(r2)

N 0.12794290 0.70610782 -0.47452456
C -0.25175622 2.04108792 -0.49718041
O -1.44420074 2.34165185 -0.58975214
N 0.73258958 2.99793657 -0.41430524
C 2.11463144 2.77444421 -0.30664904
O 2.88942628 3.73019666 -0.23995601
C 2.48474342 1.37113150 -0.28495825
N 3.79760908 1.04143246 -0.18310561
C 4.06803861 -0.28786904 -0.16891094
C 5.40230031 -0.71131447 -0.06583650
C 5.78408730 -2.06193463 -0.04293464
C 7.22498663 -2.42564778 0.06886023
C 4.77990461 -3.06748664 -0.12764746
C 5.13416624 -4.52042476 -0.10745346
C 3.44668797 -2.67036450 -0.23078288
C 3.06820739 -1.31467024 -0.25336715
N 1.74171174 -0.92715852 -0.35601046
C 1.43031574 0.41243547 -0.37335185
C 0.69265161 -1.93542205 -0.44426618
H 0.43398480 3.96729431 -0.43252620
H 6.16076091 0.07055749 -0.00174655
H 7.54835658 -3.03728965 -0.78976506
H 7.86816205 -1.53945690 0.12463752
H 7.41009399 -3.04715192 0.96069178
H 5.66759131 -4.79373660 0.81737154
H 4.24080971 -5.15308508 -0.18159911
H 5.80672498 -4.78425505 -0.93967312
H 2.68728246 -3.44647365 -0.29491117
H 0.85543263 -2.56055748 -1.33279726
H -0.26877149 -1.42676530 -0.51742996
H 0.71452484 -2.57015837 0.45206594

HCTH/cc-pVDZ_r7

N 0.09835337 0.72092994 -0.47695023
C -0.26076811 2.03993476 -0.49806116
O -1.44336929 2.39460824 -0.58974172
N 0.74882080 2.99450035 -0.41307838
C 2.11929946 2.77018833 -0.30621158
O 2.91040201 3.71347004 -0.23827217
C 2.48535682 1.35541007 -0.28487367
N 3.78498735 1.04002420 -0.18388581
C 4.06793929 -0.30626256 -0.16886544
C 5.40577965 -0.70344792 -0.06543980
C 5.80676086 -2.06066773 -0.04115298
C 7.23854680 -2.41119957 0.06982597
C 4.78616690 -3.07567720 -0.12719342
C 5.12592709 -4.51750513 -0.10800508
C 3.43823402 -2.66885708 -0.23143980
C 3.07926025 -1.32586427 -0.25248212
N 1.70875780 -0.92670940 -0.35855314
C 1.40103761 0.40467573 -0.37574910
C 0.68073948 -1.93815425 -0.44512674
H 0.45421444 3.96542477 -0.43101805
H 6.15659771 0.08516299 -0.00193167
H 7.57189841 -3.03487374 -0.78112630
H 7.87986565 -1.52465279 0.12575330
H 7.43487081 -3.04540898 0.95492344
H 5.67499138 -4.79471171 0.81082204
H 4.23535574 -5.15210626 -0.18225830
H 5.81365746 -4.78530203 -0.93130643
H 2.68282059 -3.44799065 -0.29531085
H 0.84464348 -2.57196280 -1.33090195
H -0.28643670 -1.43833558 -0.51872696
H 0.70428557 -2.58158611 0.44852389

HCTH/cc-pVDZ_r8

N 0.10092616 0.76343941 -0.47755019
C -0.31398523 2.06474707 -0.50423498
O -1.49054968 2.40327655 -0.59693211
N 0.71442748 3.00439559 -0.41653861
C 2.08962699 2.77108103 -0.30788008
O 2.90365603 3.69581721 -0.23773124
C 2.42633759 1.36615892 -0.28870029
N 3.71201063 0.95639696 -0.18850652
C 4.08570892 -0.31016423 -0.16670623
C 5.43230646 -0.74054250 -0.06350810
C 5.78277272 -2.08177294 -0.04423276
C 7.22543045 -2.47631533 0.06788353
C 4.76732847 -3.07139027 -0.13044797
C 5.09854194 -4.52995884 -0.11364963

C 3.43096870 -2.65504177 -0.23305114
C 3.05078868 -1.32179386 -0.25472264
N 1.71356533 -0.91332596 -0.35727880
C 1.38806202 0.41620779 -0.37658902
C 0.65360064 -1.90818911 -0.44259446
H 0.42220499 3.97627291 -0.43505418
H 6.19219907 0.03894114 0.00066295
H 7.54040463 -3.08713962 -0.79276582
H 7.88053204 -1.59795700 0.12095809
H 7.40228699 -3.09017608 0.96519435
H 5.63128520 -4.81477342 0.80836691
H 4.19588973 -5.14980163 -0.18563584
H 5.76426136 -4.80464820 -0.94827614
H 2.65548231 -3.41997951 -0.29879705
H 0.79490657 -2.53784177 -1.33231898
H -0.30771164 -1.38986687 -0.51886915
H 0.65219732 -2.53931986 0.45741290

HCTH/cc-pVDZ_T1

N -2.54056200 -1.28458700 0.00009200
C -3.76221500 -0.63123500 0.00018200
O -4.82013800 -1.26149400 -0.00002100
N -3.75091900 0.75122400 0.00013700
C -2.63054300 1.57613600 0.00005000
O -2.74790700 2.80120600 0.00006200
C -1.35918900 0.85095700 0.00013100
N -0.21253700 1.56952500 0.00013300
C 0.92902400 0.84107200 0.00008700
C 2.16431200 1.50875600 0.00005600
C 3.39407500 0.84459400 -0.00001500
C 4.66306100 1.62608400 -0.00009400
C 3.41817600 -0.58786700 -0.00005200
C 4.71190600 -1.33667800 -0.00010500
C 2.20902600 -1.27481600 -0.00000700
C 0.96929900 -0.60913800 0.00006500
N -0.22279700 -1.29529100 0.00013900
C -1.43007200 -0.56626700 0.00012300
C -0.24406700 -2.74851600 0.00023800
H -4.65401500 1.21492600 0.00008000
H 2.13168800 2.59904100 0.00008800
H 5.28076600 1.37811800 -0.87872300
H 4.47752100 2.70653400 0.00027100
H 5.28128300 1.37757000 0.87800500
H 5.32398200 -1.08566300 0.88115000
H 4.54773000 -2.42141400 -0.00046300
H 5.32427300 -1.08510500 -0.88098900
H 2.23723500 -2.36201400 -0.00003800
H 0.26525600 -3.13201700 -0.89503700
H -1.28486600 -3.07535100 0.00033100
H 0.26539700 -3.13188400 0.89548700

HCTH/cc-pVDZ_T6

N -2.55964000 -1.30121800 -0.00041200
C -3.75045600 -0.63273900 -0.00015000
O -4.84067400 -1.20633500 -0.00043800
N -3.73762200 0.77802200 0.00051100
C -2.62216300 1.59522800 0.00056300
O -2.73420700 2.82112400 0.00094000
C -1.34739800 0.85813400 0.00028800
N -0.23021000 1.54149700 0.00015800
C 0.93810700 0.83240600 0.00000900
C 2.14797400 1.51189400 -0.00015500
C 3.39445200 0.86187500 -0.00021100
C 4.65920300 1.64158900 -0.00007900
C 3.41160500 -0.61130800 0.00001600
C 4.71599000 -1.33671600 -0.00010700
C 2.22622600 -1.30461300 0.00018100
C 0.96349500 -0.64404800 0.00009500
N -0.22855800 -1.30529200 0.00001700
C -1.42291900 -0.61445500 0.00000300
C -0.25324000 -2.76686400 -0.00004600
H -4.64399100 1.23656500 0.00063900
H 2.10162900 2.60271200 -0.00019700
H 5.28780800 1.40607700 -0.87719700
H 4.46668000 2.72181700 -0.00161100
H 5.28600100 1.40843500 0.87902700
H 5.32603300 -1.07118200 0.87920100
H 4.57227600 -2.42451300 0.00109700
H 5.32466000 -1.07305400 -0.88096800
H 2.26580900 -2.39211900 0.00036600
H 0.25649700 -3.14402800 -0.89580500
H -1.29548100 -3.08840200 -0.00017700
H 0.25629800 -3.14408600 0.89580300

HCTH/cc-pVDZ_T8

N -2.56509500 -1.30690200 0.00795500
C -3.76138700 -0.64514500 -0.00534500
O -4.84350800 -1.25191700 -0.01048300
N -3.74470000 0.74529700 -0.01215800
C -2.62831700 1.57573700 0.00240900
O -2.75016800 2.80077500 0.00378800
C -1.36008900 0.84430900 0.01259200
N -0.21783500 1.54965500 0.02587700
C 0.93705500 0.84040600 0.02028300
C 2.15771200 1.52295100 0.03574100
C 3.41679400 0.85358600 0.02605700
C 4.67712200 1.63264000 0.07193000
C 3.42275800 -0.59679500 -0.01567100
C 4.70418900 -1.34690500 -0.04944200
C 2.20957900 -1.28756800 -0.02804400
C 0.96854000 -0.60561400 -0.00391000
N -0.23921800 -1.28650300 -0.00369100
C -1.43146800 -0.57839900 0.00527900
C -0.25336300 -2.74094000 -0.01362400
H -4.64868600 1.20693500 -0.02036800
H 2.12114700 2.61241300 0.05526600
H 5.34143900 1.39158400 -0.77688100
H 4.48770200 2.71259700 0.06657500
H 5.27053400 1.39360200 0.97391900
H 5.34295300 -1.09353700 0.81429000
H 4.54525000 -2.43151600 -0.05834300
H 5.30238300 -1.07585000 -0.93743100
H 2.24157400 -2.37323800 -0.06406200
H 0.24387400 -3.11115000 -0.92121400
H -1.29283900 -3.07092800 0.00269000
H 0.28025200 -3.12317300 0.86738000

HISSbPBE/cc-pVDZ_S0

N 0.12990073 0.70572656 -0.48159642
C -0.21190769 2.02286343 -0.50395637
O -1.36023815 2.40604806 -0.60067427
N 0.79924053 2.97933366 -0.41092180
C 2.13697858 2.75208410 -0.30020827
O 2.95259982 3.64283943 -0.22434412
C 2.48106846 1.31255202 -0.28307535
N 3.72480562 0.98167479 -0.18469990
C 4.03924003 -0.33396820 -0.16841116
C 5.38635504 -0.71273288 -0.06499845
C 5.76758899 -2.03070998 -0.04398994
C 7.20511396 -2.41534067 0.06685287
C 4.75972199 -3.02652822 -0.12985405
C 5.13908493 -4.46724735 -0.11051621
C 3.42855512 -2.67003577 -0.23177021
C 3.04446699 -1.32844992 -0.25322407
N 1.73129012 -0.94219829 -0.35480298
C 1.38704290 0.37287915 -0.37833372
C 0.69359198 -1.95348621 -0.43850833
H 0.49602408 3.94341874 -0.42970241
H 6.11962193 0.08520977 -0.00227612
H 7.52318706 -3.01702376 -0.79134126
H 7.84361531 -1.53159769 0.11817764
H 7.38667388 -3.02016393 0.96189735
H 5.68023309 -4.71542943 0.80896594
H 4.26267490 -5.11328235 -0.18022928
H 5.81113128 -4.70428998 -0.94249279
H 2.68424033 -3.45369505 -0.29616285
H 0.85213668 -2.57275737 -1.32369027
H -0.26188429 -1.44237253 -0.50922490
H 0.71684247 -2.58026649 0.45529751

HISSbPBE/cc-pVDZ_S1(r1)

N 0.16346452 0.69841297 -0.47405098
C -0.22227027 2.02171666 -0.50218982
O -1.39489087 2.33084632 -0.59689911
N 0.75770280 2.97576920 -0.41958362
C 2.11656282 2.75252216 -0.30930060
O 2.89730147 3.68643931 -0.24348018
C 2.48799886 1.35197278 -0.28258529
N 3.78388672 1.02897106 -0.17775461
C 4.06614070 -0.28458477 -0.16390275
C 5.39050209 -0.70173336 -0.05971211
C 5.76076276 -2.04435142 -0.04241070
C 7.19038828 -2.42071005 0.07121441
C 4.75994243 -3.05062368 -0.13389390
C 5.13191913 -4.49219323 -0.11940203
C 3.43888216 -2.66471816 -0.23587842
C 3.06072656 -1.31375223 -0.25264828
N 1.75604864 -0.94018798 -0.35079398
C 1.43557810 0.40675736 -0.37163801
C 0.71395935 -1.94212199 -0.43084049
H 0.45659712 3.93987571 -0.44219599
H 6.14732685 0.07276059 0.00858994
H 7.50148042 -3.03220427 -0.78428369
H 7.83503137 -1.54284361 0.12612290
H 7.36025523 -3.03731188 0.96222228
H 5.66670468 -4.75641379 0.80013960
H 4.24773470 -5.12771243 -0.19272319
H 5.79974815 -4.73963567 -0.95265495
H 2.67807486 -3.43251671 -0.30511616
H 0.86696102 -2.56653125 -1.31491496
H -0.24143026 -1.43173686 -0.50027524
H 0.73590628 -2.57110654 0.46302577

HISSbPBE/cc-pVDZ_r6

N 0.10975551 0.71542425 -0.37403049
C -0.21502397 1.99262100 -0.39863837
O -1.35980439 2.45142167 -0.20603034
N 0.76808424 2.94665557 -0.62583572
C 2.10228639 2.70108001 -0.37795900
O 2.85353775 3.67590033 -0.25795690
C 2.47252940 1.32175908 -0.30084699
N 3.77272914 1.00898758 -0.17736525
C 4.06086192 -0.30136750 -0.15846260
C 5.40052857 -0.71540722 -0.03622622
C 5.77157433 -2.04344374 -0.02415362
C 7.20767608 -2.43078758 0.10731018
C 4.77198502 -3.03217331 -0.13809317
C 5.13262761 -4.47950733 -0.13241880
C 3.44058159 -2.64716347 -0.25538698
C 3.06683548 -1.31040274 -0.26746097
N 1.73660674 -0.92327772 -0.37711181
C 1.41102937 0.39775125 -0.36575307
C 0.70999631 -1.93594180 -0.46767169
H 0.47565758 3.91856535 -0.66590729
H 6.14719312 0.06847148 0.04782886
H 7.54119014 -3.02128534 -0.75343132
H 7.84816169 -1.54987559 0.18361889
H 7.37334465 -3.05081861 0.99548990
H 5.65005062 -4.75834049 0.79291181
H 4.24657701 -5.11029031 -0.22796704
H 5.81424575 -4.72418938 -0.95537508
H 2.68794431 -3.42179583 -0.34106650
H 0.86780106 -2.55009868 -1.35844516
H -0.25435553 -1.44059057 -0.52804570
H 0.73678912 -2.57882674 0.41664825

HISSbPBE/cc-pVDZ_r7

N 0.11011583 0.71091873 -0.47641540
C -0.22500019 2.01025612 -0.50367454
O -1.40827901 2.36897675 -0.60010744
N 0.75723273 2.96706768 -0.42280655
C 2.11440129 2.74094210 -0.31072813
O 2.89512199 3.67684181 -0.24494661
C 2.48294567 1.34013312 -0.28273445
N 3.77804033 1.01867905 -0.17660784
C 4.07150932 -0.29514714 -0.16153409
C 5.40403982 -0.70055780 -0.05777732
C 5.78210019 -2.04793764 -0.04060604
C 7.20610188 -2.42191151 0.07531768
C 4.76956517 -3.05312531 -0.13487123
C 5.12237558 -4.49044834 -0.12430164
C 3.43442162 -2.65077187 -0.23746499
C 3.08570292 -1.31710618 -0.24941395
N 1.72387480 -0.91304655 -0.35176739
C 1.42540096 0.39535142 -0.37205235
C 0.70860135 -1.93005103 -0.42803245
H 0.45665329 3.93070297 -0.44686541
H 6.15767898 0.07694821 0.00989114
H 7.52201255 -3.04402358 -0.77336703
H 7.85208580 -1.54528691 0.12739174
H 7.37936488 -3.04056167 0.96699578
H 5.66700626 -4.76250236 0.78984970
H 4.23486895 -5.12053636 -0.19591735
H 5.79341543 -4.74351153 -0.95640696
H 2.67561485 -3.41987191 -0.30816088
H 0.87720129 -2.55654705 -1.31042426
H -0.26039315 -1.44539379 -0.49311153
H 0.75521524 -2.56542577 0.46283559

HSEH1PBE/cc-pVDZ_S0

N 0.11171318 0.71118062 -0.48273649
C -0.23144602 2.03575222 -0.50469202
O -1.38643961 2.42120623 -0.60120404
N 0.78754766 2.99691173 -0.41123955
C 2.13382087 2.77093574 -0.30088666
O 2.95230215 3.66944302 -0.22531437
C 2.47791877 1.32426126 -0.28402678
N 3.73297031 0.99430829 -0.18514706
C 4.04256607 -0.32875498 -0.16906878
C 5.39581546 -0.71152290 -0.06500307
C 5.78042445 -2.03683470 -0.04318490
C 7.22480808 -2.41998035 0.06886303
C 4.76838328 -3.03858004 -0.12933921
C 5.14813183 -4.48588662 -0.10952160
C 3.42977224 -2.67930353 -0.23200698
C 3.04240145 -1.33137893 -0.25399771
N 1.72331936 -0.94371139 -0.35584905
C 1.37840980 0.38269025 -0.37928974
C 0.67760097 -1.95677998 -0.43984225
H 0.48275103 3.96647381 -0.42991697
H 6.13311611 0.09097858 -0.00209387
H 7.54734707 -3.02603264 -0.79276333
H 7.86609201 -1.53061388 0.12022403
H 7.40953915 -3.02828136 0.96874788
H 5.69305532 -4.73757169 0.81445317
H 4.26690918 -5.13584659 -0.17953470
H 5.82475209 -4.72692399 -0.94515365
H 2.68174312 -3.46783779 -0.29642569
H 0.83576567 -2.58117699 -1.32924618
H -0.28037255 -1.43721385 -0.51201520
H 0.69827819 -2.58685592 0.45940454

HSEH1PBE/cc-pVDZ_S1(r1)

N 0.14190134 0.70278061 -0.47433428
C -0.24269951 2.03564300 -0.50221855
O -1.42443788 2.34162343 -0.59603097
N 0.74184404 2.99333272 -0.42111650
C 2.11251292 2.77068581 -0.31145418
O 2.89243162 3.71509951 -0.24708150
C 2.48492849 1.36561484 -0.28366446
N 3.79203491 1.04338680 -0.17789970
C 4.06845810 -0.28250535 -0.16422126
C 5.39866666 -0.70332062 -0.05926092
C 5.77420460 -2.05423066 -0.04126833
C 7.21160448 -2.42760324 0.07390232
C 4.77100348 -3.06249357 -0.13334174
C 5.13975688 -4.51214092 -0.11934353
C 3.44170390 -2.67062129 -0.23608668
C 3.06377593 -1.31275082 -0.25272436
N 1.74721807 -0.93477197 -0.35161798
C 1.42973880 0.41291453 -0.37195084
C 0.69935947 -1.94186347 -0.43177524
H 0.43983517 3.96292914 -0.44397467
H 6.15994677 0.07564249 0.00959122
H 7.52920026 -3.04318899 -0.78457846
H 7.85822193 -1.54345278 0.12904013
H 7.38634918 -3.04756678 0.96962729
H 5.67690383 -4.78176631 0.80482488
H 4.24932167 -5.14959474 -0.19378456
H 5.81145117 -4.76441447 -0.95640833
H 2.67656708 -3.44278463 -0.30582719
H 0.85535861 -2.57059264 -1.31984251
H -0.26054870 -1.42726374 -0.50257952
H 0.72239074 -2.57367448 0.46758570

HSEH1PBE/cc-pVDZ_r7

N 0.09571059 0.71553687 -0.47741403
C -0.25151086 2.02918158 -0.50892160
O -1.43344432 2.38454494 -0.60741437
N 0.74809174 2.98471098 -0.42726387
C 2.11389292 2.76158698 -0.31234836
O 2.89897140 3.70322362 -0.24621062
C 2.48319307 1.35342096 -0.28254382
N 3.78459088 1.03584335 -0.17315530
C 4.07374355 -0.29462326 -0.15889763
C 5.41054897 -0.69877878 -0.05590824
C 5.79697266 -2.05472382 -0.03951267
C 7.22607441 -2.42333734 0.07987119
C 4.77791597 -3.06727799 -0.13713868
C 5.12963795 -4.50931463 -0.12913945
C 3.43552096 -2.66135056 -0.23989550
C 3.08536660 -1.31970100 -0.24832528
N 1.71323004 -0.91620012 -0.35022180
C 1.41110904 0.40274650 -0.37281552
C 0.69201023 -1.93563117 -0.42318403
H 0.44910872 3.95486254 -0.45214192
H 6.16700764 0.08446442 0.01221166
H 7.55013980 -3.05407477 -0.76856868
H 7.87401909 -1.54068351 0.13142494
H 7.40430660 -3.04615338 0.97655632
H 5.68195403 -4.78610340 0.78724096
H 4.23803412 -5.14395238 -0.20094274
H 5.80621983 -4.76438997 -0.96530787
H 2.67261382 -3.43454105 -0.31350916
H 0.85529891 -2.56784482 -1.31072487
H -0.28003252 -1.44249245 -0.48588628
H 0.73870827 -2.57589690 0.47227252

LC-OPBE/cc-pVDZ_S0

N 0.14841927 0.68611531 -0.48119706
C -0.19313343 1.99859071 -0.50353477
O -1.33363828 2.37827997 -0.60079923
N 0.80735630 2.95212981 -0.40979823
C 2.13565757 2.73131418 -0.30005266
O 2.94707053 3.61480444 -0.22483292
C 2.48491092 1.29981408 -0.28249455
N 3.71722100 0.97909544 -0.18459110
C 4.03771109 -0.33748083 -0.16775383
C 5.37673507 -0.71207230 -0.06554269
C 5.74379268 -2.02281593 -0.04574614
C 7.15995926 -2.43249033 0.06101559
C 4.74251184 -3.00722035 -0.12982246
C 5.13914442 -4.42876394 -0.10810050
C 3.42330903 -2.65628704 -0.23056513
C 3.05488571 -1.31797831 -0.25127532
N 1.74718631 -0.93579969 -0.35311569
C 1.39742758 0.36020372 -0.37781855
C 0.72920466 -1.94333254 -0.43631179
H 0.49740665 3.91684312 -0.42951351
H 6.11649349 0.08419069 -0.00295997
H 7.45322771 -3.04051239 -0.80260051

H 7.81699399 -1.56058919 0.11633519
H 7.31808034 -3.05018787 0.95261629
H 5.68719791 -4.65498656 0.81381412
H 4.27062397 -5.08824104 -0.17800082
H 5.82037140 -4.64459150 -0.93926406
H 2.67143041 -3.43700765 -0.29532241
H 0.89792115 -2.55977772 -1.32364227
H -0.22872226 -1.42924508 -0.50693230
H 0.76224030 -2.56894755 0.45999305

LC-OPBE/cc-pVDZ_S1(r1)

N 0.18483268 0.68186751 -0.47539251
C -0.20123043 1.99940613 -0.50407492
O -1.36169939 2.31632223 -0.60130987
N 0.77601823 2.94734358 -0.41772466
C 2.11930772 2.72544681 -0.30591499
O 2.90481967 3.64305341 -0.23680833
C 2.48801439 1.32880101 -0.28071372
N 3.77986356 1.01459272 -0.17539330
C 4.06402037 -0.28587033 -0.16185323
C 5.38999546 -0.70168641 -0.05928942
C 5.73945062 -2.02296928 -0.04390518
C 7.14757377 -2.43562372 0.06212058
C 4.73626853 -3.02348415 -0.13236924
C 5.13140083 -4.44188646 -0.11405855
C 3.42460638 -2.65551856 -0.23309164
C 3.05439606 -1.31461870 -0.25093453
N 1.76818587 -0.94829265 -0.34936363
C 1.44084890 0.39681540 -0.37155584
C 0.74194551 -1.94047550 -0.43103411
H 0.47282238 3.91389312 -0.44033672
H 6.14686223 0.07674387 0.00712688
H 7.42914199 -3.04896540 -0.80232562
H 7.81281180 -1.57106113 0.12104731
H 7.29319398 -3.06397314 0.94906092
H 5.67732992 -4.67786109 0.80764689
H 4.25944034 -5.09668042 -0.18653713
H 5.81187514 -4.66297177 -0.94556986
H 2.66173340 -3.42530200 -0.30110400
H 0.90057108 -2.56321583 -1.31774801
H -0.21255063 -1.42031217 -0.50080526
H 0.76714497 -2.57046231 0.46439816

LC-OPBE/cc-pVDZ_r4

N 0.14527496 0.70278539 -0.47753233
C -0.21813115 2.00425745 -0.50087568
O -1.36081743 2.38338120 -0.59399347
N 0.77408850 2.96545121 -0.41248847
C 2.08030489 2.64589621 -0.30805866
O 2.95757740 3.55931840 -0.23267442
C 2.48890156 1.30633724 -0.28282069
N 3.75312267 1.01562314 -0.18273358
C 4.05577282 -0.31884356 -0.16687685
C 5.38256153 -0.71076728 -0.06494473
C 5.74666828 -2.03177849 -0.04520788
C 7.16455644 -2.43931316 0.06327562
C 4.75360845 -3.00791138 -0.12960477
C 5.13587882 -4.43606574 -0.10907310
C 3.43417192 -2.63519483 -0.23083069
C 3.07500160 -1.30048706 -0.25045759
N 1.74631307 -0.92047052 -0.35343981
C 1.39519212 0.36661751 -0.37680119
C 0.73792211 -1.93351159 -0.43678012
H 0.48315871 3.93498378 -0.43278946
H 6.13260000 0.07609886 -0.00092871
H 7.46030265 -3.04776432 -0.79961128
H 7.82240441 -1.56773136 0.12000847
H 7.32294041 -3.05828842 0.95435398
H 5.67741671 -4.67693123 0.81343514
H 4.25846224 -5.08444728 -0.18132840
H 5.81350136 -4.66569004 -0.93997285
H 2.67451929 -3.40916914 -0.29631233
H 0.90918749 -2.55191361 -1.32303728
H -0.22572080 -1.42998572 -0.50834724
H 0.77225438 -2.56143085 0.45863483

LC-wHPBE/cc-pVDZ_S0

N -2.54058859 -1.30291142 -0.00003964
C -3.73134627 -0.62343572 -0.00007248
O -4.81005389 -1.18502724 -0.00011925
N -3.71120384 0.77827595 0.00000948
C -2.60805491 1.58396006 0.00017047
O -2.67086845 2.79483052 0.00006110
C -1.32647194 0.82122877 0.00005832
N -0.22586267 1.48716152 0.00007779
C 0.94941432 0.78531356 0.00005981
C 2.16316971 1.49448092 0.00007670
C 3.37615934 0.85002836 0.00005748
C 4.66182703 1.62166570 0.00004239
C 3.38618849 -0.57069065 0.00002633
C 4.69110929 -1.30607480 0.00005114
C 2.20036352 -1.27906476 0.00000583
C 0.96578890 -0.61575485 0.00002197
N -0.23700842 -1.29986069 -0.00000399
C -1.42078731 -0.63196051 0.00001533
C -0.23457178 -2.75661647 -0.00006419
H -4.61973096 1.23268632 -0.00003585
H 2.10219422 2.58366308 0.00010802
H 5.26933590 1.38135843 -0.88392222
H 4.47111044 2.70108660 0.00038867
H 5.26969863 1.38084091 0.88361094
H 5.28871648 -1.03965942 0.88318616
H 4.53912195 -2.39124291 -0.00033761
H 5.28913339 -1.03906412 -0.88261436
H 2.24102980 -2.36629120 -0.00002440
H 0.27616033 -3.12612875 -0.89711433
H -1.27367116 -3.08897834 -0.00008452
H 0.27614849 -3.12620325 0.89696191

LC-wHPBE/cc-pVDZ_S1(r1)

N -2.51327770 -1.28031330 -0.00000633
C -3.73946624 -0.62926160 0.00045289
O -4.78734402 -1.24939050 -0.00008374
N -3.73120155 0.74975155 0.00008665
C -2.61526000 1.56571676 -0.00042290
O -2.72107682 2.78180532 0.00020543
C -1.34730751 0.84213756 -0.00003442
N -0.20657951 1.55800219 0.00001105
C 0.93028477 0.84143123 0.00005502
C 2.16630341 1.51204632 0.00007710
C 3.37414729 0.84745979 0.00008030
C 4.65791091 1.60953519 0.00007743
C 3.39295053 -0.58680669 0.00005242
C 4.69427084 -1.32161882 0.00010906
C 2.20040998 -1.27773327 -0.00000754
C 0.96206060 -0.61323974 -0.00001279
N -0.21324158 -1.29159483 -0.00009915
C -1.41653553 -0.57249387 -0.00009963
C -0.22741092 -2.74379919 -0.00017063
H -4.63643978 1.21024062 0.00020086
H 2.13019994 2.60159125 0.00009460
H 5.26272359 1.35520495 -0.88231946
H 4.47902318 2.69026615 0.00030348
H 5.26297001 1.35485924 0.88220064
H 5.29579535 -1.06053977 0.88314637
H 4.53668215 -2.40605634 -0.00015001
H 5.29611910 -1.06015108 -0.88258733
H 2.22577999 -2.36545450 -0.00004271
H 0.28121222 -3.12210214 -0.89647687
H -1.26838355 -3.06969890 -0.00023313
H 0.28112984 -3.12217900 0.89614936

LC-wHPBE/cc-pVDZ_r5

N -2.54098272 -1.29058259 0.10787048
C -3.73388914 -0.62738127 0.06250708
O -4.81484350 -1.20553279 0.10494982
N -3.72700249 0.77539581 -0.03583177
C -2.62428839 1.59078781 -0.07680135
O -2.71455341 2.80182341 -0.14542361
C -1.34058958 0.85962127 -0.03235294
N -0.23350415 1.52651255 -0.05546291
C 0.92746694 0.82085763 -0.01870663
C 2.15167419 1.49953869 -0.05222146
C 3.37519859 0.84955750 -0.03176638
C 4.64875137 1.62052995 -0.07268858
C 3.40234713 -0.59972790 0.02640030
C 4.71314338 -1.31950150 0.05386810
C 2.22332348 -1.29346230 0.05756374
C 0.96029229 -0.63225452 0.03823723
N -0.20794397 -1.29897301 0.06884996
C -1.40549101 -0.60868429 0.04369183
C -0.23198479 -2.75740122 0.12369327
H -4.63741592 1.22309034 -0.06135093
H 2.11204385 2.58947131 -0.09931102
H 5.25148225 1.34473228 -0.95274323
H 4.46106700 2.69962680 -0.10693534
H 5.27416869 1.40375892 0.80769836
H 5.30846629 -1.02468534 0.93039004
H 4.56657457 -2.40516519 0.08916162
H 5.31511804 -1.08264069 -0.83534135
H 2.25661761 -2.38086472 0.10162967
H 0.25636426 -3.16353329 -0.76924248
H -1.27624336 -3.07055950 0.15564197
H 0.29108050 -3.09673760 1.02458255

LSDA/cc-pVDZ_S0

N -2.54569612 -1.30431571 -0.00008731
C -3.73442566 -0.63486475 0.00019191
O -4.82328576 -1.19891058 -0.00028227
N -3.70724110 0.76624479 0.00106381
C -2.60698866 1.58102203 0.00003739
O -2.68083541 2.80180991 -0.00079551
C -1.33648829 0.82576606 0.00003454
N -0.21887419 1.50349119 0.00002756
C 0.93954918 0.80498547 0.00006133
C 2.16128787 1.50198290 0.00011778
C 3.37506445 0.84598391 0.00006837
C 4.66018817 1.58809640 -0.00001314
C 3.38066448 -0.57763040 0.00001105
C 4.67661027 -1.29508244 0.00008559
C 2.18602167 -1.28577262 -0.00004723
C 0.95875281 -0.61679216 -0.00001661
N -0.23476976 -1.28985694 -0.00005119
C -1.42854299 -0.61341015 -0.00001822
C -0.25002757 -2.73136263 -0.00021665
H -4.62678035 1.22725500 0.00076423
H 2.10579812 2.60245080 0.00020450
H 5.27741517 1.32802498 -0.88721407
H 4.49735989 2.68261797 0.00114608
H 5.27869818 1.32622039 0.88573573
H 5.28746211 -1.01244800 0.88521551
H 4.54419459 -2.39333296 -0.00086760
H 5.28852464 -1.01091820 -0.88379576
H 2.21985913 -2.38536691 -0.00015186
H 0.26340427 -3.11829087 -0.90322310
H -1.31220341 -3.03733702 0.00077957
H 0.26531092 -3.11854283 0.90158227

LSDA/cc-pVDZ_S1(r2)

N -2.52194814 -1.29496392 0.00017538
C -3.73955601 -0.64378696 0.00018762
O -4.78799807 -1.29114996 -0.00038199
N -3.74090328 0.72741774 0.00010309
C -2.62381918 1.56519043 0.00006011
O -2.76628588 2.78769358 0.00015164
C -1.36152062 0.86344574 0.00022928
N -0.21386677 1.57962863 0.00033597
C 0.92596892 0.85206778 0.00024180
C 2.16187926 1.50809176 0.00023741
C 3.38580812 0.82896040 0.00009514
C 4.65999442 1.57382618 0.00006505
C 3.39916791 -0.58945266 -0.00008870
C 4.68713059 -1.32245781 -0.00024736
C 2.18404116 -1.26554792 -0.00010353
C 0.96128423 -0.57893065 0.00008161
N -0.23466838 -1.26008912 0.00007653
C -1.40715251 -0.55869961 0.00011400
C -0.24618123 -2.70029633 -0.00002060
H -4.65691814 1.19265276 -0.00007121
H 2.13912081 2.60986758 0.00035612
H 5.28217087 1.30011121 -0.88189105
H 4.50549933 2.66884930 0.00070894
H 5.28288703 1.29908308 0.88117807
H 5.30527323 -1.05912602 0.88627796
H 4.53420966 -2.41856799 -0.00079214
H 5.30558195 -1.05824754 -0.88626616
H 2.19234608 -2.36617131 -0.00027548
H 0.27503683 -3.08294563 -0.90135468
H -1.30214290 -3.02169957 0.00022384
H 0.27556936 -3.08303701 0.90096006

LSDA/cc-pVDZ_r7

N -2.54951760 -1.30497522 0.00009401
C -3.66685842 -0.62247761 0.00065194
O -4.79578265 -1.25857282 0.00057768
N -3.75184230 0.72445780 0.00031459
C -2.60804422 1.55316235 -0.00059878
O -2.77684006 2.76724126 0.00041112
C -1.36150635 0.83824927 -0.00017249
N -0.20328699 1.55882227 -0.00007848
C 0.92858885 0.84652155 -0.00000227
C 2.17724480 1.51365237 0.00007049
C 3.38337480 0.84062518 0.00006534
C 4.67461378 1.57532910 0.00015426
C 3.38707860 -0.57926433 -0.00002798
C 4.67584018 -1.31490398 -0.00004477
C 2.17469286 -1.26527013 -0.00010435
C 0.95903783 -0.58468981 -0.00011570
N -0.24792871 -1.26544488 -0.00021487
C -1.41713554 -0.57385533 -0.00015991
C -0.25150428 -2.70482111 -0.00032761
H -4.66263545 1.20138052 0.00061189
H 2.14472935 2.61530758 0.00014049
H 5.29143567 1.30951674 -0.88590498
H 4.52081353 2.67147236 0.00057267
H 5.29172170 1.30884527 0.88580543
H 5.29525310 -1.05173545 0.88566388
H 4.52277470 -2.41135333 -0.00050202
H 5.29565670 -1.05101731 -0.88524905
H 2.19178390 -2.36575165 -0.00019934
H 0.27148716 -3.08693426 -0.90085529
H -1.30485152 -3.03474190 -0.00029762
H 0.27161522 -3.08705832 0.90006843

LSDA/cc-pVDZ_r8

N -2.55139097 -1.30550240 0.02043133
C -3.74649854 -0.65095099 0.00427962
O -4.82594432 -1.25308791 0.00750844
N -3.72702107 0.73545988 -0.01200394
C -2.61667659 1.56540209 -0.01545866
O -2.73923051 2.79080718 -0.02501309
C -1.35006898 0.85293259 -0.00687253
N -0.22057205 1.56968465 -0.00180143
C 0.93864899 0.83961841 0.00280676
C 2.15993280 1.51553998 -0.00662267
C 3.40162350 0.84463337 0.00173675
C 4.65676171 1.59991266 0.10464498
C 3.40925827 -0.58940524 -0.02028986
C 4.67482743 -1.33448783 -0.10213496
C 2.17745296 -1.26981916 -0.00876524
C 0.97539375 -0.57881367 0.00609126
N -0.25598955 -1.28148501 0.01196395
C -1.42325188 -0.58364848 0.00659446
C -0.24963178 -2.70913514 0.02961772
H -4.64221084 1.20295676 -0.01561267
H 2.12598528 2.61659360 -0.00814720
H 5.54109012 1.09827425 -0.51227074
H 4.49366636 2.69326460 -0.17432790
H 5.06344084 1.56238357 1.23394654
H 5.52738363 -0.84383193 0.54521762
H 4.51618103 -2.42909081 0.17657970
H 5.09753136 -1.19336569 -1.21089030
H 2.19045954 -2.36979351 -0.03263003
H 0.27195188 -3.10708087 -0.86986335
H -1.30393897 -3.04037258 0.04069266
H 0.29162906 -3.08587773 0.92549112

LSDA/aug-cc-pVDZ_S0

N 0.11863990 0.70289597 -0.48107630
C -0.22819046 2.01340135 -0.50306925
O -1.39400404 2.40460526 -0.59913008
N 0.78188335 2.97667868 -0.41185890
C 2.12622025 2.75559383 -0.30262100
O 2.94087195 3.66975587 -0.22786435
C 2.47670009 1.32324227 -0.28511235
N 3.73683200 0.99153185 -0.18653827
C 4.05009541 -0.32011745 -0.16965737
C 5.39847939 -0.70730752 -0.06613875
C 5.77139123 -2.03380767 -0.04433202
C 7.19465287 -2.43761704 0.06546382
C 4.75842422 -3.03133439 -0.12931520
C 5.14819504 -4.45912333 -0.10655819
C 3.42237069 -2.67105727 -0.23216305
C 3.04830280 -1.32608334 -0.25412752
N 1.73859650 -0.94050847 -0.35542065
C 1.39226788 0.38186780 -0.37830195
C 0.70441642 -1.94328803 -0.43874096
H 0.47099263 3.95484182 -0.43070001
H 6.14900725 0.09452596 -0.00302396
H 7.50452922 -3.05731051 -0.80041081
H 7.85844619 -1.55719792 0.11975707
H 7.36650207 -3.06201662 0.96571351
H 5.70616601 -4.70118087 0.82125071
H 4.27143020 -5.12559519 -0.17692889
H 5.84105625 -4.69261836 -0.94095084
H 2.66207206 -3.46068928 -0.29709436
H 0.86623431 -2.57439491 -1.33200903
H -0.25942952 -1.41620160 -0.51294259
H 0.72584451 -2.57843725 0.46608736

LSDA/aug-cc-pVDZ_S1(r1/r2)

N 0.11587945 0.71052638 -0.47209089
C -0.17655067 1.98104330 -0.49136028
O -1.42126130 2.33728464 -0.58579144
N 0.72442755 2.98309036 -0.42028875
C 2.10739676 2.73781597 -0.31422898
O 2.86155397 3.71016599 -0.25607324
C 2.46771462 1.35117639 -0.28767249
N 3.78359404 1.02019952 -0.18445825
C 4.07044095 -0.28797687 -0.16730654
C 5.41400672 -0.71174176 -0.06139394
C 5.77295696 -2.04562168 -0.04123862
C 7.19607985 -2.45546034 0.07233358
C 4.76415683 -3.03816362 -0.13068578
C 5.13686363 -4.47424574 -0.11404232
C 3.43077299 -2.65033114 -0.23316855
C 3.07108388 -1.30571701 -0.25192118
N 1.74527486 -0.91618879 -0.35255184
C 1.42547156 0.40098342 -0.37182613
C 0.71993381 -1.92416187 -0.43638756
H 0.42859304 3.96521437 -0.44205607
H 6.18109804 0.07440390 0.00561965
H 7.50706244 -3.07417531 -0.79416446
H 7.86450273 -1.57841062 0.13232734
H 7.36253451 -3.08455445 0.97062080
H 5.68364153 -4.73945388 0.81440284
H 4.24743285 -5.12454887 -0.19037495
H 5.82299109 -4.72318232 -0.94986489
H 2.65913485 -3.42940142 -0.30116465
H 0.88567327 -2.55454540 -1.33058610
H -0.25295312 -1.41559968 -0.50733789

H 0.74948900 -2.56536980 0.46491740

LSDA/aug-cc-pVDZ_r5

N 0.11152454 0.71813972 -0.48467815
C -0.24846229 2.02013102 -0.50799238
O -1.43113642 2.39500102 -0.60585220
N 0.74831301 2.97977372 -0.41872355
C 2.10034571 2.75162087 -0.30643469
O 2.90893750 3.68442819 -0.23089517
C 2.46476924 1.34153205 -0.28620845
N 3.77277837 1.03000706 -0.18057905
C 4.04076904 -0.30326067 -0.16934474
C 5.40498816 -0.73391023 -0.06351207
C 5.79595918 -2.06473713 -0.04137455
C 7.22067172 -2.45448674 0.07124147
C 4.79425466 -3.04836995 -0.13033551
C 5.14313459 -4.47536020 -0.11305327
C 3.44045081 -2.64873494 -0.23694929
C 3.05242509 -1.29290728 -0.25441228
N 1.73010920 -0.93279860 -0.35471558
C 1.40069820 0.40753117 -0.37882900
C 0.70146404 -1.93611210 -0.43017232
H 0.44511983 3.95957736 -0.43768075
H 6.16031053 0.06508260 0.00327482
H 7.53481255 -3.07467679 -0.79323287
H 7.87907998 -1.57040277 0.12481171
H 7.39413755 -3.07675661 0.97317355
H 5.71083758 -4.73297794 0.80935711
H 4.25850866 -5.12981276 -0.18642313
H 5.84676073 -4.72126190 -0.94015614
H 2.67342534 -3.43327341 -0.30855727
H 0.84381756 -2.57203468 -1.32481444
H -0.26220544 -1.40678340 -0.50123837
H 0.71239742 -2.57111303 0.47649239

M052X/cc-pVDZ_S0

N 0.11061507 0.71176497 -0.48448211
C -0.22657428 2.03943468 -0.50666944
O -1.37695778 2.42943237 -0.60444994
N 0.79313472 3.00174552 -0.41196588
C 2.13588499 2.77269636 -0.30068491
O 2.95810032 3.66343819 -0.22455700
C 2.47942147 1.32138023 -0.28309470
N 3.72872474 0.99662151 -0.18310775
C 4.03960944 -0.33187437 -0.16713845
C 5.39424888 -0.71039502 -0.06235085
C 5.77531572 -2.03410380 -0.04220230
C 7.22319870 -2.42301154 0.07008895
C 4.76365941 -3.03378400 -0.13036523
C 5.14928954 -4.48474298 -0.11255663
C 3.42698659 -2.67951321 -0.23304505
C 3.04210170 -1.32823305 -0.25320881
N 1.72035657 -0.94124949 -0.35508266
C 1.37435043 0.37769084 -0.37971190
C 0.67943128 -1.96857747 -0.43770442
H 0.48774364 3.96889607 -0.43114416
H 6.12827832 0.09126603 0.00160362
H 7.53797317 -3.02315585 -0.79379660
H 7.85801354 -1.53295041 0.12324009
H 7.39913368 -3.02962927 0.96828935
H 5.69049919 -4.72945966 0.81107312
H 4.26734349 -5.12837790 -0.18304200
H 5.82129148 -4.71577699 -0.94986666
H 2.68286776 -3.46714461 -0.29935199
H 0.84681072 -2.58148627 -1.32908916
H -0.28167146 -1.46295722 -0.50255547
H 0.71981563 -2.59489002 0.45911476

M052X/cc-pVDZ_S1(r1/r5)

N 0.14651081 0.70604726 -0.47653985
C -0.23606958 2.04432578 -0.50535354
O -1.41020433 2.36003134 -0.60120180
N 0.75365252 2.99978452 -0.42154896
C 2.11663161 2.77023173 -0.30981373
O 2.90739831 3.70063115 -0.24282248
C 2.48459539 1.35749109 -0.28252505
N 3.78850181 1.04060164 -0.17591175
C 4.06683537 -0.28206037 -0.16286510
C 5.40057234 -0.69983447 -0.05646752
C 5.76879900 -2.04296460 -0.04051513
C 7.20879248 -2.42812956 0.07475003
C 4.76186381 -3.05563256 -0.13505844
C 5.14286691 -4.50597661 -0.12207802
C 3.43406902 -2.67664111 -0.23820610
C 3.05211626 -1.31828707 -0.25379647
N 1.74351094 -0.94538709 -0.35086832
C 1.42258725 0.41275349 -0.37246909
C 0.69655332 -1.96109357 -0.42916622
H 0.45388727 3.96826534 -0.44434024
H 6.15594175 0.08043641 0.01361748
H 7.51651858 -3.03624840 -0.78759222
H 7.85055865 -1.54436601 0.13177370
H 7.37258506 -3.04512343 0.96968566
H 5.67859529 -4.76430252 0.80181810
H 4.25518924 -5.14165063 -0.19707492
H 5.81313167 -4.74426876 -0.95962637
H 2.67610223 -3.45092591 -0.30941640
H 0.85672118 -2.58046650 -1.31903530
H -0.26371184 -1.45451586 -0.49306488
H 0.73390168 -2.58967888 0.46789819

M052X/aug-cc-pVDZ_S0

N 0.11096576 0.71395002 -0.48433186
C -0.22202266 2.03446238 -0.50592854
O -1.37785029 2.43087804 -0.60429943
N 0.79160210 3.00155127 -0.41135311
C 2.13256429 2.77129215 -0.30024205
O 2.95213779 3.66926792 -0.22442958
C 2.47907496 1.32216772 -0.28295542
N 3.72734145 0.99261754 -0.18338175
C 4.04150665 -0.33091344 -0.16720183
C 5.39721989 -0.70945232 -0.06229499
C 5.77335443 -2.03292253 -0.04249535
C 7.22005030 -2.42689924 0.06994122
C 4.75998396 -3.03214693 -0.13101647
C 5.14742781 -4.48235511 -0.11314512
C 3.42434668 -2.67883085 -0.23374355
C 3.04322016 -1.32698343 -0.25340837
N 1.72337701 -0.93801239 -0.35508813
C 1.37993509 0.37654526 -0.37917604
C 0.68473503 -1.97038641 -0.43732881
H 0.48552840 3.96561889 -0.43045133
H 6.13420108 0.08691986 0.00195643
H 7.53017474 -3.02604144 -0.79318703
H 7.85403947 -1.53930229 0.12290284
H 7.39134387 -3.03205541 0.96699360
H 5.68726168 -4.72360779 0.80930554
H 4.26650668 -5.12311731 -0.18352865
H 5.81782897 -4.70993122 -0.94947276
H 2.68048725 -3.46375715 -0.30040060
H 0.85786553 -2.57759768 -1.32827408
H -0.27830735 -1.47567096 -0.50075055
H 0.73310329 -2.59223575 0.45897107

M052X/aug-cc-pVDZ_S1(r1)

N 0.15000732 0.70886645 -0.47563021
C -0.23126937 2.04239208 -0.50373712
O -1.41090011 2.36037968 -0.59940695
N 0.75175713 3.00146010 -0.42111913
C 2.11284091 2.76920499 -0.31031950
O 2.90193162 3.70772437 -0.24343402
C 2.48634012 1.36053965 -0.28233070
N 3.78520863 1.03981122 -0.17611320
C 4.06916489 -0.28407290 -0.16248638
C 5.40038273 -0.69772219 -0.05647028
C 5.76654333 -2.04353369 -0.04065597
C 7.20482953 -2.43072859 0.07436568
C 4.75964082 -3.05542759 -0.13519442
C 5.14241446 -4.50548190 -0.12233444
C 3.43317574 -2.67726807 -0.23816612
C 3.05386500 -1.31731585 -0.25339796
N 1.74721241 -0.94403391 -0.35065293
C 1.42503778 0.41233211 -0.37221330
C 0.70191478 -1.96422759 -0.42966965
H 0.45225319 3.96717310 -0.44358471
H 6.15931456 0.07650182 0.01377891
H 7.50795623 -3.03874293 -0.78691167
H 7.84544981 -1.54924291 0.13082443
H 7.36460269 -3.04676786 0.96800851
H 5.67705279 -4.76030020 0.80019029
H 4.25556236 -5.13801584 -0.19670084
H 5.81030788 -4.74033766 -0.95945329
H 2.67506632 -3.44844504 -0.30949991
H 0.86611693 -2.57738273 -1.31985083
H -0.25892085 -1.46558981 -0.49244759
H 0.74414446 -2.58869690 0.46679859

M052X/aug-cc-pVDZ_r4

N 0.11054650 0.73065000 -0.47969809
C -0.24926665 2.03750888 -0.50317120
O -1.41217307 2.42603775 -0.59644459
N 0.74693294 3.01379943 -0.41611136
C 2.07624336 2.69465715 -0.30924707
O 2.92909720 3.64872297 -0.23457078
C 2.48871910 1.33666933 -0.28310587
N 3.76654575 1.02609072 -0.18110344
C 4.06365744 -0.31437820 -0.16569129
C 5.40757732 -0.71267976 -0.06107583
C 5.77880421 -2.04694490 -0.04155433
C 7.22701491 -2.44065239 0.07205093
C 4.77281038 -3.03651574 -0.13039432
C 5.14286848 -4.49457303 -0.11406735
C 3.43750215 -2.65857136 -0.23343312
C 3.06859088 -1.30904263 -0.25170295
N 1.72670253 -0.91718170 -0.35460155
C 1.38266553 0.38798381 -0.37746453
C 0.69748298 -1.95412657 -0.43788134
H 0.46004287 3.98230934 -0.43505330
H 6.15677340 0.07262216 0.00475533
H 7.53918128 -3.03949795 -0.79096056
H 7.86289361 -1.55407266 0.12691281
H 7.39862625 -3.04762269 0.96820488
H 5.67460709 -4.75188574 0.80902970
H 4.25175125 -5.12197324 -0.18756958
H 5.80924217 -4.73694313 -0.94976070
H 2.68465091 -3.43562403 -0.30093318
H 0.87385691 -2.56341897 -1.32750127
H -0.27106044 -1.47010045 -0.50325699
H 0.74611676 -2.57819500 0.45758625

M06/cc-pVDZ_S0

N -2.55720899 -1.30471587 -0.00002302
C -3.74905887 -0.62932898 0.00000510
O -4.83201400 -1.18761198 0.00002087
N -3.71997712 0.77866803 0.00002369
C -2.61224838 1.59022146 0.00000284
O -2.67963794 2.80380072 0.00001990
C -1.33771916 0.82326601 -0.00004032
N -0.22299052 1.49227031 -0.00003839
C 0.94345629 0.79358670 -0.00002008
C 2.16289382 1.50040739 -0.00000231
C 3.38033017 0.85387781 0.00000589
C 4.66358845 1.62193979 0.00003505
C 3.39020210 -0.57086911 -0.00001265
C 4.69116883 -1.30351918 0.00000465
C 2.19829304 -1.28372798 -0.00001997
C 0.96276659 -0.62281370 -0.00001748
N -0.24163765 -1.30130750 -0.00001814
C -1.43611678 -0.62482427 -0.00005131
C -0.23832863 -2.76144031 0.00004312
H -4.62869047 1.23962260 0.00012063
H 2.10174779 2.59230642 0.00000342
H 5.27860839 1.38258271 -0.88343511
H 4.47776452 2.70474165 0.00028368
H 5.27879154 1.38219500 0.88326984
H 5.29678454 -1.03708451 0.88230498
H 4.54461891 -2.39184967 -0.00039542
H 5.29720238 -1.03646212 -0.88181154
H 2.24452623 -2.37355811 -0.00004055
H 0.27764631 -3.13213561 -0.89682383
H -1.27596587 -3.10287736 -0.00002629
H 0.27751638 -3.13204937 0.89701995

M06/cc-pVDZ_S1(r1)

N -2.52858241 -1.28728537 -0.00000536
C -3.75600285 -0.63687490 0.00002556
O -4.80339064 -1.26402722 -0.00003949
N -3.74740647 0.74423914 0.00002531
C -2.62690612 1.57499754 0.00003866
O -2.75235138 2.79200928 0.00004100
C -1.36057631 0.85643188 0.00002957
N -0.21709873 1.57316156 0.00003330
C 0.92731409 0.84685684 0.00002514
C 2.15620049 1.50960116 0.00002634
C 3.38667432 0.83816975 0.00002255
C 4.65661403 1.60771503 -0.00000571
C 3.40858067 -0.58785799 0.00002454
C 4.70427299 -1.32822480 0.00005826
C 2.20157837 -1.27005521 0.00001469
C 0.96298317 -0.59590941 0.00001256
N -0.22651755 -1.28046460 -0.00000352
C -1.42412548 -0.56693039 0.00000627
C -0.23024472 -2.73700120 -0.00002901
H -4.65495897 1.20617799 0.00001731
H 2.12922846 2.60260847 0.00003124
H 5.27385219 1.35316616 -0.87951919
H 4.47961110 2.69090110 0.00021391
H 5.27412919 1.35282652 0.87920850
H 5.31582238 -1.07587449 0.88321972
H 4.54290523 -2.41482611 -0.00015615
H 5.31607199 -1.07554981 -0.88283247
H 2.22248829 -2.36104744 0.00001000
H 0.28654106 -3.11189962 -0.89627433
H -1.26691148 -3.07979067 -0.00004860
H 0.28651701 -3.11193229 0.89621663

M06/cc-pVDZ_r5/r6/r7/r8

N -2.57119646 -1.30340193 0.04359140
C -3.71437298 -0.62824049 0.04159260
O -4.83312947 -1.22211346 0.14758889
N -3.73748475 0.74244819 -0.07416105
C -2.61836418 1.51695115 -0.03540764
O -2.70866899 2.76895261 -0.03239674
C -1.34999040 0.82647645 -0.01355274
N -0.21353288 1.54734240 -0.01186385
C 0.93684079 0.82979696 -0.00622900
C 2.17287572 1.50956159 0.00101539
C 3.39033267 0.84914118 0.00312123
C 4.67648430 1.61482049 0.01119248
C 3.39841837 -0.56641880 -0.00223315
C 4.69192855 -1.31668869 -0.00097317
C 2.18758795 -1.26068582 -0.00830508
C 0.96268369 -0.59246091 -0.01056953
N -0.25515116 -1.28312918 -0.01465476
C -1.43751566 -0.58668903 0.00232941
C -0.24486072 -2.73708782 -0.01917949
H -4.64144144 1.21163368 -0.06076536
H 2.13065910 2.60302647 0.00493613
H 5.29601459 1.37631203 -0.86993245
H 4.49623969 2.69901997 0.01435636
H 5.28884825 1.36992426 0.89555973
H 5.30322563 -1.06949984 0.88394254
H 4.52815198 -2.40346910 -0.00515855
H 5.30847115 -1.06344026 -0.88053048
H 2.21931583 -2.35184869 -0.01227318
H 0.27830651 -3.10690504 -0.91408092
H -1.27657987 -3.09530223 -0.02281800
H 0.27221522 -3.11471360 0.87624497

M06/aug-cc-pVDZ_S0

N -2.55712395 -1.30296819 0.00001707
C -3.74103639 -0.62985435 0.00001582
O -4.83125084 -1.18844410 0.00014486
N -3.71940859 0.77580774 -0.00010968
C -2.61261809 1.58474264 0.00003509
O -2.68760466 2.80124773 0.00018163
C -1.33812332 0.82246851 -0.00001789
N -0.22141629 1.48802144 -0.00003767
C 0.94386438 0.79536475 -0.00006668
C 2.16339095 1.50320397 -0.00005227
C 3.37791012 0.85475306 -0.00003513
C 4.66300320 1.61924718 -0.00013867
C 3.38663452 -0.57085602 0.00005533
C 4.68813329 -1.30110911 0.00031236
C 2.19626215 -1.28389781 -0.00000890
C 0.96275301 -0.62119823 -0.00008440
N -0.24120996 -1.29758662 -0.00011233
C -1.43079955 -0.62262018 -0.00000927
C -0.23317644 -2.75926288 -0.00019249
H -4.62655484 1.23503441 0.00005138
H 2.10735104 2.59360175 -0.00004304
H 5.27294110 1.37897535 -0.88403256
H 4.47631710 2.69988635 0.00112753
H 5.27432525 1.37705498 0.88225186
H 5.28896972 -1.03407047 0.88291740
H 4.53958351 -2.38710005 -0.00104709
H 5.29065058 -1.03196314 -0.88047439
H 2.24076289 -2.37173764 0.00005814
H 0.28402795 -3.12073278 -0.89700179
H -1.26454275 -3.10933041 -0.00022212
H 0.28393708 -3.12085719 0.89662366

M06/aug-cc-pVDZ_S1(r1)

N -2.52218901 -1.29325845 -0.00022815
C -3.74615444 -0.64927228 -0.00057664
O -4.79747508 -1.28019049 0.00039776
N -3.74818383 0.72926188 -0.00004599
C -2.63064379 1.55923386 0.00005151
O -2.76623383 2.78000344 0.00048696
C -1.36175163 0.85136007 -0.00012577
N -0.22205311 1.56499292 -0.00006186
C 0.92914572 0.84469149 -0.00004277
C 2.15142771 1.51174328 -0.00000092
C 3.38593607 0.84124846 0.00004632
C 4.65249606 1.61280861 -0.00006337
C 3.41120872 -0.58348972 0.00011574
C 4.70939049 -1.31928018 0.00030647
C 2.20738640 -1.26772790 0.00003677
C 0.96646229 -0.59456088 -0.00005183
N -0.21905278 -1.28162440 -0.00014291
C -1.41868902 -0.57368847 -0.00014979
C -0.21522693 -2.73980690 -0.00021634
H -4.65512997 1.18759121 0.00031323
H 2.12782600 2.60309344 -0.00001409
H 5.26653377 1.35838097 -0.87944919
H 4.47122790 2.69319489 0.00075978
H 5.26760898 1.35709249 0.87816972
H 5.31565241 -1.06504497 0.88363088
H 4.54780171 -2.40374131 -0.00044209
H 5.31660815 -1.06391493 -0.88201963
H 2.22862805 -2.35664182 0.00005458
H 0.30167141 -3.10627190 -0.89675112
H -1.24580072 -3.09102865 -0.00034820
H 0.30146890 -3.10637317 0.89639460

M06/aug-cc-pVDZ_r5/r6

N -2.56949312 -1.30530046 0.03099570
C -3.71073245 -0.63888949 0.02773039
O -4.83192630 -1.15646545 0.29786600
N -3.72779733 0.72303258 -0.25298714
C -2.60950744 1.51555379 -0.09426366
O -2.75912575 2.75475478 -0.03828812

C	-1.35141246	0.82436127	-0.03098614
N	-0.21404700	1.54712889	0.02353665
C	0.93695585	0.83079303	0.02176722
C	2.17156874	1.50898696	0.08604610
C	3.38853765	0.84762332	0.07633342
C	4.67408926	1.60895406	0.14881782
C	3.39761676	-0.56634907	-0.00271297
C	4.69199026	-1.31370552	-0.02084135
C	2.18843134	-1.25818388	-0.06304244
C	0.96481431	-0.58904091	-0.05262511
N	-0.25262997	-1.28122561	-0.10463897
C	-1.42883643	-0.58843113	-0.04483080
C	-0.23730251	-2.73400745	-0.18073806
H	-4.63343149	1.19214439	-0.26666593
H	2.13484899	2.59936128	0.14418297
H	5.30654506	1.41386340	-0.73112272
H	4.49164124	2.68931094	0.20408386
H	5.26516956	1.31683579	1.03057761
H	5.28615600	-1.11030836	0.88381309
H	4.52633280	-2.39640544	-0.08205315
H	5.31663313	-1.01534524	-0.87733476
H	2.21922557	-2.34578469	-0.12061856
H	0.30646268	-3.04929469	-1.08079795
H	-1.26331497	-3.09687276	-0.22729637
H	0.25849404	-3.15127276	0.70620032

M06-HF/cc-pVDZ_S0

N	-2.55732457	-1.30468563	0.00003800
C	-3.74916172	-0.62922882	0.00001807
O	-4.83216374	-1.18739678	-0.00001541
N	-3.71998043	0.77872819	0.00005802
C	-2.61225667	1.59025356	-0.00006982
O	-2.67976143	2.80383057	-0.00029082
C	-1.33772559	0.82327541	0.00001270
N	-0.22294447	1.49217578	0.00007150
C	0.94352320	0.79347367	0.00008578
C	2.16295966	1.50037976	0.00013073
C	3.38042721	0.85389320	0.00007032
C	4.66376605	1.62177367	0.00015400
C	3.39031143	-0.57084809	-0.00011829
C	4.69143140	-1.30321033	-0.00022101
C	2.19839237	-1.28378859	-0.00014756
C	0.96275512	-0.62294066	0.00000967
N	-0.24170312	-1.30140422	0.00007312
C	-1.43622295	-0.62484998	0.00006676
C	-0.23860767	-2.76158553	0.00009822
H	-4.62866644	1.23968820	0.00011016
H	2.10165137	2.59228042	0.00021589
H	5.27871949	1.38227360	-0.88332161
H	4.47827869	2.70466252	0.00056874
H	5.27900653	1.38163142	0.88325049
H	5.29697817	-1.03660776	0.88207839
H	4.54536153	-2.39163145	-0.00082514
H	5.29747861	-1.03565884	-0.88187737
H	2.24464989	-2.37363506	-0.00034699
H	0.27759387	-3.13242669	-0.89656153
H	-1.27628420	-3.10297325	-0.00033134
H	0.27675101	-3.13235566	0.89726836

M06-HF/cc-pVDZ_S1(r1)

N	-2.52848901	-1.28730724	-0.00001609
C	-3.75588011	-0.63692930	0.00023073
O	-4.80328930	-1.26406433	-0.00004117
N	-3.74733340	0.74423173	0.00010392
C	-2.62688444	1.57498115	-0.00011936
O	-2.75229669	2.79202112	0.00016928
C	-1.36051081	0.85643926	0.00001273
N	-0.21702668	1.57317315	0.00003601
C	0.92738291	0.84684639	0.00003170
C	2.15627066	1.50958337	0.00006015
C	3.38676252	0.83816029	0.00004790
C	4.65668002	1.60773269	0.00005208
C	3.40866579	-0.58785505	0.00000562
C	4.70434647	-1.32823503	0.00003115
C	2.20165358	-1.27004608	-0.00003771
C	0.96303549	-0.59593472	-0.00003065
N	-0.22648505	-1.28047641	-0.00007985
C	-1.42405955	-0.56690565	-0.00004304
C	-0.23043756	-2.73700352	-0.00013640
H	-4.65492418	1.20608857	0.00016072
H	2.12933124	2.60259439	0.00009536
H	5.27390746	1.35325746	-0.87949336
H	4.47965917	2.69091262	0.00035494
H	5.27422516	1.35278242	0.87922930
H	5.31586175	-1.07595946	0.88323604
H	4.54298677	-2.41484131	-0.00027058
H	5.31617767	-1.07550026	-0.88281773
H	2.22258449	-2.36103601	-0.00007175
H	0.28630749	-3.11198178	-0.89636818
H	-1.26718281	-3.07958691	-0.00021401
H	0.28619358	-3.11204897	0.89613426

M06-HF/cc-pVDZ_r5

N	-2.57152476	-1.30310094	-0.00035589
C	-3.71524041	-0.62818232	-0.00071783
O	-4.83455528	-1.23290217	0.00057359
N	-3.73832123	0.74556455	0.00013179
C	-2.61900799	1.51777635	0.00012864
O	-2.70451162	2.77095414	0.00063146
C	-1.34984055	0.82742418	-0.00018620
N	-0.21329767	1.54662588	-0.00011676
C	0.93746065	0.82957325	-0.00009623
C	2.17328244	1.50950456	-0.00005131
C	3.39065470	0.84906299	0.00000477
C	4.67693849	1.61464854	0.00003248
C	3.39852163	-0.56652583	0.00002809
C	4.69188996	-1.31703381	0.00012277
C	2.18769630	-1.26076482	-0.00001500
C	0.96282482	-0.59248871	-0.00007383
N	-0.25525187	-1.28246260	-0.00012432
C	-1.43792835	-0.58612701	-0.00020626
C	-0.24534299	-2.73645290	-0.00013963
H	-4.64211277	1.21449908	0.00059636
H	2.13104597	2.60298419	-0.00006119
H	5.29277406	1.37306270	-0.88282770
H	4.49677331	2.69887471	0.00019664
H	5.29289752	1.37280368	0.88273258
H	5.30565588	-1.06705814	0.88254160
H	4.52793271	-2.40380589	-0.00008896
H	5.30597418	-1.06674960	-0.88198247
H	2.21940034	-2.35193843	0.00001047
H	0.27493067	-3.11007280	-0.89525260
H	-1.27711186	-3.09448935	-0.00031930
H	0.27462638	-3.11011084	0.89513626

M06L/cc-pVDZ_S0

N	0.10166873	0.71246294	-0.48291790
C	-0.24316774	2.03896680	-0.50266497
O	-1.40068016	2.42676594	-0.59740805
N	0.78258314	3.00153284	-0.40850248
C	2.13574149	2.77570412	-0.29864978
O	2.95429516	3.67937551	-0.22289205
C	2.47467535	1.33141087	-0.28375867
N	3.73798379	1.00205264	-0.18525127
C	4.04509948	-0.32249269	-0.17034870
C	5.39806647	-0.70904114	-0.06645807
C	5.77960152	-2.03645194	-0.04429112
C	7.21635670	-2.43032036	0.06817742
C	4.76678000	-3.03823778	-0.12977326
C	5.15224821	-4.47898549	-0.10756892
C	3.42733333	-2.67719760	-0.23327902
C	3.04421896	-1.32876698	-0.25557245
N	1.72317070	-0.94009218	-0.35791103
C	1.37529928	0.39363491	-0.37967350
C	0.68376540	-1.96131040	-0.44336004
H	0.48009822	3.97213948	-0.42573247
H	6.13906813	0.09005399	-0.00355083
H	7.53449674	-3.04807541	-0.78485722
H	7.87155841	-1.55313176	0.11298880
H	7.39989887	-3.03566830	0.96858649
H	5.70525376	-4.72826486	0.81046370
H	4.28029423	-5.13890287	-0.17165468
H	5.82828149	-4.72243776	-0.94101691
H	2.67732818	-3.46440905	-0.29740904
H	0.84651756	-2.58380558	-1.33084325
H	-0.28041052	-1.45878936	-0.51361502
H	0.71157911	-2.59466709	0.45092957

M06L/cc-pVDZ_S1(r1)/r6

N	0.09194782	0.71364161	-0.47559301
C	-0.18816000	1.99373677	-0.48866246
O	-1.42545652	2.39134990	-0.57954065
N	0.72082733	2.99632570	-0.41360337
C	2.11907400	2.75070176	-0.30797836
O	2.86541176	3.71906234	-0.24670831
C	2.46350558	1.35405776	-0.28809909
N	3.79493391	1.02793810	-0.18575787
C	4.06872802	-0.28546566	-0.17104087
C	5.41978438	-0.71279287	-0.06542203
C	5.78344615	-2.04661551	-0.04291950
C	7.21872641	-2.45010694	0.07243884
C	4.77140130	-3.04378733	-0.12957755
C	5.13793453	-4.49128204	-0.10822518
C	3.43454139	-2.65511361	-0.23428489
C	3.06779765	-1.30788180	-0.25577459
N	1.73109619	-0.91416020	-0.35880373
C	1.41472258	0.41454060	-0.37515569
C	0.69943220	-1.93838564	-0.44568095
H	0.42243337	3.96775725	-0.43175489
H	6.17756017	0.07090808	-0.00057211
H	7.53518462	-3.06866270	-0.78111573
H	7.88168528	-1.57848287	0.12187864
H	7.39584145	-3.06067600	0.97100650
H	5.68059955	-4.75995974	0.81149793
H	4.25396793	-5.13614646	-0.17646533
H	5.80994042	-4.75315946	-0.94042327
H	2.67348929	-3.43242072	-0.29945167
H	0.87081662	-2.56411306	-1.33036676
H	-0.27274969	-1.45375838	-0.51965375
H	0.73054036	-2.57399743	0.44799499

M06L/cc-pVDZ_r5

N	0.09463185	0.74004337	-0.47724541
C	-0.22160715	2.02238352	-0.49217735
O	-1.42989687	2.39104268	-0.58357158
N	0.76952000	3.00428753	-0.40703157
C	2.08390174	2.71159639	-0.30654969
O	2.93534221	3.65721539	-0.23253181
C	2.44322839	1.32898775	-0.28912356
N	3.77861964	1.02892028	-0.18727666
C	4.06201671	-0.28567609	-0.17276849
C	5.41356052	-0.70643913	-0.06808665
C	5.78389622	-2.03971898	-0.04432111
C	7.22130403	-2.43612621	0.07068156
C	4.77632458	-3.03894714	-0.12882596
C	5.14687311	-4.48505393	-0.10526927
C	3.43597566	-2.65493239	-0.23383284
C	3.06003268	-1.31192382	-0.25723077
N	1.71847015	-0.93097228	-0.36145499
C	1.39817913	0.40274179	-0.37753352
C	0.68559503	-1.94803467	-0.44850751
H	0.48065929	3.97996643	-0.42276845
H	6.16806287	0.08050119	-0.00452893
H	7.54005776	-3.05605526	-0.78103240
H	7.88013849	-1.56113031	0.11612109
H	7.40282633	-3.04217056	0.97148314
H	5.69282799	-4.74970002	0.81372892
H	4.26504362	-5.13310948	-0.17026441
H	5.81834904	-4.74629730	-0.93817380
H	2.67735092	-3.43504277	-0.29779404
H	0.84505780	-2.57545560	-1.33494734
H	-0.28294681	-1.45176155	-0.52146884
H	0.70560808	-2.58608736	0.44448755

M06L/aug-cc-pVDZ_S0

N	0.10264342	0.71489468	-0.48357964
C	-0.23842559	2.03596472	-0.50301878
O	-1.40046268	2.42803280	-0.59930502
N	0.78135751	3.00142167	-0.40737004
C	2.13241097	2.77378204	-0.29755420
O	2.94861224	3.68359814	-0.22049271
C	2.47452574	1.33208481	-0.28340357
N	3.73685370	0.99915860	-0.18521777
C	4.04647769	-0.32190122	-0.17037811
C	5.39995775	-0.70841388	-0.06687669
C	5.77800288	-2.03543409	-0.04498930
C	7.21335801	-2.43500699	0.06661344
C	4.76398611	-3.03666583	-0.13008955
C	5.15250894	-4.47642389	-0.10719121
C	3.42536161	-2.67648705	-0.23322822
C	3.04480013	-1.32794226	-0.25544848
N	1.72488110	-0.93778145	-0.35778177
C	1.37932415	0.39279872	-0.37950819
C	0.68687158	-1.96257091	-0.44298052
H	0.47865977	3.96931252	-0.42370863
H	6.14294378	0.08629358	-0.00414864
H	7.52564128	-3.04950352	-0.78866077
H	7.86855745	-1.56003363	0.11415304
H	7.39066202	-3.04254519	0.96457719
H	5.70129583	-4.72100269	0.81257156
H	4.28171883	-5.13474594	-0.17459825
H	5.83096167	-4.71424356	-0.93795692
H	2.67620787	-3.46152164	-0.29703792
H	0.85693624	-2.58188167	-1.32908611
H	-0.27868487	-1.46868264	-0.51412350
H	0.72105157	-2.59150055	0.45200509

M06L/aug-cc-pVDZ_S1(r1)

N	0.12960423	0.70643069	-0.47408418
C	-0.24956056	2.03778288	-0.50007435
O	-1.44036619	2.34630128	-0.59320036
N	0.73294801	2.99951120	-0.41886938
C	2.11126036	2.77384246	-0.30954049
O	2.88757250	3.73195904	-0.24656189
C	2.48322981	1.37630054	-0.28292473
N	3.79433519	1.05248309	-0.17744990
C	4.06924913	-0.28254489	-0.16438291
C	5.39866190	-0.70283309	-0.05939710
C	5.77324415	-2.05927911	-0.04143877
C	7.20258201	-2.44265887	0.07304382
C	4.77055404	-3.06186908	-0.13385748
C	5.14477864	-4.50609425	-0.11955993
C	3.44138208	-2.66581011	-0.23616846
C	3.07095638	-1.30492041	-0.25264500
N	1.74729761	-0.92448386	-0.35262409
C	1.42802673	0.41989835	-0.37175594
C	0.70838437	-1.94551652	-0.43423075
H	0.43201861	3.96680478	-0.44098275
H	6.16547434	0.06854197	0.00977924
H	7.51284228	-3.06722558	-0.77778071
H	7.86054978	-1.57101056	0.12432111
H	7.37327733	-3.06240182	0.96607424
H	5.68762485	-4.77092168	0.79860934
H	4.26275685	-5.14967289	-0.18915189
H	5.81389300	-4.75581331	-0.95496901
H	2.67444658	-3.43377117	-0.30553403
H	0.87663011	-2.56842343	-1.31925795
H	-0.25886474	-1.45642927	-0.50455020
H	0.74420598	-2.57512062	0.46134795

M06L/aug-cc-pVDZ_r6

N	0.09255098	0.71776913	-0.47508924
C	-0.18956098	1.99446127	-0.48606445
O	-1.42657444	2.39544280	-0.57776809
N	0.72083272	2.99859961	-0.40728929
C	2.11348514	2.74829775	-0.30271353
O	2.86358175	3.72020226	-0.23773947
C	2.46242414	1.35546758	-0.28718491
N	3.79136060	1.02727314	-0.18795694
C	4.06950126	-0.28796685	-0.17312969
C	5.41949619	-0.71272174	-0.07004944
C	5.78213125	-2.04713741	-0.04569733
C	7.21679243	-2.45388945	0.06852318
C	4.77044232	-3.04293687	-0.12660374
C	5.13988727	-4.49002927	-0.10077054
C	3.43387737	-2.65513510	-0.22995421
C	3.06872086	-1.30797262	-0.25590947
N	1.73243801	-0.91394554	-0.36199445
C	1.41625233	0.41387400	-0.37541026
C	0.70294931	-1.94086928	-0.45562833
H	0.42201507	3.96736716	-0.42267591
H	6.17944034	0.06650799	-0.00716642
H	7.52747609	-3.07292975	-0.78450811
H	7.87839871	-1.58312645	0.11545365
H	7.39016920	-3.06143746	0.96770670
H	5.68326158	-4.75041339	0.81869352
H	4.25654919	-5.13330686	-0.16525869
H	5.80934864	-4.74961645	-0.93327342
H	2.67325024	-3.43022911	-0.29140134
H	0.88795248	-2.56538611	-1.33634670
H	-0.26862756	-1.46216052	-0.54075010
H	0.72917287	-2.57099667	0.44014051

M06L/aug-cc-pVDZ_r7

N	0.09135168	0.71916232	-0.47962966
C	-0.25924666	2.03511325	-0.50871780
O	-1.44555607	2.39141734	-0.60749468
N	0.74321661	2.99271389	-0.42396202
C	2.11442267	2.76587507	-0.30989364
O	2.89960285	3.71725107	-0.24246780
C	2.48268581	1.36188438	-0.28286103
N	3.78504163	1.04501477	-0.17413360
C	4.07295203	-0.29859197	-0.16084011
C	5.41027163	-0.69842034	-0.05699534
C	5.79634268	-2.05662333	-0.03857636
C	7.21789475	-2.43287526	0.08600969
C	4.77681068	-3.06608946	-0.13985998
C	5.13277815	-4.50210920	-0.13728772
C	3.43210398	-2.66052044	-0.24027647
C	3.08700707	-1.31590404	-0.24905382
N	1.71303138	-0.91344973	-0.35167173
C	1.40640445	0.40961402	-0.37453957
C	0.69788106	-1.94218699	-0.42310612
H	0.44754842	3.96174823	-0.44673360
H	6.17016460	0.07907990	0.01192184
H	7.53707807	-3.09193478	-0.73861219
H	7.87594328	-1.56102904	0.11645374
H	7.39676354	-3.03595087	0.99295192
H	5.71240813	-4.77553702	0.75921029
H	4.24872306	-5.14312139	-0.18757801
H	5.79139138	-4.75690124	-0.98406431
H	2.67009573	-3.43196067	-0.31418634
H	0.86958932	-2.57254974	-1.30560779
H	-0.27960513	-1.47147874	-0.48609743
H	0.75389858	-2.57858425	0.46988315

M11L/cc-pVDZ_S0

N	0.13641650	0.69259546	-0.48103682
C	-0.19426670	2.00460212	-0.50336886
O	-1.33159091	2.38985954	-0.60093049
N	0.81410686	2.95323936	-0.40898799
C	2.14756769	2.73890819	-0.30089575
O	2.95259684	3.62745801	-0.22788485
C	2.48693125	1.30402708	-0.28222920
N	3.73616068	0.98584882	-0.18301724
C	4.03227790	-0.32348759	-0.16805087
C	5.37489047	-0.70767666	-0.06516658
C	5.75769882	-2.02431971	-0.04446240
C	7.18420257	-2.40913892	0.06623412
C	4.75029889	-3.01803111	-0.13123697
C	5.12763528	-4.44834046	-0.11368539

C 3.41941340 -2.65976551 -0.23246996
C 3.03670101 -1.32177175 -0.25283363
N 1.73263346 -0.93941586 -0.35309804
C 1.39320256 0.37526806 -0.37704834
C 0.70709755 -1.94327382 -0.43497220
H 0.50635682 3.92073447 -0.43127136
H 6.11614799 0.09945475 -0.00101240
H 7.51337775 -3.01703603 -0.79592827
H 7.83594420 -1.52350120 0.12034783
H 7.37427806 -3.02345144 0.96503655
H 5.67627305 -4.70793605 0.80988020
H 4.24937556 -5.10753947 -0.18482949
H 5.80920682 -4.69388325 -0.94827495
H 2.67152696 -3.45599542 -0.29882966
H 0.86393280 -2.57066433 -1.32736767
H -0.26289440 -1.44339357 -0.50445953
H 0.73149687 -2.58032006 0.46403603

M11L/cc-pVDZ_S1(r1)

N 0.16336094 0.68248754 -0.46973312
C -0.20677986 2.00213895 -0.49939935
O -1.37219498 2.30763187 -0.59232373
N 0.76585404 2.94993142 -0.42147308
C 2.12376841 2.74015266 -0.31254631
O 2.88680361 3.67679996 -0.25321991
C 2.49606667 1.34827963 -0.28105818
N 3.79559214 1.04149525 -0.17476413
C 4.05635691 -0.27745883 -0.16286982
C 5.37501599 -0.70037720 -0.05902728
C 5.75229074 -2.04726782 -0.04265083
C 7.17173864 -2.41669912 0.07179593
C 4.75652340 -3.04478338 -0.13585566
C 5.11988495 -4.47864422 -0.12415663
C 3.43645683 -2.64782799 -0.23723490
C 3.06394703 -1.29708452 -0.25115985
N 1.75607093 -0.92550859 -0.34834435
C 1.44438270 0.40347174 -0.36842480
C 0.73042176 -1.92505167 -0.42680453
H 0.45943442 3.91652786 -0.44945633
H 6.14203068 0.08185428 0.01247079
H 7.49698151 -3.03752455 -0.78478844
H 7.82841665 -1.53622094 0.12950434
H 7.35251464 -3.04511886 0.96491985
H 5.65724807 -4.75904023 0.80088248
H 4.23016056 -5.12231329 -0.20160042
H 5.79617721 -4.73818669 -0.95979071
H 2.66895400 -3.42588610 -0.30988134
H 0.88529010 -2.55589212 -1.31863130
H -0.24228043 -1.43186627 -0.49375386
H 0.75850838 -2.56496512 0.47156128

M11L/cc-pVDZ_r6

N 0.12583953 0.70764491 -0.47933672
C -0.21770311 2.00992311 -0.50365994
O -1.36843237 2.38412397 -0.59771533
N 0.78097780 2.95546652 -0.41810409
C 2.12109612 2.73316306 -0.30965570
O 2.91990202 3.63929843 -0.24094379
C 2.47155395 1.32023393 -0.28608820
N 3.77719374 1.02319605 -0.18167286
C 4.02256861 -0.30120960 -0.16976222
C 5.38246918 -0.73430488 -0.06312443
C 5.78392649 -2.05673573 -0.04234310
C 7.21256821 -2.42581106 0.07426394
C 4.79114410 -3.03641061 -0.13247071
C 5.12355020 -4.46648003 -0.11969619
C 3.43533467 -2.63441631 -0.23781855
C 3.03828675 -1.28951999 -0.25361560
N 1.72276780 -0.93116793 -0.35226428
C 1.40004561 0.40377271 -0.37604769
C 0.70552600 -1.93390138 -0.42723558
H 0.48122480 3.92479344 -0.44025385
H 6.12862191 0.06982145 0.00632911
H 7.54730066 -3.03353657 -0.78578667
H 7.85714272 -1.53564748 0.12870093
H 7.40137686 -3.03673371 0.97564772
H 5.67428562 -4.74323941 0.80119861
H 4.23408060 -5.10851290 -0.19632138
H 5.81341277 -4.72628592 -0.94706284
H 2.68211621 -3.42772311 -0.31141317
H 0.84338372 -2.56588831 -1.32237880
H -0.26587731 -1.43459153 -0.49010453
H 0.72331270 -2.57626747 0.47092172

M11L/cc-pVDZ_r7

N 0.12529366 0.69504753 -0.47500684
C -0.21563454 2.00005207 -0.50554703
O -1.37735131 2.35121876 -0.60211111
N 0.77468594 2.94354025 -0.42509972
C 2.12676542 2.73171496 -0.31152104
O 2.89776832 3.66257243 -0.24753653
C 2.49369883 1.33409634 -0.28075166
N 3.78562753 1.03136197 -0.17148887
C 4.05900163 -0.29303380 -0.15962421
C 5.38728898 -0.69580794 -0.05801968
C 5.77547283 -2.04393783 -0.04273026
C 7.18700583 -2.40833915 0.07768939
C 4.76225560 -3.04757792 -0.14066506
C 5.10766950 -4.47325364 -0.13300713
C 3.42617335 -2.64184838 -0.24185600
C 3.07835825 -1.30741855 -0.24868225
N 1.72347407 -0.91431237 -0.34878533
C 1.42219958 0.39520660 -0.37137131
C 0.72136844 -1.92072312 -0.41983656
H 0.47329932 3.91181758 -0.45157759
H 6.14678904 0.09317090 0.01150463
H 7.52236953 -3.05328576 -0.76120641
H 7.84461503 -1.52861705 0.12586465
H 7.37574912 -3.03474325 0.97515832
H 5.66476540 -4.76044704 0.78195445
H 4.21709422 -5.11482210 -0.20497859
H 5.79005161 -4.73882511 -0.96609196
H 2.66434682 -3.42367206 -0.31777624
H 0.88222836 -2.56060779 -1.30831363
H -0.26141501 -1.44572292 -0.48157496
H 0.76798131 -2.56974995 0.47517435

MN15/cc-pVDZ_S0

N 0.10202917 0.72024235 -0.48348999
C -0.24195995 2.04987256 -0.50562933
O -1.39741392 2.43840775 -0.60310240
N 0.78113853 3.01236971 -0.41100654
C 2.13005133 2.78141914 -0.29981817
O 2.95594508 3.67516690 -0.22303334
C 2.47282543 1.33004930 -0.28340992
N 3.72675410 0.99929185 -0.18441995
C 4.04343537 -0.33075734 -0.16820429
C 5.40002681 -0.71108538 -0.06467284
C 5.78322422 -2.03834411 -0.04301896
C 7.22887232 -2.43308882 0.06816845
C 4.76938043 -3.03922458 -0.12944912
C 5.15907760 -4.48850461 -0.10930918
C 3.42928661 -2.68157267 -0.23204356
C 3.04298481 -1.32935551 -0.25350567
N 1.71722625 -0.94026999 -0.35592722
C 1.36831325 0.38537346 -0.37969329
C 0.67557592 -1.96396531 -0.44012958
H 0.47512022 3.98514768 -0.42988533
H 6.13681199 0.09255621 -0.00096740
H 7.54376808 -3.03980850 -0.79526620
H 7.87546215 -1.54788154 0.12161845
H 7.40631906 -3.04582882 0.96592177
H 5.70540971 -4.73337266 0.81511648
H 4.28110494 -5.14258691 -0.17993320
H 5.83840746 -4.72196203 -0.94423482
H 2.68209777 -3.47182782 -0.29681004
H 0.84112724 -2.58703960 -1.32876190
H -0.28809187 -1.45591476 -0.51175614
H 0.70468657 -2.59445231 0.45823905

MN15/cc-pVDZ_S1(r1)

N 0.13675404 0.71352491 -0.47560316
C -0.25215973 2.05030911 -0.50382492
O -1.43221224 2.36384669 -0.59896547
N 0.74077391 3.00816552 -0.42019012
C 2.11033036 2.77905354 -0.30941285
O 2.90216671 3.71493697 -0.24295328
C 2.47938638 1.36676131 -0.28276550
N 3.78780189 1.04492614 -0.17686291
C 4.06990868 -0.28083566 -0.16332758
C 5.40550062 -0.70122749 -0.05802986
C 5.77601037 -2.04855323 -0.04114546
C 7.21446838 -2.43836849 0.07256007
C 4.76761391 -3.06170644 -0.13361028
C 5.15153314 -4.51078397 -0.11834070
C 3.43727662 -2.67687027 -0.23643267
C 3.05662327 -1.31660249 -0.25319194
N 1.74093120 -0.94024658 -0.35187724
C 1.41966620 0.41882909 -0.37239038
C 0.69374166 -1.95352198 -0.43248824
H 0.44023886 3.98210941 -0.44294126
H 6.16466364 0.08027284 0.01088699
H 7.52208134 -3.05312698 -0.78918357
H 7.86755249 -1.55926697 0.13010980
H 7.38010843 -3.06242641 0.96614588
H 5.69242227 -4.77023792 0.80611690
H 4.26714502 -5.15623563 -0.19280872
H 5.82788492 -4.75289427 -0.95413254
H 2.67460951 -3.45224642 -0.30621590
H 0.85348100 -2.58130998 -1.32042348
H -0.26984284 -1.44614919 -0.50183241
H 0.72253659 -2.58707152 0.46531660

MN15/cc-pVDZ_r5/r6

N 0.09990097 0.74413306 -0.47927471
C -0.20381877 2.03633694 -0.49687777
O -1.43875808 2.39323574 -0.59510245
N 0.75014731 3.01875811 -0.41416951
C 2.09089152 2.75381239 -0.30714777
O 2.92870077 3.67799390 -0.23238160
C 2.47285175 1.34037092 -0.28475070
N 3.75426365 1.02727727 -0.18434757
C 4.05709538 -0.31526059 -0.16839237
C 5.40250140 -0.71407483 -0.06427553
C 5.78452811 -2.05160643 -0.04263611
C 7.23264388 -2.44221364 0.07030556
C 4.78011812 -3.04457933 -0.12889306
C 5.15575451 -4.49998686 -0.10923722
C 3.43760061 -2.66647570 -0.23246581
C 3.05900338 -1.31827601 -0.25345315
N 1.71786787 -0.92818703 -0.35668222
C 1.39085493 0.40756962 -0.37669180
C 0.68259833 -1.94794155 -0.44188723
H 0.45833129 3.99515605 -0.43229899
H 6.14955331 0.08070632 0.00012193
H 7.55241172 -3.04861521 -0.79217984
H 7.87852810 -1.55596238 0.12477772
H 7.41305289 -3.05517221 0.96790997
H 5.69588288 -4.75900047 0.81558926
H 4.26929692 -5.14364672 -0.18160335
H 5.83126250 -4.74770215 -0.94381870
H 2.68267365 -3.45042841 -0.29787054
H 0.84347893 -2.57553807 -1.33027751
H -0.28755887 -1.45185076 -0.51322406
H 0.70733776 -2.58577831 0.45342091

MN15/aug-cc-pVDZ_S0

N 0.10256401 0.72180005 -0.48280503
C -0.23684602 2.04384915 -0.50479304
O -1.39772310 2.44000417 -0.60167904
N 0.78032606 3.01019022 -0.41159803
C 2.12702115 2.77810620 -0.30053002
O 2.94963321 3.68024026 -0.22439702
C 2.47246918 1.33001610 -0.28364502
N 3.72593627 0.99436307 -0.18483201
C 4.04543429 -0.33066102 -0.16818501
C 5.40292040 -0.71078405 -0.06381300
C 5.78200139 -2.03751414 -0.04296100
C 7.22664354 -2.43600818 0.06846301
C 4.76551234 -3.03729622 -0.12989301
C 5.15585237 -4.48613932 -0.10972801
C 3.42615525 -2.68134620 -0.23263402
C 3.04428322 -1.32865409 -0.25365702
N 1.72059212 -0.93683707 -0.35588603
C 1.37443510 0.38419803 -0.37901503
C 0.68147805 -1.96598114 -0.43973703
H 0.47423903 3.98098429 -0.43057803
H 6.14253943 0.08817901 -0.00044300
H 7.53732255 -3.04004022 -0.79511106
H 7.86980154 -1.55112811 0.12189501
H 7.39946552 -3.04622622 0.96567907
H 5.69930544 -4.72763534 0.81428806
H 4.27706231 -5.13514437 -0.18056501
H 5.83257142 -4.71614734 -0.94440807
H 2.67865819 -3.46888225 -0.29768502
H 0.85455106 -2.58236818 -1.32814609
H -0.28448102 -1.46956710 -0.51065404
H 0.71927505 -2.59051819 0.45904103

MN15/aug-cc-pVDZ_S1(r1)/r5

N 0.14114378 0.71580113 -0.47459198
C -0.24694366 2.04720513 -0.50271005
O -1.43236586 2.36389661 -0.59666002
N 0.73930159 3.00826327 -0.42096857
C 2.10627378 2.77687012 -0.31105938
O 2.89582128 3.72211480 -0.24537987
C 2.48147606 1.36967974 -0.28304543
N 3.78478587 1.04466091 -0.17736749
C 4.07221693 -0.28306192 -0.16300939
C 5.40446438 -0.69963006 -0.05774705
C 5.77361309 -2.05005450 -0.04095072
C 7.21062613 -2.43989807 0.07290658
C 4.76562441 -3.06257762 -0.13380459
C 5.15009648 -4.51161212 -0.11896627
C 3.43678069 -2.67773465 -0.23653179
C 3.05955014 -1.31548648 -0.25275489
N 1.74570083 -0.93881317 -0.35139300
C 1.42286647 0.41848258 -0.37214597
C 0.70035403 -1.95688569 -0.43200585
H 0.43919201 3.98034819 -0.44367903
H 6.16756190 0.07594882 0.01158182
H 7.51454438 -3.05246262 -0.78879795
H 7.85978554 -1.56087185 0.13066220
H 7.37217781 -3.06241889 0.96549166
H 5.68781657 -4.76787376 0.80508112
H 4.26472625 -5.15157256 -0.19381028
H 5.82350159 -4.74993019 -0.95473125
H 2.67273284 -3.44927373 -0.30664977
H 0.86452320 -2.57746334 -1.32069571
H -0.26391563 -1.45777907 -0.50013669
H 0.73496652 -2.58481925 0.46605707

MN15/aug-cc-pVDZ_r4

N 0.09078667 0.73547385 -0.49253026
C -0.26524168 2.04592387 -0.51502867
O -1.43779348 2.43665948 -0.61714758
N 0.74615852 3.00458034 -0.41605322
C 2.10055802 2.76391225 -0.30038201
O 2.91294812 3.69250149 -0.21813698
C 2.45175249 1.34803175 -0.28845212
N 3.77302023 1.01903625 -0.18184238
C 4.03624789 -0.29923526 -0.16815613
C 5.41149059 -0.73473169 -0.06231616
C 5.81206660 -2.07730389 -0.04072695
C 7.25793141 -2.45423375 0.07452689
C 4.81508944 -3.05442673 -0.12952664
C 5.15302514 -4.50504160 -0.11517866
C 3.44236630 -2.64444595 -0.23909991
C 3.04404764 -1.29628703 -0.25143590
N 1.71221733 -0.92956023 -0.35625237
C 1.37908792 0.42040443 -0.38512905
C 0.67770585 -1.95537690 -0.42709117
H 0.45119513 3.97798512 -0.43291971
H 6.15859536 0.05846558 0.00828990
H 7.56960029 -3.06267501 -0.78559101
H 7.89443189 -1.56541912 0.12422937
H 7.42865399 -3.06005040 0.97526349
H 5.69965464 -4.76277862 0.80521407
H 4.26054957 -5.13319379 -0.18736305
H 5.83054912 -4.74885030 -0.94841591
H 2.69345537 -3.43055601 -0.32179735
H 0.82614477 -2.57858451 -1.31729842
H -0.28714117 -1.45449218 -0.48941246
H 0.70984539 -2.58267965 0.47194602

mPW1PW91/cc-pVDZ_S0

N 0.11450081 0.70798580 -0.48299108
C -0.22964715 2.03166883 -0.50504877
O -1.38410418 2.41613278 -0.60189100
N 0.78813627 2.99334289 -0.41121240
C 2.13377291 2.76809026 -0.30077299
O 2.95145905 3.66629096 -0.22514174
C 2.47841826 1.32302260 -0.28386870
N 3.73259678 0.99357226 -0.18489016
C 4.04345318 -0.32917401 -0.16877090
C 5.39571772 -0.71092250 -0.06486121
C 5.77732505 -2.03554928 -0.04342708
C 7.21946525 -2.42238159 0.06815275
C 4.76606614 -3.03561812 -0.12949674
C 5.14728316 -4.48124881 -0.10963549
C 3.42900170 -2.67765539 -0.23200042
C 3.04444137 -1.33040648 -0.25365592
N 1.72637119 -0.94275625 -0.35540989
C 1.38075287 0.38131579 -0.37918413
C 0.68206895 -1.95614063 -0.43905172
H 0.48308601 3.96149970 -0.42998075
H 6.13256481 0.08958226 -0.00198790
H 7.53766180 -3.02773845 -0.79343748
H 7.86186267 -1.53581942 0.12000936
H 7.40009101 -3.03096466 0.96661900
H 5.69111494 -4.72990721 0.81377461
H 4.26754960 -5.13053102 -0.17981522
H 5.82321848 -4.71905872 -0.94448036
H 2.68177917 -3.46427764 -0.29650717
H 0.84174587 -2.57781513 -1.32793896
H -0.27443115 -1.43720067 -0.51024124
H 0.70567416 -2.58428451 0.45932949

mPW1PW91/cc-pVDZ_S1(r1)

N 0.14559439 0.69975371 -0.47424020
C -0.24050150 2.03190056 -0.50251855
O -1.42115548 2.33732990 -0.59667959
N 0.74337951 2.98970514 -0.42120020
C 2.11287874 2.76744963 -0.31124251
O 2.89269051 3.71085654 -0.24671563
C 2.48537587 1.36347577 -0.28336075
N 3.79200391 1.04237523 -0.17745258
C 4.06943817 -0.28324239 -0.16380175
C 5.39842361 -0.70255818 -0.05903625
C 5.77078797 -2.05205147 -0.04149845
C 7.20600091 -2.42967323 0.07300204
C 4.76797873 -3.05915587 -0.13346429
C 5.13902695 -4.50681210 -0.11929803
C 3.44034580 -2.66953637 -0.23605600
C 3.06472979 -1.31269875 -0.25243084
N 1.75027936 -0.93512685 -0.35115578
C 1.43192204 0.41169672 -0.37168483
C 0.70336151 -1.94187827 -0.43117455
H 0.44129490 3.95800709 -0.44424122
H 6.15894096 0.07463746 0.00976380
H 7.51901497 -3.04373214 -0.78599455

H 7.85367783 -1.54831890 0.12895293
H 7.37646753 -3.04989596 0.96717443
H 5.67563661 -4.77276897 0.80422747
H 4.25055362 -5.14425022 -0.19376382
H 5.81039593 -4.75531755 -0.95551590
H 2.67636039 -3.44007940 -0.30580298
H 0.86043061 -2.56808693 -1.31868919
H -0.25470767 -1.42719712 -0.50127785
H 0.72837016 -2.57175341 0.46736121

mPW1PW91/cc-pVDZ_r6

N 0.11396699 0.71620285 -0.59354132
C -0.21609173 2.00129876 -0.60419490
O -1.32147521 2.46150252 -0.97156260
N 0.72362860 2.95857077 -0.20493080
C 2.08047761 2.71753656 -0.23888471
O 2.85508907 3.69177288 -0.22104167
C 2.46719612 1.33226088 -0.26839554
N 3.78171985 1.02427462 -0.18082000
C 4.06609028 -0.29755660 -0.17137702
C 5.41288042 -0.71341095 -0.08632896
C 5.78424114 -2.04827035 -0.06001420
C 7.22814143 -2.43744171 0.03358229
C 4.77660820 -3.04046200 -0.12253041
C 5.13747710 -4.49326841 -0.09714011
C 3.43825053 -2.65420207 -0.20994458
C 3.06518781 -1.30954422 -0.23462119
N 1.72749736 -0.92320122 -0.32502343
C 1.40659894 0.40620583 -0.38407233
C 0.69311355 -1.93834204 -0.40158928
H 0.42798829 3.93360032 -0.23128134
H 6.16629388 0.07335028 -0.03995227
H 7.53389739 -3.05040699 -0.82775582
H 7.87555777 -1.55334766 0.07122968
H 7.42091191 -3.04230119 0.93267005
H 5.68618089 -4.75328750 0.82111112
H 4.24483009 -5.12766948 -0.15182040
H 5.79622049 -4.75658303 -0.93896135
H 2.67899313 -3.43156273 -0.25627738
H 0.85056796 -2.57202102 -1.28461856
H -0.27110246 -1.43501339 -0.47888235
H 0.71805918 -2.56563005 0.49915518

mPW1PW91/cc-pVDZ_r7

N 0.09794217 0.71199533 -0.47715806
C -0.24702481 2.02382550 -0.50917016
O -1.43000080 2.37924180 -0.60819350
N 0.74871731 2.98106083 -0.42803036
C 2.11367786 2.75765241 -0.31249139
O 2.89805312 3.69925151 -0.24644166
C 2.48290344 1.35155452 -0.28198754
N 3.78501161 1.03401249 -0.17195964
C 4.07470519 -0.29419943 -0.15778921
C 5.41067888 -0.69847529 -0.05502411
C 5.79285916 -2.05257138 -0.03958296
C 7.22024044 -2.42691671 0.07855926
C 4.77469360 -3.06316715 -0.13729208
C 5.12921488 -4.50382197 -0.12897782
C 3.43457618 -2.65952507 -0.23998447
C 3.08670892 -1.31866354 -0.24767541
N 1.71661957 -0.91514094 -0.34939423
C 1.41444416 0.40120446 -0.37222526
C 0.69663825 -1.93500943 -0.42225570
H 0.44886793 3.94951460 -0.45371267
H 6.16677771 0.08264676 0.01315373
H 7.53877593 -3.05352543 -0.77234752
H 7.86951436 -1.54727273 0.13234008
H 7.39324335 -3.05062242 0.97300748
H 5.67895053 -4.77660985 0.78779817
H 4.23946782 -5.13828394 -0.20181490
H 5.80546257 -4.75453049 -0.96411499
H 2.67268471 -3.43094458 -0.31391072
H 0.86182694 -2.56390702 -1.30952093
H -0.27394169 -1.44258557 -0.48388211
H 0.74670736 -2.57313363 0.47226450

MPWLYP/cc-pVDZ_S0

N -2.58149092 -1.32108677 -0.00003766
C -3.78795072 -0.63958368 -0.00004748
O -4.88620793 -1.21114431 -0.00038781
N -3.75992591 0.78607750 0.00023516
C -2.63931250 1.60903885 0.00010749
O -2.71530652 2.84216908 -0.00017229
C -1.35257936 0.83591320 0.00003189
N -0.22121547 1.51732441 0.00008397
C 0.95348572 0.80520440 0.00008191
C 2.18585988 1.51519084 0.00010402
C 3.41614532 0.86068365 0.00001461
C 4.71307626 1.64074747 -0.00002530
C 3.42833876 -0.57859410 -0.00009460
C 4.74451244 -1.32158240 -0.00020542
C 2.22337996 -1.29780898 -0.00006193
C 0.97560673 -0.63136828 0.00003497
N -0.23924368 -1.31596451 0.00008605
C -1.45014009 -0.62453961 0.00000796
C -0.24813300 -2.79498325 0.00027948
H -4.67533083 1.24975056 0.00016158
H 2.12599699 2.61167042 0.00018330
H 5.33151730 1.39760923 -0.88899130
H 4.52396945 2.72969694 0.00085338
H 5.33244950 1.39628722 0.88790602
H 5.35403607 -1.05239850 0.88755498
H 4.59494646 -2.41626657 -0.00112534
H 5.35483729 -1.05094396 -0.88694413
H 2.26719205 -2.39160007 -0.00014537
H 0.27002371 -3.16903587 -0.90207638
H -1.29819884 -3.11858044 0.00043820
H 0.27007187 -3.16875021 0.90272775

MPWLYP/cc-pVDZ_S1(r1)

N -2.55981435 -1.31585130 0.00055485
C -3.79570032 -0.64968614 0.00050232
O -4.85406043 -1.30628009 0.00107617
N -3.79434158 0.74022531 0.00071604
C -2.65188401 1.58937928 0.00066232
O -2.80004807 2.82420872 -0.00030309
C -1.37963993 0.87106367 -0.00040344
N -0.21070453 1.59579614 -0.00049814
C 0.94259515 0.85558100 -0.00046637
C 2.19014831 1.52470436 -0.00022668
C 3.42881907 0.84545991 0.00000599
C 4.71902753 1.62354511 0.00008861
C 3.44479150 -0.58904606 0.00008407
C 4.75168553 -1.34842520 0.00029031
C 2.21993037 -1.27834796 -0.00000017
C 0.97862521 -0.59290517 -0.00033279
N -0.24079588 -1.28258756 -0.00056013
C -1.42728071 -0.56785588 -0.00053643
C -0.24737400 -2.76185164 -0.00053265
H -4.70707290 1.20644270 0.00083433
H 2.16274139 2.62246912 -0.00028514
H 5.33950195 1.36816551 -0.88550339
H 4.53984581 2.71348486 0.00020591
H 5.33953169 1.36795168 0.88558855
H 5.36822622 -1.09552151 0.88868456
H 4.58237232 -2.44073597 0.00011379
H 5.36867898 -1.09528660 -0.88771713
H 2.24065499 -2.37312245 0.00009594
H 0.27725744 -3.12993062 -0.90208507
H -1.29205855 -3.09802138 -0.00080020
H 0.27675480 -3.12989030 0.90132604

MPWLYP/cc-pVDZ_r7

N -2.59055247 -1.32545256 -0.00220099
C -3.80105393 -0.65694574 0.00190726
O -4.89225274 -1.26526694 0.00783290
N -3.77936732 0.75174471 -0.00101151
C -2.64634698 1.59108267 -0.00943077
O -2.77175252 2.82942052 -0.01329512
C -1.36649406 0.86205779 -0.01255304
N -0.22164664 1.58618714 -0.02371821
C 0.95686064 0.84105417 -0.01759466
C 2.18468175 1.53175690 -0.02029155
C 3.44521276 0.86248551 -0.01026009
C 4.71747403 1.64494764 -0.03069412
C 3.45738426 -0.59100288 0.01179308
C 4.74545045 -1.35274081 0.03980448
C 2.21437416 -1.28363764 0.01192639
C 0.99578627 -0.59304260 -0.00495354
N -0.26298074 -1.30887309 -0.00685818
C -1.44392826 -0.59646523 -0.00609216
C -0.25242402 -2.77391047 -0.01084718
H -4.69092898 1.22122457 0.00102520
H 2.14650932 2.62858658 -0.03149507
H 5.33021859 1.39919730 -0.92972690
H 4.53493455 2.73333774 -0.01775963
H 5.37565428 1.37994079 0.82838405
H 5.34361403 -1.10554351 0.94747438
H 4.57893800 -2.44410341 0.02103201
H 5.40423678 -1.07880822 -0.81503661
H 2.23884124 -2.37757968 0.02796073
H 0.28793076 -3.14527639 -0.90536789
H -1.29537560 -3.11925071 -0.02258572
H 0.26741243 -3.15199463 0.89321549

MPWLYP/cc-pVDZ_r8

N -2.60073029 -1.29255090 -0.00013638
C -3.84049450 -0.68438464 -0.00047354
O -4.91232005 -1.30644362 0.00009027
N -3.80495148 0.72905095 0.00003404
C -2.66704689 1.57238793 0.00016143
O -2.76993158 2.81351099 0.00059715
C -1.41159191 0.83347532 0.00009323
N -0.20805431 1.48484763 0.00013936
C 0.97790384 0.87352709 0.00008957
C 2.23470907 1.55482828 0.00004839
C 3.45014830 0.86534000 -0.00004684
C 4.75769683 1.63184718 -0.00015199
C 3.45562683 -0.56591539 0.00004091
C 4.75496680 -1.33837818 -0.00000069
C 2.21872017 -1.25253192 0.00011068
C 0.98796693 -0.59057915 0.00007426
N -0.24938319 -1.28023705 0.00005853
C -1.44682151 -0.58829877 0.00004181
C -0.26541105 -2.75593643 -0.00000960
H -4.71844005 1.19514798 0.00027444
H 2.20738767 2.65174571 0.00002032
H 5.37186394 1.37694950 -0.88883612
H 4.58596670 2.72359908 0.00009941
H 5.37224574 1.37657870 0.88815347
H 5.37552479 -1.09273168 0.88796350
H 4.57638682 -2.42943630 -0.00034338
H 5.37580420 -1.09219649 -0.88761013
H 2.22722120 -2.35098488 0.00018137
H 0.24563131 -3.13928751 -0.90402651
H -1.31588391 -3.09045115 0.00000746
H 0.24570260 -3.13936075 0.90393458

MPWLYP/cc-pVDZ_T1

N -2.55912900 -1.30770700 0.00036400
C -3.79706400 -0.64876500 -0.00013000
O -4.86311700 -1.28761900 -0.00111300
N -3.78740100 0.74918700 0.00059300
C -2.65327000 1.58593000 0.00028100
O -2.77555900 2.82190000 0.00021500
C -1.37238300 0.85575200 0.00008100
N -0.21475900 1.58529900 0.00007100
C 0.94103900 0.84978700 0.00005700
C 2.18486100 1.52435900 -0.00015700
C 3.42163600 0.85079900 -0.00016000
C 4.70984600 1.63114200 -0.00066900
C 3.44638800 -0.59176600 0.00009200
C 4.75963100 -1.33858900 0.00038900
C 2.23066800 -1.28813400 0.00024000
C 0.98289800 -0.61261300 0.00020600
N -0.22007800 -1.30727100 0.00011000
C -1.44184500 -0.57345900 0.00004400
C -0.23611700 -2.78099400 -0.00015800
H -4.70033500 1.21685900 0.00031800
H 2.15241200 2.62166900 -0.00030000
H 5.32806200 1.37764900 -0.88808200
H 4.52805700 2.72047600 0.00169100
H 5.33137000 1.37405500 0.88329900
H 5.37241700 -1.08120500 0.88996300
H 4.59971100 -2.43217200 -0.00202800
H 5.37515600 -1.07750300 -0.88612900
H 2.25604000 -2.38275400 0.00041300
H 0.28174400 -3.16039800 -0.90182500
H -1.28484200 -3.10745600 -0.00034500
H 0.28145700 -3.16072400 0.90154200

MPWLYP/cc-pVDZ_T6

N -2.57400100 -1.33186000 0.00095800
C -3.78099600 -0.65937600 0.00060500
O -4.87816000 -1.24511100 0.00109600
N -3.77510900 0.76808600 -0.00036600
C -2.64955600 1.60097500 -0.00082500
O -2.76901300 2.83746500 -0.00203300
C -1.36214100 0.86352300 -0.00045000
N -0.23639000 1.55761400 -0.00066900
C 0.94733300 0.84632400 -0.00022400
C 2.16493200 1.53506700 0.00023200
C 3.41866800 0.87950700 0.00042700
C 4.70072300 1.66099700 0.00371600
C 3.44138500 -0.60606900 -0.00093000
C 4.76761500 -1.32422900 -0.00321700
C 2.25332400 -1.31239100 -0.00078400
C 0.97845300 -0.64605100 -0.00010600
N -0.22055900 -1.31724500 0.00046300
C -1.43059100 -0.62619200 0.00036900
C -0.23756800 -2.80044300 0.00113000
H -4.69341800 1.22581600 -0.00077700
H 2.11694100 2.63285000 0.00055900
H 5.33570700 1.41753500 -0.87730900
H 4.50970700 2.74975700 -0.00054500
H 5.32657300 1.42370800 0.89323200
H 5.38003400 -1.04790700 0.88164600
H 4.63259600 -2.42135700 -0.00146400
H 5.37491500 -1.05059900 -0.89259000
H 2.29341900 -2.40735500 -0.00149600
H 0.28008100 -3.17274500 -0.90140700
H -1.28742200 -3.12267700 0.00188100
H 0.28118100 -3.17186200 0.90339600

MPWLYP/cc-pVDZ_T7

N -2.58733900 -1.32269500 0.01499100
C -3.79521500 -0.65134700 0.00322300
O -4.88880600 -1.26348500 0.00586200
N -3.77673200 0.75498000 -0.01146500
C -2.64397200 1.59330800 -0.01030500
O -2.76681500 2.83058800 -0.01948900
C -1.36899800 0.85424000 0.00065000
N -0.21132700 1.56942700 0.00290200
C 0.95332000 0.84926300 0.00385200
C 2.18686800 1.53628000 0.00719200
C 3.44795900 0.85554000 0.00451900
C 4.72925200 1.63052900 0.02717100
C 3.45158200 -0.60658000 -0.01077000
C 4.74940600 -1.35952500 -0.03050500
C 2.22828300 -1.30379900 -0.01067300
C 0.98412200 -0.60997800 0.00002200
N -0.23970100 -1.29697800 0.00624900
C -1.44055200 -0.58009300 0.00696500
C -0.25092100 -2.77151900 0.01162900
H -4.68939300 1.22276000 -0.01918800
H 2.15284300 2.63298300 0.01192200
H 5.38749900 1.36585400 -0.83080500
H 4.54715200 2.71968400 0.00599900
H 5.33309500 1.39626600 0.93423300
H 5.39033500 -1.08587400 0.83645600
H 4.59056800 -2.45234100 -0.02000700
H 5.35116700 -1.10001700 -0.93020100
H 2.25474800 -2.39748700 -0.02327600
H 0.26084900 -3.14847800 -0.89457400
H -1.29879600 -3.09980100 0.02477100
H 0.28379900 -3.14142500 0.90712800

O3LYP/cc-pVDZ_S0

N 0.10526960 0.71701139 -0.48367057
C -0.24019732 2.04481501 -0.50579447
O -1.39968756 2.43027991 -0.60323675
N 0.78413412 3.00752396 -0.41115500
C 2.13527166 2.78007696 -0.30021852
O 2.95557169 3.68305256 -0.22390490
C 2.47710303 1.32839361 -0.28392134
N 3.73689542 0.99579166 -0.18475646
C 4.04295319 -0.33083853 -0.16925282
C 5.39845960 -0.71448692 -0.06506407
C 5.78901982 -2.04159714 -0.04286822
C 7.24038929 -2.41253420 0.07004696
C 4.77602446 -3.04711237 -0.12927880
C 5.14769788 -4.50076963 -0.11043689
C 3.43431729 -2.68602479 -0.23196204
C 3.04077105 -1.33751217 -0.25432167
N 1.71790542 -0.94746492 -0.35610248
C 1.37489190 0.38732718 -0.37955367
C 0.66471279 -1.95934454 -0.43990775
H 0.48249225 3.97737248 -0.42937883
H 6.13290756 0.08909129 -0.00222713
H 7.56990626 -3.01500546 -0.79045907
H 7.87387409 -1.51859254 0.12131744
H 7.43155383 -3.01788581 0.96965194
H 5.68926663 -4.75862968 0.81283491
H 4.26340611 -5.14509952 -0.18124076
H 5.82166847 -4.74737296 -0.94550501
H 2.69101108 -3.47599614 -0.29576317
H 0.81991288 -2.58399312 -1.32873360
H -0.29107740 -1.43904941 -0.51219217
H 0.68257155 -2.58837249 0.45924069

O3LYP/cc-pVDZ_S1(r1)

N 0.13094776 0.70697004 -0.47490427
C -0.25221789 2.04461125 -0.50253043
O -1.44029405 2.34812577 -0.59557996
N 0.73545761 3.00274402 -0.42229760
C 2.11424384 2.77814659 -0.31247368
O 2.89338524 3.72901193 -0.24903435
C 2.48384695 1.37053550 -0.28398976
N 3.79597659 1.04419050 -0.17738791
C 4.06834333 -0.28727420 -0.16415904
C 5.40192608 -0.70838049 -0.05858931
C 5.78413313 -2.06088156 -0.04041924
C 7.22899748 -2.42332016 0.07637010
C 4.78000733 -3.06956556 -0.13363855
C 5.13815147 -4.52622044 -0.12163940
C 3.44682652 -2.67474180 -0.23658419
C 3.06530494 -1.31644271 -0.25279549
N 1.74099622 -0.93290288 -0.35170782
C 1.42738380 0.41447947 -0.37188981
C 0.68671579 -1.94123931 -0.43105342
H 0.43642778 3.97238666 -0.44511760
H 6.15998810 0.07220509 0.01072323
H 7.55487218 -3.03464686 -0.78085792
H 7.86795657 -1.53454457 0.13174953
H 7.41058534 -3.03969998 0.97210033
H 5.67135699 -4.80274271 0.80179274
H 4.24407952 -5.15699174 -0.19775910
H 5.80741444 -4.78353051 -0.95805310
H 2.68685260 -3.44881231 -0.30639364
H 0.84158237 -2.56921171 -1.31858608
H -0.27205525 -1.42796088 -0.50145409

H 0.70980389 -2.57124277 0.46834566

O3LYP/cc-pVDZ_r7

N 0.09225678 0.72051295 -0.48020696
C -0.26044397 2.04013976 -0.52064657
O -1.44294459 2.39522669 -0.62624972
N 0.74748802 2.99577096 -0.43806967
C 2.11771781 2.77091186 -0.31472909
O 2.90604715 3.71526458 -0.24620829
C 2.48393557 1.35702326 -0.28039675
N 3.78571993 1.03921003 -0.16272396
C 4.07140231 -0.30176984 -0.15079301
C 5.41054897 -0.70174347 -0.05051164
C 5.80721219 -2.05949242 -0.03840503
C 7.24199380 -2.41563190 0.08797963
C 4.78631593 -3.07569974 -0.14403440
C 5.13065064 -4.52234475 -0.14483188
C 3.43974595 -2.66870496 -0.24589772
C 3.08238242 -1.32564856 -0.24451526
N 1.70631876 -0.92254640 -0.34347269
C 1.40326177 0.40508993 -0.37255207
C 0.67596463 -1.93978632 -0.40652760
H 0.45239564 3.96646264 -0.46610912
H 6.16226928 0.08436676 0.01869965
H 7.57734883 -3.04677318 -0.75472739
H 7.88213164 -1.52829488 0.13922765
H 7.42450307 -3.03263975 0.98715021
H 5.68570685 -4.80813949 0.76624518
H 4.23731692 -5.15289994 -0.21716450
H 5.80318690 -4.77674322 -0.98379093
H 2.68311069 -3.44407672 -0.32663187
H 0.82191975 -2.57082686 -1.29689807
H -0.29316072 -1.44267251 -0.45815160
H 0.72669378 -2.58049085 0.48712926

O3LYP/cc-pVDZ_T1

N 0.13392000 0.71635000 -0.47466700
C -0.24931900 2.05167600 -0.50184100
O -1.43330600 2.36739600 -0.59580000
N 0.74537100 3.00858900 -0.41929600
C 2.11462600 2.77909700 -0.31094200
O 2.90734600 3.71555000 -0.24609200
C 2.48104700 1.36058200 -0.28466400
N 3.79437900 1.04189200 -0.17996900
C 4.06672900 -0.28760900 -0.16639500
C 5.40398700 -0.70677600 -0.06118700
C 5.78308900 -2.05083800 -0.04192500
C 7.22801300 -2.41797900 0.07412800
C 4.77565300 -3.06838700 -0.13322000
C 5.14302200 -4.52232600 -0.11870200
C 3.44465900 -2.68232800 -0.23564400
C 3.05803100 -1.32685100 -0.25396800
N 1.73767900 -0.95168500 -0.35208700
C 1.41964200 0.42237200 -0.37256200
C 0.68095600 -1.95309600 -0.43177700
H 0.44836000 3.97938800 -0.44129300
H 6.15883600 0.07659600 0.00637400
H 7.55049200 -3.02989600 -0.78323300
H 7.86808100 -1.52989800 0.12747600
H 7.40755000 -3.03172600 0.97139500
H 5.67915500 -4.79291600 0.80456700
H 4.25260200 -5.15831000 -0.19275600
H 5.81332800 -4.77627600 -0.95506600
H 2.68702100 -3.45857800 -0.30353400
H 0.82720700 -2.58186600 -1.32047600
H -0.27532200 -1.43405500 -0.50130300
H 0.69615500 -2.58503700 0.46664400

O3LYP/cc-pVDZ_T4

N 0.10970400 0.72188600 -0.55962000
C -0.23034500 2.01116600 -0.56890100
O -1.34119200 2.47637700 -0.92409000
N 0.71605600 2.97108700 -0.18667200
C 2.08828300 2.73067300 -0.23559600
O 2.83980800 3.73943200 -0.23485500
C 2.47376600 1.35089700 -0.26783100
N 3.78436300 1.03010100 -0.21150100
C 4.06963500 -0.30314200 -0.18954700
C 5.41351500 -0.71617700 -0.10960800
C 5.79442700 -2.05924100 -0.06681300
C 7.24740200 -2.42898200 0.02027400
C 4.78556700 -3.05663000 -0.10489200
C 5.13537400 -4.51779000 -0.05791200
C 3.44373600 -2.66501000 -0.18903400
C 3.06631100 -1.31701600 -0.23460100
N 1.72436200 -0.92525100 -0.34041900
C 1.39592900 0.40998000 -0.37479600
C 0.68471400 -1.94026400 -0.45598900
H 0.41912100 3.94720400 -0.17876900
H 6.16784600 0.07126800 -0.07866200
H 7.55686600 -3.05023800 -0.83532300
H 7.88657600 -1.53761400 0.04132900
H 7.45862900 -3.02145900 0.92476600
H 5.68375000 -4.77369000 0.86277500
H 4.23654700 -5.14590900 -0.09934600
H 5.78715500 -4.80548800 -0.89829100
H 2.68580700 -3.44400300 -0.21855300
H 0.87019800 -2.57344300 -1.33463400
H -0.27583000 -1.43562200 -0.56508000
H 0.67091000 -2.57003900 0.44437400

OLYP/cc-pVDZ_S0

N 0.09697314 0.71316578 -0.48331420
C -0.25330606 2.04360385 -0.50556389
O -1.41934145 2.42965628 -0.60248141
N 0.77306985 3.00882810 -0.41187862
C 2.12928296 2.78570817 -0.30079569
O 2.94996365 3.69726502 -0.22469547
C 2.47365157 1.33604050 -0.28439647
N 3.74256148 1.00591916 -0.18493245
C 4.04927730 -0.32407322 -0.16927008
C 5.40556042 -0.71174851 -0.06518432
C 5.78783949 -2.04375386 -0.04334173
C 7.23072852 -2.43260439 0.06856015
C 4.77364935 -3.04734992 -0.12927977
C 5.15576794 -4.49423239 -0.10863536
C 3.43007722 -2.68618887 -0.23229113
C 3.04478271 -1.33471860 -0.25427619
N 1.72294898 -0.94403587 -0.35598556
C 1.37405325 0.39376301 -0.37943532
C 0.67496963 -1.95905469 -0.43984166
H 0.46945075 3.98041417 -0.43009789
H 6.14633660 0.08805767 -0.00222579
H 7.54363642 -3.04456321 -0.79373015
H 7.87673286 -1.54560012 0.12080877
H 7.40562266 -3.04762837 0.96707471
H 5.70523159 -4.73821209 0.81589313
H 4.27500727 -5.14632628 -0.17915502
H 5.83834734 -4.72807056 -0.94273314
H 2.68190905 -3.47332832 -0.29639706
H 0.84174270 -2.58321114 -1.32894978
H -0.28192192 -1.43598386 -0.51183994
H 0.70439864 -2.58868602 0.46057661

OLYP/cc-pVDZ_S1(r2)

N 0.11625211 0.70195020 -0.47149310
C -0.26433989 2.04240024 -0.50290169
O -1.46183113 2.34314485 -0.59969329
N 0.72213347 3.00355700 -0.42365686
C 2.10914154 2.78304038 -0.31293931
O 2.88555698 3.74425707 -0.25169540
C 2.48062610 1.37887271 -0.28318901
N 3.80152051 1.05171518 -0.17577182
C 4.07388479 -0.28312042 -0.16219082
C 5.41156838 -0.70823000 -0.05705191
C 5.78410159 -2.06298038 -0.04021673
C 7.22134742 -2.44596728 0.07398782
C 4.77796731 -3.06719229 -0.13298698
C 5.14456374 -4.51807137 -0.12010101
C 3.44182806 -2.67169655 -0.23586770
C 3.07224122 -1.31181859 -0.25070458
N 1.74376022 -0.92258511 -0.35012025
C 1.42765473 0.41685661 -0.37000608
C 0.69703452 -1.93831879 -0.43113068
H 0.42094865 3.97448903 -0.44900458
H 6.17461027 0.06983466 0.01201566
H 7.52991105 -3.06460293 -0.78663600
H 7.87365644 -1.56478719 0.13169019
H 7.38723846 -3.07341872 0.96713690
H 5.68546375 -4.78253264 0.80453199
H 4.25296050 -5.15543814 -0.19617783
H 5.82185707 -4.76358176 -0.95593653
H 2.67782381 -3.44356137 -0.30607665
H 0.86688917 -2.56176597 -1.32079246
H -0.26518582 -1.42704780 -0.49861495
H 0.73781637 -2.57034586 0.46778017

OLYP/cc-pVDZ_r7

N 0.08884375 0.71629511 -0.48292367
C -0.27257672 2.04088642 -0.51882617
O -1.45881487 2.39631621 -0.62287758
N 0.73892625 2.99964845 -0.43299053
C 2.11343575 2.77914344 -0.31136425
O 2.90448974 3.72887352 -0.24057619
C 2.48238065 1.36362856 -0.28063419
N 3.78798547 1.05181705 -0.16437763
C 4.07335878 -0.30148608 -0.15326328
C 5.41402540 -0.70004251 -0.05436763
C 5.80674826 -2.06259458 -0.04091520
C 7.23451277 -2.43127643 0.08131638
C 4.78488426 -3.07560417 -0.14143728
C 5.13632207 -4.51598255 -0.13464937
C 3.43432280 -2.66980998 -0.24387481
C 3.08295057 -1.32216960 -0.24532553
N 1.71147809 -0.92274425 -0.34577174
C 1.39820035 0.40940480 -0.37454931
C 0.68545593 -1.94196670 -0.41311339
H 0.44167699 3.97226379 -0.45788268
H 6.16998415 0.08436747 0.01283357
H 7.55600563 -3.07044110 -0.76401056
H 7.88462681 -1.54926211 0.13481385
H 7.40761057 -3.06228592 0.97546189
H 5.69802843 -4.78675829 0.77976343
H 4.24644519 -5.15412429 -0.20754794
H 5.82246091 -4.76300791 -0.96765871
H 2.67456012 -3.44408058 -0.32261786
H 0.84380547 -2.57325441 -1.30325045
H -0.28330552 -1.43938554 -0.46907937
H 0.74017335 -2.58331301 0.48188268

OLYP/cc-pVDZ_r8

N 0.10685640 0.74585424 -0.47799072
C -0.32024395 2.04833001 -0.50583415
O -1.50432143 2.37895895 -0.59970560
N 0.70368230 2.99938737 -0.41793371
C 2.08582727 2.77969729 -0.30813593
O 2.89306845 3.71656793 -0.23746859
C 2.43539093 1.37549224 -0.28761048
N 3.73039031 0.97577465 -0.18585832
C 4.11315854 -0.29439385 -0.16355801
C 5.46438339 -0.72022694 -0.05970694
C 5.81345135 -2.06472758 -0.04084793
C 7.25431818 -2.46946330 0.07163519
C 4.80128047 -3.05858133 -0.12761517
C 5.15109558 -4.51374505 -0.10925875
C 3.46049671 -2.65001121 -0.23117746
C 3.08220454 -1.31382220 -0.25208648
N 1.74223775 -0.91260145 -0.35513149
C 1.40321818 0.41562833 -0.37578800
C 0.69029233 -1.91973454 -0.44044260
H 0.40138960 3.97074394 -0.43729079
H 6.22564063 0.05902745 0.00499565
H 7.55659799 -3.08547595 -0.79159802
H 7.91341035 -1.59232392 0.12532552
H 7.41771790 -3.08866341 0.96947145
H 5.69014294 -4.78236593 0.81558439
H 4.25284802 -5.14246826 -0.18176733
H 5.82492127 -4.77221539 -0.94432314
H 2.68291695 -3.41371674 -0.29810945
H 0.84536366 -2.54529024 -1.33221096
H -0.27369015 -1.40218487 -0.51453476
H 0.70495494 -2.55039548 0.46115906

OLYP/cc-pVDZ_T1

N 0.12436100 0.71257800 -0.47627300
C -0.26172500 2.04971000 -0.50225300
O -1.45278200 2.36801300 -0.59506000
N 0.73534900 3.00931300 -0.42006900
C 2.10952800 2.78527200 -0.31184700
O 2.90281400 3.73027900 -0.24751900
C 2.47842200 1.36790200 -0.28487800
N 3.79599500 1.05040100 -0.18003700
C 4.07169200 -0.28299800 -0.16632700
C 5.41003200 -0.70412500 -0.06079200
C 5.78220800 -2.05423900 -0.04210300
C 7.21909500 -2.43728700 0.07245900
C 4.77460700 -3.06819700 -0.13300700
C 5.15121000 -4.51582600 -0.11672400
C 3.44037300 -2.68168700 -0.23613900
C 3.06278600 -1.32358600 -0.25408000
N 1.74176600 -0.94674000 -0.35217000
C 1.41708700 0.42710700 -0.37279800
C 0.69112700 -1.95195300 -0.43104800
H 0.43608000 3.98175900 -0.44189500
H 6.17091900 0.07557100 0.00723400
H 7.52583800 -3.05767400 -0.78712700
H 7.87101100 -1.55587000 0.12801500
H 7.38351600 -3.06240000 0.96713100
H 5.69477200 -4.77373700 0.80803000
H 4.26363300 -5.15881000 -0.19062600
H 5.82978600 -4.75826800 -0.95224300
H 2.67818300 -3.45524800 -0.30450700
H 0.84851400 -2.58038900 -1.32025500
H -0.26659400 -1.43082600 -0.49930900
H 0.71938800 -2.58498900 0.46840100

OLYP/cc-pVDZ_T6

N 0.09884300 0.71487400 -0.48017500
C -0.25206900 2.04217800 -0.50200300
O -1.42624600 2.42048900 -0.59645700
N 0.76435600 3.01954600 -0.41250300
C 2.12918900 2.79420000 -0.30306700
O 2.92857900 3.73254900 -0.23162800
C 2.49205300 1.36475500 -0.28380700
N 3.76307100 1.04224400 -0.18550400
C 4.07175800 -0.29501200 -0.16894500
C 5.40136800 -0.69021500 -0.06743000
C 5.79481800 -2.04102800 -0.04398100
C 7.22749200 -2.42205700 0.06905900
C 4.75194400 -3.08000600 -0.13157400
C 5.15351500 -4.51831400 -0.10988900
C 3.43031900 -2.71728000 -0.23189900
C 3.03148900 -1.34563600 -0.25539400
N 1.73117400 -0.95156000 -0.35514700
C 1.38793600 0.38653300 -0.37749300
C 0.67069000 -1.95914000 -0.43969100
H 0.45824300 3.99009400 -0.43106000
H 6.15348600 0.09984000 -0.00439200
H 7.54972300 -3.04452200 -0.78634900
H 7.87451800 -1.53542900 0.11837600
H 7.41151300 -3.04040800 0.96758600
H 5.70745900 -4.76114700 0.81357200
H 4.27835900 -5.17904100 -0.17814500
H 5.83683000 -4.75129300 -0.94512800
H 2.67622200 -3.50011700 -0.29547100
H 0.83775800 -2.58396200 -1.32741800
H -0.28300000 -1.43179500 -0.51359700
H 0.69760000 -2.58627900 0.46174200

OLYP/cc-pVDZ_T7

N 0.08992100 0.71529500 -0.47278200
C -0.26990100 2.04017500 -0.50017300
O -1.46122700 2.39362600 -0.59163000
N 0.73714500 3.00164400 -0.42210300
C 2.11188200 2.78260200 -0.31338700
O 2.90385200 3.73018100 -0.25025100
C 2.47312200 1.36342100 -0.28655100
N 3.77996300 1.03895200 -0.18384300
C 4.07631500 -0.28973600 -0.16699300
C 5.41657900 -0.69089600 -0.06217600
C 5.80421000 -2.06224000 -0.04084500
C 7.23377100 -2.44007300 0.07814400
C 4.77065700 -3.07693400 -0.13487000
C 5.13764700 -4.51720500 -0.12092200
C 3.43064600 -2.69108600 -0.23828000
C 3.06322700 -1.32248600 -0.25385700
N 1.73495900 -0.92965400 -0.35180700
C 1.40722500 0.41815400 -0.37232100
C 0.68858600 -1.94103500 -0.43029500
H 0.43615100 3.97334600 -0.44508200
H 6.17394900 0.09174800 0.00367100
H 7.55709300 -3.06807200 -0.77367600
H 7.88224600 -1.55577100 0.13088300
H 7.41139500 -3.06403900 0.97498500
H 5.69476800 -4.77335100 0.79883300
H 4.25376200 -5.16390600 -0.19415000
H 5.82666900 -4.75627200 -0.95179000
H 2.67285100 -3.46768000 -0.30866700
H 0.85392500 -2.56690600 -1.31967300
H -0.27112400 -1.42356000 -0.49584100
H 0.72872500 -2.57518700 0.46763300

OPBE/cc-pvVDZ_S0

N 0.10646001 0.70356405 -0.48283503
C -0.24192902 2.02945314 -0.50488504
O -1.40523410 2.41432718 -0.60190004
N 0.77998306 2.99072621 -0.41112203
C 2.13201115 2.77172020 -0.30161002
O 2.95055921 3.68111026 -0.22672302
C 2.47627118 1.32685710 -0.28486502
N 3.74342227 1.00060507 -0.18566601
C 4.04734229 -0.32506902 -0.16993401
C 5.40059139 -0.71173105 -0.06599900
C 5.78343843 -2.04133915 -0.04366800
C 7.22060954 -2.42690217 0.06827001
C 4.77037534 -3.04302922 -0.12935001
C 5.14829237 -4.48434732 -0.10893101
C 3.42930325 -2.68157919 -0.23218702
C 3.04544822 -1.33341210 -0.25429302
N 1.72890712 -0.94485207 -0.35536903
C 1.38094710 0.38770303 -0.37910003
C 0.68422905 -1.95003814 -0.43843403
H 0.47514403 3.96130829 -0.42982803
H 6.14102842 0.08905401 -0.00326800
H 7.53389655 -3.03852022 -0.79321006
H 7.86634258 -1.54037811 0.11949101
H 7.39567053 -3.03933022 0.96766507
H 5.69730139 -4.72857634 0.81499506
H 4.26785331 -5.13596137 -0.17869101
H 5.82909240 -4.71869934 -0.94346107
H 2.68006519 -3.46911625 -0.29633702
H 0.84334606 -2.57251418 -1.32973110
H -0.26967302 -1.41887010 -0.50896604
H 0.70790105 -2.57911019 0.46213003

OPBE/cc-pvVDZ_S1(r2)

N 0.12818350 0.69252571 -0.46969048
C -0.25222827 2.02709510 -0.50274315
O -1.44679263 2.32594340 -0.59868266
N 0.73011927 2.98654960 -0.42485714
C 2.11098456 2.77114030 -0.31400295
O 2.88524965 3.73018376 -0.25355340

C 2.48504526 1.37081449 -0.28358181
N 3.80182219 1.04847212 -0.17634003
C 4.07098139 -0.28316171 -0.16270177
C 5.40501065 -0.70817642 -0.05768635
C 5.77873039 -2.06109498 -0.04049716
C 7.21035091 -2.43874534 0.07406596
C 4.77503069 -3.06422573 -0.13341605
C 5.13725009 -4.50964112 -0.12129445
C 3.44200266 -2.66677456 -0.23604538
C 3.07332747 -1.30987221 -0.25049120
N 1.74985784 -0.92439230 -0.34883352
C 1.43489945 0.41139396 -0.36920056
C 0.70702693 -1.93004909 -0.42875247
H 0.42658390 3.95611641 -0.45081658
H 6.16863671 0.07008534 0.01151881
H 7.51984102 -3.05763482 -0.78532136
H 7.86215895 -1.55795263 0.13146630
H 7.37641361 -3.06514973 0.96723840
H 5.67668554 -4.77531720 0.80296173
H 4.24552843 -5.14584259 -0.19722259
H 5.81240977 -4.75583095 -0.95768438
H 2.67581211 -3.43801915 -0.30673208
H 0.86843322 -2.55204758 -1.32079567
H -0.25260046 -1.41110389 -0.49415700
H 0.74224180 -2.56223452 0.47003478

OPBE/cc-pvVDZ_r7

N 0.10018392 0.70618730 -0.48172989
C -0.26051531 2.02587922 -0.52519330
O -1.44363979 2.37846833 -0.63426878
N 0.74646312 2.98182309 -0.44061982
C 2.11549876 2.76621660 -0.31318853
O 2.90337791 3.71420126 -0.24093538
C 2.48549368 1.35509010 -0.27880367
N 3.78811865 1.04695653 -0.15662004
C 4.07018545 -0.30074909 -0.14723487
C 5.40873881 -0.70057553 -0.05345712
C 5.80116465 -2.06020659 -0.04291715
C 7.22320716 -2.42358582 0.08559418
C 4.78194020 -3.07092243 -0.14725337
C 5.12736163 -4.50660049 -0.14450441
C 3.43467886 -2.66418740 -0.24814621
C 3.08304602 -1.31935601 -0.24162305
N 1.71856487 -0.92351915 -0.33964673
C 1.40589320 0.40419639 -0.37267631
C 0.69564891 -1.93351428 -0.40076438
H 0.44774164 3.95328299 -0.46803571
H 6.16532067 0.08420992 0.01180942
H 7.54860466 -3.06983679 -0.75226485
H 7.87316050 -1.54199971 0.13613002
H 7.39566667 -3.04912672 0.98333307
H 5.69301246 -4.78172213 0.76536604
H 4.23656026 -5.14281361 -0.21432870
H 5.80817545 -4.75445553 -0.98091015
H 2.67366500 -3.43807587 -0.33390797
H 0.84141471 -2.56314945 -1.29409991
H -0.27087727 -1.42347398 -0.45018634
H 0.75114120 -2.57558752 0.49326971

OPBE/cc-pvVDZ_r8

N 0.11951868 0.73240604 -0.47643127
C -0.31018185 2.02899196 -0.50506970
O -1.49216662 2.35505615 -0.59846105
N 0.70829398 2.97770192 -0.41870047
C 2.08647062 2.76646464 -0.30952977
O 2.88784698 3.70434424 -0.24021773
C 2.44173707 1.36782155 -0.28783100
N 3.73370047 0.97452924 -0.18630481
C 4.11355504 -0.29305054 -0.16373007
C 5.46014590 -0.72112391 -0.06014772
C 5.80916136 -2.06334547 -0.04106213
C 7.24444310 -2.46531046 0.07163707
C 4.79856867 -3.05572556 -0.12790770
C 5.14295793 -4.50572561 -0.11023210
C 3.46250409 -2.64231290 -0.23094774
C 3.08599137 -1.30921800 -0.25153095
N 1.75176535 -0.91241767 -0.35379630
C 1.41298125 0.40970991 -0.37487342
C 0.70065022 -1.90770299 -0.43861259
H 0.40249297 3.94736918 -0.43880218
H 6.22200058 0.05831146 0.00448712
H 7.54674878 -3.08059658 -0.79117415
H 7.90340768 -1.58879458 0.12494938
H 7.40726327 -3.08248796 0.97008582
H 5.68044397 -4.77558217 0.81423196
H 4.24418876 -5.13262694 -0.18247687
H 5.81443472 -4.76513818 -0.94588166
H 2.67940641 -3.40272733 -0.29838049
H 0.84455803 -2.53106355 -1.33344732
H -0.25905052 -1.37789503 -0.51149024
H 0.70515845 -2.53680721 0.46383391

OPBE/cc-pvVDZ_T1

N 0.13570500 0.70243000 -0.47502400
C -0.25021100 2.03397900 -0.50057900
O -1.43874700 2.34960200 -0.59118500
N 0.74233600 2.99141100 -0.42099800
C 2.11166900 2.77273100 -0.31385100
O 2.90184200 3.71625400 -0.25112600
C 2.48312400 1.35996300 -0.28537900
N 3.79689900 1.04707700 -0.18108800
C 4.06970100 -0.28270000 -0.16710000
C 5.40446200 -0.70376300 -0.06121500
C 5.77688700 -2.05216000 -0.04221200
C 7.20800100 -2.43094600 0.07284200
C 4.77141500 -3.06474600 -0.13344000
C 5.14332900 -4.50706000 -0.11786100
C 3.44021700 -2.67626600 -0.23651500
C 3.06471000 -1.32115600 -0.25424800
N 1.74829700 -0.94698900 -0.35126800
C 1.42500000 0.42062000 -0.37199600
C 0.70142300 -1.94304300 -0.42924900
H 0.44104900 3.96258700 -0.44312900
H 6.16608600 0.07587600 0.00714200
H 7.51537200 -3.05141500 -0.78566800
H 7.85981200 -1.55018600 0.12827700
H 7.37199500 -3.05495400 0.96761400
H 5.68540200 -4.76629700 0.80653100
H 4.25546600 -5.14864100 -0.19168500
H 5.81987100 -4.75033100 -0.95383800
H 2.67583600 -3.44911500 -0.30541000
H 0.85192800 -2.57018100 -1.32017100
H -0.25361600 -1.41458200 -0.49674500
H 0.72373100 -2.57494600 0.47076000

OPBE/cc-pvVDZ_T6

N 0.10930200 0.70583200 -0.47787500
C -0.24016600 2.02863900 -0.50014700
O -1.41145000 2.40529000 -0.59349800
N 0.77181800 3.00237800 -0.41287000
C 2.13150400 2.78068400 -0.30488700
O 2.92971400 3.71575200 -0.23490100
C 2.49417400 1.35497300 -0.28487300
N 3.76308900 1.03682900 -0.18731800
C 4.06857500 -0.29686200 -0.17019300
C 5.39554000 -0.69031400 -0.06858500
C 5.79004500 -2.03920200 -0.04422900
C 7.21747800 -2.41549300 0.07034400
C 4.74934100 -3.07620200 -0.13216500
C 5.14634700 -4.50923200 -0.11158000
C 3.42978700 -2.71245900 -0.23232300
C 3.03293800 -1.34462000 -0.25546300
N 1.73660700 -0.95257600 -0.35424200
C 1.39487200 0.38043200 -0.37664800
C 0.67980700 -1.94997300 -0.43782900
H 0.46450400 3.97193700 -0.43201600
H 6.14691100 0.10108900 -0.00582600
H 7.54140600 -3.03857900 -0.78300800
H 7.86344300 -1.52878100 0.11841700
H 7.40154200 -3.03063200 0.97019900
H 5.69964500 -4.75335200 0.81108500
H 4.27099700 -5.16876400 -0.17939700
H 5.82747000 -4.74278200 -0.94753900
H 2.67479900 -3.49573200 -0.29631600
H 0.83917500 -2.57345400 -1.32760100
H -0.27109900 -1.41504800 -0.51022300
H 0.70087500 -2.57672400 0.46369300

OPBE/cc-pvVDZ_T7

N 0.10100200 0.70479800 -0.46332900
C -0.25891000 2.02507800 -0.49634400
O -1.44691700 2.37613700 -0.58562600
N 0.74451400 2.98397500 -0.42635900
C 2.11422100 2.77066600 -0.31792100
O 2.90317800 3.71639700 -0.25823100
C 2.47713200 1.35527300 -0.28691000
N 3.77949300 1.03499200 -0.18511100
C 4.07404900 -0.28981400 -0.16755200
C 5.41072100 -0.69025600 -0.06326000
C 5.79908100 -2.06127500 -0.04184200
C 7.22301000 -2.43410800 0.08428200
C 4.76742700 -3.07344800 -0.13865200
C 5.12913400 -4.50866300 -0.12780400
C 3.43084900 -2.68638600 -0.24204900
C 3.06474800 -1.31982100 -0.25406300
N 1.74185400 -0.93030300 -0.34875800
C 1.41343200 0.41274100 -0.36869100
C 0.69917100 -1.93199100 -0.42595600
H 0.44147700 3.95436700 -0.45215600
H 6.16863700 0.09265400 0.00193800
H 7.54884600 -3.07398600 -0.75647100
H 7.87102200 -1.54960100 0.12656700
H 7.39927800 -3.04344200 0.99076400
H 5.68951800 -4.76702600 0.78850700
H 4.24469200 -5.15392600 -0.19818100
H 5.81357700 -4.74806500 -0.96153200
H 2.67150500 -3.46258200 -0.31705900
H 0.85357900 -2.55295200 -1.32052500
H -0.25816600 -1.40677800 -0.48464600
H 0.73783700 -2.56960400 0.46915800

PBE1PBE/cc-pVDZ_S0

N -2.55192708 -1.30543332 0.00001816
C -3.74065697 -0.62779945 0.00018653
O -4.82479277 -1.18944797 0.00034891
N -3.71638449 0.77563349 0.00007638
C -2.61306070 1.58622505 -0.00009807
O -2.68290490 2.80160157 -0.00017915
C -1.33667489 0.82407874 -0.00006884
N -0.22311751 1.49647781 -0.00004651
C 0.94146426 0.79576926 -0.00000623
C 2.16146428 1.50182238 0.00004880
C 3.37889766 0.85296215 0.00007445
C 4.66382621 1.62248407 0.00001872
C 3.38821016 -0.57278204 0.00003557
C 4.69020821 -1.30887864 0.00022447
C 2.19550237 -1.28448020 -0.00003930
C 0.95994198 -0.62160622 -0.00005059
N -0.24019536 -1.29913114 -0.00014765
C -1.43105684 -0.62295994 -0.00008397
C -0.24586798 -2.75637289 -0.00034891
H -4.62449695 1.23207506 0.00023979
H 2.10244390 2.59160786 0.00009475
H 5.27457876 1.38101074 -0.88430786
H 4.47566562 2.70372222 0.00128753
H 5.27592356 1.37910166 0.88286329
H 5.29203348 -1.04207178 0.88351516
H 4.53909700 -2.39547872 -0.00111074
H 5.29362969 -1.04002779 -0.88132296
H 2.23710666 -2.37216573 -0.00009756
H 0.26664575 -3.12841421 -0.89730922
H -1.28846166 -3.08150306 -0.00097032
H 0.26560016 -3.12868645 0.89710159

PBE1PBE/cc-pVDZ_S1(r1)

N -2.52308037 -1.28837444 -0.00008418
C -3.74789230 -0.63644257 0.00054090
O -4.79303275 -1.27268603 -0.00054236
N -3.74589129 0.73938102 0.00017315
C -2.62872094 1.56932099 -0.00004287
O -2.75939086 2.78843129 0.00013449
C -1.36080938 0.85822356 0.00011783
N -0.21658766 1.57517821 0.00011999
C 0.92652713 0.84823048 0.00008533
C 2.15800181 1.51060845 0.00009553
C 3.38652809 0.83704611 0.00008855
C 4.66031038 1.60787185 -0.00001205
C 3.40645565 -0.58792187 0.00008699
C 4.70187472 -1.33416239 0.00025007
C 2.19713567 -1.26992854 0.00000138
C 0.96131836 -0.59338105 -0.00002286
N -0.22856227 -1.27568284 -0.00016493
C -1.41633059 -0.56369620 -0.00006703
C -0.24058041 -2.72978441 -0.00037224
H -4.65231795 1.19771463 0.00006865
H 2.13346077 2.60147485 0.00012788
H 5.27322989 1.35288969 -0.88078877
H 4.48037109 2.68957557 0.00082306
H 5.27429005 1.35160703 0.87962563
H 5.31015648 -1.08191281 0.88414121
H 4.53490984 -2.41888515 -0.00060503
H 5.31119218 -1.08063002 -0.88253896
H 2.21436524 -2.35870544 -0.00003136
H 0.27355608 -3.10433539 -0.89678431
H -1.28111523 -3.05909901 -0.00057722
H 0.27326856 -3.10459402 0.89609952

PBE1PBE/cc-pVDZ_r5/r6

N -2.56325101 -1.30649543 0.11946054
C -3.71400441 -0.64472898 0.11255959
O -4.83115913 -1.13633798 0.39618074
N -3.72644600 0.71705032 -0.20768192
C -2.61174079 1.51583234 -0.07336012
O -2.76789079 2.75048704 -0.03051514
C -1.35023329 0.82399671 -0.01969711
N -0.21110927 1.55372357 -0.00638717
C 0.93414754 0.83456151 0.00074055
C 2.17413787 1.51138824 0.02130240
C 3.39193811 0.84803767 0.01779839
C 4.67910672 1.61553740 0.04037558
C 3.39919703 -0.56820523 -0.00670493
C 4.69221663 -1.32384040 -0.01323551
C 2.18588521 -1.25974316 -0.02340175
C 0.96002331 -0.59044599 -0.02017265
N -0.25191254 -1.28226244 -0.03283873
C -1.43138514 -0.58691651 0.00878907
C -0.24853688 -2.73330625 -0.04267557
H -4.63300781 1.18505661 -0.19793341
H 2.13593690 2.60244974 0.03970313
H 5.29892189 1.38494963 -0.84113743
H 4.49597575 2.69789029 0.05574483
H 5.28449305 1.35750801 0.92431839
H 5.30042824 -1.08704055 0.87502560
H 4.52183568 -2.40831373 -0.03005234
H 5.30648775 -1.06075345 -0.88994754
H 2.21293028 -2.34845622 -0.04266492
H 0.26475920 -3.10098816 -0.94277052
H -1.28539462 -3.07571293 -0.03856999
H 0.27029246 -3.11759060 0.84769393

PW6B95D3/cc-pVDZ_S0

N 0.11598536 0.70955337 -0.48367149
C -0.22595175 2.03280734 -0.50464294
O -1.37796246 2.41899707 -0.60243794
N 0.79262449 2.99245447 -0.40849182
C 2.13640100 2.76516218 -0.29766773
O 2.95560075 3.65973323 -0.22030521
C 2.47808528 1.32071573 -0.28230840
N 3.72994810 0.99229004 -0.18363222
C 4.04064464 -0.33013518 -0.16855519
C 5.39094602 -0.71111474 -0.06524969
C 5.77244234 -2.03320582 -0.04408836
C 7.21282016 -2.42356580 0.06692474
C 4.76285541 -3.03148871 -0.12942985
C 5.14816643 -4.47537713 -0.10859223
C 3.42824823 -2.67434135 -0.23202385
C 3.04378808 -1.32846603 -0.25388376
N 1.72565565 -0.94180197 -0.35637770
C 1.37990013 0.38121274 -0.37921427
C 0.68475876 -1.95929119 -0.44202084
H 0.48868583 3.95908252 -0.42648429
H 6.12576160 0.08791142 -0.00263198
H 7.52431675 -3.03916218 -0.78671807
H 7.85773858 -1.54154470 0.10800620
H 7.39299294 -3.02349272 0.96843905
H 5.70031728 -4.71833176 0.80850819
H 4.27264827 -5.12679357 -0.17037036
H 5.81811663 -4.71205634 -0.94550113
H 2.68294607 -3.45953385 -0.29646621
H 0.84903686 -2.57792216 -1.32920437
H -0.27312863 -1.44977782 -0.51145978
H 0.71460791 -2.58946344 0.45173730

PW6B95D3/cc-pVDZ_S1(r1)

N 0.14734227 0.70212586 -0.47516398
C -0.23695075 2.03437493 -0.50191837
O -1.41520382 2.34155136 -0.59739676
N 0.74827291 2.98889025 -0.41712299
C 2.11574971 2.76353189 -0.30634371
O 2.89751922 3.70299374 -0.23916174
C 2.48463592 1.36030958 -0.28163048
N 3.78970445 1.03992503 -0.17648712
C 4.06646750 -0.28371133 -0.16396859
C 5.39410590 -0.70283753 -0.06057204
C 5.76591885 -2.04862554 -0.04284955
C 7.19974302 -2.43032010 0.07061550
C 4.76426725 -3.05486811 -0.13279532
C 5.13982386 -4.50045296 -0.11646582
C 3.43872849 -2.66704940 -0.23528396
C 3.06263810 -1.31203282 -0.25272681
N 1.74914816 -0.93523943 -0.35284424
C 1.43146853 0.41209258 -0.37200952
C 0.70531999 -1.94563273 -0.43640146
H 0.44876192 3.95619245 -0.43865655
H 6.15220279 0.07333344 0.00704713
H 7.50564361 -3.05590474 -0.77935200
H 7.84989268 -1.55331435 0.11395775
H 7.37019747 -3.03950654 0.96947134
H 5.68692227 -4.75930434 0.80003027
H 4.25555104 -5.14045534 -0.17955537
H 5.80341836 -4.74910077 -0.95579715
H 2.67764501 -3.43714780 -0.30391242
H 0.86797574 -2.56820388 -1.32252630
H -0.25403500 -1.44032349 -0.50503335
H 0.73612113 -2.57823626 0.45703941

PW6B95D3/cc-pVDZ_r5/r6

N 0.09579614 0.71677448 -0.35245590
C -0.22994619 1.99918532 -0.38077919
O -1.37185708 2.46642745 -0.17635309
N 0.76168500 2.95775949 -0.62966346
C 2.09788341 2.71437808 -0.38183649
O 2.86059598 3.68761201 -0.27024430
C 2.46944564 1.32874428 -0.30304456
N 3.77963818 1.02381103 -0.18696582
C 4.06218365 -0.29850635 -0.16564340
C 5.40339947 -0.71379804 -0.04230305
C 5.77645731 -2.04615841 -0.02522199
C 7.21576381 -2.43739602 0.10829917
C 4.77483714 -3.03660278 -0.13472137
C 5.13970684 -4.48785540 -0.12109801

C 3.44136228 -2.65095660 -0.25395991
C 3.06736797 -1.30795421 -0.27206772
N 1.73109246 -0.92302887 -0.38785930
C 1.40466525 0.40528515 -0.36378431
C 0.70204069 -1.94165332 -0.48706986
H 0.46680919 3.93188376 -0.64966816
H 6.15176082 0.07152258 0.03952028
H 7.54419846 -3.04474659 -0.74562404
H 7.86187923 -1.55704084 0.17095992
H 7.38101952 -3.04855648 1.00568500
H 5.66362524 -4.75908509 0.80545642
H 4.25364868 -5.12316534 -0.20988311
H 5.82177674 -4.73564435 -0.94575603
H 2.68748586 -3.42719424 -0.33687966
H 0.87668745 -2.55998589 -1.37432596
H -0.26349741 -1.44753520 -0.56154060
H 0.72148490 -2.58346595 0.40101433

PW6B95D3/cc-pVDZ_r7

N 0.09853424 0.71413796 -0.47828635
C -0.24242734 2.02478630 -0.50539995
O -1.42394579 2.38378752 -0.60346391
N 0.75290550 2.98026713 -0.42047808
C 2.11587562 2.75366327 -0.30634593
O 2.90210137 3.69169931 -0.23740920
C 2.48183036 1.34847987 -0.28073748
N 3.78273333 1.03157066 -0.17342600
C 4.07230537 -0.29432094 -0.16019303
C 5.40640493 -0.69875473 -0.05842260
C 5.78800756 -2.04986969 -0.04167021
C 7.21426724 -2.42765458 0.07548791
C 4.77157714 -3.05928079 -0.13541416
C 5.13031023 -4.49844880 -0.12403029
C 3.43378970 -2.65724159 -0.23781697
C 3.08573785 -1.31790623 -0.24898096
N 1.71535841 -0.91439219 -0.35311065
C 1.41394983 0.40118338 -0.37306622
C 0.69876571 -1.93773163 -0.43215212
H 0.45446012 3.94732454 -0.44295091
H 6.16054924 0.08093535 0.00816951
H 7.52336677 -3.07476736 -0.75980119
H 7.86633871 -1.55221530 0.10673610
H 7.38857156 -3.02971743 0.98168487
H 5.69324001 -4.76343607 0.78390144
H 4.24407560 -5.13494131 -0.18168121
H 5.79453326 -4.75075221 -0.96522193
H 2.67449999 -3.42807486 -0.30895163
H 0.87496952 -2.56559987 -1.31501760
H -0.27223222 -1.45424703 -0.49949627
H 0.74854288 -2.57542904 0.45973084

revTPSS/aug-cc-pVDZ_S0

N -2.56970675 -1.32161723 -0.00022540
C -3.75766094 -0.63706917 -0.00018317
O -4.86481775 -1.20299038 -0.00007049
N -3.73773938 0.77718220 -0.00035933
C -2.62385250 1.59280134 0.00033624
O -2.70653410 2.82723348 0.00115001
C -1.34739078 0.82953772 -0.00000534
N -0.21991355 1.51289837 -0.00008837
C 0.94869696 0.80547142 -0.00019596
C 2.17550665 1.51591087 -0.00036714
C 3.39632318 0.85721986 -0.00027105
C 4.69394785 1.62675032 -0.00087255
C 3.40443867 -0.57644528 0.00022177
C 4.71674068 -1.31598119 0.00111450
C 2.20634779 -1.29478370 0.00024023
C 0.96848115 -0.62339497 -0.00007966
N -0.23991523 -1.30270728 -0.00018664
C -1.43888902 -0.61916869 -0.00015071
C -0.23574253 -2.77960781 -0.00030589
H -4.65071652 1.23560279 0.00025027
H 2.12250035 2.60841714 -0.00053081
H 5.29825062 1.37853262 -0.89150471
H 4.50118201 2.70977977 0.00184074
H 5.30173208 1.37450746 0.88619503
H 5.31272622 -1.04512272 0.89093800
H 4.55754892 -2.40400476 -0.00193559
H 5.31697720 -1.04049376 -0.88433874
H 2.24628193 -2.38495701 0.00055197
H 0.28588311 -3.13131792 -0.90223067
H -1.27634406 -3.11109804 -0.00136650
H 0.28403428 -3.13152697 0.90262553

revTPSS/aug-cc-pVDZ_S1(r1)

N -2.53922287 -1.31345752 0.00028516
C -3.76393748 -0.65218142 0.00006475
O -4.82562380 -1.30675644 0.00019260
N -3.77235278 0.72956581 -0.00034031
C -2.64026622 1.56921422 -0.00026402
O -2.79521177 2.80726494 -0.00055214
C -1.37210834 0.86582700 0.00015251
N -0.21572255 1.59192805 0.00046670
C 0.93882317 0.85055992 0.00047563
C 2.17546550 1.51886450 0.00048116
C 3.41286799 0.83715949 0.00008589
C 4.69766471 1.61198659 -0.00029991
C 3.42961310 -0.58873015 -0.00010325
C 4.73325732 -1.34457834 -0.00005104
C 2.21084630 -1.27463736 -0.00018099
C 0.97355632 -0.58588272 0.00014786
N -0.23226514 -1.27587883 0.00016938
C -1.41749689 -0.56604992 0.00034464
C -0.22763285 -2.75201711 -0.00045073
H -4.68312726 1.19007766 -0.00045861
H 2.15761044 2.61264836 0.00061977
H 5.30578532 1.35282952 -0.88709338
H 4.51101203 2.69518467 0.00127819
H 5.30788721 1.35047511 0.88432264
H 5.33696936 -1.08919702 0.88950639
H 4.55390648 -2.42994203 -0.00148293
H 5.33836919 -1.08702312 -0.88800639
H 2.22435248 -2.36584389 -0.00044386
H 0.29563622 -3.10176288 -0.90296781
H -1.26475989 -3.09125708 -0.00036603
H 0.29607052 -3.10256799 0.90148689

revTPSS/aug-cc-pVDZ_r7

N -2.57656985 -1.32131599 -0.00675504
C -3.76867297 -0.64820722 0.00867858
O -4.86730352 -1.25606787 0.01493362
N -3.75359909 0.74749810 0.01717262
C -2.62503118 1.57688872 0.00945465
O -2.75894802 2.81803891 0.01592150
C -1.35548104 0.86030086 -0.00475874
N -0.21315713 1.58231760 -0.01665660
C 0.95816942 0.83833764 -0.01402123
C 2.18319010 1.52456924 -0.01072240
C 3.43136480 0.84755157 -0.00060896
C 4.70688018 1.61731841 -0.02313091
C 3.43661241 -0.59905212 0.01801417
C 4.71946197 -1.36218470 0.05083258
C 2.19919959 -1.28565545 0.01123398
C 0.99188213 -0.58644496 -0.00817898
N -0.26264039 -1.29503497 -0.01766725
C -1.42837562 -0.58776207 -0.00748859
C -0.24178021 -2.75854290 -0.04034584
H -4.66114238 1.21472813 0.02701838
H 2.15807463 2.61760608 -0.01676679
H 5.28599757 1.38985230 -0.94269607
H 4.52465979 2.69948299 0.02186688
H 5.36765619 1.31931320 0.81540378
H 5.28882977 -1.13388015 0.97519257
H 4.54052780 -2.44536545 0.00579337
H 5.38151730 -1.06774785 -0.78705070
H 2.21569832 -2.37602745 0.02512635
H 0.30841502 -3.09407596 -0.93515589
H -1.27506546 -3.11056861 -0.06578812
H 0.27209161 -3.12604081 0.86380935

revTPSS/aug-cc-pVDZ_r8

N -2.58737213 -1.26139949 0.00149201
C -3.80908405 -0.65161356 0.00019695
O -4.88898797 -1.27043307 0.00067974
N -3.77227046 0.74847043 -0.00099516
C -2.63604534 1.57962880 -0.00103245
O -2.74735626 2.82309122 -0.00189195
C -1.38893584 0.85080242 -0.00059276
N -0.18908847 1.49834098 -0.00018658
C 0.98609756 0.87013529 0.00026265
C 2.24559367 1.53049868 0.00000914
C 3.44559347 0.82674694 0.00119589
C 4.76117996 1.57100822 0.00422907
C 3.43493783 -0.59801558 -0.00194891
C 4.72174380 -1.38189199 -0.00394426
C 2.19572767 -1.26221297 -0.00186870
C 0.98038257 -0.58346998 0.00026621
N -0.25365801 -1.25869901 0.00097648
C -1.43200623 -0.56169929 0.00034773
C -0.27949272 -2.73258560 0.00226811
H -4.68034803 1.21583914 -0.00145589
H 2.23401632 2.62382695 0.00017345
H 5.36029401 1.30540713 -0.88479070
H 4.59442465 2.65824090 0.00599817
H 5.35793441 1.30225130 0.89387113
H 5.33325088 -1.14015430 0.88437403
H 4.52100052 -2.46366959 -0.00557129
H 5.33204870 -1.13727537 -0.89231166
H 2.18091601 -2.35830277 -0.00362679
H 0.22236014 -3.10476903 -0.90388729
H -1.32974637 -3.05058600 0.00517528
H 0.22717207 -3.10305226 0.90640977

revTPSS/aug-cc-pVDZ_T1

N -2.53943400 -1.30664900 -0.00085500
C -3.76591100 -0.65131800 -0.00043000
O -4.83346900 -1.29377200 -0.00124100
N -3.76867300 0.73526400 0.00104600
C -2.64365800 1.56795900 0.00120100
O -2.78097800 2.80519800 0.00228200
C -1.36833800 0.85611900 0.00012200
N -0.21938900 1.58806300 -0.00016700
C 0.93674100 0.85203000 -0.00036000
C 2.17080900 1.52456000 -0.00103500
C 3.40244500 0.84570900 -0.00068600
C 4.68863200 1.61859100 -0.00295500
C 3.42606400 -0.58943700 0.00100300
C 4.73555000 -1.33394900 0.00348400
C 2.21654500 -1.28083500 0.00097500
C 0.97655300 -0.59848300 0.00001400
N -0.21738400 -1.29144200 -0.00069000
C -1.42967900 -0.56693800 -0.00049700
C -0.22001700 -2.76422300 -0.00123600
H -4.67990800 1.19634300 0.00176100
H 2.14994700 2.61795500 -0.00180200
H 5.29077300 1.36170200 -0.89403600
H 4.50248800 2.70176000 0.00342100
H 5.30093400 1.35214800 0.87811700
H 5.33306800 -1.07665800 0.89659100
H 4.56433000 -2.42046900 -0.00270300
H 5.34230300 -1.06751900 -0.88051100
H 2.23362300 -2.37180700 0.00191800
H 0.30021500 -3.12163800 -0.90278100
H -1.26011200 -3.09619500 -0.00276100
H 0.29765000 -3.12239300 0.90151600

revTPSS/aug-cc-pVDZ_T5

N -2.55611600 -1.33032800 0.05151600
C -3.72745000 -0.67579700 0.03965800
O -4.86262100 -1.17958000 0.30233900
N -3.75382300 0.70015700 -0.24161100
C -2.63121600 1.52699500 -0.08171800
O -2.84621200 2.77633500 -0.02358600
C -1.36644400 0.84587800 -0.03378600
N -0.21936100 1.56422500 0.02503500
C 0.95178700 0.84228600 0.01923400
C 2.18296400 1.52160500 0.06250700
C 3.42163200 0.84509300 0.04803500
C 4.70990300 1.62319800 0.09915100
C 3.43538600 -0.57032300 -0.01394300
C 4.74054200 -1.32954000 -0.03669500
C 2.21531700 -1.26344000 -0.05364200
C 0.98105000 -0.58322900 -0.03640400
N -0.23551200 -1.27908000 -0.06024300
C -1.43697300 -0.59092500 -0.03190500
C -0.21984100 -2.75073100 -0.05597100
H -4.66935300 1.16086200 -0.25074800
H 2.15779100 2.61483500 0.10945900
H 5.33395900 1.40762500 -0.78758800
H 4.51654200 2.70540500 0.13970200
H 5.30609900 1.33661200 0.98506300
H 5.34195900 -1.11113600 0.86414900
H 4.55976000 -2.41441300 -0.07984400
H 5.35069100 -1.04217000 -0.91206800
H 2.23669900 -2.35404500 -0.09979300
H 0.30848300 -3.10522000 -0.95424500
H -1.25580900 -3.09659800 -0.06241200
H 0.29758800 -3.10703200 0.84829900

revTPSS/aug-cc-pVDZ_T6

N -2.56121600 -1.33202800 -0.00109900
C -3.75167500 -0.65941600 -0.00044000
O -4.85847600 -1.23708500 -0.00079100
N -3.75468900 0.75944700 0.00057800
C -2.63379000 1.58326400 0.00100000
O -2.76469500 2.82270500 0.00179100
C -1.35833100 0.85710300 0.00044700
N -0.23582000 1.55189900 0.00034600
C 0.94043400 0.84208600 -0.00010200
C 2.15431800 1.53331200 -0.00079500
C 3.39776500 0.87426400 -0.00081200
C 4.67952100 1.64968100 -0.00284400
C 3.42030600 -0.59900900 0.00088600
C 4.74268900 -1.31524800 0.00295000
C 2.23666300 -1.30578100 0.00101900
C 0.97321300 -0.63631500 0.00000500
N -0.21695400 -1.30671600 -0.00055300
C -1.42084800 -0.62108300 -0.00035800
C -0.22007000 -2.78599700 -0.00113700
H -4.66996800 1.21314400 0.00092300
H 2.11352300 2.62690700 -0.00132300
H 5.29398500 1.40234400 -0.89012000
H 4.48276500 2.73189600 -0.00238200
H 5.29729200 1.40200400 0.88197500
H 5.33905100 -1.03866700 0.89183500
H 4.59553800 -2.40546100 0.00244300
H 5.34236300 -1.03815100 -0.88349400
H 2.27232600 -2.39686900 0.00214100
H 0.30133900 -3.13775800 -0.90272700
H -1.26110800 -3.11513200 -0.00324800
H 0.29784900 -3.13860300 0.90216200

revTPSS/aug-cc-pVDZ_T7

N -2.56911300 -1.32536500 -0.01220500
C -3.76069300 -0.65260300 -0.00476500
O -4.86158800 -1.26333800 -0.00910700
N -3.75667900 0.74358100 0.00785100
C -2.63290900 1.57767200 0.01032500
O -2.76857300 2.81645900 0.01939900
C -1.36404000 0.85374200 0.00259500
N -0.21758800 1.56644800 0.00245500
C 0.94759500 0.85134300 -0.00032400
C 2.16913600 1.53597000 -0.00329800
C 3.42444700 0.85450100 -0.00359900
C 4.70546200 1.62310300 -0.03514700
C 3.42744300 -0.60501600 0.01044500
C 4.72567400 -1.34936100 0.03366200
C 2.21718200 -1.30067700 0.00856500
C 0.97500600 -0.60436600 -0.00003200
N -0.23176000 -1.28328800 -0.00589600
C -1.42689100 -0.57925900 -0.00487000
C -0.23155100 -2.75868800 -0.01260800
H -4.66831400 1.20306800 0.01323900
H 2.14325700 2.62895400 -0.00696900
H 5.27551000 1.40472000 -0.96096900
H 4.51701600 2.70509500 0.01192000
H 5.36727900 1.33621000 0.80486700
H 5.30279200 -1.09500400 0.94387200
H 4.56141800 -2.43577000 0.00842600
H 5.36085400 -1.05827000 -0.82435800
H 2.23990900 -2.39080900 0.01852200
H 0.30245100 -3.10517400 -0.90979900
H -1.27087900 -3.09163400 -0.02605300
H 0.28081700 -3.11416100 0.89383400

revTPSSh/aug-cc-pVDZ_S0

N -2.48276103 -1.26707015 0.00000464
C -3.63151160 -0.61414202 0.00028878
O -4.69749888 -1.16024192 0.00053275
N -3.61441913 0.74733785 0.00018869
C -2.54134622 1.53320203 0.00016200
O -2.62000264 2.72182761 0.00027047
C -1.30353277 0.80112759 -0.00006194
N -0.21458551 1.45197680 -0.00020677
C 0.91883660 0.78001797 -0.00020093
C 2.10575481 1.46678951 -0.00022994
C 3.28348930 0.82871663 -0.00009086
C 4.53982980 1.56726639 -0.00066783
C 3.28929536 -0.55664797 0.00037278
C 4.55754917 -1.27024745 0.00135430
C 2.13187722 -1.24890664 0.00024822
C 0.93571128 -0.59904449 -0.00010193
N -0.23162697 -1.25481928 -0.00048754
C -1.38759615 -0.60029479 -0.00015593
C -0.22721999 -2.67623806 -0.00122195
H -4.49071329 1.18679235 0.00046809
H 2.05720345 2.51648496 -0.00039984
H 5.11771089 1.32725005 -0.85719539
H 4.36019327 2.60860263 0.00309783
H 5.12209957 1.32159597 0.85120215
H 5.12803298 -1.00985703 0.85699509
H 4.40653135 -2.31571229 -0.00228419
H 5.13286209 -1.00430285 -0.84924241
H 2.16666613 -2.29671763 0.00068277
H 0.27174108 -3.01933859 -0.86745326
H -1.22669167 -2.99861546 -0.00174790
H 0.27130377 -3.02043629 0.86478944

revTPSSh/aug-cc-pVDZ_S1(r1)/r5

N -2.44960457 -1.25720790 -0.00022204
C -3.63602490 -0.63256522 -0.00014454
O -4.66073781 -1.25564721 0.00002465
N -3.64431807 0.70231271 0.00016556
C -2.55930913 1.50955678 0.00023926
O -2.70177828 2.70255450 0.00051963
C -1.32736233 0.83286162 -0.00001245
N -0.21472712 1.52556562 -0.00006825
C 0.90590986 0.82438015 -0.00008396
C 2.09757542 1.47102875 -0.00015283
C 3.29512424 0.81151336 -0.00008270
C 4.53534263 1.55934468 -0.00069703
C 3.31508880 -0.56819311 0.00025576
C 4.57794993 -1.29160448 0.00095052
C 2.14053516 -1.23093299 0.00015214
C 0.94009797 -0.56832996 -0.00005292
N -0.21622357 -1.23477247 -0.00022291
C -1.37230815 -0.55068802 -0.00014793
C -0.21499503 -2.65395194 -0.00043436
H -4.51978542 1.14208016 0.00038532
H 2.08196151 2.52164700 -0.00028117
H 5.11648014 1.30930150 -0.85396166

H 4.35837598 2.60021021 0.00223734
H 5.12048984 1.30482159 0.84840619
H 5.15437428 -1.04420524 0.85688093
H 4.41174509 -2.33509201 -0.00180313
H 5.15788616 -1.04009080 -0.85134519
H 2.15110739 -2.27966212 0.00028498
H 0.28459047 -3.00002015 -0.86617076
H -1.21277864 -2.97974007 -0.00078937
H 0.28404848 -3.00031180 0.86550187

revTPSSh/aug-cc-pVDZ_r6

N -2.50161207 -1.25285797 0.00092458
C -3.65145677 -0.61161108 0.00039998
O -4.72081204 -1.17070593 0.00034706
N -3.63028276 0.73979976 0.00000527
C -2.54286782 1.51851680 -0.00055508
O -2.63238906 2.71758208 -0.00133338
C -1.31856311 0.80582510 -0.00008968
N -0.19911144 1.50310091 -0.00019843
C 0.90494855 0.78238522 0.00010681
C 2.14031978 1.44540786 0.00051091
C 3.33405960 0.81036997 0.00049741
C 4.58341683 1.55739498 0.00129432
C 3.33079925 -0.55326478 -0.00021794
C 4.56547495 -1.30585254 -0.00140774
C 2.12140163 -1.22797508 -0.00025843
C 0.91697804 -0.58153961 0.00001185
N -0.24460994 -1.25778017 0.00015443
C -1.40231061 -0.57216710 0.00030836
C -0.24273393 -2.67322262 0.00000152
H -4.49953308 1.19173054 -0.00061039
H 2.10094847 2.49641563 0.00076718
H 5.16402266 1.31393770 -0.85225282
H 4.39937941 2.59748399 -0.00020346
H 5.16143258 1.31607670 0.85723902
H 5.15346111 -1.05039471 0.84639153
H 4.38865845 -2.34652173 0.00221898
H 5.14791413 -1.05587747 -0.85479005
H 2.14873232 -2.27660457 -0.00056498
H 0.24909412 -3.03105346 -0.86622565
H -1.24449999 -2.99032285 -0.00035708
H 0.24851359 -3.03131930 0.86646370

revTPSSh/aug-cc-pVDZ_r7

N 2.49248835 -1.26914185 -0.00398618
C 3.63620465 -0.62338137 -0.00014006
O 4.69952195 -1.21198771 0.00007643
N 3.62909538 0.71836446 0.00358457
C 2.54146344 1.51602045 0.00378490
O 2.67108588 2.71152493 0.00720557
C 1.31337380 0.82856015 0.00013645
N 0.20233834 1.51595061 -0.00103311
C -0.92472422 0.81675421 -0.00161254
C -2.11442935 1.47656209 -0.00148900
C -3.31324395 0.81808997 -0.00125844
C -4.55328430 1.55380572 -0.01205015
C -3.31754418 -0.57919262 0.00377419
C -4.56156845 -1.31331139 0.01637787
C -2.12395658 -1.23993455 0.00156956
C -0.95829224 -0.56487976 -0.00164527
N 0.25453309 -1.24430591 -0.00384456
C 1.37546621 -0.56337774 -0.00231655
C 0.23421810 -2.65402563 -0.00781905
H 4.50098746 1.16443334 0.00637631
H -2.09430997 2.52675711 -0.00295007
H -5.16312608 1.27796417 0.81496598
H -4.38460175 2.59531793 0.01284235
H -5.12123812 1.31627738 -0.88073336
H -5.17112553 -1.04264420 -0.81194390
H -4.39088988 -2.35491610 -0.00906133
H -5.12750679 -1.08069351 0.88706454
H -2.13580774 -2.28808837 0.00439594
H -0.27372484 -2.99705053 0.85682795
H 1.22598783 -2.99815562 -0.01266366
H -0.28079097 -2.99170764 -0.87029313

SOGGA11/cc-pVDZ_S0

N 0.11151085 0.71983393 -0.48766172
C -0.24032389 2.04514070 -0.50903629
O -1.40067202 2.42822927 -0.61345749
N 0.78521083 3.00621921 -0.40368673
C 2.13786549 2.77679220 -0.29557683
O 2.96050549 3.67815179 -0.21631709
C 2.47787405 1.32351512 -0.28507535
N 3.73621388 0.98746003 -0.18802892
C 4.04105573 -0.33559752 -0.17344843
C 5.39376394 -0.72166514 -0.07066080
C 5.79295505 -2.04456908 -0.04533531
C 7.24691907 -2.40037559 0.06953009
C 4.78222512 -3.05051347 -0.12858303
C 5.14478632 -4.50562392 -0.10764339
C 3.44233061 -2.68673720 -0.23039809
C 3.03966369 -1.34320244 -0.25588330
N 1.71845167 -0.95373951 -0.35746199
C 1.37710290 0.38632516 -0.38167481
C 0.65633041 -1.95217198 -0.44128244
H 0.48411206 3.97572660 -0.41656824
H 6.12739877 0.08317807 -0.00935617
H 7.58238961 -3.01242376 -0.78404088
H 7.87248103 -1.49789360 0.11124058
H 7.44522888 -2.99789436 0.97503042
H 5.69177408 -4.76626710 0.81388515
H 4.25437561 -5.14554744 -0.17431747
H 5.81806562 -4.75930988 -0.94367769
H 2.70371655 -3.47996081 -0.29092626
H 0.79963886 -2.58392607 -1.33023587
H -0.29414150 -1.41490514 -0.51544688
H 0.66018794 -2.58519441 0.45828106

SOGGA11/cc-pVDZ_S1(r1)

N 0.12971045 0.70850871 -0.46958168
C -0.25320793 2.04343012 -0.49535539
O -1.44622851 2.34149618 -0.58526596
N 0.73306676 3.00039227 -0.41892548
C 2.11795232 2.77324559 -0.31561329
O 2.89723215 3.72454797 -0.25979872
C 2.48450736 1.36729950 -0.28656122
N 3.79501107 1.03373799 -0.18159875
C 4.06642716 -0.29291032 -0.16729892
C 5.40046497 -0.71809302 -0.06155685
C 5.78904596 -2.06404247 -0.04086203
C 7.23656873 -2.41478219 0.07957851
C 4.78639957 -3.07045536 -0.13345830
C 5.13315103 -4.52930275 -0.12141714
C 3.45382155 -2.67165748 -0.23618977
C 3.06791690 -1.31982451 -0.25396563
N 1.73961489 -0.93074872 -0.35304568
C 1.43099207 0.40892663 -0.37131354
C 0.67970655 -1.93096257 -0.43516409
H 0.43628783 3.97005968 -0.44079623
H 6.15678028 0.06467492 0.00696848
H 7.57058213 -3.03439709 -0.77077157
H 7.86871498 -1.51795347 0.12794333
H 7.42447814 -3.02436628 0.98097049
H 5.67005395 -4.81045232 0.80067538
H 4.23260000 -5.15473938 -0.19587095
H 5.80251918 -4.79274698 -0.95831679
H 2.69875683 -3.44948488 -0.30421236
H 0.83043864 -2.56375576 -1.32275231
H -0.27722222 -1.40746556 -0.50844620
H 0.69285354 -2.56512369 0.46419015

SOGGA11/cc-pVDZ_r7/r8

N 0.11698985 0.75281488 -0.47762940
C -0.30850340 2.05328226 -0.50405432
O -1.48555820 2.38527047 -0.59918090
N 0.71752720 2.99832824 -0.41231160
C 2.09553516 2.76783813 -0.30637542
O 2.90960040 3.69218715 -0.23745186
C 2.43592369 1.36260784 -0.28839077
N 3.72008805 0.95526837 -0.18893270
C 4.10384077 -0.30653486 -0.16688088
C 5.45328051 -0.72817531 -0.06367883
C 5.81811065 -2.06493425 -0.04190004
C 7.27080015 -2.43596037 0.07331029
C 4.80966648 -3.06147289 -0.12687527
C 5.14104036 -4.52428253 -0.10786534
C 3.47092514 -2.65266668 -0.23004228
C 3.07647858 -1.32415023 -0.25393939
N 1.73529497 -0.92162414 -0.35706970
C 1.40277278 0.40842412 -0.37628121
C 0.66980450 -1.91405899 -0.44442287
H 0.42154482 3.96881124 -0.42857881
H 6.20946564 0.05467288 -0.00028177
H 7.59628448 -3.05060100 -0.78234352
H 7.90897579 -1.54267890 0.11811872
H 7.45828945 -3.03965447 0.97702680
H 5.67543991 -4.80805900 0.81513280
H 4.23444551 -5.14159876 -0.17863261
H 5.80739767 -4.80093870 -0.94311297
H 2.70673663 -3.42587615 -0.29356852
H 0.80410765 -2.54457366 -1.33709962
H -0.29030162 -1.38783116 -0.51845007
H 0.66299278 -2.55077876 0.45394950

SOGGA11/cc-pVDZ_r9

N 0.09380431 0.72214050 -0.48250452
C -0.29046696 1.98820618 -0.50464838
O -1.43746824 2.45547236 -0.59551757
N 0.75018804 2.99860701 -0.41151133
C 2.12489758 2.73713134 -0.30225405
O 2.86709681 3.72307273 -0.23400078
C 2.46845273 1.32794404 -0.28636706
N 3.79496082 1.01013850 -0.18346460
C 4.06877117 -0.30191328 -0.17036842
C 5.42044977 -0.72719148 -0.06753932
C 5.80393082 -2.05657188 -0.04348074
C 7.25700233 -2.42321650 0.07037848
C 4.79202578 -3.05446688 -0.12676168
C 5.13352694 -4.51582165 -0.10635912
C 3.45163192 -2.66331955 -0.22944818
C 3.05990107 -1.32301342 -0.25424652
N 1.72531060 -0.93511196 -0.35792410
C 1.40617551 0.41518921 -0.37713568
C 0.67369675 -1.93557775 -0.44471569
H 0.46213779 3.97931078 -0.42579480
H 6.16904333 0.06340440 -0.00495427
H 7.58883155 -3.03497963 -0.78521031
H 7.89004707 -1.52589723 0.11555598
H 7.45219708 -3.02651766 0.97294487
H 5.67201762 -4.79332383 0.81609873
H 4.23169809 -5.14025455 -0.17578377
H 5.80266370 -4.78716721 -0.94104199
H 2.70347310 -3.44775720 -0.29112086
H 0.81940530 -2.57104207 -1.33244739
H -0.28242528 -1.40923161 -0.52017546
H 0.67601923 -2.57518695 0.45198600

SOGGA11x/cc-pVDZ_S0

N 0.10633977 0.71237789 -0.48330939
C -0.23149525 2.04032268 -0.50545345
O -1.38395325 2.42758157 -0.60251485
N 0.78678047 3.00344349 -0.41187163
C 2.13268529 2.77722546 -0.30018797
O 2.95093621 3.67315390 -0.22404969
C 2.47723316 1.32740559 -0.28293420
N 3.73045062 0.99905353 -0.18363937
C 4.04192505 -0.32841069 -0.16776830
C 5.39826592 -0.70869649 -0.06362914
C 5.78025733 -2.03550483 -0.04293011
C 7.22680499 -2.42605883 0.06856549
C 4.76678253 -3.03739148 -0.12969765
C 5.15201460 -4.48755443 -0.11019469
C 3.42712101 -2.68058675 -0.23226729
C 3.04192078 -1.32883609 -0.25331093
N 1.71908799 -0.94051138 -0.35553192
C 1.37378123 0.38252127 -0.37915002
C 0.67583122 -1.96308747 -0.43977917
H 0.48167213 3.97116416 -0.43127927
H 6.13583608 0.09308472 -0.00034567
H 7.54432647 -3.03023603 -0.79557038
H 7.87018795 -1.53869685 0.12159095
H 7.40654142 -3.03572073 0.96788763
H 5.69438144 -4.73531272 0.81565219
H 4.27180245 -5.13807883 -0.18200960
H 5.82911643 -4.72321319 -0.94615881
H 2.67928261 -3.46836209 -0.29718642
H 0.84263187 -2.58311096 -1.32943220
H -0.28570464 -1.45442477 -0.51114551
H 0.70615283 -2.59048599 0.45983718

SOGGA11x/cc-pVDZ_S1(r1)

N 0.14124629 0.70577549 -0.47571540
C -0.24160069 2.04191585 -0.50430692
O -1.41820232 2.35420121 -0.59970360
N 0.74598470 3.00047887 -0.42141420
C 2.11257439 2.77591622 -0.31001189
O 2.89712759 3.71341428 -0.24418320
C 2.48368308 1.36499814 -0.28212421
N 3.79084315 1.04586316 -0.17548947
C 4.06900665 -0.27947673 -0.16241448
C 5.40275466 -0.69819828 -0.05731297
C 5.77318033 -2.04744115 -0.04099175
C 7.21231818 -2.43152528 0.07331131
C 4.76583509 -3.06087338 -0.13420361
C 5.14539808 -4.51101845 -0.12017645
C 3.43620598 -2.67642992 -0.23686170
C 3.05605990 -1.31583308 -0.25270392
N 1.74319902 -0.94061577 -0.35099321
C 1.42286143 0.41644270 -0.37204016
C 0.69460960 -1.95269290 -0.43100882
H 0.44372193 3.96849082 -0.44517304
H 6.16339293 0.08053259 0.01193024
H 7.52278885 -3.04365933 -0.78916679
H 7.86164756 -1.54995892 0.13065553
H 7.37994137 -3.05250807 0.96862858
H 5.68248321 -4.77387532 0.80542723
H 4.25845313 -5.15243501 -0.19574293
H 5.81902893 -4.75521435 -0.95742653
H 2.67277867 -3.44909786 -0.30697682
H 0.85449369 -2.57809100 -1.31970599
H -0.26624913 -1.44349506 -0.50046829
H 0.72343034 -2.58253579 0.46854927

SOGGA11x/cc-pVDZ_r5

N 0.10160159 0.72180728 -0.59046604
C -0.23311838 2.00438828 -0.60395330
O -1.31408575 2.49385243 -0.97666389
N 0.72491099 2.96470390 -0.20202993
C 2.09979238 2.72684434 -0.24626877
O 2.84548376 3.71179667 -0.23011335
C 2.46860273 1.33452535 -0.27826109
N 3.78083824 1.03274059 -0.19206230
C 4.06541057 -0.29803124 -0.17826736
C 5.41286528 -0.71107434 -0.08943586
C 5.78641145 -2.05012870 -0.05986296
C 7.23467216 -2.44044787 0.03529351
C 4.77804597 -3.04432312 -0.12108440
C 5.14285651 -4.50134502 -0.09114855
C 3.43699242 -2.65834170 -0.21071908
C 3.06419247 -1.31043267 -0.23794306
N 1.71844029 -0.92300457 -0.33078280
C 1.40109297 0.40644663 -0.39008025
C 0.68638960 -1.94635662 -0.40627384
H 0.42571809 3.94172505 -0.18481236
H 6.16732996 0.07668314 -0.04247661
H 7.54149775 -3.05342333 -0.82731947
H 7.88233283 -1.55496144 0.07357327
H 7.42663147 -3.04548273 0.93603513
H 5.68840968 -4.75861362 0.83118584
H 4.24996163 -5.13744648 -0.14772211
H 5.80480369 -4.76530938 -0.93180870
H 2.67714761 -3.43686869 -0.25820672
H 0.84645451 -2.57704393 -1.29219402
H -0.28365485 -1.45376708 -0.47749373
H 0.72096904 -2.57605747 0.49354864

tHCTHhyb/cc-pVDZ_S0

N -2.56477332 -1.31241105 0.00005385
C -3.76076895 -0.63399992 0.00014644
O -4.84828443 -1.19893434 0.00023036
N -3.73629765 0.77791108 0.00021199
C -2.62834095 1.59549383 -0.00010148
O -2.70260247 2.81541772 0.00027651
C -1.34593176 0.82999896 0.00003612
N -0.22572819 1.50576811 0.00002450
C 0.94282594 0.80202411 -0.00000008
C 2.16877317 1.51079463 -0.00004094
C 3.39092045 0.85710151 -0.00005031
C 4.68353772 1.62925289 -0.00013039
C 3.40087798 -0.57496495 -0.00000986
C 4.71063657 -1.31333992 0.00002655
C 2.20296600 -1.29102289 0.00002832
C 0.96263630 -0.62432485 0.00002831
N -0.24260467 -1.30360578 0.00005996
C -1.44054677 -0.62333332 0.00002822
C -0.24905340 -2.76953820 0.00011968
H -4.64663629 1.23514384 0.00036819
H 2.11085074 2.60258715 -0.00005877
H 5.29487315 1.38417747 -0.88638514
H 4.49538436 2.71309702 0.00016798
H 5.29527861 1.38374030 0.88571552
H 5.31318369 -1.04319938 0.88513585
H 4.55956256 -2.40252306 -0.00030771
H 5.31357232 -1.04268362 -0.88465079
H 2.24419806 -2.38064666 0.00004822
H 0.26512146 -3.13969717 -0.89958294
H -1.29480269 -3.09225863 0.00025777
H 0.26535634 -3.13961792 0.89971772

tHCTHhyb/cc-pVDZ_S1(r1)/r6

N -2.53760251 -1.29834565 0.00006068
C -3.76868100 -0.64286027 -0.00025915
O -4.81624376 -1.28548938 0.00040865
N -3.76781828 0.73875653 0.00013886
C -2.64375335 1.57810230 0.00034939
O -2.78279983 2.80089488 0.00029314
C -1.37133692 0.86511229 0.00007421
N -0.21941326 1.58546835 0.00003773
C 0.92923358 0.85299696 -0.00000086
C 2.16649347 1.51856835 -0.00004567
C 3.40122142 0.84009727 -0.00005423
C 4.68279696 1.61401762 -0.00013813
C 3.41973593 -0.58864119 0.00000094
C 4.72182957 -1.34002074 0.00002628
C 2.20355851 -1.27414629 0.00005130
C 0.96426657 -0.59169609 0.00004503
N -0.23512128 -1.27678915 0.00007642
C -1.42368007 -0.56502734 0.00008734
C -0.24468700 -2.74057551 0.00008340
H -4.67631137 1.19788686 0.00026773
H 2.14281660 2.61155645 -0.00007350
H 5.29644089 1.35678472 -0.88305737
H 4.50246522 2.69828423 0.00001069
H 5.29671977 1.35656704 0.88251730
H 5.33097378 -1.08570303 0.88598448
H 4.55290212 -2.42704768 -0.00011174
H 5.33114651 -1.08549414 -0.88574832
H 2.22070625 -2.36482887 0.00008757
H 0.27276237 -3.11076357 -0.89886797
H -1.28722431 -3.07049394 0.00009865

H 0.27278545 -3.11076347 0.89902116

tHCTHhyb/cc-pVDZ_r7

N -2.57665970 -1.31987917 -0.00335293
C -3.77094312 -0.65328571 0.00489573
O -4.85381929 -1.25816252 0.00970571
N -3.75554428 0.74002040 0.00762050
C -2.63559893 1.57435724 0.00135950
O -2.76437609 2.79950728 0.00329527
C -1.36189334 0.85558670 -0.00617360
N -0.22061166 1.57522428 -0.01534445
C 0.94238569 0.84677882 -0.01393641
C 2.16976765 1.53029389 -0.01681135
C 3.41662453 0.85776656 -0.01119695
C 4.68830533 1.62935679 -0.03072272
C 3.42756848 -0.58819588 0.00532070
C 4.71192974 -1.34492485 0.02791860
C 2.19314912 -1.27441628 0.00551100
C 0.98384398 -0.58412299 -0.00623379
N -0.26835961 -1.29229529 -0.00830015
C -1.43183928 -0.58820193 -0.00494729
C -0.25255430 -2.74442942 -0.01523589
H -4.66240410 1.20197900 0.01307467
H 2.13797182 2.62261716 -0.02423057
H 5.28468652 1.38726045 -0.93389325
H 4.50979181 2.71300122 -0.00763737
H 5.33933028 1.35255400 0.82176028
H 5.30145204 -1.10403885 0.93509306
H 4.54262149 -2.43059471 0.00268732
H 5.36036603 -1.06732203 -0.82549100
H 2.21310955 -2.36435892 0.01690904
H 0.28497041 -3.10587603 -0.90909038
H -1.28925968 -3.09382117 -0.02459756
H 0.27017391 -3.11597150 0.88340829

tHCTHhyb/cc-pVDZ_T1

N -2.54246500 -1.29086700 0.00013800
C -3.77137600 -0.63586300 0.00022000
O -4.82431900 -1.26856600 0.00002100
N -3.76570800 0.74976300 0.00017700
C -2.64386600 1.57850500 0.00005600
O -2.76297100 2.80128100 0.00010500
C -1.36687700 0.85570100 0.00012000
N -0.21836100 1.58369300 0.00011200
C 0.92856400 0.85155900 0.00007600
C 2.16755200 1.52075600 0.00002500
C 3.39369700 0.84628600 -0.00002800
C 4.67889100 1.61700000 -0.00014500
C 3.41599200 -0.59053100 -0.00002000
C 4.72239100 -1.33363500 -0.00002000
C 2.20729300 -1.28022700 0.00002500
C 0.96701700 -0.60339700 0.00007100
N -0.22483100 -1.29184000 0.00011800
C -1.43342300 -0.56510800 0.00011800
C -0.24125200 -2.75292100 0.00015700
H -4.67372500 1.21060800 0.00013100
H 2.14009600 2.61341000 0.00002700
H 5.28970400 1.35878600 -0.88397700
H 4.49964900 2.70138500 0.00023800
H 5.29027900 1.35821400 0.88310700
H 5.32918000 -1.07485800 0.88593100
H 4.55974500 -2.42152700 -0.00042100
H 5.32956900 -1.07424000 -0.88551300
H 2.22745900 -2.37076300 0.00002000
H 0.27120300 -3.12984600 -0.89892100
H -1.28627100 -3.07656000 0.00024100
H 0.27134800 -3.12979000 0.89917300

tHCTHhyb/cc-pVDZ_T4

N -2.56818700 -1.31135700 0.14384000
C -3.73718500 -0.66560700 0.14647600
O -4.86087700 -1.15507500 0.40025300
N -3.74818900 0.71780700 -0.12875200
C -2.61829600 1.53156100 -0.00133000
O -2.83236700 2.77184900 0.05975700
C -1.36367000 0.84592600 0.03392600
N -0.21340400 1.56565000 0.06829500
C 0.94174900 0.84138900 0.04550200
C 2.18294200 1.51931500 0.05993700
C 3.40711500 0.84933300 0.02208100
C 4.70126100 1.61871200 0.03837500
C 3.41396600 -0.57227300 -0.03383800
C 4.71407600 -1.33076200 -0.08214000
C 2.19407700 -1.26549800 -0.04227000
C 0.96624000 -0.58927900 -0.00108300
N -0.25536000 -1.28126700 0.01438100
C -1.44079300 -0.59229800 0.04722900
C -0.24949700 -2.74194700 0.03422500
H -4.65791100 1.18744700 -0.14204200
H 2.14864900 2.61206300 0.09916700
H 5.30592800 1.40395900 -0.86100600
H 4.51979900 2.70317800 0.08098700
H 5.32257700 1.33738900 0.90762200
H 5.33937200 -1.11339300 0.80260700
H 4.54169000 -2.41708600 -0.12089800
H 5.31255400 -1.04617700 -0.96641900
H 2.21843800 -2.35571600 -0.08158900
H 0.23008300 -3.12656900 -0.88006400
H -1.28807900 -3.08280800 0.08414800
H 0.30748200 -3.10205800 0.91398200

TPSS/cc-pVDZ_S0

N -2.56966191 -1.31981885 -0.00024090
C -3.76863974 -0.63722268 -0.00014787
O -4.86553501 -1.20517868 -0.00095438
N -3.74114987 0.78103945 0.00154266
C -2.62610148 1.60100261 0.00021692
O -2.70142015 2.83121628 -0.00073944
C -1.34725678 0.83233180 0.00015218
N -0.22058095 1.51558079 0.00014262
C 0.94974801 0.80381370 0.00009402
C 2.17776073 1.51195146 0.00006026
C 3.40233325 0.85622672 -0.00002007
C 4.69706272 1.63135086 -0.00033791
C 3.41183845 -0.57811004 -0.00002936
C 4.72263577 -1.32164400 0.00012100
C 2.21095096 -1.29454247 0.00000033
C 0.97011970 -0.62588731 0.00006343
N -0.23954881 -1.30673982 0.00011767
C -1.44356359 -0.62017079 0.00010538
C -0.24975752 -2.78210321 0.00005813
H -4.65466260 1.24162493 0.00119350
H 2.12060442 2.60449776 0.00012306
H 5.30725749 1.38769464 -0.88929968
H 4.50560809 2.71562667 0.00218222
H 5.31019511 1.38394108 0.88549984
H 5.32492328 -1.05441596 0.88781827
H 4.56594381 -2.41107706 -0.00215588
H 5.32740893 -1.05089178 -0.88475360
H 2.25080015 -2.38522342 -0.00008884
H 0.26496935 -3.14868646 -0.90156708
H -1.29929932 -3.09505593 0.00042241
H 0.26570057 -3.14874627 0.90122352

TPSS/cc-pVDZ_S1(r2)

N -2.57514519 -1.32331921 -0.00044655
C -3.69992845 -0.63041371 -0.00021343
O -4.84878950 -1.26144881 -0.00031399
N -3.78325347 0.72997095 0.00010777
C -2.62232683 1.57021620 0.00040253
O -2.79943718 2.79255119 0.00077281
C -1.37216953 0.84325175 0.00014616
N -0.19901893 1.57623926 0.00010343
C 0.94004307 0.84896038 -0.00004794
C 2.19710648 1.52620257 -0.00013724
C 3.41286296 0.85219592 -0.00000051
C 4.71475421 1.61770893 -0.00007741
C 3.41852859 -0.57903885 0.00029581
C 4.72001394 -1.34163085 0.00066280
C 2.19916035 -1.27285905 0.00025340
C 0.97144384 -0.59159080 -0.00000964
N -0.25300386 -1.28011773 -0.00027165
C -1.43075462 -0.57661612 -0.00019242
C -0.25461858 -2.75251006 -0.00062226
H -4.69191939 1.19828447 0.00030783
H 2.16376670 2.62030347 -0.00024200
H 5.32540414 1.36625298 -0.88695818
H 4.53462567 2.70426941 -0.00005276
H 5.32553712 1.36622657 0.88669912
H 5.33179274 -1.09042813 0.88740620
H 4.54368890 -2.42898025 0.00053997
H 5.33238854 -1.09030720 -0.88562648
H 2.22218182 -2.36467530 0.00043561
H 0.26731798 -3.11601266 -0.90020840
H -1.29759888 -3.08378077 -0.00142781
H 0.26603144 -3.11652098 0.89951625

TPSS/cc-pVDZ_r5/r6

N -2.59263168 -1.31109970 0.00033750
C -3.73950187 -0.63588932 -0.00024536
O -4.86319808 -1.24442813 -0.00017274
N -3.75079840 0.77068002 -0.00099600
C -2.61759023 1.51616490 -0.00103453
O -2.70185987 2.79973808 -0.00168119
C -1.36584189 0.80967737 -0.00041613
N -0.21093108 1.56645087 -0.00039818
C 0.93532797 0.84315957 -0.00007436
C 2.18743265 1.52534401 -0.00007949
C 3.40807948 0.85705053 0.00002525
C 4.70596486 1.62964545 -0.00013854
C 3.41792167 -0.57182822 0.00013409
C 4.72143413 -1.33056385 0.00014143
C 2.19939580 -1.27185080 0.00028726
C 0.96757167 -0.60062159 0.00023614
N -0.25059366 -1.30173261 0.00055152
C -1.43688525 -0.59644096 0.00015001
C -0.25684836 -2.76962314 0.00131436
H -4.65892780 1.24279849 -0.00162400
H 2.14929305 2.61930261 -0.00022608
H 5.31765847 1.38311802 -0.88773762
H 4.51961257 2.71522670 0.00088988
H 5.31890923 1.38157733 0.88613995
H 5.33234599 -1.07742241 0.88701151
H 4.54905870 -2.41850475 -0.00072104
H 5.33316415 -1.07608986 -0.88576114
H 2.22749244 -2.36372977 0.00039450
H 0.25837124 -3.14316343 -0.89883094
H -1.30420448 -3.09240652 0.00208632
H 0.25946257 -3.14215537 0.90123862

TPSS/aug-cc-pVDZ_S0

N -2.56957524 -1.31865602 -0.00022739
C -3.76010151 -0.63908228 -0.00017303
O -4.86553889 -1.20747881 0.00003418
N -3.74160492 0.77695856 -0.00011507
C -2.62756435 1.59448554 0.00014880
O -2.71261752 2.82840016 0.00064621
C -1.34825248 0.83233242 -0.00002163
N -0.21930605 1.51227848 0.00004598
C 0.95034291 0.80723875 -0.00004754
C 2.17879361 1.51595008 -0.00010037
C 3.40020095 0.85728138 -0.00012078
C 4.69757743 1.62855133 -0.00065771
C 3.40823681 -0.57782186 0.00011189
C 4.71964330 -1.32015252 0.00078976
C 2.20832981 -1.29408356 0.00005852
C 0.97009381 -0.62294183 -0.00009003
N -0.23922400 -1.30209418 -0.00019502
C -1.43863475 -0.61760778 -0.00017860
C -0.24092519 -2.77955374 -0.00017231
H -4.65306212 1.23447700 0.00032418
H 2.12754002 2.60680613 -0.00010125
H 5.30259401 1.38302266 -0.88914164
H 4.50710425 2.71003652 0.00258111
H 5.30652495 1.37819412 0.88371711
H 5.31691862 -1.05262792 0.88834490
H 4.56143267 -2.40641173 -0.00270056
H 5.32134196 -1.04728811 -0.88205256
H 2.24642245 -2.38263221 0.00013358
H 0.27652008 -3.13611038 -0.90049597
H -1.28211439 -3.10570732 -0.00062302
H 0.27555820 -3.13611314 0.90072951

TPSS/aug-cc-pVDZ_S1(r1)

N -2.54341045 -1.30579406 -0.00306485
C -3.77006166 -0.64717603 -0.00124727
O -4.83096690 -1.30199223 -0.00392539
N -3.77796287 0.73563729 0.00353525
C -2.64438477 1.57645531 0.00522314
O -2.79997443 2.81404128 0.00941376
C -1.37505482 0.87211472 0.00167187
N -0.21664844 1.59340063 0.00025516
C 0.93932668 0.85366931 -0.00107080
C 2.17722946 1.52002347 -0.00336924
C 3.41563907 0.83729169 -0.00179833
C 4.70024135 1.61322315 -0.00425373
C 3.43046935 -0.58950953 0.00196924
C 4.73261324 -1.34924065 0.00588545
C 2.20946870 -1.27295878 0.00261587
C 0.97221449 -0.58350308 0.00055486
N -0.23521435 -1.27205224 -0.00148652
C -1.42112330 -0.56131053 -0.00120942
C -0.23733674 -2.74855101 -0.00440613
H -4.68683773 1.19598292 0.00537813
H 2.16111496 2.61209031 -0.00579921
H 5.30889555 1.35478768 -0.88852406
H 4.51602558 2.69494151 -0.00445405
H 5.31227292 1.35538956 0.87776928
H 5.33552285 -1.09602523 0.89422452
H 4.55363875 -2.43272745 0.00557229
H 5.34129475 -1.09622062 -0.87846931
H 2.22089843 -2.36254778 0.00470415
H 0.28454681 -3.10216580 -0.90413026
H -1.27537268 -3.08166643 -0.00780866
H 0.27959226 -3.10595885 0.89669526

TPSS/aug-cc-pVDZ_r7

N -2.57801681 -1.32233470 -0.00047578
C -3.77380541 -0.65569841 0.01579387
O -4.86952516 -1.26836720 0.02408470
N -3.76340349 0.74105716 0.02276016
C -2.63603921 1.57481453 0.01305845
O -2.77464699 2.81521613 0.01909186
C -1.36297880 0.86158384 -0.00275176
N -0.22039444 1.58224417 -0.01726777
C 0.95415569 0.84368098 -0.01534202
C 2.17884094 1.53177302 -0.01478873
C 3.42953037 0.85751411 -0.00524704
C 4.70268586 1.63159251 -0.02988386
C 3.43753562 -0.59030121 0.01513222
C 4.72105746 -1.35304964 0.04785941
C 2.19992692 -1.27798950 0.01060293
C 0.99087093 -0.58165832 -0.00801545
N -0.26346603 -1.29274425 -0.01460455
C -1.43127547 -0.58792674 -0.00373312
C -0.24494580 -2.75676904 -0.03429702
H -4.67052073 1.20512193 0.03373612
H 2.15215354 2.62305042 -0.02200404
H 5.28628666 1.40176562 -0.94368561
H 4.52011667 2.71212723 0.00803880
H 5.36364153 1.34289342 0.80940083
H 5.29510467 -1.12074239 0.96596408
H 4.54550959 -2.43521169 0.00983811
H 5.38081937 -1.06535619 -0.79173649
H 2.21714920 -2.36669536 0.02563152
H 0.30030428 -3.09799954 -0.92781349
H -1.27811962 -3.10609257 -0.05643951
H 0.26810470 -3.12584979 0.86754420

TPSS/aug-cc-pVDZ_r8

N -2.57992860 -1.28537812 0.00005840
C -3.80893701 -0.68936929 0.00076404
O -4.88173251 -1.31953093 0.00017012
N -3.78654426 0.71238070 0.00131376
C -2.65713592 1.55535594 0.00060080
O -2.78084226 2.79749971 0.00099311
C -1.40232376 0.83820082 -0.00047140
N -0.20664857 1.49399575 -0.00178284
C 0.97497413 0.87881706 -0.00113254
C 2.22869058 1.55125729 -0.00167445
C 3.43646468 0.85927541 -0.00063750
C 4.74426681 1.61827742 -0.00043180
C 3.43911793 -0.56676398 0.00100942
C 4.73276710 -1.34054409 0.00074461
C 2.20535724 -1.24293157 0.00339520
C 0.98350015 -0.57607613 0.00057789
N -0.24566435 -1.26227311 0.00007033
C -1.43094927 -0.57576297 0.00017222
C -0.26279802 -2.73679038 -0.00184720
H -4.69723294 1.17051957 0.00183354
H 2.20762753 2.64259938 -0.00183661
H 5.34561876 1.36085476 -0.88795005
H 4.56906588 2.70225768 0.00128037
H 5.34645814 1.35817449 0.88573483
H 5.34210296 -1.09555324 0.88729789
H 4.54401304 -2.42255238 -0.00091234
H 5.34288953 -1.09292336 -0.88448363
H 2.20124274 -2.33715901 0.00482562
H 0.24342842 -3.10611751 -0.90465512
H -1.30985374 -3.06110859 -0.00443668
H 0.23966159 -3.10898278 0.90186103

TPSSh/cc-pVDZ_S0

N -2.56191408 -1.31411227 -0.00010107
C -3.75617039 -0.63315298 0.00000841
O -4.84782649 -1.19801025 -0.00029479
N -3.73031227 0.77895939 0.00097512
C -2.61999233 1.59457698 0.00007737
O -2.69319321 2.81852660 -0.00056352
C -1.34289744 0.82881931 0.00004815
N -0.22177720 1.50740592 0.00004592
C 0.94659761 0.80034613 0.00003577
C 2.17079068 1.50811555 0.00005538
C 3.39166530 0.85486365 0.00003750
C 4.68334553 1.62858493 -0.00019036
C 3.40092393 -0.57563552 0.00002794
C 4.70886970 -1.31749753 0.00025599
C 2.20408965 -1.29020922 -0.00001981
C 0.96592586 -0.62348681 -0.00001583
N -0.24012354 -1.30296487 -0.00005732
C -1.43816238 -0.62097661 0.00002527
C -0.24798983 -2.77329755 -0.00024065
H -4.64094446 1.23721201 0.00080466
H 2.11318664 2.59796151 0.00011214
H 5.29190981 1.38636556 -0.88705147
H 4.49150891 2.71001643 0.00208607
H 5.29447595 1.38297171 0.88391611
H 5.30912691 -1.05142152 0.88603193
H 4.55207991 -2.40414155 -0.00185778
H 5.31154197 -1.04817563 -0.88284113
H 2.24407422 -2.37817623 -0.00009736
H 0.26549064 -3.13901502 -0.89957573
H -1.29344671 -3.08975927 0.00002733
H 0.26606811 -3.13928802 0.89864228

TPSSh/cc-pVDZ_S1(r1)

N -2.53517370 -1.30114094 -0.00005395
C -3.76371588 -0.64289006 -0.00006196
O -4.81384187 -1.29029501 -0.00019849
N -3.76292879 0.73763837 0.00036967
C -2.63525085 1.57667322 0.00028370
O -2.77727796 2.80315470 0.00076077

C -1.36853938 0.86498602 0.00010443
N -0.21359368 1.58795204 0.00011224
C 0.93329445 0.85163920 0.00005476
C 2.17080910 1.51595171 0.00004527
C 3.40242543 0.83778534 0.00003476
C 4.68461494 1.61261262 -0.00012146
C 3.41935926 -0.58891254 0.00005738
C 4.71896091 -1.34493815 0.00021471
C 2.20333634 -1.27246624 0.00000361
C 0.96817247 -0.58889501 -0.00001928
N -0.23484481 -1.27415601 -0.00014568
C -1.41895613 -0.56170891 -0.00005466
C -0.24482688 -2.74305700 -0.00039871
H -4.67122540 1.19857907 0.00050271
H 2.14831970 2.60703443 0.00006191
H 5.29546969 1.35822171 -0.88348384
H 4.50069311 2.69459231 0.00096291
H 5.29685508 1.35656973 0.88176748
H 5.32640270 -1.09391034 0.88613119
H 4.54369863 -2.42920165 -0.00077631
H 5.32756445 -1.09242319 -0.88445591
H 2.21809488 -2.36143719 -0.00001727
H 0.27284692 -3.10711266 -0.89889842
H -1.28660712 -3.06801699 -0.00048560
H 0.27278740 -3.10742404 0.89800804

TPSSh/cc-pVDZ_r7

N -2.57244972 -1.32029443 -0.00465243
C -3.76795791 -0.65386143 0.00384209
O -4.85342140 -1.25808055 0.00846506
N -3.74912544 0.74134942 0.00706321
C -2.62725390 1.57462607 0.00110598
O -2.75555035 2.80373612 0.00325349
C -1.35778477 0.85581512 -0.00682316
N -0.21791019 1.57865184 -0.01627750
C 0.94702839 0.84431385 -0.01448831
C 2.17108787 1.52704960 -0.01668042
C 3.41817899 0.85548137 -0.01068358
C 4.68806521 1.62936613 -0.03159508
C 3.42836712 -0.58952881 0.00648224
C 4.71035413 -1.34961516 0.03105058
C 2.19436833 -1.27404867 0.00591459
C 0.98734617 -0.58298609 -0.00682084
N -0.26504086 -1.29239040 -0.00971554
C -1.42977596 -0.58694426 -0.00604028
C -0.25092947 -2.74920604 -0.01819425
H -4.65591736 1.20537228 0.01244212
H 2.14031736 2.61738664 -0.02415919
H 5.27926894 1.39286199 -0.93765117
H 4.50532230 2.71033285 -0.00381408
H 5.34091660 1.35112972 0.81669624
H 5.29474916 -1.11346236 0.94074806
H 4.53595342 -2.43248591 0.00175366
H 5.36055765 -1.07099529 -0.81836258
H 2.21223391 -2.36224028 0.01771592
H 0.28591373 -3.10627120 -0.91133369
H -1.28792277 -3.09116727 -0.02969861
H 0.26793581 -3.11848930 0.88076446

TPSSh/cc-pVDZ_r8

N -2.57366369 -1.27535950 -0.00006955
C -3.80422773 -0.68505717 -0.00004731
O -4.86289595 -1.31445120 -0.00000652
N -3.77611612 0.71387132 0.00024594
C -2.65146338 1.55445515 0.00019679
O -2.76463555 2.78427437 0.00044199
C -1.39538232 0.83307455 0.00011357
N -0.20700267 1.49708769 0.00012995
C 0.97078530 0.87348307 0.00007150
C 2.22071391 1.54361930 0.00011329
C 3.42848958 0.85663385 0.00005236
C 4.73033252 1.61767536 0.00002023
C 3.43056302 -0.56449990 -0.00004372
C 4.72069294 -1.33795575 -0.00001710
C 2.20087061 -1.23961180 -0.00007946
C 0.97980915 -0.57503014 -0.00003459
N -0.24610486 -1.26363401 -0.00008214
C -1.42608784 -0.57543730 -0.00000347
C -0.27027350 -2.73043098 -0.00024595
H -4.68642107 1.17275047 0.00034329
H 2.19609522 2.63435330 0.00020709
H 5.33504395 1.36371452 -0.88616970
H 4.55443905 2.70173398 0.00096837
H 5.33600128 1.36227215 0.88512176
H 5.33194895 -1.09484353 0.88556203
H 4.53364595 -2.42044378 -0.00110750
H 5.33303096 -1.09319323 -0.88436891
H 2.19888894 -2.33300528 -0.00015128
H 0.23045100 -3.11155895 -0.90173497
H -1.32108283 -3.04731883 -0.00026303
H 0.23047824 -3.11176317 0.90114102

TPSSh/cc-pVDZ_T1

N -2.53623300 -1.29942900 -0.00026100
C -3.76519100 -0.64368600 -0.00004100
O -4.82000500 -1.28079300 -0.00022900
N -3.75976600 0.74106700 0.00040700
C -2.63599500 1.57029500 0.00022900
O -2.75827000 2.79642300 0.00028600
C -1.36289800 0.84995100 0.00003000
N -0.21481200 1.58194900 -0.00007200
C 0.93293400 0.84664700 -0.00008100
C 2.16951400 1.51687900 -0.00016100
C 3.39529500 0.84482600 -0.00007200
C 4.67896200 1.61914700 -0.00048900
C 3.41872900 -0.58970900 0.00017400
C 4.72426600 -1.33490200 0.00061900
C 2.21162900 -1.27932600 0.00017900
C 0.97301300 -0.60321100 -0.00000600
N -0.21980300 -1.29426800 -0.00021200
C -1.42766400 -0.56761100 -0.00011100
C -0.23436600 -2.75997900 -0.00019400
H -4.66807500 1.20310300 0.00044700
H 2.14074400 2.60759600 -0.00025400
H 5.28706600 1.36580300 -0.88542800
H 4.49404000 2.70092300 0.00196800
H 5.29027700 1.36208200 0.88108000
H 5.32831700 -1.07917900 0.88726600
H 4.55726800 -2.42036200 -0.00167600
H 5.33105800 -1.07572500 -0.88309000
H 2.23105300 -2.36813500 0.00034400
H 0.27908300 -3.13100900 -0.89848500
H -1.27815100 -3.08031400 -0.00057200
H 0.27845400 -3.13098700 0.89846400

TPSSh/cc-pVDZ_T4

N 0.07771400 0.71855900 -0.48019800
C -0.28212900 2.03772700 -0.50434900
O -1.45405800 2.41895600 -0.59676800
N 0.73878700 3.00243200 -0.41857900
C 2.10174900 2.77109800 -0.30956300
O 2.91792000 3.69691600 -0.24024700
C 2.44430400 1.35234900 -0.28817100
N 3.79335600 1.01911000 -0.18126300
C 4.04983600 -0.28949800 -0.16754900
C 5.42311600 -0.72777000 -0.06044600
C 5.81955100 -2.08159300 -0.03963600
C 7.27480400 -2.44762800 0.07603700
C 4.82979700 -3.05537000 -0.12806700
C 5.14899100 -4.51989700 -0.11711900
C 3.44295200 -2.63948100 -0.23529000
C 3.05043800 -1.30465900 -0.25326000
N 1.71276500 -0.93033900 -0.35458300
C 1.37218200 0.42221500 -0.37780700
C 0.66255400 -1.94880100 -0.43377100
H 0.43900500 3.97561500 -0.43815100
H 6.16945400 0.06600400 0.00641600
H 7.59728800 -3.05542500 -0.78514500
H 7.90612000 -1.55064900 0.12352100
H 7.45739000 -3.05003100 0.98118900
H 5.69397900 -4.79508900 0.80195400
H 4.24136100 -5.13371100 -0.18104400
H 5.80822700 -4.78248600 -0.96246700
H 2.69353500 -3.42758700 -0.30419000
H 0.82152900 -2.57470100 -1.32342300
H -0.28997400 -1.41872300 -0.50579700
H 0.68644500 -2.57448400 0.46995400

TPSSh/cc-pVDZ_T6

N 0.10555600 0.71471400 -0.56565800
C -0.25165400 2.00340800 -0.56615100
O -1.37051000 2.47352200 -0.88329300
N 0.71078400 2.97422500 -0.21410800
C 2.08962000 2.73617600 -0.24053600
O 2.83099700 3.75849900 -0.22595500
C 2.47397400 1.36007200 -0.26432300
N 3.79041400 1.03807900 -0.20364800
C 4.07736800 -0.29880800 -0.18341000
C 5.42246100 -0.71307300 -0.10432500
C 5.79483100 -2.05957400 -0.06518700
C 7.24672200 -2.44519700 0.02328200
C 4.78350700 -3.05569900 -0.10731500
C 5.14110400 -4.51807700 -0.06423600
C 3.44077600 -2.66421300 -0.19174500
C 3.07236900 -1.31225600 -0.23172100
N 1.72900700 -0.91962800 -0.33921100
C 1.39644800 0.41406900 -0.37569700
C 0.68762400 -1.94390900 -0.45117900
H 0.41154200 3.95393500 -0.22209600
H 6.18159500 0.07118000 -0.07004300
H 7.54503400 -3.07246200 -0.83381500
H 7.89233500 -1.55661900 0.04278900
H 7.44597800 -3.03898900 0.93155000
H 5.69420100 -4.76821500 0.85697000
H 4.24092100 -5.14691500 -0.10491800
H 5.79314300 -4.79584300 -0.90976500
H 2.67627200 -3.43966400 -0.22764000
H 0.87978500 -2.57246100 -1.33274500
H -0.27140000 -1.43191700 -0.55551000
H 0.68818400 -2.57130500 0.45182600

VSXC/cc-pVDZ_S0

N 0.08093001 0.72099805 -0.48297803
C -0.25696402 2.05479315 -0.50298004
O -1.41509910 2.45617517 -0.60152905
N 0.77546206 3.01965922 -0.40403203
C 2.13184115 2.79312220 -0.29730102
O 2.96135921 3.69386126 -0.22482902
C 2.46870518 1.34280410 -0.28286302
N 3.74028727 1.01939007 -0.18399201
C 4.04589429 -0.31286802 -0.16928301
C 5.40568737 -0.69903305 -0.06511700
C 5.77856042 -2.03494315 -0.04546900
C 7.21840054 -2.45859418 0.06397400
C 4.76108834 -3.03651322 -0.13133501
C 5.16960937 -4.48209132 -0.10748001
C 3.41324025 -2.68214319 -0.23427502
C 3.03743022 -1.32255509 -0.25533402
N 1.71359812 -0.93206007 -0.35695703
C 1.36185210 0.40334103 -0.37895803
C 0.68090805 -1.97450014 -0.44095903
H 0.46671303 3.98954029 -0.42483603
H 6.14963744 0.10106501 -0.00107600
H 7.51704855 -3.06952522 -0.80597706
H 7.88150255 -1.58416212 0.13033001
H 7.37507453 -3.09695722 0.95114907
H 5.71839439 -4.71422434 0.82242606
H 4.29814631 -5.14700537 -0.18847201
H 5.87267443 -4.70001534 -0.93075907
H 2.66881219 -3.47916125 -0.29823902
H 0.87907306 -2.60047619 -1.32411510
H -0.29340802 -1.48739711 -0.51386104
H 0.74253906 -2.60746919 0.45731303

VSXC/cc-pVDZ_S1(r2)/r6

N 0.07401329 0.72474653 -0.47362284
C -0.20024562 2.01289975 -0.48514042
O -1.44523005 2.40674357 -0.57760261
N 0.71284196 3.01739653 -0.40825029
C 2.11448581 2.76933489 -0.30385157
O 2.87438563 3.73342057 -0.24165872
C 2.45711145 1.36626137 -0.28510707
N 3.79631149 1.04528761 -0.18173556
C 4.06837077 -0.27538783 -0.16857349
C 5.42630062 -0.70314492 -0.06188286
C 5.78157541 -2.04472666 -0.04401483
C 7.21918854 -2.47917573 0.06897766
C 4.76564042 -3.04242944 -0.13380467
C 5.15772932 -4.49437698 -0.11196473
C 3.42097303 -2.66084128 -0.23843717
C 3.05996711 -1.30170217 -0.25643145
N 1.72071632 -0.90700426 -0.35890841
C 1.40099577 0.42292588 -0.37346101
C 0.69462197 -1.95080154 -0.44632431
H 0.41808231 3.99059702 -0.42707372
H 6.18667811 0.08185250 0.00626485
H 7.51575603 -3.09026428 -0.80208351
H 7.89067250 -1.61121021 0.14175388
H 7.36708406 -3.12435424 0.95324377
H 5.69579270 -4.74595597 0.82000399
H 4.27571387 -5.14591290 -0.19961969
H 5.85894250 -4.72809947 -0.93339782
H 2.66615044 -3.44856656 -0.30529356
H 0.90007125 -2.57676898 -1.32893111
H -0.28629742 -1.47893667 -0.52000141
H 0.76059879 -2.58875134 0.44911432

VSXC/cc-pVDZ_r5

N 0.06986333 0.74355369 -0.47559878
C -0.23584015 2.03589705 -0.49033104
O -1.44130482 2.43408891 -0.58057296
N 0.76170462 3.02075103 -0.40654979
C 2.07690990 2.72661546 -0.30832719
O 2.94277911 3.66945284 -0.23670082
C 2.43907072 1.33879170 -0.28933933
N 3.78335904 1.04897561 -0.18752846
C 4.06298601 -0.27447993 -0.17250822
C 5.42068314 -0.69611078 -0.06703727
C 5.78235753 -2.03765401 -0.04570843
C 7.22207176 -2.46590048 0.06646980
C 4.76998793 -3.03608092 -0.13097002
C 5.16481477 -4.48701496 -0.10466322
C 3.42186867 -2.65825393 -0.23548943
C 3.05204774 -1.30289040 -0.25732303
N 1.70809766 -0.92270019 -0.36067894
C 1.38236610 0.41452701 -0.37682639
C 0.68404155 -1.96242885 -0.44528937
H 0.46563300 3.99490073 -0.42367449
H 6.17860736 0.09152440 -0.00192259
H 7.52148184 -3.07588732 -0.80446653
H 7.88982408 -1.59488503 0.13814092
H 7.37411148 -3.10965341 0.95111622
H 5.70130557 -4.73461894 0.82938195
H 4.28485170 -5.14119095 -0.19286903
H 5.86940433 -4.72072976 -0.92328402
H 2.66892171 -3.44833298 -0.29940764
H 0.88100496 -2.59368176 -1.32692545
H -0.29473522 -1.48293862 -0.52045870
H 0.74072297 -2.60059043 0.45152772

VSXC/aug-cc-pVDZ_S0

N 0.08153217 0.72428892 -0.48303461
C -0.25191009 2.05132714 -0.50296734
O -1.41723754 2.45784463 -0.59906674
N 0.77388955 3.02069790 -0.40924159
C 2.12798343 2.79071581 -0.29906887
O 2.95397587 3.70137607 -0.22263105
C 2.46832335 1.34353748 -0.28321533
N 3.73935240 1.01425867 -0.18376760
C 4.04799516 -0.31384156 -0.16863733
C 5.40930445 -0.69934617 -0.06417161
C 5.78028384 -2.03511545 -0.04449710
C 7.22216553 -2.45594488 0.06605068
C 4.76073659 -3.03714860 -0.13120842
C 5.16588170 -4.48483608 -0.10853360
C 3.41282666 -2.68367196 -0.23438840
C 3.03826252 -1.32389493 -0.25498323
N 1.71515503 -0.93090537 -0.35707044
C 1.36704113 0.40141644 -0.37905296
C 0.68125942 -1.97671588 -0.44115179
H 0.46613000 3.98814341 -0.42775290
H 6.15629730 0.09605089 0.00004631
H 7.51910154 -3.06803311 -0.80040536
H 7.87965514 -1.58001863 0.12706808
H 7.37877019 -3.08609340 0.95596632
H 5.71582667 -4.71596217 0.81795012
H 4.29266411 -5.14368088 -0.18526605
H 5.86143132 -4.70294198 -0.93482874
H 2.66847052 -3.47809236 -0.29901222
H 0.88159534 -2.59681035 -1.32433001
H -0.29366315 -1.49875691 -0.51330122
H 0.74589637 -2.60479303 0.45668879

VSXC/aug-cc-pVDZ_S1(r1)

N 0.10778067 0.71586508 -0.47483899
C -0.26295532 2.05523075 -0.49900534
O -1.45925639 2.37300478 -0.59288647
N 0.72331924 3.02023312 -0.41649278
C 2.10544768 2.78947463 -0.30749754
O 2.89111146 3.74977409 -0.24313759
C 2.47681842 1.38787684 -0.28194854
N 3.79721245 1.06797286 -0.17731394
C 4.07067954 -0.27442581 -0.16520194
C 5.40849603 -0.69452539 -0.06101619
C 5.77520977 -2.05811613 -0.04300184
C 7.21071387 -2.46558416 0.06951207
C 4.76771871 -3.06217080 -0.13265862
C 5.16069635 -4.51380450 -0.11407041
C 3.42917623 -2.67271504 -0.23539325
C 3.06488967 -1.30046740 -0.25250162
N 1.73628331 -0.91647128 -0.35402322
C 1.41780444 0.42674440 -0.37224281
C 0.70066087 -1.95908260 -0.43810169
H 0.42248339 3.98868623 -0.43810124
H 6.17895360 0.07815255 0.00761483
H 7.50247284 -3.08915213 -0.79289598
H 7.87354886 -1.59492314 0.13224988
H 7.35992339 -3.10760602 0.95452713
H 5.70477533 -4.76102118 0.81213141
H 4.27803104 -5.16044302 -0.19212565
H 5.84871355 -4.74452652 -0.94373390
H 2.66718388 -3.45058306 -0.30355307
H 0.90039742 -2.57964413 -1.32190572
H -0.27554520 -1.48551259 -0.50921965
H 0.76624931 -2.58918865 0.45901813

VSXC/aug-cc-pVDZ_r7

N 0.07093059 0.72919807 -0.47800314
C -0.27221040 2.05194971 -0.50190510
O -1.46314941 2.42198610 -0.59617420
N 0.73498373 3.01367876 -0.41845178
C 2.10920302 2.78331134 -0.30920577
O 2.90561862 3.73547874 -0.24384030
C 2.47721005 1.37358999 -0.28199833
N 3.78691152 1.06167690 -0.17589642
C 4.07322584 -0.29215362 -0.16149748
C 5.41818235 -0.68887008 -0.05743407
C 5.79802186 -2.05513499 -0.04061872
C 7.22507167 -2.45707201 0.07150968
C 4.77311636 -3.06630758 -0.13315008
C 5.15060513 -4.50808335 -0.11805363

C	3.41892033	-2.66889035	-0.23663812
C	3.07914350	-1.31247412	-0.24946928
N	1.70130927	-0.90792311	-0.35493471
C	1.39259220	0.41721540	-0.37381790
C	0.69026820	-1.95612143	-0.43547842
H	0.43697243	3.98325366	-0.44071938
H	6.18098522	0.09064547	0.01038250
H	7.52479639	-3.09028983	-0.78528995
H	7.88872947	-1.58806457	0.13584224
H	7.38229504	-3.11099229	0.95046774
H	5.70879014	-4.76076555	0.80253394
H	4.27008278	-5.15562557	-0.19731917
H	5.85263777	-4.74149307	-0.93991551
H	2.66284961	-3.45128398	-0.30644249
H	0.89732471	-2.58332858	-1.31650178
H	-0.29520903	-1.49996358	-0.50435368
H	0.76878535	-2.59409469	0.45855879

VSXC/aug-cc-pVDZ_r8

N	0.09184601	0.75983685	-0.47110169
C	-0.32175243	2.05921371	-0.50895226
O	-1.50665643	2.40593488	-0.60827870
N	0.69991969	3.01409849	-0.42272931
C	2.07982464	2.78606641	-0.30755891
O	2.89110323	3.72494571	-0.23730254
C	2.42932464	1.38553485	-0.28471169
N	3.72863768	0.99049189	-0.17755635
C	4.11275183	-0.28239832	-0.15407144
C	5.46657402	-0.70947903	-0.05601179
C	5.80457943	-2.05872251	-0.04267109
C	7.24391699	-2.49515141	0.07280477
C	4.78977403	-3.05168127	-0.13315034
C	5.16470690	-4.50824030	-0.11657061
C	3.44617658	-2.64810503	-0.23546040
C	3.07733028	-1.30240180	-0.24611993
N	1.73530317	-0.89944687	-0.35037518
C	1.39708117	0.42571500	-0.37265274
C	0.69706096	-1.93679320	-0.43398984
H	0.39701203	3.98253532	-0.44766685
H	6.23815213	0.06071694	0.00710376
H	7.52996211	-3.11514981	-0.79194369
H	7.91411326	-1.62883940	0.13294323
H	7.38690259	-3.12588558	0.96471461
H	5.70940939	-4.76368529	0.80740616
H	4.27470528	-5.14543008	-0.19226758
H	5.84804737	-4.74813501	-0.94786635
H	2.67217236	-3.41888287	-0.31078686
H	0.87989364	-2.55445485	-1.32425257
H	-0.28223456	-1.45892469	-0.50060886
H	0.75335635	-2.57023094	0.46187053

wB97XD/cc-pVDZ_S0

N	0.11029743	0.71351746	-0.48485606
C	-0.23243046	2.04166985	-0.50693630
O	-1.38505444	2.42596648	-0.60529996
N	0.78731789	3.00638205	-0.41079274
C	2.13421704	2.77856284	-0.29871224
O	2.95298509	3.67343308	-0.22110732
C	2.47922329	1.32573753	-0.28271127
N	3.72885732	0.99665811	-0.18365325
C	4.04054291	-0.33315991	-0.16815245
C	5.39561760	-0.71210842	-0.06427137
C	5.77908536	-2.03615761	-0.04316893
C	7.22859181	-2.42224681	0.06840653
C	4.76786888	-3.03773764	-0.12915972
C	5.15244602	-4.49014656	-0.10944422
C	3.43127478	-2.68072627	-0.23156680
C	3.04426876	-1.32994386	-0.25322391
N	1.71896037	-0.94218698	-0.35587414
C	1.37509126	0.38075839	-0.37983548
C	0.67336931	-1.96203322	-0.44031135
H	0.48189149	3.97543328	-0.42937639
H	6.13253317	0.09038108	-0.00121132
H	7.54644281	-3.02885497	-0.79382257
H	7.87076729	-1.53346550	0.11720624
H	7.41066963	-3.02735025	0.97028837
H	5.69806023	-4.73749240	0.81443420
H	4.27211098	-5.14151276	-0.17662730
H	5.82483411	-4.72754936	-0.94859530
H	2.68417772	-3.46946982	-0.29616978
H	0.83418161	-2.58208433	-1.33155672
H	-0.28794997	-1.45062882	-0.51085057
H	0.69874735	-2.59059098	0.45913792

wB97XD/cc-pVDZ_S1(r1)/r5

N	0.14490819	0.70801782	-0.47708463
C	-0.24238457	2.04517563	-0.50575545
O	-1.41776889	2.35785014	-0.60236089
N	0.74862516	3.00268703	-0.42049914
C	2.11503687	2.77399444	-0.30829949
O	2.90321380	3.70743226	-0.24063266
C	2.48279287	1.35992073	-0.28185365
N	3.78959670	1.04032184	-0.17507505
C	4.06771659	-0.28527801	-0.16241832
C	5.40262856	-0.70237923	-0.05756410
C	5.77317144	-2.04407377	-0.04107991
C	7.21520320	-2.42811394	0.07287869
C	4.76497353	-3.05824359	-0.13361628
C	5.14537511	-4.50964217	-0.11926567
C	3.43740005	-2.67802937	-0.23620606
C	3.05367501	-1.32034743	-0.25291081
N	1.74187984	-0.94524086	-0.35161490
C	1.42366953	0.41446711	-0.37259388
C	0.69010402	-1.95341022	-0.43202880
H	0.44849657	3.97294115	-0.44341820
H	6.15984670	0.07979974	0.01124301
H	7.52587358	-3.04122294	-0.78828937
H	7.86416496	-1.54552880	0.12610051
H	7.38524044	-3.04332291	0.97123553
H	5.68764470	-4.76960733	0.80395167
H	4.25970327	-5.15382371	-0.18936914
H	5.81501205	-4.75351310	-0.95970750
H	2.67690059	-3.45383539	-0.30590361
H	0.84345587	-2.57914846	-1.32222280
H	-0.27003731	-1.44032355	-0.50086827
H	0.71288549	-2.58447165	0.46741446

wB97XD/cc-pVDZ_r6

N	0.08597218	0.72281376	-0.31562198
C	-0.23433154	2.00591723	-0.35795124
O	-1.35178040	2.49953461	-0.09939379
N	0.76356576	2.96234889	-0.64388692
C	2.11293256	2.73518008	-0.37550491
O	2.87123652	3.70088567	-0.29410298
C	2.47075277	1.33211367	-0.28510828
N	3.76608376	1.03684756	-0.18007682
C	4.05641063	-0.30969399	-0.15854149
C	5.39572544	-0.71191237	-0.03860008
C	5.77915654	-2.05003809	-0.02306941
C	7.22847348	-2.42972463	0.11230535
C	4.78043820	-3.04422928	-0.13472539
C	5.14932454	-4.50300828	-0.12442402
C	3.44364654	-2.66171892	-0.25339828
C	3.06620523	-1.31470288	-0.27058106
N	1.71774182	-0.92976117	-0.39122689
C	1.39635569	0.39931155	-0.34159061
C	0.68583434	-1.94861355	-0.50558073
H	0.46597206	3.93652954	-0.73045827
H	6.14427614	0.07896958	0.04237185
H	7.56673046	-3.02700173	-0.74965402
H	7.86813014	-1.54017998	0.18348019
H	7.39799716	-3.04616142	1.00975759
H	5.67352329	-4.77531584	0.80563732
H	4.26070134	-5.14174871	-0.21378242
H	5.83063035	-4.74810065	-0.95495792
H	2.68862204	-3.44258932	-0.33432681
H	0.87368479	-2.57308563	-1.39035181
H	-0.28176276	-1.45355691	-0.60508602
H	0.67675494	-2.58625736	0.39063514

X3LYP/cc-pVDZ_S0

N	0.10393651	0.71778171	-0.48356242
C	-0.24266465	2.04610920	-0.50591976
O	-1.40068822	2.43148844	-0.60313829
N	0.78047768	3.01168595	-0.41167949
C	2.13142901	2.78318067	-0.30028788
O	2.95127233	3.68469900	-0.22391326
C	2.47612072	1.33256672	-0.28362967
N	3.73312490	0.99912609	-0.18444214
C	4.04539489	-0.32825416	-0.16845905
C	5.40125730	-0.71131968	-0.06428517
C	5.78474290	-2.03896721	-0.04292922
C	7.23405165	-2.42546620	0.06918312
C	4.77162160	-3.04246270	-0.12930393
C	5.15473198	-4.49478768	-0.10937552
C	3.43097353	-2.68376878	-0.23206761
C	3.04328909	-1.33307693	-0.25385919
N	1.71917252	-0.94315706	-0.35612511
C	1.37345142	0.38785753	-0.37954478
C	0.66872278	-1.96340701	-0.44049110
H	0.47636086	3.98241615	-0.43026075
H	6.13862611	0.09030832	-0.00120473
H	7.55519043	-3.03165813	-0.79353028
H	7.87647177	-1.53641866	0.12155310
H	7.41727107	-3.03579316	0.96863099
H	5.69935338	-4.74575019	0.81553738
H	4.27339366	-5.14527749	-0.18023201
H	5.83255317	-4.73464307	-0.94497008
H	2.68421121	-3.47181527	-0.29640398
H	0.83153268	-2.58782612	-1.32918609
H	-0.29003522	-1.44725152	-0.51301748
H	0.69364957	-2.59306509	0.45910023

X3LYP/cc-pVDZ_S1(r1)

N	0.13267721	0.70929126	-0.47548567
C	-0.25408727	2.04720155	-0.50344993
O	-1.43883373	2.35345805	-0.59792196
N	0.73435364	3.00747170	-0.42137485
C	2.11089840	2.78110440	-0.31091108
O	2.89252816	3.72820249	-0.24591601
C	2.48155379	1.37266829	-0.28342494
N	3.79271686	1.04672698	-0.17700739
C	4.07173642	-0.28337639	-0.16346038
C	5.40468801	-0.70347229	-0.05837224
C	5.77912145	-2.05603844	-0.04088316
C	7.22154904	-2.43419913	0.07425105
C	4.77381575	-3.06509332	-0.13326831
C	5.14620827	-4.51964875	-0.11909551
C	3.44209008	-2.67527774	-0.23621965
C	3.06391473	-1.31504425	-0.25267575
N	1.74290097	-0.93382387	-0.35205891
C	1.42453204	0.41755385	-0.37238241
C	0.68976592	-1.94806791	-0.43251286
H	0.43380118	3.97849454	-0.44404562
H	6.16525093	0.07531581	0.01060423
H	7.53788046	-3.04851360	-0.78606607
H	7.86916743	-1.55026314	0.13059141
H	7.39491624	-3.05537504	0.96982153
H	5.68376578	-4.78770707	0.80588935
H	4.25618925	-5.15839039	-0.19429111
H	5.81970517	-4.76999389	-0.95594558
H	2.67928489	-3.44792004	-0.30591346
H	0.85037671	-2.57654344	-1.31996039
H	-0.27068680	-1.43647873	-0.50356557
H	0.71721569	-2.57920787	0.46723706

X3LYP/cc-pVDZ_r6

N	0.08515981	0.72485815	-0.35932531
C	-0.25298632	2.01396846	-0.39466666
O	-1.40710505	2.47283739	-0.21928690
N	0.74846551	2.97876935	-0.62178196
C	2.08802973	2.73355221	-0.38038499
O	2.85967907	3.71044862	-0.26719709
C	2.46731174	1.34298021	-0.30714778
N	3.78390762	1.02733628	-0.19164833
C	4.06937430	-0.29684234	-0.16864875
C	5.41761433	-0.71452428	-0.04517438
C	5.79032202	-2.05223795	-0.02544862
C	7.23786802	-2.44263115	0.10689476
C	4.78325211	-3.04716688	-0.13172443
C	5.14635029	-4.50644546	-0.11594405
C	3.44353524	-2.66021417	-0.25039749
C	3.06745602	-1.31199641	-0.27100513
N	1.72630841	-0.92209187	-0.38706398
C	1.39853012	0.41397596	-0.36682958
C	0.68692598	-1.94321761	-0.48433618
H	0.45233409	3.95599794	-0.61785472
H	6.16872376	0.07329192	0.03531376
H	7.57203298	-3.04426644	-0.75475047
H	7.88370255	-1.55681203	0.17650099
H	7.40655549	-3.06181681	1.00369488
H	5.66903211	-4.78116395	0.81577088
H	4.25491590	-5.14194526	-0.20658658
H	5.83203214	-4.75922404	-0.94226127
H	2.68781085	-3.43901274	-0.33198872
H	0.86021284	-2.56834501	-1.37235956
H	-0.27909557	-1.44161275	-0.56251044
H	0.70074062	-2.58339570	0.41033390

X3LYP/cc-pVDZ_r7

N	0.08639862	0.72210818	-0.47935524
C	-0.26157792	2.03947676	-0.51121744
O	-1.44669517	2.39683228	-0.61095325
N	0.74150488	2.99872839	-0.42836072
C	2.11239899	2.77188920	-0.31195151
O	2.89987524	3.71598571	-0.24498462
C	2.48057615	1.36049628	-0.28199855
N	3.78534549	1.03971853	-0.17131150
C	4.07714123	-0.29520722	-0.15733428
C	5.41614174	-0.69836566	-0.05408970
C	5.80180786	-2.05608867	-0.03883085
C	7.23576888	-2.43044080	0.07987307
C	4.78062725	-3.07038408	-0.13744588
C	5.13766181	-4.51695885	-0.12927765
C	3.43630362	-2.66630636	-0.24049144
C	3.08614946	-1.32229085	-0.24793189
N	1.70789625	-0.91587696	-0.35012011
C	1.40564248	0.40645939	-0.37333560
C	0.68143202	-1.94217781	-0.42251750
H	0.44337615	3.97004423	-0.45351532
H	6.17179822	0.08469126	0.01463140
H	7.55750764	-3.05644021	-0.77305790
H	7.88481700	-1.54821068	0.13492935
H	7.41115325	-3.05595257	0.97525346
H	5.68765258	-4.79129843	0.78957493
H	4.24701628	-5.15342505	-0.20377260
H	5.81701065	-4.76842428	-0.96461224
H	2.67524017	-3.43943789	-0.31453484
H	0.84738806	-2.57347471	-1.31010052
H	-0.29082700	-1.45125886	-0.48435149
H	0.73246476	-2.58135662	0.47337624
